# Differential gene expression in the evolution of sex pheromone communication in New Zealand’s endemic leafroller moths of the genera *Ctenopseustis* and *Planotortrix*

**DOI:** 10.1186/s12864-018-4451-1

**Published:** 2018-01-26

**Authors:** Alessandro Grapputo, Amali H. Thrimawithana, Bernd Steinwender, Richard D. Newcomb

**Affiliations:** 1 Department of Biology, University of Padova, Padova, Italy; 2 The New Zealand Institute for Plant & Food Research Ltd, Auckland, New Zealand; 3 School of Biological Sciences, University of Auckland, Auckland, New Zealand

**Keywords:** Tortricids, Sex pheromones, Gene expression, Transcriptomics, RNAseq, Desaturases, Odorant receptors, Pheromone binding proteins

## Abstract

**Background:**

Sex pheromone communication in moths has attracted the attention of evolutionary biologists due to the vast array of pheromone compounds used, addressing questions of how this diversity arose and how male reception has evolved in step with the female signal. Here we examine the role of changing gene expression in the evolution of mate recognition systems in leafroller moths, particularly focusing on genes involved in the biosynthetic pathways of sex pheromones in female pheromone glands and the peripheral reception repertoire in the antennae of males. From tissue-specific transcriptomes we mined and compared a database of genes expressed in the pheromone glands and antennae of males and females of four closely related species of leafroller moths endemic to New Zealand, *Ctenopseutis herana* and *C. obliquana*, and *Planotortrix excessana* and *P. octo*. The peculiarity of this group, compared to other Lepidoptera, is the use of (*Z*)-5-tetradecenyl acetate, (*Z*)-7-tetradecenyl acetate, and (*Z*)-8-tetradecenyl acetate as sex pheromone components.

**Results:**

We identify orthologues of candidate genes from the pheromone biosynthesis pathway, degradation and transport, as well as genes of the periphery olfactory repertoire, including large families of binding proteins, receptors and odorant degrading enzymes. The production of distinct pheromone blends in the sibling species is associated with the differential expression of two desaturase genes, *deast5* and *desat7*, in the pheromone glands. In male antennae, three odorant receptors, OR74, OR76a and OR30 are over-expressed, but their expression could not be clearly associated with the detection of species-specific pheromones components. In addition these species contain duplications of all three pheromone binding proteins (PBPs) that are also differentially expressed among species.

**Conclusions:**

While in females differences in the expression of desaturases may be sufficient to explain pheromone blend differences among these New Zealand leafroller species, in males differential expression of several genes, including pheromone binding proteins, may underpin differences in the response by males to changing pheromone components among the species.

**Electronic supplementary material:**

The online version of this article (10.1186/s12864-018-4451-1) contains supplementary material, which is available to authorized users.

## Background

Moths rely on a sophisticated olfactory system for their survival and reproduction. Mate finding typically involves long-range attraction of males by sex pheromones emitted by females. Moth sex pheromones often consist of species-specific blends of modified long-chain unsaturated fatty acids with terminal functional groups as such alcohols, aldehydes, or acetate esters. Sex pheromone communication systems are considered to be under strong purifying selection to maintain the link between the females’ pheromone and the males’ ability to recognise only conspecific blends [1, 2]. This has created a conundrum of how new pheromone blends arise, with both gradual evolution and saltatory shifts being suggested to explain the resulting species diversity we see today [3].

One of the challenges in understanding sex pheromone evolution is determining how male preference can change in step with the female signal. Pheromone evolution might reflect selection on the receivers rather than on the emitters because of the differential investment of the two sexes [4]. Therefore, according to Phelan’s asymmetrical tracking hypothesis, selection on the female pheromone signal will be weak and females should have relatively large among-female variation in the pheromone blend. The corresponding response in males will track the female signals, but again selection should be weak. Males should show a wide breadth in pheromone responses to locate potential partners and maximise their fitness, this in turn will reduce the selection pressure on females [2, 4]. Still other hypotheses include variants of sexual selection with males choosing among variable blends produced by females that may act as proxies for beneficial traits or females striving to be different and detecting these differences themselves [5].

Notwithstanding these various hypotheses, the different phenotypes favoured by selection of male and female traits can be obtained through a number of potential molecular evolutionary mechanisms. With the genes involved in pheromone production in the female and recognition in the males becoming better understood (see below), the modes of molecular evolution can be explored. One mode might include structural mutation of important proteins, while a second might involve changes in gene expression, including differential gene expression in the two sexes. Evidence has been accumulating on the importance of gene expression differences in reproductive isolation and rapid divergence [6–9]. Certainly sex-biased expression is widespread among organisms and sex-biased genes show unusually rapid sequence evolution and are often labile in their pattern of expression [10]. Many examples of reproductive isolation involve gene expression differences in the olfactory system [11] causing shifts in sex pheromone preference in insect populations [12–14].

The composition of sex pheromones is likely under the control of a small number of genes [15] and recently substantial effort has been invested in identifying the genes involved in pheromone biosynthesis [2]. Moth sex pheromones are generally synthesized de novo in specialized pheromone glands, modified membranes between the 8th and 9th abdominal segments, by a battery of enzymes similar to those involved in fatty acid biosynthesis and other gland-specific activities [16–18]. Enzymes common to both pathways are acetyl-CoA carboxylase, fatty acid synthase, desaturases and specific chain-shortening enzymes. Gland-specific enzymes include reductases that reduce fatty acids to alcohols and acetyltransferases that acetylate fatty alcohols to generate acetate esters [16]. Most research has focused on the identification and characterization of desaturases [19–29], because the position of double bonds within the acyl chain underpins much of sex pheromone diversity. Most evidence has been found for differential gene expression between species (e.g. [27]), however some structural differences have also been described [29]. More recently fatty acid reductases (FARs) have also been targeted [30–34] with structural variants identified that differ in their ability to use various unsaturated substrates [33].

Pheromone reception in moths takes place within specialized hairs on the antennae, with sensilla each containing one to three olfactory sensory neurons (OSNs). Odorants penetrate the sensillum through pores binding to odorant binding proteins then receptors on OSN dendrites before finally being degraded to reset the system [35]. Four major families of membrane proteins are thought to be involved in odour reception in moths including odorant receptors (ORs), ionotropic receptors (IRs), and sensory neuron membrane proteins (SNMPs). Single amino acid changes responsible for specificity differences between species have been identified in pheromone sensing ORs [36]. Fewer differences have been found in gene families involved in transporting odorants to the receptors (e.g. odorant binding proteins and chemosensory proteins) or enzymes involved in resetting the system through hydrolysing odorants (e.g. carboxylesterases and glutathione-S-transferases).

Torticid leafroller moths of the sibling genera *Ctenopseustis* and *Planotortrix* are endemic to New Zealand and together with the Australian lightbrown apple moth, *Epiphyas postvittana*, form a complex of economically significant pests of fruit crops [37]. Both genera are widespread on the two islands and contain several closely-related species that are clearly differentiated by their sex pheromone composition [38]. The peculiarity of this group, compared to other members of the Tortricidae is the use of (*Z*)-5-tetradecenyl acetate (Z5–14:OAc), (*Z*)-7-tetradecenyl acetate (Z7–14:OAc), and (*Z*)-8-tetradecenyl acetate (Z8–14:OAc) as major sex pheromone components. Females of the sibling species *C. herana* and *C. obliquana* produce Z5–14:OAc and a blend of Z8–14:OAc and Z5–14:OAc in a 80:20 ratio, respectively [39]. Similarly, the sibling species *P. octo* and *P. excessana* produce a blend of Z8–14:OAc and 14:OAc (98:2) and a blend of Z5–14:OAc and Z7–14:OAc (60:40), respectively [40, 41]. These female sex pheromone differences are mirrored by male species-specific responses. For example, males of *C. obliquana* are attracted by a blend of 90:10 of Z8–14:OAc and Z5–14:OAc, whereas *C. herana* males are attracted by Z5–14:OAc alone [42, 43]. The characterization of the molecular components involved in odorant reception, as well as the biosynthetic pathway of pheromone production in these moths is well underway. Recent research has focused on the isolation and characterization of desaturases showing that the production of these distinctive sex pheromones in closely related species involves the differential regulation of the desaturase gene *desat5* [27, 44]. On the pheromone reception side two orthologous receptors, OR7 and OR30 display biased expression in male antennae compared to females in all four species. The receptor OR7 in particular shows signatures of positive selection and responds to Z8–14-OAc suggesting it acts as pheromone receptors in both genera [45, 46].

Here we use a combination of next generation transcriptome sequencing (RNAseq), bioinformatics and phylogenetic analyses to compare genes expressed in the antennae of both sexes and pheromone glands of *C. obliquana*, *C. herana*, *P. octo* and *P. excessana*. We identify orthologues of candidate genes of the pheromone biosynthesis pathway, as well as genes of the periphery olfactory repertoire, including large families of carrier proteins, receptors and odorant degrading enzymes. We use these data to compare modes of molecular evolution (sequence vs expression) in this mate recognition system.

## Methods

### Sampling and RNA sequencing

Four endemic species of New Zealand leafroller moths (*Ctenopseustis herena*, *C. obliquana*, *Planotortrix excessana* and *P. octo*) were obtained from the insect rearing facility at Plant & Food Research, Mount Albert Research Centre, Auckland, New Zealand. The history of the laboratory strains has been reported previously [47]. A fifth species, the light brown apple moths (*Epiphyas postvittana*), for which mined curated genes and gene models from a genome assembly were available [48, 49], was used as an outgroup in the evolutionary analyses.

Antennae were dissected from 100 2–3-day-old male and virgin female adult moths of each species. Pheromone glands were dissected from the 100 virgin adult females of each species. RNA was extracted from each pool of tissues using 800 μl of Trizol (Invitrogen, Carlsbad, CA, USA) following the manufacturer’s protocol. After DNase treatment (DNaseI amplification grade, Invitrogen) cDNA was synthetized using the iScript cDNA Synthesis Kit (Bio-Rad, Hercules, CA, USA) from 1 μg of total RNA, incubated at 25 °C for 5 min, 42 °C for 30 min and 85 °C for 5 min.

Pair-end RNAseq libraries were constructed from each male and female antennae and pheromone gland pools using Illumina’s standard protocols and sequenced at Macrogen (Seoul, South Korea). Raw sequence data are available in the NCBI-SRA archive under the bioproject numbers: PRJNA236627, PRJNA236626, PRJNA243920, PRJNA243922 and PRJNA236624 respectively for *C. herana*, *C. obliquana*, *P. excessana*, *P. octo* and *E. postivttana*. Quality of the raw reads pairs for each library was assessed using FastQC (version 0.11.2) [50]. Prior to assembly mitochondrial contamination was removed by mapping the reads to a set of reference mitochondrial genomes of Tortricidae downloaded from NCBI, as well as the mitochondrial genomes of *C. obliquana* and *P. octo* using bowtie2 (version 2.2.5) [51], where reads mapping to the references were discarded. Thereafter the reads were screened for adapters and trimmed to a quality score threshold of 20 using fastq-mcf from the ea-utils package (version 1.1.2–806) [52]. An in-house perl script was then used to trim 15 bases of their 5′ end and to discard reads containing Ns and mononucleotides. Finally, possible contaminants from bacteria and fungi were screened out using Kraken with the Refseq fungal and bacterial databases (version 0.10.5-beta) [53].

### Assembly and annotation

Assemblies were conducted independently for each species by combining sexes and tissues for each species. We conducted several assemblies for each species using SOAPdenovo-trans (version 1.04) [54] with kmer series of 31–75 with increments of four bases and Trinity (version 2.0.6) [55]. For *C. obliquana* and *P. octo* for which a partial draft genome sequence was available (unpublished data) we carried out both a de novo and a genome-guided assembly with Trinity. These two assemblies were combined using PASA [56]. Final optimized and cleansed transcriptome assemblies were obtained combining all SOAP and PASA assemblies with EvidentialGene, tr2aacds perl script [57]. The CDS sequences of the “okayset” of EvidentialGene were used for the subsequent analyses and from now on are referred to as genes. Assemblies are available in the NCBI-TSA archive under the same bioproject numbers indicated above.

Orthologous genes among the five species were identified using ProteinOrtho (version 5.11) [58] with default parameters.

Gene annotation was obtained by sequence similarity searches using NCBI blastx [59] against the non-redundant (nr) peptide database (including all non-redundant GenBank CDS translations + PDB + SwissProt + PIR+ PRF) with E-value 1e^− 4^ within the Blast2Go package (version 3.1). A maximum of 10 hits were considered for each query and the E-value of 1e^− 4^ was used to maximise the number of hits. Functional categories were assigned by mapping Gene Ontology (GO) terms using Blast2GO [60] with default parameters (E-values 1e^− 6^, annotation cut-offs > 55, GO weight > 5). InterProScan annotation was also conducted via Blast2GO. Information obtained for domains were included to improve global annotations.

### Evolutionary analysis

Sequences with blastx hits (cut off E-value 10^− 4^) to gene families of odorant binding proteins, ionotropic receptors, sensory neuron membrane proteins, chemosensory proteins, carboxylesterases, pheromone binding activating neuropeptides, elongation of very long chain fatty acids proteins (ELOVLs), fatty acyl reductase (FAR), acetyltransferases, fatty acid synthases (FAS), fatty acid transport proteins (FATP), fatty acid amide hydrolases (FAAH) and acetyl-coa carboxylases (ACC) were extracted from the data sets and codon based aligned with Clustal W [61] in Geneious ver. 8.1 (Biomatters). The presence of multiple contigs in orthologous groups identify by ProteinOrtho suggested the existence of duplicated genes. Sequences were therefore manually curated and short sequences and putative alleles excluded. A phylogenetic tree for each gene family was obtained with FastTree ver.2.1 [62] from the protein sequences using the JTT model of evolution. The Shimodaira-Hasegawa test was used to compute the local support values at the nodes.

Phylogenetic clusters were compared to the previous orthologous groups obtained with ProteinOrtho to confirm duplicated genes and each group, whenever possible, tested for evidence of positive selection using the Codeml program in the PAML package [63]. Nested models M0-M3, M1-M2 and M7-M8 were compared using a likelihood ratio test (LRT) as twice the log-likelihood difference between the nested models. Significance was assessed by a χ^2^ test with the difference in the number of parameters of the models as the degrees of freedom.

### Differential expression

The barcoded sequence library of each tissue (female antennae, male antennae and pheromone glands) of New Zealand endemic leafroller moths were individually aligned to the reference genes of their own species using Bowtie (version 2.2.5) [64] and counted using HTseq ver. 0.6.1p2 [65]. Each barcoded library was also mapped back to the curated genes and counted with HTseq. We used the Bioconductor package, DESeq2 (version 1.12.2) [66] to perform the differential expression analysis. We did not have RNAseq data available for the whole body of any of the species, therefore we assessed the biased expression relative to the other tissue (antennae or pheromone glands). Since we did not have within species replicates we applied the variance-stabilizing transformation to the raw read counts and then calculated the log fold change between pairs of samples within species using a threshold of 2 to consider a gene as biased expressed. Enrichment of GO terms in the biased expressed genes was assessed using Piano [67] an R package that collects several Gene Set Analyses (GSA) methods and allows consensus scoring of the results of the multiple GSA runs. These methods in Piano make use of the full data set as they do not require of a priori cut-off of gene significance. We used six methods for calculating the gene set statistics, mean, median, sum, maxmean, GSEA and PAGE using the log fold change as input.

## Results

### Transcriptome assemblies and orthologous genes

Illumina RNAseq produced an average of about 67.4 million reads (SE ± 3.6 million) per species and tissue (Table 1). After trimming and mitochondrial and contaminant removal, we were left with an average of 53.6 million (SE ± 3.5 million) reads per species and tissue, corresponding to an average of 79% (SE ± 2.76%) of the initial raw reads (Additional file 1: Table S1).

**Table 1:** Summary statistics of blastx hits and GO term annotations

	*C. herana*	*C. obliquana*	*P. execssana*	*P. octo*	*E. postvittana*		Orthologs groups		
							*OGs*	*OGs 5*	*OGs NZ*
Blastx						Blastx			
Tot N CDS	24,989	20,840	23,304	20,883	23,591	Tot N OG	18,447	6595	8977
CDS with Blastx	17,460	14,318	16,000	15,448	16,702	CDS with Blastx	16,165	6482	8702
%	69.87%	68.70%	68.66%	73.97%	70.80%	%	87.63%	98.29%	96.94%
Not in OGs	9853	6623	8384	6294	12,871				
%	39.43%	31.78%	35.98%	30.14%	54.56%				
GO terms						GO terms			
CDS with GO terms	12,499	11,358	11,331	11,052	11,094	CDS with GO terms	13,555	5570	7457
%	50.02%	54.50%	48.62%	52.92%	47.03%	%	73.48%	84.46%	83.07%
# of GO terms	54,633	50,298	48,888	49,018	35,900	# of GO terms	50,288	21,265	19,716
Biological Processes	20,606	18,559	18,578	18,497	12,253	Biological Processes	18,986	7627	7042
Molecular Functions	19,066	17,035	17,704	17,322	14,479	Molecular Functions	17,850	6923	6372
Cellular Components	11,240	10,263	9659	9606	6810	Cellular Components	10,116	4372	4064

*OGs* sequences present in at least two species, *OGs 5* sequences present in all five species, *OGs NZ* sequences present in all New Zealand endemic tortricids

The number of contigs (or genes according to Trinity) produced by different assembly algorithms and kmer sizes ranged from 10,638 (*P. octo*, SoapTrans k75) to 173,448 (*C. herana*, SoapTrans k31). The assembly statistics for the different algorithms and kmer sizes are reported in supplementary material (Additional file 2: Table S2). Scaffolds and genes obtained with SoapTrans and Trinity (both *denovo* and guided) were combined with the EvidentialGene tr2aacds.pl pipeline script into a set of optimized and cleansed transcriptome assemblies. These assembles consisted of 24,989, 20,840, 23,304, 20,883 and 23,591 coding sequences (CDS) for *C. herana*, *C. obliquana*, *P. excessana* and *P. octo*, respectively (Additional file 3: Table S3). These numbers are comparable with those found with the Evidentialgene method in *Nasonia vitripennis*, but higher than those found in *Bombyx mori*, see [57] for a comparative table. From 53% (*P. octo*: 11,055) to 58% (*P. excessana*: 13,566) of these CDS were considered full length by EvidentialGene.

For the CDS, 15,896 (63.61%), 14,318 (68.70%), 14,610 (62.69%) and 14,133 (67.68%) showed similarity (Blastx, E-value 1e^− 4^) to protein sequences in the NCBI non-redundant protein database. Despite the relative high E-value cut-off used about 30% of the contigs of each species remained without any hits. The number of genes assigned to functional groups (GO terms) for *C. herana*, *C. obliquana*, *P. excessana* and *P. octo* were 12,499, 11,358, 11,331 and 11,052, respectively (Table 1). For all species, the most abundant GO categories within Biological Process were the “macromolecule metabolic process” and the “cellular macromolecule metabolic process” both represented by 13–16% of the genes. The most abundant GO categories with Molecular function were “nuclear acid binding” and “cation binding” representing about 11% of the genes in both cases. Finally, within Cellular Components the most abundant GO categories were “intracellular membrane-bounded organelle” and “cytoplasm” represented by 9–10% and 7–8% of the genes, respectively. A complete list of blastx descriptions, best hits and GO annotations for each species is given in Additional file 4: Table S4 and the GO distribution in the three categories of each species is shown in the Additional file 5: Figure S1.

Comparing the translated coding sequences among the moth species, including *E. postvittana*, we identified 18,447 orthologous groups (Additional file 6: Table S5), of which 5644 showed a simple one to one relationship in all species, 184 had multiple orthologs in at least two of the species and in 11,852 cases orthologs were missing in at least one of the species. A significant blast hit (Blastx, E-value 1e^− 4^) was obtained for 16,165 (87.63%) of the groups. Out of the total number of orthologous groups, 6595 contained at least one sequence from each of the five moth species and 98.29% (6482) of these had a significant blast hit (Table 1). The proportion of coding sequences that were not assigned to any orthologous group ranged from 30 to 40% in the New Zealand leafroller moths, reaching 54% for *E. postivittana*.

### Biased gene expression

The Evidentialgene CDS sequence set for each species was used as a reference transcriptome for quantifying the expression levels of genes in female antennae, male antennae and pheromone glands. We mapped reads to CDS instead of transcripts to increase the accuracy of read assignment to putative genes and kept distinct putative CDSs found in long transcript UTRs. The gene expression values for each gene and species are given in Additional file 5: Table S4.

The number of expressed genes in the different tissues varied among species. In *C. herana* and *P. octo* the greatest number of expressed genes was found in the pheromone glands, whereas in *C. obliquana* and *P. excessana* the greatest number of expressed genes was in male antennae (Table 2). The difference in number of expressed genes among tissue and species was not related to sequence depth.

**Table 2:** Summary statistics of reads mapping to references and expression bias

	Ch_Fant	Ch_Mant	Ch_PG	Co_Fant	Co_Mant	Co_PG	Pe_Fant	Pe_Mant	Pe_PG	Po_Fant	Po_Mant	Po_PG	Ep_Fant	Ep_Mant
Mapping														
alignment_not_unique	0	0	0	0	0	0	0	0	0	0	0	0	0	0
ambiguous	226,743	194,577	346,698	55,850	104,348	193,117	110,969	115,212	169,597	36,251	62,890	118,739	385,785	421,537
no_feature	0	0	0	0	0	0	0	0	0	0	0	0	0	0
not_aligned	38,309,762	34,261,420	34,140,479	19,746,155	26,398,702	25,082,595	30,230,266	36,931,907	29,725,145	26,269,131	26,654,273	33,259,985	100,679,610	95,586,120
too_low_aQual	9,317,093	9,880,342	11,591,146	2,645,541	5,459,073	7,607,995	8,258,990	9,179,365	10,704,574	3,559,744	4,928,872	8,668,419	18,172,147	23,175,876
Sum of counts	15,920,614	16,858,439	20,289,017	6,630,706	13,768,913	20,582,121	11,553,029	15,484,897	22,889,282	8,567,174	11,906,235	24,475,076	19,763,985	27,284,785
Expression														
Genes	18,743	17,826	19,491	16,221	17,605	17,266	17,778	18,378	18,061	16,572	16,844	17,618	19,591	19,817
Genes w 0 counts	6246	7163	5498	4619	3235	3574	5526	4926	5243	4311	4039	3265	4000	3774
Sex biased gene	17	12	–	14	10	–	227	38	–	8	7	–	25	12
Tissue biased gene	1743	–	1743	1430	–	1778	556	–	987	1479	–	1855	–	–

*Ch Ctenpseustis herana*, *Co C. obliquana*, *Pe Planotortrix execssana*, *Po P. octo*, *Ep Epiphyas postvittana*, *Mant* male antennae, *Fant* female antennae, *PG* pheromone glands

In the comparison of gene expression between antennae of the two sexes we also included *E. postvittana.* Few genes were differentially expressed between male and female antennae, with the exception of *P. excessana* where 227 displayed greater expression in female antennae. In all species, male antennae biased genes were mostly olfactory receptors and pheromone binding proteins. Two olfactory receptors, OR7 and OR30, were the most male-biased in their expression in all five species (Additional file 7: Table S6). Two pheromone binding protein (PBP1 and 3) were also male biased in all species except in *P. excessana*. Finally, two lipases (OG3363 in the New Zealand moths and OG362 in *E. postvittana*) display greater expression in male antennae making them candidate sex pheromones degrading enzymes. The olfactory receptor OR4 was female biased in the antennae of all species. Another olfactory receptor (OR21) was biased in female antennae in all species except *E. postvittana*. Three female biased genes were shared between the *Ctenopseustis* species; a cytochrome p450 (OG4842), one hypothetical protein (OG11897) and an unknown transcript (OG15104). *Planotortrix* females also shared three antennal-biased genes; a cytochrome p450 (OG8571) a predicted protein (OG6394) and an unknown transcript (OG13098) (Additional file 7: Table S6). GSA analysis did not show common enriched GO terms for either male or female antennae among all species (Additional file 8: Table S7, Additional file 9: Figure S2).

**Fig. 1:**
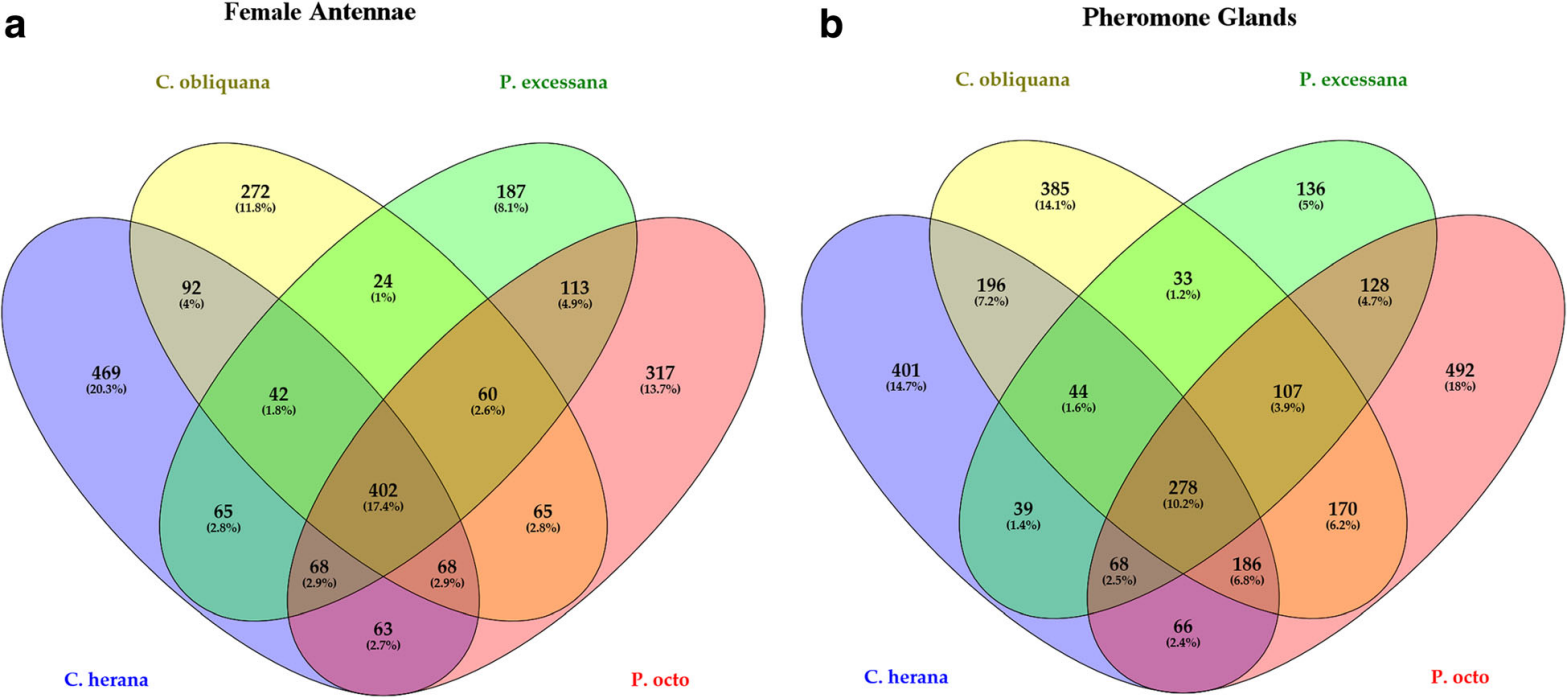
**a** Venn diagram showing the number of orthologous groups shared between female antennae of the four New Zealand leafroller moths, *Ctenopseustis herana*, *C. obliquana*, *Planotortrix excessana* and *P. octo*. **b** Venn diagram showing the number of orthologous groups shared between pheromone glands of the four New Zealand leafroller moths

No transcriptome is available for *E. postvittana* pheromone glands, therefore the comparison among pheromone gland transcriptomes only considered the four New Zealand endemic leafroller moths. Here, biased expression is relative to the other tissue (antennae or pheromone glands) as no RNA-seq data was available for the whole body. The number of differentially expressed genes comparing female antennae and pheromone glands ranged from 1543 in *P. excessana* to 3334 in *P. octo*, with about half biased in their expression in either antennae or pheromone glands. Biased expression in female antennae was observed within 2307 orthologous groups of which 402 had a gene in each species (Fig. 1a). The annotated genes with the highest expression bias included mainly odorant binding proteins (OBPs), pheromone binding proteins (PBPs) and general odorant binding proteins (GOBPs), which are described in the mined gene section below. Among the top 20 most biased genes common in female antennae we also found an unknown protein (OG2944) with a twelve-fold change in expression, one hypothetical protein (OG11897) similar to *Danaus plexippus* EHJ71609 and a glutamate receptor kainate 2 (OG451). Expression values in female antennae for all genes are reported in Additional file 5: Table S4. In all species, genes displaying female biased expression in antennae were over represented in gene ontology (GO) terms mostly related to sensory perception (Additional file 10: Table S8, Additional file 11: Figure S3). In the Biological Process category, among the most enriched GOs were G protein-coupled receptor signalling pathway (GO:0007187), detection of chemical stimulus involved in sensory perception of smell (GO:0050911), sensory perception of smell (GO:0007608) and ionotropic glutamate receptor signalling pathway (GO: 0035235). In the Molecular Function category, over represented GO terms were odorant binding (GO: 0005549), olfactory receptor activity (GO: 0004984) and ionotropic glutamate receptor activity (GO: 0004970). The most significant over represented GO terms in the Cellular Component included membrane (GO:0016020), intraciliary transport particle B (GO: 0030992) and other GO terms related to membrane and cilium.

We found 2729 orthology groups with biased expression in pheromone glands, of which 278 had a gene in each species (Fig. 1b). Genes with the most biased expression in pheromone glands included a vitellogenin (OG4241), a juvenile hormone binding protein (OG4718) and a takeout protein (OG3767), although none of them was among the most biased genes in all four species (Additional file 5: Table S4). Among the genes with biased expression in pheromone glands we also found three desaturases (OG14247, 13,411 and 10,612) corresponding to the desaturases *desat2*, *desat4* and *desat5* described in [27], two alcohol dehydrogenases (OG11355 and 13,787), three cytochrome P450s and two takeout-like protein 2, a CSP (CSP6a-OG3423) and an odorant binding protein (OBP17). A more detailed description of biased genes with putative function in pheromone biosynthesis, degradation and transport is reported in the mined gene section below. In all species biased expressed genes in pheromone glands showed over represented GO terms mostly related to fatty acid synthase (Additional file 10: Table S8, Additional file 11: Figure S3). In the Biological Process category, among the most over represented GO term in pheromone glands was oxidation-reduction process (GO:0055114) in all four species. Oxidoreductase activity (GO:0016491) was among the most over represented GO terms in Molecular Function, while fatty acid synthase complex (GO:0005835) was in Cellular Component. *Ctenopteustis herana*, and to some extent *P. excessana*, showed enrichment in the Molecular Function for acyl-carrier proteins (GO:0004313, GO:0004314, GO:0004315, GO:0004316, GO:0004317, GO:0004319, GO:0004320, GO:0016295, GO:0016296).

### Manual curation of gene families and evolutionary analyses

**Fig. 2:**
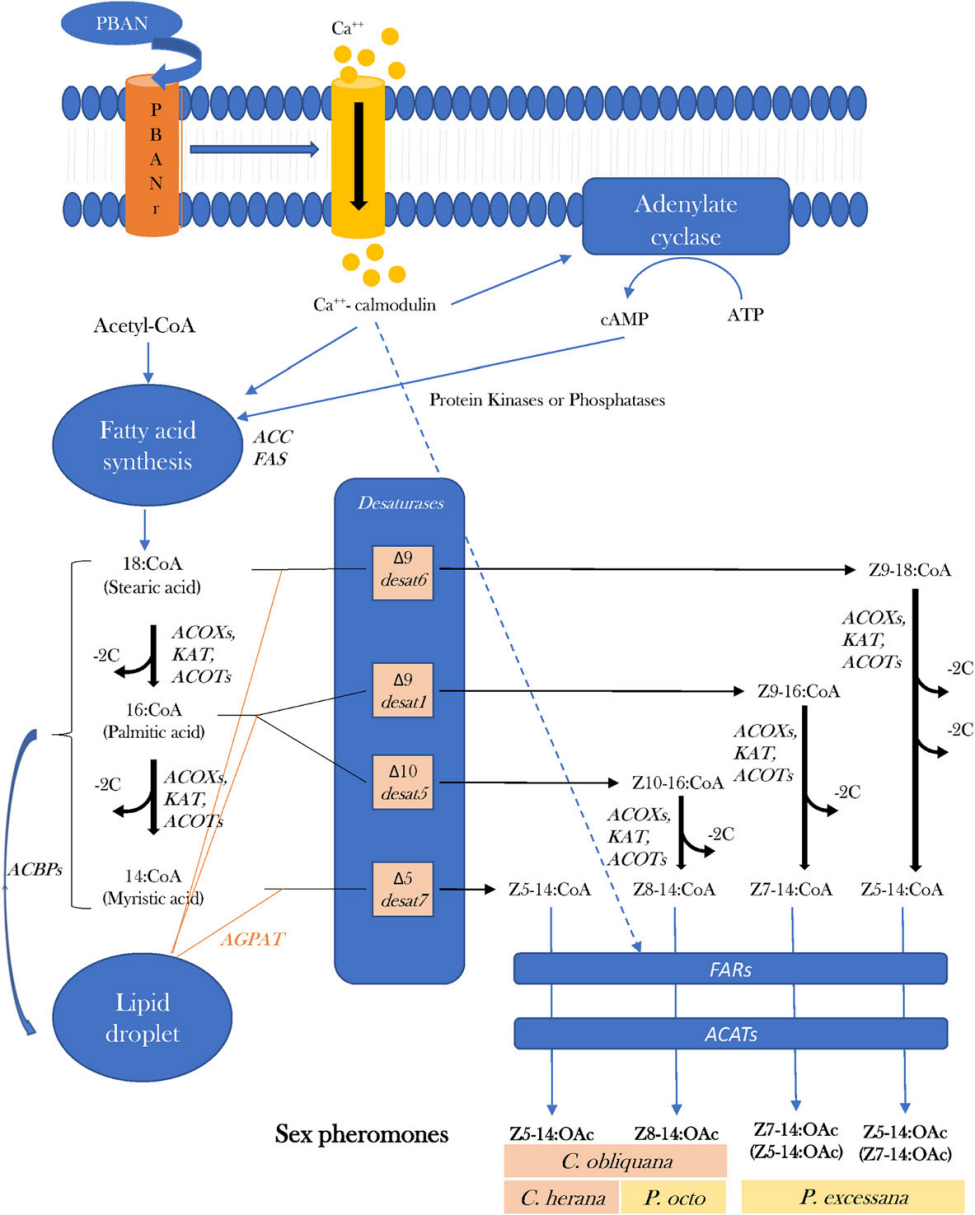
Schematic view of the pheromone biosynthesis pathway in the four New Zealand leafroller moths, *Ctenopseustis herana*, *C. obliquana*, *Planotortrix excessana* and *P. octo* and the putative genes involved

**Fig. 3:**
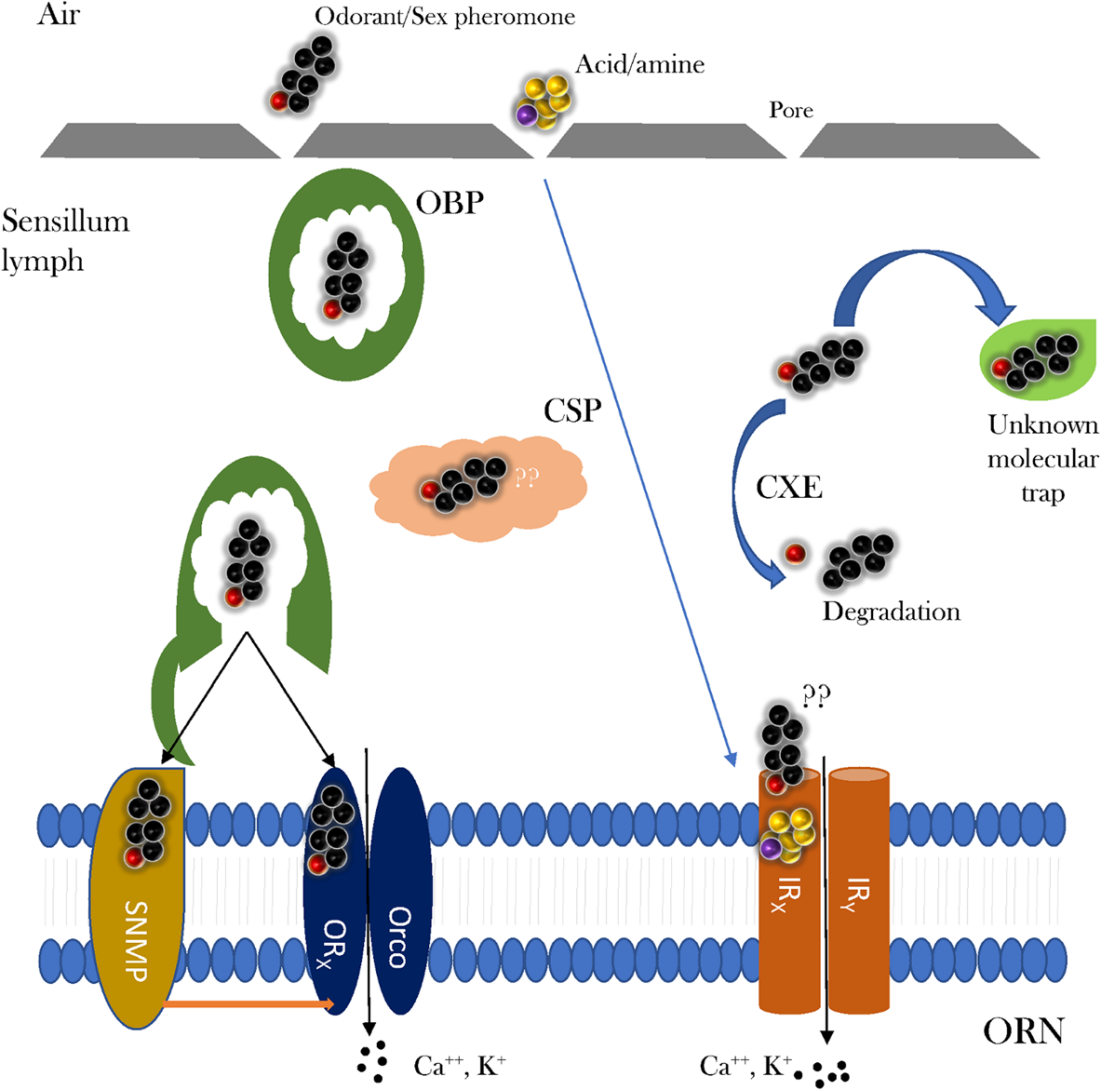
Schematic view of the mechanisms involved in odorant binding, release and inactivation in moth antennae highlighting the gene families mined in this study

We focused on the genes of the pheromone biosynthesis pathway and peripheral olfactory repertoire and proceeded to mine all genes with blast hits for the gene families described in the following paragraphs. Diagrams of the pheromone biosynthesis pathway and pheromone reception system with the putative genes involved are presented in Figs. 2 and 3, respectively. The list of genes and their expression values in the different tissues are reported in Additional file 12: Table S9 and the analyses of positive selection in Additional file 13: Table S10 with a summary in Table 3. The nucleotide sequences of the mined genes are included in the Additional file 14.

**Table 3:** Summary of gene families involved either in pheromone biosynthesis or reception for presence of positive selection and expression bias between antennae in the two sexes and between pheromone gland and antennae in females

		Selection	Expression	
	Gene Famyly	PAML	Sex - Antennae	Female Gland/Antennae
Pheromone pathway	PBAN	–		–
	PBANr	–		> 2
	FAS	–		> 2
	ACC	–		> 2
	ACOX	–		> 2
	Desaturase	–		> 2, > 8
	FAR	–		± > 2, > 8
	ACAT	–		> 2
	ACBP	–		± > 2
	FATP	–		–
	FAAH	–		> 2
	FAT	–		± > 2
	ELOVL	–		± > 2
Pheromone reception	OBP	+	± > 2	
	CSP	–	–	
	SNMP	–	–	
	OR*	+	± > 2	
	IR	–	–	
	CXE	+	–	

+ gene families containing genes under positive selection

># indicate log fold change

± gene families containing genes with bias expression in both tissues

*Analysis from [46]

### Pheromone biosynthesis activating neuropeptide (PBAN) and pheromone biosynthesis activating neuropeptide receptor (PBANr)

Five sequences, one for each species of leafroller moth, had a good match to PBAN*.* The sequences had on average an amino acid identity of 48% with *B. mori* PBAN. A transcript of the PBAN receptor (PBANr) was found in all species except *E. postivittana,* for which we did not have a pheromone gland transcriptome, but we recovered the gene sequence from the genome (unpublished data). In the New Zealand leafroller moths the PBAN receptor was over expressed in the pheromone gland compared to antennae (Additional file 12: Table S9). No evidence for positive selection was detected for either gene using Codeml.

### Fatty acid synthase (FAS) and acetyl-CoA carboxylase (ACC)

**Fig. 4:**
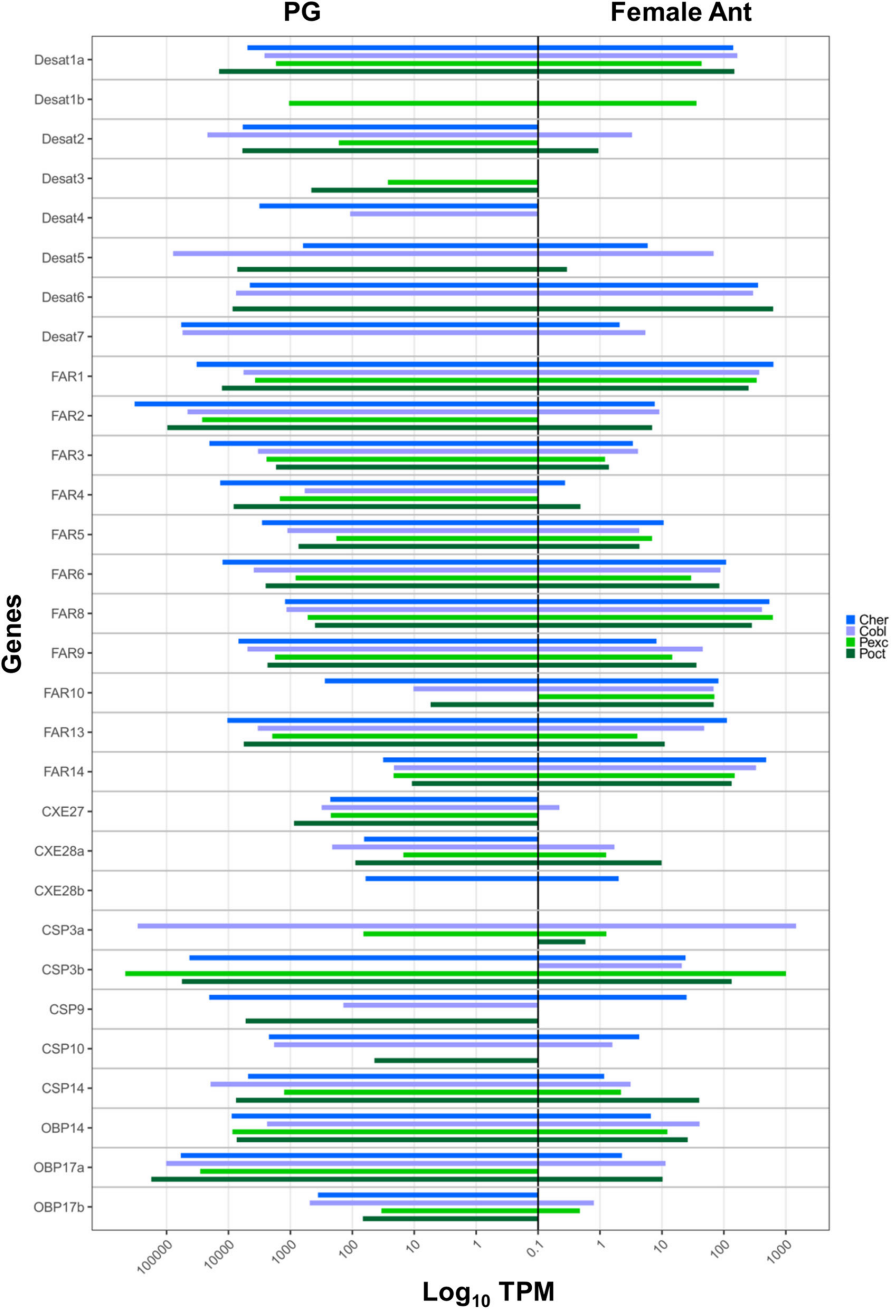
Gene expression in Transcripts Per Kilobase Million (TPM) of desaturares, reductases and genes of the peripheral odorant reception repertoire with biased expression in the pheromone glands compared to antennae in the four New Zealand native leafroller moth species. Only genes showing a log fold change over a threshold of 2 in at least one species are shown

The first step of sex pheromone production is the synthesis of saturated fatty acids by acetyl coenzyme (CoA) carboxylase and fatty acids synthase (FAS). We found only a short fragment of about 900 bp similar to FAS corresponding to the sites between positions 4800 and 5700 of the *D. melanogaster* FAS CDS which totals about 7 kb. This group of orthologous genes showed consistently higher counts (LogFC > 2) in the pheromone gland compared with female antennae across all four endemic New Zealand species (Additional file 12: Table S9). The full CDS of about 7 kb of the acetyl CoA carboxylases (ACC-OG10860) was found in the endemic New Zealand moths, whereas only two fragments (for a total of about 1.1 kb) were found in the antennal transcriptomes of *E. postvittana*, although the full CDS was obtained from the genome (unpublished data). ACC is a multi-domain enzyme that catalyses the ATP-dependent carboxylation of acetyl-CoA to malonyl-CoA, providing the substrate for the biosynthesis of fatty acids catalysing the synthesis of palmitic acid (C16:0) [68]. Malonyl-CoA is also the substrate for distinct elongases in the pathway of very long-chain fatty acyl-CoA synthesis [69]. ACC was more highly expressed in the pheromone gland compared to antennae in all New Zealand species except *P. excessana* in which the Transcripts Per Kilobase Million (TPM) in the pheromone glands was about ten times lower in expression than the other species (Fig. 4). This result is consistent with the previous finding that suggested that in *P. excessana* and *Epiphyas postvittana* synthesis and desaturation of the pheromone occurs outside the pheromone gland [70]. No evidence for positive selection was found for any of these genes.

### Desaturases

**Fig. 5:**
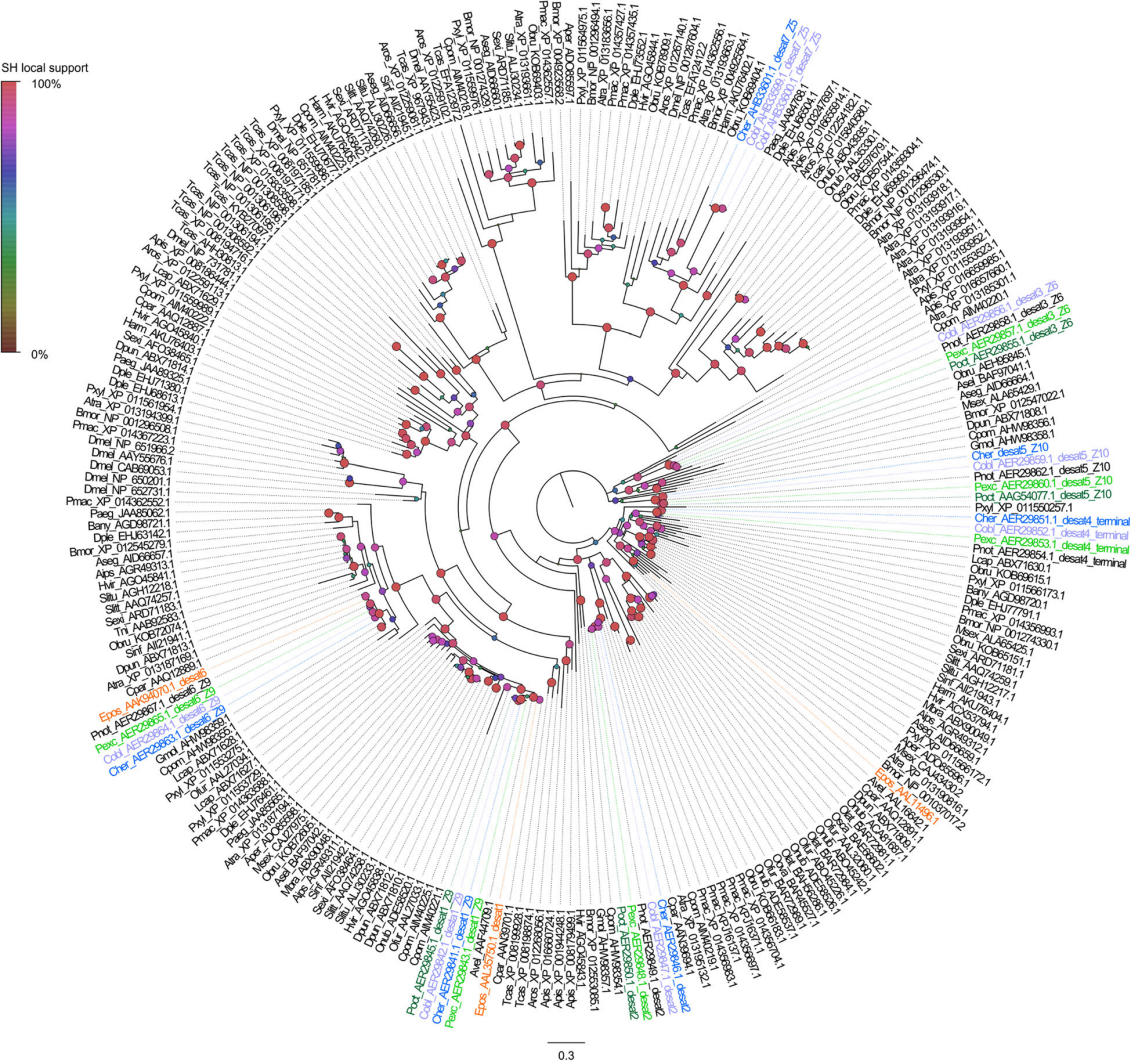
Maximum likelihood tree showing the evolutionary relationships among desaturases retrieved from GenBank from available Lepidoptera, including the New Zealand leafroller moths *Ctenopseustis herana* (Cher, highlighted in blue), *C. obliquana* (Cobl, highlighted in light purple), *Planotortrix excessana* (Pexc, highlighted in light green) and *P. octo* (Poct, highlighted in dark green) and the horticultural pest *Epiphyas postvittana* (Epos, highlighted in orange) and *Drosophila melanogaster* (Dmel), *Tribolium castaneum* (Tcas) and *Acyrthosiphon pisum* (Apis). Circles at nodes indicate Shimodaira-Hasegawa local support, with colours and size of circles being proportional to the percentage of support (0–100%)

Fatty-acyl desaturases introduce double bonds at specific carbon positions along the backbone of pheromone precursors and therefore are considered a major player in the diversity of sex pheromones. The analysis of the transcriptomic and genomic data did not reveal new genes other than the seven desaturases already described by [27, 44, 71], therefore we redirect the reader to those papers for the results. However, while these previous publications found that *desat5* was expressed in *C. herana*, they did not present its sequence, which is now reported here. The analysis of both transcriptomic and genomic data of *Planotortrix* species failed to find an orthologue of *desat7* in this genus. *Desat7* has been described as a divergent desaturase by [71], clustering away from other desaturases. In Fig. 5 we show the phylogenetic relationships of the seven *Ctenopseustis* and *Planotortrix* desaturates with *desat7* clustering with several desaturases of other moths (e.g. *Plutella xylostella* and *Amyelos transitiella*) and aphids (e.g *Acyrthosiphon pisum*) in a group that is sister to Δ11 desatusares of non-lepidopteran insects. The expression of these desaturases in pheromone glands and female antennae are presented in Fig. 4.

### Acyl-CoA oxidases (ACOX), 3-oxoacyl-CoA thiolase (KAT) and Acyl-CoA thioesterases/hydrolases (ACOTs)

Modification of pheromone precursors involve chain shortening by β oxidation enzymes. This step has not been well characterized in insects but most likely is similar to vertebrates. β-oxidases act in concert with a 3-oxoacyl-CoA thiolase to chain shorten acyl-CoAs by removing an acetyl group [18]. We found 35 sequences with similarity to acyl-CoA oxidases (COAX1 and 3) from which 11 were excluded after further inspection because they were too short or, after correction, were identical to other transcripts. The phylogenetic relationships of the remaining 24 sequences are shown in Additional file 15: Figure S4. The tree shows two main clusters which correspond to *B. mori* ACOX1 and 3. Three clusters of ACOX1 (a, b and c) were found in the leafroller moths compared with four found in *B. mori.* Two copies of ACOX3 (a and b) were found both in the leafroller moths and *B. mori*, however in *E. postvittana* we failed to find a transcript for ACOX3a (OG11206), but recovered the full CDS from the genome. An orthologous group (KAT), with one full CDS sequence per species, had 78% similarity to *B. mori* 3-oxoacyl-CoA thiolase (XP_004930405). No evidence of positive selection was observed for any of the oxidase and thiolase genes. Although the four species of New Zealand tortricids investigated display different routes of pheromone biosynthesis (Fig. 2), ACOX3a was the only β-oxidase with biased expression in the pheromone glands and no differences were observed among species (Additional file 12: Table S9). ACOTs (or acyl-CoA hydrolases) are a group of enzymes that catalyze the hydrolysis of short to long-chain acyl-CoAs and promote β-oxidation by regulating the levels of free coenzyme A (CoASH) for the thiolase reaction [72]. CoASH is also required for the final step of β-oxidation, the 3-ketoacyl-CoA thiolase reaction (see below). Two thioesterase groups were found in leafroller moths, one more similar to palmitoyl-protein thioesterase (ACOT2) and the other to acyl-protein thioesterase (ACOT1). In *H. virescens* palmitoyl-protein thioesterase is over-expressed in pheromone glands [26], however in all leafroller moths both thioesterases were equally expressed in antennae and pheromone glands (Additional file 12: Table S9). No evidence of positive selection has been observed for either of these genes.

### Fatty acid reductases (FAR)

**Fig. 6:**
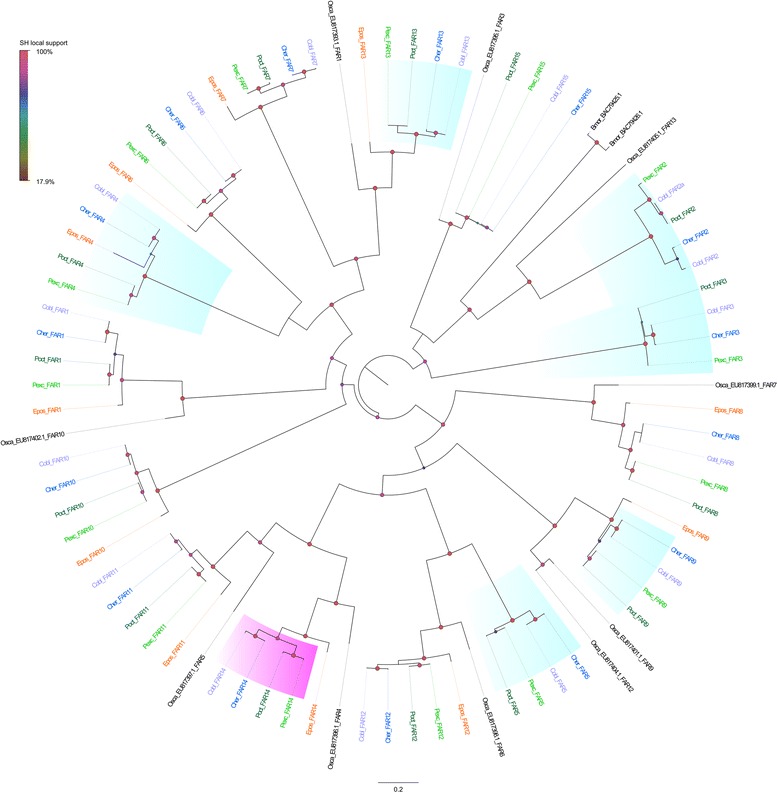
Maximum likelihood tree showing the evolutionary relationships among fatty acid reductase (FAR) proteins mined from the transcriptomes of the New Zealand leafroller moths *Ctenopseustis herana* (Cher, highlighted in blue), *C. obliquana* (Cobl, highlighted in light purple), *Planotortrix excessana* (Pexc, highlighted in light green) and *P. octo* (Poct, highlighted in dark green) and the horticultural pest *Epiphyas postvittana* (Epos, highlighted in orange) and including *Bombyx mori* (Bmor) and *Ostrinia scapulalis* (Osca). Circle at the nodes represent the Shimodaira-Hasegawa local support. Colours and size of circles are proportional to the percentage of support (0–100%). The clusters shaded in aquamarine highlight biased expression in pheromone glands relative to antennae and clusters shaded in fuchsia indicate biased expression in female antennae relative to pheromone glands

Once the specific double bond of the pheromone intermediate is produced, in tortricids the carbonyl carbon is typically modified to an acetate ester. This first requires the reduction of a fatty-acyl precursor to an alcohol by a fatty acid reductase [18]. One hundred and eight sequences had a blast hit with FARs. After manual curation 30 sequences were excluded from further analyses because they were too short or after correction, were identical to other transcripts. The phylogenetic relationship among the FARs is given in Fig. 6. The tree shows 15 clusters of orthologous sequences. For four clusters we failed to find an orthologue in *E. postvittana*. Three orthologous groups FAR2, 3 and 4 showed the greatest expression in the pheromone glands compared to the antennae in all four New Zealand endemic moth species, with log fold changes ranging from nine to 33 (Fig. 4, Additional file 12: Table S9). FAR2 was closely related to *Ostrinia scapulalis* OscaFAR XIII and *B. mori* pgFAR, which are pheromone gland specific in both species [30, 31] and the main determinant of the phenotypic variation in female pheromone between the *Z* and *E* races of *O. scapulalis* [33]. FAR3 is closely related to OscaFAR II, whereas FAR4 had no similarity with *O. scapulalis* and *B. mori* FARs. Distinct from FAR XIII and pgFAR, none of these three FARs were specific to pheromone glands, although the number of counts in the female antennae was extremely low. Two FARs, FAR10 and FAR14, showed biased expression in antennae compared to pheromone glands (Fig. 4). No evidence of positive selection was detected for any of the FAR genes.

### Acetyl-CoA acetyltransferases / 3-ketoacyl-CoA thiolases (ACAT)

Acetate ester pheromones are produced from the fatty alcohol intermediate by a acetyl-CoA:fatty alcohol acetyltransferase [18]. This enzyme is one of the last steps in the pheromone biosynthetic pathway of many moths, however, to date no pheromone alcohol acetyltransferases has been isolated. Four orthologous groups, with a sequence from each of the five species, contained a conserved domain for acetyl-CoA acetyltransferase (Additional file 16: Figure S5). Except for one group (ACAT4), only partial CDS were recovered from the antennal transcriptomes of *E. postvittana* and the full CDS became available only after mining the genome. Also, only a partial transcript was recovered for both *Ctenopseustis* species in the orthologous group ACAT2. All these sequences also contained a thiolase conserved domain and showed similarity to the 3-ketoacyl-CoA thiolase, the enzyme that performs the last step of the β-oxidation cycle. Two genes (ACAT1 and ACAT2) showed pheromone gland biased expression (Additional file 12: Table S9). No evidence of positive selection was observed for any of the four groups.

### Acyl-CoA-binding proteins (ACBP)

ACBPs bind straight-chain (C14-C22) acyl-CoA esters protecting them from hydrolysis [73]. They serve as carriers or cellular deposits for the acyl-CoAs used in pheromone biosynthesis [74]. We identified three orthologous groups with similarity to ACBPs. One group (ACBP1) clustered with the midgut ACBP (mgACBP) of *B. mori*, whereas the pheromone gland ACBP (pgACBP) of *B. mori* clustered with a transcript found only in *P. excessana* (Additional file 17: Figure S6). The sequences forming the two remaining clusters, although showing similarity with ACBPs, were more than 200 amino acids long compared with the approximately 90 amino acids of mgACBPs and pgACBPs. Biased expression was found for ACPB1 but it was not consistent across species. *C. obliquana* showed over expression in antennae, while in the two *Planotortrix* species the gene was over expressed in the pheromone glands (Additional file 12: Table S9). No evidence of positive selection was observed.

### Fatty acid transporter proteins (FATP)

Fatty acid transport proteins (FATPs) belong to an evolutionary conserved family of membrane-bound proteins that facilitate the uptake of extracellular long chain fatty acids into the cell and catalyze the ATP-dependent esterification of very long-chain fatty acids to the corresponding acyl-CoA derivatives. Twenty-one sequences had a blast hit with very long chain fatty acid transport proteins. The sequences (except one which was excluded from the analysis because it was too short) were clustered into four orthologous groups and their phylogenetic relationships are shown in Additional file 18: Figure S7. The *B. mori* FATP (NP_00127727), homologous to FATP3, is predominantly expressed in pheromone glands and is up-regulated 1 day prior to eclosion [75]. Although, our moths were collected as adults, FATP3, as well as FATP4, showed biased expression in the pheromone glands of all species except *P. excessana* (FATP3) and *P. octo* (FATP4) (Additional file 12: Table S9). No evidence of positive selection was observed for any of the FATP genes with any of the models tested, however for the FATP3 we found a positively selected site at position 640.

### Other proteins involved in fatty acid biosynthesis

Before adult moth emergence, pheromone gland cells produce and accumulate pheromone precursor in the form of triacylglycerols (TAG). In eukaryotes TAGs are synthetized through two major pathways, the glycerol-3-phosphate (G3P) and the monoacylglycerol pathways, although in most cells TAG is synthetized mainly by G3P. Triacylglycerol synthesis occurs in the order; glycerol-3-phosphate acyltransferase (GPAT; EC 2.3.1.15), 1-acyl-sn-glycerol-3-phosphate acyltransferase.

(AGPAT; EC 2.3.1.51) and phosphatidate phosphohydrolase (PAP; EC 3.1.3.4) to produce sn-1,2-DAG, a precursor of TAG, phosphatidylcholine and phosphatidylethanolamine [76]. In animals, members of AGPAT have been shown to transfer the unsaturated fatty acyl groups, glycerol-3-phosphate [77]. We found four distinct orthologous groups similar to AGPAT1, 2, 3 and 6 of *H. virescens.* A sequence similar to AGPAT5 was found only in *P. octo* (Additional file 19: Figure S8). None showed biased expression in the pheromone glands, but one group, AGPAT1, showed biased expression in the antennae compared with pheromone glands (Additional file 12: Table S9). No evidence for positive selection was observed.

Sixty-three sequences from the five species of leafroller moths had a blast hit with elongation of very long chain fatty acids proteins (ELOVL) and were clustered in 14 orthologous groups by ProteinOrtho. After manual curation, we discarded 8 sequences because they were too short or after correction they resulted in transcripts that were identical to others. The phylogenetic analysis revealed 10 clusters, each containing one representative of each species, shown in Additional file 20: Figure S9. No evidence of positive selection was observed for any of these clusters. Two orthologous groups (ELOVL2 and ELOVL1) consistently displayed biased expression in pheromone glands across species (Additional file 12: Table S9).

A total of 106 sequences had similarity with fatty acid transferases (FAT), however 12 of these were excluded from further analysis because they were either too short or contained many missing nucleotides. The remaining 94 sequences clustered into 23 orthologous groups and their phylogenetic relationships are shown in Additional file 21: Figure S10. Of these groups, 12 were represented in all 5 species. One group (FAT7) showed antennal biased expression relative to pheromone glands in all New Zealand species, whereas FAT19, was antennal biased except in *P. excessana*. FAT17 instead was consistently over-expressed in the pheromone glands across all species (Additional file 12: Table S9). No evidence of positive selection was observed.

A total of 31 sequences had similarity with fatty acid hydrolases (FAAH), four of which were excluded from further analysis because they were only partial. FAAHs belong to the serine hydrolase superfamily and are secondary targets of organophosphates. FAAHs can act as scavenging or detoxifying enzymes and they are principal targets for pest management [78]. FAAH sequences clustered in 5 orthologous groups as shown in the phylogenetic tree in Additional file 22: Figure S11. Sequences belonging to the orthologous groups FAAH1 and FAAH2 were consistently biased in their expression in the pheromone glands of all four New Zealand species (Additional file 12: Table S9). No evidence of positive selection was found.

### Odorant receptors

**Fig. 7:**
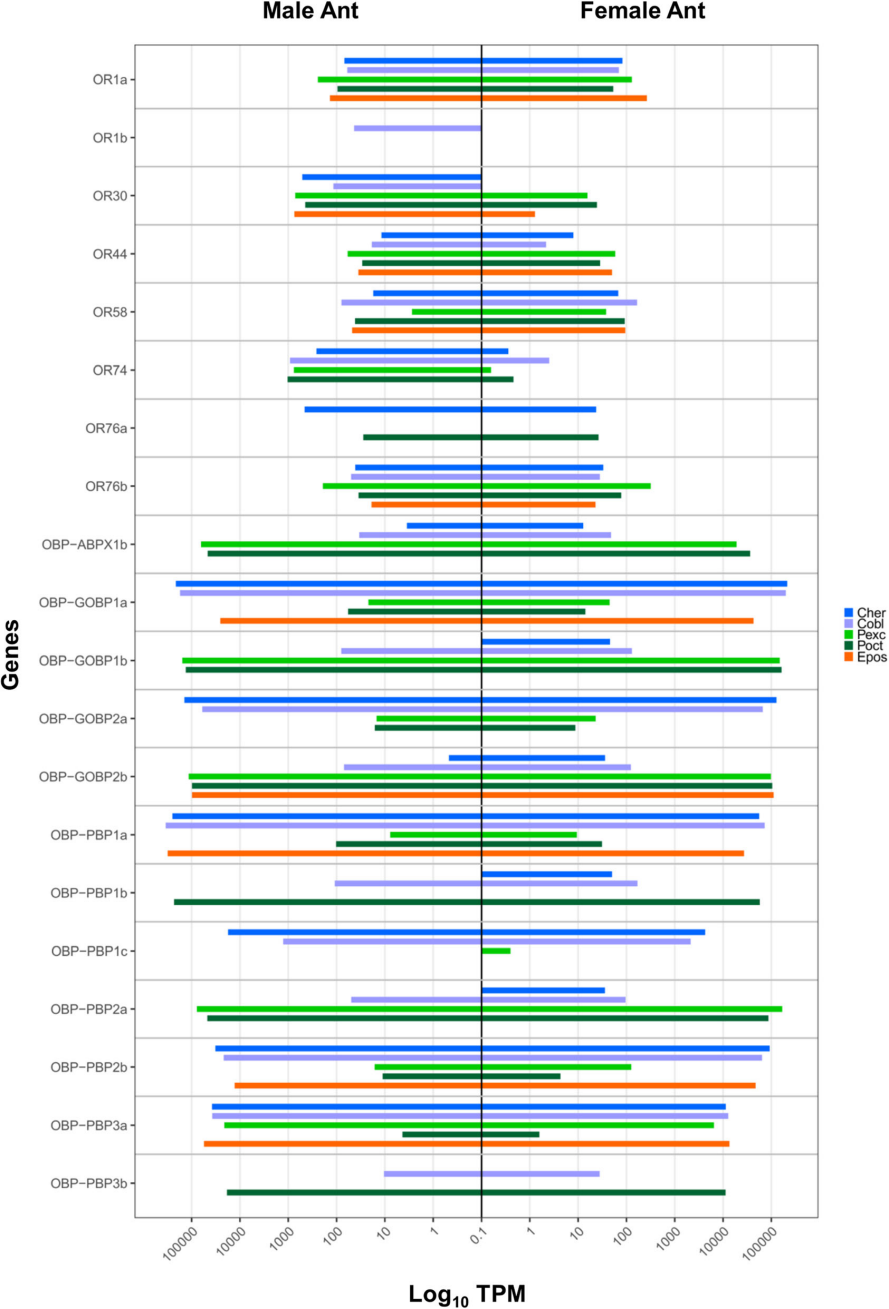
Expression levels in Transcripts Per Kilobase Million (TPM) in male and female antennae of the genes putatively involved of the peripheral olfactory repertoire in the four New Zealand native leafroller moth species, *Ctenopseustis herana*, *C. obliquana*, *Planotortrix excessana*, and *P. octo*. Only genes with a log fold change over a threshold of 2 in at least one species are shown

Odorant receptors (ORs) of the *Ctenopseustis* and *Planotortrix* species and of *E. postvittana* have been mined previously [45, 46, 49] and no new genes have been discovered in this analysis. The biased expression of ORs between male and female antennae was consistent with previous results and only two ORs (OR74 and OR30) were biased in male antennae of all New Zealand endemic moth species (Additional file 12: Table S9, Fig. 7). We are not reporting results on these genes further and direct the reader to the original publications for results. However, OR expression will be discussed in light of all other genes mined in this paper.

### Chemosensory proteins and odorant binding proteins

Chemosensory proteins (CSPs) and Odorant binding proteins (OBPs) are small (10 to 30 kDa), globular, water-soluble proteins characterized by four to six highly conserved cysteine residues and the formation of two to three disulphide bonds, respectively [79]. Members of these two classes of protein function as carriers facilitating the transfer of odour molecules to the receptors on the dendrites of olfactory neurons [80–82]. These families are thought to be related, with a common ancestor dating back to after the terrestrialization of the Arthropoda about 380–450 Ma [83]. In contrast to OBPs, which are very diverse and classified into several subfamilies, CSPs are more conserved across insects [84].

A total of 65 sequences in the five leafroller moth species showed similarity with chemosensory proteins and another 23 with ejaculatory-bulb specific proteins. These sequences were clustered into 14 orthologous groups by ProteinOrtho and they were all characterized by the presence of four conserved cysteines typical for chemosensory proteins [85]. Sequences of CSP11 were considerably longer than other CSPs with about 300 residues instead of the typical ~ 120 amino acids. After manual correction, 79 sequences remained for which phylogenetic relationships are shown in Additional file 23: Figure S12. CSP4 was most closely related to CSP19 of *Sesamia inferens* when CSPs from other insects were included in the phylogenetic tree. CSP19 from *S. inferens* is over-expressed in male antennae and it is thought to play a role in sex pheromone reception [86]. In our moths, none of CSPs showed biased expression in male antennae, whereas CSP1 (OG12340) showed biased expression in female antennae compared to pheromone glands in all New Zealand endemic species (Additional file 12: Table S9, Fig. 4). CSP1 clustered with CSP1, 2 and 3 from the alfalfa plant bug *Adelphocoris lineolatus* in which these three CSPs are thought to mediate host recognition [87]. In general, CSPs were biased in their expression to pheromone glands (Additional file 12: Table S9, Fig. 4). We found no evidence for positive selection in any CSPs.

**Fig. 8:**
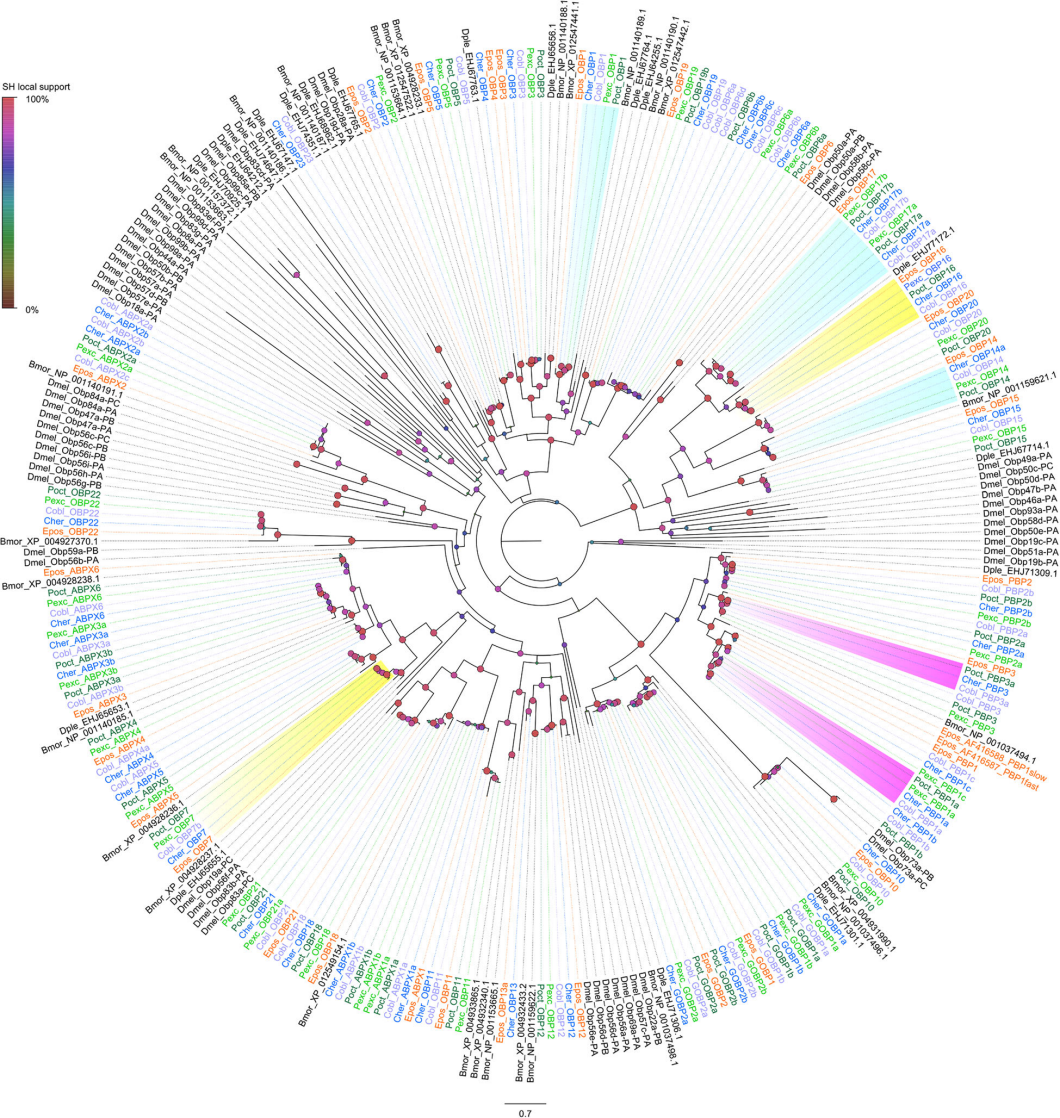
Maximum likelihood tree showing the evolutionary relationships among odorant binding proteins (OBPs). The tree includes the genes isolated from the New Zealand leafroller moths *Ctenopseustis herana* (Cher, highlighted in blue), *C. obliquana* (Cobl, highlighted in light purple), *Planotortrix excessana* (Pexc, highlighted in light green) and *P. octo* (Poct, highlighted in dark green) and the horticultural pest *Epiphyas postvittana* (Epos, highlighted in orange). Circles at the nodes represent the Shimodaira-Hasegawa local support, with colours and size of circles being proportional to the percentage of support (0–100%). Groups shades in green were antennae biased in male compared to females, whereas orange shaded groups were pheromone gland biased. Groups in blue indicate genes under positive selection. Bmor = *Bombyx mori*, Dmel = *Drosophila melanogaster*, Dple = *Danaus plexippus*

We found 209 sequences with similarity to odorant binding protein gene members, including the specific subclass of antennal binding proteins X (ABPX), general odorant binding proteins (GOBP), odorant binding proteins (OBP) and pheromone binding proteins (PBP). Of the 209 contigs, 157 were grouped into 38 orthologous groups, of which 10 were ABPXs, three GOBPs, 13 OBPs and five PBPs. Following the manual curation of contig alignments, we were left with 193 sequences for a total of 44 genes for which phylogenetic relationships are shown in Fig. 8. GOBPs and PBPs are thought to have evolved by a duplication event. They form a monophyletic clade within the OBP gene tree and are typically located in a physical gene cluster [88] (see also Fig. 8). In fact, PBPs and GOBPs form a monophyletic clade with the inclusion of OBP10. The other two subfamilies of odorant binding protein members, ABPXs and OBPs were intermingled and formed four main clusters. One of these contained all odorant binding proteins with extra cysteines and a conserved proline (plus-C group in Fig. 8). Odorant binding proteins that were missing two conserved cysteines were also clustered together (minus-C group in Fig. 8). Two GOBPs and three PBPs were described in *E. postvittana* [49, 89]. In the New Zealand tortricids, we found that both GOBP1 and 2 were duplicated and that a similar duplication event seems to have occurred for each of the PBPs. We identified three genes closely related to EposPBP1 (here called them PBP1a, b and c), which differ by more than 70 nucleotide substitutions within a species. These duplications seem to predate the diversification of the two genera, indeed in all the PBP and GOBP orthologous groups the *C. herana* gene is sister to *P. excessana* and the *C. obliquana* representative sister to *P. octo* in the phylogenies. In most of the clusters the expected relationships of genes from co-generic species were observed, apart from ABPX3a for which the *C. herana* representative was the sister of *P. excessana* and *C. obliquana* of *P. octo*.

While GOBPs are generally expressed at similar levels in both male and female antennae, PBPs show strong sexual dimorphism ([90] and references therein). PBP1a and PBP3a were biased in males of the *Ctenopseustis* species, whereas PBP3b was biased in males of *P. excessana*. PBP1b and PBP2a were biased in female antennae in *C. herana*, while PBP1b was male biased in *P. octo* (Additional file 12: Table S9, Fig. 8). PBP1 and 3 are both capable of binding pheromone components [89, 91]. The level of expression of the different PBP members was quite different among species (Additional file 12: Table S9, Fig. 7). PBP1a showed the highest expression in *Ctenopseustis* species whereas, PBP1c was highest in *P. octo*. In *P. excessana* all three PBP1s were hardly detectable at the transcript level. PBP2a was more highly expressed than PBP2b in both *Ctenopseustis* species and the opposite was true for the *Planotortrix* species. A similar expression pattern was observed between the GOBPs and PBP3s, with GOBP1a, GOBP2a and PBP3a highly expressed in *Ctenopseustis*, whereas GOBP1b, GOBP2b and PBP3b were in *Planotortrix*. Similarly, another couple of OBPs, ABPX1a and b showed an alternative expression pattern with ABPX1a more highly expressed in *Ctenopseustis* and ABPX1b in *Planotortrix* (Additional file 12: Table S9, Fig. 7).

In the expression comparison between female antennae and pheromone glands fourteen groups showed biased expression in the antennae of all species, whereas two plus-C OBPs, OBP14 and OBP17 were pheromone gland biased in their expression (Additional file 12: Table S9, Fig. 4).

Some evidence of positive selection was observed in at least two of three model comparisons for two genes, OBP7 and OBP16, with one significantly selected site in each gene (Additional file 13: Table S10). A significant site was also found in each of ABPX3a and OBP18. PBP1 isolated from several species within *Ctenopseustis* and *Planotortrix* revealed rapid rates of sequence evolution along the lineage leading to PBPs used by *C. filicis* and *C. fraternal*, species that utilize 16 carbon acetates instead of the 14 carbon acetates usually found among member of the two genera [92, 93]. The tortricids investigated in this study have pheromones composed of 14 carbon acetates and no evidence of positive selection was found for either PBP1 or any other of the PBPs.

### Sensory neuron membrane proteins (SNMPs)

SNMPs are membrane proteins related to the human fatty acid transporter CD36 and are associated with chemosensory neurons in insects. Two classes of SNMPs have been described in insects of which SNMP1 has been shown to be essential for detection of the pheromone *cis*-vaccenyl acetate in *Drosophila* [94, 95]. Four orthologous groups, comprising 16 sequences, showed similarity with SNMPs, however OG17722 after visual inspection and manual correction was the same sequence as SNMP2. Of the three remaining groups one showed similarity to SNMP1 with orthologues in all five species and one to *Drosophila* SNMP2. This latter group had no orthologues in *E. postvittana*. The last group SNMP3 was sister to SNMP2 and was found in all five leafroller moths but did not have an orthologue in *D. melanogaster* (Additional file 24: Figure S13). SNMP1 has been shown to be antennal specific in several lepidopteran species [96, 97]. In the New Zealand tortricids SNMP1 was barely detected in the pheromone glands, and showed higher counts in male antennae compared with females (Additional file 12: Table S9), albeit no significant biased expression was observed. The third group of SNMPs was expressed also in the pheromone glands in contrast to the findings of [98] who did not find SNMPs expressed in pheromone glands (Additional file 12: Table S9). No evidence of positive selection was found for any of these genes.

### Ionotropic receptors (IRs)

The Ionotropic Receptors (IRs) are chemosensory receptors found across all protostomes, forming a highly divergent subfamily of the Ionotropic Glutamate Receptors [99, 100]. IRs detect volatile acids and ammonia and most are expressed in sensory neurons present in coeloconic sensilla, which do not express odorant receptors [99]. In contrast to ORs, many IRs are conserved in most insects suggesting they comprised the original family of olfactory receptors [100, 101]. A total of 152 sequences had a blast hit with insect IRs and ionotropic glutamate receptors (iGluR). ProteinOrtho clustered 126 of these sequences into 28 orthologues groups. After sequence alignment and manual curation, 120 sequences remained for which the phylogenetic relationships are shown in the Additional file 25: Figure S14. In the phylogenetic analysis we also included *Drosophila melanogaster* iGluRs and IRs as outgroups. We identify 22 IR genes in the New Zealand tortricid moths compared to the 18 identified in *E. postvittana* [49] and 63 (including 9 putative pseudogenes) found in *D. melanogaster* [99, 100]. In *Drosophila* 15 IRs are expressed only in antennae and are referred to as antennal IRs [99]. At least 16 clusters of leafroller moth IRs group with the “antennal” IRs of *D. melanogaster*, seven of which were expressed exclusively, or almost exclusively, in the antennae (S Additional file 12: Table S9). Two groups clustered with “divergent” drosophila IRs. These IRs included orthologues of the presumably “ancestral” IR25a of *Drosophila*, which is expressed in the chemosensory neurons of insects, nematodes and molluscs [100]. This gene displays biased expression in the antennae of the New Zealand tortricids compared to pheromone glands (Additional file 12: Table S9) and was most closely related to IR8a, which is thought to be evolved by duplication from IR25a [100]. IR8 also shows biased expression in antennae. Both the IR25a and IR8a genes retain sequence similarity to AMPA iGluRs as shown in the tree (Additional file 25: Figure S14), and are expressed in many coeloconic sensilla where they are thought to act as coreceptors for the more divergent IRs [102]. Four IRs in *D. melanogaster* are not found in coeloconic sensilla, IR21a, IR40a, IR64a and IR93a, with IR21a expressed in aristal neurons and the other three in sacculus neurons [103]. Arista contain thermosensory and not olfactory neurons, while the sacculus house both olfactory and thermosensory neurons [104]. Neurons expressing IR64a are olfactory, whereas the function of IR40a and IR93a expressing neurons are still unknown [103]. These IRs, except for IR40a, have homologues in the five tortricid species. In contrast to [98] who did not find IRs in the pheromone glands of *A. ipsilon*, two IRs (IR76 and IR25) were also expressed in the pheromone glands in the New Zealand tortricids (Additional file 12: Table S9). Four orthologous groups clustered with iGluR (Additional file 25: Figure S14), two of which, IR-iGluR3 and IR-iGluR4, were both biased in their expression in pheromone glands (Additional file 8: Table S8). None of IRs showed evidence of positive selection, although a positive site at alignment position 646, was found in IR-iGluR4 (Additional file 13: Table S10).

### Carboxylesterases

**Fig. 9:**
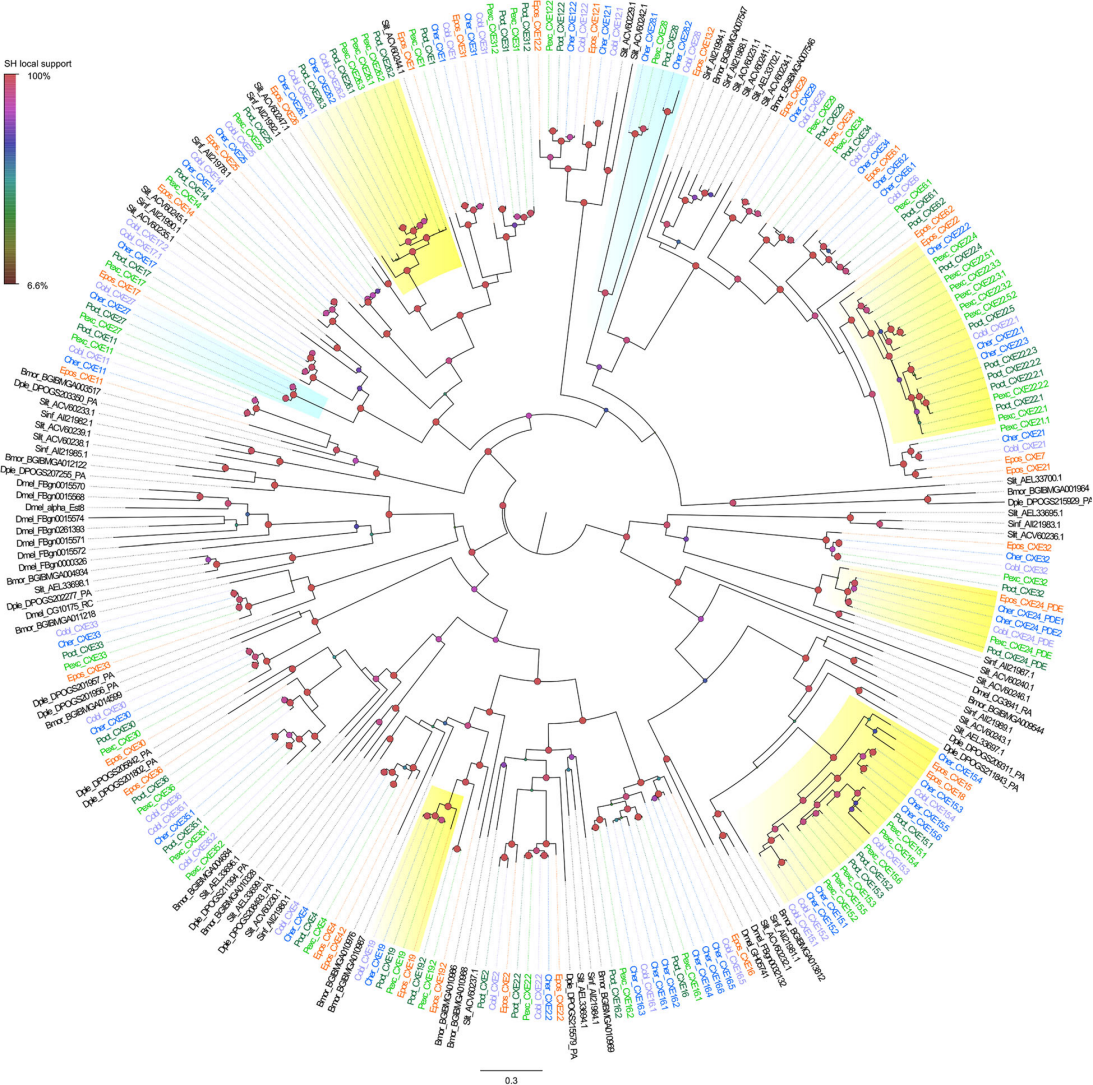
Maximum likelihood tree showing the evolutionary relationships among carboxylesterases (CXEs). The tree includes the curated genes from the New Zealand leafroller moths *Ctenopseustis herana* (Cher, highlighted in blue), *C. obliquana* (Cobl, highlighted in light purple), *Planotortrix excessana* (Pexc, highlighted in light green) and *P. octo* (Poct, highlighted in dark green) and the horticultural pest *Epiphyas postvittana* (Epos, highlighted in orange). Circle at the nodes indicate the Shimodaira-Hasegawa local support, with colour and size of circle being proportional to the percentage of support (0–100%). Groups shades in orange were pheromone gland biased. Groups in blue indicate positive selection. Bmor = *Bombyx mori*, Dmel = *Drosophila melanogaster*, Dple = *Danaus plexippus*, Sinf = *Sesamia inferens*, and Slit = *Spodoptera littoralis*

The pheromone signal needs to be degraded so the next signal molecule can be detected by the olfactory sensory neurons (OSNs). Several classes of enzymes are thought to be involved in the degradation of pheromones including cytochrome P450s, GSTs and CXEs, but clear evidence has been found only for CXEs [105]. A total of 322 sequences showed a blast hit (e-values < 2.46e^− 11^) with carboxylesterases and 20 with juvenile hormone esterases (JHE). These sequences were grouped into 82 orthologous groups by ProteinOrtho. After inspection of the aligned sequences, manual correction and elimination of the shortest sequences, 210 sequences remained whose phylogenetic relationships are shown in Fig. 9. The carboxylesterase CXE12 of *E. postvittana* identified by [49] had the best similarity matches to JHEs and had orthologues only in the two *Ctenopseustis* species. The gene formed a monophyletic group with another JHE (CXE12b) which had an orthologue in all five species. *Epiphyas postvittana* EposCXE24 (OG513) has been suggested to be the putative carboxylesterase with pheromone degradation ability as it clusters with other pheromone degrading enzymes from other moths [49, 106]. This putative pheromone degrading enzyme forms a monophyletic group with five sequences (CXE24), one from each of the New Zealand endemic leafroller species, except for *C. herana* in which this gene is duplicated. Pheromone degrading enzyme sequences had a best match with the FE4 esterase of the peach potato aphid, *Myzus persicae*, which is involved in insecticide resistance [107, 108]. This gene also showed some evidence for positive selection in two out of the three tests and one site under positive selection (Additional file 13: Table S10), but it did not show biased expression (Additional file 12: Table S9) and most likely is ubiquitously expressed as is its orthologue CXE13 of *S. littoralis* [106]. In three groups of carboxylesterases, CXE6 CXE15 and CXE16, we found multiple paralogues in each of the New Zealand moth species that could indicate a recent expansion. These carboxylesterases have the best matches with the antennal carboxylesterases CXE14, 5 and 10 of *Danaus plexippus*, *S. exigua* and *S. litura*, respectively. CXE15 and two other clusters CXE13 and CXE26 also showed evidence of positive selection in all three model comparisons (Additional file 13: Table S10), however in the case of CXE15 this result should be taken with caution because of the presence of multiple paralogues. Only two carboxylesterases showed biased expression across all four New Zealand species. CXE2b showed biased expression in the pheromone glands whereas CXE27 was biased in the female antennae (Additional file 12: Table S9, Fig. 4). For CXE27 we failed to find an orthologue in *E. postvittana*. CXE27 clustered with SlitCXE8 which shows biased expression in the antennae in *S. littoralis* [106]. CXE2, CXE4 and CXE14, although expressed in all three tissues, showed biased expression in female antennae compared to pheromone glands, but there was no difference between male and female antennae (Additional file 12: Table S9). CXE2 and CXE4 lack a signal peptide and cluster with SlitCXE10 and SlitCXE3, both of which belong to the α-esterase clade, a group of intracellular esterases that are known for their involvement in the detoxification of insecticides and xenobiotics [109]. CXE14 however contains a signal peptide suggesting it may be secreted into the sensillum lymph [49].

## Discussion

We seek to understand the mode of molecular evolution underpinning sex pheromone evolution in New Zealand’s endemic sibling genera *Ctenopseustis* and *Planotortrix.* Given that changes in pheromone biosynthesis to produce the different pheromones seem to involve changes in gene expression of desaturase genes [27, 44], we sought to examine whether gene expression evolution is more generally involved in the evolution of pheromone biosynthesis in females and pheromone reception in males. To do this we undertook an analysis of RNAseq data taking both a total transcriptome and candidate gene approach to investigate expression and sequence differences that might underpin the evolution of these mate recognition systems. We identified and manually curated more than 70 new candidate genes in the pheromone biosynthesis and peripheral reception pathways of four closely related species of leafroller moths, *Ctenopseustis herana* and *C. obliquana* and *Planotortrix excessana* and *P. octo*. These four species are mostly sympatric and polyphagous, but differ in their pheromone blend (reviewed in [38]). Each species pair are likely to be sibling but this is not completely certain. A fifth tortricid species, *Epiphyas postvittana* was also included in part of the analyses, however only the antennal transcriptome of both sexes is currently available. The transcriptomes used in this study were the same as used by [45, 46, 49], however here we carried out de novo assemblies with several algorithms of all tissues combined for each species. These assemblies were merged within species with Evidentialgene, so the resulting number of final transcripts described here are not directly comparable with those obtained in the previous studies. The newly identified genes from these species such as PBAN and the PBAN receptor, synthases, ACC, β-oxidation enzymes, FARs, FATs, FATPs, OBPs, CSPs, SNMPs and CXEs have been added to the list of ORs and desaturases already published for these species. Since we did not have biological replicates for each species for the comparison of the expression of these candidate genes we used the closely related species as “biological replicates”. We will discuss the commonalities and differences in their expression among species using the RNAseq data. Previous comparisons of differential expression of desaturases and ORs based the RNAseq data on these transcriptomes and quantitative RT-PCR were in very good agreement justifying our comparison using only RNAseq information [27, 45, 46, 49].

The comparison of the transcriptomes of the pheromone glands and expression of mined genes did not reveal new genes thought to be involved in pheromone biosynthesis of these tortricid species. Of all curated candidate genes of the pheromone biosynthetic pathway that could play a role in pheromone differences, we found 11 genes with higher expression in pheromone glands than in antennae in *Ctenopseutis* and *Planotortrix* species. These included one β-oxidation enzyme, six fatty acid reductases, one very long chain fatty acids proteins, two fatty acid hydrolases and one carboxylesterase. Although two of these genes (ACOX3 and FAR2) have been found to be involved in sex pheromone phenotypic variation, β-oxidases in *Agrotis segetum* [110] and OscaFAR XIII in *Ostrinia scapulalis* and pgFAR in *B. mori* pgFAR which are orthologs of FAR2 [30, 31], none display differences in the expression pattern between species and signature of positive selection that could explain the different pheromones blend of the New Zealand species.

In the pheromone biosynthetic pathways, the only clear differences among these four species relies on the presence of specific desaturases and their differential expression. *Ctenopseustis herana*, *C. obliquana*, *P. excessana* and *P. octo* differ in the use of (*Z*)-tetradecenyl components of their sex pheromones, with *C. herana* producing Z5–14:OAc exclusively, whereas *C. obliquana* produces a blend of Z8–14-OAc and Z5–14:OAc, *P. excessana* a blend of Z5–14:OAc and Z7–14:OAc, and *P. octo* almost exclusively producing Z8–14:OAc. These compounds are produced by different desaturases encoded by different genes, which are present in all species (perhaps with the exception of *desat7*) but are differentially expressed in the pheromone glands. In *P. octo* and *C. obliquana* Z8–14:OAc is produced by Δ10-destauration by a desaturase encoded by the *desat5* gene that is down regulated in *C. herana* and *P. excessana* [27, 44]. The sex pheromone component Z5–14:OAc is part of the blend of both *Ctenopseustis* species and is produced by a desaturase with Δ5-destaurase activity encoded by the *desat7* gene [71]. This gene is highly expressed in both *Ctenopseustis* species but no orthologue is expressed in *P. excessana* despite the fact that this species makes use of Z5–14:OAc in its sex pheromone blend. We did not find an orthologue of *desat7* in the genome of both *P. octo* and *E. postivttana*, suggesting that *P. excessana* lacks this gene also. Hagström et al. [71] had shown that *Ctenopseustis* desat7 clusters distantly from the other classical lepidopteran desaturases. Here we reported several lepidopteran and other insect desaturase genes that could be orthologues of *desat7* (Fig. 5) suggesting an ancestral diversification with loss (or loss of function) of the gene in *Planotortrix* rather than a recent duplication in *Ctenopseustis*. The lack of *desat7* or at least its expression in *P. excessana* could be due to the biosynthesis of Z5–14:OAc in this species not requiring a specific pheromone gland desaturase. Instead Z5–14:OAc and Z7–14:OAc are most likely biosynthesized from the common monounsaturated fatty acids, oleic and palmitoleic and require two and one cycle of 2-carbon chain-shortening, respectively [70]. A Δ9-desaturase found in other tissues, other than pheromone glands, is probably involved in the biosynthesis of the oleate and palmitoleate moieties, precursors of Z5–14:OAc [70]. The Δ9-desaturases are encoded by *desat1* and *desat6* genes, however only the former was found to be expressed in *P. excessana* and no difference in expression were observed between species [44]. None of the desaturases displayed signatures of positive selection, however we cannot rule out that specific site mutations could be important in explaining the differences in the composition of the sex pheromones among these species. Experiments that manipulate the expression of the desaturases are required to test whether these expression differences are sufficient to generate the altered sex pheromone blend that are observed.

The comparison of male and female antennal transcriptomes revealed that only a few genes have biased expression in male antennae and three of them have some relationship with the pheromone components characteristic of the New Zealand moths. OR74 (OR7 in [45]) and OR30 were the only two genes with male biased expression in all New Zealand species. Three more ORs were male biased in only a single species, OR76a (OR1a in [45]) in *C. herana* compared with OR1 and OR44 in *C. obliquana*. These expression results were similar to those obtained with qPCR in [45, 46]. OR74 clustered with the pheromone receptors of other insects and has been shown to respond to Z8–14:OAc in both *Ctenopseustis* species. In *C. herana* OR74 also responded to Z7–14:OAc [45], but interestingly showed the lowest TPM among all species. Instead in *C. herana,* which exclusively uses Z5–14:OAc as its sex pheromone component, the ORs with the highest TPM were OR30 (TPM = 513 and LogFC = 8.01) and a duplicated gene of OR76 (OR76a: TPM 459 and logFC 4.46). Only the latter clusters in the sex pheromone receptor clade. OR30 was also highly expressed in *P. excessana* which uses both Z7–14:OAc and Z5–14:OAc as pheromone components, but in this species we found only a single copy of OR76. Despite this relationship between the expression of OR76a and OR30 with Z5–14:OAc, no ORs has been found to respond to Z5–14:OAc in cell-based functional analyses [45]. Recently a QTL-mapping approach in *Ostrina nubilalis* has revealed that several genes involved in neurogenesis may account for the difference in male response observed between the *E* and *Z* strains in addition to ORs [111]. Similar to the *Ostrinia* strains, the two *Ctenopsustis* species have swapped the neuronal identity of the pheromone-sensitive neurons, the Z5–14:OAc-responding OSN has a larger spike amplitude than the Z8–14:OAc-responding OSN in *C. herana*, whereas in *C. obliquana* the spike amplitude is reversed [112]. Therefore, a more complex system involving several genes may be operating in the response to different pheromone components in these tortricid moths. In *Drosophila* expression of ORs and hence the specificity of OSNs can depend on two POU transcription factors, the abnormal chemosensory jump 6 (*acj6*) and POU domain motif 3 (*pdm3*), and their interaction [113, 114]. Both genes are also expressed in the antennae of these tortricid moths and show higher, albeit not significant, expression in male versus female antennae. The expression and functional analysis of these genes needs further investigation.

The fundamental role of OBPs in the reception of odours is supported by several studies which have shown that the majority of these proteins are expressed in the antennae [84]. However, with the discovery of new OBPs by genomics it is becoming clear that many are not associated with the sensory organs and may have very different functions. Our study is not an exception, most of the OBPs were indeed expressed in the antennae. Three OBPs and most of CSPs, showed biased expression in pheromone glands. Two of these three OBPs belonged to the minus-C OBPs, which have been reported to have very broad expression in non-antennal tissues [88]. These “non-olfactory proteins” possess the same structure of olfactory OBPs and CSPs, respectively, making it reasonable to assume that they could be used for the transportation, protection from degradation and release into the environment of hydrophobic molecules [115]. The ability of CSPs to bind sex pheromone has been detected in the pheromone glands of *Mamestra brassicae* [116], suggesting a role in transporting pheromone for CSPs and OBPs expressed in the pheromone glands.

Odorant binding proteins including PBPs, GOBPs, ABPX3 and OBP18 are all highly expressed in the antennae of both males and females of the New Zealand tortricid moths. PBPs have been shown to solubilize hydrophobic pheromonal compounds, displaying distinct binding specificity [117], interacting differently with different pheromone components [118] and having an effect on the ligand specificity of ORs [119]. Three paralogous PBPs have typically been described from moths, including the close relative to the New Zealand tortricids, *E. postvittana* [49]. In the New Zealand tortricids, instead we found that all three PBPs seem duplicated and the divergence in all cases seems to predate the divergence of the two genera. In addition to the PBPs, a similar duplication event seems to have occurred for each of the two GOBPs. One possibility is that these different forms represent alleles. Newcomb et al. [89] described two forms of PBP1 in *E. postvittana*, a fast and a slow form, differing by 18 nucleotide mutations, which through some simple genetics were shown to be alleles of the same locus. In the phylogenetic tree (Fig. 8) the two alleles of EposPBP1 clustered with each other. In the New Zealand tortricids on the other hand, we identified three genes closely related to EposPBP1 (PBP1a, b and c), and two for both PBP2 and 3. These forms of PBP found in the New Zealand tortricids do not cluster with each other in the phylogeny containing a high number of substitutional differences and are therefore more likely to be different genes than alleles of the same locus. In other species where high quality genomic information is available, PBPs and GOBPs are often collocated in the genome so perhaps an entire section of the chromosome containing these five genes has been duplicated. These presumably duplicated PBP genes also have distinct expression patterns in male and female antennae, with the PBP1s and PBP3s usually expressed more highly in male antennae and the PBP2s displaying the opposite with higher expression in female antennae. Interestingly the different duplicated PBP genes also show some differences in expression between species. We are not aware of similar results in other moths. Whether this enlarged number of PBPs and differences in their expression between species allows for a greater distinction in binding specificity for different pheromonal compounds remains to be tested. However there are several indications to suggest the important role for PBPs in pheromone reception in these tortricid moths, with PBP1 and 3 having been shown to bind pheromone components in *E. postvittana* [89] as well as similar male biased expression also observed in *E. postvittana* [120] and in the two *Ctenopseustis* species. Furthermore, PBP1 from several species within *Ctenopseustis* and *Planotortrix* revealed rapid rates of sequence evolution along the lineages leading to those PBPs used by *C. filicis* and *C. fraternal*, species that utilize 16 carbon acetates instead of the 14 carbon acetates usually found among the members of the two genera [92, 93]. In comparison, we did not find clear evidence for positive selection among any of the PBPs. However all the species investigated in this study utilise pheromone components composed exclusively of 14 carbon acetates.

## Conclusions

From the global analyses of antennal and pheromone gland transcriptomes from these New Zealand endemic leafroller moths, only a few genes showing expression differences were identified and similarly when 300 genes involved in pheromone production and reception (including desaturases and ORs) were mined and manually curated, few showed biased expression mirroring the difference in pheromone composition of these species. Furthermore, none of the curated genes showed any clear signs of positive selection at the sequence level. While no clear evidence for selection was found using Codeml, there remains the possibility that sequence differences within some of these genes might confer differences in the mate recognition systems of these species. Certainly differences between species in pheromone biosynthesis seem to be explained predominantly by changes in the expression of two desaturases, *desat5* and *desat7*, with two genetic factors, one in *trans* and one in *cis*, regulating *desat5* [44]. More regulatory mutations may be expected to be able to fine tune expression in a set of standing genes in a gene family allowing a more rapid evolution than structural mutations that would impact the specificity of the enzyme [121]. In this way females could rapidly evolve changes in the type and composition of sex pheromones components in their blend. Intraspecific variation in female sex pheromones has been observed in several species both within and between populations [122, 123]. In males, we did not find a clear association between the expression of ORs and sex pheromone reception in the different species, suggesting that other genes could be involved. These tortricid species are characterized by a duplication of their PBP genes, with different paralogs varying in expression in male antennae of the different species. These PBP genes, together with the POU domain transcription factors, could have a role in modulating the specificity of OSNs and need further investigation. In conclusion, this study provides databases and some candidate genes potentially involved in the evolution of new pheromone systems in an enigmatic group of tortricid moths.

## Additional files

Additional file 1: Table S1. Number of raw and cleaned reads for each of mRNA library. (XLSX 9 kb)

Additional file 2: Table S2. Evidential gene cleansed transcriptomic summary statistics. (XLSX 9 kb)

Additional file 3: Table S3. Assembly statistics for the different algorithms and different kmer sizes used to assemble the transcriptomes of *Ctenopseustis herana*, *C. obliquana*, *Planotortrix excessana* and *P.octo*. (XLSX 14 kb)

Additional file 4: Table S4. Blast description, blast best hit, and GO annotation for each species of tortricid moths used in this work. (XLSX 34301 kb)

Additional file 5: Figure S1. Gene Ontology (GO) classifications of species of New Zealand tortricid moths according to biological processes, cellular component and molecular function. (TIFF 1218 kb)

Additional file 6: Table S5. List of groups of orthology obtained with ProteinOrtho. (XLSX 1587 kb)

Additional file 7: Table S6. List of genes with significant bias expression in the comparison of sex antennae and between female antennae and pheromone glands. (XLSX 62 kb)

Additional file 8: Table S7. Gene set enrichment analyses from the comparison of male and female antennae gene expression. GSA was obtained with Piano using the statistical method “mean”. GO terms were used to define gene sets. (XLSX 3609 kb)

Additional file 9: Figure S2. Consensus heatmaps of the GSA analyses of male and female antennae showing consensus scores, based on rank aggregation, for each directionality class (up = up regulated genes in male antennae, down = up regulated genes in female antennae). (TIFF 1567 kb)

Additional file 10: Table S8. Gene set enrichment analyses from the comparison of female antennae and pheromone glands gene expression. GSA was obtained with Piano using the statistical method “mean”. GO terms were used to define gene sets. (XLSX 3169 kb)

Additional file 11: Figure S3. Consensus heatmaps of the GSA analyses of female antennae and pheromone glands showing consensus scores, based on rank aggregation, for each directionality class (up = up regulated genes in female antennae, down = up regulated genes in pheromone glands). (TIFF 2099 kb)

Additional file 12:Table S9. List of manually curated genes and their expression values in the different tissues and species. (XLSX 231 kb)

Additional file 13: Table S10. Positive selection results on the manually curated genes. (XLSX 30 kb

Additional file 14: Nucleotide sequences of the manually mined genes. (DOCX 447 kb)

Additional file 15: Figure S4. Maximum likelihood tree showing the evolutionary relationships among acyl-CoA oxidase (ACOX) proteins mined from the transcriptomes of the New Zealand leafroller moths *Ctenopseustis herana* (Cher, highlighted in blue), *C. obliquana* (Cobl, highlighted in light purple), *Planotortrix excessana* (Pexc, highlighted in light green) and *P. octo* (Poct, highlighted in dark green) and the horticultural pest *Epiphyas postvittana* (Epos, highlighted in orange). Number at the nodes represent the Shimodaira-Hasegawa local support. The cluster (ACOX3a) shaded in orange showed bias expression in pheromone glands relative to antennae. (TIFF 301 kb)

Additional file 16: Figure S5. Maximum likelihood tree showing the evolutionary relationships among acetyl-CoA acetyltransferase (ACAT) proteins mined from the transcriptomes of the New Zealand leafroller moths *Ctenopseustis herana* (Cher, highlighted in blue), *C. obliquana* (Cobl, highlighted in light purple), *Planotortrix excessana* (Pexc, highlighted in light green) and *P. octo* (Poct, highlighted in dark green) and the horticultural pest *Epiphyas postvittana* (Epos, highlighted in orange). Circle size and colour at the nodes represent the Shimodaira-Hasegawa local support. Aips = *Agrotis ipsilum*, Bmor = *Bombyx mori*, Dmel = *Drosophila melanogaster*, Dple = *Danaus plexippus*, Hvir = *Heliothis virescens*, Hsub = *H. subflexa*, Osca = *Ostrinia scapulalis* and Pxyl = *Plutella xylostella*. (TIFF 431 kb)

Additional file 17: Figure S6. Maximum likelihood tree showing the evolutionary relationships among Acyl-CoA-binding proteins (ACBP) mined from the transcriptomes of the New Zealand leafroller moths *Ctenopseustis herana* (Cher, highlighted in blue), *C. obliquana* (Cobl, highlighted in light purple), *Planotortrix excessana* (Pexc, highlighted in light green) and *P. octo* (Poct, highlighted in dark green) and the horticultural pest *Epiphyas postvittana* (Epos, highlighted in orange). Circle size and colour at the nodes represent the Shimodaira-Hasegawa local support. Bmor = *Bombyx mori*, Dmel = *Drosophila melanogaster*, Dple = *Danaus plexippus*, Harm = *Helicoverpa armigera* and Pxyl = *Plutella xylostella*. (TIFF 430 kb)

Additional file 18: Figure S7. Maximum likelihood tree showing the evolutionary relationships among fatty acid transporter proteins (FATP). The tree includes the genes isolated from the New Zealand leafroller moths *Ctenopseustis herana* (Cher, highlighted in blue), *C. obliquana* (Cobl, highlighted in light purple), *Planotortrix excessana* (Pexc, highlighted in light green) and *P. octo* (Poct, highlighted in dark green) and the horticultural pest *Epiphyas postvittana* (Epos, highlighted in orange). Circle size and colour at the nodes represent the Shimodaira-Hasegawa local support. The clusters shaded in orange showed bias expression in PGs relative to antennae, whereas that in green showed bias in female antennae compared to PG. Bmor = *Bombyx mori*, Dmel = *Drosophila melanogaster*, Dple = *Danaus plexippus* and Pxyl = *Plutella xylostella*. (TIFF 450 kb)

Additional file 19: Figure S8. Maximum likelihood tree showing the evolutionary relationships among 1-acyl-sn-glycerol-3-phosphate acyltransferase (AGPAT). The tree includes the genes isolated from the New Zealand leafroller moths *Ctenopseustis herana* (Cher, highlighted in blue), *C. obliquana* (Cobl, highlighted in light purple), *Planotortrix excessana* (Pexc, highlighted in light green) and *P. octo* (Poct, highlighted in dark green) and the horticultural pest *Epiphyas postvittana* (Epos, highlighted in orange). Circle size and colour at the nodes represent the Shimodaira-Hasegawa local support. The cluster shaded in orange showed bias expression in PGs relative to antennae, whereas that in green showed bias in female antennae compared to PG. Bmor = *Bombyx mori*, Dmel = *Drosophila melanogaster*, Dple = *Danaus plexippus*, Hvir = *Heliothis virescens* and Pxyl = *Plutella xylostella*. (TIFF 473 kb)

Additional file 20: Figure S9. Maximum likelihood tree showing the evolutionary relationships among elongation of very long chain fatty acids proteins (ELOVL). The tree includes the genes isolated from the New Zealand leafroller moths *Ctenopseustis herana* (Cher, highlighted in blue), *C. obliquana* (Cobl, highlighted in light purple), *Planotortrix excessana* (Pexc, highlighted in light green) and *P. octo* (Poct, highlighted in dark green) and the horticultural pest *Epiphyas postvittana* (Epos, highlighted in orange). Circle size and colour at the nodes represent the Shimodaira-Hasegawa local support. Bmor = *Bombyx mori*, Dmel = *Drosophila melanogaster*, Dple = *Danaus plexippus* and Pxyl = *Plutella xylostella*. (TIFF 585 kb)

Additional file 21: Figure S10. Maximum likelihood tree showing the evolutionary relationships among fatty acid transferases (FAT). The tree includes the genes isolated from the New Zealand leafroller moths *Ctenopseustis herana* (Cher, highlighted in blue), *C. obliquana* (Cobl, highlighted in light purple), *Planotortrix excessana* (Pexc, highlighted in light green) and *P. octo* (Poct, highlighted in dark green) and the horticultural pest *Epiphyas postvittana* (Epos, highlighted in orange). Circle size and colour at the nodes represent the Shimodaira-Hasegawa local support. The clusters shaded in orange showed bias expression in pheromone glands relative to antennae, whereas those shaded in green showed bias in female antennae relative to pheromone glands. Aips = *Agrotis ipsilon*, Bmor = *Bombyx mori*, Dmel = *Drosophila melanogaster*, Dple = *Danaus plexippus*, Hvir = *Heliothis virescens*, Osca = *Ostrinia scapulalis* and Pxyl = *Plutella xylostella*. (TIFF 906 kb)

Additional file 22: Figure S11. Maximum likelihood tree showing the evolutionary relationships among fatty acid hydrolases (FAAH). The tree includes the genes isolated from the New Zealand leafroller moths *Ctenopseustis herana* (Cher, highlighted in blue), *C. obliquana* (Cobl, highlighted in light purple), *Planotortrix excessana* (Pexc, highlighted in light green) and *P. octo* (Poct, highlighted in dark green) and the horticultural pest *Epiphyas postvittana* (Epos, highlighted in orange). Circle size and colour at the nodes represent the Shimodaira-Hasegawa local support. Bmor = *Bombyx mori*, Dmel = *Drosophila melanogaster*, Dple = *Danaus plexippus*, and Pxyl = *Plutella xylostella*. (TIFF 433 kb)

Additional file 23: Figure S12. Maximum likelihood tree showing the evolutionary relationships among chemosensory proteins (CSPs). The tree includes the genes isolated from the New Zealand leafroller moths *Ctenopseustis herana* (Cher, highlighted in blue), *C. obliquana* (Cobl, highlighted in light purple), *Planotortrix excessana* (Pexc, highlighted in light green) and *P. octo* (Poct, highlighted in dark green) and the horticultural pest *Epiphyas postvittana* (Epos, highlighted in orange). Circle at the nodes represent the Shimodaira-Hasegawa local support. Colours and size of circles are proportional to the percentage of support (0–100%). Groups shades in orange were pheromone gland biased. Bmor = *Bombyx mori*, Dmel = *Drosophila melanogaster*, Harm = *Helicoverpa armigera*, Hvir = *Heliothis virescens* and Pxyl = *Plutella xylostella*. (TIFF 968 kb)

Additional file 24: Figure S13. Maximum likelihood tree showing the evolutionary relationships among sensory neuron membrane proteins (SNMPs). The tree also includes the CD36 genes of *Drosophila melanogaster* (Dmel). Numbers at the nodes represent the Shimodaira-Hasegawa local support. Cher = *Ctenopseustis herana* (highlighted in blue), Cobl = *C. obliquana* (highlighted in light purple), Pexc = *Planotortrix excessana* (highlighted in light green), Poct = *P. octo* (highlighted in dark green), Epos = *Epiphyas postvittana* (highlighted in orange), Aips = *Agrotis ipsilum*, Bmor = *Bombyx mori*, Dple = *Danaus plexippus*, Hvir = *Heliothis virescens*, Pxyl = *Plutella xylostella*. (TIFF 486 kb)

Additional file 25: Figure S14. Maximum likelihood tree showing the evolutionary relationships among ionotropic receptors (IRs). The tree includes the genes isolated from the New Zealand leafroller moths *Ctenopseustis herana* (Cher, highlighted in blue), *C. obliquana* (Cobl, highlighted in light purple), *Planotortrix excessana* (Pexc, highlighted in light green) and *P. octo* (Poct, highlighted in dark green) and the horticultural pest *Epiphyas postvittana* (Epos, highlighted in orange). Circle at the nodes represent the Shimodaira-Hasegawa local support. Colour and size of circle is proportional to the percentage of support (0–100%). Groups shades in orange were pheromone gland biased. Bmor = *Bombyx mori* and Dmel = *Drosophila melanogaster*. (TIFF 1441 kb)

## Abbreviations

ACBP: Acyl-CoA-binding protein
ACC: Acetyl-CoA carboxylase
ACOX: Acyl-CoA oxidase
AGPAT: 1-acyl-sn-glycerol-3-phosphate acyltransferase
AMPA: α-amino-3-hydroxy-5-methyl-4-isoxazolepropionic acid receptor
CSP: Chemosensory protein
CXE: Carboxylesterase
ELOVL: Very long chain fatty acids proteins
FAAH: Fatty acid amide hydrolase
FAR: Fatty acid reductase
FAS: Fatty acid synthase
FAT: Fatty acid transferase
FATP: Fatty acid transport protein
GO: Gene Ontology
GOBP: General odorant binding protein
GSA: Gene Set Analysis
IR: Ionotropic receptor
JHE: Juvenile hormone esterase
LRT: Likelihood ratio test
OBP: Odorant binding protein
OSN: Olfactory sensory neuron
PBAN: Pheromone biosynthesis activating neuropeptide
PBP: Pheromone binding protein
PG: Pheromone glands
SNMP: Sensory neuron membrane protein
TPM: Transcripts Per Kilobase Million
