# Supplementary figures and images for: Differential gene expression in the evolution of sex pheromone communication in New Zealand’s endemic leafroller moths of the genera Ctenopseustis and Planotortrix
Source: BMC Genomics. 2018 Jan 26;19:94. doi: 10.1186/s12864-018-4451-1 (PMC5787247; doi:10.1186/s12864-018-4451-1)

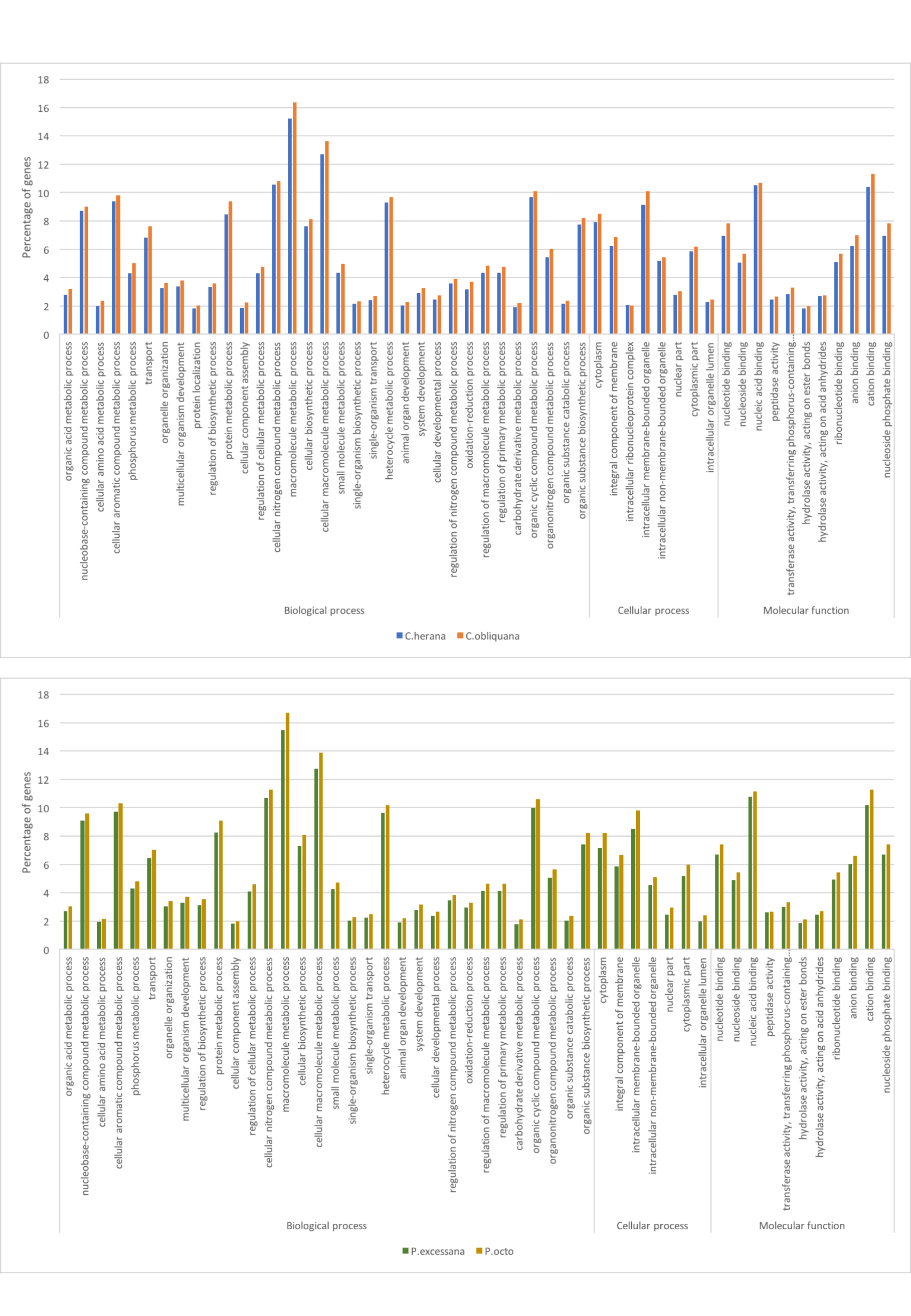

Supplement: Supplementary file 5 — Gene Ontology (GO) classifications of species of New Zealand tortricid moths according to biological processes, cellular component and molecular function. (TIFF 1218 kb) [file 12864_2018_4451_MOESM5_ESM.tif]

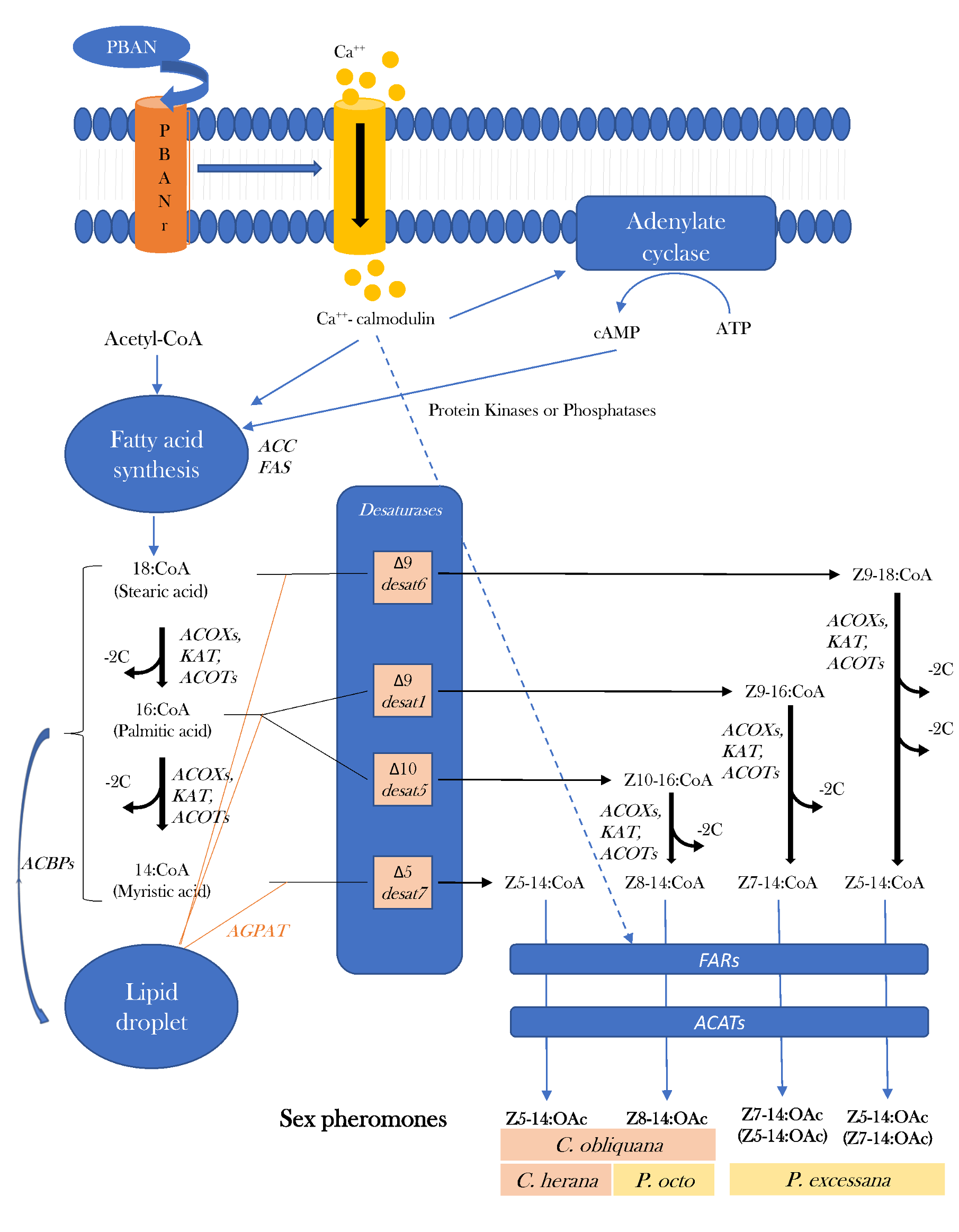

Supplement: Supplementary file 9 — Consensus heatmaps of the GSA analyses of male and female antennae showing consensus scores, based on rank aggregation, for each directionality class (up = up regulated genes in male antennae, down = up regulated genes in female antennae). (TIFF 1567 kb) [file 12864_2018_4451_MOESM9_ESM.tif]

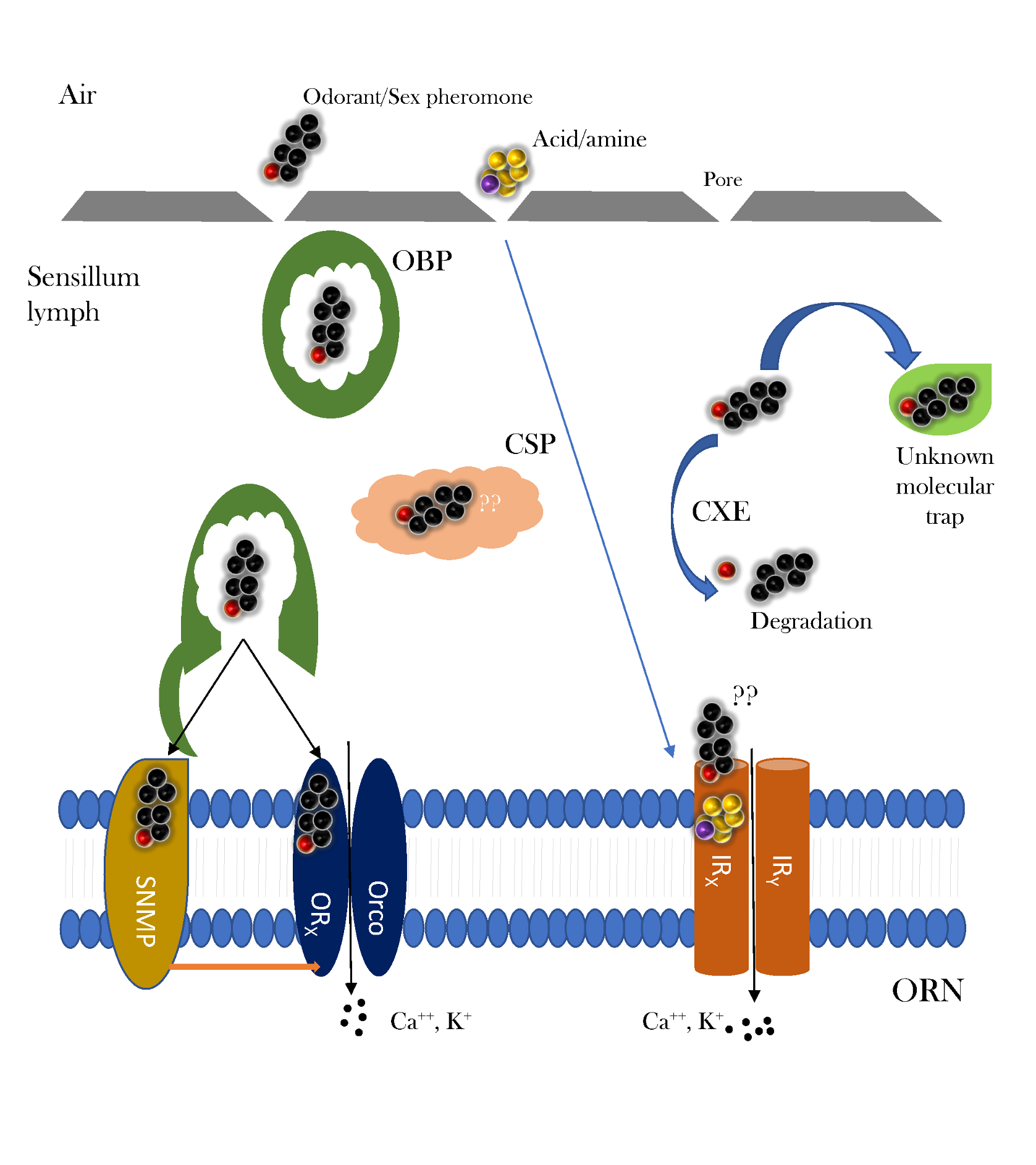

Supplement: Supplementary file 11 — Consensus heatmaps of the GSA analyses of female antennae and pheromone glands showing consensus scores, based on rank aggregation, for each directionality class (up = up regulated genes in female antennae, down = up regulated genes in pheromone glands). (TIFF 2099 kb) [file 12864_2018_4451_MOESM11_ESM.tif]

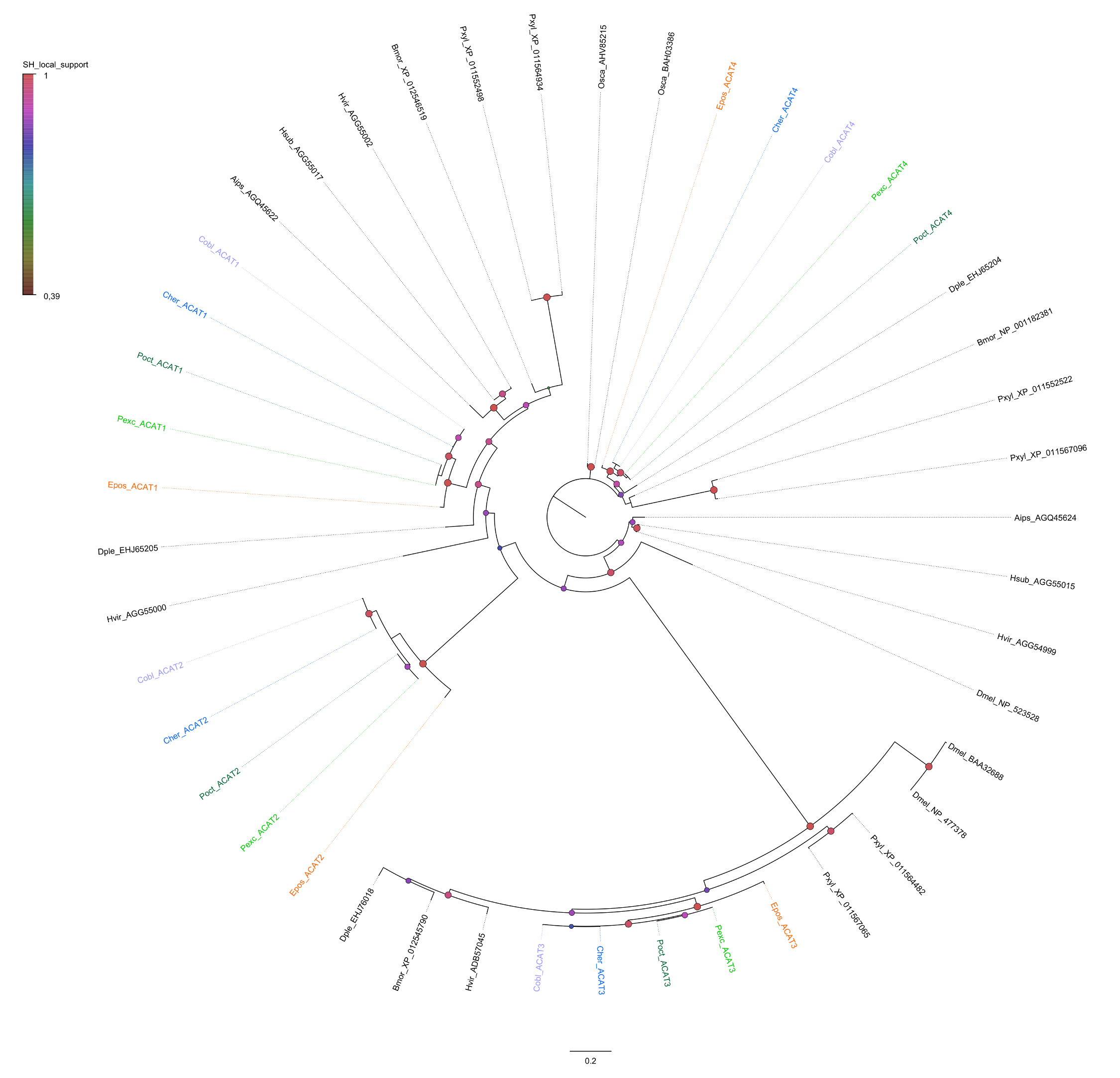

Supplement: Supplementary file 15 — Maximum likelihood tree showing the evolutionary relationships among acyl-CoA oxidase (ACOX) proteins mined from the transcriptomes of the New Zealand leafroller moths Ctenopseustis herana (Cher, highlighted in blue), C. obliquana (Cobl, highlighted in light purple), Planotortrix excessana (Pexc, highlighted in light green) and P. octo (Poct, highlighted in dark green) and the horticultural pest Epiphyas postvittana (Epos, highlighted in orange). Number at the nodes represent the Shimodaira-Hasegawa local support. The cluster (ACOX3a) shaded in orange showed bias expression in pheromone glands relative to antennae. (TIFF 301 kb) [file 12864_2018_4451_MOESM15_ESM.tif]

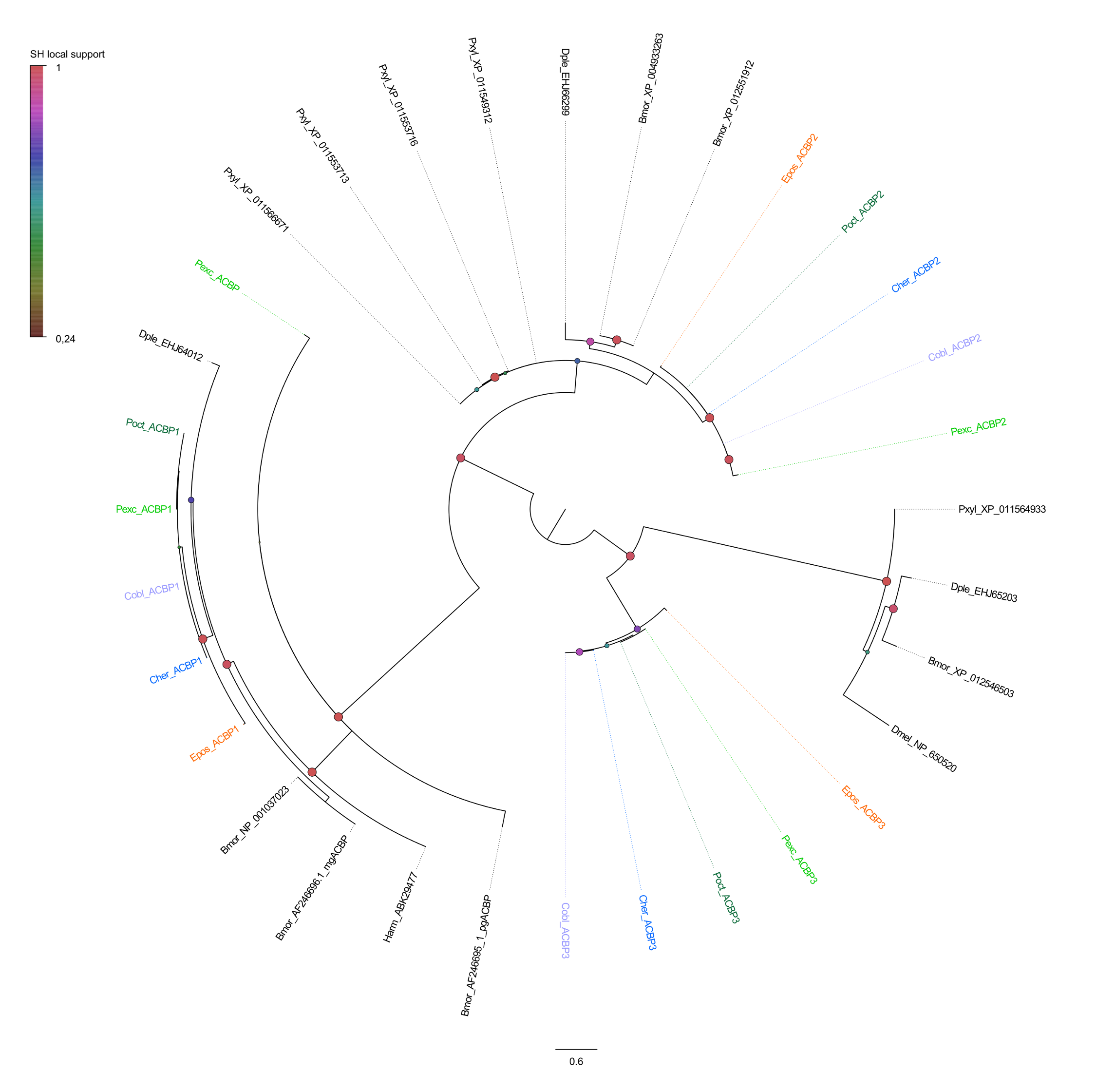

Supplement: Supplementary file 16 — Maximum likelihood tree showing the evolutionary relationships among acetyl-CoA acetyltransferase (ACAT) proteins mined from the transcriptomes of the New Zealand leafroller moths Ctenopseustis herana (Cher, highlighted in blue), C. obliquana (Cobl, highlighted in light purple), Planotortrix excessana (Pexc, highlighted in light green) and P. octo (Poct, highlighted in dark green) and the horticultural pest Epiphyas postvittana (Epos, highlighted in orange). Circle size and colour at the nodes represent the Shimodaira-Hasegawa local support. Aips = Agrotis ipsilum, Bmor = Bombyx mori, Dmel = Drosophila melanogaster, Dple = Danaus plexippus, Hvir = Heliothis virescens, Hsub = H. subflexa, Osca = Ostrinia scapulalis and Pxyl = Plutella xylostella. (TIFF 431 kb) [file 12864_2018_4451_MOESM16_ESM.tif]

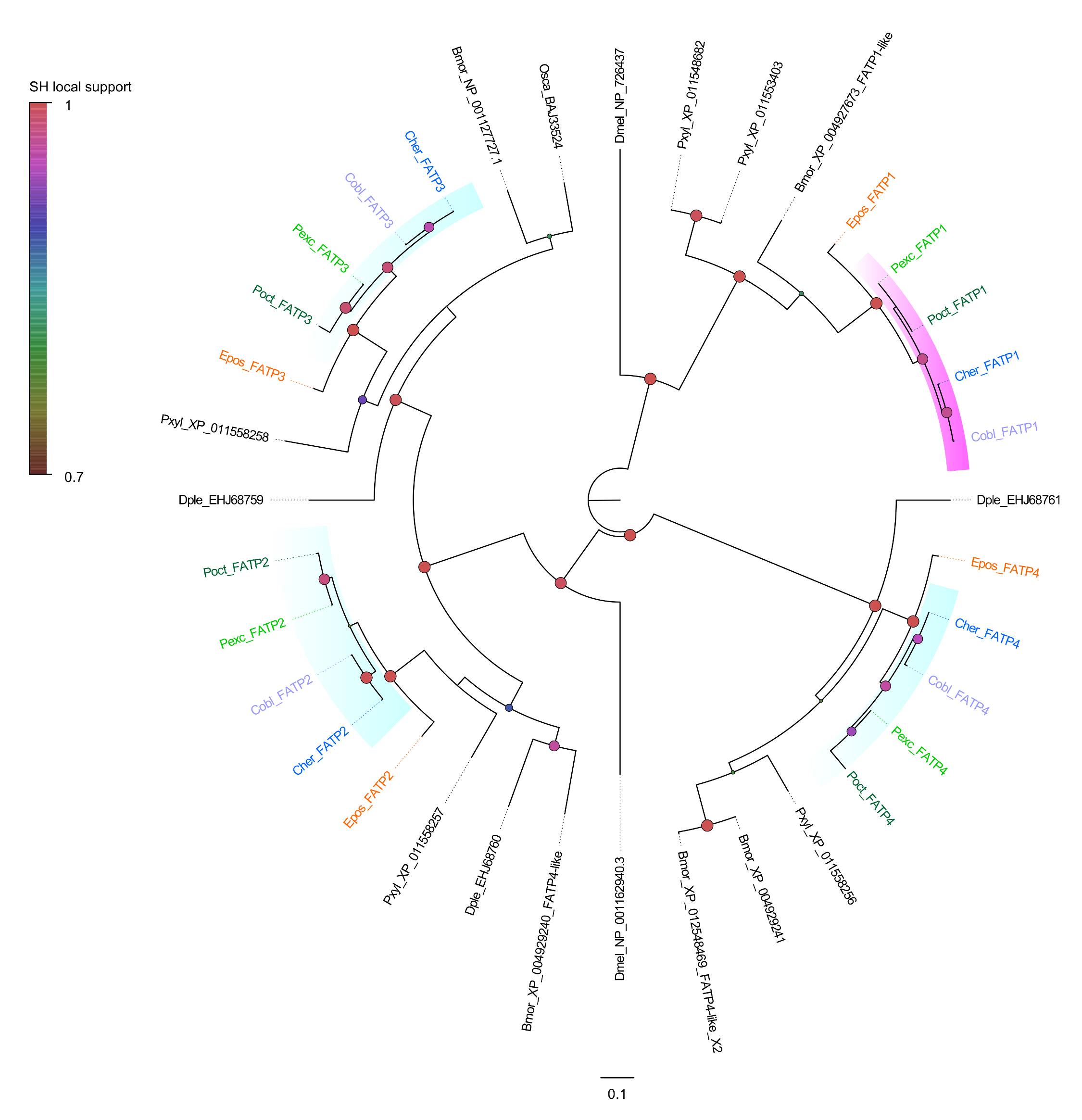

Supplement: Supplementary file 17 — Maximum likelihood tree showing the evolutionary relationships among Acyl-CoA-binding proteins (ACBP) mined from the transcriptomes of the New Zealand leafroller moths Ctenopseustis herana (Cher, highlighted in blue), C. obliquana (Cobl, highlighted in light purple), Planotortrix excessana (Pexc, highlighted in light green) and P. octo (Poct, highlighted in dark green) and the horticultural pest Epiphyas postvittana (Epos, highlighted in orange). Circle size and colour at the nodes represent the Shimodaira-Hasegawa local support. Bmor = Bombyx mori, Dmel = Drosophila melanogaster, Dple = Danaus plexippus, Harm = Helicoverpa armigera and Pxyl = Plutella xylostella. (TIFF 430 kb) [file 12864_2018_4451_MOESM17_ESM.tif]

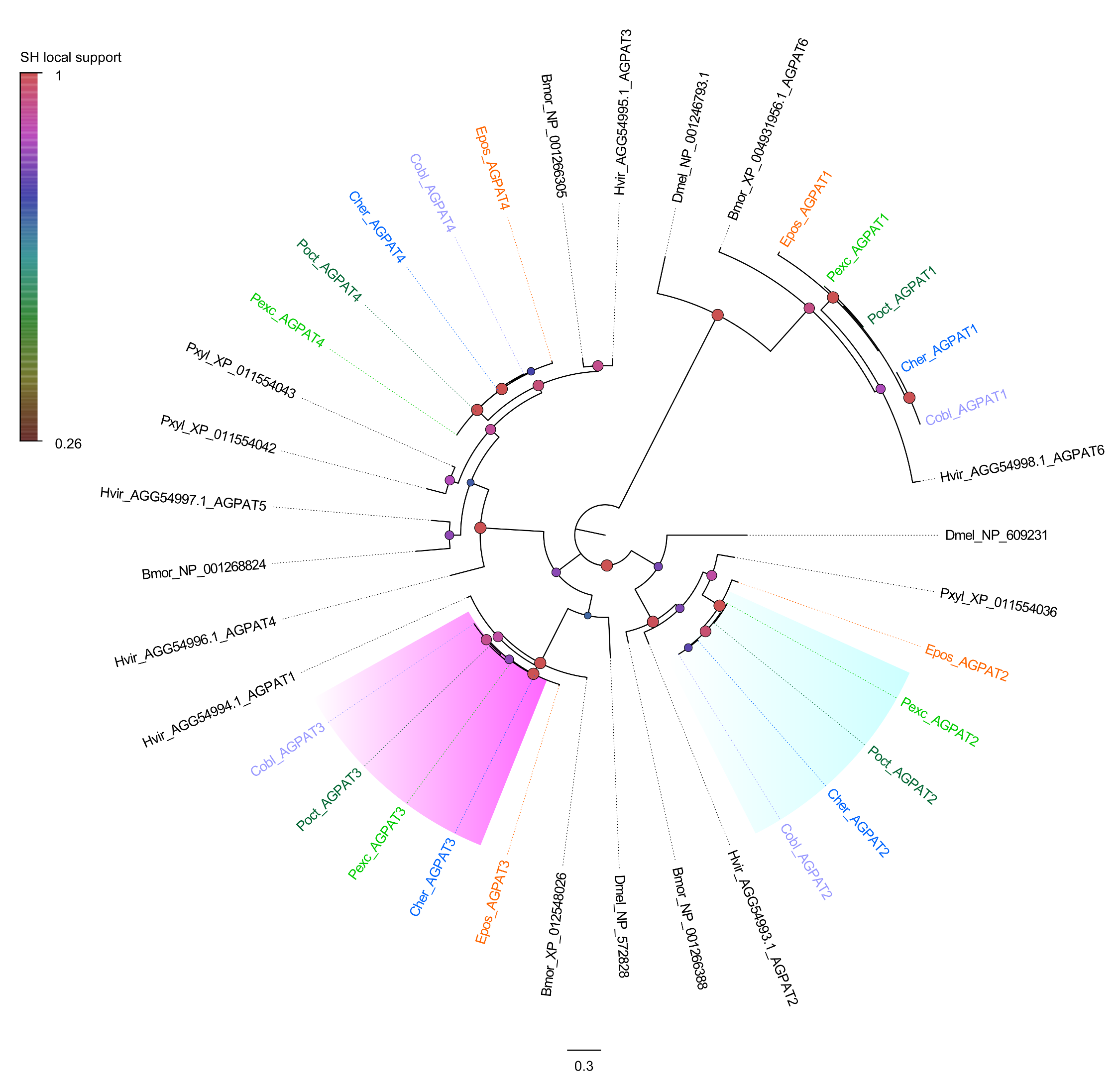

Supplement: Supplementary file 18 — Maximum likelihood tree showing the evolutionary relationships among fatty acid transporter proteins (FATP). The tree includes the genes isolated from the New Zealand leafroller moths Ctenopseustis herana (Cher, highlighted in blue), C. obliquana (Cobl, highlighted in light purple), Planotortrix excessana (Pexc, highlighted in light green) and P. octo (Poct, highlighted in dark green) and the horticultural pest Epiphyas postvittana (Epos, highlighted in orange). Circle size and colour at the nodes represent the Shimodaira-Hasegawa local support. The clusters shaded in orange showed bias expression in PGs relative to antennae, whereas that in green showed bias in female antennae compared to PG. Bmor = Bombyx mori, Dmel = Drosophila melanogaster, Dple = Danaus plexippus and Pxyl = Plutella xylostella. (TIFF 450 kb) [file 12864_2018_4451_MOESM18_ESM.tif]

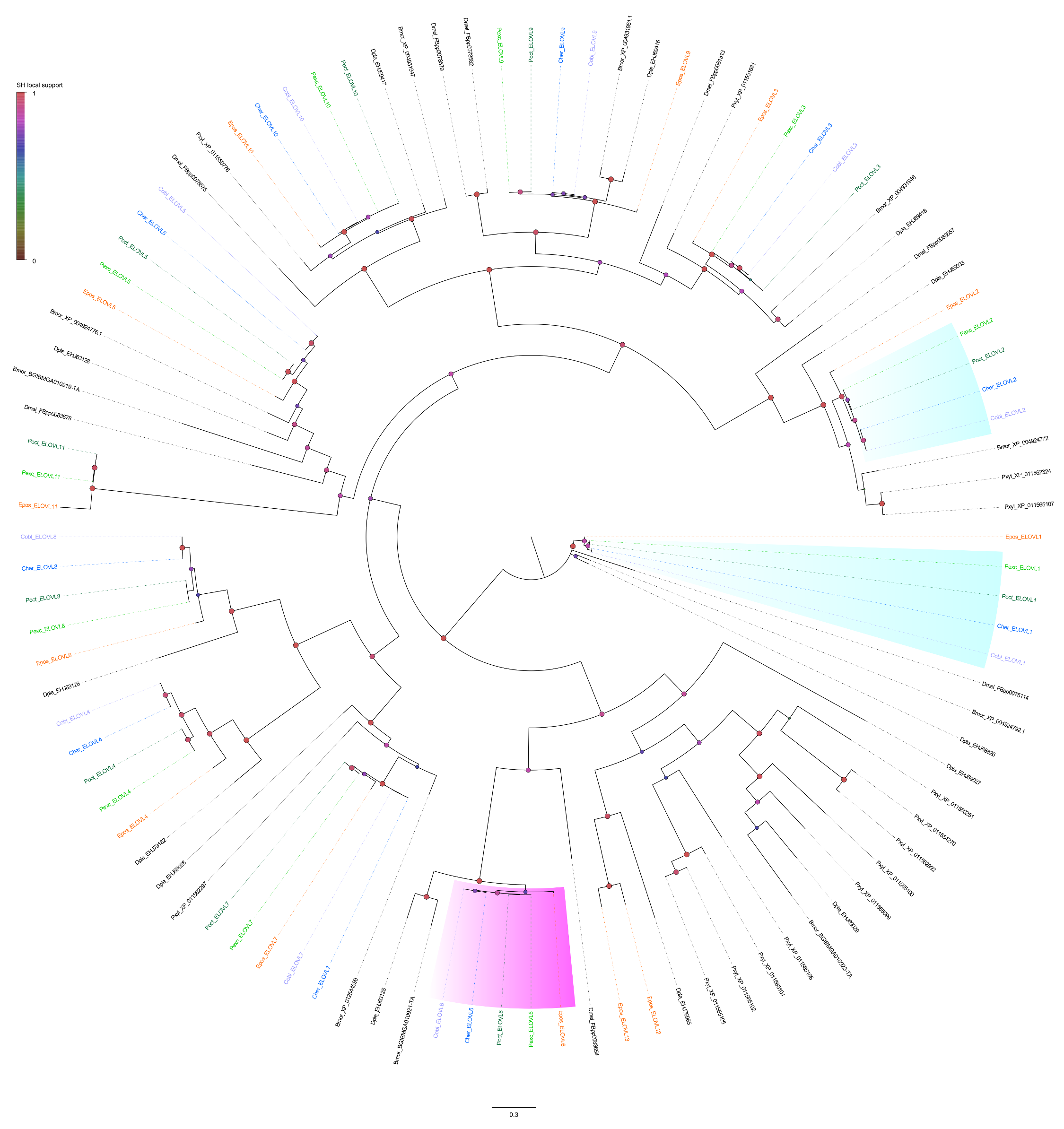

Supplement: Supplementary file 19 — Maximum likelihood tree showing the evolutionary relationships among 1-acyl-sn-glycerol-3-phosphate acyltransferase (AGPAT). The tree includes the genes isolated from the New Zealand leafroller moths Ctenopseustis herana (Cher, highlighted in blue), C. obliquana (Cobl, highlighted in light purple), Planotortrix excessana (Pexc, highlighted in light green) and P. octo (Poct, highlighted in dark green) and the horticultural pest Epiphyas postvittana (Epos, highlighted in orange). Circle size and colour at the nodes represent the Shimodaira-Hasegawa local support. The cluster shaded in orange showed bias expression in PGs relative to antennae, whereas that in green showed bias in female antennae compared to PG. Bmor = Bombyx mori, Dmel = Drosophila melanogaster, Dple = Danaus plexippus, Hvir = Heliothis virescens and Pxyl = Plutella xylostella. (TIFF 473 kb) [file 12864_2018_4451_MOESM19_ESM.tif]

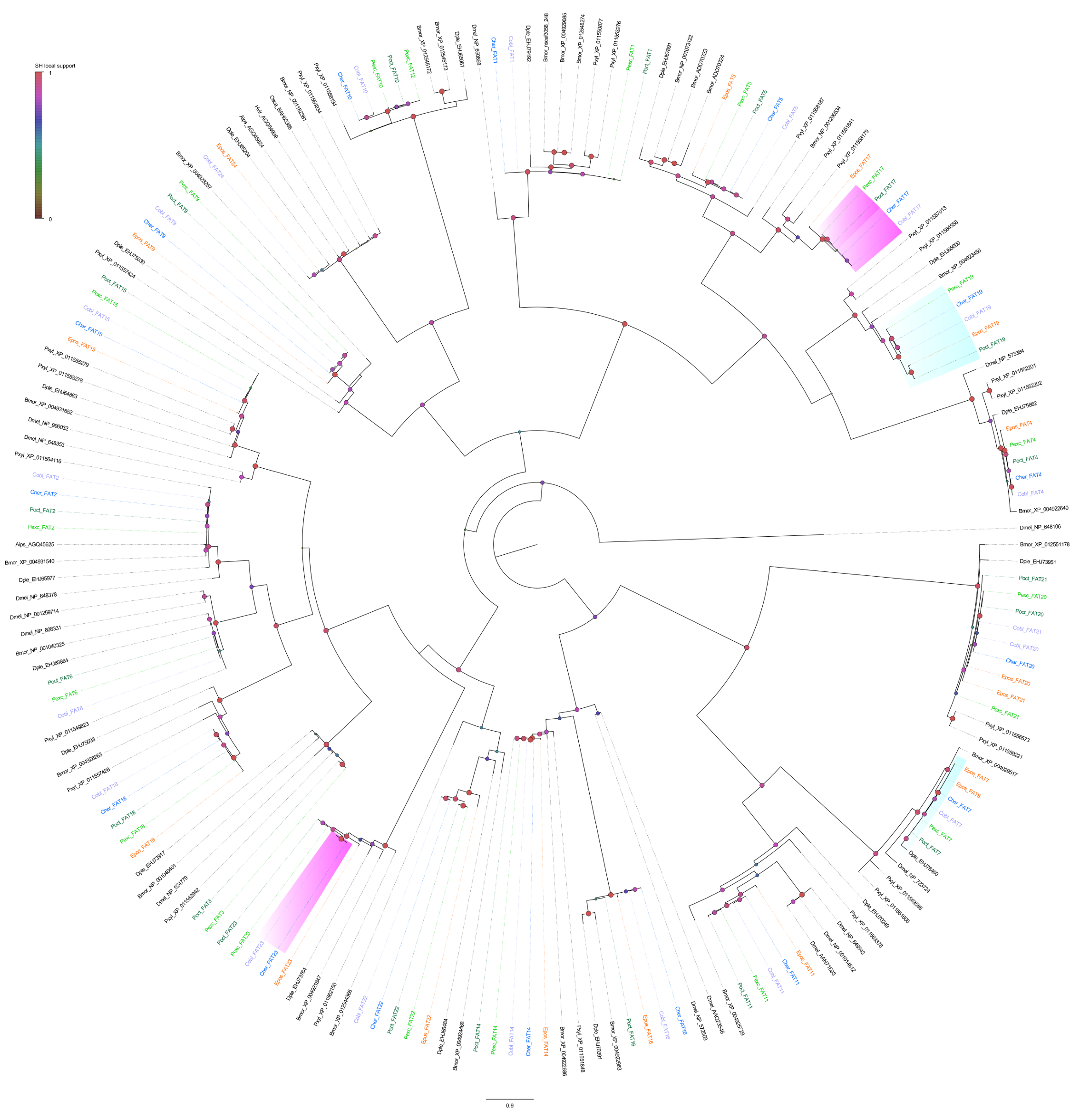

Supplement: Supplementary file 20 — Maximum likelihood tree showing the evolutionary relationships among elongation of very long chain fatty acids proteins (ELOVL). The tree includes the genes isolated from the New Zealand leafroller moths Ctenopseustis herana (Cher, highlighted in blue), C. obliquana (Cobl, highlighted in light purple), Planotortrix excessana (Pexc, highlighted in light green) and P. octo (Poct, highlighted in dark green) and the horticultural pest Epiphyas postvittana (Epos, highlighted in orange). Circle size and colour at the nodes represent the Shimodaira-Hasegawa local support. Bmor = Bombyx mori, Dmel = Drosophila melanogaster, Dple = Danaus plexippus and Pxyl = Plutella xylostella. (TIFF 585 kb) [file 12864_2018_4451_MOESM20_ESM.tif]

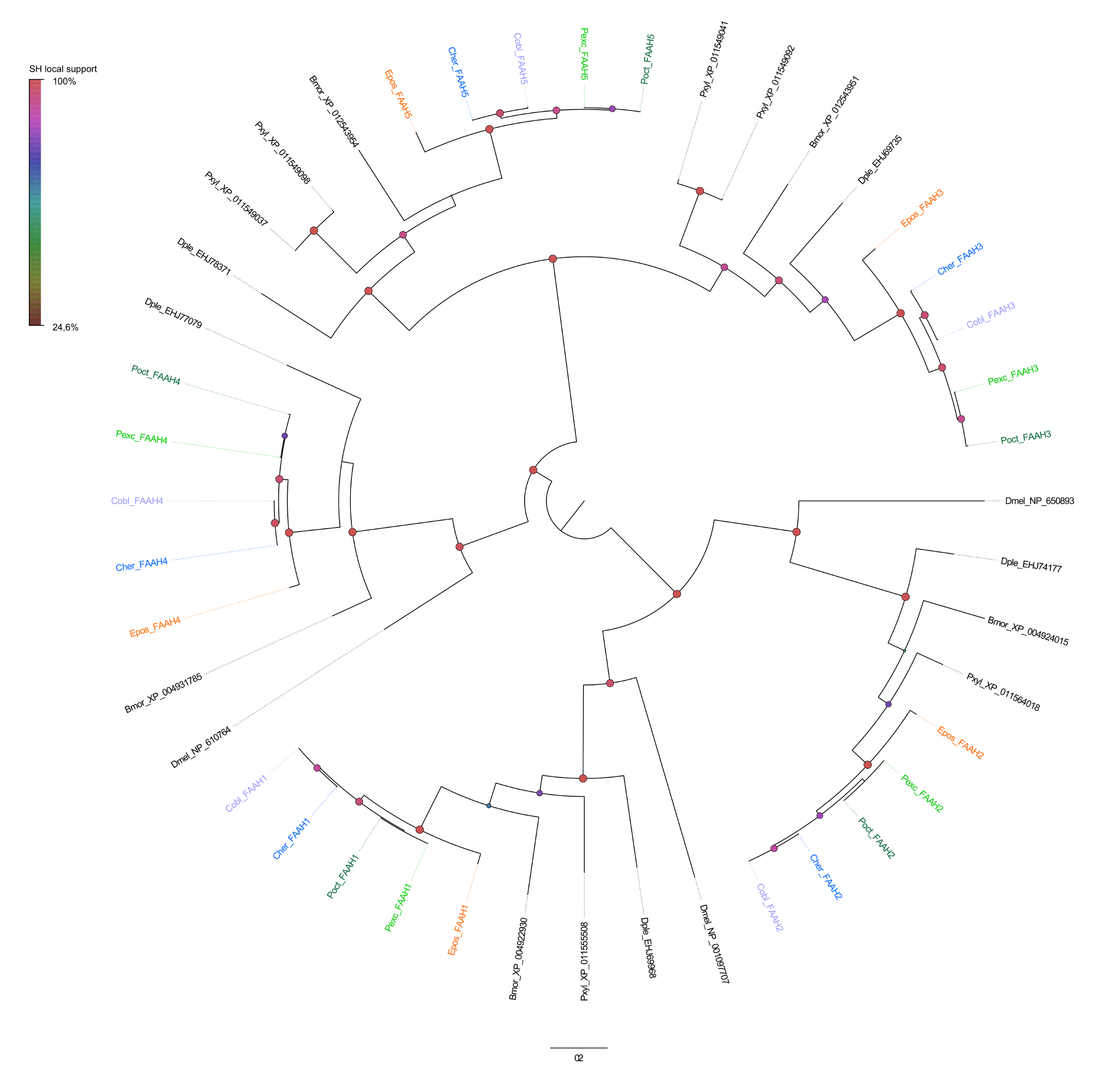

Supplement: Supplementary file 21 — Maximum likelihood tree showing the evolutionary relationships among fatty acid transferases (FAT). The tree includes the genes isolated from the New Zealand leafroller moths Ctenopseustis herana (Cher, highlighted in blue), C. obliquana (Cobl, highlighted in light purple), Planotortrix excessana (Pexc, highlighted in light green) and P. octo (Poct, highlighted in dark green) and the horticultural pest Epiphyas postvittana (Epos, highlighted in orange). Circle size and colour at the nodes represent the Shimodaira-Hasegawa local support. The clusters shaded in orange showed bias expression in pheromone glands relative to antennae, whereas those shaded in green showed bias in female antennae relative to pheromone glands. Aips = Agrotis ipsilon, Bmor = Bombyx mori, Dmel = Drosophila melanogaster, Dple = Danaus plexippus, Hvir = Heliothis virescens, Osca = Ostrinia scapulalis and Pxyl = Plutella xylostella. (TIFF 906 kb) [file 12864_2018_4451_MOESM21_ESM.tif]

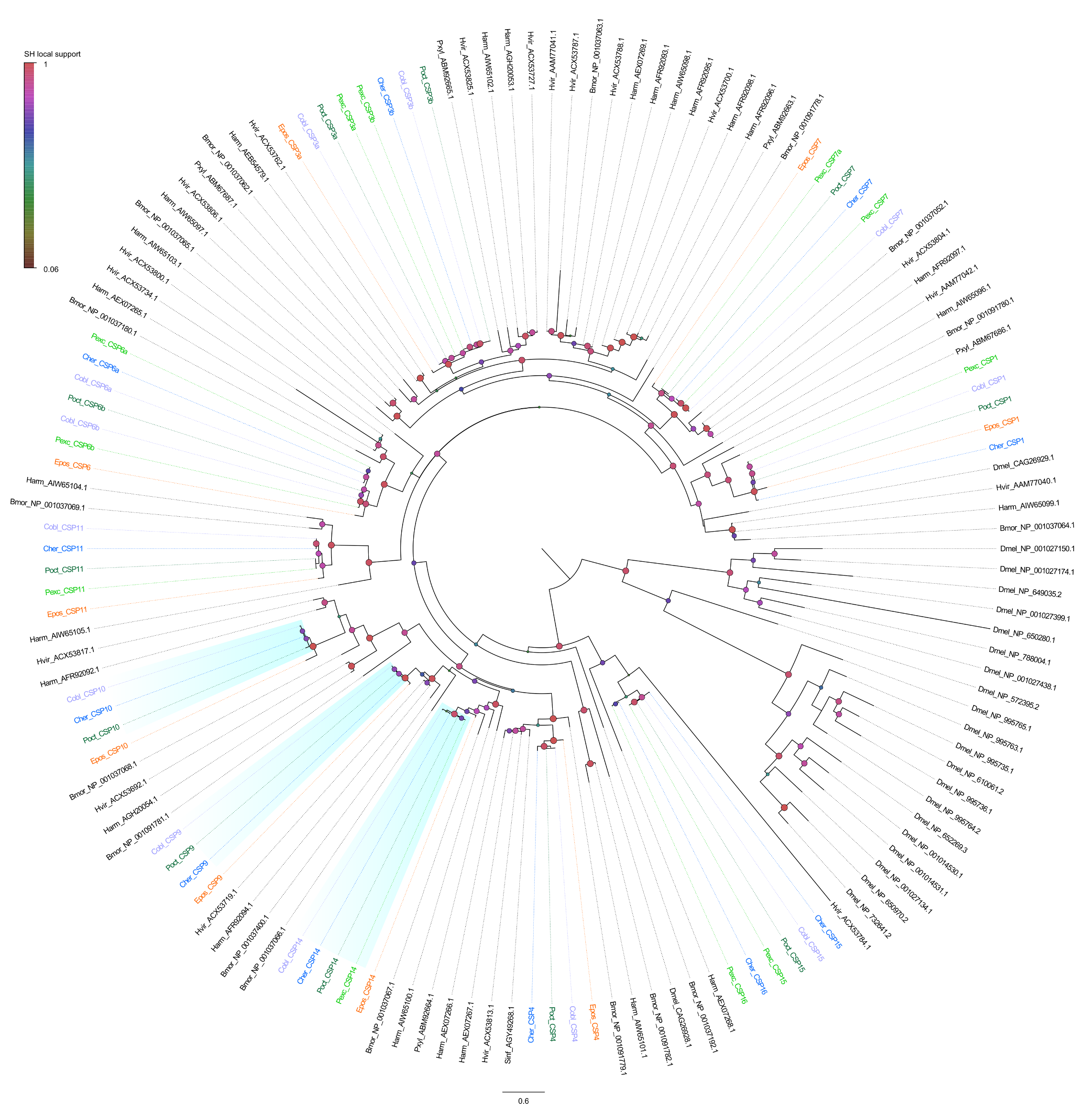

Supplement: Supplementary file 22 — Maximum likelihood tree showing the evolutionary relationships among fatty acid hydrolases (FAAH). The tree includes the genes isolated from the New Zealand leafroller moths Ctenopseustis herana (Cher, highlighted in blue), C. obliquana (Cobl, highlighted in light purple), Planotortrix excessana (Pexc, highlighted in light green) and P. octo (Poct, highlighted in dark green) and the horticultural pest Epiphyas postvittana (Epos, highlighted in orange). Circle size and colour at the nodes represent the Shimodaira-Hasegawa local support. Bmor = Bombyx mori, Dmel = Drosophila melanogaster, Dple = Danaus plexippus, and Pxyl = Plutella xylostella. (TIFF 433 kb) [file 12864_2018_4451_MOESM22_ESM.tif]

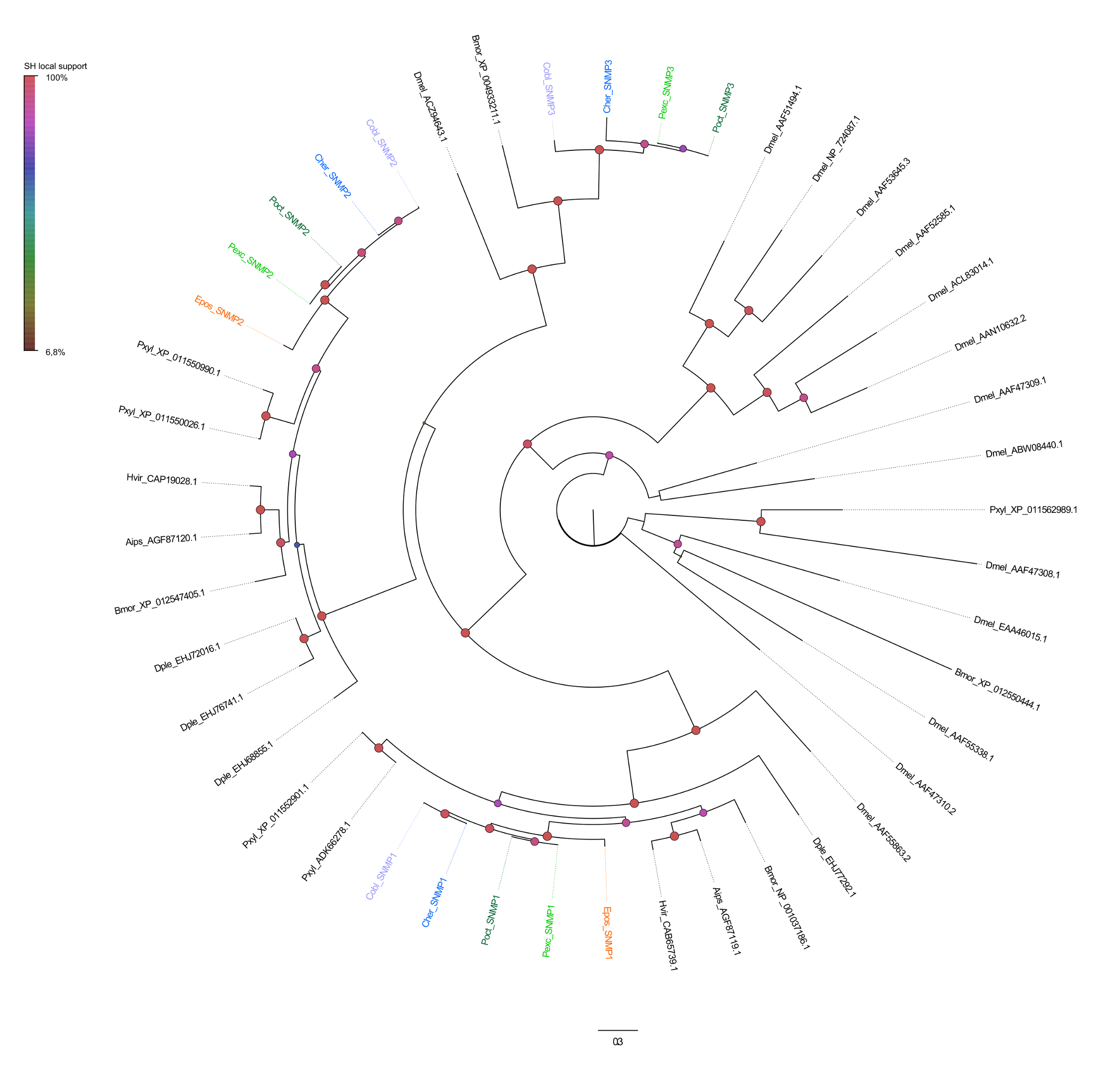

Supplement: Supplementary file 23 — Maximum likelihood tree showing the evolutionary relationships among chemosensory proteins (CSPs). The tree includes the genes isolated from the New Zealand leafroller moths Ctenopseustis herana (Cher, highlighted in blue), C. obliquana (Cobl, highlighted in light purple), Planotortrix excessana (Pexc, highlighted in light green) and P. octo (Poct, highlighted in dark green) and the horticultural pest Epiphyas postvittana (Epos, highlighted in orange). Circle at the nodes represent the Shimodaira-Hasegawa local support. Colours and size of circles are proportional to the percentage of support (0–100%). Groups shades in orange were pheromone gland biased. Bmor = Bombyx mori, Dmel = Drosophila melanogaster, Harm = Helicoverpa armigera, Hvir = Heliothis virescens and Pxyl = Plutella xylostella. (TIFF 968 kb) [file 12864_2018_4451_MOESM23_ESM.tif]

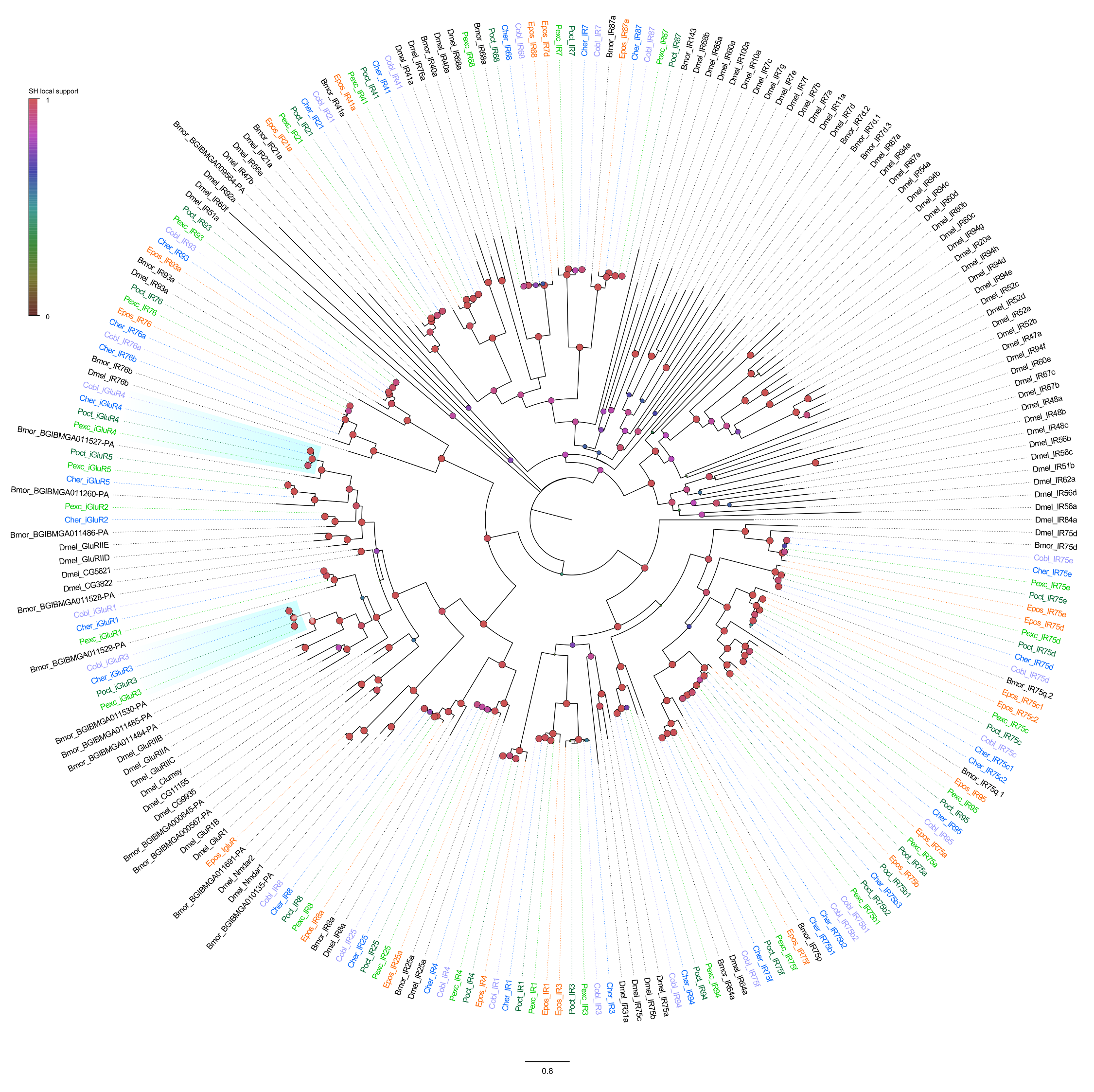

Supplement: Supplementary file 24 — Maximum likelihood tree showing the evolutionary relationships among sensory neuron membrane proteins (SNMPs). The tree also includes the CD36 genes of Drosophila melanogaster (Dmel). Numbers at the nodes represent the Shimodaira-Hasegawa local support. Cher = Ctenopseustis herana (highlighted in blue), Cobl = C. obliquana (highlighted in light purple), Pexc = Planotortrix excessana (highlighted in light green), Poct = P. octo (highlighted in dark green), Epos = Epiphyas postvittana (highlighted in orange), Aips = Agrotis ipsilum, Bmor = Bombyx mori, Dple = Danaus plexippus, Hvir = Heliothis virescens, Pxyl = Plutella xylostella. (TIFF 486 kb) [file 12864_2018_4451_MOESM24_ESM.tif]

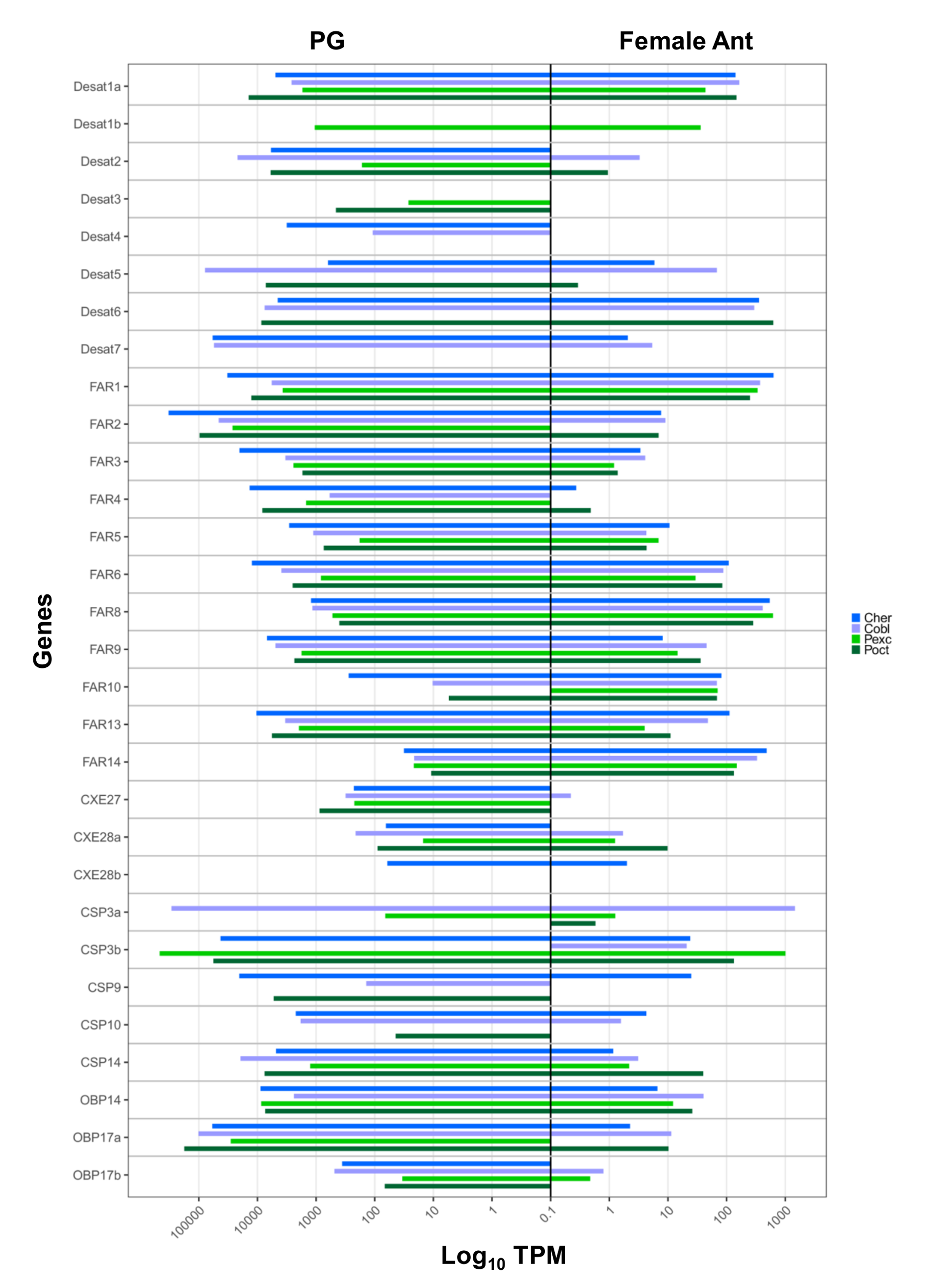

Supplement: Supplementary file 25 — Maximum likelihood tree showing the evolutionary relationships among ionotropic receptors (IRs). The tree includes the genes isolated from the New Zealand leafroller moths Ctenopseustis herana (Cher, highlighted in blue), C. obliquana (Cobl, highlighted in light purple), Planotortrix excessana (Pexc, highlighted in light green) and P. octo (Poct, highlighted in dark green) and the horticultural pest Epiphyas postvittana (Epos, highlighted in orange). Circle at the nodes represent the Shimodaira-Hasegawa local support. Colour and size of circle is proportional to the percentage of support (0–100%). Groups shades in orange were pheromone gland biased. Bmor = Bombyx mori and Dmel = Drosophila melanogaster. (TIFF 1441 kb) [file 12864_2018_4451_MOESM25_ESM.tif]
