# Supplementary material for: Differential gene expression in the evolution of sex pheromone communication in New Zealand’s endemic leafroller moths of the genera Ctenopseustis and Planotortrix
Source: BMC Genomics. 2018 Jan 26;19:94. doi: 10.1186/s12864-018-4451-1 (PMC5787247; doi:10.1186/s12864-018-4451-1)
Supplement: Supplementary file 14 — Nucleotide sequences of the manually mined genes. (DOCX 447 kb) [file 12864_2018_4451_MOESM14_ESM.docx]

>Cher_ABPX1a

ATGTCAGGACACGCAGCACTCTGCTGTGTCGTGGTGGCAGCATTTGTGCTGGGCGCCCACTGCATGGACGAAGAGATGGC

CGAGCTGGCCAAAATGTTGCACGACAACTGTGGAGAAGAAACCGGAGCCGACCTGAGCTTGGTGGATAAAGTAAACGCTG

GAGCAGACTTGATGCCCGATGCCAAACTGAAATGCTACATCAAGTGCATCATGGAAACTGCGGGCATGTTATCAGACGGA

GAAGTCGATGTGGAGGCAGTGATAGCTCTCTTGCCCGAGGACATGGCCAAGAAAAACGGAGACGCCCTGCGAGCATGCGG

CACCCAGAAAGGAGCAGACGACTGTGACACGGCATTCCTCACCCAGGTATGCTGGCAGAAGGCAAACAAGGCAGAATACT

TCTTAATTTAG

>Cher_ABPX1b

ATGTCAGGACACACAGCACTCTGCTGTGCCATGGTAGCAGCATTTGTGCTGGGAGCCCACTGCATGGACGATGAGATGGC

CGAGCTGGCCAAAATGTTGCACGACAACTGTGGAGAGGAAACCGGGGCCGACCTGAGCTTGGTGGATAAAATTAATGCCG

GAGCAGACTTGATGCCCGACCCCAAACTGAAATGCTACATCAAGTGCATCATGGAAACCGCAGGCATGTTGTCGGAGGGA

GAAGTTGATGTGGAGGCAGTCATAGCTCTCTTACCTGAGGACATGGCCAAGAAAAACGGAGGTGCCATACGAGCATGCGG

CACCCAGAAAGGAGCAGACGACTGTGACACCGCATTCCTCACCCAGGTATGCTGGCAGAAGGCAAACAAGGCAGAATACT

TCTTAATTTAG

>Cher_ABPX2a

ATGTCTAAATTCACCGAGTACGCGTTTGCCACAGCAGTTCTGCTGTCTCTGGTACTTTCGCTGGCAGAGTCTGCGAGTAC

CAAGGAATCAGAAAACGCTAAGGATGATGTCAAAGCTAACATCGAAGACACGACTCCTGCGCCGGCTGATCGGGACGAAA

TGGACATCATGAGTGCTATGATGGACTGCAACGAGACCTTCAGGATTGAAATGTCATATTTGCTGGCGCTGAATGAAAGC

GGCAGCTTCCCTGACGAAACTGACAGAACACCAAAGTGCTACATTCGCTGCGTACTGGAGATGGTAGAGATAGCGTCGGC

CGACGGGCAGTTCGACGCCGCGCGCGCCGAGCCGGCGCTGAGCAGCATCCGCGGCGTGCGCCTGCTCAGCGACGTCGCCG

CGACCGCCGCCACGTGCGCTGCTGATCGACAGGAAGCATGCAAATGCGAGAGATCTTACCAATTCATCAAGTGCCTAATG

GAAATGGAAATCAAGATGGCTGAGAAATCTTAA

>Cher_ABPX2b

TCGCTGGCAGAGTCTGCGAGTACCAAGGAATCAGAAAACGCTAAGGATGATGTCAAAGCTAACATCGAAGACACGACTCC

TGCGCCGGCTGATCGGGACGAAATGGACATCATGAGTGCTATGATGGACTGCAACGAAACCTTCAGGATAGAGATGTCAT

ATTTGCTGGCGTTAAATGAAAGTGGCAGCTTCCCTGACGAAACCGACAGAACACCAAAGTGCTACATCCGCTGCGTCCTA

GAGATGGTGGAGATAGCGTCTGCCGACGGGCAGTTCGACGCCGCGCGCGCCGAGCCGGCGCTGAGCAGCATCCGC

>Cher_ABPX3a

ATGGGACGAAAGGACTGGCTCTTTCTGGTGGTGGTTGTGGTATCAGTTTGCGGGATAAATGCTTTGACGAGACCACAGTT

GAAAAAGTCGATGCAAATGTTAAAAAAAAAATGTTTGACAAAGATAGATGTCACAGAAGATATGGTCGTTGATATAGACA

AAGGAAAGTTTATCGAAGAAACTAATTTCATGTGTTACATCGCATGTGTTTACCAGATGGGGCAAGTTGTAAAAAATAAC

AAATTGAGTTACGAGGCATCTATGAAGCAGGTGGACTTGATGTACCCTGTAGAGATGAGGGAGGCTGTAAAGAAAGCCAT

AGATAGCTGTAAAGACGTATCCAAAAAGTACAAAGATCTGTGTGAGGCCTCATACTGGACAGCAAAATGTATTTACGAAG

CCGATCCGAAGAACTTTGTATTTGCATAG

>Cher_ABPX3b

ATGGGGCGAAAGGACTGGGTTTTTCTGGTGGTGGTAGTGGTTTCAGTCTGTGGGATTAATGCTCTGACCAGACAACAATT

GAAAAAGTCTTCGCAAATGTTCAAAAAGAAATGTATGGCAAAGATAGATGTCACAGAAGATATGGTCGGAGATATAGACA

AAGGAAAGTTTATCGAAGAAAAGAATGTAATGTGTTACATCGCATGTGTGTATCAGATGGCACAAGTTATAAAAAATAAC

AAATTGAGCTATGAAGCATCTATGAAACAGGTCGACTTGATGTACCCTGCAGAGATGAGGGAGGCTGTGAAGAAAGCCAT

AGATAGCTGTAAAGACGTATCCAAGAAGTACAAAGACCTGTGCGAGGCTTCATACTGGACCGCAAAATGTATTTACGAAG

ACGATCCGAAGAACTTTGTATTCGCATAG

>Cher_ABPX4

CTTTTAAAGAAAACTTGCATGCCAAAGCATGATGTCACAGAAGAACAAGTAGGGAAGATAGACCAAGGTGTATTCTTGGA

AGAGAAGAATGTGAAGTGCTACATCTCTTGCATTTATTCGGCCGGAGGGGCAGTGAAAAACAATAAAATTGTGCACGAGG

CTATGCTCAAACAAGTGGACATGATGTTTCCCCCTGACATGAAGGACCCTGTCAAAGCGGCTATGGAGAACTGCAAAGGC

GTCGCCAAAAAATATAAAGACATCTGCGAAGCTTGCTTTTACACTGCTAAGTGTCTCTACGACACTGACCCAGCCAATTT

CATGTTCCCTTGA

>Cher_ABPX5

ATGACAGTCATGAAGAAGCAGTGTATACCGAAAGTAGGAGTGGCTGAAGACAAAGTGGCTAGAATTGAAGAAGGCGTATT

TCCAGAAGACCGTAACGTCATGTGCTACGTGGCCTGTATCTATAAAACTATCCAAGTGGTTAAGAATAACAGGCTGAAYA

AAGAAATGATATCRAAACAGATCGATATTATGTACCCTGCAGAGATGAAGGCGGCCGTCAAGAAATCTGTTGCGAAGTGC

GTTGACGTTCAGGATAACTACGAAGACGAGTGCGAACGYGTGTACTACGCTACCAAATGTTTATAYGAAGAYGACCCACC

GAATTTCATATTTCCATAA

>Cher_ABPX6

AAAAATTCCGGAAAGATGATGAAAAAATCGTGCATGCCGAAAAATGACGTGACTGAAGAGCAAGTTGGGGAAATAGAGCA

AGGGAAATTTATAGAAGAAAAAAATGTTATGTGCTACATAGCTTGTGTATACTCCATGACACAGGTGGTGAAAAACAATA

AGCTGAGCTACGAAGCCGTCGTCAAACAAGTGGACATGATGTTTCCTGCAGAAATGAGAGATGCTGTGAAGGCTGCGGCT

GAGAAATGCAAGGATGTCTCTAAGAAGTACAAAGACATATGTGAGGCGTCATACTGGACTGCAAAGTGCATGTATGACTC

TGATCCGAAGAATTTCGTTTTCCCTTAA

>Cher_ACAT1

ATGGCCGTAGCAATAAACAAAGGTATATTCATAGTCGCAGCCAAGCGCACGCCATTCGGCCGCTATGGGGGCAAGCTGCG

TGATATTGCCCCTTCAGACCTGCTGGCTACCGCTGCCAAAGATGCCTTCAAGGCCGGCGGAGTTGCGCCAGCGCTGGTTG

ATACCGTCAACATTGGACAAGTCAACGGGCTGAGCGGCTCATCAGACGGTGGCCTGGCCCCGCGCCATGCGTCCCTCAAG

TCCGGAGTACCACAGGAGAAGCCGGCCCTCGGAGTCAACCGGCTCTGCGGCTCTGGCTTCCAGGCCATCATCAACAGCGC

CCAAGACATCATCACAGGAGCCGCGAAAGTATCCCTCGCAGGAGGCACCGAGAACATGTCAGGCATGCCTTTCGTGGTGC

GCAACGTGCGCTTCGGAACAGCCCTTGGACAGAACATCGAGTTCGAAGACGCTCTGAACAAGGGTTCCCTGGACACGTAC

TGCAACTTCACGATGCCCCAGACCGCTGAGAACCTGGCTGAGAGGTACAAGCTGCAGAGGAGTGAGGTGGATGCCTTCGC

GCTGCAGTCGCAGCAGAGGTGGAAGGCTGCCTACGACGGCGGCTTCTTCAACGAGGAGATGTCCCCCGTGACAGTAAAGG

TCAAGAAGCAGGATGTTGTCGTGTCGATGGACGAGCACCCGCGCCCGGAGACCACGGCCGAGGGCTTGGCCAAGCTGCCT

GTTCTGTTCCGCAAGGGCGGAGTAGTCACTGCTGGGAACTCTTCTGGGGTAAACGACGGAGCTGGCGCTGTTATTCTCGC

TAGTGAAGAGGCGACGAGCCAGAACGATCTGAAGCCTATTGCCCGCCTGCTGGGATGGTCCTTTGTGGGCGTGGACCCCA

GCGTCATGGGAATTGGACCCGTGCCCGCTATCCAGAATCTGCTCGCCGTCAACAAGCTCAGCCTTAAGGATATTGATCTC

ATTGAGATCAACGAGGCTTTCGCAGCGCAGACCCTAGCGTGCGTGAAGGAGCTCGGCATTGACCAGAGCATCCTGAACGT

GAACGGAGGCGCCGTCGCACTGGGGCACCCTGTTGGGGCTTCAGGGGCTCGCATCACCGCGCACCTGGCCTATGAACTCA

GACGCCGTGGGCTGAAGAGGGGTATTGGATCGGCTTGCATCGGCGGAGGACAAGGCATTGCTCTGCTCATTGAGACTGTT

TAA

>Cher_ACAT2

GTTGGCGCGAAAAGGACACCATTTTGTGGCTATGGCGGGCCGCTGCGGGAGTTGCCAGCCTGCCATGTGTTCGCTGCCGC

GGCAAAGGACGCGATCCGTTCAGCCAACGTCGATCCCAGTACCATCGATAATACTGTTGTTGGAAATGTTAATTTTCTGA

GCCAATGCGATGGCGGAAAGACTGCAAGGTACTGCGGCATTTATTCCGAGGTTCCTATAGACAGGCCAGCTTTGGGCGTG

AGCAAAGCCTGTGGAACTGGCATTCAGGCCATCATCACGAGTGCTGTGGATATTTTAACAGGTGCAGCAAAAATATCCCT

AACAGGGGGTACAGAGAACATGTCAGCAATGCCACTTGTAGTTCGGAACGCTCGCTTCGGGACTGTTCTAGGGGGGTCGT

ATCACGTCGAAGACTATATCACGAAACAATACCTAGACTCATATAGCGGCCTAACACTGCAACAGATTGCAGAGGAAGTT

GCGAAGAAGTGTGGAATCACAAGGGAAGAGGTCGATGAATTTGCACTGCAAAGCCATTTGAAATGGAAG

>Cher_ACAT3

ATGGCATCCCACGTAGCGAAAACCTTGTTAAAAGTTCCCCATGCCAGCTCGACGGTCAAGTTTGACACAGCTCGACGAGC

CCTGAGCATCGGGGCAGCGCTACAGTCGAAGAAGACCCTGCAGGACCGCACGGGCAAGAACATCGTGCTCGTGGATGGAG

TCCGCACGCCATTCCTCGTCTCCTTCACCGACTACTCCAAGATGATGCCGCATGAGCTGGCGAGACACGCACTGCTGGGT

CTCCTCCAAAAGACAGGCATCGACAAGGACCTGATAGACTACATCATCTATGGCACGGTCATCCAGGAGGTGAAGACGTC

CAACATCGGCCGGGAGGCAGCGCTGGCTGCTGGGTTTAGTGATCGCACACCGTCCCATACTGTCACCATGGCTTGCATAT

CTTCCAACCAGGCTATTACTACTGGCATCGGCATGATAGCAGCCGGCGCATACGACATAATAGTGGCCGGCGGCGTCGAG

TTCATGTCGGACGTGCCGATCCGACACTCGCGCAAGATGCGCTCGCTGCTGCTGCGCGTGAACAGAGCCAAGACGCCCGC

GCAGCGGCTGTCGTTGCTCGCATCTATACGGCCCGACTTCTTCGCGCCTGAGCTGCCAGCAGTAGCCGAATTCTCCTCGG

GAGAAACTATGGGTCACAGCGCCGACCGACTCGCCGCCGCATTCGGTGCATCAAGACAAGAGCAGGACGATTACTCGCTC

CGCTCCCACAAGTGCGCTGCTGAGGCTCAGGCCAAGGGATACTTTACTGACCTCATCCCCGTCAAGGTTGACGGCAAAGA

CGCTCTGGTGGAGAAGGACAACGGCATCCGCGTGTCCACGCCCGAGCAGCTCGCCAAGCTGCGGCCCGCGTTCGTCAAGC

CGCACGGCACCGTCACGGCCGCCAACGCTTCGTTCTTGACGGACGGCGCATCTGCGTGCCTGGTGATGCCGGAAGCGAAG

GCGAAGGAGCTAGGCTTCAAGCCCAAAGCCTACCTGAGGGACTTCACTTACGTTGCTCAGGACCCCGTGGACCAGCTGCT

GCTGGGCCCGGCGTACGGCATCCCCAAGATCCTGGAGAAGGCCGGCCTCACCATGAAGGATGTCGACTCTTGGGAGATCC

ACGAGGCCTTCGCTGGCCAAATCTTGGCTAACTTGAAGGCTATGGACTCTGACTGGTTCGGCCAGAACTATCTCGGCAGG

CAAGGAAAGGTGGGTTCCCCGGACATGTCAAAGTGGAACAACTGGGGCGGCTCTCTCTCCATCGGACACCCGTTCGCGGC

CACTGGCGTCCGGCTGGCCATGCACACGGCGAACCGGCTGGTGCGCGAGGACGGGCAGATCGGCGTCATCAGCGCCTGCG

CCGCCGGCGGCCAGGGCGTCGCCATGATCCTGGAGAGACACCCTGATGCTAATGCTGAGTAA

>Cher_ACAT4

ATGTCTGTGGCATCTAAAGGTATATTCATCGTGGGCGCCAAGCGCACAGCCTTCGGCACGTTCGGCGGCGTGTTCCGTAA

CACGTCGGCCACGGAGCTGCAGACGCACGCCTTCAAGGCGGCGCTCACCTCGGCCGGCGTGGCGCCCGCGCAGGTCGACA

CCGTCGTCGTGGGGCAGGTCATGTCGGCATCTCAAACCGACGGCATCTTCACCCCCCGCCACGCCGCCCTGAAGGCCGGC

ATCCCGCAGGAGAAGCCAGTTCTCGGCATCAACAGACTCTGCGGCTCTGGCTTCCAGTCCATCGTGAACAGTGCACAGGA

CATCCTAACAGGCGCAGCACAAATCTCCGTAGCAGGGGGCGTAGAGAACATGTCACAAGCCCCCTTCGCAGTCCGCAACG

TTCGCTTCGGTACCGTGTTAGGCCAAACCTTCGCTTTCGAGGACACCCTGTGGGCAGGCCTTACTGACTCATACTGTGGG

ATGCCGATGGGCATGACTGCTGAGAAACTGGGGGCACAGTATAAGGTTACGAGGGATGAGGTGGATAATTTTGCCCTGCA

GTCGCAGCAGCGGTGGAAGACTTCCAACGACGCTGGCGTGTTCAAAGCCGAGATCGAACCCGTGACCATCACAGTCAAGC

GCAAGGAGGTGTCTGTCACCACCGACGAGCACCCGCGCCCCCAGACAACACTAGAGGGATTGAAGAAACTAGCCCCCGTG

TTCAAGAAAGAGGGGCTGGTCACCGCTGGTTCCGCTTCTGGTATCAGCGACGGCGCCGGCGCCCTAGTGCTCGCCAGCGA

AGAAGCCGCCAAAAACCTCAAACCCCTGGCGCGCCTCGTGGGTTGGTCCTACGTCGGGGTTGACCCCAGCATTATGGGGG

TCGGCCCCGTGCCCGCCATACAGAACCTGCTCAAGGTTACTGGGTTCACGCTCAACGATATCGACCTGATTGAGATCAAC

GAAGCCTTCGTAGCGCAGACCCTGTCCTGCGCGAAAGCCCTAAACCTGGACATGTCCAAACTGAACGTGAACGGCGGCGC

CACCGCGCTCGGGCACCCGCTCGGCGCTTCCGGGTCGCGCATCACCGCGCACCTCGTGCACGAACTCAGACGCAAAGGCC

TCAAGAGGGCGATCGGATCCGCCTGCATCGGCGGCGGACAAGGCATCGCCGTCATGGTCGAATCTGTTTAA

>Cher_ACBP1

ATGTCTCTCCAAGAGCAATTCAAGTCCGTGAGCGATTCCGTGAGGAACTGGAAGACCAAGCCCGCTGACAGCGAGAACCT

GGCTCTGTACTCCCTGTACAAGCAGGCCATCGCCGGCGACGTCAACATCCCCGAGCCCTCCGGTCTGGTCGAGAACGCCA

AGTGGAAGGCATGGACCAGCCGCAAGGGAATCTCTGCTGACGACGCCAAGAAGCAGTACATCGACCTCGCCGGCCAGCTC

CAGGGCAAATACGCGTAA

>Cher_ACBP2

ATGTCGCTCAAGGAAAAGTTTGATGCCGCTGTGAATGTTATAAGGAGCCTTCCTAAGAGTGGGTCATACCAGCCCAGCAA

TGAGCTGATGCTGCGTTTCTACAGCTACTTCAAGCAGGCGACCGAGGGGCCTTGCGACAAGCCCAAACCAGGCTTCTGGG

ACGTCGTCAACAGAGCAAAATGGGAGTCGTGGAACAAGCTAGGCAATATGACAGAGGATGAGGCGATGCAGGCGTATGTC

GATGAGCTACACAAGATAGTAGAGACGATGTCCTACAGCGCTGACGTGGCGTCGTTCCTCTCAGTGGATGACGAGGGCGA

GGAGTTCCCCAGCCGCGACCTGGAGCTGGTGGCGGGCGACGTGCTCAAGCGCGCGCGCAGCGAGCACAACAGCCCCTCAG

GGTCGCGCTCGGCGAGCGGCGCG

>Cher_ACBP3

ATGGCCGAGGCACTTAGTTATCCCGATTCAGACTTCTCTGACGAGGACCAGTCACCTCTAGACATATCTTTCAACAAGGC

TGCTGATCATGTGAGGAAACTAACCTCCAAACTTGGTAACAACCAGCTGTTAGAACTCTACGGATTATACAAGCAGGGCT

TAGAAGGCGTATGTAATGTGCCCAAGCCAGGTTGGCTAGATGGTAAGGGTCGTAGAAAATGGGAAGCGTGGAGAGCTCTC

GGAGACATGCCTTCGATCGAAGCTAAGGAAAAATACATAGCGCTAGTCCAGAAATACGACCCGGAATGTTCAGACTTAGT

TGACATAAATACCAAAGAGGCCTGGGTGACCGTGTCATCCCTCCGATACTCTCCCGAGCCTGAGCTCATCCACAACGAGT

TGTCTCTACTAGACGCATCACGAGAAGACTGTGGGGAACGCGTCATGGAGCTGCTGTCACAAAACCCTGAGCTTAAACAC

GAGAGGAGCGAGGATGGTCTGACTGCGCTACACTGGGCGGCTGACCGAGACGCCACAAAAGCTTTAGCAGCTGCGTTAAA

AGGAGGTTGCTTCATTGATGCAGTTGATGACAATGGTCAAACCGCATTACATTATGCTGCGTTCTGTGGCCATCTCAATT

CCACAATCATCTTGGTGGAGGCAGGAGCTACTTTAGTGAAGGATAGCGACGACTGCACTCCTCTGGACTTGGCGACTGAC

GATGAAGTGAAGAAGGTTCTCCAAGGTGCTAAGTCTTAA

>Cher_ACC

ATGACTCCGCCCGTTTTCGGATCGTTTGCCACAATTGCCAACTTTGTTTACAACCTATTCACAATGATGAAACGACGGAC

CTCCAAGCGCTTCGTCCTCGGGGATAATGAGGAACAGCCATCCTGGGATATGGATGACGAGGCTACAGCGGTGTTGCCAG

ACCTTCAGAAGTTCCAGATGACCTTAGAGGCGGAGCCGGAGAGTGAGAAGGAAGAGAAGAAGGAGCAGAGCACTAGTAAA

GGGCTGCTGCGGCCGCCTAATCAGACTTTGCAACCGTCAATGTCCCAGGGCACCGTCATCCACAGTCAGCGGTTTCAAGA

GAAGAACTTCACCGTTGCCACTCCCGAGGAGTTCGTTAAACGGTTCCAGGGGACTCGGCCCATTAATAAGGTCCTAATAG

CGAACAACGGTATCGGCGCTGTAAAATGCATGCGCTCCATCCGAAGATGGTCCTACGAGATGTTCAAGAACGAGCGCGCC

GTACGGTTCGTCGTCATGGTGACCCCAGAGGACCTGAAGGCGAACGCGGAGTACATAAAGATGGCGGACCACTACGTGCC

GGTGCCGGGCGGCAGCAACAACAACAATTACGCCAACGTCGAGCTCATCGTCGACATCGCCATCCGGACGCAAGTCCAGG

CGGTATGGGCCGGCTGGGGCCACGCGTCAGAGAACCCGAAGCTACCGGAGCTGCTCCACCGAGCCGGCGTGGTCTTCATA

GGGCCCCCGGAAAAGGCAATGTGGGCCCTCGGGGACAAGATCGCTTCGTCCATCGTGGCGCAGACCGCTGACATACCCAC

GCTGCCTTGGAGCGGGAGCGAACTGAAAGCCGAATACAACAGCAAGAAGATCAAAATATCCTCTGAACTGTTCGCCAAAG

GCTGCGTCACCACGCCCGAGCAGGGCCTCCAGTCTGCCTACAAGATCGGGTTCCCTGTGATGATCAAGGCGTCAGAAGGC

GGCGGCGGCAAGGGTATCAGGAAAGTTGACAACCCTGATGACTTTCCCAACATGTTTAGACAGGTCCAAGCCGAGGTGCC

TGGATCCCCGATTTTCGTGATGAAGCTCGCCAAATCAGCCCGGCATTTGGAGGTGCAGCTGATTGCCGATCAGTATGGTA

ACGCCATCTCTCTCTTCGGGCGCGACTGCTCCATCCAGCGTCGTCATCAGAAGATCATTGAGGAGGCCCCCGCCGCCATC

GCTAAGCCTGACGTTTTTATTGAGATGGAAAAGTCGGCGGTCCGCCTAGCAAAAATGGTCGGCTACGTGTCAGCAGGCAC

AGTAGAGTATCTCTACGAGCCGGGAACAGGGCAGTACTACTTCCTGGAGCTGAACCCGCGGCTGCAAGTAGAGCATCCTT

GCACGGAGATGGTCGCTGATGTCAANNNNNNNNNNGCGCAGCTGCAGATAGCAATGGGTCTCCCCCTATACAACATCAAA

GACATCCGCCTTCTCTACGGGGAGTCCCCATGGGGTCTCTCCGAGATAGAGCTCGACGACCCCAAGCAGCGCCCGTCCCC

CTGGGGTCATGTTATCGCCGCTAGAATCACGAGCGAGAACCCCGACGAAGGTTTCAAACCCTCATCAGGCACTGTCCAGG

AGCTAAACTTCCGCTCCTCAAAGAACGTATGGGGTTACTTCAGCGTGGCGGCTTCCGGAGGCCTGCACGAGTTCGCAGAT

TCCCAGTTCGGGCATTGCTTCTCGTGGGGCGAGACGCGCGAGCAGGCGCGCGAGAACCTAGTAATAGCTCTAAAAGAGCT

CAGTATTCGTGGCGATTTCCGTACCACAGTCGAATACCTGATCACTCTGCTGGAAACCAGCGCTTTCCAGAATAACGACA

TCGACACCAGCTGGCTCGACGCCCTTATTGCTGAGAGGATGCAATCAGAAAAGCCAGACATAATGCTGGGCGTACTCTGT

GGCTCTATCCTTATAGCGGACAGCCTTATCACTACCCACTTCCAAGACTTCAAGAGTGGGTTGGAGAAAGGCCAGATCCA

AGGTTCAAGCCAGCTCTCAAACTGCGTCGAAGTGGAACTGATACACACCGGAAGCAAATACCGAGTGCAAGCCTGCAAAT

CTGGCACCACTACCTACTTCCTGGCAATGAACGGGAGTTTCAAGGAGTTGGAGGTCCATAAGTTGACTGATGGCGGCACC

CTCCTCTCAGTGGACGGCGCTTCCTACACAACCTACCTTAAAGAAGAGGTGGACAAATACCGCGTAGTCATCGGAAACCA

AACAGTTGTCTTCGAGAAGGAGAAAGACCCTTCGAAACTGAGAGCGCCATCTGCTGGCAAACTGATCAATACTCTGATAG

AGGACGGCGGTCACGTTGATAAGGGACAGCCTTACGCTGAGATTGAGGTGATGAAAATGGTGATGACATTATCAGCTCCG

GAATCCGGGAATGTGACCTTTAGTCTAAGACCGGGCGCGGTTCTGGAGAGCGGCTCCCTTATTGGTATCCTTGAGCTCGA

CGACCCATCCTTAGTGACAACAGCGCAACTATACAAGGGACAATTCCCTACTGCAGATAACCCCCATCTGTCGGAGAAAC

TCAGTCAACAACATACCAAATATAGGGCTATACTTGAGAACGTTCTAGCAGGTTACTGCCTCCCTGAGCCCTACAATACA

CCTCGCTCCAGAGATATCGTAGAGAAATTCATGCAGAGTCTACGCGACCCTTCGCTGCCTCTACTGGAGTTGCAGGAGGT

ACTATCATCAGCCTCTGGCCGTATACCAGTGGCCGTAGAGAAGAAAGTCCGGAAACTGATGGCTCTGTACGAGAGAAACA

TCACCAGTGTGTTAGCACAGTTCCCGAGTCAGCAGATCGCCAGCGTTATTGACCACCACGCCGCGTCGCTGCCTAAGAGA

GCCGACCGAGACGTATTCTTCATGAGCACGCAAGCTCTAGTCGTATTAGTTCAGAGATACAGGAATGGAATTCGTGGTAG

AATGAAGGCGGCCGTACACGATTTGCTCAGGCAGTACTATCAAGTTGAGAGCAACTTCCAACTGGGGTCCTATGACAAAT

GCGTGATGGCTCTTCGGGATAAACATAAGGATGACATGCAAGCAGTCGCTGACATCATATTCTCGCACAACCAAGTCGCC

AAGAAGAATCTATTGGTGACCCTGTTAATTGACCACCTCTGGTCCAACGAGCCCGGTTTGACAGACGAGCTGGCGGCCAT

TTTGAATGAGCTGACGTCACTGCATCGCGCGGAGCACAGCCGCGTCGCTCTTAGAGCGAGACAAGTACTAATAGCAGCCC

ACCAACCAGCGTACGAGTTGCGTCACAACCAAATGGAGTCAATATTCCTATCAGCCGTCGACATGTATGGCCACGATTTC

CATCCGGAAAACCTGCAGAAACTGATACTGTCAGAGACTTCTATCTTTGACATACTGCACGATTTCTTCTACCACACTAA

TGCTGCGGTATGCAACGCGTCTCTTGAAGTGTACGTGCGTCGCGCCTACACTTCGTACGAGATCCAATGTCTCCAGCATC

TGGCCTTGTCTGGCGAATTGGGCGTGGTTCATTTCCAGTTTGTACTGCCGACTGGACATCCTAACAGGATCCCCCTCAGC

CAATCAGAGATAGAGCTCGCCTCAGCGGAAGACGCAGAAGGCATACCAGCGGAACTGTGCACAGCGGCGATGAGCAAATG

CCACCACCGCACCGGGGCGTTAGCCGCGTTTGACTCCTTCGATCAGTTCGCTCAGTACGCTGACGAGTTGCTGGATCTGG

TGCATGACTTCGCTAGCACCGCTTCTGTGAGGAGAGAGGACCTCCAAGCCTTACAGGACGGCAGCGAAGGCCGCGATAGC

ACCAGTATCAACGTTGGCATGGACTTCAAGCCGGTGGACGCTGAAGAAGCGGACCTGGAGCCAATCCACATCCTGATGAT

CGGCGTGCGTGACAACGGGGAATCGGACGACGTCGCTCTAGCTCGTCGTTTTGGTGGCTTCTGCCGCGCGCATCGCCACG

AGCTGCATCAGAAAAGGGTCCGAAGAGTCACTTTTATGTCGCTGATCAAACGTCAGTTCCCGAAATTCTTCACCTACCGC

GCCCGCAACGATTTCACAGAAGACACAATTTACCGCCATCTTGAACCCGCATCAGCCTTCCAACTAGAACTGTATAGAAT

GAGGAGCTACGAGCTAGAAGCTTTACCCACCAGCAACCAGAAGATGCATCTGTACCTAGGAAAGGCTAAGGTCAAGAAGG

GTCAAGAGGTGACCGACTTCCGTTTCTTCATCCGCTCCATCATCCGGCATCAGGACCTCATCACGAAGGAGGCCAGTTTC

GAGTATCTGCAGAACGAAGGAGAAAGGGTGTTGCTGGAAGCGATGGACGAGCTGGAAGTGGCGTTCTCGCACCCGCTGGC

CAAGAGGACGGACTGCAACCACATATTCCTCAACTTTGGACCCACCGTCATCATGGATCCTTCTAAGATCGAAGAGTCTG

TTCTTGGTATGGTGATGCGCTACGGACCTAGGCTGTGGAAACTGAGAGTGCTCCAAGCTGAGATCAGATTCACACTGCGA

ATTGGACCGGGAGCGCCCACTAAGAACGTGCGTCTGTGCCTCTCCAACGGCTCAGGTTATACCCTAGACGTCTACACCTA

CGAGGAGGTTTCCGATCCCAAGATTGGCGTTATAATGTTCCAATCATTCGGCACGCGCCAAGGCCCGATGCACGGCCTGC

CTATCTCCACGCCCTACGTTACCAAGGATTATCTTCAGCAGAAGAGGTTTTTGGCAACGTCGCAGGGCACAACTTACGTG

TATGACATGCCCGACATGTTCAGACAGATGGTTGAAAAGAGATGGCGCGAGTGCATCGAGGATGGCAGCGTTGAAGGTCC

AGCACCGGATAACGTGATGTCTGCAGTCGAGCTGGTGGTGGAAGCAGAAGGTGAACGCAGGGTGGTCGAGGTCACGAGGC

TACCTGGACAGAATACTGTCGGCATGGTAGCATGGCGTCTAACCCTCTTCACGCCGGAATGCCCTAAAGGCCGAGACATA

GTGGTCATAGCGAACGACTTAACATACTATATGGGGTCTTTCGGACCCCAAGAAGACTGGGTGTACTATAGGGCCTCCCA

GTACGCCAGGGAACGTAAGATTCCCAGGATCTACATGAGCGTAAACTCAGGCGCCCGAATCGGCGTAGCGGAAGAAGTGA

AATCAGCGTTCAACGTAGCCTGGATAGAAGCGGAAAGGCCGGAGCGCGGCTTCAAATATCTCTACCTCACGCCGGAGGGC

TACAGCAAACTGGGCCCGCTGGGCGCAGTCAAGACGCAGCTGATCGAGGACGAGGGCGAGTCGCGGTACAAGATCACTGA

CATCATCGGCAAAGAGGACGGCTTGGGCGTGGAGTGCCTGCGCGACGCAGGCCTCATAGCGGGTGAGACCGCACAAGCGT

ACGAAGACATCGTCACTATATCCATTGTGACGTGCCGCGCTATTGGCATCGGCGCTTATGTCGTCAGACTCGGCCACCGC

GTTATTCAAGTGGAGAATTCCTACATAATCCTAACCGGCTACGCGGCCCTGAACAAAGTGCTCGGGCGCGCTGTGTACGC

CAGCAACAACCAGCTCGGCGGCATTCAGATCATGCACAACAACGGTGTGACCAGCGCTGTCGCTGCTACTGACTTAGACG

CTGTCGGTTCGGCCGTACGGTGGCTCAGTTACGTACCCAAGGATAAACTCTCAATGGTGCCTATAATGCGAGTCTCAGAC

CCCATCGACAGGCCAGTGGAATGGAAGCCTCCGCGCTCCGCGCACGACCCGCGCTTAATGTTGACCGGTGACGGCGCCCG

GGGCGGCTTTTTCGACGCCGGAACGTTTGATGAAGTCATGCGTCCGTGGGCGCAGACTGTTATAGCTGGCCGCGCCCGCC

TGGGCGGTATACCAGTGGGCGTGATCGCAGTGGAGACGAGAACAGTGGAACTGACGCTGCCCGCTGACCCTGCTAACTTG

GACTCCGAATCCAAGACTTTACAGCAAGCTGGGCAGGTTTGGTTTCCGGACTCAGCCTACAAGACGTCTCAGGCTATCAA

CGACTTCTCCCGCGAGGGGCTGCCCATCATGATATTCGCGAACTGGCGCGGTTTCAGCGGCGGACAAAAGGACATGTACG

AGGAAATACTCAAGTTCGGTGCCGAGATCGTCCGCGCCCTCCGCCGCGCGACCGCCCCCGTACTCGTCTACATCCCGCCG

GGCGCCGAGCTGCGGGGCGGCGCCTGGGCGGTCGTCGACCCGAGTGTCAACAGCGCGAGGATGGAGATGTATGCCGACCC

TGAAGCCAGAGGCGGCGTGTTAGAGCCAGAAGGAATAGTTGAAGTGAAATTCAAGGAACGGGACACCGTCAAAGCGATGC

AGAGACTCGACCCGCAGTTACAACGTCTCATCGCTAGGATTGCTGAACTTAAAGAGCAAATAGCAGAGCTGACGAAAAAC

TTCGACCGCCGCGGCTCCATAGACGACGTACTAATAAAGACCGATGCGGGAAGAGAGGCGGAGAGCAGAGCGAGGGAGAT

GGAGACAGAGCTGCTGCAATGCGAGAGGAGCGCCAAGGCGAGGGAGAAGGAGCTACAACCCATATACCACGAGATCGCAG

TACAATTCGCAGAGCTTCACGACACGGCTGAGAGAATGCTTGAAAAGGGCTGCATATTTGACATAATCCCCTGGCGCGAC

TCCCGTCGCTTCCTCTACTGGCGTCTTCGCCGTCTGATGCTACAGAACGAGCAAGAGAGACGCGTACAGAGCGCGGTTCA

AACGGCAGATAGTATGGACCACGGGGCAGCCTCCGCTACTCTCAGACGGTGGTTCACTGAAGACCGTGGGGAGACGCAGT

CGCACCAATGGGAGCACGACAACGAGGCCGTCTGCCATTGGCTGGAAGCGCAGGTTTTGGACGACAATTCCGTGCTGGAG

CGAAATCTGCGAGCGATCAAACAAGACGCCGTCATGCAGGCTGTCAATGGACTTGTTATGGATCTGACGCCATCACAACG

GGCCGAA

>Cher_ACOT1

ATGGAACCGAATCCGGTAATTATTGCAGCGACTGCAAAACAAACAGCATCCCTAATATTTTTGCATGGGCTCGGTGATAC

TGGCCACGGATGGGCGAGCACGATCGCTGCTATAAGAGGGCCTCACGTAAAAGTGATCTGCCCGACGGCCGCGACGATCC

CCGTGACGCTGAACGCGGGGTTCCGGATGCCGTCCTGGTTCGATCTGCGGACCCTGGACGCGACTGCTCCCGAGGATGAG

GAGGGCATCATGAGAGCCACTGGCCTGATCCATGGCCTGATTTCAGATGAGATTAAAGCTGGCATCCCAGCTAACCGAGT

TCTGCTTGGCGGTTTCTCCCAAGGAGGAGCACTGGCCCTGCATTGCGCCCTCACATACACTCAGCCACTTGCTGGTGTCA

TCTCCCTGTCGTGCTGGCTGCCGAGACATGCTCACTTCCCTGATGGAGTAAAAGCACCTTTGGATATGCCGATATTCCAA

GCCCACGGAGATTGCGATCCCGTGGTGCCCTTCAAGTGGGGTCAAATGACTGCATCCTTCCTCAAGACATTCATGAAAAA

TATTGAGTTCACCACATATCAAGGACTAACACACAGCTCCTCGGAGGCAGAGCTTAAAGACATGCGGGCATTCATCGAAA

GGACGATACCTGCCGCCAAATGA

>Cher_ACOT2

ATGAGGCTATTAGTTTGTTTAGTGTTTTTAATCAAAACAATAATTGACCCTGCCGATGCGACACCGACTCCGATAGTATT

ATGGCATGGAATGGGTGACACATGCTGTACTGCATACAGCCTAGGTGTGTTTAAACAGTTCTTGGAGAAACAAATACCTG

GAGTATATGTGAATTCCGTAAGGATCGGCAACAACACTATTGAAGACTTTGAAAATGGTTTCTTCATGAACCCTAATACG

CAAATAGAATCAGTGTGCAAACAACTTGCTGTGGACCCTAAATTGCAGAACGGATTTAATGCCATTGGATTTTCTCAAGG

GGGTCAATTTCTACGAGGTGTGATTGAGCGGTGCGGTCACAAATTGCCACCGGTCAAAAACTTCATCACCCTCGGAGGCC

AGCATCAAGGAGTGTATGGGCTCCCGCATTGCATGGCCCTTGCTCACAAGACTTGCAACTATATCAGGGAGTTGCTGAAC

TATGCAGCATATCATAATTGGGTGCAAAAAGCTCTAGTCCAAGCGACATACTGGCACGACCCACTCCACGAAAACGACTA

CAAGCGCGGCAGCATCCTCATGGCAGACATCAACAACGAGTTAAACGTCAACCAGACATACAAAGACAACCTGAGCAAGC

TAGATCACTTCATCATGGTCAAGTTTGATAACGACTCTATGGTGCAGCCGAGGGAGTCTGCTTGGTTTGGCTTCTATGCG

CCAGGACAGGGGAAGGTGCTGCTGACGTTGCAACAGTCGCAGATCTATTTGGAGGACCGCCTCGGTTTGCAGAAGATGGA

CAAGGCCGGGAAACTCGTGTTTCTCTCCCTCCCCGGCGACCATCTGCATTTCACCGTCGACTGGTTCGTCGCTAACATCA

TTAAACCTTATCTGCTTAATTAA

>Cher_ACOX1a

ATGACGGATTCCGGTAAAGTTAATCCTGATCTCCAAAGAGAAAGGGACAATTGCACTTTTGATGTTACGGAATTGACAAA

TTTAATTGATGGGGGTGTCCAGAAAACAGAAGAGAGAAGGAAACGAGAGGAAATGGTGCTAAAGGAAGGCATCCACATAG

AAGAGGTGCCGTCCGTGTACCTCAGCCACAAGGACAAGTACGAGCTGGCCGTCAAGAAGGCCTGCTACCTGTTCAAGATG

ATCCGGAGGCTCCAGGAGGAAGAGAACACGGGGATGGAGAACTATCAAGAGGTGCTAGGCGGCTCCCTCGGCTCAGCCAT

ACTTGGGGACGGTTCCCCGCTAACTCTCCACTATGTGATGTTCATCCCCACTATTATGGGACAGGCGTCTGTGGAGCAGC

AAGTGCATTGGCTCGGGAGGGCCTTCAACTGTGATATTATTGGGACTTATGCACAGACGGAGCTAGGTCACGGCACGTTC

ATCCGTGGCCTCGAGACGACAGCTACATATGACCCCTCCACCAAGGAGTTTGTCTTACACAGTCCCACACTCACGTCGTA

CAAGTGGTGGCCCGGCGGACTGGCTCACACAGCAAACTACTGCATAGTAGTCGCCCAGCTCTACACCAATGGCAAATGCC

ACGGGATCCACCCATTCATAGTACAACTACGCGACGAAGAAACCCACATGCCACTCCCTGGCATCAAGATTGGCGAGATT

GGCGCCAAGCTTGGCATGAATGGCACCAACAATGGCTTCCTTGGCTTTGATAAAGTGAGGATACCGAGGGAGTATATGCT

GATGAAGAACGCTAAGGTTTTAGAGGACGGTACATACATGAACGCGCCAAGCTCTAAGCTGGCGTACGGCACCATGATGT

TCGTACGCGTTGTGCTGGTCAATGACATGTGTTGTTACATGGCTAAAGCGGTCACCATCGCCACCCGGTACTCGGCTGTA

AGGCATCAGTCGCAGCCCAAACCCAACGAACCAGAGCCTCAGATCCTAGAATACGTAACTCAACAGCACAAACTAATCAT

CGGTATTGCGTCAGTACACGCGCTGCGTCTCAGCGGCACCTGGATGTGGAATATGTACAACAATGTTACCGCTGAGCTGG

AGAGCGGCGACCTGGACCGACTGCCCGAGCTGCACGCGCTCTCATGCTGCCTAAAGGCAGTGAGCACAGCCGACGCGGCC

GAGTGTGTCGAGCGCTGCCGGCTCTCGTGCGGCGGCCACGGCTACATGCTGGCGTCGAGCCTGCCGCTCATGTACGGGAT

GGTGACCGCCGCCTGCACGTACGAGGGCGAGAACACTGTGCTGCACCTGCAGACTGCCAGATACCTAGTAAAGGCGTGGC

AGCAGGCGTTGGGTGGTGACGTTCTCACCCCGACCGTCGCGTACATCGGTAAAGTAAGCAACGGCCGTCGCTCGCCGCCC

TGGGAGAACACCATCCAGGGAATAGTGCTCGGGTTCCAGAGAGTCGCTGCCGGTAAAATCGCTCAGTGCGTGGCGAACAT

CGAAAAGCGACAGAAGGGCGGTATGTCCTACGAAGACGCCTGGAACATGACATCCGTGCAGCTGACCGCCGCTTCTGAGG

CCCACTGTCGCGCCTTCATCTTATCCACATACTTCGAGGAAACGGAGAATCTGGTGAAGAATGTTTCTCCAGCGCTGCGC

ACAGTGCTTCTCCAACTAGTGGACCTGTATGTCGTGTTTTGGTCGCTACAGCGCGTCGGCGACCTGCTGAGGTTCACGTC

GATATCGGAGCGGGATATAGAGCAACTCCAGAGCTGGTACGAAGACTTGCTTCTCAAGCTGCGCGTTAACGCCGTGGGAT

TGGTCGACGCGTTCGACTTGAAAGACGAGATCCTAAACTCAGCCCTCGGCGCGTACGACGGTCGCGTGTACGAGCGGCTG

ATGGACGAGGCAATGAAGAGTCCTCTTAACGCAGAACCTGTCAATGACAGCTTCCACAAGTACCTCAAACCCCTCATGCA

GGGGAAACTGGTGCCTCACAAACTGTAA

>Cher_ACOX1b

ATGTGTGAAGTCAACACAGATTTGCAGAAGGAGCGGGACAAATGCACGTTCGATGTCAAGGCTCTGACGACTTTTATAGA

TGGAGGGGCCGACCGGACTTCGGAGCGTAAATATCGAGAAAAGATAATGTTAAGTGAGAAGGAGCTGACGGAGGGGGTTC

CTGAAGAGTACCTCAGCCACAAGGAGCGATACGAGAATGGTATCAAAAAGTCCGTTATACTTTTCAAACTGGTTCGTCAA

CTACAAGAGGAAGGCCGCTCGAATATCACTAACTACAGGGAAGTGATGAACGGGCTGCTGGGGACATCAAACACGCGTGA

CGGCTCCCCGCTCGGGCTCCACTACATCATGTTCATGCCGGCCATCATCAACCAGGGCAGCGAGGAGCAGCAGGCCCAAT

GGTTACCCAGAGCATGGAACTGCAGCATTCTTGGCAGTTACGCACAGACGGAGCTTGGCCATGGGACCTTCATCCGAGGG

CTAGAGACAACGGCGACGTACGACCCTGAGACCAAAGAGTTCGAGCTACACAGCCCCACTTTAACTTCCTACAAATGGTG

GCCCGGTGGATTGGGCCAAACATGCAACTACTGCATAGTGGTTGCCCAGCTATACACCAAAGGCCAATGCCACGGCATCC

ACTCCTTCATCATCCAAGTTAGAGACGAGGAGACGCACATGCCCCTCCCCGGGATCAAAGTGGGCGACATCGGGGCCAAA

CTCGGGCTCAACTCGGTCAATAATGGCTTCCTTGGGTTTGAGCATTTAAGGATACCGCGGGAGCAGATGCTGATGAAGCA

CGCTCAGGTTTTGGAGGACGGTACCTACGTCAAATCGAAGAGCAGCAAGCTCAACTACGGCGCCATGGTATTCGTGCGTG

TCGTCATCGTGTACGACATGGTGAACTATCTGTCAAGGGCTGTGACCATCGCCACGCGCTACTCCGCCGTGAGGAGACAA

AGCCAGCTCAGACCGGGAGAACCCGAGCCCCAAATCATCGACTATGTGACGCAGCAGCACAAGATCTTCATCGGTATTGC

GGCGTGCCACGCGCTGCGGCTGACTGCCAACATGCTGTGGGAGACCTTCCACGATGTGTCGGAGCAGCTGGTCGGCGGCA

ACCTGCAACGGTTACCGGAGTTGCACGCGCTAGCCTGCTGCCTTAAAGCTGTGAGCACGGCAGACACCGCCATGTATGTA

GAGCGCTGCCGGCTAGCGTGCGGCGGCCACGGGTACATGCACTCTTCGAACTTGCCAATGACGTATGGCCTGGTCACAGC

CGCTTGCACCTATGAGGGAGAGAATACTGTGCTGCTGCTGCAGACTGCTAGATACCTGGTAAAGACCTGGCAGGCGATAG

ACAGCACAGATATGGCCCAGATTCCGACGGTAGCTTACATGAAGGCAGCCAGCGCCGCCGGCTTCTGCGCTCGCTGGGAG

AACTCCGTCGAAGGCGTCATCAGAGGCCTGCAGAAGGTCGCTATGGGAAAGGTGTCTACGTGA

>Cher_ACOX1c

ATGGGTGTAAATAAAAAAGTAAACGAAGACTTGCAGAAGGAGCGGGACAAATGCAATTTTAATGTCGCTGAGCTGACACG

GTTCCTTGATGGAAGCGACAAGCTTACGAAGAAAAGGAAAGAGATCGAGAACCGAGTCCTCAGCGTGGAAGGTCTCATAG

ACGCGGTGCCTGATGAGTACATGAGCCACAAGGAGCGGTACGAGAATGCTGTACGGAAGTCCGTCATCATGCATCAGACC

TTGGGAAACCTGGAAGACAAGACCCTGTCCGTGGTTGAGAGCGCCAGGTTTGCGCTCCGCTCCACCATAACCTCAGCCAT

ATTCAAGGACAACTCGCCTCTGATGCTGCACTACTCCATGTTCACCAACGTGGTCGAAGGTCAGGGCGACGAGGAGCAGA

AGAAATACTGGTTACCGAAGATAAAGAATATGGAAATTATTGGCACTTACGCTCAGACGGAGTTGGGCCACGGCACGTTC

ATCCGTGGTCTGGAGACCATTGCCGAATACGACCCGAAGACCGAAGAGTTTGTGCTCAACAGTCCCAAGCTCACGGCGTA

CAAATGGTGGCCTGGTGGACTGGCGCACACAGCCAACCACTGCGTGGTTATGGCACACACCTACAGCCAGGGCAAGAGCT

GCGGCATCCAGCCCTTCGTGGTCCAGATCCGGGACCTGGAAACCCACATGCCGATGAAAGGCGTGAAGCTTGGAGAAATC

GGCGCTAAGCTCGGCTTCAACACTGTGAATAATGGATTCCTGGGCTTCGAGCACCATAGGATCCCTAGGGATAAGATGCT

GATGAAGAATTCTCAGATCATGAGGGACGGAACATTCAGAACAGGTCCCAACAGTAAGCTGACGTACGGCACCATGGTCT

ACATCAGGGTATCCATTCTGGGATCGATGGCCAATGCCTTGGGCAAGGCGGTCACCATCGCTACCCGCTACGCTGCGGTC

AGGCGGCAGTCGCAACCTAAGCCAAACGAGCCGGAGCCTCAGATTCTGGACTACGTGACGCAGCAGCACAAGCTGTTCAT

CGGCATCGCCACCAGCCACGCGTACTACGTCAGCTGCTTCTGGCTGTGGACGCTCTACAGCCAGGTCAACAAGGAGCTGG

CTGCTGGCAACCTGGACAACCTGGCTGAGCTGCACGCGCTAGTCTGCTGTCTCAAGGCGACGACGTCAGCTGACGCAGCA

GTGTTAACTGAGCGCTGCCGTCTGGCTTGCGGCGGCCACGGATACATGCTGTCTTCGAACTTGCCCCAGACTTATGCTAA

CGTCACCGCTGCCAACACCTATGAGGGAGAGAACACTGTGATGCACCTGCAGACTGCGAGGGCTTTAGTGAAAGCCGGCA

TGCAAGTAGCGCAAGGGCAGAAAGTGAGCCCCTCCATGGAGTACCTGGCCGACCAGAGCAAGATTGCCAAGTGGGACAAC

TCCGTGGACGGCATTGTTAAGGGATTCCAGAAGGTTGCTGCTGGGAAAGTGGCAGCTTGCCTGGCCAGCCTGCAGAAGCA

CACCAACAATGGGCAATCATCCGAAGACGCGTGGAATCTGACCTCAGTGCAACTGGTGGCTGCCTCTGAGGCCCACGCCC

GCCTGATTCTGGTGTCAGTATTCAAGTCCCAGATGGATGCAACGACGTTCACGCTCAGCGGGGAGACCAGAACTGTCCTC

TCTCAGCTAGTGGAGCTGTACGTGGTTTACTGGGCCCTTGAGAAAGTCGGCGATTTGCTACTGTATACCCCGATTACCGG

CATGGACGTGCAGGAGCTGCAGCAGACGTACGAGGGTCTGCTGGCGAAGATCCGGCCCAACGCCGTCGGCCTGGTCGATG

CCTTCGACTTCAGGGACGAGGTCCTAAACTCGGCTCTCGGCGCATACGACGGTCGCGTGTACGAGCGGCTGATGGACGAG

GCAATGAAGAGTCCTCTTAACGCAGAACCTNNCAATGACAGCTTCCACAAGTACCTCAAACCCCTCATGCAGGGGAAACT

GGTGCCTCACAAACTGTAA

>Cher_ACOX3a

ATGGAAGACTTGAGTTTCGTGCCTGATCTGCCGAGCGGGCCTCTGGATGCCTACAGGAGTGCTGCTTCATTCGACTGGAA

GCGTTTAAAGCTGGCTCTGGAAGGGGACATAGAGAATCTTAGACTTAAGTACAAAATATGGCACACGCTAGAGAAGGACC

CCCTCTTCTCGCACCACTCTGTGACCCCGCCCACCGAGGAGCAGAAGCGCCTCACCCAGCTCCAGGTCCTCAGGATAAAC

CAGTACAAGTTCCTGTCAGATGACGTGTTCAGATCCAGTTATAGTAAAAAGACCAGAGCACTGATGTCCCTCAACGAGGC

GGTGCAGTCCCTGAACCCGAGCGTGTCTGTCAAGATGGCGATCGGGATTTACCTCATCTCGAACGCTCTGCTCTCGCTGG

GCACCGAGCGCCACTACAAATTCTATGAGGCCACTTTAATTAAGAGAGAGATCCTAGCATGCTTCGCGCTGACAGAGGTG

GCGCACGGGTCGGACGCTCGACTCATGCGCACCACGGCTACCTACGACGCGAAAACAAAGGAGTTCGTCATCCACTCGCC

TGACTTCGAGGCGGCCAAGTGCTGGCTCGGGAACCTAGGTAGGACGTGCACGCACGGTCTGCTCTTCGCGCAGCTGATCA

CGCCGGACGGGACCAACCACGGGCTGCACGGGTTCGTGGTCCCCATACGGGACCCGGCCACGCTCGAGACGTACCCCGGC

CTGATAGTGGGGGACATGGGAGAGAAGATTGGGGTCCATGGGATCGATAACGGATTCATGATGTTTAACCAGTACCGGAT

ACCGCGCGAGAATCTCCTGAATCGGTCAGCGGATGTGACGGAGGAAGGCGTGTATGAGAGCAAGTTCTCAGAGCCTTCCA

GAATACTCGGCTCCGCCTTGGAGAATTTGTCTGCAGGGCGCATAGGCATAATGCAGGAGAGCTGTCACTCCATATCGAGC

GCTGTCTGCATCGCTATTCGTTACGCCGCCACGCGACAGCAATTCGGGACGGCAGCGCAAGAGATACCGCTCATCGAGTA

CCAGTTGCACCAATGGCGACTATTCGGATTTGTGGCTGCAGCAGTAGTGTTCAAATTATACATCCAGAATATGACACAGG

TGTATCTGAAGGTTGTGGAGAGCTCCAACGCAGGCTTGAAGCTCGACAACTTGAGCGAGACGGTGTCAGAAATCCACGCG

ATGGTGTCAAGCAGCAAGCCTATGACGACTTGGACGGTGCTCCGCGCGGCGCAGCAGTGTCGTGAGGCCTGCGGTGGACA

CGGGTTCCTCAAGAGAGCAAATCTCGGCGACATCCGCAGCAACCACGAAGTAACCGTGACCTACGAGGGAGACAACAGCG

TGCTCTCTCAACAGGCCGGCAACTGGCTGCTCCGTCAGTTCGAGTGCGCGCGCGCAGGACAGCCAGTGGACTCGCCGCTG

GGGACCGTCGCCTTTCTGCCGCGCTGGCAGGCCATACTAGCCACCAAGTTTAGCTGCCAGGGGACTGGAGAGCTTAAGAA

TACTGAATTCATCCTCTCAACCTACCGATGGCTCCTCTGCTGGCTACTAAAGGAAACCCACGAGCAATACGAATCCCTAA

TCGCTTCCGGCCTCACAAAGTTCCAAGCCAAAGCCAAAACGCAAGTCTACAAATGGCAGACTCTAACCGAAGTATACGCG

GAATACCTCGCTTTAATATTTAGCTTAGAATCCATTACACGGAAAGAAAAAGAGCTCCAACCAATTCTACAAAAACAATT

CTTGCTTTATGGTCTATGGTGTTTAGACAGGCATTTGGTGGAGTTGTACCAAGGGGGGTTCGCGGCGGGAGAGGGATTGG

CTAAGCTGGTCAGGCAGGCTGTGTTGGAACTCTGTTCGGATGTGAAGGGCGAGGTTGTCAGTATTGTGGATGCGCTGGGG

CCGACGGATTTCGTGCTCAATTCGGTGATTGGAAAGAGCGATGGCAAGTTGTACCAAAACCTACAAAAGGAGTTGTTCCA

TAACCCCGGAGCGATGCAGCGGGCGTCGTGGTGGCGAGAGGTAGTGCCTTCCAAACTC

>Cher_ACOX3b

ATGGAGGTGAAAGAAATACCACCAGCCAGTGATGAAGAACTCAAGAGTTGTTTCTCTGATCTGCCTTCAGGGCCTTTGGA

CATATATAGAAAGAAGGCTTCTTTCGATTGGCGGCGAATGAAACTAGCTTATGATAGCTTAAACACTATTAAAACTAAGA

ATAAAGTATGGAACTTCATGGAGTCCCATGCCCTCTTCAAACACCCGGAAGCCACTCCAACATTGGATGAGCAGCGCCAA

ATCGCCACAAAGAGGATGTATGTCATCCACAATGTTGACTTTGTGCCTTTGGAAGAGATAGCAGACGACCCGAGACTGTT

CCAATCCTTCACGGAAGCAATCTTCATGTTCGACAGCTCGCTAGCCGTCAAACTGTCTCTAACATTCCGTATGTTCGCTA

ACACCATCCGAGGCAGCGGCAGGGAACACCACTTCCATCTAATCGAGGATAATGACAATGGGAAGATCGGTGGATGCTTC

GCGTTAACGGAGATTGCTCACGGGTCGAATGCTAAAGGCATGAGGACAACTGCTACGTATAATGTTGAGGAGAAATGCTT

TGTCATGCATACTCCTGATTTTGAGGCAGCTAAGTGCTGGGTCGGATCTATGGGAAAATGCGCGACCCACGCAATAGTGT

ACGCGAAGCTGATCTCAAAGGGCAAGAACCACGGGCTGCACTCGTTCGTGGTGCCCATTCGGGACCCGCGGACCCTGAAA

CCCTTCGCTGGTATCACTGTGGGCGATATTGGGGAGAAGGTTGGGCTTAATGGGATCGATAATGGTTTCCTTATGTTCAA

CAAATACCGGCTGCCCAAAGAGGCATTACTGGACAAACTGGGAGGCGTAGACGACAACGGCGACTACAAAACTCCTTTCA

AAGACCCCAGCAAGAGGTTTGGAGCTGCCCTGGGTATTCTGTCGGGCGGCAGAGTGCACATTACCTCCATTTCAACCAAC

TACCTTCAGAAAGCAATAGTCATAGCTGTCAGGTACTCAGCAGTCCGAAGGCAATTCGGACCAGAAAATGCTACTGAAGA

GACTCCAGTACTGGAGTATCAGCAGCAGCAAATCCGCCTGCTGCCATACTTGGCAGCAACTTACGCTATGCGCGTGTTCT

GCAACTGGTTTGGGGCCATGCACGTTGCTATGACCATTAACAGCATGCTGGGTGCGGACGACAACGCAGCGGCGCGTGGA

ATAGAGATGCACGCCCTCTCGTCCGCTGTGAAGCCTGTGTGCGGCTGGACGGCCAGGGACGGCATACAGAATTGCCGCGA

GTGCTGTGGCGGGCATGGCTATTTGAAAGCCTCCGCAATCGGCGACCTCCGCAACGATAACGACGCAAACTGCACATACG

AAGGCGAGAACAGCATGCTACTACAGCAGACCAGCAACTGGCTGCTAAATGTTTGGGCCAAGAGAGCCACACCAGGGTTC

GCTGATACCCCGTTCGGTTCCATCGAGTTCCTGAACGGAGCTCAGGGTTTACTGGCTGGGAAGTGCCAATGGAACAGTGT

GAAGGAGATTGTGCAACCTGCCAATGTAGTACACATGTACAAATGGCTGACAGCCTACATGGTCAAGCTGACAGCGGAAA

AGGTGGCCCAGCTGAAAAGCCAGGGCAAGGACAGTTACGAAGCCAAGAACGATTCGCAGTCTTACAACGCTGTCACACTG

TCTGCGGTGTATGGAGAGAACTTCATCGTGAACCATTTCTACAAAGCGGCGCTGGCGTTTAAAGATGCGGCGTGCAGAGC

CGTGCTGCTGAAACTGGTGTCGCTGTACGGATCTTTCCTGCTGGAGAAACACATGGCTACTTTATACATTGGTGGGTTCT

TCTCAGCGGAACAAGGCCTGCTCCTCCGTGAGGGTATACTCTCTCTATGCGCTGAGCTGACCCCAGAAGCCGTGGCGCTA

GTTGACACCCTGGCGCCCCCTGATTGGTGCTTGAAGTCGGTGCTAGGGCAGGCTGATGGTGAAGCCTATAAGCATATCCA

AGACAGCATAATGACGTACCCTGGATCGATGACAAGGCCTGAATGGTGGCGCGACGTCGTCCACTGGGAGACTTACGTAC

CGGCTAAATTGTAG

>Cher_AGPAT1

ATGATTAACTTAAACATAATAAAACAATCGACCTTCGTCCATGTCTGTTTTGCTATATCCTATTTCACATCGGGAATATT

ACTCAGCATGGTGCAAGCATCCTTATTCTATGGACTCAGACCTTTCAACAGGAGTTTGTACAGAAAAATAAACTATTATC

TTGCTTACTCACTGAACAGCCAGTTGGTGTTCATGTCACAATGGTGGGCAAGGACCAAGATGTCAGTTTACATCAAGAAG

GATGAATATGACAAATACTATGGCAAAGAACATGCTTACCTTTTCATGAACCATAGCTTTGAAATAGACTGGCTTATGGG

CTGGCATTTCTGTGAAGTCATCAAAGTTTTAGGAAATTGTAAAGCCTACGCCAAGAAATCAATCCAGTACATGCCGCCCA

TCGGATGGATGTGGAAGTTTTCCGAATTCGTGTTTTTGGAGAGGTCATTCGAGAAAGATAAGGAAACTATAAAGACGCAG

ATAAAAGAAATATGTGACTACCCCGATCCAGTTTGGCTATTAATGACTCCAGAGGGCACGAGATTCACGAAGAAAAAACA

CGAGGCCTCGTTAAAGTTTGCGAAGGAGAAAAATCTACCTCTCCTCAAGCACCATTTGACGCCGCGCACCCGGGGGTTTA

CCACCAGCCTGCCCCACTTCAGGGGGAAGATTCCAGCCATTTACAACATACAGTTGGCGTTTGATAAAGATGCCAAGAAC

CCGCCCACCATAACGAGCTTGCTCTACGGAAAGCAAGTCCACGCCCATCTGTATATCGAGAGGATCCCCTTGGAAAAAGT

GCCGGTGGACGAAGCAGCCGCATCACAATGGGTCCATGACGTTTTTGTTATTAAGGACAAGATGGCTGAATCTTTCCACA

ACACTGGAGATTTCTTCCTCGAATCCGGAGTGGAACGGATCGAGCCATTCGACTACCCAGTCCGCATCTACTCTTTAATC

AACACGCTGGGGTGGGCGATTATCACGCTTACGCCGATGCTGTATTATCTCATGGGATTGCTGCTGAGTGGCAAGCTTCT

GTACTTCTCTATCGGTGTCGCCATCTTCTTTGGATTTTACATCATCCTGCAGAAATCCATAGGCATGTCAAAAATTAGTG

AAGGTTCATCTTATGGGACTGAAAAGAAGTAA

>Cher_AGPAT2

ATGTCTGCCTTCAACGTCGTGCTGGGGTGCGTGATGGCACTTCTCTTCATATTATTTACGACAAGTGCTATCGCACGATA

CTATATTAAGTTCACATTTTTCATGGTAGCGTCGCTGGTGTTCTCGTCGGCCCCACTCCCGCTGATGCTGTTCAGACCCT

TCAGTCCGAAGAATGCATTACTTCCCGCGGCGCTGCTGCGGTATTCGGCGAGGCTTCTCGGGATCCGCTTCAAGGTGCGA

GGGCTCGAGAACGTGGACAACTCGCGAGGCACTGTCGTGCTGCTGAACCATCAGAGCGGACTGGACCTCTTTATACTGGC

AGTGCTGTGGCCGCTGATGGCTCGCTGCACGGTGATATCGAAGCGCTCAATGCAGTACGTGGTGCCCTTCGGCACCGCCG

CTTGGTTGTGGGGCACGGTGTTCATCGACCGCGGGTCCCGCTCCGCGCACGACGCCCTCAAAAAACAAGCGAGGGCCGTC

AAGGAAGAAAAGCGCAAGCTGTTGCTCTTCCCCGAGGGCACACGGCACGGCGGTGACAAGCTGCTGCCCTTCAGGAAAGG

GGCCTTCCACGTAGCTATGGACGCAGGGGCCGCCATCCAACCCGTCATCGCTTCCAAGTACCACTTCCTTGACTCCGAAC

GACACCGGTTTGGTTCAGGTGAGGTAATCGTCACGATATTACCCATGGTCGAAACAGCCGGCTTGACCAAGGACGATCTC

GAGGCGCTGGTAGAGAAGACTTATGCGAATATGCAGGAGACGTTCACCAGGTCGTCCCACGAAACCCGCCGGCCGGTTCC

A

>Cher_AGPAT3

ATGGCTTCCTCCTATTCATATCCTGAATTATTTTTGGCGGGTTTTATATTTACCTTGCCATTTCTTTACGAAAAAAGTGA

TGTTTTTCGATATTACCTAAAGTTTTTTCTTTATTATGTGTATGTTCTAACGATTTGCACTGTCTTGTTACCCGTAGTTC

TATTATATCCGCGCGACGTAACTAACTTAGTTGTTGCTTCCAGGTTTTGCCGTTACGCCTCTTATATAGTGGGAATAGAA

TGGGAGTTAAGGGGTCTGGAGCACTGGGAGAGTGAACAGTGTTACATTGTGATTTCCAACCATCAGAGCTCCCTAGACAT

CCTAGGGATGTTTGAGATGTGGCCTCGAATGAAGCGCTGCACCGTGGTTGCTAAGAGACCTCTGATGTTCACCGGAGCAT

TTGGATTTGGTGCTTGGTTGTCGGGGCTGGTATTCATTGATCGCCTGAAGACTGACCGAGCTCGGCTGCTCATGAGAGAA

GCTACTGAGCGTGTTATTCAAGAAAAGACTAAGCTCTGGATATTCCCGGAAGGGGCGAGGTTCAACAAAGGCTGTATCCA

GCCATTCAAAAAGGGAGCGTTCTATCTAGCCATTGACGCTCAGATACCTATAATGCCTGTTGTATTCAGCCAGTACTATT

TCCTAGATAGTGACACTAAAACGTTTGAACCGGGAAAAGTAGTGATCACCACCCTGCCACCTATACCAACAAAAGGAATG

ACCCGCGACGACATTGAAGTACTCTCCGAGATGGCGCGCCAGAAAATGATAGAGGTATTCAACGAGAGTTCTAAGGATCT

GGTAATGCAAAAAAAAATCGCCATCTAA

>Cher_AGPAT4

ATGTGGGACATAGCAATTTATCTAGTCGTAATAACTTGTGTCGCCTACGTGCTGAAACAATTATTTTCAGAGACTCCCAA

TTTCGTGAAGTTCAAGTTGAAATTTCTAATTTATTACGTATGGACCTCCTTCATTTCCCTGATGTTGATGCCATTCTTCA

TACTAGATCCAAAGAACGTGAAAAATATTCTATTCTCCGTTGCTATTATTAAACATGTAACAAAAGTAATCGAGGTGAAA

TGGCATCTAAGGAATGGGAAAATCTTGGCGGAAGACAGAGGAGCGGTGATTGTGTCAAACCATCAGTCTACTATTGATGT

TCTGGGCATGTTCAACATTTGGCATGTCGCTGATAAAGTAGCCGCTGTTGCCCGGAAAGAACTATTCTATGTGTGGCCTT

TCGGTCTTGCTGCATACCTCTGTGGAGTGGTGTTCATAGACAGAAACAATGCCAAGGATGCCTACAAACAACTGCAAGTT

ACATCAGACATCATGGTGAAAAATAAGACTAAGATCTGGCTGTTCCCGGAAGGCACACGAAATAAGGATTTCACGAAGCT

GTTGCCATTCAAGAAAGGAGCTTTCAACATCGCCGTGGCAGCACAAGTGCCAATTATTCCTGTTGTTTTCTCCCCATACT

ACTTCATCAACAGGCCGAAGTATATTTTTAACAAAGGACACGTGGTCATCCAATGCCTGGAAGCCGTGCCGACAGCGGGT

CTCACGATGGAGGACGTGCCCGAGCTCATCGACCGCGTTCGCAGCACCATGGAGATGGCATACAAAGAGCTCTCCAAGGA

GGTCCTTAGTGCCCTCCCGTCCGACTATCCGCTGTACAGTGAGAGTGCCATCTGA

>Cher_CSP1

ATGAAGACTCTCACCGTCATCTTCGTCATCTTCGTCATTTATCTCTGCCAATGCCGCGCCGAAGCTCCGACGTACACGAC

GAAGTATGACGGGATCGACTTGGACGAAATACTGGCCAACAACCGACTTCTGACCGGCTACGTCAACTGCTTAATGGACT

TAAGACCCTGTACGCCTGATGGGAAGGAGTTGAAAAAGAATCTCCCCGACGCCATATCAAACGACTGCGGCAAATGCACG

GAGCGGCAACAGCAGGGGGCCGACCAAGTGATGCACTACATCATAGACCACCGCCCCGAGGACTGGGAGCAACTCGAGAA

GAAATACAACTCCGACGGCTCTTACAAGCGAAAATACATGGAAAAGAAGGTACCTACCGAAGAAACCTCTACTGTAAAAG

TAAATGAAGAAGAAACAAAAGACAACACGACGAAGGAAACAAAAGAAGAATGA

>Cher_CSP3b

ATTATCCTCGTTTGCTTCTTCGCCCTGGCCGCGGTGGCCTACGCCCGTCCCGGGGACGTTTACACCGACAAGTTCGACAA

TGTCGACCTGGACGAGATCCTGTCCAACCGCCGACTTCTCATCCCATACCTCAAGTGCGTGCTCGAGCAGGGAAAATGCA

CCGCTGACGGCAAGGAGCTGAGATCCCACATCAGGGAGGCTCTCGAGAACAACTGCGCCAAGTGCACCGAAACTCAGCGC

AAGGGCACCCGTCGCGTCATCGCTCATCTCATCAACAAGGAGGACGACTACTGGAACCAGCTCTGCACCAAGTACGACCC

TGACCGCAAATACGTAACCAAATACGAGGAGGACCTGAAGACTGTCGCCTAA

>Cher_CSP4

ATGACATTCAGGTGTTTAGTTCTGGTCACCGTGCTCTCAGTGACTGTCGCAGACTTCTACAGTCCCAAGTACGATGAGTT

CGACATTCAGCCTATCCTTGACAATGACAGGATCATGCTCAGCTACACAAAATGTTTCCTGGACAAGGGTCCTTGTACCC

CGGACGCTAAGGATTTTAAAAAGGTAATCCCCGAGGCTCTTCATACAACCTGCGCGAAATGCACCCCTAAACAGAAGCAG

CTGATCAAGCAGGTCATCAAAGGCATCATGGAGAAGCACCCCGACTCCTGGAAGGAGCTGGTGGAGAAGTTTGACCCGGA

CAAGAAGTATAGAGACAACTTTGACAAATTTCTAGTTGAAGATAGAAAGTAA

>Cher_CSP6a

ATGAAGACTGTCCTGATTTTGTGTCTGGCCGCGTTTGTGGTGGCAGAAGAAAAGAAATATGACGACGTTGGTGATGTTGA

TCTTTCGAGCGTGCTAAACAACCAGAGGTTGTTGGTGCAGTACACCAACTGCCTAATTGACAAAGCCCCCTGCACCCCTG

AATTGAAACAACTGAAAGAAAAACTGCCAGAGGCTCTGGAAACCCATTGTGCCAAGTGCACAGACAAGCAAAAGACGGTC

GGAAAGAAGTTGGTTCAGGACTTGAAGGCGAAGCACCCGGATCTATGGCAGAAACTGGTTGCCAAATATGATCCTACTGG

CAAATACCATCAGTCTTTCGAAGATTTCCTTAAGAACTGA

>Cher_CSP7

ATGATGATCAAATATTTCGTGGTCCTGTGCTGTGTGGCGTTTGCGCTGGCCGAGGAAAAATACACGGACAAGTATGACAA

CATCGACTTGGACGAAATCTTGGACAACAAACGGCTGCTCCAGGCTTATGTTAATTGCATACTCGATAAGGGCAAGTGCA

CCCCTGAAGGCAAGGAGCTAAAAGATCATCTCGAAGACGCTCTCACGTCAGGTTGCGAGAAATGCACAGAAGCCCAGAAG

AAGGGGTCATCAAAGGTCATTGAGCACCTCATCAAAAACGACCAGCCCATCTGGCAGGAGCTGACTGCACGCTTCGACCC

TGAGGGGAAATTCAAGAAGGTGTATGAGGAGCGCGCGAAAGCCAATGGCATCACGATCCCGGAGTAA

>Cher_CSP9

ATGAGGAGCTACATTGTGTTCGCTTGCCTGCTTGTGTCGGTGTTCGCCGCTGATAAATACAATTCGAAATATGACAACTT

TGATGTGGAGACCCTGATCACAAATGACAGGCTGCTGAAGTCGTATGTCAACTGCTTCTTGGACAAGGGCCGGTGCACGC

CCGAAGGCTCGGATTTCAAAAAAACACTCCCCGACGCCGTTGAGACAACGTGCGAAAAGTGCACAGAAAAGCAGAAGATC

AACATCAAGAAAGTGATCAAAGCCATCCAGGCCAGGCACCCGAAGCAGTGGGACGAGCTCGTCAAGAAGAACGACCCTAC

CGGCAAATACATCGCGAACTTCAACAAGTTCATTGACAGCTAA

>Cher_CSP10

ATGAAGTTCGTCATTGTCCTGTCAACCCTGGTAGTCCTGGCTTTCGGGCAGGGCACCTACACCTCGGAGAGTGATAACCT

GGACATCGATGCCATCGTGAGCAACCCGGAGAAGCTGCAGGCCTGGTTCGGCTGCTTTGTGGATAAGAGCCCTTGCGACA

AGGTGCAGAGCAGCTTCAAAGTGGACATGCCCGAGGCGATCCGTGAAGCTTGCGCAAAATGCACCTCAGCACAGAAGGCA

ATCCTCAAGAAGTTCCTCACAGGCCTTCAGGAGAAGTCACCAGCCGACTACGAAGTCTACAAGAAGAAATACGACCCAGA

AAACAAATACTTTGCACCACTTAATAAGGCTATTGCATAA

>Cher_CSP13

ATGTTCTATTTATACCTGTGTGTGTTGTTGTTTGGTGTAGCGTACGCGCAAGATGCGTTAGTGAACACGACCCAAGGCCA

AGTGCAGGGGGCATTGGCGAACGATGGAAACTACTTAGTGTTCTACGGGATCCATTATGCTGGGTCAGCGTCGGGGACTA

ACCGATTCAAGCCACCAACGCCGCCTGCGCAATACCCAGGAGTGTTCCATGCGGTTGACAACAGCGTCATTTGCGCGCAC

CCCACCCCGCGCGGCATCGTCGGAGTAGAAGACTGCCTGGTCCTCAGCATCCACACAAGAAACCTGACTTCACCCAAACC

AGTCTTAGTCATTCTCTTAGGAGACGAGTACACCTCCATAGACACCACGACTTACTCCTTCAGCCGGCTAGTCCTCTCTG

ATGTCGTAGTAGTGAAGATGAACTATCGACTATCGATCTTCGGCTTCTTGTGTTTGGGCGTGCCGGACGCACAGGGCAAT

GCCGGATTAAAGGACGTCGTTCAAGGATTAACTTGGATTAGGAACAATATTGCCGGATTTGGTGGCGATCCGAATAATGT

TGTACTGTTCGGACACGGCTCAGGAGCGGCCATGGTTGATTTAATTACGATGTCGCCTTCAGCGGACAATTTAGTGCACA

AAGCAATTACGCAAAGCGGGTCCGCTCTCGCGCCCTGGGCAGTCGCGTACGAACCGCTTCAATACGCTCGAGTGTTTGCT

GAAAAGTTAAGCTATTCCGGTGACACGCCTCAAGAACTAGCTCGAAAACTAGCGGTAACCGATTTGAGTTTATTGTCAGC

CGCATTACAAGGCTTCAGTTTCTACAACAATTCTGCCTTGTTCGCACCCTGCCTTGAAAACAGAGCGTTGAACAATACGT

TTATCACTGAAGCGCCTATCGACCTACTGCGTTCTGGAAACTATAGCCAAATCCCTTACATTGCCGGGTATACTGACAGA

GAAGGCACGATCAGATCAGGACAAGCTGTAAACGATGGATGGCTAGAGCAGATGGAAGCGAACTTTACAAACTTCGTCCA

GGTTGATTTAGCGTTCAGCAGTATAGAGAACAAGACTGCTGTAGCGACTAACATCAAGCAGAACTACTTCGCAACGAGTG

CTGTGAGCATGGCGACTATAAGAGATTACTTGAACTATCACGGAGATACAATGATACGTGTGTCGGTGATACGAGGGGCT

CGGGAAAGAGCGTTGACTTCAAGAGCTGATGTAAGACTGTTGGAATTCACATACAGAGGGACCAGGAACTCTGATTGGAT

TTTCCAACAGATACCTATTGACGGGGTGCCCCATGGCGGCCTGCTAAACTACTTGTTTGATTACGACTTAAATCCTGCAG

ATGAAGCAATCAGAGCTTCGATTTACAGACGTTACATTTCGTTTGCCAATACCGGAAACCCTATAAACAATGCCTCAGAA

CAAACTATGTGGAATCCTGTCACCAGCTCAGCTATCAACTACCTAATCTACACTGGAGAAAGCATCAACCAATCTACCAT

CTGGGGAGAATCTGCTCGGGCCAACCCCCACTCAGAAATCATGACATACTGGGACAACCTCTACCGCATTTACTACCAGC

CTCCAGTTTCTATGTCGTCGGCTGATAAAGTGATTAGCGTTGCATTTGTTTTGTTGCTATCTCAATTTGTGCAGCGGCTG

TTATAA

>Cher_CSP14

ATGAAGACCGTGCTAGTTCTATCCGTGTTGGTGGCAGTGGCGCTCGCGCGGCCCGACGAGCCCTTCTACAACCTTCGCTA

CGACAGCTTCGATGCGCAGGAGTTGGTCGGCAACATTCGCCTGCTGAAGAGCTACGGCAACTGCTTCCTCGGCAAGGGAC

CTTGCACTTCTGAGGGGACCGATTTTAAGAAAACGATCCCAGACGCTCTGAGGACCAGCTGCGCCAAATGCTCGCCGAAG

CAGCGCGAGCTCATCCGAACAGTGGTCAAGGGTTTCCAGTCCAAGCTGCCTGAGCTCTGGAAGGAGCTATCCCAGAAGGA

AGACCCCACTGGCCAGTACAAAGCTAACTTTAACAAGTTCCTGAACTCTTCTAAGTAG

>_Cher_CSP11

ATGTGGGCAGTACTTTTCGTGTTCGCGTGTGCGTCCGTGGTTTTAGCACAGAATATAAACGATGTGAGGAACTTGCCGAA

ATACGATAAGAGATATGACTATTTGAGTGTTGATGCCATTCTGGAGAATAAGAGACTTGTCAGGAACTATGTGGATTGCC

TGATCAACGCGAAACCTTGCACGCCTGAGGGGAAGGCGTTGAAAAGGATACTGCCAGAAGCGTTGCGTACAAAGTGTATA

CGCTGCACCGAGAGCCAGAAGCAGACCGCAGTGAAGGTGATACGGCGCGTGAAATCAGAGTACCCTGAGGATTGGCACAA

GCTCGCCTCCCGCTGGGACCCTACTGGCGACTTCACCAGATACTTCGAAGAGATTCTGATAAAGGACCACTTTAATACCA

TACCGAACAGTAACGAGATCCCCGGCTCCTCGCCGACCACGCCGCCCGCACGCTGGCCCAGCACGCAGCCACCAGCACCC

AGCACGCAGCCACCGTCGCCGCTGCCGCTGTTCATACCCCCCAGCCTGCCAACGTTCCCGCCCGCCAGCCCGCCAGCGTT

CGTGCCCGCCAACCAGCCAGCGCTGGTCACTCCGCCCCGCCCGTCTGTCTTGAACCGCTTCGACGAAGATGGCGAGTTGA

TGGTGAACCAGAACAACCCGCAGTCACGCCCGCCGTCACCGCCGGTCGTCCGCCAGACCACCGCGCGACCTGTCGCCACC

ACCCGATCTTCCCAAATGACCTGGGCGACGGCGGCTTCCGACAACATCCCCACACGGTTCAACCTTCGACCAACGAAGGA

CATCTCGCCCCCATACACCACAGCTATAACCCTGATCGACCAGATCGGGTACAAGATCATACGGACCACGGAACTGGTGA

CTGACATACTGAGGAACACCGTCAGGGCAGTTGTGGGGTGA

>Cher_CXE1

ATGTGCTATCAACGGACTCTGGTCGGAGACATATTTCGGGGACAAGAACATTGTCTGATACTAAATGTGTTCACTCCGCT

GTACCCCTCGGATACTCTGCTCCCGGTCATGGTATTCATCCACGGAGGAGGATTCATAGAGGGATCAGGAACACCTTTGG

TATATGGCCCAAATTATTTGGTACCAAAAGGTGTAATTTTAGTGACAATTAATTACAGGCTTAATATTCACGGGTTTCTC

GGTCTGGGAATCAAGGAAGCGCCTGGCAACGCTGGAATGAAAGATCAAGTCGCAGCATTGAAGTGGGTTCAGAAGAATAT

AAGAGTGTTTGGAGGTGATCCTGATAATGTGACTCTATTCGGCGAGAGCGCTGGTGGTGCCTCGGTGTCGTACCACTTAC

TGTCTCCTATGTCCACTGGGCTCTTCCATAAGGCAATAACACAGAGCGGGTCAGCCCTGACGGCATGGGCACATCAAGCG

AACCCCGAGTACAATGCTCGACTTGTTGCAAAAGCTATGGGATACAATACTGAAGATCCTTACGAACTGTATAACATTTT

CAAAAACGCGGCTTTAGATGAACTTACTAAAGTAACAATCCCTCTAGATGAGAAAAAAGTGATGTTATCTAGACTCATTT

ACTCACCCAGTGTTGAAAAGGTATTTGAAGGAGTAGAACCTTTTTTGACAAAGGATCCTTACAAATTATTTCTCAATGGA

GAGTACAACAAAGTTCCTATGATTACAGGAACAAATGACGAAGAAGGCTATTTATTCGCAGGCGTAGAGGACCCTTTAGT

CATACCTAATGTTGATATCGAGCGCTCTCTACCAAATAATTTAGAATTTCCGTCAGAAGGTGAGAGAAAACGAGTTGCAG

AAGAAATTAGGAACATGTATTTGGGTGACGAACCCATATCGGAGTCAGCTTCCTCTCAGAAGAAATTTTCCAGATTTCAT

GGAGAACCATTCTTTAATTATCCTCCGATGGCTGAAACAGAACTATTGTTGAATTCAAGTGGTCTCCCGTTATACCATTA

TTACCTTACCTATGATGGGTGGAGAAACTTTGGAAAATTTGCGTCTGGTGCCATCTTCAGAAATTCTTCTGGAGTGTCGC

ATGCAGACGACTTATTTTATTTATTTTATCAACCCTTTTTACCGGGGTGGATCGAGATGGAAATGATCAATAAAATGACT

ACCTTATGGACCAATTTTGCAAAGTATGGAGACCCAACACCAGAAACGACGGAGCTGTTGCCTCTACGATGGCTACCCGC

TACCAAGGAGGCGCCGCAGGCTTTTGTTTTAGACAAGGAGCTGTCTACCATCCCAATGTGGCATAGAGAATCGTTGCGAT

ACTGGAAAGACGTGTACTCAAAGTATAGGAGGAAATAA

>Cher_CXE2.2

ATGAGTCAAGTGCAAGTCAGTGATGGCCTGGTGGAAGGTGAGCTGGTGGAGAACCCCTTGGGTGGGCAGTTCCATAGCTT

CAAGGGGATTCCGTATGCTGCACCGCCTGTTGGAGATCTGAGGTTTAAAGCCCCGCAACCCCCAACTCCATGGGAAGGAG

TCCGCAGTGCCACAGAATTTGGATCCATCTGCTATCAAGTAGACCCTTACTTCGACCCCACGCCTAAGGGCAGCGAGGAT

TGCCTGTACATCAACGTGTACACTCCTGATCTGACTCCAACCAAGCCCTTGCCTGTCATGGTTTGGATCCACGGCGGAGG

CTTTATCTCCGGCAGCGGAAACGATGACTTCTACGGCCCTGAGTACTTGGTCAGAAACGACGTCATCCTAGTCACTTTTA

ACTACAGGTTGGAAGTCCTCGGGTTCCTCTGCCTTGACACTGAAAAAGTGCCCGGAAACGCGGGTATGAAGGACCAAGTA

GCGGCTCTGCGTTGGGTCCAGAAAAACATCAGCCAGTTTGGTGGAGACCCTGACAATGTCACCATTTTCGGAGAGAGTTG

TGGAGCGGCTAGCGTCAGCTTCCACTTGATCTCGCCCATGTCTAAAGGACTGTTCAAACGCGCTATTGCGCAGAGCGGTC

ATGTCACTAATGGCTGGCCGGTCTCTGAAGTGCCGCTGTTGAGGGCTGCTGCGTTAGCTCGACAGCTGGGCTGCGAATCC

AATGATCCTCAGGAGTTAGCGACTTTCTTTAAAGAGCAACCTATGTCCTCCCTTGTTAAAGTGAGGCCCCATATCTATTT

AGCTGAAAAAGTGATGGATGCCATCGATATTTACTTCGCAGTGGCCAGCGAGAAGCAATTTGGTGACAACGAGAGATTTT

TCTATGGCAACCCTATCGAAGCGCTGCGTAATGGTATCCATGAGGGGGTAGAAGTCATCACCGGGTACAATGAACACGAA

GGTGTGTTCAGTTTCCTTCTCAGAGGAGGCTATGATAAAATTAAAGATCAAGCAAAAACCTACTTTGAATACTTCGTTCC

GAGACCTTTCTTGTTGAACTGTGCGCCACCTGTGCAGTTCGAGGCAGCCATGAAAGTAAAACAATTCTATGGGAATGGAG

GCAAGGTGGAAGACGTTGACACGTTGACGACGTTCTGTACCGTGGAAGATTTCGCATTCGGAATCATACAGTGGCAAAAG

ATAAGTGCGAAAGCGAGTAAGAATAAATTTTATTTCTACAAGTTTTGTTGCAAGTCTGAGTTGAATGTGCTGACTCATGC

CTTCGGGCTGGGGGAGACCTTTGGACCTCGGCATATGGTGTCCCATGCTGATGATCTGGCATACCTGTTCCCCATGAAAA

TTATGAACATGAAGATTGACCCTGAATCCGCTACATTCAAAATGGTTGACAATGTGACGAAACTTTGGACCAACTTTGCT

AAATACGGGAACCCGACGCCAGACGAAAGCCTGGGTGCACAGTGGCCTGAATACACCACAGAGACAGAAGCTTACCTGGA

CATCGGAGAACAGCTGGTCCCATCATCAGCTCCCAACCAGAAGGAGGTGGAAATGTATGAGAGCATCTACAGGGAACATC

TGCCGCATTTGATCGCTTAA

>Cher_CXE4

ATGGTCAAAGTAAAGGTACAGCAGGGTTGGTTGGAAGGAGACAAGCGCGAGGTGGTGACTAAGGATGGCCACTACTTCAG

CTTCAAGGGAGTGCCATACGCAGCCCCACCCGTGGGCAAACTGCGGTTCAAGGCGCCTCAACCACCTCTGCCATGGGACG

GAGTAAGAAAGGCGGTAGAACATGGTCCTGTCAGCCCCCAGCAGGACATATTCACCCAAGAACTGATCCCCGGCAGCGAA

GACTGCTTGTACCTCAACGTGTACTCGCCAGAGCTCGAGCCAAAGACACCGCTGCCTGTCATGTTCTTCATCCACGGCGG

AGGCTACAGGAGTGGCTCTGGAAATGACTCCCACTATGCTCCTGATTTCTTAGTCAAACACGGCGTGGTCCTGGTAACCA

TTAACTACAGATTAGAAGCCTTTGGATTCCTTTGCCTGGACACCAAAGATGTCCCTGGTAATGCGGGGTTAAAAGACCAA

GTTGCAGCTCTGAAGTGGGTGAAAGAGAATATAAGCAAGTTTGGTGGTGATCCCTCCAATGTTACGGTCTTTGGTGAGAG

CGCTGGAGGAGCGTCAGCAGCGCTCCATGTCCTGTCTCCGTTGTCGAAAGGGTTGTTCAAACGATCCATCCCAATGAGTG

GTGTTCCATTGTGTGACTGGTCCGTGGCCTTCCAACCGGCTAAGCGAGCCTTCACTCTCGGTAAAATATTGGGATTCGAA

ACTGATGATCCAGATAAACTTCTAGAGTTTTTACAAGGCGTTCCATCAGAGAAACTCATCCTTACCGATCCTTGCGTATT

GAGTTTTGAAGAAAAAGCCAACAACCTTTTGAAAATGTACCACTTTACGCCTGTGGTTGAAAAAGACTTTGGACAAGATT

ATTTTATGATCGAGGAGCCGTTGGAAATTTTGAAACAAGGAAAAACTAATGCAGTTGATGTCCTAATAGGACATACGAGC

GAAGAATCTCTTCTTGGAGTGCCTGTTTTCGAAAGCGTGTTAATAAAACAGTACAACAGGTATCCCGAAATATTAGTGCC

TCGGGAACTTCTTTACCAGTGCACTCCTAAAAAGGTTCTAGAACTGTCACATAAAATCCAAGAGCATTACTTCGGCAAAA

AACCGATCGGTGTCGACACCATGAAGGAATTCGTCGGCTATGTTTCCGAAACCTCGTTCATATACGATGTCCACAGGTAC

TTGGAGAAATTACCGAAGAATGGCAAAAACAAGACATATTTCTACAGATTCTCCAGTGTTTCTGAGCGCAATATTTATGG

CGCTAGCGGTGCTAAGTTCGGGCTTACTGGTGCAAGCCACTTGGACGACTTGATGTACCTGTTCCAGGCCAACTATGCCA

GTGTACCGATTGACAAGAGCGGCAAAGGGTATAAGATGATACAGCTCGCTTGCACAGTCTTCACTAACTTTGCGAAATAT

GGTAACCCAACCCCTGACGCCTCTCTGCCGACCTGGCCGACCTACGACAACGCCTCGAAGAGCTACGGAGACATCAGCGA

CACCGTGACCATTGGACAAGCACCGCACTCCAAAGCAGTAGCCTTCTGGAAGTCCGTGTTTGATGCGGCTGGAGTTGACT

TCTAA

>Cher_CXE6.1

TCCGCGCAGAGCGAGGCGCGCGAGTCGCGCACCGTGCAGACCGCGCAGGGACCCGTGCGCGGGTACAAGAATGACGGAAT

ATATGAATTTTACGGAATTCCTTACGCTACAGCACCCACTGGTCAGGACAGGTTCAAAGACCCACTCCCAGGACCAGTTT

GGATGAACCCTTTAGACGCAGTCGATAAAAAGATAGTATGTCCACAAGGCAAGTTTACATTCGTGGATATCACGACGTAC

ACCATGCAAGAAGACTGCTTAATCGCAAATGTTTACATGCCGGACACAGAGGAGACCAATCTACCCGTACTAGTGTATGT

ACATGGTGGAAGTTACCAAGTAGGATTTGGTTACTTATTTGGACAGAAAAGTTTGGTAAAGTCTCAAAAAATTGTTGCAA

TCAACTTTAACTATCGCCTTGGTATACACGGCTTTCTATGTCTCGGCACTGAAGGCGCTCCCGGCAACGCGGGCATGAAG

GACCAAGTCGCGCTGCTGCGCTGGGTGCAGAAGAATATCGCAAATTTTGGTGGAAACCCTAATGACGTCACTATTGCTGG

AGGCAGCGCAGGATCCTCAGCAGTAGATCTGCTTATGCTTTCTAAAGTGACAGATGGTCTCTTCAATAAAGTTATACCCG

AGAGTGGTGCCAGCCTAAGCCCATTTAGTATCCAGCTGGACCCAGTACAGACTGCGAAGGAATACGCCACAAAACACGAT

TTTAATGAAGTTGACGACGTTCACGCCTTAGAAGATTTCTACAGAACGGTTTCCTATGACGTATTACTCAGTGACATATC

CCTTGACTCAAACATATCCTTAATGTTCATACCATGCGTTGAGCGCTACGTTGCAGGCGTTAAAACGTTCCTTGAAGATA

CTCCAATAAATATTTTAAAACGAGGAACATACAAAAAGGTTCCTGTACTCTACGGATTCACAAATATGGAAGGCTATATG

TTTCTAGGGGCATTTGACGCTTTAAAAGATAAAGTGAATGAGCGCTTCTCTGATTATATACCGAATGATTTACAATTTGA

CTCTGAAGCGCAGAAGGCTGAAATAGCACAAAAAATTAAAGAATTCTATTTCGGAGACCAACCCATTAGTGAAGATAATA

TTGTTGATTTTATCAACTATAACTCTGATATTGTGTTCGGTTATCCGCATCTTAGATCTACACAATTGCAAGTAGAAGCC

GGTAGCAATTCGATATATTTGTATGAATATTCATTTCCATCTCCTGTCCCTGAAGGTGATCATGTACCTGAAATCATGAA

GAAAGTTCAGGGAGCGCCTCATTGTGCACAGTCTGCAGCAGTTCACGATGTTGTATTTGACTTAGTTCCACCTGATTCGA

GTGAGGAGTATGTCAAGTTGAGAGAAACTTTGAGAGAGATGTGGCTGAACTTTATAATCACTGGAAAACCGGTTCCTGAA

GGATCGAAATTACCAGCATGGCCTCCAGTTGGTGCCAACCGATCCCCTTACATGGACTTAGGAGAGAAGATAGAGCTCAA

AGGTTCACTTCTAGAGGAGCGAGCTCGATTCTGGGATGGCATCTACGAACAATTCTACAAGTCACCTATTGCGCCGAAGC

ACACACGAGTTCGAACTGAACTGTAA

>Cher_CXE6.2

ATGAATCCTTTAGACGCAGTCGATAAAAAGATAGTATGTCCACAAGGCAAGTTTCCATTCGTGGATATCACAACGTACAC

CATGCAAGAAGACTGCTTAATCGCAAATGTTTACATGCCGGACACAGAAGAGACCAATCTACCAGTACTAGTGTACGTAC

ATGGTGGAAGTTATCAAGTAGGATTTGGTTACTTATTTGGACAGAAAAGTATGTCGAAGTCTCAAAAAATTGTTGCAGTC

AACTTTAACTATCGGCTTGGTATACACGGCTTTCTGTGTCTCGGCACAGAAGGTGCTCCCGGTAACGCGGGCATGAAGGA

CCAAGTCGCGCTGCTGCGCTGGGTGCAGAAGAATATCGCAAATTTTGGTGGAAACCCTAATGACGTCACTATTGCTGGAG

GCAGCGCAGGATCCTCAGCAGTAGATCTGCTTATGCTTTCTAAAGTGACTGATGGTCTTTTCAATAAAGTCATACCTGAG

AGTGGTGCCAGCCTTAGCCCGTTTAGTATTCAGCTAGATCCAGAACAGACTGCGAAAGAATACGCCAAAAAGCATGATTT

TAATGAAGTAGACGACATCTACGCCTTAGAAAATTTCTACAGAACGGTTTCCTATGAAGTATTACTTAGTGACATATCCC

TTGACTCAAACATATCCTTAATGTTTATACCATGCGTAGAGCGCTACGTTGCAGGTGTTGAAACGTTCCTTGAAGATACT

CCAGTAAATATTTTAAAACAAGGAACGTACGAAAAGGTTCCTGTACTCTACGGATTCACAAATATGGAAGGCTACATGTT

TCTAGGGGCGTTTGACGCTTTAAAAGATAAAGTGAATGAGCGCTTCTCTGATTATATACCGAATGATTTACAATTTGACT

CTGAAGCGCAGAAGGCTGAAATAGCACAAAAAATTAAAGAATTCTATTTCGGAGACCAACCCATTAGTGAAGATAATATT

GTTGATTTTATCAACTATAACTCTGATATTGTGTTCGGTTATCCGCATCTTAGATCTACACAATTGCAAGTAGAAGCCGG

TAGCAATTCGATATATTTGTATGAATATTCATTTCCATCTCCTGTCCCTGAAGGTGATCATGTACCTGAAATCATGAAGA

AAGTTCAGGGAGCGCCTCATTGTGCACAGTCTGCAGCAGTTCACGATGTTGTATTTGACTTAGTTCCACCTGATTCGAGT

GAGGAGTATGTCAAGTTGAGAGAAACTTTGAGAGAGATGTGGCTGAACTTTATAATCACTGGAAAACCGGTTCCTGAAGG

ATCGAAATTACCAGCATGGCCTCCAGTTGGTGCCAACCGATCCCCTTACATGGACTTAGGAGAGAAGATAGAGCTCAAAG

GTTCACTTCTAGAGGAGCGAGCTCGATTCTGGGATGGCATCTACGAACAATTCTACAAGTCACCTATTGCGCCGAAGCAC

ACACGAGTTCGAACTGAACTGTAA

>Cher_CXE11

ATGTCGTCTATAAGTATATTGTGTGCTCTTTTACTCTTTAAGAGTGTTGTCAGTGAATTTAGGACAGATCCCTTAGTTAA

TACTAATGTAGGGTTAATCAGAGGATTACCAGCAACGGACGGGGACTATTCCATGTTTTTGGGGATACCATATGCCACTG

TGGATAAGGCCAATCCTTTTAAGGCTTCCATACCCCACCCAATATTCGAGGACACTTTCGAGGCCTACGACGACTCGGCT

CTCTGTCCTCAGTTAGATGACAACACCAACACCATCAGAGGAACTCTGGACTGTCTCCATCTCAATGTCTTCTCACCGAA

CTCTGCACACTCCGGCAACCTCCTGCCAGTAATGGTCTGGATCCACGGGGGTTTTCTACAAAGAGGCGCTTTCGGCAGGC

AGACTTACGGCCCAAAGTTCCTTGTCAAACATGAAATTATTTTAGTAACTATTAATTACCGCTTAGGACCTTATGGGTTT

ATGTGTCTTGACACACCAGAGTATGCTGGTAACCAAGGACTGAAAGACCAGATAGTAGCTTTGAAGTGGATTCAAGATAA

TATTAGAGCTTTCGGTGGGGATCCAGCTCAAGTCACCTTGGCAGGACAGAGTGCTGGAGGTGCAGCCGTAGATTTCCATT

TGTTTTATCCTGGAGAAAGGCTCTTTAATAAGGTAATACTACAGAGCGGAGTATCTTTAACTCCTAGAAGACTTGTCGAA

TCAGATACCACGAAGCCAATTACGCTAGCTGCACATTTCGGATTTGTTACCAGCAATTTAGACGAAGCTCTATCATTCCT

AGCAACCGTAGAGACCGATTTTATCATAGCAGCAACGCTTGAACTAGGGTTAGAATTCAGATCGTGCGTTGAGAAGAAGT

TTGATGGTGTGGAAACATTTATACCGCAGCATCCTGCTAATGCCGATAGACCACAAGTTTCAAACATTCCTGTCCTGATA

GGGACCGTTGACAAAGAACTGTTAGCAAAATATATCAACCAAAATTCTGAATATTTTAGAAACCTTAATGTGTTTGAAGA

GGAATTGGAAAAGACATTCGATTTCGGTGAGAATTTGGACGCTATGCATGATGTTGTAAGAAACTTTTACGTCGGTGATG

ATGTCATGACTGAAGATGACAGATGGAGTGTCATAGACTTTGAATCAGATTTTACTTATGTCCATGCTGCCCAGCGCGCT

ATGAGGAAGTATATTCAAAGTGGGGCAGAAAATATATTCTTCTACGTCTTTACTTATGAAGGTGGAAGAAATATTATTAA

ATATAGGAATGGCATATTACATGTGCCAGGAGTGGCTCATGCCGATGAACTGGGGTACATTTTTGACCCCTCGTTTATCA

CAGAAGAACCTACTCCAGCAGACCAGGCTGTTATTGACAGAATGACTACAATGTGGACTAACTTCATGAAGCAAGGAAAC

CCAACCCCTGAAACATCAGAGCTGCTGCCCGTCTCGTGGACACCAATCACAACTGAAAAGTGGCACTGCCTCAACATAGG

TGCAGAGTTAACCCTCCAAGGCAGGCCTTTCCACAGGAGAATGGCTTTCTGGGATCTGTTCTTTAGCATGAATGGGAAAT

TAGAAAGGGGAGTAGAGATTGTAAGAGAGTAA

>Cher_CXE12.1

ATCACATTCAACTACAGGATCGGACCGTTTGGTTTCCTCTCGCTGAACTCTTCGAGTATCCCGGGCAACGCGGGGCTGCG

GGACATGGTGACGCTGCTGCGCTGGGTGAAGGACAACGCGGCGGCCTTCGGCGGCGACCCCGACGACGTCACGCTGGCGG

GCCAGAGCGCCGGCGCCTGTTCCGCGCATCTGTTGACGCTGTCTCAGGCGGCGCAGGGGCTCTTTAAGAGAGCTATATTG

ATGAGTGGGTCAGGCGTACGAAACTTTTTCTCCCCTTCGCCAGTTTATGCGCAGTACGCAGCGGAATTATTCCTCACCCA

GCTTGGCATCAACGGCACCGGCCCCGAGGAGGCGCACCGCCTGCTCGTCGCTATGCCACTCGAGGATATCATAGCTGCCC

ATAACGCTCTTCAAGACACTATCGGTATCACAGTTTTTGCACCGGTCGTCGAATCTCCACATCCTGGCGTGGAAATAATC

TTGGATGACGACCCGGAAGTTTTGCAAGCTCAGGGACGAGGCAAAGACATCCCCCTGCTAATAGGTTTCACAAACGCCGA

GGGCGAAACTTTCCGCCCCATATTTGAGGCTTCTGGCATAGCGAGTCAAATCGAAAGCAACCCTCAGGTGTTGCTACCTC

CAGGGGCGTTATATGCCCTGCCGACGGACGAGGTCCTTCCCAAAGCGAGACAAATCGAGCAGAAATATTTCAACGGAACG

GCGACTCTGGACAGGTTCTTAGAGATGGCTACAGACTTTTACTTCGTGTATCCTACGTTGAAGCTGGCGGAAGTGCGAGC

CGCGAATGGGGGCGCTCCCTTGTACATGTATCGGTTCGCGTACAACGCCGACTACAGCGTGTTCAAGGAAGCTCTTAACC

TGACATACAGCGGTGCGGGGCACTCGGAGGACGTCACCTTCGTGTTCCGCGCGAACTACTGGCTCGGGAACATGACGTTG

TCATCAAACGACGAAACGATGGTGGACATCATGACGACGTATTTCACTAATTTCATGCGATACAGTGATCCATTAAACGG

AGAAGCTGGGTGGCCGGCAGCAGGTGCAGAAGGTGAGGGGATCCGGTACCAGGACATCGCCGGCTCCGAACCTAGCTCCG

TGGAGGTGAGCGGGGCATATCAAGAGGTGCTA

>Cher_CXE12.2

ATGAGGGACAACGTCGTTGTCATCACATTCAACTACAGGATCGGACCGTTTGGTTTCCTCTCGCTGAACTCTTCGAGTAT

CCCGGGCAACGCGGGGCTGCGGGACATGGTGACGCTGCTGCGCTGGGTGAAGGACAACGCAGCGGCCTTCGGCGGCGACC

CCGACGACGTCACGCTGGCGGGCCAGAGCGCCGGCGCCGCCGCCGTGCACCTACTCACGTTGTCTGAAGCCGCACGGGGA

CTATTCAAAAGAGCCATACTAATGAGCGGCAGCAGCTCACGAAGTTTCTTCTCGACCTCACCGGCTTACGCCCAGTACGT

GGCACAGCTGTTCCTCACAAAACTCGGCATCAACGGCACCGACCCCGAGGAGGCGCACCGCCAGCTTATAGCCACGCCCA

TCAAGGATATCGTCGATGCACATGACTACCTTCAAGATATTATCGGTATAACAGTTTTTGCACCGGTCGTCGAATCCGCC

CAACCCGACGTGGAAACAATTTTGGATGACGACCCGGAAGTTTTGCAATCTCAGGGACGAGGCAAAGACATCCCTCTGCT

GGTGGGCTTCACAAGCGCTGAGTGCCAAACGTTCCGCCCACGGTTTGAAGCTCTCGACATCATGAGTCGGATCGAAGAAA

GCCCTGTGCTGGTGATGGCCCCCGGGGCAATTTATGTTACGCCACCGCAGGAGATGCCCAACAAAATAGGACAAATCATA

CAGCGATACTTCAACGGAACACCGAACTTGGACAAGTTCATAAGGTTGTGTTCGGATTCCTACTTCGTATATCCTGCTTT

AAAGATTGCGGAAGCGAGAGCCGCGAGCAACGGTGCGCCCGTGTACTTGTATCGTTTCGCGTACGAAGCCGACTACAGCG

TGTTCCAGAGAACACTCGGGCTGAAGTTCCGCGGCGCCGGGCACTCGGAGGACCTCACCTTCGTGTTCCGAGCCGACCAC

GTGCTCGGGGAGAGGCCTTTGTCCTCGAGAGACCGGATGATGGCTGACGCCATGACGACGCACGTCACTAACTTCATGCT

GCATAGTAACCCAACTAACGGAGCATTAGGGTGGCCGGCAGTGACGCCCGAGAAGCTGCAGTATCAGAACATCATCACCC

CCGATCTCAGGACCACCGAGGTCAGCGGGAGTCAGCGCGATATGATGACGTTCTTCGATGGCATTTACAGCTGA

>Cher_CXE14

ATGTCTAAGCTGTTGACTTGGTGTATTTTTAAAGTGCTGTTCCTTCAGTCACAATGCGCTTCCTTACACACCCCCGTGCT

TCAAATCCCTCAAGGGAGATTGGTGGGCCTGGAAACCAAACGAGGATATTTGAGGCAGTATTTGGGAATACCTTACGGCA

CGGTGGATGAAAGATTTCAGGAAGCAGGACCACCCCCGCGCTGGCTCGGGGTTTTCAACGCTTCAGACATGAGCATCGCC

TGTGCCCAATACCACGAGCGGCTAGGGCTGCCCACAGGGGTCGAAGACTGTCTCACGCTGAACGTGCACACCCCTGGCCA

AGTCAGCTTCAAGCGGCTCTACCCCGTCATGGTGTTCATACACGGAGGCGGCTACAAGACTGGCAGCAACACCAACTATA

TCTACAACCCACAGCTTCTAGTCCAAAAAGGTGTCATCGTCGTCACCGTTAACTACCGCTTGGGCGCCTTTGGCTTCCTC

TGCCTCAGAATCAAAGGCGCTCCGGGAAACATAGGCTTGAAGGATCAAGTAGCAGCTCTTCGATGGGTTAAGGAGAATAT

TAGAACTTTTGGTGGCAATCCCGACTCGGTGACCATATTCGGCGAAAGTGCAGGCTCCGCGTCAGTTAGCTACCTCATTA

TGTCGCCCACAGCTAAAGGACTGTTCAGACGAGCCATCATGGAAAGCGCCTCAGCCCTCTCACCGTTCGCTTTTTCCAAC

GATCCCATTGAAAGAGCATCATTAGTTGCATCCAAGATGGGCTACAACACCAAAAACCCTTTTGAACTTTTAAGGATTTT

TCAGAATGCTACCAAAAACGACATTTTAATGGCCAGTGCATCGAACACGTCGGCTAATTTGTGGTCGAAATACGTTTTTA

GGCCGTGTGTCGAAAAACAGACGGTAAGCAGTAAACCGTTCCTTACCATGAGCCCTCAGGAAATGCTGGAGTCGGGGACT

TACAATAAAGTGTCCATGATCATTGGCTACAACGACAAAGAAGGCATACTTTATGTAAAGCATTACAACAAGCAACTATA

CCAAAAGTTAGACAATAATTTCAGTGACATACTGCCTGACAATCTTTACTTTTCCGACAGCAGAGAAAAACGAAAAGTCG

CAAGTAAAGTTAAGTCGTTTTACTTTGGAAATCGGACAATAAATGAAGATTCTGTCGACGGTTTAATAGATTTCATATCT

GATATGATGTTCCATTATCCTTCAGTGTCAATAACGGAATACTTCTTGAACCACAATCACTTGCCTATCTTCAACTACTA

TTTCCAGTATGATTCCTTTAGGAACTTGGCCAAGATTCTGTTGGGGATGAAAGGGCAGAAAGGGGCGGCTCACGGCGACG

AGCTATTTTACTTATTCCAGCCTGTTATTTTTTGGCCTGTGCCTTTAGTTGGAAACGATAAAAAAGTCGTGGAGAGAATG

ACTAGTATGTGGACGAACTTTGCTAAGTTTGGAAACCCAACATCGTTGAAATCGCCAGTGTTGACTGTAAATTGGACTGC

CAGTGACGACACCAGCCTAAGATATCTCACCATAGATAAAGGTTTATCCATGGATTCTCTGCCAAATCCCGAACGCATAG

CCTTCTGGAGGAACCTTTACAAGACACAACAAAATTACTTCTTAAAATCTAACCGTTTACTTAATATAACTTTTTAA

>Cher_CXE15.1

ATGACGAGACTGCTGCTGCTGCTGGCGCTCTGCGCTTGCGCAGCCGCGCATCATCACCATCATGAGCACCCAGGTTCTAC

AGCCACTACAGCTCCCACTAAGCCTACAGCCCCTACTAGGCCCGAGGCCCCTACAAAGCCCGAGGCCCCGACGAAGCCCG

TGCCCCCTACCAAGCCGGCGCCAGTGACGGTGACGCCCTCGGGTGTCATCCGCGGCTCCTGGATGGAGTCGCGGCGCGGG

CGCAGCATCGAGGCGTACCGAGGCGTCCGCTACGCGCAGCCGCCCACAGGGGAACTGAGGTTCCAGCCGCCGGTTGACAT

GGTGAAATATACTTCTGAAGTGGACGCGAGTGAGGAGGGCCCCGCGTGCCCGCTGCCCATCGAGCCTGGCGAGTACTACG

TCGACGAGGACTGTTTGCGGCTCAACGTCTACACGCCTGGCAACAACAGTTCCAAGAAGCTGCCTATAATATTCTTCATC

CACCCGGGCGGCTTCTACTCCTTTAGCGGCCGCAGTGACGTCTTCGGGCCCCACTATCTCCTGGATCATGGTGTCGTGCT

CATCACCATCAACTACCGCCTCGGATCGCTCGGTTTCCTCAGCACGGGCGACGCAGTGGCTCCCGGCAACAACGGCTTGA

AGGACCAAGTGTCGGCGCTGCGCTGGGTGCAGCGTAACGCAGCGGCCTTCGGCGGAGACCCGGGACAAGTGACGATCACG

GGCTGCAGCGTCGGTGCCATCAGCGTTATGCTGCACATGATCTCGCCTATGTCTAAAGGTCTATTCCACCGCGGTATCGC

ATCGTCGCCGGCACCGATGGGCAAGGAGCCACTGCCTTCGCATCAATTAGACCTGGCGCAGAAGCAGGCGCGCATCCTCA

ACTGCCCTACCAACTCCTCGCGCGCTATCATCGACTGCCTCAAGACCAAAACTTGGCAGGAGCTGGGGAACTCGCTGCTT

GGATTTTGGGATCAATTCGGCTTTGATCCCATCTGCTTGTGGACGGCTGTGGTGGAGCCGGACGTGGGGCAGCCGCGCTT

CCTGGCCGTCCAGCCGGACGCGGCGGTGCGCTCGGGCCACTTCTACAGCGTCCCGCTGCTCGTCAGCCGGACTGAAGACG

AGTTCTTTTGGAAGGCTTTCATTGTGACCAAGAACAAGACGCTCGTGGACCGCATGAACGCGGAGTGGGAGACGATCGCG

CCGCTCTCGTTCGACCTGCCCAAATTGCCCGCTACCAACGCGGCCGCTGCCAGCCGCCGCCTCAAGGAGGCGTACCTGGG

AGGGCAGGATGTGGCGGATACTGCTGAGAGCGCGGATGGCCTGGGAAAACTGTATGGAGACTCCTGGGTTGGGCTCGCGG

CACACAGGTTGGCCAACCTGATGTCCGTACATTCTTCTAAGCCTGTCTGGTACAGCGACTTCAGCTACATCGGCAACAAC

AGTTTCTACGAGGACGAAAAAACCGGGAAACCCGCTGGCGCGGCACATCATGACGACCTGATCTACCTGTTCTCCATAAG

CTACTCCCGCCCCCCGATCGCGGCGCAGTCGCCGCCGCCGCCCGCGCCGCAAGACTCAACAATGGTGGACCGCATGACCG

CCATCTGGTACACCTTCGCTAAATACGGAGACCCCAACCCCCGCCCCGGCGAGCTGCCGGAGCTGGCGACGCTCTCCTGG

CCGGCCATGAAGCCGGGAGACAGGAAGTACCTTCGCGTGGACAAGGAGTTCTCCGTCCACGAGAAGCTGCACGAGACGAG

GCTGCAAGTGTGGGAGGAGCTCTACCCCATCGTGTATTAG

>Cher_CXE15.2

AGACTGCTGCTGCTGCTGGCGCTCTGCGCTTGCGCAGCCGCGTATTCAGGTTCTACAGCCACTACAGCTCCCACCAAGCC

TACAGCCCCTACTAAGGGCGAGGCCTCTACCAAACCCGAGGCCCCTACGTCGCCCGCGCCCCCTGCCAAGCCGGCGCCAG

TGACGGTGACGCCCTGCGGCGTTTTCCGCGGCTCCTGGATGGAGTCGCGGCGCGGGCGCAGCTTTCAGTCATACCGCGGC

ATCCTCTACGCGAAGCCGCCCACAGGGGAACGGAGGTTCCAGCCGCCTGTAGACATGGTGAGGTATACCTCTGAGGTGGA

CGCGAGCGAGGATGGCCCCGCGTGCCCGCAACCCGTCGCGCCCGGGTACTACGTCGACGAAGACTGTCTGCGGCTCAACG

TCTACACGCCTGCCAAYAACAGTWCCAARAAGCTGCCGATAATATTCTTCATCCACCCGGGCGGCTTCTACTCCWTGACC

GGGCGCAGCGACCTCTTCGGGCCGCAYTATCTCCTGGACCATAACATCGTGCTCATTACCATCAACTACCGCCTCGGCTC

GCTCGGTTTTCTCAGCACGGGCGACGCTGTGGCTCCCGGCAACAACGGCTTGAAGGACCAAGTATCGGCGCTGCGCTGGG

TGCAGCGTAACGCAGCGGCCTTCGGCGGAGACCCGGGACAAGTGACGATAGCGGGCGACAGCGCCGGCTCTACCAGCATC

GGGCTGCATATGGTCTCGGCTATGTCTAAAGGTCTATTCCACCGCGGTATCGCAATGTCTGGGTCCCCGTTTAGCAAAGG

GCCAGTGCTGCCACACCAGCTCGATCTGGCGCAAAAGCAGGCGCGCATCCTCAACTGCCCCACCAACTCCTCGCGCGCCA

TCGTTGACTGCCTCAAGACCAAGACGTGGCAGGAGCTGGGGAACTCGCTGGATAAATTTTGGGATCAATTCAGCTTCGAC

CCCATCGGCTTNNNNNNNNNNGTGGTGGAGCCGGACGTGGGGCAGCCGCGCTTCCTGGCCGAGCAGCCGGTCGCGGCGGT

GCGCGCGGGCCGCTTCACCAGCGTCCCGCTGCTCGTCAGCCAGACTGAAGACGAGCTCATCTGGAAGGCCGTTGGTGTGA

TCAGGAACAAGACGCTCCTGGATCGCATGAACGCGGAGTGGGAGACGATTGCGACGCTCGCGTTCGAACTGCCCGCAAGC

ACTGCGGCAGCTGCCAGCTGCCGCCTCAAGGAGGCGTACCTGGGAGGGAAAGATTTGACGGCTACTGCTGAGAGCACGAA

TGGCCTGGGGAAGCTGTATGGAGACGCCTGGCTTGGACTCCAGGCGCACAGGTTGGCCAACCTGATGTGCCGACGCTCGT

CTAAGCCCGTCTGGTACAGCGACTTCAGCTACGTCGGCAACAACAGCTACCACGAAGACCCAGCAACCGGAAAACCCATT

GCAGGTGCAGCGCATCAGGACGACCTGATCTACCTGTTCTCAATGAGCTACTTCCGCAAGCCGATCGCGGCGCAGTCGCC

GCCGCCGCCCGCGCCGCAGGACTCGACAATGGTGGACCGCATGACCGCCATCTGGTACACCTTCGCTAAAAACGGAGACC

CCAACCCCCGCCCCGGCGAGCTGCCGGAGCTGGCGACGCTCTCCTGGCCGGCCATGAAGCCGGGAGACAGGAAGTACCTT

CGCGTGGACAAGGAGTTCTCCGTCCACGAGAAGCTGCACGAGGCGAGGCTGCAAGTGTGGGATGAGCTGTATCCCATCGA

GTATTAA

>Cher_CXE15.3

ATGGAGTCGCGGCGCGGGCGCAGCTTCCAGTCATACCGTGGCATCCCCTACGCGAAGCCGCCCACAGGGGAACTGAGGTT

CCAGCCGCCTGTAGACATGCTGAGGTATACATCTGAGGTGGACGCGAGCGAGGATGGCCCCGCGTGCCCGCTGCCCGTCG

CGCCCGGCGAGTACTACGTCGACGAAGACTGTCTGCGGCTCAACGTCTACACGCCTGCCAACAACAGTACCAAAAAGCTG

CCGATAATATTCTTCATCCACCCGGGCGGCTTCTACTCCATGACCGGGCGCAGCGACCTCTTCGGGCCGCATTATCTCCT

GGACCATAACATCGTGCTCATTACCATCAACTACCGCCTCGCCTCGCTCGGTTTCCTCAGCACGGGCGACGCGGTGGCTC

CCGGCAACANCTCCATCAGCGTTATGCTGCACATGATCTCGAATATGTCTAAAGGTCTATTCCACCGCGGTATCGCAATG

TCTGCGTCCCCGATGTACAAGGAGCCGCTGGCATCCGACCAGTTCGATCTAGCACAGAAGCAGGCGAGCATCCTCAACTG

CCCCACCAACACCTCGCGCGCCATCGTCGACTGCCTTAAGACTAAAACGTGGCAGGAGATAGGAAACTCGCTGCCTGGAT

TTTGGGATCAAGGCGCAGGTTTACAGTCAGACGCAATCGGTTTCTGGAAGCCGGTGATCGAGCCGGACTTGGGGCAGCCG

CGCTTTCTGACCGTCCAGCCGGACGAGGCGGTGCGCGCGCGCAGCATCCACGCCGTGCCGCTGCTCATCAGCCAGACTGA

AGACGAGTTCTACTGGAAGGCTTTCGGTGTGACCAGGAACAAGACGGTCCGGGACCGCGTGAACGCGGAGTGGGAGACTG

TTGCACCGGACTCGTTCGACATGCCCAAATTGCCCAAAGACAAAGCGTCCTTCGCCAGCCGCCGCCTCAAGGAGACGTAC

CTGGGAGGGAAGGATGTGGAGGATACTCCGGAGAGCGCAAAAGCCTTGGGAAAAATTTATGGAGACAGTTGGATCGGATT

TAACACGCACAGAATGGCCAACCTGATGTGCCGGCACTCGCCTAAGCCTGTTTGGTACAGCATGTTCAGCTACATTGGTA

ACAACAGCTACTTTGAGGACCCACTCACTGGGAAACCCGCTGGCGCGGCGCATCATGACGACCTGATCTACCTGTTCTCG

ATGAACTTCTTCCGCAAGCCGATCGCGGCGCAGTCGCCGCCGCCGCCCGCGCCGCAAGACTCCGTCATCGTGGACCNTTG

GTACACGTTCGCTAAATATGGAGACCCCAACCCCCGTAAAGGCGAGCTGCCGGAGCTGGCGACGCTCAACTGGCCGGTCA

TGAAGCCGGAAGACAGGAAGTACCTTCGTGTAGACAAGGAGTTCTCCGTCCATGAAAAAATGCACGAGAATCAGCTCCAA

GTGTGGGAGGAGCTGTACCCTATCGTGTATTAG

>Cher_CXE15.4

ATGGTCTCGCCCATGTCCAGAGGTTTGTTCCACCGCGGTATTGTGGTGTCGGGCTCGCCGGTGGGCAAGGCCCCGCTGCC

GCCGCATCAGTTCGAGCTGGCTCAGAAGCAAGCGCGCATCCTCAACTGCCCCACCAACTCCTCGCGCGCCATTGTCGACT

GCCTCAAGACCAAGACGTGGCAGGAGCTGGGAAACTCGCTGGGCGGCTTTTGGGACCAATTCGGTTCGGACCCGGTCGGG

TTTTGGACGCCGGTGCTGGAGCCGGACTTAGGGCAGCCACGCTTCGTGGCCGAGCAGCCCGACGCGGCCGTGCGCGCGCG

CCGCTACCAACCTGTACCGCTGCTCATTAGTCAGACTGAAAACGAGTTCTACTGGAAGGCTAACACTGTGACCAGGAACA

AGACGCTCCTGGACCGCGTGAACGCGGAGTGGGAGACTGTCGTCGCGCCGCTGTTCTTCAACCTGCCCGCGACCAACTCG

GCCGCCGCCAGCCGCCGCCTCAAGCAGGCGTACCTGGGCGGGAGGGACGTGGCCCAGACTGCGGAGAGCGCGGCAGACCT

GGGGAGGCTGTACGGCGACAGCTGGATTGGGTTCAACACGCACAGGATGGCCAACCTGATGAGCTGTCGCTCGCCGCAGC

CGGTGTGGTACAGCGCGTTCAGCTATGTCGGCAACAACAGCTTCTACCAGGATCCGCTCACCGGGAAACCTGGTGGCGCG

GCGCACCACGACGACCTGATATACCTGTTTTCGATGAGCTTCATACGGCCCCCGATCGCGGCGCAGTCGCCGCCGCCGCC

CGCGCCGCAGGACTCGACAATGGTGGACCGCATGACCGCCNAAGTGTGGGAGGAGCTGTACCCCATGGAATATTAG

>Cher_CXE15.5

ATGGAGTCGCGGCGCGGGCGCAGCTTCCAGTCATACCGTGGCATCCCCTACGCGAAGCCGCCCACAGGGGAACTGAGGTT

CCAGCCGCCTGTAGACATGGTAAAGTATACCTCTGAGGTGGACGCGAGCAAAGAGGGGCCGGCTTGCCCGCTGCCCGTCG

AGCCCGGCGAGTACTTCGCCGACGAGGACTGTCTGCGGCTCAACGTCTACACGCCTGCCAACAATAGTTCCAAGAAGTTG

CCGATAATATTCTTCATCCACCCGGGCGGCTTCTTCGCCTTTAGCGGGCGTAGCGACATCTTCGGGCCGCACTATCTCCT

AGATCGTGACATCGTGCTCATCACCATCAACTACCGCCTCGCCTCACTCGGTTTCCTCAGCACGGGCGACGCGGTGGCTC

CCGGCAACAACGGCTTCAAGGACCAAGTGTCGGCGCTGCGCTGGGTGCAGCGCAACGCCGCGGCCTTCGGCGGAGACCCG

GGACAAGTGACGATCGCGGGCTGCAGCGCCGGTTCTACCAGCCTCGGGCTGCATATGATATCGACTATGTCGAAAGGTCT

ATTCCACCGCGGTATCGGAATGTCTGCGTCCCCGATCGGCAAGGAGCCACTGCCGTCGCATCAGTTCGAGCTGGCACAGA

AGCAGGCGAGCATCCTCAACTGCCCGACCAACTCCTCGCGCGCCATCGTCGACTGCCTCAAGACTAAAACCTGGCAGGAG

ATAGGGGACTCGCTGCCTGGATTTTGGAACCAAGGCAACACTCAGTCAGACGCAATCGGTTTCTGGAAGCCAGTGATCGA

GCCAGACTTGGGGCAGCCGCGCTTCTTGACCGTCCAGCCGGCCGAGGCGGTGCGCTCGCGCAGCATCCACGCCGTGCCGC

TGCTCATCAGCCAGACTGAAGACGAGTTCTACTGGAAGGCTTTCGGTGTGACCAGGAACAAGACGGTCCGGGACCGCGTG

AACGCGGAGTGGGAGACTGTTGCACCGGACTCGTTCGATATGCCCAAGTTGCCCAAAGACAAAGCGTCTGCCGCCAGCCG

CCACCTCAAGGAAACGTACCTGGGAGGAAAGGATGTCGAGGATACTGCGGAGAGCGCAAAAGCCTTGGGAAAAATTTATG

GAGACAGTTGGATCGGATTTAACACGCACAGAATGGCCAACCTGATGTGCCGGCACTCGCCTAAGCCCGTTTGGTACAGC

ATGTTCAGCTACATTGGTAACAACAGCTACTTTGAGGACCCACTCACTGGGAAACCCGCTGGCGCGGCGCATCATGACGA

CCTGATCTACCTGTTCTCGATGAACTTCTTCCGCAAGCCGATCGCGGCGCAGTCGCCGCCGCCGCCCGCGCCGCAAGACT

CAACAATGGTGGACCGCATGACCGCCATCTGGTACACCTTCGCTAAATACGGAGACCCCAACCCCCGCCCCGGCGAGCTG

CCGGAGCTGGCGACGCTCTCCTGGCCGGCCATGAAGCCGGGAGACAGGAAGTACCTTCGCGTGGACAAGGAGTTCTCCGT

CCACGAGAAGCTGCACGAGACGAGGCTGCAAGTGTGGGAGGAGCTCTACCCCATCGTGTATTAG

>Cher_CXE15.6

ATGACGAGACTGCTGCTGCTGCTGGCGCTCTGCGCTTGCGCAGCCGCGCATCATCACCAACATGAGCACACAGGTTCTTC

AACCACTACAGCTCCCACCAAGCCTACAGCCCCTACTAGGCCCGAGGCCCCTACAAAACCCGAGGCCCCTACGAAGCCCG

TGCTCCCTACCAAGCCGGCGCCAGTGACGGTGACGCCCTCGGGCGCCATCCGCGGCTCGTGGATGGAGTCGCGGCGCGGG

CGCAGCTTCCAGGCGTACCGCGGCGTCCGCTACGCGCAGCCGCCCACAGGGGAATTAAGGTTCCAGCCGCCTGTAGACAT

GGTAAAGTATACCTCTGAGGTGGACGCGAGCAAAGAGGGGCCGGCTTGCCCGCTGCCCGTCGAGCCCGGCGAGTACTTCG

CCGACGAGGACTGTCTGCGGCTCAACGTCTACACGCCTGCCAACAATAGTTCCAAGAAGTTGCCGATAATATTCTTCATC

CACCCGGGCGGCTTCTTCGCCTTTAGCGGGCGTAGCGACATCTTCGGGCCGCACTATCTCCTAGATCGTGACATCGTGCT

CATCACCATCAACTACCGCCTCGCCTCGCTCGGTTTCCTCAGCACGGGCGACGCGGTGGCTCCCGGCAACAACGGCTTCA

AGGACCAAGTGTCGGCGCTGCGCTGGGTGCAGCGCAACGCCGCGGCCTTCGGCGGAGACCCGGGACAAGTGACGATCGCG

GGCTGCAGCGCCGGTTCTACCAGCCTCGGGCTGCATATGATATCGACTATGTCTAAAGGTCTATTCCACCGCGGTATCGG

AATGTCTGCGTCCCCGATCGGCAAGGAGCCACTGCCGTCGCATCAGTTCGAGCTGGCACAGAAGCAGGCGAGCATCCTCA

ACTGCCCGACCAACACCTCGCGCGCCATCGTCGACTGCCTCAAGACTAAAACCTGGCAGGAGATAGGGGACTCGCTGCCT

GGATTTTGGAACCAAGGCAACACTCAGTCAGACGCAATCGGTTTCTGGAAGCCAGTGATCGAGCCAGACTTGGGGCAGCC

GCGCTTCTTGACCGTCCAGCCGGATGAGGCGGTGCGCTCGCGCAGCATCCACGCCGTGCCGCTGCTCATCAGCCAGACTG

AAGACGAGTTCTACTGGAAGGCTTTCGATGTGACCAGAAACAAGACGGTCCGGGACCGCGTGAACGCGGAGTGGGAGACT

GTTGCACCGGACTCGTTCGATATGCCCAAGTTGCCCAAAGACAAAGCGTCTGCCGCCAGCCGCCGCCTCAAGGAGGCGTA

CCTGGGAGGGAAAGATGTGGAGGATACTCCGGAGAGCGCAAAAGCCTTGGGAGAGATTTATGGAGACAGTTGGGTTGGAC

TCAACACGCATAGGATGGCCAACCTGATGTGCCGGCACTCGCCTAAGCCCGTTTGGTACAGCATGTTCAGCTACATCGGT

AACAACAGCTATTTCGAGGACCCACTCACCGGGAAACCCGCTGGCGCAGCACATCATGACGACCTGATCTACTTGTTCTC

AATGAACTACTTCCGCAAGCCGATCGCGGCGCAGTCGCCGCCGCCGCCCGCGCCGCAAGACTCGACAATGGTGGACCGCA

TGACCGCCATCTGGTACACCTTCGCTAAATACGGAGACCCCAACCCCCGCCCCGGCGAGCTGCCGGAGCTGGCGACGCTC

TCCTGGCCGGCCATGAAGCCGGGAGACAGGAAGTACCTTCGCGTGGACAAGGAGTTCTCCGTCCACGAGAAGCTGCACGA

GACGAGGCTGCAAGTGTGGGAGGAGCTCTACCCCATCGTGTATTAG

>Cher_CXE16.1

ATGGCGAAAGTCAAAGTAAATGACGGTGTTTTGGAAGGAGAAATCCTAGATAATGTGCTCGGAGGGAAGTACTACAGTTT

CAAGGGGGTTCCTTACGCTGCTCCTCCGGTCGGAGACTTTCGATTTAAGGCCCCTCAACCAGTTAAACCTTGGGATGGAG

TCCGCAGCGCCACTGCCCACGGACCAATCTGCCACCAGTTTGACATGTTTACTCACACCATTGACATTGGCAGTGAGGAC

TGCCTGTACCTAAACGTGTACACCCCAACATTGACACCTGAAAAGCCTCTGCCTGTCATGTTCTGGATACATGGAGGTGG

TTTCATGAGTGGCAGCGGCAACAGCAACACCTACGGACCGGATTTCCTCGTCGCCCAGGATGTAGTCCTGGTCACCATTA

ACTACAGACTAGAGGTTCTCGGGTTCCTATGCCTGGATACAGAGGATGTTCCAGGAAATGCAGGCATGAAGGACCAAGTA

GCAGCCCTTAGATGGGTACAAAAAAATATAGGACATTTTGGTGGTGATGCTGACAATGTCACTATCTTTGGTGAAAGCGC

CGGCGGAGCGTCGGTTGCTTACCATTTGGTATCGCCCATGACGAAAGGACTATTTAAAAGGGGTATACTTCAGAGTGGTG

TGAATTTATGCCATTGGGCTCGGAACTTCGAACCGCGCCTGAGAGCTGAAGCGATTGCGAGATCACTTGGCAAGGACACT

ACAGATGATAAAGAACTGTATGAGTTTTTCAAATCACAACCTATTGAAAAAATAATAGCAATAGCCGCGCCCCTAACAGT

TGAAGAAGAAGCTGAAATACGTATAAATATAACATTAAATGTAGCTGATGAGAAACAGTTTGGTGGCAATGAGAGGTACT

TTTATGGTGATGTTTATGAACGGCTTCGTAGCGGCGTTCACGAGGGCGTTGAAATCATGATAGGATATACTACCCATGAA

GGGACTATTCAATTCAATAATTTGCCAGAAGAGGGTACTTTCGATAAACAGAAAAAGTTTTTACAAAGCTTTGTACCGAT

AGACTTGCTGCTGCAATGTTCAGTGAAAGACCAGCTGGCAATTGGCAAAAAGGTCAAAGAACACTATTTTGGAAACAAAC

AAGTGGGAACTGATACTGTTGACCCACTTATAAAATACTATACATTGCAGCTATTGGAGTATGATATTTTTCAATATGCC

AAAACCTGTGTACAGTCGAATGCCAATCCATTGTTCTTTTATCAGTTTGATGTTGAAACAGAAAGAAACATATATGCCGA

CATGTTTAAAGCCAGACCCTTAATAAAAGGGAAGAATGTCTGCCATTGCGACGATTTGATGTATTTGTTTGATTATGAAA

AGGTACCTGTAAACCTGAATTCTGAAAGCCACAAAATGATTAAAAATGTCACAAAATTATGGACTGACTTCGCCAAATTC

GGAGACCCGACTCCGGATGGTAAACTGCGCGTGCGCTGGCCGGCGTTCGACCTGCAGCGGCAGCAGTACCTCGTCATTGG

GAACGAGCTGACCGTCGCCGCATTCCCCGACAAGGAGGAGTTACAATTCTGGGAGGAACTCCACCAACAATACTTGGAGA

AGTAG

>Cher_CXE16.2

ATGGCGCAAGTGTCCATATGTGACGGTGTTCTGGAAGGAGAAATCCTGGATAATGTACTCGGGGGAAAGTACTACAGTTT

TAAAGGAGTGCCTTACGCCGCCCCTCCTGTCGGAGACCTGAGGTTTAAGGCGCCTCAACCAGTTAAACCCTGGGATGGAG

TCCGCAGCGCTACTGCCCACGGCCCAATCTGCCACCAGTTTAATGTGTTTACTAACACTATCGAGGGCAGTGAGGACTGC

CTGTACCTAAACGTGTACACACCGACATTAAAACCTACAAAACCCCTGCCCGTCATGTTCTGGGTACACTGTGGTGCTTT

CGTGAGTGGCAGTGGCAACAGCGATTCCTATGGACCAGATTTTCTCGTCAACCAAAATGTAGTCTTGGTCACTAGTAACT

ACAGACTAGAGGTTCTCGGATTCCTCTGCCTGGACACAGAAGACGTTCCAGGGAATGCAGGTATGAAAGACCAAGTTGCC

GCTCTTAGATGGGTACAAAAAAACATTGGACATTTTGGTGGTGACGCTGACAATGTCACTATCTTTGGAGATAGTGCCGG

CGGAGCGTCTGTTGCTTACCATTTGGTGTCACCTATGACCAAGGGATTGTTTAAACGGGCTATAGTTCAAAGTGGTCAGA

ACTTAAGTTGCTGGGCTCAGAACTTCGAACCACGCCTGAGAGCTGAAGCGATTGCGCGATCACTCGGCAAGGACACTACA

GATGATAAAGAACTGTATGAGTTTTTCAAATCACAACCTATTGAAAAAATAGTAGCAATGGCCGCGCCACTAACAGTTGA

AGAAGAAGCTGAAATACGTATAAATATAACATTAAATGTAGCTGATGAGAAACAGTTTGGTGACAATGAGAGGTACTTTT

ATGGTGATGTTTATGAACGGCTACGTAGCGGCGTTCACGAGGGCGTTGAAATCATGATAGGATATACTACCCATGAAGGG

ACTATTCAATTCAATAGTTTGCCAGAAGAGGGTACTTTCGATAAACAGAAAAAGTTCTTACAAAGCTTTGTACCGATAGA

CTTGCTGCTGCAATGTTCAGTGAAAGACCAGCTGGCAATTGGCAAAAAGGTCAAAGAACACTATTTTGGAAACAAACAAG

TGGGAACTGATACTGTTGACCCACTTATAAAATACTATACATTGCAGCTATTGGAGTATGATATTTTTCAATATACCAAA

ACCTGTGTACAGTCGAATGCCAATTCATTGTTCTTTTATCAGTTTGATGTTGAAACAGAAAGAAACATATATGCCGACAT

GTTTAAAGCCAGACCCTTAATAAAAGGGAAGAATGTCTGCCATTGCGACGATTTGATGTATTTGTTTGATTATGAAAAGG

TACCTGTAAACCTGAATTCTGAAAGCCACAAAATGATTAAAAATGTCACAAAATTATGGACTAACTTCGCCAAATTCGGA

GACCCGACTCCGGACGGTAAACTGCGCGTGCGCTGGCCCGCGTTCGACCTGCAGCGGCAGCAGTACCTCGTCATCGGCAA

CGAGCTGACCGTCGCAGCATTCCCCGACAAGGAGGAGTTACAATTCTGGGAGGAACTCCACCAACAATACTTGAAGAAGT

AG

>Cher_CXE16.3

ATGGCGCAAGTGTCCATATGTGACGGTGTTCTGGAAGGAGAAATCCTGGATAATGTACTCGGGGGAAAGTACTACAGTTT

TAAAGGAGTGCCTTACGCCGCCCCTCCTGTCGGAGACCTGAGGTTTAAGGCGCCTCAACCAGTTAAACCCTGGGATGGAG

TCCGCAGCGCTACTGCCCACGGCCCAATCTGCCACCAGTTTAATGTGTTTACTAACACTATCGAGGGCAGTGAGGACTGC

CTGTACCTAAACGTGTACACACCGACATTAAAACCTACAAAACCCCTGCCCGTCATGTTCTGGGTACACTGTGGTGCTTT

CGTGAGTGGCAGTGGCAACAGCGATTCCTATGGACCAGATTTTCTCGTCAACCAAAATGTAGTCTTGGTCACTAGTAACT

ACAGACTAGAGGTTCTCGGATTCCTCTGCCTGGACACAGAAGACGTTCCAGGGAATGCAGGTATGAAAGACCAAGTTGCC

GCTCTTAGATGGGTACAAAAAAACATTGGACATTTTGGTGGTGACGCTGACAATGTCACTATCTTTGGAGATAGTGCCGG

CGGAGCGTCTGTTGCTTACCATTTGGTGTCACCTATGACCAAGGGATTGTTTAAACGGGCTATAGTTCAAAGTGGTCAGA

ACTTAAGTTGCTGGGCTCAGAACTTCGAACCACGCCTGAGAGCTGAAGCGATTGCGCGATCACTCGGCAAGGACACTACA

GATGATAAAGAACTGTATGAGTTTTTTAAGTCTCAAGCACTTGATAAATTAGTAGCCATTACTGCACCACTGACAGTTGA

GGAAGAACCTGGAATACGTGTAAATCTAATTTTAACTGTCACTGATGAGAAACTGTTTGGTGACAATGAGAGATACTTTT

ACGGTGACACTTCCGAAAGATTGCATAACGGCATTCATGAGGGAGTTGAAGTCATGATGGGATTTAATACACACGAAGGC

ACAATTCAATTTCTTTATTTGAACGAAGAGGGCCCTTTCGATAAATATAACAAGTACTTACAAAGCTTTGTGCCGATAGA

GTTGCTGCTGCAATGTTCAGTGAAAGAGCAGCTGGCAATTGGCAAAAAGGTCAAAGAACACTATTTCGGAAATAAAAAAG

TGGCAATCGATACTATTGACCCATTCATAAAATTCCATACAATGCAGTTAATGGAGTATGATATTTTGAAATATGCCAAA

ACCTTTGTGCAGTCGAATGCCAATCCATTATTCTTTTATCATTTCGACGTTGAAACAGAAAGAAACATATTTACCGATGC

TTTTAAAGCCAGACGCTTCATAAAAGGGAGGAATGTCAGCCATAGCGACGATTTGATGTATTTGTTTAATAATGAACAGG

TGCCCGTAGACCTGAATGCAGAAAGCTACAAAATAATTAAAACTGTTACAAAACTATGGACCGACTTCGCCAAATTCGGT

GACCCGACTCCTGACGATACCCTGCGCGTGCGCTGGCCGGCGTTCGATCTGCAGCAGCAGCAGTACCTCGTGATTGGGAA

TGAGCTGACTGTGGCAGCATTCCCCGACAAGGAGGAGCAACAGTTCTGGGAGGAACTCCACCAACAGTACTCGAAGCAGA

AGTGA

>Cher_CXE16.4

ATGGCGCAAGTCCGAGTAAGTGACGGTGTTCTTGAAGGAGAAATCTTGAATAATGTACTCGGAGGAAAGTACTATAGTTT

TAAAGGAGTGCCTTACGCTGCCCCTCCTGTCGGTGACCTGAGGTTTAAGGCACCTCAGCCAGTTAAACCTTGGGATGGAA

TCCGGAGCGCCACTGACCATGGACCAATTTGCTGTCAGCACGATATATTTTTTAGCACTGTCGAAAGTGGCAGTGAAGAC

TGTCTTTATCTAAACGTGTACACACCAACACTGAAACCCAAAATACCTTTGGCTGTTATGTTCTGGATACATGGTGGTGG

ATTTTTAAGTGGCAGTGGCAACAGCGAAATCTTTGGACCAGATTTTCTCGTAGAACAGAACGTAATCCTTGTCACCATTA

ATTACAGACTAGAAGTTCTCGGATTTTTGTGCCTGGATACAGAAGACGTTCCAGGAAATGCTGGTATGAAAGACCAAGTG

GCTGCTCTTAGATGGGTACAAAAAAACATAGGACATTTTGGCGGTGATACTAACAATGTCACTATCTTTGGAGAAAGTGC

CGGTGGGGCATGCGTTGCTTACCACCTGGTGTCACCTATGACAAAAGGGCTATTTAAACGGGCTATACTTCAAAGTGGTC

AGAATTTAAGTCATTGGTCTCGGAACTTTGAACCGCGTCTAAGAGCTGAAGCGATTGCGCGATCACTCGGCAAACACACT

ACTGATGATAAAGAACTGTATGAATTCTTCAAATCTCAACCAGTTAAAAACTTAGTAGCCATTAACGCACCACTGACAGT

TGAGGAAGAACCTGGAATACGTGTAAATCTAATTTTAACTGTCACTGATGAGAAACTGTTTGGTGACAATGAGAGATACT

TTTACGGTGACACTTCCGAAAGATTGCATAACGGCATTCATGAGGGAGTTGAAGTCATGATGGGATTTAATACACACGAA

GGCACAATTCAATTTCTTTATTTGAACGAAGAGGGCCCTTTCGATAAATATAACAAGTACTTACAAAGCTTTGTGCCGAT

AGAGTTGCTGCTGCAATGTTCAGTGAAAGAGCAGCTGGCAATTGGCAAAAAGGTCAAAGAACACTATTTTGGAAACAAAC

AAGTGGGAACTGATACTGTTGACCCACTTATAAAATACTATACATTGCAGCTATTGGAGTATGATATTTTTCAATATACC

AAAACCTGTGTACAGTCGAATGCCAATTCATTGTTCTTTTATCAGTTTGATGTTGAAACAGAAAGAAACATATATGCCGA

CATGTTTAAAGCCAGACCCTTAATAAAAGGGAAGAATGTCTGCCATTGCGACGATTTGATGTATTTGTTTGATTATGAAA

AGGTACCTGTAAACCTGAATTCTGAAAGCCACAAAATGATTAAAAATGTCACAAAATTATGGACTAACTTCGCCAAATTC

GGAGACCCGACTCCGGACGGTAAACTGCGCGTGCGCTGGCCCGCGTTCGACCTGCAGCGGCAGCAGTACCTCGTCATCGG

CAACGAGCTGACCGTCGCAGCATTCCCCGACAAGGAGGAGTTACAATTCTGGGAGGAACTCCACCAACAATACTTGAAGA

AGTAG

>Cher_CXE16.5

ATGGCGCAAGTCCGAGTAAGTGACGGTGTTCTTGAAGGAGAAATCTTGAATAATGTACTCGGAGGAAAGTACTATAGTTT

TAAAGGAGTGCCTTACGCTGCCCCTCCTGTCGGTGACCTGAGGTTTAAGGCACCTCAGCCAGTTAAACCTTGGGATGGAA

TCCGGAGCGCCACTGACCATGGACCAATTTGCTGTCAGCACGATATATTTTTTAGCACTGTCGAAAGTGGCAGTGAAGAC

TGTCTTTATCTAAACGTGTACACACCAACACTGAAACCCAAAATACCTTTGGCTGTTATGTTCTGGATACATGGTGGTGG

ATTTTTAAGTGGCAGTGGCAACAGCGAAATCTTTGGACCAGATTTTCTCGTAGAACAGAACGTAATCCTTGTCACCATTA

ATTACAGACTAGAAGTTCTCGGATTTTTGTGCCTGGATACAGAAGACGTTCCAGGAAATGCTGGTATGAAAGACCAAGTG

GCTGCTCTTAGATGGGTACAAAAAAACATAGGACATTTTGGCGGTGATACTAACAATGTCACTATCTTTGGAGAAAGTGC

CGGTGGGGCATGCGTTGCTTACCACCTGGTGTCACCTATGACAAAAGGGCTATTTAAACGGGCTATACTTCAAAGTGGTC

AGAATTTAAGTCATTGGTCTCGGAACTTTGAACCGCGTCTAAGAGCTGAAGCGATTGCGCGATCACTCGGCAAAGACACT

ACTGATGATAAAGAACTGTATGAATTCTTCAAATCTCAACCAGTTAAAAACTTAGTAGCCATTAACGCACCACTGACAGT

TGAGGAAGAAGCTGGAATATTTATGCATTTAAAATTAAACGTCACTGATGAGAAACTGTTTGGTGACAATGAGAGATACT

TTTATGGTGACGTTTACGAAAGATTGCGTAACGGCATTCATGAAGGAGTTGAAGTCATGATGGGATACACGACACACGAA

GGCCTTCTTACGTTCAATTATTTGAACATAGAGGGTACTTTCGATATATTTAACGACTACTTACAGTGTTTTGTGCCTGT

AGAGTTATTGTTGCAATGTTCTGTGAAAGACCAGCTGGCCATTGGCAAAAAAGTCAAAGAATATTACTTCGGAAACAAAA

AAGTGTCTATTGATACTGTCAACTCGTTCATCAAATGCATTTCAATGCAGTTCTTACAGTATGATAGTTTGCAATACGCG

AAAAATTTCGTACAGACGAATCCTAATCGATTGTTTTTATATCAATTTGACATTGATACTGAGAGAAACAAATATGCAGA

TCACTTTAAAGCCAGACCTTTTATAAAAGGGAAAAGTGTCTGTCACTGCGACGACTTGGTGTATTTGTTCAATGATGAAG

AAGTACCTATAAACCTCAATGCTGACAGTTACGAAATGATAAAAAATGTGACGAAATTGTGGACTGACTTCGCCAAATTC

GGTAACCCGACTCCTGACGGTAGCCTAGGTGTGGATTGGCCGGCGTTCGACCTAGAACATCAGAAGTTTCTCATCATCGG

GAATCAATTGACAATCGCAGATTTTCCCGAAAAAGATGAGTTAGAATTCTGGGAGCAACTACACGAACAGTACCTACCTA

AGTAG

>Cher_CXE16.6

ATGGCGCAAGTCCGAGTAAGTGACGGTGTTCTTGAAGGAGAAATCTTGAATAATGTACTCGGAGGAAAGTACTATAGTTT

TAAAGGAGTGCCTTACGCTGCCCCTCCTGTCGGTGACCTGAGGTTTAAGGCACCTCAGCCAGTTAAACCTTGGGATGGAA

TCCGGAGCGCCACTGACCATGGACCAATTTGCTGTCAGCACGATATATTTTTTAGCACTGTCGAAAGTGGCAGTGAAGAC

TGTCTTTATCTAAACGTGTACACACCAACACTGAAACCCAAAATACCTTTGGCTGTTATGTTCTGGATACATGGTGGTGG

ATTTTTAAGTGGCAGTGGCAACAGCGAAATCTTTGGACCAGATTTTCTCGTAGAACAGAACGTAATCCTTGTCACCATTA

ATTACAGACTAGAAGTTCTCGGATTTTTGTGCCTGGATACAGAAGACGTTCCAGGAAATGCTGGTATGAAAGACCAAGTG

GCTGCTCTTAGATGGGTACAAAAAAACATAGGACATTTTGGCGGTGATACTAACAATGTCACTATCTTTGGAGAAAGTGC

CGGTGGGGCATGCGTTGCTTACCACCTGGTGTCACCTATGACAAAAGGGCTATTTAAACGGGCTATACTTCAAAGTGGTC

AGAATTTAAGTCATTGGTCTCGGAACTTTGAACCGCGTCTAAGAGCTGAAGCGATTGCGCGATCACTCGGCAAAGACACT

ACTGATGATAAAGAACTGTATGAATTCTTCAAATCTCAACCAGTTAAAAACTTAGTAGCCATTAACGCACCACTGACAGT

TGAGGAAGAAGCTGGAATATTTATGCATTTAAAATTAAACGTCACTGATGAGAAACTGTTTGGTGACAATGAGAGATACT

TTTACGGTGACGTTTACGAAAGATTGCGTCACGGCATTCATGAGGGAATTGAAGTCATGATGGGATACACCACACACGAA

GGCCTGCTTAGATTCAATTATTTGAACAAAAAGGGCACTTACGATCAATTTAACAAGTACTTACAGTGTTTTGTGCCTGT

AGAGTTATTGTTGCAATGTTCTGTGAAAGACCAGCTGGCCATTGGCAAAAAAGTCAAAGAATATTACTTCGGAAACAAAA

AAGTGTCTATTGATACTGTCAACTCGTTCATCAAATGCATTTCAATGCAGTTCTTACAGTATGATAGTTTGCAATACGCA

AAAACATTCGTACAGACGAATCCTAATAGATTGTTTTTATATCAATTTGACATTGACACTGAGAGAAACAAATATGCTGA

TCACTTTAAAGCCAGACCTTTTATAAAAGGGAAAAGTGTCTGTCACTGCGACGACTTGGTGTATTTGTTCAATGATGAAG

AAGTACCTATAAACCTCAATGCAGACAGTTACGAAATGATCAAAAATGTGACGAAATTGTGGACTGACTTCGCCAAATTC

GGTAACCCGACTCCTGACGGTAGCCTAGGTGTGGATTGGCCGGCGTTTGACCTAAAACATCAGAAGTTTCTCATCATTGG

GAATCGATTGACGGTCGCAGACTTCCCCGAAAAAGACGAGTTAGAATTCTGGGAGCAACTACACCAACAGTATCTACCTA

AGTAG

>Cher_CXE17

ATGAAGTATGCAAAGTACGTGGTACTATTTACGTTGTTCGCCTTGAACCTGGTCGACCAACCGGCGCCAGAGCTCGAAAT

TGAACAAGGCAAATTGAGCGGTAAAGTCAGCGATGATGGGTCGTATTTTGAATACGTCGGGATACCATATGCTTCGACTA

ACCACAGCACGAGATTTCAGGCACCCGGTCCTCCGCCATCATGGGAAGGCGTGTTCAAAGCAACAGACCACATTTACATG

TGTCCTCAAAACTCCAGGACTGGGACAGTCGGCAGAGAAGACTGCCTCACCATCAATGTTTACGTCCCGACCATGGCCAA

TGGCCCCTTACCCGTCATGGTCTACATTCACGGAGGAGCATTTGCCCTTGGAAGTGGAGGAAAATTCATGTATGGACCTG

ACTTTCTTGTGAAACATGATGTTATTGTAGTCACCTTCAACTATAGACTAGGAGCTCTCGGATTCATGTGCTTGGGAATC

AAAGAAGCACCAGGTAACGCTGGCCTTAAAGACCAATTGGCAGCTCTGAGATGGGTTAAGAAGAATATTGCTGCGTTTGG

TGGTGACCCTGATAATATCACTGCGTTTGGCGAAAGCGCTGGAGCGGCGTCGCTGTCTTTGCTAGTGGCTAGTGATACAG

CGAATGGCTTATTTAAAAGAGCTATAATTCAAAGTGGGTCGTCTATAGGTAACTGGGCGATTAACAGAAAGCCAGTTTGG

GTGTATAGCCTTATTGCGAAGCAGCTAGGATATAAAACAGAAGATCCACATGAACTTTACAATATTTTTAAAGATCTACC

AGTCAAAGATTTCCTTCGCACAAGACCAAGAAAGCCTCTTAGCAAGTTTTTTGATACCCAGTTATTGCACCTGCCTTGTG

TTGAGAAATCTTTCCCTGGTATAGAGCCCATTATCACAGAGTTACCGTATGACATTTTGCAAAGAAAAACTATAAATATA

CCGTTAATGTACGGTTCAAACAGCAAAGAAGGCTTATTCTTAATAAGCGAAGAGACGCCAGAAACAGTTGATGAAAGAAA

CGGCCGCTACCTGTTTGCCTCTGATTTAGAATTCAAAGATGAAGATACAGCAGACAGAGAAGCTGAAACAGTAAAAGAGT

TTTACTTCGGCAAGGAAACTATTGATATGACTAAAATACTTAACATGTCAGAACTTTATACACATTTGTACTTTGAAATG

CCGGCATTGATGGAAGCTGAAGTTATAGTCAGTCGAAGTGATAAACCATTATATTATTACTACTTCGACTATGCTGGAAG

CAGAAACGTACTGAAATACATATCGGGATATGGTGGAAGTGAAGGAGCATGTCACGGGGATGATTTGTTCTATTTGTTTA

AACAAGATTTTTTGCGATTCTGGATCAATAAAGAAGATCGGAGAATAATTGACAGCATGACTGAGATGTGGACCAATTTT

GCCAAATATGGGGTTCCAACACCAGAGTCATCAACCAATCCACCTCGTTGGACACCAAGCACAAAAGAAGAGACAAAGCT

ACTTTATATAGGAAAAGAATTGAAAATGGGCCCGATACCCAATCCCAAAGCGTATGAAATGTGGAAACGGGTGTTTACCA

CGTATCGTAGGAAGTCTAAGTAA

>Cher_CXE19

ATGGTGAACCGAGATCTAAGAGTGTGGGTGAAGCAAGGGTGGTTAGAAGGAGAAAAAAAAGAGTTGATCACAGGAAATGG

GACCTACTACAGCTTCAAAGGCATACCGTATGCGGCGCCACCAATCGGAAGATGGCGATTTAAGGCCCCCCAGCCGCCTC

TCTCTTGGACAGGAATCAAGAAAGCAACTGAACACGGACCAGTATGTCCTCAATATGACTTGCTGAACAACCAGATGTGC

CCAGGCATAAGTGAAGACTGTCTATACTTAAACGTTTACACTAGAAATATAAAACTAGAAAAACCTCTTGCAGTCATGGT

TTTCATTCATGGAGGAGGCTTCAAATTGGGCTCCGGAAACGAAGACAATTACGGTCCAGATTTCCTAGTTGAACAAGACG

TAGTCTTAGTAACGATTAACTATAGGCTAGATGTACTAGGATTTCTTTGTTTAGACACGAGAGATGTCCCAGGTAATGCA

GGAATGAAGGACCAAGTGGCAGCATTGAAATGGGTCAAAGATAATATCCGGCAATTTGGTGGTGATCCTCGTAACATTAC

AGTTTTTGGTGAAAGTGCTGGTGGAGCTTCTGTATCACTTCATGTCATATCACCACTAGCTAAAGGATTGTTTAATAGAG

CACTTACAATGAGCGGAACTGCGTTTAGTGAGTGGGCAATGACAGAAGATCCCTTGAAACGGGCCTTTGCTCTCGGAAGA

CAATTAGGAATAGATACTGAAGACCCCGTTGAACTGCATAACTTCCTACAGAACGTCCCAGTAGAGAGCCTTGTAAACAC

TAAGACAGCCCTTAATGACTTTGAAGAAAAAACTAATTACATGTGGAGAGTAAATCAGTTTGGCCCAACTGTGGAAAAGG

CATTAGGACAAGAGCCATTCTTAGCTGAGGATCCAATGAGTCTAATGCTGAAGAGAGGTGTCGGCCAAGACGTAGATTTT

ATGATAGGTTATACAAACGAAGAAGGTATATTAGGCATATCAACGTTTGCAGAACTTTGGGTTAAATCATTCAGCGAATC

TCCAGAGCTTTTTCTGCATGCGCCCATTGTCACTAAAAGTAACAAAGTTACAGAGCTGTCTCAAAGAGCTTTTGATCGCT

ACTTTGGAGACAAAGATTTAAATGAAAAAGGTGCCAGAGGAATGGTGACATATTTGTCAGAAAAGGCAATTTATGCGATC

TTACTGTACCTCAATCTTCTGGCAAAATCTGGTAAATCGAAGAGATATCTCTACAGATTTTGTTGCGTATCCGAAAGGAA

TATTTATGGTAATACTGGTCAGGAATACGGTATTAAAGGAGCTAGCCATATGGATGACATGGTGTATCTGTTTGATCCAA

AACAGATGAATTTACAATTGGACACAAGCAGCAAAGCTTATGAAATGATTAAACTGTCGTGTAAAGTTTTTACCAATTTT

GCAAAGTTTGGTAACCCAACTCCTGATTCTTCGCTCGGCATCAAATGGCCTGTCTACAGCGACTACTCGAGGAGTCACGT

GGATATCGGTGAAGAACTGACCCTGGGTGCAAATCTTGACGAAGACGTGCTCGCCTTCTGGCGAAAGATATTCTTAGACG

CAGAATTGCCTAAATGA

>Cher_CXE21

ATGAAGACTGTTGTGGTGATATGTCTGGTGTTCGCGAGCGCGGCGTCCGCGCAGAGCGAGGCGCGCGAGTCGCGCACCGT

GCAGACCGCGCAGGGGCCCGTGCGCGGGTACAAGACTGATGACGTTTTTCAATTCCTTGGAGTACCTTACGCTACGGCGC

CGACTGGTGAAAACCGGTTTAAAGCACCACTTCCAGGACCTATTTGGATGAACCCTTTAGAAGCGGTTAATAATAAAATA

ATCTGTCCACAAGGCACAAGCGTATTTTTTAGCTTTTCTTCATTCTCTATGCAAGAAGACTGCCTTGTTGCAAATATTTA

TATGCCGGACACGGAAGATACTAACCTGCCAGTAATGGTTTATGTGCACGGGGGCGCGTATCAAATGGGATGGGGTGAAA

TGATCACACCAAAATCTTTGGTACAAAGCAAAAAAATTGTGGCGGTCTCTTTCAACTACCGACTCGGTATCCATGGTTTC

CTGTGTCTGGGAACAAATGACGCTCCCGGCAACGCGGGCATGAAGGACCAAGTCGCGCTGCTGCGCTGGGTGCAGAAAAA

CATTGCCAACTTTGGTGGAAACCCTAAAGACGTCACAATTGTTGGCAGCAGTGCAGGATCATCTTCAGTTGATCTCCTAA

TGCTTTCCGAAGCTACAAAAGGTCTCTACAGTAAGGTTATTCCTGAAAGTTGTGCTAGTGTTGGAGTTTGGAGTGTTCAG

CTCGATCCGGTGCAAAACGCAAAAGATTTCGCGGGAAAGAACAATTTTACTGAAGTAAACGATATCCATGCGTTAGAACA

ATTTTATAAAACTGCATCATTTGAACTACTTACCTCCGACGTATTTATGGCTCAAAAAGATTCGACTTTTGTATTCTCCC

CATGCGTAGAAAACAATATAGGGGAAGAAACGTTTCTTGAAGATAATCCAGTAAATATACTGAAAGAAGGAAAATACAGA

AAAGTTCCCGTTCTCTATGGATTTGCAAATATGGAGGGTCTGTTTAGGATGGATAGATTTGGAGAATGGAAGGACCAAAT

GAACGAGCATTTCTCAGACTTTCTGCCAGCCGACTTAACGTTCAAATCTGAAATAGAACGTTCTGAAGTTGCACAAATAG

TAAAGGAATTTTATTTCGGTGATAAATCTGTAGGAGAAGAAACAGTTCAAGCTTACATCGACTATTTCAGTGATGTCATG

TTTGCTTACCCGCATCTTAGATCCACACAGCTACAAGTGGCAGCTGGTAGTGACTCCATATACTTGTATGAGTATTCCTA

TTATACACCCCCACCGCAATTGGAGGATATGCCACCTGTACCAGAATTCATGAAACAAATCCTTGGAGCTAATCACTGCG

CTCAATCCAGGGCAGTCTACGAAAGTATTATGTTTCCAGATTTTGAAATCGACGAAGAACACCGAAAGAACAGAGATACA

ACGCAAGAGATATGGTTAAATTTTATAACAACTGGTAAACCAGTTCCCGAAGTGTCAAAGCTTCCAGCGTGGCCCCCAGT

CGGTGCCAACAGGTCCCCGTACATGGACCTCGGGGAGGAGATGACACTCAAGGGTCCCCTCCTGGAGAAGCGAGCTCGCT

TCTGGGACGACATATATGAGCGATACTACAGGTCGCCAGTGGCGCCTACGTTGCATTCCCCAGCAAGAGATGAATTGTAG

>Cher_CXE22.1

ATGAAGACTGTTGTGCTGGTGTGTCTGGCAGTCGCGTGCGCGGCGTCCGCGCAGAGCGAGGCGCGCGAGTCGCGCACCGT

GCAGACCGCGCAGGGGCCCGTGCGCGGGTACAAGAATGACGAGATCTTCGAATTTTACGGAATTCCTTACGCCACCGCTC

CTACTGGAAAAGACCGGTTTAAAGCGCCGCTGCCAGGTCCTGTTTGGATGAGTCCTTTAGAAGCAGTCAATGATAAAATT

ATGTGTCTACAAGGCCAAATGCCAGGTTTTCCTATGAACGGATTAACCATGCAAGAAAACTGTCTGATCGCAAATATCTT

AATGCCTGATACTGAAGAAACAAATTTGGCAGTTGTTGTTTATGTCCACGGGGGTGGATATCAATTGGGAGCCGGAAATA

TGCTTCAACAGAAAAGTCTAGTAAAGAGTAAAAAGGTAGTAGCAGTATCCTTCAACTACCGCCTTGGTCTCCATGGGTTC

CTGTGTCTTGGCACAGAAGGTGCTCCTGGCAATGCGGGTATGAAAGATCAAGTCGCCCTTCTGCGATGGGTAAAGAAAAA

CATTGCCAACTTTGGTGGAAACCCCGACGACGTTACAATCGGCGGCTATAGCGCAGGGTCCTCGGCAGTGGACCTCCTCA

TGCTTTCTGAATCAACCAAAGGTCTTTTCAATAAGGTCATACCTGAAAGCGGTGCCAACGTTGCTGTTTGGAGTATTCAA

ATTGATCCTATACAAAACGCGAAAGAATTTGCTAAGACCAACAATTTCACAGCAGTAGACGACATCAACGCCTTGGAAGA

ATTTTACAAAACTGCGCCATTCGACTTACTTATGTCGGATGTATTTCTGTATAGACTGGACTCAACGTTTTTGGCTTCTC

CGTGTGTAGAGCGCAACATTGGTCAAGAAACGTTTTTAGAAGATAACCCAGTCAATAATATTCTGAAGCAAGGTAAATAC

AGAAAAGTACCTGTCTTATATGGGTTTGCAAATATGGAGGGTCTATTTAGAATGGATAAATTTGAAGAATGGAAAGTCAA

AATGAATAAACAATTTGCAGACTTCTTACCAGCTGATTTAACTTTTAAATCTGAAAGCGAACGTTCTAAAGTTGCGCAAG

AAATAAAAGAATTCTATTTTGGTGAAAAACCCGTAGGAGAAGAAACTGTTCAAGGTTACATCGACTATTTCACTGACGTT

ATATTTGCTTATCCGCATCTCAGATCCGTACAATTACAAGTAGCAGCTGGCAGTGACTCCACATACTTGTATGAGCATTC

TTTCTACTCACCACCTCCTGAAATCGAAGGCATGCCGCCCATACCGGAATACATAAAGAAAATTCGAGGAGCCAACCACT

GTGCTCAGTCATTTGCGGTACACGAGGTCACAATACCATTAATTGGCTTAGACAATACTACCGAGTACTTGAATTTCCAG

GAAAAATTGCGAGAAATTTGGTTAAACTTCGTACTCACCGGAAAACCGGTTCCCGAAGGGTCAAAACTGCCGGCGTGGCC

CCCGGTCGGTGCCAACAGGTCCCCGTACATGGACCTCGGGGAGGAGATCAAACTCAAGGGTTCTCTCCTGGAGAAGCGAG

CTCGCTTCTGGGACGACATCTATGAGCGATACTATAGGTCGCCGGTGCCTCCGTCGCCTACTCAAACAAGAGACGAATTG

TAA

>Cher_CXE22.2

ATGAAGACTGTTGTGCTGGTGTGTCTGGCGGTCGCGTGCGCGGCATCCGCGCAGAGCGAGGCGCGCGAGTCGCGCACCGT

GCAGACCGCGCAGGGGCCCGTGCGCGGGTACAAGAATGAGGGGATCTTTGAATTTTACGGAATTCCCTACGCTACCACAC

CGACTGGACCACACAGATTTAAGGCGCCGCTTCCAGGACCGATATGGATGAGTCCTTTAGAAGCAGTCAAGGATAAAATA

ATTNCGCCGCAAGGCAGCACGGGCAATTTTCCTATGACGGGAGTGCAGGTGCAAGAAGACTGTCTTATTGCGAATGTTTT

AATGCCTGATACAGAAGAGACAAACCTTCCTGTAGTTGTTTATGTCCACGGTGGTGCTTACCAATTGGGATACGGAAATA

TGCTTCTACAGAAGAGTTTTGTGCAAACTAAAAAAGTAGTGGCAGTAACCTTTAACTACCGCCTTGGTCTTCATGGGTTC

CTGTGTCTTGGCACAGAAAATGCTCCTGGCAACGCGGGTATGAAAGATCAAGTCGCGCTCCTGCGATGGGTAAAGAAGAA

CATTGCCAATTTTGGTGGAAACCCCGACGATGTGACAATCGGCGGCTATAGCGCAGGGTCCTCGGCAGTGGACCTCCTCA

TGCTTTCCGAATCAACCAAAGGTCTTTTCAATAAGGTCATACCCGAAAGCGGCGCCAACGTTGCGGTTTGGAGTATTCAG

CTTGATCCTATAGAAAACGCGAAAGAATTTGCTAGAAACAACAATTTCACAGCAGTAGACGACATCAATGCCTTAGAAGA

ATTTTACAAAACTGCGTCATTCGACTTGCTTATGTCGGACATGTTTATGCATAGACCGGACACAACGTTTTTGGCTTCTC

CATGTGTAGAGCGCAACATTGGTCAAGAAACGTTTTTAGAAGATAACCCAGTAAATATTCTGAAGCAAGGAAAATACAGA

AAAGTCCCTGTTTTGTATGGGTTTGCTAATATGGAAGGTCTATTCAGAATTGATGTATTTGAGCAATGGAAAGTCAAAAT

GAATGAACAATTTTCTGACTTTTTACCGGCTGATTTAACGTTTAAGTCTGAAAGCGAACGTTCTAAAGTTGCTCAAGAGG

TAAAGGAATTCTATTTTGGAGATAAACCCGTAGGAGAAGAAACTGTTCAAGGTTACATCGACTATTTCACTGACGTTATA

TTTGCTTATCCGCATCTCAGATCCATACAATTACAACTGGCAGCTGGCAGCGACTCTATATACCTGTATGAGTATTCCTT

CTACTCACCACCTCCTGTAATGGAAGGCATGCCACCTTTACCCGAATACCTTCAAAAGACTCGAGGAGCCAACCACTGTG

CTCAGTCATTTGCGGTACACGACTGCTCAATACCATTCCTTGCCTCTGATAATAGAGACGAATTCTTAAATTTCCAGGAG

AAGTTGCGAGAAGTGTGGTTAAACTTCATAATTACTGGGAAACCGGTTCCTGAAGGGTCAAAGCTGCCAGCGTGGCCCCC

GGTCGGTGCCAACAGGTCTCCGTACATGGACCTCGGGGAGGAGATCAAACTCAAGGGTTCTCTCCTGGAGGAGCGAGCTC

GCTTCTGGGACGACATCTACGAGCGATACTATAGGTCGCCGGTGCCTCCGTCGCCAACTCAAACAAGAGACGAATTGTAA

>Cher_CXE22.3

ATGAGTCCTTTAGAAGCAGTCAATGATAAAATTATGTGTCTACAAGGCCAAATGCCAGGTTTTCCTATGACGGGAGTGCA

GGTGCAAGAAGACTGTCTTATTGCGAATGTTTTAATGCCTGATACAGAAGAGACAAACCTTCCTGTAGTTGTTTATGTCC

ACGGTGGTGCTTACCAATTGGGATACGGAAATATGCTTCTACAGAAGAGTTTTGTGCAAACTAAAAAAGTAGTGGCAGTA

ACCTTTAACTACCGCCTTGGTCTTCATGGGTTCCTGTGTCTTGGCACAGAAGGTGCTCCTGGCAATGCGGGTATGAAAGA

TCAAGTCGCCCTTCTGCGATGGGTAAAGAAAAACATTGCCAACTTTGGTGGAAACCCCGACGACGTTACAATCGGCGGCT

ATAGCGCAGGGTCCTCGGCAGTGGACCTCCTCATGCTTTCCGAATCAACCAAAGGTCTTTTCAATAAGGTCATACCCGAA

AGCGGTGCCAACGTTGCTGTTTGGAGTATTCAAATTGATCCTATACAAAACGCGAAAGAATTTGCTAGAACCAACAATTT

CACAGCAGTAGACGACATCAACGCCTTGGAAGAATTTTACAAAACTGCGCCATTCGACTTACTTATGTCGGATGTATTTC

TGTATAGACTGGACTCAACGTTTTTGGCTTCTCCGTGTGTAGAGCGCAACATTGGTCAAGAAACGTTTTTAGAAGATAAC

CCAGTCAATAATATTCTGAAGCAAGGTAAATACAGAAAAGTACCTGTCTTATATGGGTTTGCAAATATGGAGGGTCTATT

TAGAATGGATAAATTTGAAGAATGGAAAGTCAAAATGAATAAACAATTTGCAGACTTCTTACCAGCTGATTTAACTTTTA

AATCTGAAAGCGAACGTTCTAAAGTTGCGCAAGAAATAAAAGAATTCTATTTTGGTGAAAAACCCGTAGGAGAAGAAACT

GTTCAAGGTTACATCGACTATTTCACTGACGTTATATTTGCTTATCCGCATCTCAGATCCGTACAATTACAAGTAGCAGC

TGGCAGTGACTCCACATACTTGTATGAGCATTCTTTCTACTCACCACCTCCTGAAATCGAAGGCATGCCGCCCATACCAG

AATACATAAAGAAAATTCGAGGAGCCAACCACTGTGCTCAGTCATTTGCGGTACACGAGGTCACAATACCATTAATTGGC

TTAGACAATACTACCGAGTACTTGAATTTCCAGGAAAAATTGCAAGAAATTTGGTTAAACTTCGTACTCACCGGAAAACC

GGTTCCCGAAGGGTCAAAACTGCCGGCGTGGCCCCCGGTCGGTGCCAACAGGTCCCCGTACATGGACCTCGGGGAGGAGA

TCAAACTCAAGGGTTCTCTCCTGGAGGAGCGAGCTCGCTTCTGGGACGACATCTACGAGCGATACTATAGGTCGCCGGTG

CCTCCGTCGCCAACTCAAACAAGAGACGAATTGTAA

>Cher_CXE24_PDE1

ATGTTAGCTGTCAAACTTCTTTTGTTTCTCGCGGTGGTGGCCTGTGCGCGGGCGCAGGCGTCCAAGCCGGTGGTGAGGGT

GACGCAGGGCATACTCCAAGGGTCCTGGAAGGTGTCGACTAAGGGAAGAACTTATGCCAGCTTTGAAGGGGTCCCTTATG

CAAGGCCACCTGTCGGAAAATATAGGTTTAGGGAGCCACAGCACTTAAAGCCATGGGCAGGAGTGTGGGACGCGACCAAA

ACACTCCCCAACTGCATGCAGTACGAACCATTCAATAAGAAGGTTGAAGGCTCAGAGAACTGTCTCTACGTGAACGTGCA

CACGCCGAAACTCAGCGCCGGCGCAGCCTTGCCCGTCGTGGCGTTCATCCATGGCGGCGCGTTCATGTACGGCGCTGGCT

CCCTGTACGACGCGGGACATCTCATGGACAGGGATGTGGTGGTGGTGACCATCAACTATAGATTGGGACCGCTAGGTTTC

CTCAGCACCGGAGACGAGTTCGCCCCAGGCAACGCTGGCCTGAAGGACCAGTCCTTCGCTCTCCAGTGGATCCAGAGCAA

CATCATGATGTTCGGCGGAGACCCTGATAGTGTCACCCTGACGGGGTGCTCCGCTGGCGGCGCCAGTGTCAACTACCACT

ACCTGTCGCCTATGTCTAAAGGCAAGTTCGCCCGCGGCATCGCCTTCAGCGGCGCTGCTTTCGCCTCCTGGGCGCAAGCG

GTCAAGCCGCTGCAGAACGCAAAGAGCCTAGCAGCCATCGTCGGGTGCCCCACCGGCGCCAGCAAAGAGCTGGTCGACTG

CCTCAAGTACCGACCGGCTGAGGTCGTGGTCGGCGCGCAGACTGAGATGTTTGAGTTCCCCTACCTCGAAATGTTCTCGC

CGTTCACGGCGACCGTGGAGCCAGCCGGTACCAGGGACGCATTCCTGACGCAGTACCCCTTCCACGTGGCGCAAGCTGGG

GGTATGCACAAGGTGCCCCTCATCGCCTCCGTCACCTCCGAGGAGGGGCTGTACCCTGCTGCTGTGTACCAAACGTCACC

TGACATTCTGCCTTACCTGGACGCCAACTGGGAGCAGCTGGCCAGCAATATCTTCGAGTACAACGACACTTTGGCCGTTA

ACCTGAGGGCCAATGTAGCGGCCAAGATCAAGCAGCACTACCTTGGAAACAAACCTGTCAGCCAGGAGACCTTCCCGCAG

TTAGTTCAGGCTCTCGGCGACCGTCTGTTCGGGGTTGACGTGGGCAAGCTGGCTGAGATCCACGCGCGCAAATCTGGCCA

GCCCACCTACTTGTACCGGTATTCGTTCCGCGGAGAGAAGAGCCTGTCCAACATGATGGCTCGAAACAATAACAACTATG

GTGTGAGCCACGCTGATGACGTTTTCCACATCTTCAAGTTCGACGTGCTGTCGTCCACGACGGCTGAGGATATGAAAATG

ACGGAAGCCCTTCTCGACATGATTTACAGCTATTCTACTACTGGCAACCCGAAGTTGACCAACGAGGCGCCAGCGTGGAC

GCCACTCACTCCTGGCTCCACCGAGCTGAATTACCTGGAGATAGCCTCGCCGAGCAGCATCGAGATGAGAACCAGCTCCG

ACTTCGGGCAGCGTAGCTTCTGGGGCAGCCTGGGGTTGGTGGAGAATGAGAATTACCGGGTTAGGGACGAGCTTTGA

>Cher_CXE24_PDE2

ATGTTAGCTGTCAAACTTCTTTTGTTTCTCGCGGTGGTGGCCTGTGCGCGGGCGCAGGCGTCCAAGCCGGTGGTGAGGGT

GACGCAGGGCATACTCCAAGGGTCCTGGAAGGTGTCGACTAAGGGAAGAACTTATGCCAGCTTTGAAGGGGTCCCTTATG

CAAGGCCACCTGTCGGAAAATATAGGTTTAGGGAGCCACAGCACTTAAAGCCATGGGCAGGAGTGTGGGACGCGACCAAA

ACACTCCCCAACTGCATGCAGTACGAACCATTCAATAAGAAGGTTGAAGGCTCAGAGAACTGTCTCTACGTGAACGTGCA

CACGCCGAAACTCAGCGCCGGCGCAGCCTTGCCCGTCGTGGCGTTCATCCATGGCGGCGCGTTCATGTACGGCGCTGGCT

CCCTGTACGACGCGGGACATCTCATGGACAGGGATGTGGTGGTGGTGACCATCAACTATAGATTGGGACCGCTAGGTTTC

CTCAGCACCGGAGACGAGTTCGCCCCAGGCAACGCTGGCCTGAAGGACCAGTCCTTCGCTCTCCAGTGGATCCAGAGCAA

CATCATGATGTTCGGCGGAGATCCTGACAGCGTCACGCTGACGGGGTGCTCCGCTGGAGGCGCCAGTGTCAACTACCACT

ACCTGTCGCCGATGTCTAAAGGCAAGTTCGCTCGCGGCATCGCCTTCAGCGGCGCTGCTTTGGCCTCGTGGTCACATGCG

GTCAAGCCGCTGCAGAATGCGAAGAGCCTCGCAGCCATTGTTGGGTGCCCCACCGGCGCCAGCAAAGAGCTGGTGGACTG

CCTCAAGTACCGACCGGCTGAGGTCGTGGTCGGCGCGCAGACTGAGTTGTTTGAGTTCCCCTACCTCGAAATGTTCACGC

CGTTCACGCCAACGGTGGAGCCAGCTGGTACCAGGGACGCATTCCTGACGCAGTACCCGTTCCGCGTGGCTCAAGCTGGG

GGCATGCACAAGGTGCCCCTCATCGCCTCCATCACCTCCGAGGAGGGGCTGTACCCTGCTGCTGTCTACCAAACATCACC

TGACATCCTGCCTTACCTGGACGCCAACTGGGAGCAGCTGGCCAGTAACATCTTCGAGTACAACGACACCTTGGCCGTTA

ACTTGAGGGCCAATGTAGCGGCCAAGATCAAGCAGCACTACCTTGGGAACAAACCTGTCAGCCAGGAGACCTTCCCGCAG

TTAGTTCAGGCTCTCGGCGACCGTCTGTTTGGGGTAGACGTGGGCAAGCTGGCTGAGATCCACGCGCGCAAGTCAGGCCA

GCCTACCTACTTGTACCGGTACTCGTTCCGCGGCGAGAAGAGTCTGTCCAACACGATGGCTCGAAACAATAACAACTATG

GTGTGAGCCACGCTGATGACGTGTTCCACATCTTCAAGTTTGACGTACTGTCGTCCACGAAGGCTGAGGATATGAAGATG

ACGGAAGCCCTTCTCGACATGATTTACAGCTATTCTACTACTGGCAACCCGAAGTTGACCAACGAGGCGCCAGCGTGGAC

ACCCCTAACTCCTGGCTCCACCGAGCTGAATTACCTGGAGATAGCCTCGCCCAGCAGCATCGAGATGAGAACCAGCTCCG

ATTTCGGGCAGCGTAGCTTCTGGGGCAGCCTGGGGTTGGTCGAGAATGAGAATTACCGGGTTAGGGACGAGCTTTGA

>Cher_CXE25

ATGGCAAACAAAACTAGTCTGGTGTCCCGGCGCGACATGAAGTGGGTGGCGCTGGTCTCCCTGATCCTGGCGACGGTGCT

GCAGCAGCCCACGCCGCTGGTGCGCACGCGCGGCGGCCTGGTGCGCGGCCGCGTCTCCCACAACGGCCGGTTCCATGAGT

ACCTGGGGATCCCCTATGGGACCGTCGGCAAGGAGAACCGGTTTCAGGCGCCGCTACCCCCACCGAAGTGGGAGGGGGTG

TTCGAAGCCGTCAACACCAACACGAGATGCCCGCAGACCCTGCTCGGCTCCGTGGGCATCATCGGCCAGCCCGACTGCCT

CAAGCTGAACGTCTACACCCCCGTCCGCACCAGGACGGACAAACTCTTGCCTGTCATGGTCTACATTCACGGGGGCTGCT

TTTTCGAAGGCACGGGATCTGCCTTCCTGTACGGAGCCGACTACTTCGTTGAACATGGAGTTGTGTTCGTTGGAATCAAC

TACAGATTAAACGTGGAAGGCTTCCTGTGCTTAGGCATTAAAGAAGCACCAGGTAATGCTGGCTTGAAAGACCAAGTAGC

AGCGCTGAGGTGGATCAAAGATAACATAAGCGCTTTTGGAGGAAACCCAGACAGCGTGACGTTATTTGGCGAGAGCGCTG

GAGCTGTGTCGTCCTCGTTCATGGTGCTCTCACCAGCAGCCAGAGGACTGTTCCATAGAGTTATCCTTCAAAGCGGCTCC

TCGCTGGCCCCATGGGGATTGCAACACGACCCAATAAGAACAGCTCATGCTCTAGTCAAACAATTCGGCTACGATACCAA

AGACCCCTACGAAATATACGATATCTTATCAAATAAAACTGTAAACGAATTAATTAAAGCTAAAGCTTTTGATAATAAGA

ACTATATTACATCTGACCGGCTGTTCGTTCCGTGCATCGAAAAAGACTTACCTGACGTCGAAGCTATCGTCACCGAGTAT

CCCACCAGCATAATAGAGTCTGGTAACTACACTAAAGTTCCCATGATAATTGGTTTTAACGACAACGAAGGCATTTACTT

CGTCGCTAAAGATTATGGCACTAGTTTGAAATCGATCGATCCCAAAGAAAACCTGACGCCTGACCTGGAGTTCCCTACTG

AATTCGACATGAACAACACGGTGGAAAGTATAAGGAGTCATTATTTTCCGCCGGATAAGGAGGAGCTGATTCAGGACATG

GTGGACTTGTACTCTGATCTCCACTTTAAGTACCCGTCTGTGGTGGAGTCGGACCTGTACGCGAAGACGTCGGGGAAGCC

GATATACTTCTACCTCTTCAAGTACGGCGGCTACAGGAACATGGCTAAGATAATATCGCGGTTCGGGGCGACGGGCGGCG

CGTCGCACGCCGACGAACTGTTCTACCTCTTCAAGCCGCACTCCATCCCGTTCCCGCACTACTTGGAGAGGCGCATGATC

AAGCGCATGGTGACGCTGTGGACAAACTTTGCCAAGTACAGCGACCCAACTCCGCAGACGTCGCCGCTGCTGCCGCTGCG

CTGGCGGCCCGGCCGCGCCGGCAACCCCGCTGCGCTGGTGATCGACAGCCAGATGTCCACAGCGAGACTCTGGGACCACA

CTGCCGTCGAGCTCTGGAACCACACCTACACTAGATACAGGAGAAAGCGGTATGGGTTCCACTGA

>Cher_CXE26.1

GGCGGCCGCGTGCGCGGGAGCGTCGCGCACGACGGCTCGCACGCAGCCTACCTCGGTATACCTTACGCCACTGTCACGCC

GGAAAACCGTTTTAAGGGTCCAGGACCCGAACCAAAATGGGACGGAATCTTCGAGGCATATAATGAAAACGTCAAATGCT

CGCAACGTTTCAACTCGTTCGTTACCGGTCAAGAAGACTGCTTAGTAGTCAACGTGTACACCCCCCTCGCCCCCGCGCCC

CCGCTGCCCGTGCTCGTCTTCATCCACGGCGGCGGCTTCCGCGACGGGTCGGGGTCCCCCTTTCTCTACGGACCTGAATA

CTTAGTCAAAAAAGGAGTTATACTAGTCACCTTTAATTATAGATTGGAAATCATGGGATTCTTATGTCTGGGCACTAAAG

ATGCTCCAGGAAATGCGGGTATGAAGGACCAGGTGGCAGCTTTGCGATGGGTDAAAAGAAACATAAGAGCGTTCGGTGGT

GATCCCGACAATGTAACAGTTTTTGGTGAAAGCGCCGGTGCGGCGTCCGTGTCCTATCATCTTATCTCACCAATGTCTAA

AGGACTGTTTAATAAAGCTATCATGCAAAGCGGCACGGCCATGGCGCCGTGGAGTTTTCAGTTTGAACCCACAAAGATCG

CCAAAGCGTTAGTAAAGAACATGGACTACAAGACCGAAGATATAGATGAGATTTATAAAATCTTGATGACAAAATCAGCT

AAAGAATTAATAAGTGCACGAGTACCAAGAGAAGAAGGTGATCAATATCAGTCTGAAGCGATTTTTGTTCCCTGTGTAGA

AAGTAAAGTGCCAGGAGTCGAACAATTCTTGCCAGATACTCCTTATAATCTTATTTTAAATGGACAGTATCTTAAAATAC

CGATGATAATTGGTTACAACAACGCCGAAGGATACCTCTTTGCTGGCAAAGAAAATGATACAACGATATCTAAACTAGAT

TTCTCGAAATCCTTGCCAAGGGATCTGATCATACCTAACGAAGAAGATAAATTGGAAGTAGCTCTTAARTTACRGGATCT

RTACWTGGGTGAGAAAGMTATATCAAAGGAGACGTTAGAGCAATTCTCTTTTTTTGAAGGTGACTCGACAATCACGTACC

CTGTTCTAGCTACCATAGACCTGTTTCTTAAGACCAATAAAAAACCTATATTTACGTATAGATTAGATTATGATGGCTGG

TTGAATGTGGCCAAGCTGTTATCTGGTTTCATGAGGGCGCCAGGGGCGACGCATGCCGACGATCTGTTTTACATGTTCAA

GCCCTTCTTTCCGACGCTTCATATGTTGGAACGAGATATGGTGGATAAAGTCACCAGCATGTGGACGAATTTTGCTAAAT

TTGGGGACCCCACACCGGAAGCTTCAGACTGGCTTCGAAGATGGCTGCCAACTGACAGCGCAAACCCTCACGTCTTCGTC

ATAGACAAGAAGTTTCACACACAACCGCTGTGGGAGGACGAAACTATGCGGTTCTGGAACCAAACCTATTTTAAGTTTAG

AAGAAAACAGTGA

>Cher_CXE26.2

GGCGGCCGCGTGCGCGGGAGCGTCGCGCACGACGGCTCGCACGCAGCCTACCTCGGTATACCTTACGCCACTGTCACGCC

GGAAAACCGTTTTAAGGGTCCAGGACCCGAACCAAAATGGGACGGAATCTTCGAGGCATATAATGAAAACGTCAAATGCT

CGCAACGTTTCAACTCGTTCGTTACCGGTCAAGAAGACTGCTTAGTAGTCAACGTGTACACCCCCCTCGCCCCCGCGCCC

CCGCTGCCCGTGCTCGTCTTCATCCACGGCGGCGGCTTCCGCGACGGGTCGGGGTCCCCCTTTCTCTACGGACCTGAATA

CTTAGTCAAAAAAGGAGTTATACTAGTCACCTTTAATTATAGATTGGAAATCATGGGATTCTTATGTCTGGGCACTAAAG

ATGCTCCAGGAAATGCGGGTATGAAGGACCAGGTGGCAGCTTTGCGATGGGTTAAAAGAAACATAAGAGCGTTCGGTGGT

GATCCCGACAATGTAACAGTTTTTGGTGAGAGCGCCGGCGCGGCGTCCGTGTCCTACCATCTTATCTCACCAATGTCTAA

AGGACTATTCAATAAAGCCATCATGCAAAGTGGCACGGCAATGGCGCCGTGGAGTTTTCAGTTTGAACCCACAAAGATCG

CCAAAGCGTTAGTAAATACCATGGGCTACAAGACTGAAGATATAGATCAGATTTATAAAATCTTGATGTCAAAATCAGCT

ACAGAGTTATTGAAGACACGAGTACCTAGAAATGAAGGTGATGTTGTTCAGTCTGAAAACATTTTTGTTCCCTGTGTAGA

AAGAAACATACCCGGAGTCGAACAATTCCTGCCAGATACTCCTTATAATCTTATATCAAATGGACAGTATCTCAAAATAC

CGATGATAATCGGTCACAACAACGCAGAAGGGTACTATTTTGCTGGTAGAGAAAATGATACAACGATATCAAAACTAGAT

TTTTTGACAGCTTTGCCAAGGGATCTGATCATACCTAACGAAGAAGATAAATTGGAAGTAGCTCTGAAGTTACGGGATCT

ATATATGGGTCAGAAATATATATCAAAGGAGACGTTAGAGCAATTCTCTTTTTTTGAAGGTGACTTGGGGATCACGTATC

CTGTTCTAGCCACAACAGACCTGTTTCTTCAGACCAGTAAAACACCTGTTTTTACGTATAGATTAGATTATGATGGCTGG

TTAAATGTGGTCAAAATAGTGTCTGGTTTCAAGAGTGCTCCAGGGGCGACGCATGCCGACGATCTTTTCTACATGTTCAA

GCCTTCCTTTCCGACGCTTCATATGTTGGAACGAGATATAGTGGATAAAATCACCAGCATGTGGACGAATTTTGCTAAAT

TTGGGGACCCCACACCGGAAGCTTCGAAATGGCTTCAAAGATGGCTGCCAACTGACAGCGCAGACCCTCACGTGTTCGTC

ATAGACAAGAAATTTCACACACAACCGCTGTGGAAAGACGAAACTATGCGGTTTTGGAACCAAACCTACTCCAAATTTAG

AAGAAAAAACAGTAACTTTACAGTGGAAGTTAATCCGTGGCTGAAAAAGTAA

>Cher_CXE27

ATGAGGACGGGTAAAAATATAGTACTTTTCTCGCTCATAGCCATGAATTTGGTGGATCAGCCGGCCCCTGAAGTGACGAT

TGAGCAAGGGACCCTGAGTGGCAGGATCAGTGATAACGGATTGGTGTTTGAGTACCTTGGCATACCTTATGCGGCGATCA

ATCGGAGCTCGAGGTTTACGGCTCCTCTGCCCCCACCAAAATGGGAAGGCATCTTCAGAGCAACCGACGCCTCAACAGCA

TGTCCTCAAAACAACTACATTGTAATCCGGGGCACAGAAGAATGCCTCAAAATCAACGTATTTGTACCAGCTTTTGCCAA

GAAACCACTTCCCGTCATGATCTTTATACACGGTGGCGCTTTTTTTCTAGGCGATGGCGGAAAAATATTCTTTGGACCAC

AATTCTTACTAAACCATGATGTCATTCTAGTCACTTTTAATTATAGACTTGGAATCTTAGGCTTCCTGTGTTTAGGTATT

GAGGAAGCACCGGGAAATGCCGGTCTTAAAGACCAACTAGCAGCTTTGAGATGGGTGAAGAAGAATATAGCAGCATTTGG

AGGAGATAGCAATAGGATTACCGTTTTTGGAGTCAGTGCTGGTGCAGCGTCGGTATCATTGCTTATAGCTAGTGGTAAAA

TAGATGGGGTTGTTAATAGAGCTATAATACAAAGTGGTTCCGCTGTAGCTCATTGGTCAATCAGCAGGAAACCGGTGTGG

GTTGCAAGCTATGTTCTCAAATCGTTAGGCTATGATACCGAAGATCCGTATGAACTATATAACATTTTGTCCAAAATGTC

TGCGAGAGAATTAGCAGCATTGCCGCATGAGAAACCACTCGAAATATATCTCGATACCCAGCTGATACACTTACCATGTA

TTGAGAAATCGATTCCCGGGACAGAATCACTAATAACTGATTATCCTTTTGATCTTTTCAAAAAGAATCCTACTAACATA

TCCATTGTTCATGGTACTACTAACAAAGAAGGACTATTTTTTGTTGCATATGACCAAGAAGAAGGAATTAACCACAGAGA

TGGAAACTATTTATTTGGGTCCGATTTAGAATTTGGAACCAGTGAAGAGGCTGTAAACGTGTCCAAAGGTATACATGAAT

TTTATTTTGGAAAGAGACGTATTAATACAGAAAGTATTATGGAACTGTCCGAGTTGTACAAACACTTACACTTTGAGATA

CCTAGCTTAATTGAATCGGAGATCATAGCAGAAGTCAGTAGCGCTCCGCTGTACAATTATTATTTCAAGTATTTTGGTAA

CAGGAACTACTTGAAACTCTCTTTAGGGATTGATAAGTCAGAAGGCGCGTGTCATGGAGACGAACTACTGTACCTTTTTG

ATGCAGTTTTTTGGCCTTTTAAAATCAATGACAAAGATGACCTTATGATTAGAAGAATAACTCGGATGTGGACAAATTTT

GCTAAATACGGAGATCCAACTCCAGAAATAACGAATGATCTTCCAGTAAAATGGCTGCCAAGTTCAAAGGAAAAACTGAA

ATTCTTTCACATAGGACACAATATGAGTATGGAAGACATGCCCAGCCCAGAAGCATACAAAATGTGGAAGGATATCTACA

CAAAATATCGTAAAACTAGGTTATAA

>Cher_CXE28.1

CTGTTGTCAGGAGCAATAGCTGGACTCATACATAGAGCTATATTAGAGTCTGGGTCAGCGATCTCACCGACGTCACTGAG

CAGTGATCCTGTTGCTACAGCTCTAAATGTAGCAATTTCTCTCGGGTATCAGGATGCTGAGACTGGATCGGCTGATGACC

TTAGCAATTTTTATCAAGAGCTCTCAGAAAATGTGTTACTCAATGTCTCCACCAGCTTTATGCCATGCATAGAGAGTGCT

TCACTTTCATTATCCAATTTATTACACATTGACCCTATGGTTATTCTGCGCAGTGGGTCATTGGAGCATGTGCCTCTTCT

GATCACATACACAAAAGAGATAATAGACGAATCTTCTCTAAATGAAAACACAGCCAAGGTTTTCCACACTCTACCTGATG

AATTTGAGGAGCTCTTACCAAATAATTTAGAGTTTGATAGTGAGGATATAAAAATTAGAATTGGTGAGCAAATAAAAGAT

GTTTACTTCGCGGATGATATCTTGGGCGGAGATGTGGCTGAGTGCTATGAAAATTACATGAATGATGTGTTTACTGTGTA

CCCCATAGTCAAGTCAGCGACCTTGCACGCCGCTTCCAGCCACCTTCCGGTATACCTTATGGAATTCTCGCTTCAGGGGG

GAAACAAGAGCATCTTTGACTACATTTTTGCTAAAGACATTCTCAGCGATGAGGATGAATTAGTGGCTGAGAAGCTTGTC

AGTTTATGGAGTAACTTCATGAAGTATAGTGACCCCACACCTCTAGCCACATCAATATTGCCAGTTGTATGGCAACCGGT

AACTTTTGCAGTAAGGAATGAGAAAGCGGACATCAGCACAGCGACGTGCCTCATCTTTGACTCAGGCAGCGGGCTTGCGA

TGGGCCGGCCATCATCAAGTCACACATTGATCATCTGGGACCACATTTATGACAACTTT

>Cher_CXE28.2

ATGTTGTGGTCTTCGCTTTGTGTTTTAATAATTCTGGAATATTCTGTGGCGGTGGTCATTCCGGCTAAAGGGGCCTTTAG

CTCTGTCCCTCTTGTGACCAGTACGAGAGATGTGGCTACAGAATCTGGTGTGGTTCGAGGGCGTCTGGTGGACGAAGGAG

AAGGAGACTATTATGCCTTTCTAGGAATACCTTACGCTACGCCGCCTACGTTTGAACGACGGTTTAGGCCTCCCGTTCCA

CCCGTACCATGGGATGGCATCTTTGTCGCAAACCGCACAGTCCAATGCCCCCAAAGAGGCACCGGCGAAGAGGACTGCCT

CGTCGTCAACATCTTCACCCCCACCACAATAGTGAACAGCCGAGCCCCTGTGCTGGTTTACCTCCACGGAGGATCCTTCA

TCATGGGAGCAGCGCAAACCCAAGGAGTCGGGCCTTTGATCAAGCAGAACATCATAGTCGTCACGGTCAACTACAGGCTC

GGGGCGCTGGGTTTCCTCTGCTTGGGAATAGACGAAGCCCCAGGTAACGCAGGGCTGAAAGACCAGAGCGCAGCGCTCCT

ATGGGTTCAGAAGAACATAGGACATTTTGGAGGAGATCCTGCCCAAGTGACTTTGTATGGCATGAGTGCTGGAGCGGCAG

CGGTGGAGCTCCACGTCCTATCGAAAACAAGCAAGGGATTGTTTCAGAGAGCAATCGTGGAGTCGGCGTCTGCCTTGTCT

GTCTGGTCTGTCGACGCTGAGCCGATACAAACCGCCTTGACCTTCGCGGAATCTTTACACCTTCCCTCACATATAACCAA

ACGTCTGTACAGTTTGATCAGCTATCTCCAACACGTGCCCGCTGATACACTAAGCACATCCAACCATGAGTATTATAGCA

ACTTTACCGATATGACGTTCGGTTTCGCACCGTGTATCGAGAAGAAGTTCTACGGCACCAAACCGTTCATATCGAAGTCC

CCTTTGGAGATACTGACCAAACAGAACTATTCCAAGGTTCCGATCATGTTCCTCTATTCGGCTTTAGAAGGCCTGTTCCT

CAAAAGTGAAGAGTATTACACGCAAGACTACAAGGACAGAATGGATTCGAATTTTCTTGAGTTCATGCCATCTGATCTTG

TCTTTGACACGGAAGAGATAAAGCAAGAAGTAGGCAAGAATATAAAGAGTTTCTACTTCGGCAATAAGACGGTCGGAGAA

AACAGTGTAGTTAACTATTTGAACTACTTTGGGGACGGGGCTGTAATGGACGCGATGCTCAATTCTGCTGAGTTGCACGC

TAGGGGCGGGAACCCGGTGTACTTGATGGAATTTGCATATAAAGGGAACATGGGAAGCTATGAGAAGTTTTATGAGAACC

TGACGTTGGCGGGGCATGGTGACGTGGTGAAGCATGCGATCCTGGAGAAGCCTGTGAGAAGCCCCACCGATAGACTAGCG

GTGGACCGTGTCGCAAAGTTGATTGCAAACTACGTCAAGTTTGGTAACCCCACACCAGCCCCAACAGAACTCCTCCCAAC

CCACTGGCCCACAGTGAAACCCGGCAACGTGAGCTGTCTCTACATGGACCAGGCCTTCACAGTGCACGAGGAGCCCCACT

GGGAGCAGCGGCGGCTCTGGGACAGCCTGTACAGCCGCTACCGGAGGCCGATACGGCCTGTGGACCCGGGGTACCTGTGG

CGGGCTTTTTTTTTGACACTGTGGTGCATA

>Cher_CXE29

CGGCTGGTGGGCACGGCGCAGGGGCCGGTGCGCGGGTACCACGCGCCGCAGGGAGACCACTTCGAGTTCCAAGGCATCCC

TTACGCTACTGTGCCTGCCGGATCTAGCAGGTTCATGGCACCTCTCCCTGGACCAGTTTGGCTGCAGCCTCTGGAAGCCG

TCAACGAAACTACGATATGCCCTCAATTCAACGCTATACCTCGGATGGCAACAGACAAATTTATCAGAGAAGATTGCTTA

ATTGCAAATGTTTATGTTCCTGATACAGAAGAAAAAAACCTTCCTGTAGTCGTCATAGTGCACGGAGGTGCTTACCAAGT

CGGGTTCGGTAATATGATGAAGCCACAAGGCTTAATGAAAAGTAAAAAAGTGATTGCGGTAACATTTAACTATCGTCTAG

GAGTTCATGGCTTCCTGTGTCTGGGCACAGAGGGGGCTCCAGGCAATGCCGGTATGAAAGATCAGGTTGCCCTGCTGCGA

TGGGTGAAGAAGAATATTAGGAAATTCGGTGGCAACCCCGACGAAGTCACAATCGCTGGCTACAGCGCTGGCAGTTCATC

TGTGGATCTTTTAATGTTATCGAAAATGACAAAAGGTCTCTTTAACAAAGTCATTCCGGAAAGTGGTGCCAATGTGGGAG

TGTGGAGCGTGCAGATTGATCCGCTTGAAAACGCAAAGGCATTCGCTAAGCAACTCGGCTTCGAGGACGTGGACGACGTC

TACGCCTTGGAAGAATTTTACAAGAGCGCTCCCTTGGAGTCTTTGGCCGCGGCCATGTTTACAGATAGGAAAGATTCCAG

TTTTGTTTTTTCTCCGTGTGTTGAACGGAATACCGGGGTTGATAAATTTCTCGATGATACGCCAGTAAATATTATAAAGC

AGGGTAAATACAGCAAAGTCCCCGTCTTGTATGGCTTTTCAAATATGGAAGGTATTTTCCGTTTACCGGAATTCGAAAGG

TGGAAGGATGAAATGAACACAAATTTCTCTTTGTTTTTACCGCTGGATCTCGAGTTTCAAAATACGGAAGAGAGAGAAGA

AGTTGCCAATTCAATAAAGGAGTTTTATTTCGGTAGCAAGCCGGTCGGGGAGGACACGGTTTTGGATTACATTGATTACT

TTTCGGACGTAATTTTTGCTTATCCGACGTTGCGCTCGACGCAGTTGCAAGTGGAAGCAGGAAGCAAGTCAATATTCTTG

TATGAATATTCTTTTCCTATTAGTATATTTACTAGTTACTTCTTCAACCCCGACCCGGTGCCCGAGTACCTGCGGCGCGT

GCGCGGCGCGCGGCACGTGGCGCAGACCGACGCCGTGCTGGACTCGATGATGGCTGCGCCGGCGCTGGCCGACGGCACTA

CAGCCGAAGACTACGAAAAGCATAAAGAAGTAATCAAAGAATTGTGGATAAATTTTATAACTACTGGCAAGCCCAGCAGC

ACCGACGCGAGCGCCGCGGCGCTGGCTGCGTGG

>Cher_CXE30

ATGTCGGGCCAAGTAAACGTCGCCCAAGGCACTCTTCAAGGAAAACTGTGCACTTACAAAGAGAAAACGTATTATAGTTT

TGAAGGCATACCATATGCTAAGCCGCCCGTTGGAAAGTTGAGATTCAGAGAACCACAAGAGCCTGAAAGTTGGACCGGAG

TTCGCGATGCCACAAAACCAGGAAACAAATGTGTGCAAATCAATCCAGCAACTATGAGTGACATAATTGGCTCCGAAGAC

TGCTTATACCTCAATGTTTACACACCTAACTTGCCAAAAAAGCAGCTCAAGAAACTTCCTGTAGTATTTTTTGTCCACGG

TGGCAAACTAATATTCGGCTATGGAGACTACTACAGACCAGACTATTTCATTGAACAAGATGTTATTCTTGTGACAATGA

ATTATAGATTACATGTGCTTGGTTTTCTATGTCTTAATGAAGCTGAAGTAGCAGGAAATATGGGACTTAAGGATTCTGTT

ATGGCATTAAACTGGGTTAAAAGAAACATAGAAAAATTTAATGGTGACAGTAATAACATAACTGTATTTGGAGAGAGTGC

CGGTGGGGCGGTCGTGACATCATACTTAACATCCAAAATGGCGACTGGTTTCAACAGAGTTGTTGCTATGTCGGGCTGCT

GCGTGTCAGACTTGTATTTGATCGATGATGATCCTATTTTTAAAGCTAGACATTTAGCTTCCGTTCTAGGAAAAGAATTC

CGTGATCTCAAAAGTTTACACGAATTCCTGGTAAATGTTCCAATAGAGGAGTTAATATTTGGATTGGGCGTTGTGGAATC

GTCTAGGCCCCCATCAATAATAAATGCATATTTTCTCCCCGTCGTAGAAAAGAAATTCGATGGTATGCAGCGCTTCTTTG

ATGAACATCCTATTGTCACATTACGTGAGAATCGGTACAAAAAAGTACCTTTGCTGTTTGGCGTAAATAGCCACGAAGGG

GCATTTTTTGCACAAAAAGATAAAAAAGGACGCATAGTGTTCGAGAAGGACTTACTGTACTTTGTACCACGCTTTTTGTC

TGTGAATTCATACAGCCCTGAAGCTATTAAAATTGCAAATAATTTGAGAGATTTCTACTTCAATGGTGAAGCAATAGACG

AAACTAAAATGTTGGAATACATAGAATTTGTTTCAGATGCTTATTTTAATAGAGATATTGTGACTTTTTTGGATGCTTTC

GGAAAATATCATAATGAAGTATATACGTTTCGGTTATCATATTCAAGTAACATGAATACTAGGATTATGAAAAATCTTGG

TCTGAAAGGCACGACACACGGGGATTTGCTGCAATATATTTTTTACAGAAAGAGGAAAGCTGACAGTGCTAACGATAAAG

ATAGAGAAATAATGAACATTTTGAACGAGACTGTCTGCAACTTTGCGAAAACTAGTATTCCAACATGGTCGACAAAAACT

GTAAAATGGGAACCATACTCCTCTAATAAACGCAGCTTCCTCGAAATTAACGAAGAACAAAAACTTGTGCACAATTTCAA

CAACAATAATCATAAAGTTTGGCTGAAAATGGGAGAGAGGTCTAAACTGTGA

>Cher_CXE31

ATGGTCTTCATCCACGGAGGAGCATTCATGGAAGGATCAGGTTCACCCGGCCTATACGGCCCGAAATATATAACCTCAAA

AGGGGTCATCCTTGTCACTATAAATTACCGACTCAACGTTCATGGGTTCCTCTGTTTACGTATAAAAGAAGCTCCGGGAA

ATGCGGGTGCTAAAGACCAAGTGGCTGCGCTGCGGTGGCTCCAGAAGAATATCAGGGTTTTTGGAGGAGATCCCGATAAT

GTGACGCTGTTCGGTGAGAGCGCCGGCGGGGCATCCGTGTCCCATCACCTATTGTCCAAAATGTCCAAGGGTTTGTTCCA

TAGAGCGATCATGCAGAGCGGAACAGCTCTAGCTTCTTGGGGACATCAGTCAGATCCGGTGAGGGTCACCAACTCTGTTG

CAAAGACCATGGGCTACAAAGTTGACGACCCGCTCGAACTTTACAAACTGCTCATGAGTATGACCGAAGTAGAACTTATC

ACCCCCAGGATACCTCGTGAGCTCGGTGGTACCATCATGTCTGAAATGCTAGGTTCTCCGTGCGTCGAAAAGATAATAGA

TGGCGTGGAACCATTTTTAACAGAGGATCCTTACACGCTGCTGGCGAAAGGAGAATTTACAAAGATGCCTGTAATGATAG

GCACAAACAGCCAAGAAGGGTATTTCTTAGTCAGTTACGAGAACAGCACAACGTTATCACGGATGAATTTTGAGAGTGCT

CTGCCCAGAAATCTAGAGTTCCCTACAGAACAGGAACGAAAAGAAGTAGCGTGTAAAGTTAAGGAAATGTACATGGGTAA

CGATACGATTTCTAAAGATACTCTTGAGAAATTGTCTAAGTTTCATGGCGAGCCGTTCTTCAAGTATCCTGCGGTAGCTG

AGACGGAGTTGATCTTGGAAAATAGTGATCAGCCGGTGTATAACTACGTGTTTAATTATGATGGTCGAAAGAACATAGCT

AAGTTGTACGCTGGGTTTTGGGGATTGCCTGGCGCGTCGCACGGCGATGAGTTGTTCTACTTGTTCGAGCAACACAGCGT

GCCCAACATGTTCGAGTGGAGGATGATGGACAAGGTCACGACCTTGTGGACCAACTTCGCCAAGTTCGGAGACCCAACCC

CAGAGGTGTCAGAACTGCTACCAACGAGATGGCAGCGGACCAACAAAACCAGTCCTCAAGCATTAGTCATAGGCCGAGAA

CTCTCCACTGCCCCTCTGTGGTCCAGTAGCTCGCTCAACTACTGGCATGAGGTGTATACTAAATATAGGAAAATTTCATC

GCAATGA

>Cher_CXE32

ATGCCAAACCCGAGAGGTGTACGCGAGGAGCACAAAGGAGGTGCTCTGCCGGCGCGACACCGACATGATCAGTCGGTGCT

CATTTTCCTAGAGTCGAACCGACGTTCTAAAAGTACGAGAGCCGTGCGTTTGTTTCCACTTGGTTTTTTGCTTTCGAGGC

AGCACGATCATCTCGCAACCATGCCAAACCAGTTTACATTATTTAGTTATTTTAGTGTGGGTCGTAATAAATCATTATCG

GTGTTGATATTTTGCATGATTGCGACTGCATTTGCCGGTGCCGATGCTGATAATCCCACAGTCGAGACTCCTCTCGGTGC

AGTGAGCGGACAATACCTTAAGACACGAGAGGGAAGAACTATTTCGGCGTTCACGTCTGTCCCGTACGCCGTACCGCCTG

TCGGAGATTTGAGATTTAAACCTGTGGTACCAGTTGAGAAATGGGACGGAACATTAGACGCGACGCAAACGAGCCCTGCC

TGTGTGCAGAGGAACCCCTACACCCGTCAGCAGGAGATAGTGGGGCAAGAAGACTGCTTATACGTCAACATATATACCCC

GTACACCAGTGAAGATCTCGCCGGGAGCCACAAGCCGCTGCCGGTGATGGTCTTCATCCATGGAGGCGGTTGGATGTGCG

GAGACTCTTCCACGGACATGTACGGGCCAAAGCACCTTTTAGACAGAGACGTGCTGTTGGTGGGGATCAATTACAGACTG

GGGCCCCTGGGTTTCCTGTCCACGCAAGACGCCCTCTGTCCCGGCAACAACGGGCTCAAGGACCAACAAGAAGCATTTAG

GTTTATCCAGAAGGTCATCTCCAGCTTTGGAGGAGACAAAGACTCCGTAACAATTTTCGGGGAGAGCGCTGGTGGCGCTA

GCGCCAGTTTCCACATGTTGTCTCCAACCAGTAGAGGGCTATTTCACAAAGTAATAGCGGAGTCAGGAGTGTCGCTGGTG

CCGTGGGCGGAGGCCCCGCCCGGCGAGGCGCTCAAACAGGCCTTCCGTCTGGCGAAGTTCCTGGACTGCCCGCAGGCGCC

TTCCGAAAAAATGCTCCAGTGCCTTCGCACCAAGGACAGCTATGATGTTATCAACACTGAATTCCAGTTTTACGGTTGGG

ACTATGAGCCTATGACTCCGTTTAAGGCAGTGGTTGAGCCAGACCTCCCCGGAGCCTTCTTGACTGCCGCGGCGAGAACG

ATTCGTGACATCGCGGACGTGCCCATGATGACGGGTCTGAACAATGCGGAAGGATGCCTAAAATCTGTCTGGATAACAGC

CAATGAGTCAAGATACAAAGAATTTAAGTCTGGATTCGACACAATAGCACCCATCACGTTCTATTACGAAAATTCGCCAT

TTGCTGATGAGATTACCAAGGAAGTCAAATCATTTTATTTAGACGGAAAAGACGAAGAGGGATACAAACAAGGCATTCTC

GATATATACACGGACTCCTACTTTGCGTATCCCGCAATAGAGTCCCTTGAACACACGATGAGTTACTCCAAAAGCCCGGT

GTACCTGTACGAGTTGACGTACAGGGGGAATAACAGCTTTTCTCAGATATTCGGTGATACGACTGGGGATTACGGTGTAT

GCCACGCTGATGAACTAATGTATTTGTTCCCGGTAGGATTTCTTCTAAATGAACCTTCAAAAGAAGATGTGATAATAAGT

AAACTCATTGTTACTTTGTGGACAAATTTTGCCACCACCGGGAACCCAAGTAAACCAAATGAAGTGCCCTTCAAGTGGGC

GCCGGCGACTAGCTCGAAGACCCTAGAGTACCTGGACATAAGCGAGCAGCCGACTATGAAGAAGCACCTTTCCGAGCGCG

CGCGCTTCTGGGGTACGCTGCCGCTCTGGCATAGCCTGCGCACGCACAGACTCGCCGACGAGCTGTGA

>Cher_CXE33

ATGGTCTACATACACGGAGGCGGCTTGCAATTCGGCTCAGGCAATTCTAGCTCTTACGGCGCTGATTTCCTAGTCGAGAA

AGACGTGGTGGTGGTCACTATTAACTACAGGGTAGGAGCGTTAGGTTTCCTCAGTCTAAACACTCCTGAGGTGCCCGGAA

ACGCTGGCTTGAAAGACATGATTGCCGCGATACGATGGGTGAAGGAGAATATTAAGAATTTCGGTGGCAATTCTGGCAAT

ATCACGCTTTTCGGTGAAAGCGCGGGCGGTGGAGCTGTATCTGTCTTGACAGCTAGCCCATTGACTAGAGACTTGATCAG

CAAAGCTGTCATCCAATCAGGGACCGGCCTCAACAGCTGGGCGTGGCAAAAAAGTCCGTTTGAAAACGCTAAGAAGCTCG

CTCACTACATCGGTTGTGAATCTGACGATTCTGAAGAAATCTTAGAGTTTCTCCGAGCGACTGTCATCAAGGACATAGTT

GAAGCTACTAACAAATTGTTCCCGCTTGACTTATTCTTAGAAGGCAAGGAGTTTGGCTTCGCTTTCGTGGTTGAAAAGGA

GTTCCCAGGCGTTGAAGCAGCAGTTACCGAATCATTCCTAACCATGCTTACATCTGGACGCGTCGCCGAAGTTCCAATCA

TGATCGGTTCAACAACAATCGAGTTCCTCTTCAAAATCGATTCTGAAGACTTACAAGTCTTCATCCCTAACAACCTAAAC

ATCGAGAGGAATTCAGACGAAGCTATCCAAATAGCCAATCGGCTCAAGACTCTATACTTCAAAGGGAACCACACAGCTGT

TGAAAATATCCACGGCTACTTCGAACTGATTTCAGACAAGCTCATTAACATAGACACCCACAGACACGTGCAGTATCTAG

TGCAGGCTACCACGAAACCAGTGTATTATTACAAGTTTGATTACGTGGGTGAACTGAACCTTTCTAAGAAAATGATGGAT

TCTCTGAATGTCACTCACGCTGGGCATACAGATGAGCTCGGGTATCTGTTCAAGAATGACTCGCTGGAAGGTATTGAACC

AACGGCGCAGGATGTCAAGATGAGGGAAAGGATGGTGCGGCTTTGGACCAACTTCGCTAAGTCTGGCAACCCAACCCCAG

ACGAGAACCACTACATCAACATAACCTGGCAGCCANNNNNNGTGACGAAAGACAACCTTTACTACCTGAACATCGGCTCA

GAACTCTCCGTCCAAACCAGCCCTGACAAGGAAAAAATGGACTTCTGGGACGAACTCTACAGCAAGTATTTTAAGATCTG

GGACCATCCGCAGACCAACCAAGAAGAGTCGCCAGTTAAAGCCCATAAGGAGTCAGTAGAAGTTGTTGAACCAGAGGTTA

TTCAACCAGAGGTGATACAACCAGAGGTGATACAACCAGAGGTTGTACAATCAGAGGTTGTACAACCAGAGGTGGAGTCG

GAACCGGTGGAGCCGGAGACGGGAGAGGCAGTACAAGAGACGGTTGAAGAAGTATTGGTCGAAAATTTGGTCGCGGTCGA

GCCTCAGGCCAATGGTCACGCGAACGGTGTAAACGGAGACACTGAGAGAAAGCCTAGGGCTTCCAATGAGATTAAGATGG

TCCAAAGCAATGGCGCTCCTAAGGACGTCATCAGGGCAAATGATCCACCA

>Cher_CXE34

ATGCAGCCTTTCGAGGCAGTGAAAAACGACATAATATGTCCTCAAGGAAAGTTCAAATACTTAAATTTGGACGACTACGA

CATGCGAGAGGACTGCTTGGTCGCCAACGTTTACATGCCAGACACAGAGGAGACCAACCTTCCAGTCGNANNNNNNNNNN

NNNNNNNNNNNNNNNNNNNNNNNNNNNNNNNNNNNNNNNNNCCAACAGCTTTAGTGAGAAGTAAAAAGGTAGTCGCTGTC

ACTTTCAACTACCGATTAGGTGTTCACGGCTTCCTGTGTCTGGGCACAGAAGGCGCTCCCGGCAACGCGGGCATGAAGGA

CCAAGTCGCACTGCTGCGCTGGGTGCAGAAAAACATTGCCAACTTTGGTGGAAACCCTGATGACGTCACCATCGCTGGAT

ACAGTGCAGGTTCCTCAGCAGTGGATCTTCTTATGCTCTCTGATACGACTGAGGGACTTTACAATAAAGTAATACCTGAA

AGTGGTGCAAGCGTCGCCGTTTGGAGCNNNNNNNNNNNNNNNNNNNNNNNNNNNNAGAAATTCGCCAAACAATTGAACTT

CAGTAATGTAGACGATATTTACGCCCTAGAAGAGTTTTATTCTACTGTGTCCTATGATGCCATAACATCGGATGTGTTTT

TCGATCAGAAAGATTCGACGTTCGTATTTTCGCCTTGTGTGGAGCGTGACACAGGCGTAGAACGGTTTCTTGCCGATGCT

CCTGTAAATATCTTAGCGGAAGGCAAGTACCGGAAAGTGCCTGTTTTATACGGGTTCGCAAATATGGAAGGTCTATTCCG

TATGATGGACTTTTTTCAATGGAAAGACCAAATGAATGAGCGTTTCTCGGACTTCTTACCTGCTGATCTGCAATTTAGCA

GTGACAAGGAAAGAGAACGAACTGCGCGAGCAATTAAGGAATTTTACTTTGGAGACGACCCGGTGTCGGAAAAAACTATA

CAAGGCTTCATTGACTATTTCTCGGACGTCATATTTGCTTACCCCCATCTGAGATCCGTGGAGTTCCAAGTCAACGCTGG

TAGTGATTCCATCTACCTCTATGAATACTCATTCTTCAAGCCATTCCCTGAGAACATAGGCCTGCCAGACTTTATAAAGA

ATATACACGGAGCAGCCCACTGCGCTCAGTCAAATATGATTCAAGAAGGAAGCAAGTACTCCAGTAGTGTGGTTGATGAA

GAAGAAGATTTTAAGAAAATGAAAGCAACCATGGTTGAACTCTGGCTGAACTTTATAACCACCGGCAAACCGGTGCCTGA

AGGGTCAACGCTCCCATCTTGGCCTCCTGTCAAAGGGAACAGGTCACCTTATATGGAGCTCAATTCGGAGTTAAGTCTGA

AGGGCCCTCTCCTCAAGAAGCGTGCACTTTTCTGGGATGACATTTATGACCGTTTCTACAGGATGCCAGTGGCGCCACCT

GCTCCTAAAGTTAGGAGTGAACTTTAA

>Cher_CXE35.1

CTGCACGATGGCTCGTCCTTCTTCAGCTTTAAGGGAATCCCTTACGCTCAGCCGCCGGTTGGGCAGCTTCGGTTTAGGGC

CCCTTCGCCACCTCAACCATGGAGCGGCACCCGTAACGCGACGCAGTACGGCGACATCTGCACCCAGTATGACCCCGAAG

CAGGTGCCATCCTAGGCAGCGAGGACTGCCTTTTCGTCAATGTCTTCACTAAGTCCCTGACTCCTTCCTCCAAAATACCC

GTCATGGTTTTCATCTATGGAGGATCCAACTCAGCCGGATCAGGCAACGACGACCTCTATGGACCGAAATTCTTCCTCCA

ACAAAACGTTATCCTAGTTACTTTTAACTATAGACTTGAAGTACTAGGTTTCCTAGCCTTAGATACCCCTGAAGTACCCG

GCAATGCGGCCATGAAGGACCAAGTCGCGGCGCTGAAGTGGGTGCAGTGTAACATTGAACAGTTCGGAGGAGATCCTAAC

AAAGTTACCCTCTTTGGTGAAAGTTCGGGCGCGGCTTCTGTCGGATTCCACATGCTGTCACCAATGTCCCGGGGTTTGTT

CCACAAGGCTATAATGCAGAGCGGGACCAGTGCCAACGATTGGGCAGAAGGCCATGACGGTAGAATCAGGGCGTTCAGAG

TTGGCAAAGTCTTGGGAAAAGATACCAACGATGTAAATGAACTCTTGGCGTTCCTAAGAAGCGTTTCAGCGGTGGATTTA

ACTAATATGACCATGAGGACTTTGTCGGAAGATGAAAAATATAGAGGCCTGCCTGAGACCTTCATAACGGTCGTTGAAAA

GAAGTTTGACAATGTCGTAGCGTTTTTGCCTGAACATGCATTGGACATTCTTGTCTCGAAAAGAGCGTGCAGTGTTCCGT

TGATGCTCGGATACAATTCTGCTGAAGCTATAATAATGCTTGAAGATAGGATGCAAAAATTAGACATTTACAATAACAAT

CCCTCCTATGATGTTCCTAGAGAAATAGCTGAAAAGGTTACGCAAACGAAAATGACTGAATTTGGGGAAAGAATAAGGAA

CTTCTACGTCGGAAATAGAAAATTTACAAAGAATGATGGGGAAAAAATTGTGGCCATGTTGTCTGATATGCATTTCCTAT

ACGGTGCGCATAGATTTGCTTATTTGTATTCTAAGTATAGCAATCCAATCTACATGTACAGGTTTAGTCTCGTGAGTGAT

CTCAATATTGTTAAGAATTCAATAAATTCGACTGTAAAAGGAGTATGCCATGCCGATGACCTATTCTACATGTTTTCAAA

TGATTTGACTAAAGATCAATATGAAGCACAGGATACATTGAAAACCTATGTTTCGAAGGTGACGAAACTGTGGACCGACT

TCGCAAAAACAAGTAACCCGACCCCAACCAGAAGCCTGGGGGCGACGTGGCCGCGGTACACAGCGAAGAACAAACGTTTC

CTGGACATCGACGTTGAACTGACGCGCGGAAAATATGCGGAGAAGGAACGGGTGGAGTTCTGGAACTGTCTGTTTAAGGA

AGTTGACTTGCCTCATATAAACTAA

>Cher_desat5

ATGCCGTCAAATTCAGAAGAGATTTTCCTACAAGAAAAGGAGGTCTACGAGAAACTAGTGGCGCCACAAGCGGCTCCGAG

GAAACATGAGCTGGCGATATTGCCCGTCTCACTCTTCACCTACTGGCACGTCGCTGGCTTGTACGGACTGTGCCTGATCT

TCACTGCGGCAAAATGGCAGACCTTAGTGTTCACTCTGTTTATGTACAACGCCGGCATTCTGGGCATCACGGCGGGAGCC

CACCGCCTCTGGGCCCACAAGACATACAAAGCCAAGAGGCCCCTAGAGATCCTGCTCATGATATTTCACAGTATGACCAG

CCAGAATACTGTGCGACACTGGGCAAGGGACCATCGGTTTCACCATAAGTACAGTGACACAGACGCTGACCCACACAACG

CGACTCGGGGCTTTTTCTACTCCCACGTCGGCTGGCTGCTGGTAAAGAAACACCCGGAGGTCCTAAAGCGATCGAATACT

ATAGACATGTCCGATATTTACAACAATCCTGTGCTGCGGTTCCAGAAGAACTACGGCCTGCCCGTCATAACTTTCTTCGC

CTACCTCCTCCCAACCTTAATCCCAATGTACTTCTGGAACCAAACGTTCAACACAGCCTGGCACATCAACCTACTCCGCA

TCATAGCTAACCTCCACGCGAGCTGCCTAGTGAACAGCGCAGCCCACGCATTCGGAAACAAGCCTTACGACAAGCGCATA

GCAGCCACGCAAATCTCCACCCTATCCTTCATAACTTTAGGGGAGTGTTTCCATAACTACCACCATGTCTTCCCTTGGGA

CTACAGGACTGCAGAACTGGGGAATAATTGGTTGAATTTAACAACTATTTTCATTGATTTTTGTGCTTGCTTTGGCTTGG

CGTGTGATTTGAAGATTGTGTCTGATGATATGGTGGAAGCGAGAGCTAAAAGAACAGGGGATGGCACGAATTTATGGGGG

TGGGGGGATAGAGATTTA

>Cher_ELOVL1

ATGGAGTCCGCAACTTGGACCTACAATTTGGACACTACGCCAAACTACTCGTACATTTTTGATTTTGAGAGCGAGTTTAT

TCACCAGAATTCTAGGAAATGGATGACCGAGAATTGGACTGTGGCGTTTTACTACATCTCGGTGTATATGGCGTTTATTT

TCTTCGGGCAGTTGTACATGCAGAACCGTCCCAGATTCGAGCTCCGACGGACGCTGATCATCTGGAACGGTGGACTGGCC

GCCTTCAGCATCATGGGAGCCTGCCGGACGCTCCCTGAATTCATCCACGTGCTACGAAACTATGGGATCTACCATTCCGT

CTGCGTGCCAAGCTTCATCGAAGCGGACAAAGTATCCGGCTTCTGGACGTGGATGTTCGTCCTCTCCAAAGTGCCCGAGC

TAGGGGACACAGTATTCATTGTCCTCCGCAAGAAGCCGCTCATCTTCCTCCACTGGTACCACCATATAACGGTGCTGCTG

TACTCCTGGTTCTCATTCACCGAGTTCACCTCCTCGGCGCGCTGGTTCGTGGTCATGAACTACTGTGTGCACAGTGTGAT

GTACTCCTACTACGCTCTAGTGAGCATGGGCAAGTACCCCCCAAAATTCCTCGCAATGACAATCACCTTCCTCCAACTCA

CGCAGATGATCGTCGGCTGCGCGATCAACATTTGGGCGCACAACTACTTAACCTCCTCCACAGGTTCTTGCGGCATCAGC

CAGTTCAACATCAAACTGTCCATGGCTATGTATTTCTCCTACTTCATCTTATTCGCCCGCTTCTTCTATAAAGCCTACCT

AGCACCCAAAGCAGGCAAGAAGGCTAAGGCCGAGCCTGCCATCGATATCCCGAGCGCCAAGAAGAATTATGACGAATACC

CTCGGCAACGGAACGGCGTTGTCGCCCACTAG

>Cher_ELOVL2

ATGTCTGGCTTTGTGAAAATCGCCATCGACGATTACTATTCCGATAGTAATTGGACGACCATCATCGAAAAGTACTGGAT

GATGGCCGAAAAAGTTTCAGATCCTCGCGTGCAAGGATGGTTCCTTTTCGACACGCCCCTCCCCACTCTTGCCATGGTGA

TAGCCTACATCGCCTTCGTGATGGTCATCGGACCTCTGTGGATGGAGAACAAGAAACCTTTCCAGATCAAGAACACCCTG

GTGGGATACAACGCTTTCCAGGTGTTGCTGTCGTCGTATATGTTTTATGAGCACTTGATGTCGGGATGGTGGGGCGACTA

CAGTCTGGCGTGCCAACCGGTAGACTACAGCGAGAGTGAACAAGCGAGAAGGATGCTGCATCTATGCTGGGTGTACTACT

TCTCCAAGCTGTCAGAGTTCGCGGACACGGTGTTCTTCGTGCTACGGAAGAAGAAGAGTCAGATCACGTGGCTGCATCTA

TACCACCACTCGCTCACGCCTTTTGAGGCTTGGATGCTGGTCAAATTCATTGCTGGCGGTCACGGAACCTTCTCGAACAT

CGTGAACAACCTCGTCCACATCATCATGTACGCGTACTACATGGCATCGGCCATGGGACCCCATATGCAGAAGTACCTAT

GGTGGAAGAAGCACCTCACCACTCTTCAACTAGCCCAATTCTTCATGGTGCTGTTCCACTCCATCAGTGCACTCGTCTGC

GACTGCGGATATCCAAAGATCATCGCCTTCGGCCTCATTCTCCACTCGAGCATCTTCATCGTCCTCTTCACAAACTTCTA

CATCCAAGCGTACAAGAAGGACAAAATCAAAGCCAAACCAGAACCCACCAATAACAACAGCCTTGCCAACGGTTACGCCA

AAACCAACGGCCAAGTGCCCAATGGCCAAGTATCAAACGGCCAAGTGCCCAATAGCCAATCCAATGGCCATGTCAAAAAT

GGCTTAACGAACGGTCACGCCACTGACGTCAGAGAAAAAAAAGTTTCATAA

>Cher_ELOVL3

ATGGAGCAGCTGAATAAGCTAGTCCAGGGTTACCATGACCTTATGGACAACAAGAGTGATCAGAGGGTCAAGGATTGGCC

ACTGATGTCCTCTCCCTTCCCCACATTGGCGATATGTCTCACATACGTGTTTGTAGTCAAAGTAGTGGGGCCAAAGTTTA

TGGAAAATAGAAAACCGTACGAATTAAAACAAGTGCTTATCTGGTACAATTTAATTCAAGTCATCTTCAGCATTTGGTTG

TTTAATGAGAGTGTGGTCAGCGGTTGGTTCACGACGAACAGTTTCCGCTGCCAGCCCGTCGACTACTCGCGGTCGCCCAT

CGCTATGCGGACGGCGAACGGTTGTTGGTGGTACTATTTTTCGAAATTTACGGAATTCTTCGACACGCTCTTCTTCGTGA

TGCGCAAGAAGTTTGACCACGTGTCGAAGCTGCACGTCATCCACCACGGCATCATGCCCATGTCCGTCTGGTTCGGAGTC

AAGTTCACACCAGGTGGCCACTCGACCTTCTTCGGATTGCTGAACACGTTCGTGCACATCATCATGTACTCGTACTACCT

CCTGGCCGCGCTGGGGCCCAGCGTGCAGAGGTATCTCTGGTGGAAGAAGTACCTCACCTCCCTGCAAATGATCCAGTTCG

TGTTGGTATTCTTACACGCGTTCCAGTTACTGTTCGTTGATTGCGACTACCCTCGCGCCTTCGTCTGGTGGATCGGCATG

CACGCTGTGCTGTTCTACTATCTATTCTCTGACTTCTACAAACAGGCCTACCGCAAGAAGGCAAAGGCCCAAATCGCTCG

AGCGAAAGCAAAAGTAGCTGAAGCGGAGGAGACAAGCAAGTCGGAGACGAAAGACATGATGTACCCGTTGCTAAATGGGT

TCAGTAATGGCGCGGCACTCGGCGACGTGCGGCAGCGGGTCGCGGGCGCCGTGGCCTCGCAGTGA

>Cher_ELOVL4

ATGGGGACACTAGTTCATAACATCACGCATTTCTACCATTATATGAACACAGATGTGGCAGATCCTCGATCGAACAGTTT

CTGGCTGGTATCGAGTCCATGGCCCATCATGATCATAATGTACGCGTACCACCAGTTCGTGCGCAAATGGGGTCCCGCCT

TCATGGAGAACCGGCCCCCGTACCAGCTCAAGAACCTTATCATCCTCTATAACGTGGTGCAGATATATTTTTCGGCATAC

ATAGCTTTGCGTTGCTTCCTGACCCTGTATTGGTCGGGGCACTACAGCCTGTGGTGCCAGAAGATGATCATCGACGAATC

GCCCATCGAAACGATGGTGGTCAACCACGTGCACTATTACTATATCATCAAAGTCATAGACTTGCTTGACACGGTTTTCT

TCGTGTTGCGCAAGAAGTTTTCGCAGGCGTCGTTCCTGCACGTATACCACCACCTTGGGATGTGCTTGCTGGGATTCGTG

GGGACCAAATATGTTCCAGGTGGGCACGGCGTGATGCTCGGCTTCATCAACTCCCTGGTCCACGCAGTCATGTACTCGTA

CTACCTGGTGTCCATTGTGCGCCCGCAGTGGGTGCGGCGCTGGTGGAAGAAGTACATCACGCAGATGCAGATCCTACAAT

TCTTCCTTCTCATCCTGCACTTTGGACATGTGCTGTTCGAGCCGTCCTGCGAGTACCCGAAGTGGGTGTCGCTCGCCTTC

CTGCCGCACAACATATTTATTCTGTTCTTGTTCTTAGACTTCTACTTTAAGGAATATGTGTACAAA

>Cher_ELOVL5

ATGGCAAATCCTCAAGTCAACATGTCGATAATCGACTCTTACAAGAGCTTTATGGAACGAAATAGTGATCCCCGGACAGA

AGCATGGACACTAATGTCTGGACCGGGCCCCCTGCTCACTATCCTTGCCACTTACCTGTACTTCTGCACGTCAGTGGGGC

CCAGCTATATGAGAGACCGGAAACCATTTGACTTAAAGAACACGATAGTGATATACAATGTCATCCAAGTATTGAGCAGC

ATGTTTTTGGTATATGAGGGTATGGCTTCGGGTTGGTGGAATGACTACAGTTTTTCTTGCCAGCCAGTGGACTATTCAGA

TAATCCCAAAGCTAAAAGGATGGCAGCAGCAGTATGGTGGTACTTCATGGCTAAAGTCGTTGAACTGCTGGACACTATCT

TCTTCGTGCTGAGAAAAAAGAACCGGCAAATTTCGTTCCTGCACCTTTACCATCACTTCATGATGCCCATCTGTGCCTGG

ATCGGAGTGAAGTTCTTGCCAGGTGGTCATGGCACGCTTCTGGGCGTCATCAACTCCTTCATCCACATCATCATGTACAC

GTATTATCTCATCTCTGGACTCGGGCCCCAGTACCAGAAATACTTGTGGTGGAAAAAACATGTTACTACTTTACAGTTGA

TCCAGTTCTGCATTATCTTCTACCACAATTTCTCGGTAATGTTCTGCGACTGCAACTACCCTAAGGTCATCAATTTCTTG

CTTGCTCTTAATGCTGGTCTCTTCTTGTACATGTTCGGTAAATTCTATTACAAGAACTATGTCAAATCCAACACCAAGTT

AGTTCAAAATGGTAGTGTTAAGAAAGAAGTAGTTGTAAATGGTGATCTTAAAATCAATGGTGCGGCCAATGGAGAAGCGC

AAAAGGAATCTGATCATAAAACAGAAGTTAGTAACGGAAAAACAAAAGAATGCTAG

>Cher_ELOVL6

ATGAGCTACCTGGAGAAAGTTGACCGGTACCTGGACTCGCTGAAAGTTGGGAAAAGCGCCATGGTGGACTCATGGTTCAT

GATGTCCAGTCCCATGCCGATCCTTGCCGTGGTGATAGCTTACCTGATCGTCGTGCGAGTGGGGCCGCGGATGATGAAGA

ACCGGCCCCCGCTCAGGATCACCAGACTCATCACTTACTACAACGCCACACAAGTGCTGTTCTCGGTGATGATCGTTTAT

AAGGCCCTCAATTTGAACATATTTCGAGACGGCGTCTTATACGCCGGGTGCAGATATCCGTCGAATACTCAAAATAAGCA

GTTATTGGACCTGGGCTGGTGGTACTTCTTTGCAAAATTCACTGAACTTTTAGACACGGTGTTCTTCGTATTGCGGAAGA

AAAATAAACAGGTGACGTTCCTCCACGTGTACCACCATGCGATAATGGCGCTGTACTCCTGGTCCTACCTGAAGTTCGCA

GCTGGAGGGCAGGGCACCATCCTCGCGCTCCTCAACTCCGCAGTGCACGTAGTCATGTATACCTACTACCTCCTGTCCGG

GCTGGGACCCCAATTCCAAAAGTATCTGTGGTGGAAGAAGTATGTTACTACTTTGCAGTTGGTCCAATTTGTGATAATGC

TTCTCTACTGCCTGTGGACTCACTTCTCTCCTAGATGTCAATTTGCAGTCGGGTTCACCTACTTTATCTCTACGAACGTA

ATCATCTTCCTCTTTCTCTTCCTCAATTTCTATTCTAAAAGCTACAAAAAAAAGAGAATCGCGGAAAAAGAGATCCAAAA

GGAGTTTGAGAGAGCGAGAAATGGGACCAAACAAAATGGAGTCACTGAGGCAAATGGACTTACGGAGAAATGTGGGAAAA

AGAACGGGGTGTGCACAAATGATTGTTCAGATATTCCCAATTTAGAGGAGGTCCCTTGTGAAGTAGATAAAGCGTGTGGT

GACTACATCAAAAAGGGTAAAGTGTTCCTTACTAGACGTACGGCTAAGCAAGCCTACGGACCAGAATATGACTTCGAAAG

CATAACAAAGAAATGA

>Cher_ELOVL7

ATGTCGCTCTCCAACAACTCATTCGCACGGACCTACAACCGACTATTTGTAGAACTAGCAGACCCCCGCACCAATGACTG

GTTCCTCATCACCAGCCCGATACCAGGGCTCACAATCATCTACCTGTACCTCCAGTTCGTACTGAAATGGGGCCCGCGCT

ATATGGCAGACAAGCAGCCCTTCCAGCTTCAGAAGACTATGGTGGTCTACAATTTCCTGCAAGTGCTGGTTAGCTGCTGG

CTGTTCCACGAGGGTTTGGATGCGGGGTGGCTGAGGACTTACAGCTGGAAATGTCAACCAGTAGACTTCTCGGATACGCC

AGAAGCAGTCCGGGTCGCGCGAGGAGTATACGTCTACTTCCTAGCGAAAATGTCAGAGCTCCTCGACACAGTTTTCTTCG

TGATCCGAAAGAAGGAGCGGCAGATCACGTTCCTCCACATGTACCATCACACGGTCATGCCCATGATATCCTGGGGCGCC

ACCAAGTACTACCCCGGGGGCCACGGGACCCTCATCGGGGTCATCAACTCCTTCGTCCACATCATCATGTATACCTACTA

CATGTTCGCTGCCATGGGGCCGAAGTACCAGAGACTGTTTTCGTGGAAGCGTCATATAACTACGCTGCAAATGGTCCAGT

TCTGCATCACGTTCCTCCACTCCTCTCAGCTCCTGTTCTACGACTGTGGCTACCCGCGCTGGTCCGTGGTCTTCACTCTA

CCCAACTCCATCTTCTTCTACTATCTTTTCTACGATTTCTACTACAAGTCCTACAAGACCCCGGAAGAAACGAAAACGAA

CGGCCAAGTCAAAGAAAACGGAGTAAAGGACCACCCAAAATTAGGAAATGGGACCAACGGGATTAAAGATCAATGCGAAG

CGAAAAAGGTCAAATAA

>Cher_ELOVL8

ATGGCGCTAATTTTGAGGACTGCGTGGCAAGGGTACCGATACTTGTTTGAAGATTTGACGGACCCAAGAACGAACACATG

GTTTTTAGTGGCAAAACCTCACCAAGGCGTCGCATTATTGGGCCTTTATCTCATGTTCGTGCTGAAATGGGGACCTGATT

TTATGAAGAATCGACCAGCATACAACTTGGATAAGGTCCTTATCGTGTATAATGCACTTCAGATATTGGTCTGCGCTCGA

CTTTTCGCTGGGTCCTTATTATATGCCTGGGCCTTCCACTACCGATGGATTTGCGAGCCTGTCGACTTCTCGAATAATGA

ACATGCTATCCAGGTAGCAACATACGTCCACGCCTACTTTCTCATCAAGGTCTTTGACCTCCTCGACACAGTATTTTTCG

TTCTGCGTAAAAAATTCAATCAAGTCACTTTTCTGCACGTCTATCATCACTTCGGTATGGTCATGCTGACTTGGGGAGCA

GCGACTTACTTCCCAGGTGGTCACGGCACACTGGTGGGGGTAATAAACTCCTTCGTCCACGTGGTCATGTATGGATATTA

TCTGCTGACAGTGGCCATGCCAAGTTTGAAGAACTCCTTGTGGTGGAAGAAATACATCACACAGTTGCAAATTTTACAGT

TCTTCTGGACCGTAGTGCACATGGGGGCTTTAGTTTTTAAGACGGACTGTGCCTACCCTCGGTGGGTGGCTGCCATATTT

TTACCACAGAACTTGTTTATGCTGGTTCTGTTTATAGATTTCTACATAAAAGCCTACATTAAACCTAATAAAGGGAAGAA

AGAAAATGGCGTCCAAAATGGTAACCAGAACGGCGTCCAAAATGGTAACCAGAACGGCGTCCAAAATGGTAACCAGAACG

GTGTTCAAAATGGTGCACAAACCGGAGTCCAAAATGGTCAAAAGAATGGACTTAATGGTGTTAGTGAAGGAAATGGACGA

GTGAAAGCCGATTAG

>Cher_ELOVL9

ATGGCGCTAATATTAGAGTATATAGACCGTATAAATATTTTTATGGACAAATATGGAGATTCCCGGACAAATCCGTGGTT

CCTGATGAGTTCCCCATTCCCTACATTAATATTATGTTTAAGCTATGTTTATCTAGTCAAGGTTCTAGGACCAAGGTTTA

TGGAAAACCGAAAACCATACAATTTAGGAAATATTTTAGTAGTATACAACTTTTTACAAGTACTGTTCAGTACGTGGCTG

TTTTACGAGATCGGGTTGTCGGGATGGTTCACAGGCAGGTATAACTATCAATGTCAGCCGGTCGACTACTCCAAACATCC

ACAAACAATGAGGATGGTACACGCTTGCTGGTGGTACTATTTCTCGAAGTTCACAGAATTCTTTGATACGATCTTCTTCG

TGATGCGTAAGAAGTACGACCACGTGTCGACGCTGCATGTGATCCACCACGGAGTTATGCCGATGTCTGTCTGGTTCGGA

GTTAAGTTCACACCAGGCGGTCACTCCACTTTCTTCGGTCTGCTCAATACGTTCGTGCACATCATTATGTACACGTACTA

TATGCTCGCGGCGATGGGACCCTCGCTGAGAAAGTACCTTTGGTGGAAGAAGTACCTCACTGCGCTGCAAATGGTACAAT

TCATTGGCATCATGCTGCACGCGTTCCAGCTCCTGTTTATCGACTGCGACTACCCGCGCGCGTTCGTCTGGTGGATCGGC

ATGCACGCCGTTATGTTCTTCTTCCTGTTCAAAGACTTCTACAACCAGTCTTACAGCAAGAGGCCTAGGGTCCGCGCAGC

ATCGCCCAAGCCGGAGATAACAGAAATCCCAGCGCCGTACAAGAACGGCGCGCCGCTCAAGAACGGCTTTAGCAACGGGC

ACGCGAACGGCGCGCCGGCGCTCCGCGCGCGCACCGTGCTGCCGGCCGGCAACTGA

>Cher_ELOVL10

ATGACGAGCGTCTACAACTGGTACCGGGACCTCCTGGACAACAGGAGCGATCCTCGCGTGAAGGATTGGGCGATGACGTC

ATCACCTTGGCCCACGGTGGCTGCGTGTCTCTGCTACGCTTACTGCGCCAAGTGGCTCGGCCCACGGCTGATGGCCAACC

GCAAACCCTTCGAACTAAGGGGCGTCCTCATCGTATATAATCTAGCCCAGACACTCTTCAGTACCTGGATATTCTATGAG

TACATGATGAGCGGCTGGTGGGGTCACTACGACTTCAGGTGTCAACTCGTCGACTACAGTAAGAGCCCAATGGCCATGAG

GATGGCAGCGACGTGCTGGTGGTACTACTTCAGCAAGTTCACGGAATTCTTCGACACTCTGTTCTTCGTGCTCCGCAAGA

AGAACGAGCACGTGTCGACTTTGCACGTCATCCACCACGGGATTATGCCCTTCTCTGTCTGGATGGGGGTTAAGTTCGCA

CCAGGTGGCCACAGCACTTTCTTCGCACTGCTCAACACGTTTGTGCACATCGTGATGTATTTCTACTACATGGTGTCGGC

CATGGGACCCAAATACCAGAAGTACATCTGGTGGAAGAAATATCTCACCGCTTTCCAGATGATCCAGTTCGTGCTAATCT

TCAGCCACCAACTCCAAGTGCTGTTCCGGCCGTCGTGTCAGTATCCGCACGCGTTCGTGTACTGGATCGCCATGCACGGT

TTCCTGTTCCTGTTCCTCTTCAGCGACTTCTACAAGGCGCGGTACACGAAGCGCGAGAAGTCGAACAAGCGGACCGGAGG

ACTCTGCATGGTGGTGGACGAGAGCGGCCCGCTGAACGGCAAAAACGGATACAAGCAAGAGGAGGGTTCGGAGGTCCCGA

GTTCATACGCATCTTCGTCCGCGGACGCTTTCGTGCGCCGGCGCCCCGTGTCCTAG

>Cher_FA2H

ATGGTTGCCGCGATGGATACTCCGCACTTCCCGGTCCAAGTCGACGGCAAGCGCTATGACATAAAAAAGTTCCTTCGCGA

CCACCCCGGGGGGGTGGGAACCTTAAAAAACTATGAAGGAAAGAGTATCCTTCAAGCCATGGAAAAGTTCGGACACAGCA

TCAGTGCGTATCACATGTTAAATGACTTCAAAGTGGACAGTGAGTTAAAAGATAGTAATTTGACGGGTGGAGTGAGTGCG

AACGGTAGGATTATCACAAACGACGAGGGCAGCCGGGATAAGGCGGAGATCGAGTATTTGGAGGAATTGGAGGGCAGACT

AGACTGGTCGAAGCCAATTCTCTGCCAGCTCGACGCCATCGCACCAGACTACGAGCGATGGGTCAACAGCGCGGTCTACC

GGCACTGCCGGATCTTCGCCAGCCCTATCCTGGAAGCCATGACCTTCACGCCCTGGTACCTCGTGCCAGCCTTCTGGATA

CCCATCATACTGTATCTTGGGTACACGCAGTTCGGCGAGCACGTGCTGAATGCAGATACGCAAAACGCGATGTCCCTCCT

CGAGTACGGGTACCACATGGTCTTTGGGACCCTCATGTGGACCATCCTGGAGTACTCGCTCCATCGGTGGGTGTTCCATC

TGGACCCGGGGAGGTCCATCACTATGATCAAGCTGCACTTCCTAATCCATGGCTTACATCATAAGGTGCCATTCGACGGT

CTGCGTCAGGTGTTCCCTCCAATTCCAGCGTTCGTGTTGGCGTCAATAATCTACATGCCGGTGCGGCTTCTGCTATCTTA

TCCACTGATCAAATTGACCGGAGGACTCATCGGTTACCTAATATACGACATGATCCACTACTACGTGCACCACGGCTCGC

CGGAAGACGGCACGTATCTCTACACGATGAAGCGGTATCACTCTAACCACCACTTCATTAACCATGACAGAGCGTTTGGC

ATCAGCAGCGACATCTGGGACCACGTTTTCAAGACATTCGTAACAGTCAGGAGGCTAGGTTTCAGTTTGAGATGGCAGTA

A

>Cher_FAAH1

ATGGAGGTAGGGGTCCAGATAATAGGCATAGTTCTACGACTCATCAACGCGCTGTTGGCTCCGTTATTCTGGCTCTACAC

GCGAGGGCCGCAGCCCCGTTTGCCACCGACGAAGGCCATACATCTGAGGAGCGCTACCGACCTAGCCAGAGCAGTTAGAG

ATGGAGAGATGACAAGTGAGCACCTAGTATCGACGTTCATCGATCGTGTAAAAGAAGTGAACCCACTTCTCAATGCCGTT

GTCGATGAAAGGTTCAGCGCGGCGCTAGCGGATGCTAAAGAGGTGGACCGGCAAATAGAAGAAGCCAAAAGAAGTGGGAG

CTTCAAGGAGCTAGTGGCGAAGAAGCCGCTGCTGGGGGTCCCTTTTACGGTTAAGGAAAGCTGTTCATTGGCTGGCTATT

CAAACGCGGTGGGTTGCCTCGAGAATGCTGGTCGCCGCGCGACCACAGACGGAGCCGCGGTCAAACTGGTGAAAGAAGCC

GGGGCCATACCACTCCTCGTGTCCAACACTCCCGAACTATGTCTGGGGTGGGAGACAACCAACCTGGTCAACGGCACAAC

CAACAACCCTTACTGCCTCACTAGGACACCTGGCGGGTCGTCGGGCGGTGAAGCGGCTCTCCTAGCGTGCGGCGCCTCAG

TATTCGGTGTGGCGTCGGACATCGCAGGTTCAATCCGTATCCCAGCTGCCTTCTGCGGTGTGTTTGGACACAAACCCACG

CCCGGCATAATCCCAATCGACGGCCACATCCCGACTCTGAACGATGAAAACTATCCAAAGTTTCTGACGGTCGGGCCGAT

AACGCGTAAAGCGGAGGATTTGCCGCTGCTACTGAACGTGATGGCGGGAGAGAACCGACACAAGCTCAGGCTGAATGAAC

CTGTTGATCTGAGTAAACTAAAGGTTTTCTACATGACTGAAGCAACCGACTCCATAGCTCTACTTAATGTCGATAAAAGT

ATACAGGATACGATCAGAAACGCTGCTCGGTATCTTGGGAAAGAATGCGGTGCTACTATAAGTGAAGAAAAGTTTGAGGA

GCTAAGAGACTCGGTGGAGTTATCGATATCAGTATTCTTCTCTATGAAGGACATTCCTAACATGTTGCAGGACCCAGCAA

ATCCAAAGCGTGAACGCAGTCTAATACTCGAGCTTATCAAATACTTCCGCGGCGACGGGTCCCGGTCTCTCCAAGGACTG

GGGTTCTCACTCATCGACAAGACCAAACTCTTCATACCGGCCAGCCGGCGGAAACACTATTGCGAGAGAGCTGACGCGCT

GAAGGAAAAGATCACGCGGACGCTCGGCGAAAACGGTGTGCTGCTGTACCCGGTGTTTACCGACACGGCACATCACCACC

ATCAGGTGTTCGCGCGCGCGTCCGGTGTGGTCTACACAATGCTGGTCAACGTGCTTGGCCTGCCCGCGGCCGCCGTGCCC

GCCGCCGCCGCCCGCAGCCAACTGCCCGTTGCCATACAGGTTATAGCGGGCCCATATCAAGACCGGCTATGTTTGGCGGT

GGCGAAACAACTCGAAGTCGGTTTCGGCGGCTGGCAACCACCCTCTTAG

>Cher_FAAH2

ATGCTGAACGGCTTCATGCGATGGCTGCTCCGGTTCCTGGTGTCCCTCATAGCTATCGGAGTCCGGCCCCTCACATACCT

CCTCACCATCAGGAGAACGAGGAAATGCCCTCCACCAACCAACCCCATACTCTTCAAGTCTGCGACTACGCTCACTATGA

TGATTAGGAATAAACAGATCACCTCAGAAGAAGTAGTGGCAGCGTACATAGAGAGATGCAAAGAAGCGAATCCCTACATC

AATGCTGTAGTGGAACCACGGTACGACCAGGCCTTGAGAGAAGCCAGGAGCATAGACAAGATGATAGCTTCTACCGACAG

GACACCTGAAGAGCTGGGGAAGGAGTATCCATTGCTTGGAGTTCCCATGACTGTGAAGGAAAGCATAGCTGTTGAAGGCA

TGAGCAACGACTGCGGAACCGTTCGTCCGACCAGAATCCCAGCGAGCAAAGACGCTGCCATCATCAGCCTAGCCAGAGCC

GCGGGGGCCATACCCATAGCTGTCACCAACACCCCCCAGTTCTGCATGAACTGGGAGACTTACAACAACGTCACGGGACT

CACTATGAACCCTTATGATCAGAAGCGGACGACTGGTGGGTCTTCGGGTGGAGAGGCTGCCTTGATATCGTCCGCTGCGT

CAATAATGGGCGTGGGATCCGACATTGCGGGTTCCTTGAGACTGCCTCCCATGTTTACGGGTATCTTCGGGCACAAGCCT

ACTCCGAGATTACTGTCCGTTGTAGGTCATGTTCCAGACTGCCCTGCGTCTGATTTTGAAGATTATTTCGCTCTGGGACC

TTTAACTCGATACGCTGAAGATCTGAGTTTACTTCTCAAGGTCTTGCGACAACCTGGAGCACCGGACGTGCCACTAGACA

AGCCCGTAGACGTTTCAAAGTTGAAGTTCTACTACATGGAGGGGGACAACAGTAACGTCACCGACAAAATTGGCTCTGAC

GTCAAAAAGGCGATGGAAAAGGCAAAGGCGTACATGAAAAGTACTTACAACATTGAAGTTAAAGAGCTCAAAATAAAGAA

CATCGAGCACATTTGGGAGATCAGCGTAAGAGTCCTAATGAACATCCGCAACGTTCAGAACATCTACACTGACCCGGAAA

AAAGGGACCAATGGGTGTCCGTATGGCCTGAAGTACTCAAGAAAATGGTCGGTCTCTCTGACCACAACTTCACGTGTGTG

GCGTATGGACCTCTGCAGAAATTCTTCGACTCCCTACCTAAGAGCTACTACGCGAAACTGTTGGCTATGTTTGAGGAAAT

GAAGAAAGATTTTGAAGCTGCCCTATCTGACGACGGCGTATTACTATACCCAGCGTTCCCTTACCCAGCTCATTTACACT

ACAGAGTGTATTACAAGTTCCTAAACTGCGGGTACCTGACCATTTTCAACGCTTTAGGATTACCAGCGACGGCGTGTCCT

ATAGGACTTACTGACAAGGGCCTACCAGTGGGCCTGCAGCTAGTGGCTAACAAATGCAATGACCATCTGACGATAGCTGT

TGCTAAAGAGTTTGAGAGAGCATTCGGTGGCTGGACTCCACCCAACAAAGAAATGATTAGCGTCTAA

>Cher_FAAH3

ATGGCTACGAACAAAGTCGTAAGAGGAACAGAACAAAATAAACGCGGAAAAACAACAAAAATCCTAAAAGGAATGGCGTT

CAACATGTTCAAGCATGTCTTCATCTTCATCCGAGGATACGTGGAGCTGATCATCGACCTGATATTCGCGTTCGTATGGG

ACGGGGAACGGCAAGCCGTGCCCGACCTGGAGGCAAGGCACGCAATGCTGATGGAGAGTGCTGTGGAACTGGCTAAAAGG

ATTCGGAATAAGACTTTGAAGTCCGAAGAGCTGGTGCGGGCTTGCGTGGAGAGAATTCAGATTGTTAACCCAGTTCTTAA

CGCAGTGGTGGACCAGAGGTTTGAAGAGGCGCTGAAGGAGGCACAAGAGATTGATAAGCAGATCCAGAGTGGCCTGCCTG

ATAAGTACTTTGAGGAGAGGCCTTTTCTGGGCGTGCCATTCACGGCTAAGGAAAGCCACGCAGTGCGCGGCCTGCTGCAC

CCTCTCGGCATCGCGGCGCGAGCTAACGTGCGCGCCACTGAAGACGCGGAGTGCGTGCGTCTCCTGCGCGCCGCTGGCGC

CCTGCCCGTGGCTGTCACCAACGTGCCTGAGATTAACAAGTGGCAAGAGTCCCGCAACAACGTGATTGGACAGACTTGCA

ACCCGTACCACACGGGGCGCACGGTGGGCGGCTCCAGCGGCGGCGAGGGGGCGTTGCACGCCGCGCTAGCCACGCCCATA

TCCTTGTGTTCGGACATCGGCGGGTCTACGCGTATGCCTGCGTTCTACTGCGGATTGTTCGCGCTCAACCCCACTGCCGG

CTACACCAGCCTTAAAGGGTCTGCGCTGCGAACCGGGCTGGACCCAACGATGGCGTCGATCGGATTCATCAGCAAACACC

CTGCGGACCTGGCGCCGCTCACCAAAGTGGTCGCCGCCGACCAGGCCGGGAAACTCAACCTGGACAGAGTTGTCGATTTA

AAGACTGTGAAGGTGTACTACGTGGAAACAGCTCAAGACCTGCGCGTGAGTCCCGTCAGCTCGGAACTAAGGGAGGCCAT

GAACAAAGTGGTGTCAAAACTGTCAGAGCTCGGTCGAGTGACAGCCTCATCCCCTCAACCATACTACCACCCCGGGTTCG

AGCACATGTTCGCGCTGTGGAAGCACTGGATGACCAAGGAGACTGAAGACTTCCCGAGAATGCTGACCGATAATAAAGGA

GTCGCTCATGGCCTTACGGAGCTTGGGAAAAAGCTAATTGGCACATCGCAATACACGATCGCTGCCATCTTGAAGCTCTT

GGACGAGCAAGTGCTTCCGCCTGTCAACGCTGCGTGGGCTGAAAAACTTACCGACAAGATGCTCGACGAACTCACGACGG

TGCTGGGCGACTCGGGCGTGCTACTATTTCCGTCAGCACCGATGGTGGCGCCCTACCACTACTCATTGCTGCTGCGTCCC

TTCAACTTCGCCTACTGGGGCGTGCTCAACGCGCTCAAGTTTCCTGCCGCACAGGTCCCTCTCGGTCTGAACTCGGCCGG

CATCCCCCTGGGCATACAGGTAGTGGCGGCGCGCGGGCAAGACGCGCTCTGCCTCGCTGTCGCTAAGCATCTGGACTCGC

TGTTCGGCGGCTACCGGCCGCCCTGCACCATACTGCACTGA

>Cher_FAAH4

ATGGCCACATCAGATATTACATCGAGTAAACCGCCCGGGATGAGGGTCAAAGTGTTACATGTAGTTAGATTGGTCCTCGC

CGGTCTCGCCAGGTTGTTCTGTTCCCTGTACTATGGAAAGACGGGAGAGAAGTTTCCGCCGATAACCGCCGACATCCTGA

AACTGCCGGCTGTCGAGGTCGCAAGCAAGATCAGGAATAAAGAGATCACCAGTGTGGAAGTGCTGGAGGCTTGCAAACAA

CGCATCAAAGACATAAACTCGGCTCTCAATTGCTTTGTCGAAGATCGTTTTGAGCTGGCTTTGCAAGAGGCTAAAGAGGC

CGACGAACTCGTTCGAAGTGGGGCCATGACCGTCGAGCAACTGGCGAGAGAAAAGCCCTTCTTAGGCGTGCCTTTCACCA

CGAAAGACTGCATCGCAGTTAAAGGCTTACATCACACGTCGGGAGTCGTTCTTCGCAAAGATGTCATCGCTAAAGAAGAC

GCTGAATCAATAAAGTTGTTGCGAAATAATGGAGCCATTATTATCGGACTAACCAACGTGCCGGAACTTTGCATGTGGTG

GGAGACCCACAACCACATCCACGGGCGGACGAACAACCCCTACAACACGACCAGGATTGTTGGGGGCTCGTCTGGTGGCG

AGGGGTGCCTTCAGGGCGCCGCGGGCAGCCTTTTTGGCGTTGGTTCGGACATCGGAGGCTCTATCCGAATGCCAGCTTAT

TTCAACGGTATTTTCGGACATAAGCCATCGCGACTTATAGTTTCCAACGAAGGCCAGTATCCCACGCCTCAAACCGAGCT

TCTGAACTCATATCTAGGTCTCGGACCAATGACACGACATGCGGTTGATCTCAAACCCCTTTTGAAGATTATGTCAGGCG

ATAATGCAAGAAAACTCGACCTAGATAAAAGTGTCGACGTCGGGAAACTAAAAGTCTTCTATCAGTTGAGTAACAACGCT

CCCATGACGGACTCTGTTGACCCAGAAATCACAGCAGCGTTGAAGAAAGTTGTCGAATTCTTTAGCATCAAGCACAAGAT

ACAGGCTGAGGAAAAAAAGTTCAAATTTCTGCAAAAATCCCTCCCAATCTGGATGACTACTATGAAGAATAAAGAGCCTT

TCGAAAGCTTGATAATGGGAAAAGAAGGCACCGTAGCGATTGTTCTGGAAATCTTCAAAAATCTCATTGGATGTTCAGGC

AACACGATGATCGGTCTTTTCACGGCGTTGATGGACCGTTCTGGTGCAGAGATCGGCAGTGACAAATACAACTACCACCT

CAAATTACGTGATAATTTAGAGAAGCTTTTCGTGGAGATGCTCGGAGACGACGGTGTGTTTCTGTACCCCACGCATCCGA

CGCCGGCCCCCTACCACAACGAGCCATTGATCAGACCTTTTAACTTTAGTTACACTGCCGTTATAAATTGCCTTGGCCTC

CCCGCAACGACAGTTCCCCTGGGGTTGAGCAGCGAAGGGCTACCTATTGGAATTCAAGTGGTAGCTAATCATAATAATGA

CCGGCTATGTCTAGCGGTAGCTGAGGAACTAGACAAAGCTTTTGGAGGTTGGGTGGAACCGAGGCACTGA

>Cher_FAAH5

ATGGCGTGCACAACATTAAAATCATTTTTATGTTGCCTTCGGGTCTACATAGATAAAATGATAGATTTCTTCTTCGGGCT

GTATTGGGACGGCAAGAAAGTGTCCATACCGCCTTTGAGCAAGGAGCATGCATTTCTGGCTGAAAGCACTGTGACTCTGG

CGAGAATGATAAAAGAAAAACAGCTGAAGTCGGAAGACTTGGTCAGAGCTGTCATTAAGAGGATAAGAGAAGTAAACAAC

TTAGTGAACGCTGTCATCGAAGAGCGGTATCACGAGGCGATAGAAGACGCGCGGGCGGTGGACGATCTCATAGCAGCTGG

GCTGCCTGAGGAGGAAGCTGCCAAGAAACCGTTCTTAGGTGTTCCCTTCACGACCAAGGAGAGCCAGGCCATCAAGGGTT

TCCGCTACACCATGGGCCTGTGGTCTCGCCGGGAGATGCGCGCCGACGAGGATTCCGAAGCCATCGTCAGGCTCAAGGCA

GCCGGTGCCATCCCCTTGGCTGCTACTAACCTGCCGGAGCTGCTGATCTGGCAGGAGACCCGCAACCCCGTATACGGCAT

GACGAACAACCCGCACCACGCGGGCCGCTCGCCAGGGGGCTCCAGCGGCGCTGAGGCGGCCCTCACTGCCACCTACGCTA

CACCTATCAGCTTGTGTTCCGACTTGGGAGGATCCACCCGTATGCCTGCTTTTTACTGCGGAATGTTCGGCCACCACGCG

ACTGCCGGAACCACCAATGGCAGAGGAGTCATTTTCCGCAAGGGTGACGAGGAATCTATGCTCAGCTTAGGGTTCATAAC

GAAGCACGTGGAAGACTTAGCACCTTTGACCAACATTATTGCTGGGGACAAGGCCCCACTTCTCAAACTCGACCGCAATG

TCGATATTAAGAACATAAAGTTCTACTACTTGGAGTCTGCTAACGACTGTCGGCTCAGTTCCATCAGGCCAGAGCTCAAG

GATGTTATGACGAGCGTTGTATCAAAGCTCGGCAAGGAAATGCCTCCGCAGAACAGCCCTGAGGCTTACCAGCACGAAGG

GTTCGACCACATGTACCAGCTATGGTCTTACTGGATGAGCAAAGAGCCAGAGAACTTCGCCAGCCTGTATAACAACTACA

AGGGCGAGGCTAACGGATTCGTGGAGCTTCTTAGAAAGATATTTTGCCTCAGCAAGCACGGACTGTCCGCTGTGATCCGG

TTATTCGAGATCCAGATCATGCCGCTATTCCCTGCCTGGGCAGACAAACTCACTACTGAACTGAAACAAGATCTTTTTAG

TAAACTGGGCGACAACGGCGTCCTGTTATTCCCGAGCGCTCCTCACCCCTCCCCGTACCACTACTCTTGCTTCCTGCGAC

CATACAACTTCTCTTACTTTGCCGCCGTCAATATGCTCAAGTGCCCTGCTACTCAGGTGCCTCTGGGTGTGAACAGTGAT

GGTCTGCCACTTGGTATTCAGGTGGTAGCAGCCCCGTACAACGACGCGCTCTGTCTCTCCGTCGCCAAGTATTTAGAAAA

AGAATTCGGCGGCGCCGTCATGGCTTGTAAAAAGCAATAA

>Cher_FAS

CTGGTGCACGCGGGCACGGGCGGCGTGGGGCAGGCCGCCATCGCCATCGCGCTGCACGCCGGCTGCACCGTGTTCACGAC

GGTGGGCACGGCCGACAAGCGCGCCTTCCTGCGCGAGCGCTTCCCCGCGCTGCCCGAGGCGCACGTGGGCAACTCGCGCG

ACTGCAGCTTCGAGCAGCTGGTGCTGCGGCGCACGCGCGGGCGCGGCGTCGACCTGGTGCTGAACTCGCTGGCCGGCGAC

AAGCTGCAGGCGTCGCTGCGCTGCCTGGCCGTGGGCGGCCGCTTCCTGGAGATCGGCAAGCTGGACCTCAGCGCCAACTC

GCCGCTCGGCATGGCCGTGCTGCTCAAGAACACCACCGTGCACGGCATCCTGCTGGACGCGCTGTTCGACGCGCGCGGCG

ACCACCCCGAGAAGGCCGAGGTGGTGCGCTGCGTCACCGAGGGCATCGCCCAAGGCGCCGTGCGCCCGCTGCCCGCCACC

GTCTACGCCGACTCGCAGCTGGAGCAGGCGTTCAGGTACATGGCGACGGGCAAGCACATCGGCAAGGTGCTGATCCGCGT

GCGCGAGGAGGAGGCGGGCGCGCGCGCGCCGCAGCCGCGCCTGCTGTCCGCGCTGCCGCGCACCTACATGCACCCCGCCA

AGAGCTACGTGCTCGTCGGCGGGCTGGGCGGCTTCGGGCTGGAGCTGGGCGAGTGGCTGGTGGGCCACGGCGCGCGCACG

CTCGTGTTCAACTCGCGCAGCGGCGTCAAGACCGGCTACCAGGCCTGGTGCATCCGCAGATGGCGCGAGAAAGGCGTGCG

CGTGGTGGTGTCGACGGCGGACGCGACGTCGGCGGCGGGCGCGCGGCGTGCAGAAGATCCTGCAGGACTACAAGTCGCT

>Cher_FAT1

ATGGCGGTGCGGCCACAGCACATGAACGACGGAGGGATCATTGAGCGCCGGCTACGTCCTATTTACGATTGGCTGGACAA

TGGGAATAATAAGAAAGCGCTGCAGGAGGCGGAGAAGGTGCTTAAAAAGAGTCCTTCGCTGCAGGCGGCCCGGGCTTTGA

AGGCTCTGTCGCTTTTTAGGTTAGGAAAAGCACCGGAGGCCCACGGCGTGCTGGAAGCCCTAGCGGACGAGAAGCCGTGT

GACGATACTACGCTGCAGGCCATGACGATATGCTACAGGGAGTGCCAGCAATTGCACAAAGTGTGCTCCCTGTACGAGGC

GGCGGTGAAGGCTGACCCGACGAGCGAGGAGCTCCACTCGCACCTGTTCATGTCCTACGTCCGTGTCGGGGAATACCGGG

CACAGCAACGGGCCGCGATGGTGCTCTATAAGTTTGCACCCAAGAACCCGTATTACTTCTGGGCCGTGATGAGCATTGTG

TTGCAGGCGAAAGAATCCGAGGACGCCGCGAAAAAGGGCATCCTCCTCGCTCTAGCGCAACGCATGGTCGACAACTTCAT

ATCCGAGAACAAGATGGAGGCTGAACAAGAAGCCCGACTCTACATCATGATCCTAGAACTGCAGGAAAAATGGGAGGATA

TCCTTAATTTCATCGAGAGCCCGTTGTACGCGAAACTCCTCCCTGGAGCCACGGCCCAAGCCTGCATACCTTACTTGAAA

AAACTCCAGCAATGGCGACGTCTCAACCTCATATGCAAGGACCTTCTCTACGACAACCAAGACCGATGGGACTACTACAT

ACCCTACTTCGAATCGGTCTTCCAACTCATGAAGTGTTCGGACAAGAACGACTTGACCGTAGACGATACGGCTGAGAAAT

GTCACGAGTTTATCTGTCAATTAGTGGAGAGTATGTCGTCCGGGAGGGTGCTGCGGGGGCCTTATTTAGCTAGACTGGAA

CTGTGGAAACGTTTGTCAGTTGACGGAGACCCAACGGAGCTCCTTGGCAGCGGGGTGGCGTTGTGCGTGCAGTATTTGCG

GGTGTTCGCGAACAAGCCGTGTGCCGTGCCGGATCTGAGGCCGTATCTGGAGATGATACCGCAGAGCGAGAGGGAGGAGC

ATTGCAGGGATTTCCTCACGTGTCTCGGGTTCGATGAGAATAGTGAGCCTAGTAATCCGGACGACATCCAGCGCCACATC

TCCTGCCTCTCGGCGTGGCGGCTGACGGCGGCGCCGCTGCCCGCGCCC

>Cher_FAT2

ATGAATATTCGCTGTGCACGCCCGAGTGACCTGATGAACATGCAGCATTGCAACTTGCTTTGCTTGCCAGAAAATTATCA

GATGAAATATTACTTCTACCACGGACTCTCCTGGCCACAGCTCAGCTACGTAGCTGAAGACGAAAAAGGACACATTGTTG

GCTATGTGCTGGCTAAGATGGAAGAGGATGGCGAAGACAACAGACACGGGCACATAACGTCACTGGCTGTGAAGCGGTCG

CATCGCCGGCTGGGGCTCGCCCAGAAACTTATGAACCAGGCTTCACTGGCTATGGTGGAATGCTTCCAGGCAAAATATGT

GTCCCTCCACGTCAGGAAAAGCAACAGGGCTGCGTTGAACCTCTACACAAACTCGCTAGGCTTCAAGATCCTGGAGATCG

AACCCAAGTACTACGCTGATGGCGAAGACGCTTACTCAATGATGAGGGACTTGAGTTCATTCTCCGCAGACAGCAAACCA

GAGACTCAGGCGTCAGATAATTTGGAAATCAAATCTGATTCTGCTGTTATATCGCAGTGTTGA

>Cher_FAT4

ATGCCACCAAGTAATCCTTTACCGCCTAAAGAAAATGCGCTATTTAAAAGAATTTTGCGTTGCTACGAACATAAACAATA

CAAAAATGGCTTGAAATTTGCCAAGCAAATCCTGTCCAATCCAAAATTTGCGGAGCATGGAGAAACATTGGCAATGAAGG

GCCTAACACTAAACTGCCTTGGCCGCAAAGATGAAGCTTATGAGTATGTCCGCCGAGGCCTCCGTAATGATCTCAAGTCC

CCAGTTTGCTGGCACGTTTATGGTCTTCTGCAACGGTCTGACAAGAAATATGACGAGGCCATCAAATGTTACCGGAATGC

TCTCAAGTGGGAAAAGGAGAACATACAGATTCTTCGAGATCTCTCTCTCCTTCAGATCCAAATGAGAGATTTAGAAGGAT

ACAAGGACACCCGCTACCAACTCTTCATGCTGCGCCCGACACAGAGAGCATCCTGGATAGGTTTCGCGATGAGCTACCAT

CTCCTCGGAGACTACGAGATGGCCAACAGTATCCTGGACGCCTTCCGTACCAACCAAATGAAAGGCCCGTACGATTACGA

GCACTCGGAGTTGCTGCTGTACCAAAACATGGTGTTGGCCGAGTCCGGGCAGTTTGATCGGGCGCTCTCGCATCTACACA

AGTTCCAGACGCAGATACTTGATAAGTTATCCGTGAAGGAGACTAGTGGCGAATATTATTTGAAGTTAAAGAGGTTCAAA

GAGGCTGAGGCGGTGTACGAAGACTTACTGAAGAGGAACCCTGAAAATGTTATGTATTACCATAAACTTATAGAAGCTAA

ACAACTAAGTGATCCTGATGAAAAAGTGGCCTTCTTTGATGTATATAAGAAAGAGTATCCCAAAGCAATAGCGCCCCGCC

GGCTGCAGCTCTCGGAGACGGGCGCGCCGGCCGCGTTCGCGCGGCTCGGGGACGAGTACCTGCGGCACGGGCTGCACAAA

GGCATCCCTCCGCTGTTTGTTGACATTAGATCCCTATACACGGACAAAGTCAAAGCGGAAACGATAGAGAACCTGGTCCT

CCAGTACATAGAGAATTTATCCAAGTCCGGAGCATTTAGCTCAGACCCGAACGAAGTGAAACAACCTGCCAGTGCGCTGT

TGTGGGCTTACTACTATGCTGCGCAGCACTTTGACTATAAAAAGGATACGGACCGGGCTCTGAAGTACATTGACGCCGCG

ATAGAACACACGCCGACTTTGATAGAGCTGTTTATTGTTAAAGGAAGGATATTTAAGCACGCCGGCGATCCCATATCCGC

GTACCAGTGGCTCGAAGAGGCGGCGGCGATGGACACGGCCGACCGCTACGTCAACAGCAAGTGTGCTCGCTACATGCTGC

GCGCGGGGCACGTGGCGCGCGCGGAGGAGATGTGCGCCAAGTTCACTAGAGAAGGTGTGCCAGCGACAGAGAACCTGAAC

GAGATGCAGTGCATGTGGTTCCAGACGGAAGCCGCGACGGCGTACCAGCGCGCGCAGCTGTGGGGAGAGGCTTTGAAAAA

GGCGCACGAGGTTGATAGACACTTCTCCGAAATAATGGAAGACCAGTTCGACTTCCACTCCTACTGTATGCGCAAGATGA

CACTCCGTTCCTACGTGGGCCTCCTCCGGCTCGAGGACGTTCTGCGCGCTCATCCGTTCTACTTCCGCTGCGCGCGCGCC

GCCATACAAGTATACCTGAGACTGGACGCTTTCCCGTTGCAAGACGTGCCGCAAACACAGGAGCCCGATACAGAGAACCT

GGCGCCGTCCGAACTGAAGAAGTTGAGGAATAAGCAGAGGAAAGCGAAGCGCAAAGCTGAGCAGGAGAGCATGCTGGCCG

CACAAGTTCAGGTGAAGCGCGAGCAGCACCACAAGGCGCGGCAGCAGCAAGAACAGGGCGACCCGGAGGCGCCGCAACTC

GACGAACTCATACCCGACAAGCTGGCAAGGGCGGACGATCCTCTAGAACAAGCGATAAGGTTCCTGCAGCCGCTCCGGAC

GCTTGCCAACGATAGGATAGAAACACATTTAATGGCCTTCGAAATATACTATAGGAAAGAGAAACCCCTCCTCATGCTGC

AGAGCATAAAGCGCGCGTGGCGGCTCGACAGCGGCCACCATCACCTCCACGACTGCATGGTGCGGTTCCGCGGCTTCCTC

GACGAGCACCCCGACCAGCCCGCCGCCGTCGCGGAGGTCGTCGAGAAGGAGACACGGGAGTCGGTCCGCGGTCGCACGGC

GCGGCAGATGGCGGAAGAGTTCATGACTCACTCGGCGCAGCAGTCCCAGAGCGCAGCCCTGTGGGGCGCGCGCGCGCGCT

GC

>Cher_FAT5

ATGGCTGTCACGGGTGCCAGGAGTATTGTGAATCTGAAGAAGTCCCACTTCGAACATGAGAATGCTAGGATCGATAAATA

TACGCTGGCGGACGAGCTGCCGGCGCAGCTGTCGGAGCTCGGCCTGCGCCCGCGCAGCATGCAGAAGCCCGGCTATACGC

TGCGCAAGCTCTCCGCCAGCGACAAGGAGATCACGCTCGAATTCTTGAGACGGTTTTTCTTCCGCGATGAGCCCATGAAC

TTGGCAGTCCAGCTGTTGGAGACAGCAGACTCCCGCTGCCCTGAGCTAGAAGACTATGCGTCCTCGTCGCTGACTGATGG

CGTGTCCGTCGCTGCCTTCGACGACCTGGGAGAGATGGTTGGAGTGGTCGTGAATGGGGTTGTCAGAAGAGAGGAAGCCG

ACTACACGGATAAGTCCGAAGAGTGCCCCGACCACAAGTTCCGGCGGATCCTCAAGGTGCTGGGACACCTGGACCGCGAA

GCCAAGATCTGGGAGAAGGTGCCTGAGAACTGCAACAAGGTCCTGGAGATCAGAATAGCGTCGACACACTCTGACTGGCG

CGGGCGAGGGCTTATGCGGGTGCTTTGCGAAGAGACAGAACGCGTGGCCCGTGCGATCGGCGCAGGCGCCCTCCGCATGG

ACACAACATCGGCGTTCTCCGCCGCCGCCGCTGAGCGGCTCAACTTCCGCAGCGTGTACTCAGTTCTATACGCAGACCTG

CCCGACGCGCCGCAGCCGGAATCTCCGCATCTTGAGCCTAGAGTCTACATTAAAGCTCTGTGA

>Cher_FAT7

ATGCCTGTGCAGGTGTACCCGAGTCCGGGGGCGGCGCGCGGCGTGTGGGCGCTGCCGCAGCCGCCCCCCTGCAGCATGGC

GGCAGAGGACTGTCTACCGCCCTGTTCGCCGCTATCGCCCGATGCGCCCGCTGCACCGCCCCGTACCATCACCAGCTTTT

ACCACCATTCGGATCTGCATCAGCTGATCTTTGAGGCTGTGAGGTCCAGCGAGGTGTCAGAGATCGAGCGTCTAGTGGAG

AAGCTGGGTGCTGAAGTGCTGAGCGCACGCGACCAGCATGGGTACACGCCTGCGCACTGGGCTGCACTCGATGGCAGCGT

AGCAGTAATGCGTTACTTGGTGGAACGGGGCGCGCCGGTTGACCTGTCCTGCCTGGGCACTCAGGGTCCTCGACCAATCC

ATTGGGCCTGTCGCAAAGGCCATGCGTCAGTGGTGCAAGTGCTGTTACAGTGCGGGGTTGCAGTTAACGCAGCTGACTTT

AAAGGTTTAACCCCTCTCATGACAGCCTGCATGTACGGCAAAACCGCTACAGCAGCCTACCTCCTAGGCATGGGAGCAGC

CACCAGGTTATCAGACATCAATGGAGATACCGCCCTCCATTGGGCTGCTTACAAGGGACATGCAGACCTGGTGCGATTGT

TGATTTATTCTGGAGTCCCGCTGCACTGTACGGATAACTTCGGCTCTACACCGCTGCACCTGGCTTGTCTGTCGGGGAAC

CTGACGTGTGTGAGATTGCTGTGCGAGAAGGTAAATAAATATGTAAAAGCCGAGCTAGAGCCTCGCGACAAAAACGGAAA

AACCCCCCTAATGCTGGCACAAAGCCACCGCCACGCTGAAGTAGTGAAGCTACTGACCAAGGAGATGAAACGCAAGTCCC

ACTGGATGCCGCCGCTGTCGGAGTTGTGGGCTCTGCTGTTTGGCGGCGCTGGGGATTCTAAGGGACCACTGCTGTTTTTC

TTGGTTTCTGTGCTGCTGTGGGGGTACCCTATGTATGTTTTTAGATGCATCCCCCTAACCTGGACAACGCTCCGCCTCTC

CCACTACTGCTTCCTCTACTGGAACGCCATAATGTGGCTAAGCTGGGTCATCGCCAATCGCCGTGATCCCGGCTACATAC

CGCAGAACTCTGACACGTACTACCGCGCCATCCGGCAGATACCGTACTATGACAAGTGGAAGAAACGGAACGTGGTGCTG

TCGCGCTTGTGCCATACTTGCCGCTGCTTGCGGCCGCTGAGAGCCAAGCACTGTCGCATCTGCAAGCGCTGCGTCGCATA

CTTCGACCACCACTGCCCCTTCATCTACAACTGCGTCGGCGTCCGCAACCGAATGTGGTTCTTCCTGTTCGTGATGAGCG

TCGCCATTAACTGCACGCTGTCCATCTACTTTGCGTGCTACTGCCTGCTGCTGGAGGGATTCGGGCTGCTGTATTTGCTG

GGGCTGCTGGAAGCACTCACGTTTTGCGCGCTCGGATGGATTTTGACGTGTACTTCTATCCTCCACGCCTGTATGAATTT

GACGACAAACGAGATGTTCAACTACAAAAGATACCCCTATTTAAGAGACAAGCGAGGCCGATACCAGAATCCCTTTTCTA

GGGGTCCTATAATGAATTTGTTCGAGTTTTTCGTTTGCCTACCAGATAAGTGCGACGAGCAAGATTTCTTCCACGAGGAG

AGCATTTAA

>Cher_FAT9

ATGACAGGTCGCTGTCAAAACACAGAGTCAAACAATTTGCTCAAAAATTGGAAAATTGTTGGAAATATTTCCTTTAGTGT

ATACCTATACCTATTCAATAAAAACATTTACATCTATCATCAAGCAATGAGTAACCCACATAATACCAGCCAGCTACCGG

AATGTTTGAGGGAGGATGAAAATGCAGATGGGAAGACGAAGCTTAGAAAATGGCTGAACATGAATTTCAGAATTGAAATG

ACAGACGGAAGGGTCCTTATTGGTGTCTTTCTGTGCACAGATCGAGATGCTAATGTAATATTAGGTGCTTGCTCGGAGTA

TCTCAAGGGCAATGACGGTGAAACCGAAGAGCCCAGAGTGTTGGGTCTCGTCATGGTCCCCGGCCGGCACATAGTCTCCA

TACAAATAGACGACATGACTGCACATCGCAGAAGTGCTCCCATAATGAATGACTCTATGTGGCTTTAA

>Cher_FAT10

ATCAATTTGGGCGTTAGACACAGGATAAGAAAGCCCAAAGTGATGAAGAATAAAATGTTCAAAAATGGTCCCTATATGAA

TAAGTCTGACACAGCGGTTTTGAATAACAGCAGCGTTTCGAATATTTCCGTCAACAATTTGAGTATGAACAACGTTTCTA

TGCTGAGTCAAAATTCCTCACAAGAAATGGACAAAGAAAAAGCCGACCCCTTCAGCAAAGAAAAACAAACCATTGAAGCG

CTACTCAGCCAATGGACGGAAGAAGAAGTGCCCGAGTCCGAGATCCACAAACCGGAGCTCCCCTGTTTCAACAGCACGGT

CATCGAGGAGACCAACCAGATCTTCCAGCCGATCACAGCGATCCCCGTCTCAGGACAAGAGGCACCGGAGGGCGAGGCAG

TGATTATTGAACTCGGAGCTGGTGCGAAGCCAGGGTTGCCTGATGATGTGACGATGACGGAGCTCCATAAGGCGAGTCAC

GGTGATGTTCCGAGTGCCAAGTTCCTGCCGGTTGACGGAGGGTTTATACTGGTCGAGGATGCTGTGAAAGCACCAACCCA

AGAAGACCTGACAGACTTGGACGAGATCGAGCGCGAACTCAAAATGCTCGACGAACAGATACTACTGATGACGCAGGTCG

ACGACCCCGCGCCTGCTAACCTCCCGCAAGCCACCACAGCCCCTACAACGGAGGCTGCCAAAGACCAAGCTGACAAGCTG

TTCCCCATCTTTAGCAAACCCAACCCTGGGACCAGCACACCTAGCTCTAAAGCGACAGCTACCAAAAGCAAAAAGTCGAT

ATTGAAGGACGGCAGCGACCAATACGTCATCGACGCGGGACAAAAGCGGTTCGGAGCTACGCAGTGCACGGAGTGCGGTG

TCATTTACCAGATTGGCGATCCTCAAGACGAGCACGATCATTTGGTGCACCACAACGCGACGGACGTA

>Cher_FAT11

ATGGAGTCGGAAATACTCGAAGTGCTGCCTCTACATAATTATCCTGAGTTTATGAAGGATACCTGCGACTTGATAAATGA

TGAGTGGCCCCGCGGCGAAAACGCCCGGCTGATGTCCCTACAAGCCTCGTGTGACTACCTACCGACAAGTTTTATTTTAG

TTAGAGATAGAAAGCAAGTATTAGGCCATTGTAAACTAACCGCTATACCCAGTATTCCAGAAAGTTGTTTTATAGAAACC

GTAGTCATAAGTAAAGCTTTGCGTGGGAAGAAACTAGGAACTTATTTGATGAAACACGTCGAAGATTACTGCAAGAATGC

ACTGAAACTAAAGATGGCGCATTTGTCGACGAAAGGACAGGAAGAGTTTTATGCGAAACTTGATTACGAGATTTGCGCAC

CAGTGTCCATATACGGCGGTTATTTGCCAGCCAATTCTAAACTTAGACCTAGTAGCCATACGATTGAAAATTCAGTGCCA

GAAATGAGCGTGCCGGTTAGCGGCGCGCCGCCGCCACCGCCGCTCCCACCGACGTACGAAACTGCTTGGAACAATAATAA

TAACAACACCATTAAAACTACCAAGACCTATATGTTTAAGTATTTGTAG

>Cher_FAT14

ATGGTGAAAAAGAAGATTGATAACCGTATCAGGGTTATGATAGAAAATGGAGTCAAGCTGGGACACCGTACAATGTTCCT

GCTTGTCGGAGATAAGAGTAGAGATCAAGTGCCAATTCTATATGATATTCTGGTGAAATCAACTGTCAAATCCCGACCTA

CGGTTTTATGGTGCTACAAAAACAAAGATGAAGCTATAAGCAATCATGGCCGCAAACGGGCAAAGAAAATCGCAGCAGGG

AAGCTGGAAGTGTCAGAAGAATCTCTGTTTGATGCGTTCAGAGTGGCGACTACGATACACGGACGGTATTACTCTGAGAG

TCATGCTATGCTTGGTCAGACTTATGGAGTGTGTGTGTTACAGGACTTTGAGGCCCTCACTCCTAATCTAATGGCTCGAA

CAATAGAAACAGTGGAAGGTGGTGGACTCATCATCTTTCTGCTAAAAACCATGGATTCTCTGCGGCAACTGCATTCCATC

ACCATGGATGTCCATTCAAGATTCAAAACAGAAGCCCACGATACAGTAGTGAACAGATTCAACGAACGGTTCCTATTGTC

GTTAGCTGACAACCCCCGTTGTTTGATCCTTGATGATGCACTGACAGTGCTTCCGATATCTTCGAAAACTGCACAAGTGG

AACCAGTCAATGAGACACCAGAGCGTATCAACCCGAAGCTGACAGAGCTAGTGTCGTCCCTCTCCGACTCGCCGCCCGCC

GGCCCGCTCGTCGCTCTCTGTCGCACCTACGACCAGGCGTCAGCGCTTATCGCTATCATAGACACACTAGCTTCCAAAGC

ATCCAGGCCTCCCCACTGCCTCACAGCGGCCCGAGGCCGCGGCAAATCAGCCACTCTCGGCCTCGCGGTCTCAGCAGCAG

TGGCTTTAGGCTACGTTAACATATACGTAACCTCTCCCCACCCAGAAAACTTAATCACATTCTTCGAATTCGTCCTGAAA

GGCCTTGACGTCTGCTTGTACCAGGAGCATATCGATTACAGTATACTGAGGTCGACGAACCCGGATTTCAAGAAGGCTAT

TGTTGGGATTAATGTTGCTAGGAATTCGAGGCAGACTGTACAGTACATAACTCCCGACGATCACTCGCTCCTAAGCGCCG

CAGACCTCGTACTAGTAGACGAAGCAGCTGCCATACCAATAGCTCACGTTTCCGCGGCCGCTACCAAGGCACCGCTGGCT

TTACTGTCGTCAACAGTATCTGGGTACGAGGGCACGGGACGAGCACTTTCGCTGAAACTGTTTGCTATGCTGCAGACGAA

ACACGATGCACCAGCTCCGATAAAACTAGAAGAGCCAATCCGCTACCGCACCAACGACCCAATAGAGGCGTGGCTTAACT

CCCTCCTCTGCCTCGAGTCCCCCGCCCCCTCGCTGGGCGTGGGCTCCCCCTCCCCCCACTCCTGCGAACTGTATCGGGTC

AACAGGGATGCCCTTTTCTGTTACCATAAGGCGGCTGAAGCGTTCTTGCATAGGCTGGTGGCTATTTATGTTGCGAGTCA

TTATAAGAACAGTCCCAATGACTTGCAACTGCTGGCGGACGCACCAGCGCACTGTCTGTTCGTGCTGCTGGCGCCGACAC

CGCCCAACGCGACTTCTATGCCAGAAGTGCTCTGTGTTGTACAAATGTGTCTGGAGGGAAACATCTCCGATAAGTCAGTT

CGCGACAACTTAGGGCGCGGTCGCAAGGCGGCCGGCGACCTGATCCCCTGGAACATCTGCGAGCAGTTCGGGGACAAAGG

CTTTCCCAAACTTTCCGGAGCCAGGATCGTGAGGATAGCCACGCATCCTTCTTACCAACGGATGGGCTACGGCAAACGTG

CCTTACAACAACTAGCAGCATACTACTCGGGAGAGATCCCGTGTCTGGACGACGCGCAGCCCGACAGCGACGACGGCAAC

GACCGCCGCAGCGACACGCTGCACACAGAGACTATTGTGCCTAGAGCGAAACCACCAACTCTTCTGAAGAGGCTATCAGA

GGTGCAAGCAGAACACCTGGACTATCTCGGGACCAGTTTCGGTCTCACCGAGGACCTGCTCAAATTCTGGAAGTCGCAGA

AATACGTGCCTGTGTATGTGAGTCAAAAAGCCAACGAGCTAACCGGCGAGCACTCCTGCATAATGCTGCGCTCGCTGTCT

GACGGCGCGTGGCTAGTCGCCTACAGCGCAGATTTCCGCCGCCGCCTGGCGCGTTTGCTCGCGCGCGCGCTACGCACGCT

ACCGGCAGCGTTAGCGCTGCCCACACTGGTCAATGATAGCGTCGCTGTTAAGAAGGCTGCCCTAACAAAGGAGCTAATAT

CCCAGCACCTGACGAACCACGACTTAGCCCGACTAGAGTCGTATTGCCGGCAGCAGGCCGACTACCGACTTATCACCGAC

CTGCTCGCGCCCATCGCCGGCCTCGTGTTCCAGGCTAAGGGTGCCAAGCTGGACGCTATTCAGCAGGTCATATTCGTAGC

AATGGGCTTCCAAATGAAGGAACCTGACGACATAGCGTCCGAACTCGGCCTCCCCGGTTCACAGATCCTAGCCAAATTCT

ACGAGGCCTGCAAAAAGATCAATGCTAGTTTCAACTCCGTTGTCGAACAATCGGCAGCCAAGGAGATCGGTATAGAGGAT

ATGTCGGCCGAGTTGGAGGGAAGCGGGCCCGTCAAACAGAGCCTGAATGAAGAACTGAACAAGGCTGCTAAGGAGTTGGA

GCGGAAGCAACGCAAGGAACTGTCCAAGCTGATGGGTGAGGACCTGGCTCAGTACCGCATCAAGGGTTCCGATCAGGATT

GGGGGCAGGCGCTCGCAGCTACTAAGGCTAAGGCGCTGGTCTCCGTTAAGAGCGGTGAGAAGCGGCTTGGCGATGACAGC

AAGGAATTAGACGACCTGATGGAGCGAGACAACAGCAAGAAGAAGAAGAAACAGAAACACAACCGCAAAGACGTGTAA

>Cher_FAT15

ATGGCTGGCTTCAGCTGGTACCTTTCTGAAAGCTTCCAAGTAATCATTGAGAAGTCGAAAGATGAGAAATGCTCCTTACA

AGATGTGCAATTACGTTTCTTGTGCCCTGATGACTTAGAAGAGGTACGGTGTCTATGCAGAGAATGGTTTCCCATAGAAT

ACCCGCAGTCTTGGTACGAAGACATCACATCATCAGAGAGATTCTTCGCTCTAGCCGCTGTCTACAAGTCGCAGATCATT

GGCCTTATTGTGGCAGAAATCAAGCCTTATCTAAAACTGAATGCCGAGGACAGAGGCATATTGTCAAGATGGTTCGCCTC

AAAGGACACCCTTGTCGCGTACATACTGTCTCTAGGCGTGGTTCGCTCGCACCGGCGCTCGGGCGTGGCGACGATGCTGC

TGGATGTACTGATGCGGCACCTGGCCGGCGCGGCGCCGCGGCCGCCGCACGACCACCGCGTGAAGGCCATCTTTCTGCAT

GTCCTGACCACCAACACCGAGGCGATACTGTTCTATGAGCAGAAACGATTCCGCCTCCACTCGTTTCTCCCCTACTACTA

CTCCATCAAGGGCCGCTGCAAAGACGGCTTCACCTACGTATACTACGTGAACGGGGGCCACGCGCCGTGGGGCATCTACG

ACTACGTGAAGTACGTGGCGCGGGCGGCGTGGCGCGGCGGCGGCCTCTACCCCTGGCTGTGGGGCAAGCTTCGCACGGCT

CTCACCATCGCGTGGCACAGGAATGCCTACAGAATTTAA

>Cher_FAT16

ATGAAGTTAAATTATTGTACCAAAATAGTTGGTGAACAAATCGTTTTAGTCCCGTACAGAGATTTACATGTCAGAAAATA

TCATGAGTGGATGAAATCTGAAGAACTCCAAATGCTAACAGCATCAGAACCTTTAACACTGGAACAAGAATTTGAGATGC

AGAAATCCTGGAGAGAAGATGAAGACAAATGTACATTCATTGTATTGGATAAAGCCAAGTTTGAAGATTGTCATGAAGAA

GTTGATGCAATGATTGGCGACACCAATATATTCATCACTGATAAAGAAACCTCTACAGGAGAAATAGAAATAATGATTGC

AGAGAAGAATGCCAGGGGCAGAAAAATAGGATGGGAAGCTGTCATCTTAATGTTACTGTATGGGATCAAGCAAATAGCTC

TAAAGAACTTAGAGGCCAAAATTTCGATGAAGAATGTAATAAGCATCACAATGTTTCAAAAATTACTCTTTAAGGAAACT

TCAAGAAGTGAAGCGTTTCAAGAAATAACACTTGCAAAGGCAGTTGATGAGGATTGGATAAATTGGTTAGAAGAACATTA

TAAATATCAAATACAGCCACATTAA

>Cher_FAT17

ATGTCGCAACCACCCTACACATTGGAGCCGGTCGAGAAGGGAGACGAAGAAAATATCATGAAACTGCTCAAAAAGACATT

CTTCATCGACGAGCCCTTAAACCAAGCAGTGGGTCTCATAACCTCAGAGAACGAGACCTGCACAGAACTGGAGGAGTACT

GCACGCATTCCCTGATGGAGGGGCTGTCCTTCAAAGCGGTAGACAGCCAGCACCATATAGTGGGGGTCATGATCAGCGGG

GTTATGCCCCTTAAAGAGGACACCATCAACGGCAACGATCTTCTAAGCCAAGCGCAGCGGTGCAAGAACCCAAAATTCCA

GAAGATTCTATACATCCTGGCGAGACGGGAGGCGGGAGCTCGTCTCTGGGAGAAGTACCCTGAGGAGCAGAACCTGGTCG

AGATAATGGTGGCAGCCACAGACCCTAGCTGGAGGAGGAGGGGGATTATGAATGAGCTGCTGAATAGGACTGAACAAGCC

ACAGCGCAGCGAGGTATCCGTCTCTTACGTATGGACACATCAAGTGCGTACTCCGCGATGGCAGCAAAGAGGTTCGGCTA

CACCAACGTGTACAAGGCTCTATACACGGAGATTAAGATGGACGGACGGCCTATTGTAGTGCCTGAACCACCGCATGTGG

ATGACAGGGTTAATATTAAGAAGTTATTTGATTAG

>Cher_FAT18

ATGAATCAACTCGCTGACGAAAATGTGCAGAACGTCAAGAGTAGTCTCAAAACGACGAGCACAAAAAACAAAAATAAAAC

CGCCAAAGATAACTCACCTTCTAAAACGACTGACGAGGTAAGAATAGTAAATGAAGAGAAATTAAGTGCGGCTCTAGCTA

GTACTTTACACGTTAACAACATCAATAGCAACCTAACAAATGGCACGAGCGACCACTCGAGAGAGGAAATAGCATCGTCC

GGCAATGACAAAGCGGGCGACGAAGGCCCTAGAGATGCAAGCAGTGTACAGCCAAGTGATGCCAGCAACGGGGACAGCAG

AGGGACATGTGCTTCTCAAATGAATATGGTCAGATACATCAGCGAAGAGACTCTAGAAAACCTTGAGAACACTTTCGACC

TGCAAGGTCAGTCCTCGCAAGAAGAAATTGAAATCATATCATATGAATCTGAACTGCAAATGCCTGAAATCATGAGAGTC

ATACAGAAGGATCTATCAGAACCATACTCAATCTACACTTATAGATATTTTATACACAACTGGCCTAAGCTTTGCTTCTT

AGCGACTCATGGTGAGAAGTGTATTGGTGCCATTGTATGTAAGCTAGACATGCATCGAAATGTGGTGAAAAGAGGATATA

TTGCTATGCTAGCTGTGGATGAGAAATACAGGAATAAGAAAATTGGTTCAAGGCTGGTACGGAAAGCAATACAGGCAATG

ATTAACGATAATGCAGATGAAGTAGTCTTAGAAACTGAAATAACGAACAAACCAGCACTCATACTTTACGAGAACTTGGG

GTTTGTGCGTGACAAGCGTTTATTTCGATATTACCTCAATGGTGTGGATGCGCTGCGATTGAAGCTATGGTTAAGGTGA

>Cher_FAT19

ATGGCGAAGATGACAGAAGCCGAGATCCTGGCCAAAATGAAGGTGCTGGAAGACCGGATCAAGGCCCCCTCGATCTGGGG

CCGCGTGCCCTGCGGAGTCCGGTTTGAGGATCTGCAGGAGAAGCGGTATGAAGACACTGTACGGCTGCTGAAGAAGCACT

ATCTACCTGACGAGCTAACATACAGATCAGTGAAGATAGCCGACGACAAAGAAGGTACCGACGAGTTCACCCACAACCTG

AGGATATGGATGAAAGACAAGATGTCCATCGCTGCCGTGAAGGAGGGAACAGACAAGCTGGTGGGCTGCCTCATCATGAG

AATACAGGAGAAACACGCCTTCTCCCGAACCTTCAGCCGAGTTAAGATAACCTACAATCCACAGTACAGCACAGTGATGA

CGTTCTACAACGCCGTCGAGAAACCAGTGGACGTGTACGATAGACTCGGCGTGAGAAGATACTTCAAAGTTTACCTCGTG

GCCCTGAAGCCCAGATATCGGCATCGAGGGTTAGCCAAGGAGATGCTAAAAGCCGCCTTCTTATTGGCTGCAAGTGCCAA

TATCCCAGCAGTCGCTGGCATATTTACCACCGGCAATATGCATCAGAAGGCTGAAGAGCTGGGCTTCCAGAAGTTCAACG

AGATATACTACGTTAGATATCTTATTAACAATGAGATTGTCTTCTGGGACACGGGCCTCGGTAACTATGGCGCTGCCCTA

ATGGGGTACAGGGTACCAGATGTAGAGGAACCTGCGGAATTGAAAAAGCAGCAGTCCTCGAGGTTCAACGTACAATTGGA

AGATATCGAGGGTGATGAAGACGAATAG

>Cher_FAT20

ATGGGTGATAACGACGGTTATTACGATGGTGTCGGGACCATGGAGTCTATGCCAGGTTCACCTGAAGTCACCTACAACTG

GGTTGATGTAACTACAGATTTCTTCAAAAATATAGAAGACCTCCGACTTGGGGAGTTATTGCACGATGGCCACCTATTTG

GTTTGTTCGAAGCGATGTCGGCCATCGAAATGATGGACCCTAAGATGGATGCAGGGATGCTTTGTAACCGAGGCGTTGCT

AAGCCTCTTAACTTTACGCAGGCTGTTCAGGCCGGAAAACTGAAAATTGACGATCTTGAACCAAATGAACTGATTGGTAT

AATCGACGCTACCCTGGCTTGCATAGTGTCTTGGCTAGAAGGCCATTCCCTAGCCCAGACTGTCTTCACTAACCTGTACT

TGCATCAACCACATTCAATAGTGAACAAGACTTTAAAGGCATATTGTATAGCTGTTTATAAACTACTGGATTGTATACGG

GACTGTATTAACAAGGCCCAAGTGTTTGAAGAAGAAGATTTCCAACCAATGGGTTATGGTTATCGTCTGGGCTCGAACCC

GCAGACTGGCAGCACTTTCGACCCCAACCTGGATGTTTCAGAGCAGAAATGCATTGCTCTGCTGCGAGAACAGGAGGAGG

AGCTGAATAAGAGATCTCGGGGTACAGATGATGAGGACAACCTTTGGACTGCACTTGCAGCTAGAATAAGGTTCACAAGG

ATGTTCTACCAAGCACTTCTCCTGATCACTAAAAAGGATTCACAGTCCGGTGCTGACTGCGTGGCTCTGCTCAACGGCTG

CTCTGAAATGATGAAAGTCATCATCAGGACATCTCCGAAGGGGACACAAGCTGTGGAGAATTCGGACTCTCCAAACCCCA

TGGGGTTCGAGCCGATGATCAACCAGCGGCTGCTACCGCCCACGTTCCCCCGCTACACGCGCATCAAGCCGCGGGCCGAG

GCGCTCGCCTACTGCGACGAGCTCGTGGCGCGGCTGAGGCGCGCCTGGAAGATCACGTCTTGCACCAACTTCCATACGGC

TTTGGACTTCTTCATGGAGTTCAGCCGCGCGCGCGCCTGCATCCTCTCCCGCTCGGCGCTCCAGCTGCTGTACCTCAGCC

CCTCCCCCGCGACCACCGCCTCCATGGCCCAGAGCGCCATGAGCGCACCGCCCACCGCCCCCCGCCCGCCCCACGCCTTC

AGGGAGATACTGCGCGAGTCGGTGCGGAGCTTTGTTAACCCGCCCGCCCTGACACCAAAGTCGCCTGTGCTGGCGACTCC

ACAGGCTCGCGAGTTCGTGGAGAATTTCCTCGCTCGCTGCGTGCGTCCGTTCGCGGTGCTGCTGCAGGTGTGCGGGCACA

ACCGCGCGCGGCAGCGGGACAAGCTGGCCCTGCTGCTGGACGAGTTCGCTGCCCTACAAGAAGAGGCAGAAAGTGTGGAC

GCGGTGGTGAGCGGAGCCGCGGGGACCGCCCCGCGCGCCTGCTTCGGCACTTGGCTCCTGTACCACGTGCTACGCGTCAT

GATCGCGTATCTACTCTCCGGGCTCGAGCTAGAACTGTACAGCGTGCACGAGTACCATTATATCTTCTGGTACCTATACG

AGTTCCTCTACGGCTGGCTAGTCTCAGCGCTAGGGCGGGCGGAAGGCCTAGCGGGCGAGGGCGCGCGCAGGCCCGACACG

AAAAAGAGCGGAGCGCGCAAACAGAAGAAACGGACGCGGCCTTATGCGCGGGAGGCGCTCATGTGCCAAGTCATGCAGAA

TATGTGCGGGGGGTATTATAAGGCCCTAGTAGCGTTCAAACTCCAAGGCAAGATCCGTCAGCCAGCATCGGAGTTCGACA

ACGCTTGCGTCCGCTACAAGCACCGCTTCGCGCCGCTGTCCGTGCTCACGCCGCCGCAGGTGCACTACCACGAGTTCGTG

GAGATGACGCAGCCGCTGCAATATGAAAACCCTGTGATACTATACCTAGGCGGCTGCAAGCACTTCCAGCAAGCTCGATC

ACTTCTAGAAACTGTCACAACACCAGACCAAGAGGTAACGGATCTTCTAAAGGTAGCAAAAACAAATTTCGTAGTACTCA

AATTACTAGCCGGTGGACACAAGCGCGACTCGACGACGCCGCCAGAATTCGATTTCACCGTACATAGACATTTTCCTATC

ATCAAACTTGTATAA

>Cher_FAT22

GCGCTGGGCGCGGCGGCGTACCAGCGCGGCTATTGGTCGTTCAGCGGACGCACGCTCGTGGTCTTCGGCCTGTATGTGAG

CCCCCCGGCGCGTCGCCAAGGGGTCGGCAGACGCCTGGTGCTTGAGATGTGTCGGTTGGGGCTAGCAGAGAACGCGCAGG

TGGACTGGCTGCAACCCCGAGGCCCTGACTCTGAGGCATTCATAGCACAGTTCGGGGCTGTCAGAAACGAGGTTTTTGAT

GACCATCACATGATGAGGATGCGATTTAACTCCATTAACTTAGCTGCCAGCGGAGGCGATGGAAGCAGCGGTGTGCACAA

TCAAGGGCCGGATGCAAATGAAACCGTCCTAATTCGCCGGGCGAGCCGGGATGACTTGGCAGCCATGATCGATATGATAA

ATGAGCTGGCTACCTTTCAAGGGAAACCGGAGGGGCCTCAACTAACTGTTAAAGATTTAGTGGAGGACGGTTTGACGTGT

TCCGTGCCGTGGTTCTTCGCGTATGTGGCGGAGCTCGGCGGTGCAGTGGTGGGCATGGTCGTTGGGACTCGCCCCTGGAA

CGGCTACAGGGTCCATTATCTCCAGAATCTGTACGTGCGGCCCTGCGCACGGCGGCGCGGCGTCGCCGACCAGCTGATTA

GGGAATTTTGCAAGTTTTCCCTGGAGGAAGGGGTGGAGCACGTGGACTTGCACGTGTCACACAACAACCCGCCGGCTCAG

CAGCTGTACCGACGCCTGCAGGCCTCCGAGGTGCCCGCGCGAGACATCCCGCCTGTCTACCGGCTCACCAGGAACCAGAT

CAAAGACATTGTGGCGCAATCTTTACTATAA

>Cher_FATP1

ATGGCAGAAAAGACTGAGGTTCGCTTCGAGGAGGCGGAGCGCAACAAGGCAGCCATCTTAGCTTCCATCGGAGCAGTCAG

CACAGTCCTGGGATGGCTCTCCCTCGGCTCAGGGGTCCTCGTGGCCGCTGTCGCCCTAGTAGCCATTTACCTAATGACCG

GGGACCGGTACCAATGGATCTACATATGGCACAAGACTGTTTACAGGGATTTATTAGGTCTCCGGGTGCTCCTCATCACG

ATGTTCAAGATCTGGATGTGGGAGCGGCGCGGCACGACGGTGGTGAAGCGCTGGGAGGAGGTCGCACGCTGGGCTCCTGA

CAAGAAGGCTTTCATCATGGGCGAGCGCTCGCTCACCTTCCGGCAGGGGGACGAATTCAGCAACCGCATAGCCCGGTACT

TCAAACGGCAAGGGTTCAAGAGTGGCGAGGTCATCGCGCTCTTCATGGAGACGCAGCCAGAGTACGTCTTCATCTGGCTC

GGGTTGGCCAAGCTCAGGGTCACCACTGCCCTCGTCAACACCAACCTGAGAGGAGCCCCACTCACGCACTGTCTCAAGAT

TGCTGGGTGCAAGGCGCTGATCTTTGGGGATGAGATGACGGATGCTGTCAAAGAAATCCAGAACGAGATTCGTGACATCC

CACTCTTCCAATTTAACTCGATTGAGCGTAGTGAAGCCGCCACTCTCCAGGACACGTCTCCTCTGGCTTCTGAACTGACT

GAGATGAGCTCTGAACCCTTCACTGAAGCCGAGCCGGCGAACCCGCGGGACACGCTCATGTATATCTACACCAGTGGTAC

CACTGGGTTCCCTAAGGCTGCTGTTATCACGAACATCAGGTATTTCTTAATCCCACTCGGCGTGCACACATCAGCGTGTC

TCTCTTCTGAGGACATAATTTACGACCCCCTCCCCCTTCACCACACGGCGGGCGGTGTGTTGGGAGCAGGACAAGCCCTC

ATATTGGGCAGTACGGTTGTGTTGAGGAAGAAGTTCTCTGCTTCTAACTACTGGAGCGACGTGGCGAAGCACGGTTGCAC

TGTAGCCCAATACATCGGCGAGATCTGCCGCTACCTCCTGGCTGTCCCGCCAGCGGCCACCGACCAGGCGCACCGAGTCC

GCGTGATCGTGGGCAATGGACTAAGGCCGCAGATCTGGCAGGAGTTCGTCCAGAGATTCGGAATTGAACGGGTGCTAGAG

TTCTACGGGGCTACTGAGGGGAACAGCAATTTAATCAATTTGGATTCAAAAGTGGGTGCGATAGGATTCTTGAGCCGTTT

AGTATCTTCGGTGTACCCGCTTACTCTAGTCAAATGCGATGAAATATCAGGAGAAATATTGAGGAATAGTGAAGGAAGAT

GTGTCAACTGCGGGCCACACGAACCAGGACTCCTACTAGGCAAAATAGACGCCAAGAAGGCCATCCTAACGTTTTCCGGG

TACGCTGACAAGACAGCTTCTGCCAAGAAGATGGTCAGAGACGTGAAAAAGGAGGGCGACTGCTACTTCAACACGGGGGA

CATCCTCGTCATGGACCACTTCGGGTACTTCTACTTTAAGGACCGGACTGGGGACACTTTCAGGTGGCGTGGTGAGAACG

TGTCAACCGCAGAGGTGGAAGGAGTCATCAGCACCCTCGTGGGATTAAAAGACGCCATTGTATATGGAGTTGCGATTCCC

AACGTAGAAGGTAAAGCAGGCATGGCGGCCATAGCCGATCCAGAAAGGAAGCTGGACTTCTCCCTCCTCACGAAAAGCCT

CCGCTCGTCGCTGCCAGTCTACGCCAGACCCCTCTTCATCAGGATACTGCCCGAGCCCCCCCTCACAGCCACCTTCAAAC

TCTGCAAGAAGGACTTAGTGGAACAAGCGTTCGACTTGGGAACCCATGAGGATCCTATGTACTTCTTGGACCAGAAGACT

GGTGAATATGTTCCTTTGACGAAGAAGTTGTATGATGATATTGTTAAGGGTGTTGTGAGATTGTGA

>Cher_FATP2

ATGTTGTTCGTACTGGGAGGTGTAGCCGCGGCTTGCGTCGCCGTTTGGCTGTATTACGGCTTTCTCTCCACTTTAGCACT

TTCTGCAGCCTTAGTGTTATTGTACGGAGTTTTGTTCCATTGGAGATGGATCTACGTTGCCCTGAGGACGGCCCCTCGGG

ATCTTCTAGCACTAACATGCTATGTAAAGCTGCTGTGGACCACATGGAGGTTTTCAAAGAAGAACTGGACTCTCCCGGAC

ATGTTCCATGATACCGTGAGTCGGTACCCGGACAAACCTTGTTTCCTGCACCAGGACGAAGTGTGGACGTTTAAAGAGGT

CGAAGACTTCAGTTTACGGGTGACAGCCGTCCTCAAGGCCCAAGGCGTCAAGAAAGGCAGCATAGTGGGCATGCTAGTAA

ACAATTGCCCCCAAATGCCCGCCATTTGGATGGGCGCGGGGCGGATCGGGGCGGTCTGCCCCCTCATCAACACTAATCAA

CGCGGGAACGCGCTGGTACACTCAGTCAATGTCGCTCAGTGTGACGTCGTCATATTCTCCGAGGAATATCAATCAGTGAT

TCAAGAAGTGGCAAGTCAACTAAGCCCGTCAATTAAATTTATGAAGTTCATTCACCGTCGCCTGAACACGGCGAATGCAG

AGGTCAAAGCCAGCGGTGACCACTTCGCGGATTTGACCTCTTTGCTGGAGAACACCCCGCCGGCGCCTTGGACCTTAGCT

GATGGGGAAGGCTTCCATGGAAAACTACTTTATATCTACACATCGGGGACCACTGGGTTGCCTAAGGCCGCTGTTATCTC

TAGTTCAAGAATCGTGTTCATGGCAGCAGGCCTTCACTACCTGGCAGGTCTAAGTCCAGAGGACATCGTGTACTGCCCAC

TGCCACTCTACCACACTGCGGGAGGCCTGCTCAGTGTCGGTCAAGCCCTTATTTTCGGTTGTACAGTCGCTGTCAAAACC

AAGTTCTCCGCATCTCAATATTTCCCTGACTGTATCAAGTATAAAGCCACAGTAGCCCACTACATCGGGGAAATGTGTCG

CTACGTTCTCGCCACGCCGCCGGCTCCCGCCGACACACAACACTCGGTGCGGACAATATTCGGCAACGGCATGAGACCAC

AGATTTGGGATGAGTTTGTGACCAGATTCAACATTAAGCGTGTCGCCGAATTTTATGGAGCCACTGAAGGAAACGCAAAT

ATTGTGAACATCGACAGCAAAGCCGGAGCGATAGGTTTCGTGTCCCGGATATTCCCGCGAGTGTACCCGATCGCCATCAT

TAAAGTGAACCAAGAGACGGGCGAGCCCATCCGAGATGCAAACGGACTTTGTCAGTTGGCTCAAGCAGAAGAGCCGGGCG

TGTTCATCGGCAAGATTTCCGCGGGCAACCCGGCGCGCGCGTTCCTCGGCTACGTCGACAAATCCGCGTCCGACAAGAAG

ATCGTACGAGACGTGTTTGCTAAAGGAGACTCTGCTTTTATTTCTGGCGACATCCTAATATCGGACGAGCTAGGCTACCT

ATACTTCCGCGACCGCACGGGCGACACGTTCCGCTGGCGCGGAGAGAACGTCAGCACCACTGAGGTCGAGGCCGCCGTCT

CCAGAGTCGCCGACAACAGAGACGCAGTCGTTTATGGAGTCGAGGTACCGAACACGGAAGGCCGCGCGGGCATGTGCGGC

ATAGTGGATGCTGACGGCACGCTCGACCTGACGAAGCTGGCTCGCGACCTGGCCAAGGACCTACCAGCCTACGCCAGGCC

TACCTTCTTACGGGTCATGGAATCCGTCGACATGACAGGTACCTTTAAAATGAAGAAGACAGACTTACAAAAGGATGGTT

TCGACCCAAATGCAGTAAAGAAAGACAAACTCTACTATCTCGATTCCAAACTGGGCCAGTATCTGCCACTCGGCCCCGAG

GAGTACGAAAAAATTATCAACGGCCGAATAAGACTATGA

>Cher_FATP3

ATGCCAGACATGTTCCACGACTTCGTGAAAAAGCACCCCAACAAAGCCTGCTTCTTATTTGAGGAAGAAACATGGACATT

CCAACAGGTCGAGGAGTACAGCTTGCGCGTGTCAGCTGTTCTCAGAGCCCAGGGTGTCAAGCCTCAGAGTAAGGTGGCTG

TGATGATGATCAACTGCCCTGAAATGCCGGCGACGTGGATCGGCGCCTGCCGCCTGGGTGCGATTGCACCGCTCATCAAC

ACCAACCAGACCGGTGCTGCGCTGATACACTCCATCAACATTGCTGACTGCGATGTTGTTATCTACGGGAGCCAGTTTGA

ATCTGCAATCCAAGACATTGCCAAAGACCTCAATCCATCCATCAAACTCTTCAAGCACATTCGTCGTCCTCTGAACACGT

CTGGCGATGGTGTGAAGGTGGCAGAGTCCTCCAACGATTTCACGGCTATGCTGGAGTCCACCCCGCCCTCACCCTGGTTC

CTGACTGAAGGGAACGGCTTTAACGGCAAGATGCTGTACATCTACACCTCTGGAACTACTGGGTTGCCCAAAGCTGCTGT

TATTTCCTCGTCCAGAATGGTGTTCATGGCTTCAGGCGTCCACTATCTCGGCGGCTTGAGAAAAAAAGACATAATTTACT

GCCCTATGCCGCTCTACCATTCGGCTGGCGGCTGCATCACGATGGGACAAGCCTTCATCTTCGGCTGCACGATCGTATTG

AAGACCAAGTTCTCGGCATCTGCGTACTTCAAGGATTGTGCCAAATATAATGCTACTGCTGCTCATTATATTGGTGAGAT

GTGCCGTTATATTCTTGCGACTCCTCCTTCAGCGGCTGACAAGCAACACAAAGTGCGCACGGTATATGGAAACGGAATGA

GACCAACTATATGGACCGAGTTTGTAACCCGGTTTAACATAAAAAAGGTTGTGGAATTTTACGGCGCCACGGAAGGAAAT

GCTAACATAGTGAACATAAACAACAAAACTGGAGCAATCGGATTTGTATCGAGGATCATACCGCAAGTGTATCCTATTGC

TATTCTGAAATGTGACGAAGAAACGGGTGAACCTATTCGAACTTCACGAGGATTATGCCAGGTGGCGAAACCTAATGAGC

CAGGAGTGTTTATCGGTAAAATCAAACCGAACAACCCTTCAAGAGCCTTCCTCGGCTATGCAGACAAAGCAGCGTCTGAA

AAGAAAGTTGTGAAAGACGTATTCACTCGTGGAGATTCTGCTTTCATATCAGGTGACATCCTGATAGCAGACGAGCTGGG

CTACCTCTATTTCCGCGACCGCACGGGTGACACGTTCCGGTGGCGCGGAGAGAATGTCAGCACCACAGAGGTCGAGGCCT

CCGTCTCCAGAGTCGCTGACAACCGGGACGCAGTCGTTTATGGAGTTGAGGTCCCCAACATCGAAGGTCGCGCGGGCATG

TGCGGCATCGTCGACACAGACAGCTCTTTGGACCTGGAACAGCTGGCACAGCGCCTGGCGAGAGACCTGCCGGCGTATGC

GCGGCCCGTCTTTATGAGGGTCATGACTAGCTTGGATATGACGGGTACATTCAAAATGAAGAAGGTTGACTTACAAAAGG

AAGGATTCAATCCAACAATTATTAAAGATAAATTATTCTACTTAGATTTAAAACAGGGGAAGTACCTTTCTCTTGGTCCA

GAAGAGTACCAGAAGATTGCGTCTGGGCAAATAAGACTGTGA

>Cher_FATP4

ATGGACGCAGTGCTGGCAGCTCTCGCCGCGCTCATGGCACTGGCCGCCGCGATGGCCGCCGTGCTAAGCACGCTCTCCAA

AGCCGCGATTTTCGCTCTCCTCGCGATCGCGCCCTGCGTATACCGCTACAGGAAGCGCATTTACGTAATCGGCAAGACGT

TACCCCGGGATCTCAAATTCCTATGGCGCTACGGCAACAGCATGGTCCGTCAGATGGCGTGGGGCCGGCAGAACGCAACG

GTGGCCGCGCTGTTCACGGCCCGCGCCGAACGCACGCCCGACGCTGCCTGCTTCATCGTGGTCGGCGACCGCACCTGGAC

CTACAAAGAGATAGCGTCCAAATCCAACCAAGTGTCGCGTGTGATGCAAGAGCACGTCGGCCTGAAGCGCGGCGACGTGG

TCTGCGTCTTCATGCCCAACTGCGCAGAGTATGTCTGTACCTGGATGGGCATGGCCAAAGTCGGCGCTGTCTCGGCACTG

ATCAACAGCAACCTTCGCCACAAGCCGCTGCTGCACTGCATCCAGGTCGCGAAGGCCAAGGCCGTCGTGTTTTCGGATCA

GCTGGCTGAAGCCATCGCCGAGATCCGCGACCAGCTCCCTACAGACATGAAGCTGTTCCAGCTCTACGGTCAGTGCGCGC

CAGGAGTGCTCGACTTTAGTGCGGAGATGGCGCGCCACCCGCCCGACTACCCGCTGGTGACGGAACAGCTGAACTACAAG

GATACGCTCCTGTACATCTACACTTCGGGAACCACTGGGATGCCTAAGGCTGCTGTGCTGCCTAATTCCAAGTACCTCCT

CATCGTGCTAGCAACGGTCCACATGCTGGGTCTCCGCGCCTCGGACCGCATGTATAACTCTCTGCCGATGTACCACACGG

CTGGCGGCGTGGTCGGCACAGGCGCGGCGCTTGTCGATGGCATCCCTTCTGTCATCCGGCCGCGGTTCTCCGCCTCTAAC

TTTTGGACGGACTGCATTAAGTATCAGTGCACGGTGGCGCAGTACATCGGCGAGATGTGCCGCTACTTGCTGGCGCAGCC

GGCGCGCGCGACGGACGCGCAGCACCGCGTGCGCATCATGGTCGGCAACGGCATGCGCCCCGCCATCTGGCAGAGCATCG

TCGACAGGTTCAAAGTACCGCAGATAAATGAAATATACGGCGCGACCGAGGGCAACGCCAATATAATAAACGTGGACAAC

ACGGTGGGCGCGGTGGGTTTCCTGCCGAAGCTGCTGCCGGCGCGCGCGCTGCCCATCGCCGTCGTGCGCGCCGACGAGAG

CGGCGCGCTGCTGCGCGGCGACGACGGCTACTGCGTCCGCTGCGCACCCGGCGAGCCGGGCATGTTCATCGGCCTGATTT

CTCAGGGGAACGCTTCGAGGGAGTACTACGGTTATGTGGACAAGAGCGACAGCAACAAGAAGCTCGAGCGCGACATATTC

TGCAAGGGCGACGCGGCCTTCGTGAGCGGCGACATCCTCGTCGGCGACGAGCTCGGTTACCTCTACTTCCGCGACCGCAC

CGGCGACACCTACCGCTGGAAGGGAGAGAACGTGTCCACTGGCGAGGTTGAGAACGCGATCAGTCCCGCCGTGGGACAGA

AGGAGGCTGTCGTGTATGGAGTGTCGATCCCAATGAACGAGGGCCGCGCAGGCATGGCAGCGGTAGCCGACCCGGCGCGC

GCGCTCGACCTGGCGCAGGTGGCGCGCGACCTGGACGACTCGCTGCCCTCGTACGCGCGCCCGCTGTTCCTGCGCATCGT

CAACGACATTGACATGACCAGTACATTCAAGCTCAAGAAACTGCAGTACCAGAAGGAGGGTTTCGACCCCGACGTGATCA

CCGACCCGCTGTATTTCCGCGCCGGCGCGCAGTTCGTGCCCATCACCTCGGCGCTGTTTAAAGACATCTGCAACGGACAA

GTCAAGCTC

>Cher_GOBP1a

ATGTTCTGGTTGGGCTGGCTGGGTCGGACGCTGCTGCTGGTGGTGATGCTGGGCGCGGCGCAGGGCACCGTCGAAGTCAT

GAAGGACGTCACGCTGGGTTTCGGAGAGGCCCTAAATAAGTGCAGGGAGAGCAGCCAGTTGTCGGAGGAGAAGATGGAGG

AGTTCTTCCACTTCTGGCGCGACGACTTCAAGTTCGAGCACCGCGAGCTGGGCTGCGCCATCCAGTGCATGTCGCGCCAC

TTCAACCTGCTCACCGACAGCGAGCGCATGCACCACGAGAACACCGACAAGTTCATCAAGTCCTTCCCCAACGGTGAGGT

GCTCTCGCAGCAGATGGTGTCGCTGATCCACGGCTGCGAGCAGCAGCACGACGCGGAGCCGGACCACTGCTGGCGCATCC

TGCGCGTGGCGGAGTGCTTCAAGACCGGCTGCACGGAGCGCGGCATCGCGCCCACCATGGAGATCCTCATGGCTGAGTTC

ATCATGGAGTCCGAGTCTTGA

>Cher_GOBP1b

GCGACGCTGCTGGGCGTGGCGCGGAGCACTGTCGAGATCATGAAGGACGTCACGCTGGGTTTTGGAGAGGCGCTCAAGCA

GTGCAGGGAGAGCAGCCAGTTGACGGAGGAGAAGATGGACGAGTTCTTCCACTTCTGGCGCGAGGACTTCAAGTTCGAGC

ACCGCGAGCTGGGCTGCGCCATCCAGTGCATGTCGCGCCACTTCAACCTGCTGACCGACAGCGAGCGCATGCACCACGAC

AACACCGACAAGTTCATCAAGTCCTTCCCCAACGGCGAGGTGCTGTCGCAGCAGATGGTGGCGCTGATCCACGGCTGCGA

GCAGCAGCACGACGCGGAGGAGGACCACTGCTGGCGCATCCTGCGCGTGGCCGAGTGCTTCAAGGTCGGCTGCCAGGCGC

GCGGCATCGCGCCCACCATGGAGATCCTCATGGCCGAGTTTATCATGGAGTCGGAGGCT

>Cher_GOBP2a

ATGGTGGCGGGAACTGCTGAGGTGATGAGCCATGTCACTGCTCACTTTGGGAAGGCTTTGGAGCAGTGTCGGGAGGAGTC

AGGCCTGTCTCCAGCGATCCTCGAGGAGTTCCAGCACTTCTGGCGCGACGACTTCGAGGTGGTGCACCGCGAGCTCGGCT

GCGCCATCCTCTGCATGAGCAACAAGTTCTCGCTCATGCAGGACGACGCGCGCATGCATCATGAGAACATGCATGACTAT

GTCAAGAGCTTCCCTCAAGGTGAGGTCCTCTCAGCAAAGATGGTGGATCTGATCCACAACTGCGAGAAGCACTACGACGA

CATCAAAGACGACTGCGACCGCGTGGTCAAGGTGGCGGCGTGCTTCAAAGTGGACGCCAAGAAGGCTGGCATCGCCCCTG

AAGTAGCCATGATTGAGGCTGTTATGGAGAAATAT

>Cher_GOBP2b

GTTTGTGCTGCGGTGCTAGTCGCTGGGGGGCAGACGGTGGCAGGGTCTGCTGAGGTCATGAGCCATGTCACTGCCCATTT

TGGAAAAGCTTTAGATGAGTGTCGAGAGGAGTCAGGCCTGTCTGCTGAGGTCCTAGAGGAGTTCCAGCACTTCTGGCGCG

ACGACTTCGAGGTGGTGCATCGTGAGCTCGGCTGCGCCATTCTCTGCATGAGCAACAAGTTCTCCTTGATGCAGGACGAT

GCTCGCATGCATCATGAGAACATGCACGACTACGTCAAGAGCTTCCCTCAAGGTGAGATCCTCTCAGCAAAGATGGTGGA

GCTGATCCATAACTGCGAGAAACCTTATGATGACATCAAGGACGACTGCGCCCGCGTTGTCAAGGTGGCGGCTTGCTTCA

AAGTGGACGCCAAGAAAGCTGGCATCGCACCTGAAGTGGCCATGATTGAGGCTGTGATGGAGAAATAC

>Cher_IR1

CGCCAGCGGGACGCGGCCGTGGGCGTGGTGCTGGATGGGGCGTGTCGCGACACACAGGATGTTTTGATTGACGCCTCGGA

ATCCAGGTTGTTCGACGCTGCGCATGCCTGGCTGGTGCTAGACGATGATCAAGATGGCACCGGCGTTGGCGCACGATTCG

GCGATCTGAAACTTAGTGTTGATGCTGATGTTGTGGTTGCTACTTACTGCGAGGATGACGGTGTGTACCACTTCAGTGAT

GTTTTCAACTTTGGACGCATCCAGGGCAATCCTTTGGAGGTCAAGAAACTAGGAACCTGGACTATGGAGATCGGTTTGAA

CGTTTCGCCCCCAAGATTTAAGTACTACGATCGCTGGGATTTCCACAATCTAACTCTGAGAGCTGTATCCGTGGTAAGAG

ACATTTCTCAAAAGTTTGACGAGAAAATGCTCTCTGCGCCGGCGTACACGGACGGAGTAGCCGCAATGACGAAGATCAGC

ACTCAACTCCTCAACTTGCTCAAGGAAATGCACAACTTCCGTTTCAACTATACGATAGTGGGGCGCTGGACAGGCCTGCC

AGAGCGCAATACCACTCCGACAGTCTCCAACACGCTGTTCTGGCGCGAACAGGACATCTCCTCGACATCAGCGCGGCTGT

TCCCGGCCTGGCTGGAGTGGGTGGACACCTTCTTCCCCCCCGTCACGCACCTGGAAACGAAATTCTACTACATAATACCA

GACAAAGGCGTCGGAGACTATGAGAACCAGTTCCTCACCCCAATGTCTCCAGGAGTGTGGTGGTGCTCGGCTGCAGCCGG

CGTGGTGTGCGCGTTGGTGCTGATCGCTGCTGCCGCCACGGAGGGAAGACCGGAGCCGAGGTCCTATGGGATATTCAGCG

TTCTGGCTGCTGGGTTTCAGCAAGACTATGAAGATGGAGCGCAATCTCTAGAAGACAGTTCAACTAACAGTCGCAAGCTA

GCGCTGCTAGTAGTGGGTCTGACAAGCATGCTTCTCTACAACTACTACACCAGCAGCGTGGTATCCTGGCTTCTGAACGC

GGCAGCACCATCCCTGGACTCCCTGGATGCACTCATCAAGAGTGACTTCGAGCTCATCTTCGAGGATATAGGGTACACGC

AGGGCTGGATGGCTAATCCTGGATTTTACTACTACAGCGGCTTCACAAACGAGAAAGAAGACGAGTTGCGACTGAAGAAA

GTGACCAACGCCAAGCGAACAGTGCCGCTGATGCAGACTGTCGAGGCTGGGACCGAGTTGGTGCGGACTGGCAGTTATGC

GTACCACACAGAACCGTACACAGCCAGCCAGATCATCTCGCGTACGTACGTGGACAAGGAGCTGTGCACGCTGGGGGGCC

TGCAGATGATGATCCCGGCGCACGTGTACGTCATGGGGCAAAAGAGGAGCCCTTACAAGCAGTTCTTCGTCTGGAGTATG

ATGCGCCTGCTAGAGCGCGGCCATATGAAGGCGACGCGTGCGCGCGTGGGGGGCGTGGTGCCCCCCTGCTCGGGCACAAA

CCCCCGAGCATTGTCCCTCGGGCAGGCTGCACCGGCCTTTCTGCTGCTCGCTCAAGCCATGGTGCTCGCTCTGCTCATGT

TAACTGCTGAGCTGTGGTGGCACAGATACAAACAGCGCACAGCGATCGACGGACGACGGCCACTGGTACAGGTGGTAGAG

AGATCACGTTGA

>Cher_IR3

ATGCTTTCAAACACTTTGTTGCTTTTCTTGGTTAGTACAGTTTCGGGTTTGCAGCAAAATGTTAAGGAATTTCCTTTAGA

TTTCTTTAAAACAAGAGATGTGAAGTTTATCTGTTTGTTGACTTGCGGGGATAAAAGTTGGAATCAGCAGTTTGCTGTTA

ATGCCTCAAAATCGTCAATTGCTGTTTCAAGTGTTACTATTGACAATTCATCATCAGATTATGCTGATGCATTAAGAGTT

TGTTTGACGCATAAATACACAGCCATTGGCGTTTTCATAGATGAACAATGTCCACTTTTTGAAGACGTGCTGCTATTTGC

TTCTGAAAGCGGTTTCTTTGACGCAAATCACAAATGGTTGATAGTTGACAATGATGACTGGATGTGCAATGTCACAAGTG

GTGCTGAAGATTTTGAGCGAAACATTAATGTCACCAGAGACTCAGAATACTCTGAGAGTAGCGTGAATTTGACCAGGCTA

AATGATATATATGATAAACTAAATCTAAGTGTAGATGCTGATGTGACGCTTTCTTTGCAAGAAGGACCCGAAAATAAAAT

TTACGAAGTCTACAGCTTTGGTACTATTCGCGGTGGAAGTACTATTGTCAATAAACTTGGATCTTGGAAAAACAAATCAG

CTTTAGTGCCGCAACTAAATGGATACAAGTACTATCGACGGTGGGACTTCAATCAGTCATCTTTAAACGTAATTGCTGTT

ATGTCCACTCCTCCCGAAGTATTTGACGTAGACATGATAATTGGAGACCAGCCCAAGGTGGGTGTTGCCATAATAACTAA

AACGAGCCTGGAGGTTCTGGAGGAACTGAGAGAGCTGCACAATATCAGATATAACTATACAATTGTGGACCGGTGGATAG

GTGACTTTAATAAGAACAGTTCAAGGGTTGCTGCTAATTCTCTGTACTTCAAGGAACAGGACATCACACCAGTACTACGT

GTCACGCGTGAAATTTTCCAGAAAGTGGACATGCTGCTTCCACCACTGACAGCTATTGAAACAAGGTATTACTACCGCAT

CCCAACGACTGGCCCTGGAAAGTTCGAGAACCAATTCCTACGTCCATTGACCCCTGGTGCATGGGGCTGCGTGACAGCAG

TCACTCTTCTATGCAGCTTCATGCTATTCCTGGCAGCTAAGGTGGAACGACGTCCATCGGCGGTCCAGTATGCAGTGTTC

TCTGTCATGGCCGCATTTTGCCAGCAATTTTATGACGATAATATTGGCAACGAAGATCCAACACAGCAATCCTCAGCTCG

CCAGCTCGCCGTGTTGGTGACAGGAGCATCCTGCGTGCTGATCTTCAACTACTACACCAGTAGCGTGGTCAGCTGGCTCC

TGAACGGGCCACCACCTTCCATCAACTCTTTGGAAGAACTCTTGGAGAGTCCCCTGTCGCTTATCTTCCAGGATATAGGA

TATAGTCGTTCTTGGCTGCAGAACCCAACATATTACTTCAACAAAAAAAACGCCGAAGTTGAAGACAAATTACGCAGATA

TAAAGTATTCAATAAAAAAGTGGGGGCTCCCTTACTGGTGCCTCTGGAGGAAGGCATCGAGATGGTGAAAGCTGGAGGAT

ATGCCTACCACACTGAAGTGTACAACGCCAATACTCTGATCGCTAAGACGTTCACGCAGAGTGATCTGTGCGAGCTCGGC

TCTCTCCAGTCGATGGAGAAGTCGCAACTCTACGCTTCAGTTCCAAAAGATAGTCCTTACAAGGAGTTCTTCAATTGGAA

CCTCTTCCGCCTCCACGAGACTGGCGTCATCTCTCGCATCCAGCGCCGGACCAGCAGCCCTGAGATCGCCTGCGAGGGCA

GTTCCCCGCGAGCCCTCGCTCTGGGAGGAGCAGCGCCTGCTTTCATGTTGCTCGCCTTTGGATTCTTTCTGTCCACTGTT

ATACTTCTGATGGAGAGATTTATCAGCAGAAACCAATCGAGATTGTCAAAAGAATTAAATTTAAAAGCACCGGGTTGTTA

G

>Cher_IR4

ATGTTGTTCATACCGGCAGTGGTGGCGTTTTTCAAGTACAAAATTGTGTACAGCGTCATCGTTTTAGCATGTGGAAACAA

ATTTGAACAAATCAACGTACTGCGTCAGTTGTCTAAACATGGCATGAGAGCTACCGTAGCCTGCGACCCGACTGTCCTGG

ACGAAATGCAAGCTCGAGTACAAGGAGTCCTGTACGTGATGCAACCCAATGATACAATGCTGGACCAGGTTAAACAAGAG

CATTTCGCAATTTGGAACAAGTGGCTTCTAGTTGGTGACGAGATACCTGACAAGCTCCACCGAATACGTTATGATGCCGA

TGTAGTGCTTCTGGATTATCTTCAGACCGATACTGGAAATAATGGAATACTAACCGGNNNNNNNNNNNNNNNNNNNNNNN

NNNNNNNNNNNNNNNNNNNNNNNNNNNNNNNNNNNNNNNNNNNNNNNNNNNNNNNNNNNNNNNNNNNNNNNNNNNNNNNN

NNNNNNNNNNNNNNNNNNNNNNNNNNNNNNNNNNNNNNNNNNNNNNNNNNNNNNNNNNNNNNNNNNGTCGGACACTATGA

TGAAGCTGAGTACCGTGATAGCTTCGCACAGTACGTTCTTGACAACTCCATGCTAGAACGCGACTCCGCCATCAGATGTG

GCTACGGCGCCTCCGCACTCATTTTGGAGGCACTCAACGCTGAGGTCTTATTGCAAGTTCAGTTCTGGACCCCAGAAGTG

GGCAACAGTTCGAGTATGATGACGGAGGTTGCAAGCGGTACGTCACAGCTGAGCGGCGCGACTCTGCGCGTGAGAACGGA

CCGCATCGAGCGTCTTGACTATGTCTTGCCTATATGGCCTTTTGNNNNNNNNNNNNNNNNNNNNNNNNNNNNNNNNNNNN

NNNNNNNNNNNNNNNNNNNNNNNNNNNNNNNNNNNNNNNNNNNNNNNNNNNNNNNNNNNNNNNNNNNNNNNNNNNNNNNN

NNNNNNNNNNNNNNNNNNNNNNNNNNNNNNNNNNNNNNNNNNNNNNNNNNNNNNNNNNNNNNNNNNNNNNNNNNNNNNNN

NNNNNNNNNNNNNNNNNNNNNNNNNNNNNNNNNNNNNNNNNNNNNNNNNNNNNNNNNNNNNNNNNNNNNNNNNNNNNNNN

NNNNNNNNNNNNNNNNNNNNNNNNNNNNNNNNNNNNNNNNNNNNNNNNNNNNNNNNNNNNNNNNNNNNNNNNNNNNNNNN

NNNNNNNNNNNNNNNNNNNNNNNNNNNNNNATCCGATACGCCATGTTCGACGTAAAAACAACCTGGGAGGACCTCGAATA

CCTCAAGCGCAAGAAGATGACGTCAAACTTCTACCAGAGCACACAGCGCGGCATGCAGCTCGTTCTAGAAGGAAACACGG

CCTTCCACGCCGAATACAACCACATATACCCGTACATGAACATATTCAACGACGAGCAGATCTGCAAACTACAATATGTG

GACACAGTGCCCGAAATTATGTCATGGGTGGTGACAACGAAACACAGTCAATGGAAGGACGTACTCCGCTCCGCTGGCGG

CTGGGTTTACGAGACAGGGCTGGCAAAACGCCTCCTGACCCGCTGGCAGGTGAAGCCCCCGCCCTGTCGAGCCGCCCTGC

TCGCCGAGCGCGTCAAATACGGCGACGTGGCACCCCTGCTCACTTTGTCCACTGTTGGCTTTATAACGTCCGTGGTACTA

CTGTTCGTAGAGAGGACTGTGGCGAGATGGACGGCTATGAGGAGAGTAAAGTCAACTGGCGATGATGAGGCTGGAGTATC

GGGAGAGGAGAATAATTGA

>Cher_IR7

ATGATTTTAATAGTTATTTTTCTTGTGCTTGGAGTAAAAGCGCGAATCAACCCACATGGACCCACAGTAGTGAATGATTT

CGCGAATTGTGTCAACAAACTCATAGACATAAATTTTGCTCAACCGGGACTGCTCTACTTCATCAACACGAATGAAGTTA

GCACATCAGCCGCCGCGATAAGAACAGCAATACTAAAATCAGTGCACGATAAACTTAAATATTCTGTAAAAATTGCTAGA

CCAACTAAACAAGATAAAGCTATCTGCAAGAATGACCAAGAAGTATTAGCGACGTCAGGTTTGCACATAGACCACTTCGA

AGCAGTACCACTTGCTGACTACTTCGTTGTCATTATAGACAACTACGATGATTTCACCCATGTTGCGAGTAGACTGGCAA

GAGCAAGAAGTTGGAACCCAAAAGCGCTGTTCATTATTGTATTTTTTGGAATAACTAACACAGACGATCTGAACATCGAC

CACGCGGAAAGCATGCTACTCTGTCTTTTTAAACTGAATGCCATTAACACTGTGGTCATTATTCCACAAGCAAGTAATAT

TAGACGAGCAACCGTTTATGGTTGGAAACCTTACGACCCCCCTCAATATTGCGGTTATTTCAATGAATCAGCTAGAAAAA

GGTTGTTTATAGAAAACGAGTGTGACCGCGGCATTGCCAAGTTTACGAAGAGTATTTTTGAAGACAAAATTCCATCGGAC

ATGAAAGGATGCACTTTGAAAATGCTTGCACTGGAACGACAACCGTTTATAAGTCGAGAACCCAGTGATGCCAATATTGA

GCAGTTGTTGATCAACGATGTTGCAAAACGATATAATATAGTATTGAGTTACGAATTTCTTAATGTATTTCGTGGTGAAA

AGCAGACAGCAGGACTCTGGGATGGAGCTCTTAATTACCTTGTATCCAAAAAAGGACAGATGCTTTTAGGAGGTATTTTT

CCTGACAATGAGGTCCATGAAGACTTTGAATGCAGCTCTACCTATTTAGCTGACTCTTACACCTGGGTTGTGCCACGAGC

TTTTCATCAACCATTATGGTTGGCGTTGTTTATCATATTTCAGAAGACAGTGTGGCTCTCAGTTGTAGCGGGGTTCATTG

TACTTGCATTAAGTTGGAAGATTTTCGCTAAATTGAGTAGAGACCCTACCTATAGGAATAATCTAGAACATTACTTTATC

AACACTTGGATCAGTAATTTGGGATTTGTCGCGTTTTGTCGGCCTGTAACTCATAGTTTGCGTTTGTTTTTTATTTTCCT

TAATTTATACTGTGTTTTGCTATTAACGGCATATCAAACTAAACTAATCGATGTCCTGACAAACCCGAGCTTTGAACATC

AGATATCGACTATTGAAGAATTAGCAGCGAGCGACTTAGGATGCGGAGGTTCAGAAGAATTACGTGATCTTTTTGAAAAC

TCGACTGATCCTATGGATATATATTTTTCTAGTAAGTGGGAGAACATTATTGATATAAAGGAAGCAATTATTCACGTGGC

ACTTTACAGGAATTTCTCTCTGCTATGTAGCCGTCTGGAGTTGGCACACATTTCAGCAGTAGTACCGGAGCTCAGTGACG

AGTTTGGGAACTCTAAAGTCTATGCATTTGAAGGTAACGTTTTCACAGTACCTTTGGAGATGGTATCTTTGAAAGGGTTA

CCATTTATGAAGAAGTTTTCGAAGACTTTAGAGTCATTCAGACAGTATGGTGTAAACGACAAGGTGCGTCGATACTTTGC

AGGCTACACGTTAATGAAGAAAGCAACGCTACTAAATGATCTAGAAATGGAAAACACTGGTAGAGAAGCCTTATCCACTG

AATCTTTACAGGGTGGATTTTTGGCGCTTATCTTTGGTTATATGTTCGGTAGCATAGTTCTTGTAATGGAAATAATTATG

AATACCAAATTTGTTAAGAGTATAAAA

>Cher_IR8

ATGGATTTCTATTGTATCTTTTTGTTTATTTTCGTTATAAACTTGGGCTGCGTGGTTTCAGAACTGAGTTTACGATTTGT

GTTTATCATAGAATCCCACGAGCAGGACTTGCCGCAGCTGGTCGGCCGAGCGCTGAAGATAGCCGAGGACTCGCAGCCGG

AGACCAGGCTTAGCGACTCCATCGTTCTGCTTGACCGGGAGAACGAGGAGGACAGCTATAGGCAGCTATGCTCATCGGTA

TCCAGCGGCGTGTCCACAATCATCGACCTATCCTGGTCCCCCTGGGACTCAGCAGACCAGCTGGCTGCTGACTCGGGAGT

GCCTCTAGTGAGGACGCTGCTAGGGTCACAGCAGCTGCTTAGGGCTTTGGACGAGTATCTGGAGTCGAGGAATGCGACCG

ACGCAGCGCTGCTGCTGGAGAGCGAAGGAGACGTGGACCGCACCCTCTACGAGTTGCTGGGCGAGTCCAACATCCGCGTG

TGGGTGCACGCCGGCCTGACGCGGGACTCTGCGCGTGCGCTCAAGACCATGCGTCCCGAGCCGAGCTTCACGGCTATCAT

AGGAGGGAGCGCGTTCGTCGCTGATACCTATAAGAGGGCCGTGAAAGAGAAGCTCGTCCGTCGCGACTACCGCTGGAACC

TGGTGCTCACAGACTACAGTACCCTGGACGTGTCTCCGGCCGTGCCAGCTATGACCCTTCATGTAGACCCTGCCGAGTGC

TGCAGGATTCTCGGGCAGAAGGACGGATGCGGCTGCGGGCAGGATTTCGAGAGGAAGCTGCCGATCCTCTCGGCTCTGCT

CCAACTCCTGGCGGAGACGTACTCCAAGCTGGAAGACGAAGGGACCCTGACGGCCAGCGTGGACTGCGACAACCTGGTGC

CAGAGCTAAACGATACCAGGGCGCGGCTGTACCGACAGCTGGCTGAGGATTCGGGAGCTAGCAATGAGAGCGTGTTCTAC

TGGGATGGAAACAGGTACGGTCTCTTCCTCCGCTCCCGCTTCATCCTCTCGACCTCTAAGCCTGACGCCGGGCTGCAGAC

AGTAGCGTCGTGGACAGCTGATGAGGAGTACAAGCTGTTACCAGGAATGACACTGGAGCCGCTGCGACAGTTCTTCAGGA

TTGGAACTGCTGCTTCAGTACCATGGACGATGGCGAAGCTGGATCCGACCACGGGGGAACCGATGTTCAATGAAGAAGGA

CAACCGTTGTACGAGGGTTACTGCATAGACCTGATACAGAAGCTTTCTGAGGCAATGGATTTTGACTACGAGATTGTGAC

CCCAAAAGCGGGTACTTTTGGACGTAAGCTCCCAAACGGGACTTGGGATGGCGTGATCGGGGACCTTATGAGAGCGGAAA

CTGATATGGCTGTCTCATCTTTGACTATGACGGCGGAACGAGAGGAGGTTATTGATTTCGTGGCGCCGTACTTTGAGCAG

AGCGGAATACTTATCGTGATCCGCAAGCCGACCCGAAAGACCTCCCTCTTCAAGTTCATGACAGTCCTTCGTACGGAGGT

GTGGCTGAGCATCGTGGCGGCGCTGGTGCTGACCGGCTTCATGATCTGGCTGCTGGACAAGTACTCGCCGTACTCCGCCG

CCAACAACCCCGGCGCGTACCCTTACCCCTGCCGGGAGTTCACCCTGAAAGAGAGTTTCTGGTTCGCGCTGACCTCCTTC

ACCCCGCAAGGGGGCGGGGAGGCCCCGAAGGCGCTCTCAGGCCGGACCCTGGTGGCAGCCTACTGGCTGTTCGTAGTGCT

CATGCTCGCGACCTTTACTGCCAATCTGGCTGCGTTTCTGACTGTGGAGAGGATGCAGACGCCGGTGTCTTCGCTGGAGC

AGCTGGCGCGCCAGTCCAGGATCAACTATACAGTCGTGGAAGGTTCGACCATCCACCAGTACTTCATCAATATGAAGTTT

GCTGAGGACACCTTGTACAGGGTATGGAAGGAGATAACCCTGAACGCCACTTCCGACCAATCACAGTATAGGGTTTGGGA

TTATCCAATCAGAGAACAGTACGGGCATATACTGCTGGCTATCAACGCTTCTATTCCTGTATCAGATGCTAAGACAGGGT

TTAGACAAGTGGACGAACACACCGACGCCGATTTCGCATTCATACACGACTCTGCCGAGATCAAGTACGAAGTAACCCTG

AACTGCAACCTGACGGAGGTGGGCGACGTGTTCGCTGAGCAGCCGTATGCCATCGCCGTGCAGCAGGGCTCCAGGTTGCA

GGAGGAGCTGTCGCGAGCACTGCTGGACCTGCAGAAGGAGAGGATGCTGGAACAGCTGGCTGCTAAATACTGGAACGAGA

CGGCAAGACAGCAGTGCCCTGACGCGGACGAGTCAGAAGGCATCACTTTGGAGAGTTTGGGAGGTGTATTCATAGCAACT

CTCTTCGGCTTGGGTCTCGCCATGATAACCCTGGCGTGGGAGGTGTTCTACTACAAACGGAAGGAAAAGAACAAAGTACA

ATTTCTGGAGGACACGGAGAAGAAACCTAAGAAGGCTTTTGAGAAGGATCTGGATAAGAAGATAAGTGATGGCGTTGCCA

GACTAAGAAAGGGAAAGAAGAAACCAGGCAGAGTTACAATAGGGGACACCTTTAAACCTGTCTCGGAAAAAGAAGGCGTA

TCGTACATCAGTGTGTACCCCAAAACTGAATTTAAGCCGTGA

>Cher_IR21

ATGCTACTAGCAATAGTTCTAATAAAAATTGTCATACTTCATCACGCAGCAAGTCAAGATGTTGAATTCTATCCTTCACA

GTCTTTTCTTAATAATGTCAATAGAAAAATACTTCCTAAAAGACATAAACTTAAATTTGCTGTAAAATCTAAACCATATC

TTGAAAGCCACTTTGAATATAAAAGGGAAGTCCAGTGGAGGAAGTTTTATAAGGACGATGCCGATGATGTGCCTAAAAAC

AATACCCAAAAGAGAGCTGTAGACCCGGTCTTTCATGGTCATCCAAAAACAAGAGAAGAGCTATGGAACGAGCGTTTTAT

AAACACGAGCTCGACTTTCGATCAAACGCCGTCATTAATCGGCTTGATACGTAATATAACCCTAACCTATCTCACCGATT

GCATCCCAGTTATACTTTACGACAGTCAGGTGAAGTCCAAAGAAAGTTACTTATTTCAGAATCTTCTGAAAGATTTCCCG

ATAGCCTTTGTCCATGGCTACATCAACGAAGATGACGAAGTTGAAGAACCAAAACTTCTTCATGCTTCTAAAGACTGTGT

TAATTTCATTGCGTTTTTTTCTGATGTCACTAAAAGCGCCAAGATTCTAGGGAAGCAGGCGGAAAGCAAAGTGGTCATAG

TGGCTGGGTCCTCGCAATGGGCCGTGCAAGAATTTCTTGCTGGGCCACAGTCTAGAATGTTCATCAATTTGCTTGTTATT

GGACAAAGCTTTACAGACGGCAAAGATGATTCCTTGGAAGCGCCTTACATCATGTATACTCACAAGCTGTATACAGATGG

ATTAGGAGCAAGTCGGCCGGTAGTTCTGAATTCCTGGTCTCACGGAAAGTTTTCGAGACCAGTGAACTTATTTCCACCTA

AAATGGCTCAAGGATATGCAGGCCACAGATTTGTAGTAGCTGCCGCGCATCAACCACCTTTTATATTTCGAACAATAAAA

ACTGATCTAGACGGAGGCAACCCAAGAGTTGTGTGGGAAGGAATCGAAATGAGGCTGCTGGCATTATTAGGTGAAAGGAA

CAATTTCTCCATTGAAGTTAAGGAACCTCAAGATTTGCATTTGGGGTCCGGAGATGCAGTAGCAAGGGAAATTAAGTCTG

GTCGAGCTGACATAGGAGTTGCTGGTATTTATTTAACCAGCGAGAGAACCAGGGACATGGACATGAGCCTCCCTCATTCA

CAGGACTGCGCCGTCTTTGTCACACTCATGTCGACAGCCTTGCCTAGGTATCGAGCAATCCTTGGTCCATTTCATTGGCA

CGTCTGGCTGGCTCTCACTCTGACGTATCTTTTCGGCATATTTCCATTGGCTTTCTCAGACAAACATACTTTGAGGCACT

TGTTACACAACAGTGGAGAAGTCGAAAATATGTTCTGGTACGTTTTTGGGACATTCACCAACTGCTTCACATTTGTTGGA

AAAAACTCTTGGAGCAAAACTACTAAGATCACGACTAGGCTTCTAATCGGTTGGTACTGGGTGTTTACTATAATCATAAC

AAGTTGTTACACCGGTTCCATTATTGCGTTCGTGACATTACCAGTGTTTCCTGAAACGATTGATAGCATACAGCAGTTAC

TAGATGGATTCTATCGAGTTGGTACTTTAGATCGAGGCGGTTGGGAGAAATGGTTTCTGAACTCTTCGGATCCACAAACG

AATAAGCTTTTGAAGAAACTGCAGTTGGTATCAGACGTGGCATCAGGCATTAGGAACACGACGAAAGCATTCTTCTTGCT

ACCCTTTGCCTATCTAGGTTCACAGGCAGAGCTTGAATACATCATTCAATCGAATTTCACTCTAAGTAAAAAAAGCAAAA

GAGCAACGCTACACATCTCCAACGAATGCTTTGTACCGTTCGGGGTATCTTTAAGTTTCCCGAACAACTCCATCTATTCG

GCTAAGCTGAGTGGCGACATTGCTAGAATGGTACAGAGCGGGCTAGTGAAGAAAATCGTGGATGAAGTCAGATGGGAGAT

GCAACGAAGTGCCGCCGGAAAACTACTAGCTGTATCTTCAGGATCAATTAACATTGTATCAGTTGAAGAGAAAGGATTAA

CTTTAGCAGATACACAAGGCATGTTCCTGCTTCTAGCTGCTGGTTTCATTCTAGGCGCTTCAGCATTAATCTCCGAATGG

ATGGGGGGCTGTTCCAGAAGATGTCGTTTGAGAAAGAAAGACGATACTCCATCTAGTGCAAATTCTAGAGAACACTTGGT

ATTAACACCTAAAACAGATATTGAATCTGAAATTAAAGTTATATCTGATAGTGCCGACAGTAGATTTCATCTGAACCCTA

GGACTGCTAGCGCAGATTCTAAAGGCAGCCTTGATGGTGCAATTATTAATTTAACAAAGGATAATATCAGTATTCATGAT

AATTTCTCCGTCGATGGATGGGGTTCTAGAAGGTCCAGTTCAGTTGATATCGATAGGGAAGTCAAAGAGATATTTGAGAA

AGATGAAAACAGAAGGAGGGTTAAGTCTACAGCGGGTGTGGAGCTAACAGATAGCCAAAGGCAAGCAACAGCTTCTAAAG

GGGCCTTTGGTGCACATCTATCTGATCCATAA

>Cher_IR25

ATGTCTTCACTCACCATCTTGCTTCTATTCCTGTTCGTTCCAGTTACGCTTTCTCAGACGACGCAAAATATAAACGTCCT

GCTTATCAATGAAGAGAATAATGCGTTAGCTGAGAAATCTTTCGAAGTAGCGAAGGAGTATGTGCGGAGAAATCCAACTC

TCGGTTTGGCCGTTGATCCTGTGATTGTGGTAGGCAACAGAACAGACGCTAAGGCTTTTTTAGAGAATGTTTGCAGAAAA

TATAACGACATGTTGTCAGCGAAGAAAACTCCCCACGTTGTCCTCGATTTTACAATGACCGGCGTCGGCTCGGAGACCAT

AAAGTCGTTTACTGCAGCATTGGCCCTGCCCACTATTTCGGGCTCGTTCGGGCAGGCCGGTGACTTGCGCCAGTGGAGGA

ACCTGAATGCTAACCAAACCAAGTTTCTTCTGCAAGTGATGCCGCCTGCTGACATCCTTCCAGAATCAATCAGAGCCATA

GTCACTAAGCAAGATATTACAAACGCTGCCATCATTTTTGACGAGTTCTTTGTCATGGACCATAAATACAAATCGCTCCT

GCAAAACATCCCGTGCAGGCACGTCATAACACCGATCAAAAGTTTTAACAAAGACGAAATTAAAACGCAACTGAGAAGTT

TGCGGGAACTGGACATTGTCAACTTCTTCGTGGTTGGGAATCTACGGACTATAAAGAATGTTCTAGACGCAGCGGACGAG

AATCAGTACTTTGGGAGGAAAACTGCGTGGTTCGCTTTATCTCTGGACAAAGGGGATATAAGCTGTGGGTGTAAAGACGC

CACCGTAGTTTATATGAAACCAACCCCCGACGCAAAGAGCAGAGATCGCCTCGGCAAAATCAAAACAACGTACAGCATGA

ACGGCGAACCTGAAATCACATCTGCGTTCTACTTCGACCTCTCCCTGAGAACGTTCCTGACAGTCAAATCACTGCTGGAC

TCTGGCAAGTGGCCAAATGATATGCGATATATTACTTGCGACGACTACGACGGCAAGAATACCCCCAACCGGACCTTGGA

TCTGAAATCGGCGTTTCAAGAGATAAAAGAAACACCGACGTACGCGCCGTTTTTTATTCCCGAAGACGATCCAATGAACG

GGAGGAGTTACATGGAATTTAGTACCGATCTGACGGCAGTTACCGTGAAGGACGGCGCGTCTATAGGTAGCCGCTCGCTC

GGTTCCTGGAAGGCAGGCCTCGCGAACCCCCTGTCGTTGACCGATCCGGAAAATATGAGCGATTACTCTGCGCAGCTCGT

GTTTAGAGTGGTCACAATAGAGCAAAACCCTTTTATTATAAGGGACGACGACGCGCCGAAGGGGTATAAAGGTTACTGTA

TCGATCTTATAGAGGAGATACGTCAAATTGTGAAGTTCGACTATGAAATAACGTTAGCGCCTGACGGTAATTTCGGGACT

ATGGACGACAACGGCAACTGGAACGGGATTATCAAGGAGCTCATTGAGAAACGGGCGGACATCGGTCTGACGTCACTGTC

AGTGATGGCAGAGAGAGAAAACGTGGTAGATTTCACTGTGCCCTACTACGATTTAGTAGGGATCACTATCCTCATGAAAC

TGCCCCGAACACCGACCTCCCTTTTCAAATTTTTAACGGTCTTAGAGAACGATGTCTGGCTGTCCATATTGGCTGCGTAT

TTCTTTACTAGTTTTCTCATGTGGGTATTCGACAAATGGAGTCCGTATAGTTACCAGAATAACCGCGAAAAGTACAAAGA

TGACGAGGAAAAACGCGAATTCAACCTAAAGGAGTGTCTATGGTTTTGCATGACGTCACTCACCCCCCAGGGAGGGGGGG

AGGCGCCCAAAAACCTCTCTGGCCGGTTACTGGCCGCGACTTGGTGGCTGTTTGGGTTTATCATCATAGCGTCATACACT

GCTAATTTGGCGGCGTTCCTTACGGTGTCAAGACTGGACACGCCTATTGAGTCCTTGGATGACCTGTCAAAGCAATACAA

GATTCAGTACGCCCCGCTCAACGGGTCCGCTGCTATGACGTACTTCGAACGCATGGCGCATATCGAAGTCAGATTTTACG

AGATATGGAAAGAGATGAGTTTAAACGACAGCTTGAGCGACGTGGAGCGAGCAAAGCTAGCCGTGTGGGACTATCCCGTC

AGCGACAAGTACAGCAAGATGTGGCAGGCTATGAAGGAGGCTGGGCTGCCTAACTCTGTGGAGGAGGCTGTTCAGAGGGT

GCGAGACTCCAAGAGCTCGAGCGAGGGCTTCGCCTGGCTCGGAGACGCGACGGACGTGCGCTACCACGTGCTCACCAGCT

GCGACCTGCAGATGGTGGGAGACGAGTTCTCACGGAAGCCCTACGCCATTGCTGTGCAACAGGGGTCGCCGTTGAAGGAC

CAGTTTAACAACGCGATCCTCCAACTCCTGAACAAACGGAAGCTAGAGAAGCTCAAGGAAAACTGGTGGAACAACAATCC

TGAAGCAATGAAGTGCGAGAAACAGGACGACCAGTCAGACGGGATCTCCATCCAGAACATTGGGGGCGTGTTCATTGTCA

TTTTCATGGGTATTGGACTGGCTTGTATTACCCTGGGGGTGGAGTATTGGTGGTACAAGTGGCGGAAACGGCCGTTGGTT

GGGGATGTTACACAGGTGGAACCCTCAAAAACAACAAGAAACAATGCTGACCACGGAACCACGAAAATTGGTGACGGATT

CACGTTTAGATCAAGAAATTTAGGTCTTTCTAACCTAAGATCCAAGTTCTAA

>Cher_IR41

ATGTTAGTACCAAGCACGTTACATCCGGTAGAAACATTACTCCAAATTTTAATAAATAAGTACTTGCTGGTATCCTATTG

CGTAACGTTAGTTACGGAGACAGAATTAACATTCCGTCCGCCATCAAACCTAAGCTTCATGTATATTCATCCCGAGTCCA

ACCTCACGGATCAACTCTTGGATGCATCAGAAAAAGGCTGCTCCGATTTCATTATACAAATGAACGAACCTGAGATCTTC

ATGGCTGCTTTTGAAATGACTAACCATTTGGGAGACATAAGAAGAAGCGACAGAAAGTTGATATTCTTACCTCTACAAGA

TAAGGTTTTCAACGGTAGTGCTCTTGATATTTTGGGGCTCAAGGTGACAGGATTCGTAGCAAACATTTTGCTAGTTCTAC

CATCGGCGAATTGCCCAGAAGACTGCGATTACTATGACTTAGTGACGCATAATTTCGTTGGCCCGGATGCAGAGGTCGAT

CAGCCTCTTTACTTAGCCCGATGGGACTTTGGCCTAGAGCAGTTCGACGAGAGTGTCAATCTGTTTCCTCATGACATGTC

TAACATGAACAGCAAGACGGTCAAGGTAGCTGCTTTTACGTATAAGCCATACGTATTGCTTGATCTTGATCCGTCAGTAA

ATGCTTTTGGTCGAGATGGCATAGAAATGAGGATCATTGAAGAATTTTGCAGATGGGTAAACTGTACCGTGGAAATTGTA

AGAGACGATGAAAACGAGTGGGGCGAAATATATGACAATCTCACAGGAGTTGGGATTTTAGGCAACGTCGTTGAAGATCG

AGCCGATATGGGAATCACGGCTCTGTATTCGTGGTATGAGGAATACAGAGTGATGGATTTTTCGGCTGCAGTTGTTAGAA

CGGCTATAACTTGCATCGCACCTGCAGCCAGGATCCTATCAAGTTGGGATCTACCATTCCTGCCTTTCGCTGGGTTGATG

TGGCTGTGCCTTATTTTCACATTCTTCTATGCTGCCTTTGCTCTGTTCATAGCCCAGCGCTCAACCGACAAGATATTTTT

ATCGACATTCGGGATGATGATTACACAAACACGAGACGACTCCGGAGACTCATGGCGCATACGCAGCATCACAGGCTGGT

TGCTGGTCACCGGTCTGATCATAGACAACGCGTACGGGGGTGGCTTGGCTTCTAGCTTCACCGTGCCCAAGTACGAAGCC

TCCATAGACACGGTCCAGGATATGGTGGACCGGAAGATGGAGTGGGGGGCTACTCACGATGCTTGGATATTTTCTATTAT

TTTATCAGAAGAGCCTCTTGTTAAGAAATTAATCAATCAGTTTAAAACGTATCCTGCTGAAGAATTAAAACGGAAGAGTT

TTACACGGAGCATGGCTTTCAGCGTCGAACATTTACCGGCAGGAAGCTTCGCAATCGGCGAGTACATCACACAAGAAGCG

ACGGAGGACCTAGAGCTGATGCTAGAAAACTTCTACTACGAGCAGTGCGTGGTGATGCTGCGCAAGAGTTCCCCGTACAC

CGCCAAGCTGAGTGAGCTGGTGGGCCGGCTCCACCAATCAGGGCTCTTGCTGGCTTGGGAGACACAGGTCGCTTTAAAAT

ATCTCGATTTCAAAGTACAACTAGAAGTTAAACTCTCACGCACCAGGCGAGATGTGGACGAAGTAGAGCCATTGAGCTTA

AAACAAATCCTGGGTATCTTCATAATATACTTCGGTGGTTTGTTTATATCTTTAGTTTGTTTCCTGGTTGAAATACTTAC

AAAATGCAGGAAAACTTCCATTGTAATATAA

>Cher_IR68

ATGTGGAAATGTTTAGTTTTTGTCGTGTTGGTTGTATCCTGCGTTTCCTCAGAGATATCTCCCATCATAGAAGACATCGA

AGCGAGCAAGGACCTCGAGTCAGTGCTCATAGACCTGCTGAGCGGCCTGTCTCGCCGCCGCGACGTGGCGTGCGCGGCGA

TCGTATGCGACGCGGTCTACTTGAACGTGTTTGAGGGCGCGCTGTTCAAACGCACGCTAGATGTCTCTGTGGTTATGATA

GTAGTAGAAGAATATGAAGATCTCCTTTCTCCAAACTTCTACACGCTGGAGTCGCTGCGGCAAGCGAGGAAAGACGGGTG

CAACGTTTATATCATTTTATTGGCCAATGGCCTGCAAGCAACACGATTGCTTAGATTCGGTGACAGGCATCGAGTCCTAG

ACACCAGAGCAAAATACATAATGCTCCACGACTTCAGACTCTTCCACAGCGACCTTCACTACATATGGAAGCGTATCGTC

AACGTAGTCTTTCTCCGATACCATAAGAAGATACACGGGTCAGCGAAAGGCAAAGCCTGGTTCGACCTGTCCACTGTACC

GTTTCCGAACCCTATTAAGACCATATTTGTGTCGAGAAGAGTGGATATTTGGAGGAATGGGAGATTTCATTATAACAGAC

CGCTATTTGCAGATAAAACGAAGAATCTAAATGGTGAAGTGCTGAACGTAGTTTACTTGGACCACGTGCCTTCTGTTGTT

GTTGTTATGAAAAACAACGACACCAACAAAATCGGCGGCGTTGAAATTGAAATTCTTAACACCGTAGCCGAGAAAATGAA

TTTCAAACCGCATCTGTACCAAGCAATGAATGCTGAATACCATAAGTGGGGGCAGAAGCAAGCTAACGGGTCCTTTTCAG

GTCTTCTCGGAGAGATGGTGCACGGCCAAGCAGACGTGGCGCTGGGCAACCTACAGTACACCCCGTACCACCTGGAACTG

CTGGACCTCAGCATCCCCTACACCTCGCAGTGCTGGACCTTCCTCACGCCCGAGGCGCTCACTGACAACTCCTGGAAGAC

GCTGATATTGCCTTTTAAACTGTACATGTGGATAACCGTGCTGCTGGTGTTGCTGATAACGGGTGCCATTTTTTACGGCC

TGGCGAGGTTCTACATGCATCTCCTGCAATACCAGAGCGACCATAGTCCCGTTGTTATTACTGACAAGGAAGAAGCAGAC

GAATATTTCGACGAGAAACCCGTCGGCATGTACCTGTTTGGCGATATAATTAACAGCATACTGTACACGTACGGAATGTT

GCTAGTCGTTTCCCTTCCCAAGTTACCTACTGGCTGGGCTATCAGACTTCTTACGGGCTGGTACTGGCTTTACTGCATAC

TCCTGGTGGTTTCTTACCGAGCTAGTATGACTGCGATCCTAGCAAACCCAGCCCCTAGAGTCACCATAGACACGCTGCAA

GAACTAGTGGACAGCAAAGTGACCTGCGGCGGCTGGGGGGCTGAAACTAAGCATTTCTTCGAAGAGTCCATAGACGAGAT

CGGACAGAAAATTGGGGAACGCTTTGAAATGATTGACGATCCTGACGAAGCTGCTAACAAGGTAGCTAAAGGCGTGTACG

CTTATTACGAGAATGAGTATTTTTTGAAGTATTTGAGCGTGAAGCGGAAGAATTCGGATGAGAAAATGAATATAGAAAGT

CAAAATTCGACAAACGCAACCGTTCAGGTCAAACAAGAGTCTGATAGGAACTTGCATATAATGACGGATTGTGTTGTAAA

CATACCTATTTCACTAGGTTTTCATAAGAACTCTCCTCTGAAACCTTTAGCTGATGTCTATATGAGAAGAACAGTTGAAG

TTGGTTTAGTGGGAAAATGGATGAATGATGTTATGTACCCTTTAAGAGCTTTAGACGCCACGGACAATGAAATCAAAGCT

CTCATGAACCTGAAAAAACTGTACGGAGCTTTTATAGCTTTAGCTATCGGTTATGTTCTAAGCTTAGTTTGTTTAATTGG

TGAACTTATTTACTGGAATTGTATTGTGAAAAGAGATCCTAGATTCGATAAGTATGCAATGGACCTCTATTATAAAAAGA

AAAAAATAGTTGCTTAA

>Cher_IR75b1

ATGAAGACTTTCTTTTTATTAAACTTTGCTTTATGCTTTCGTTTAGTATTTAGTTTGGGCAGCGACGATATTAACATGAT

AGTCTCATTTTCCAAACAAGATGGTAGACCTACGTCTGTTCTGGCACCTCACGTTTGCTGGAGCTCAGACGAACTGGCTT

CCCTGGCGAAGAGTCTCCACGAGGTCGGCGTGAGCGTGGCCGGGTCCCTGCAGCCGCGCAGGACGGAGTACTACCTGCAG

AACCTACTCGTCCTGGCCGACCTTGGCTGCAGAGGGACTGAGGAGTTCCTGGTCAAGGCCAGCGACGATGGTTTCTTCAA

GTCCCCGTACCGCTGGCTGCTGGTGACCAAGGATGCAGAAGACCTGGACATTCTGGAGCGTCTCGTCATGCTGACGGACA

GCGATGTGGTCCTGGCGCAAAAGACCAATGACAGCTATGAGCTGATAGAAGTGTACAAAATCATCGCCAACTCCGAACTG

ATTTTCAATACTCGAGCTGTGTGGCGAGCCAGCAACGGAACTATCGCAAATACGACAGTAACTGACGGCACCGATACGGC

TCTTGCCACCAACGACAAAATCACCAGTATGACTGTAGTGGAAGACAAATATGGAGTTCTAGAAGACCATAGAGCGAGCA

AAGTTCTGTCTACCAGGAGAATGGACATAAGAAAGCATACGCTGACTATGTTGAATGTGATAACGGACAGCAACGACACG

ATGAAGCACATGGCTGATAGATTGCAACTCCACCAGGACTCCATAACTAAGATGACGTGGATGGTGATAAGGATCTGCTT

CGAGATGATGAACTCGACGGAGAAGATGGAATTCTACAACACTTGGGGCTACGTTGACAAGAATGGGTCGTGGAACGGAA

TTGTAGAGCAGTTCATCAAGAAACAAGGGGACTTGGGAACGCAAATGCTTTTCACTCAGGCACGCATGCAACTCATCGAC

TATGTCGCGATGGTCGGTACAACGGGTATCCGATTTGTTTTTAGAGAGCCGCCACTCGCCTACGTGTCGAACATATTCGC

GTTGCCCTTCGCCGGCTCTGTGTGGTTGGCAAGCTTTGCGTGTGTGCTTGCTTGCGCTCTGTTCCTATACGTCACGTCCA

AATGGGAAGCCACTATGGGTGTGCACCCTACGCAACTGGACGGGTCGTGGGAAGACGTACTGATCCTGATAATTGGCGGT

GTGCTGCAGCAGGGGTGCATTCTGGAGCCGAGGTTCGCAGCAGGTCGTATGGTAACTCTTCTGCTGTTCATCGCCCTCAC

GATTCTGTACGCGGCTTACACAGCCAACATCGTGGTGCTCCTCAGGGCTCCAAGTTCCTCAGTCAAGAATCTCCAGGACA

TGCTGGACTCTCCTCTGGAACTGGGCGCCAGCGACTTCAATTATAATAGATATTTTTTTAAGCAATTGAATGAGCCGCTG

CGCAAAGAGATATATAACAAGAAGATTGCTCCAAAAGGCAAGAAGGCCAACTTTTACACAATGGAGGAGGGGGTGGAGAA

GATACGACGGGGTCTATTCGCCTTCCACATGGAGCTGAACCCCGGATACCGTCTCATCCAGGAGACTTACCAGGAAGAGG

AGAAGTGTGACCTGGTGGAGATGGACTACATCARCKWRATAGATCCCTGGGTCCCGGGGCAGAAGAGATCGCCTTACAAG

GATATGTTTAAGATCAACTTCATAAAAATCCGCGAAGCCGGCCTCCAAGCGGCGATCCACCAGCGCCTGCACGTCGGCAA

GCCGCGCTGCCTGGGCGCCGTCAGCACGTTCAGCAGCGTCGGCGTCACCGACATGTACCCGGCAATGCTGGCCACGCTGT

ACGGCGTGCTGCTCGCGCCCGCTGTGCTGCTGCTCGAGCTGGCCTATCACCGGCTAATTATTATCAGAGACAAAAGAATA

GCGAAGCGTCATTGTAAACAAAATGCTACC

>Cher_IR75b2

ATGAAGACTTTCTTTTTATTAAACTTTGCTTTATGCTTTCGTTTAGTATTTAGTTTGGGCAGCGACGATATTAACATGAT

AGTCTCATTTTCCAAACAAGATGGTAGACCTACGTCTGTTCTGGCACCTCACGTTTGCTGGAGCTCAGACGAACTGGCTT

CCCTGGCGAAGAGTCTCCACGAGGTCGGCGTGAGCGTGGCCGGGTCCCTGCAGCCGCGCAGGACGGAGTACTACCTGCAG

AACCTACTCGTCCTGGCCGACCTTGGCTGCAGAGGGACTGAGGAGTTCCTGGTCAAGGCCAGCGACGATGGTTTCTTCAA

GTCCCCGTACCGCTGGCTGCTGGTGACCAAGGATGCAGAAGACCTGGACATTCTGGAGCGTCTCGTCATGCTGACGGACA

GCGATGTGGTCCTGGCGCAAAAGACCAATGACAGCTATGAGCTGATAGAAGTGTACAAAATCATCGCCAACTCCGAACTG

ATTTTCAATACTCGAGCTGTGTGGCGAGCCAGCAACGGAACTATCGCAAATACGACAGTAACTGACGGCACCGATACGGC

TCTTGCCACCAACGACAAAATCACCAGTATGACTGTAGTGGAAGACAAATATGGAGTTCTAGAAGACCATAGAGCGAGCA

AAGTTCTGTCTACCAGGAGAATGGACATAAGAAAGCATACGCTGACTATGTTGAATGTGATAACGGACAGCAACGACACG

ATGAAGCACATGGCTGATAGATTGCAACTCCACCAGGACTCCATAACTAAGATGACGTGGATGGTGATAAGGATCTGCTT

CGAGATGATGAACTCGACGGAGAAGATGGAATTCTACAACACTTGGGGCTACGTTGACAAGAATGGGTCGTGGAACGGAA

TTGTAGAGCAGTTCATCAAGAAACAAGGGGACTTGGGAACGCAAATGCTTTTCACTCAGGCACGCATGCAACTCATCGAC

TATGTCGCGATGGTCGGTACAACGGGTATCCGATTTGTTTTTAGAGAGCCGCCACTCGCCTACGTGTCGAACATATTCGC

GTTGCCCTTCGCCGGCTCTGTGTGGTTGGCAAGCTTTGCGTGTGTGCTTGCTTGCGCTCTGTTCCTATACGTCACGTCCA

AATGGGAAGCCACTATGGGTGTGCACCCTACGCAACTGGACGGGTCGTGGGAAGACGTACTGATCCTGATAATTGGCGGT

GTGCTGCAGCAGGGGTGCATTCTGGAGCCGAGGTTCGCAGCAGGTCGTATGGTAACTCTTCTGCTGTTCATCGCCCTCAC

GATTCTGTACGCGGCTTACACAGCCAACATCGTGGTGCTCCTCAGGGCTCCAAGTTCCTCAGTCAAGAATCTCCAGGACA

TGCTGGACTCTCCTCTGGAACTGGGCGCCAGCGACTTCAATTATAATAGATATTTTTTTAAGCAATTGAATGAGCCGCTG

CGCAAAGAGATATATAACAAGAAGATTGCTCCAAAAGGCAAGAAGGCCAACTTTTACACAATGGAGGAGGGGGTGGAGAA

GATACGACGGGGTCTATTCGCCTTCCACATGGAGCTGAACCCCGGATACCGTCTCATCCAGGAGACTTACCAGGAAGAGG

AGAAGTGTGACCTGGTGGAGATGGACTACATCAGCGAAATAGATCCCTGGGTCCCGGGGCAGAAGAGATCGCCTTACAAG

GATCTGTTTAAAATCAATTTCATAAAAATCCGCGAAGTCGGCCTCCAATCCGCCATCCACCAGCAAATACAGGTCGGCAA

GCCGCGCTGCCAGGGCGAGGTCAGCACGTTCAGCAGCGTCGGCGTCACCGACATGTACCCGGCCATGCTGGCCACGCTGT

ACGGCGTGCTGCTCGCACCCGCCGTGCTGCTGCTCGAGCTGGCCTATCACCGGCTGATTTTGATCAGAAAAAAAAGACTA

GCGAAACTTCAATGTGAACAAGACACCACATAA

>Cher_IR75c1

ATGAAGAAGTACAATTTAATGGTGTCACTAATATTTTTTTCAGGTTGTTACGCGGAAAATGAATCAAAGCTATTTATGGT

AGCCGACGTCATCCGATCTATGCAAAGGCCGTCGTCCGTGATCGCCATGCTTTGCTGGTCTTCACGTATGAAGATACAAC

TATATTCGGCCCTGGGAGAAAATGACACGCATATTAATATGATGCAGGTTTTGAAAAGTGGGACAGTGCCTCAGAGGCAT

GCTCAGGATCAACATATTGTGTTCCTAGCTGACTTGGACTGTCCTGGAATATCTTCGTACTTTGCTATGAGCAGTTCAGA

AAAGCATTTTCGATCTCCCTTTCGTTGGCTTCTTGTCGGCACTGACTACAATGACACCGGAGTGGAGGGAAGCTATATAC

CTGAGGCATTAGCTGGTGTTGGTATACTGGCGGACTCTGAAGTGATTCTGGCTCAGCATTTGGGGAACGGGTCATATGAA

TTAAATCTGATATACAAAGTAGGCGTCAATACAACGTGGAAAAAAGAACTCTACGGCACTTGGGACGAAGACAAAAGACT

CCAAACAACACTAATGGAAGGCGAAATAGTTCAACGGAGACTCAATCTGGAAAACTATGAGATACCTATCTCTTACGTCC

TTACCGACAATGACAGCATTAACCATCTCTTTGATAATGTAAACGATCACATAGACACAATAACGAAGGTAAATTTCCCA

ACAACTAACCACTTGCTCGACTTCCTGAACGCGAGCAGGAAGTATGTGTTTGCAAACACCTGGGGCTACAAAGTCAACGG

GACTTGGAATGGGATGACTGGGTACTTAGTACGAGAAGAAGTCGAAGTAGGAGGATCACCAATGTTCTTCACAACTGAAC

GTATATCGATCGTCGAGTACATATCGAGCCCTACGCCGACGCGGTCGAAATTCGTGTTCCGGCAACCGAAACTGTCGTAT

GAGAACAACTTGTTCCTGCTCTCATTCCGAGCGGCTGTGTGGTACTGCACCATAGGACTGCTCATTTTGCTGGTCTTGGC

TGTATTCATAGTTGCGGTTTGGGAATGGAAGAAGACGCATGGACATGACAATAAAAAGAGCGAGTCTGATCCTAGTATCT

TGAGGGCTAATATGATGGACGTTATTGTATTAATATTGGGCGCTGCCTGTCAGCAGGGCAGTACGGTAGAACTTAAAGGT

TCCTTAGGGCGTGTGGTGATGTTGGTACTGTTCCTGGCTCTCATGTTCCTGTATACTTCGTATTCGGCCAATATTGTAGC

ACTACTGCAGTCCAGCTCATCCCAAATAAAAACACTGGACGATATACTACATTCCAGAATCAAATTTGGTGTCCATGACA

CGGTCTTCAACAAGTATTATTTTTCAACGGCCACCGAGCCTGTCAGAAAGGCCATTTATGAAACAAAGGTGGCGCCTCGA

GGCTCCACACCTAGATTCATGACTATGGAAGAAGGCGTTAAGAAAATGCAAAAGGGTTTATTCGCATTTCACATGGAGAC

AGGTGTCGGCTACAAATTCGTGGGAAAGTACTTTCAGGAGTCTGAGAAATGTGGTCTAAAAGAGATACAATATTTGCAAG

TAATAGACCCGTGGTTGGCTGTTAGGAAAAATACTCCTTTTATGGAAATGTTTAAGATAGGAACTAAACGAATTCAAGAA

CATGGATTGCAGAGCAGAGAGAACCGTTTGCTGTACGAAAAGCGACCTAAGTGCTCGGGCCAAGGGGGCAGTTTCGTATC

CGTGAGCATGGTGGATTGCTATCCTGCTTTATTGGTGCTCTCTTATGGCAGCATTCTGGCTGTTATGATACTCTTCATGG

AAATTATTAGGGCTAGAAAAAGTATTATTTTAAGCAAAATCTGTCGCGCGACAGAAACTGAGGATGAGGATTAA

>Cher_IR75c2

ATGAAGAAGTACAATTTAATGGTGTCACTAATATTTTTTTCAGGTTGTTACGCGGAAAATGAATCAAAGCTATTTATGGT

AGCCGACGTCATCCGATCTATGCAAAGGCCGTCGTCCGTGATCGCCATGCTTTGCTGGTCTTCACGTATGAAGATACAAC

TATATTCGGCCCTGGGAGAAAATGACACGCATATTAATATGATGCAGGTTTTGAAAACTGGGATGGTACCAAAACGGCAT

GCTCAGGATCAACATATTGTGTTCCTGACTGATTTGGATTGTCCAGACGTATCTACGTACTTTGCAATGAGTAGTTTAGA

AAAGCATTTTCGGTCTCCATTTCGTTGGCTTCTGGTCGGCACTGACTACAATGACACCGGAGTTCAAGGAGACTATATAC

CTGAGGCATTAGATGGCGTTGGTATACTGGCGGACTCTGAAGTAATTCTGGCTCAACATTTGGGGAACGGGTCATACGAA

TTAAATCTAATATACAAAGTAGGCGTCAATACAACGTGGAAAAAAGAACTCTACGGTACTTGGGACGAAAACAAAAGGCT

CCAAACAACACTAATGGAAGGCGAAATAGTTCAACGGAGACTCAATCTGGAGAACTATGAAATTCCTATCTCTTACGTCC

TTACCGACAATGACAGCATTAACCATCTCTTTGATAATGTAAACGATCACATAGACACAATAACGAAGGTAAATTTCCCA

ACAACTAACCACTTGCTGGATTTCCTGAACGCGAGCAGGAAGTATGTGTTTGCAAACACCTGGGGCTACAAAGTCAACGG

GACTTGGAATGGGATGACTGGATACTTAGTACGGGAAGAAGTCGAAGTCGGAGGATCACCAATGTTCTTCACAACTGAAC

GTATATCGATCGTCGAGTACATATCGAGCCCTACGCCGACGCGGTCGAAATTCGTGTTCCGGCAACCGAAACTGTCGTAT

GAGAACAACTTGTTCCTGCTCTCATTCCGAGCGGCTGTGTGGTACTGCACCATAGGACTGCTCATTTTGCTGGTCTTGGC

TGTATTCATAGTTGCGGTTTGGGAATGGAAGAAGACGCATGGACATGACAATAAAAAGAGCGAGTCTGATCCTAGTATCT

TGAGGGCTAATATGATGGACGTTATTGTATTAATATTGGGCGCTGCCTGTCAGCAGGGCAGTACGGTAGAACTTAAAGGT

TCCTTAGGGCGTGTGGTGATGTTGGTACTGTTCCTGGCTCTCATGTTCCTGTATACTTCGTATTCGGCCAATATTGTAGC

ACTACTGCAGTCCAGCTCATCCCAAATAAAAACACTGGACGATATACTACATTCCAGAATCAAATTTGGTGTCCATGACA

CGGTCTTCAACAAGTATTATTTTTCAACGGCCACCGAGCCTGTCAGAAAGGCCATTTATGAAACAAAGGTGGCGCCTCGA

GGCTCCACACCTAGATTCATGACTATGGAAGAAGGCGTTAAGAAAATGCAAAAGGGTTTATTCGCATTTCACATGGAGAC

AGGTGTCGGCTACAAATTCGTGGGAAAGTACTTTCAGGAGTCTGAGAAATGTGGTCTAAAAGAGATACAATATTTGCAAG

TAATAGACCCGTGGTTGGCTGTTAGGAAAAATACTCCTTTTATGGAAATGTTTAAGATAGGAACTAAACGAATTCAAGAA

CATGGATTGCAGAGCAGAGAGAACCGTTTGCTGTACGAAAAGCGACCTAAGTGCTCGGGCCAAGGGGGCAGTTTCGTATC

CGTGAGCATGGTGGATTGCTATCCTGCTTTATTGGTGCTCTCTTATGGCAGCATTCTGGCTGTTATGATACTCTTCATGG

AAATTATTAGGGCTAGAAAAAGTATTATTTTAAGCAAAATCTGTCGCGCGACAGAAACTGAGGATGAGGATTAA

>Cher_IR75d

ATGTTTCTCTATATCTCGTTGTGCGTTTGGTTTACAATCACACGCGCGTATAATTTGAATGGCGAATTTGTAGAAGATTT

CCTTCAACGCGAAGAAAGGCCTGCTACCATCGTCTCATATTTACAATTGACTAAAAAAGAACATGTGCAACTCAGCAAAT

CATTTTTCGCAAACAATAAACAATTTCAACTAGCACCTGACACTGCACTTACAAGTAATAAACATGTTGTACATTTAGCT

GATTGCAAAAATAATGACACCTGGGAATTGTTGCAAAACGCGATTACGGATAATTCAATCAGGTGCCCAGCGCGATGGCT

ATTGTTACTGACTGACAACCTCGGAAATGATTTAGATTCTGCAGCCAGTGACGACTATAAATCACACATAGAACGTCGCT

TACATAACATTTACGTCCATATGGATAGCGAGGTTTACATCGCATTTCAGCGACTAAATTACGTTCAATTGTTTTCAGTG

TACAAAATAAAACCTCATCTTCCGTTAATATGGGAGGACCATGGCCTTTGGTCGAGAGGTGGCTTCAACAAGCCTCGGCC

CGAGCCCCTGGCCATGCGCAGACGCAACATGAGGGGCGCTGACGTCGTCGTCGCGGCCGTCATATTGGACAATCGGACCG

TGGAATACATGCCTGATTATCTGCACCGAGAGGTGGACACGCTCACGAAGCTGATGTACCACATGACGCTCCACCTGGTG

GAGTGGGTGAACGGCTCGCACGTGCTGAACCGGACCGGCTCGTGGGGGTTCGTGCAGCCGGACGGCCGCTACGACGGCGT

GGTGCGGGAGATGCAGGACGGCCGTGCTGACTTGGCAGGTACGGTCATGACACCAACAACAGAACGAACAAAATTCATGA

ACTTCGTGCTGGCCCCAGCGCCCGTGGAAGCCAAGTTCATCTTCAAAAAGCCGGCTCTCGCGTCGGTGACCAACATCTAC

GTGCTGCCCTTCAGCATTGGTGTTTGGATGTCTATCTTGGTTCTGATTCTCGTGTCCAGCTTGACTTTATTCCTGGCCTA

CTACCGGGAAGAAGTCGTCAATCCGCGCTACGACATGTCGTGGCCCCAAAAAGTTTCAGAAGGATTATTTGAAACGTTGT

GTTTAGTATTTCAACAAGGAACTGCATCTGACCCCATTTCAATCGCTGGGCGTCAAATCCTGCTGCTCGGCCTCGTGGCC

TTCATGTTCCTGTACACGGCGTACTCGGCCAACGTGGTGGCGCTGCTGCAGTCGCCCAGCAACGACATCGGCAGCGCCGA

GACGCTGCTGGCCTCGCCGCTCGCCTGCGGCGCGCAGGACGTGCAGTATAATCGGAAGATGTTCGCGCATGAGACACGGC

CCATACATCGAGCTTTGAGCTCTCGAAAAATACTACCGCAAGGAGAGAAAGCATACCTTACCGTCGATGAAGGAATACGC

AAAGTCAGAGAGGGTATGTACGCGTTCCACGTAGAGACGACGGCGGGCTACGACCAGATCCAGAAGACGTTCCTGGAGGA

CGAGAAGTGCAACCTCGGCGCCATCAAGTACATGTCCATCGCGTACCCGCTGCTGGCGCTGGCGCAGGGCTCGCACATCA

AGGAACAGCTTCGAATCGGTGCCCACCGTTTGATGGAAGGCGGCGTGCAGCGGCGCACGGTGCGG

>Cher_IR75e

ATATCGCAGCCGCAGTACTTCAAGGGCTGGGCGGACCTGACGATGCGGCACATCGACACCTTCCCCAAGCTCACCTATCC

GCTCATGCTGCTGCTCGCCGAAGACCTTCACTTCAGGTACAACTTACGGCAGGTGGACCTGTACGGCGAGGAGCGCAACG

GCTCGTTCGACGGGCTGGCGGGGCAGCTGCAGCGGCGCGAGATCGAGGTCGGCATCACCTCAATGTTTCTACGGGCAGAC

CGCACGCGCGTGCTGCACTTCTGCTCCGAGACTGTCGAGCTCCGGNNNNNNNNNNNNNNNNNNNNNNNNNNNNNNNNNNN

NNNNNNNNNNNNNNNNNNNNNNNNNNNNNNNNNNNNNNNNNNNNNNNNNNNNNNNNNNNNNNNNNNNNNNNNNNNNNNNN

NNNNNNNNNNNNNNNNNNNNNNNNNNNNNNNNNNNNNNNNNNNNNNNNNNNNNNNNNNNNNNNNNNNNNNNNNNNNNNNN

NNNNNNNNNNNNNNNNNNNNNNNNNNNNNNNNNNNNNNNNNNGTCCCGGCGCTGGCGTCGGCGCGCGTGGCGATGTTCTG

CGCGCTGCTGACGTCGCTGTTCGCGTTCACGGCGTACTCGGCCAAGATCGTGGTAATCCTGCAGACGCCCAGCGCGGCTA

TCCGCACCATCGACGACCTCGCGCGCTCGCCCATGACGCTCGGCGTGCAGGAGACCACSTACAAGAGGGTKTAYTTYGCG

GAGAGCACGCAGCCGGCGACGCAGCGGCTGTACCGGCGCAAGCTGCTGCCGCTGGGCGAGCGCGCCTACCTCAGCGTGGT

GGACGGCGTGCAGCGCCTGCGCACCGGCCTCTTCGCCTTCCAGGTGGAAGAGCCGTCAGGCTACGATATCATCAGCAAGA

CGTTCACGGAGCGCGAGAAGTGCGGGCTGCAGCAGATCCAGGCCTTCAAGATGCCCATGGTGGCCGTGCCCATCCGCCGC

CACTCCGGGTACAAGGAGCTRTTCGCCTCCAGG

>Cher_IR75f

CTGCCGTACCGCTGGCTGGTGCTCACCAACTCACCAATCGAGGCGGCGTCGCCCTGGTGGCACCATTTCCTGGTCAACAG

CGACCTGGTGGTGGCTGAGTCGGATGGTGAACGCTACAAAATGACTGAAGTTTACAAGGCAGCCCCGACCGGGCCGCTCA

TTCTAACCCCTCGCGGCGCCTTCGACGGAGCCCTGACCGACGTGCGGCCGCACCGCGCGCTGTATCGCCGGCGCCGGGAC

CTGCGCGGGACCCCGCTGACCATGACCAACATCATACAGGACAGCAACGCGACTAGGTTCCATCTGGTGCAGGAAGACAG

GCTGAACCTTCACCAAGATTTTATGGCCAAGAACAGCTGGACTTTAGGCAAGTTAGCCTTCCTCATGTTGAACGCGACTC

CAAGAGCCATCTTCACTAATCGGTACGGGTACATACACAAGGGGGAGTGGACAGGCACCATCAGAGACCTCGTCGACAAC

AAAGCTGAAATAGGTACCAACGTGGGACTAAACGCAGACCGAATAAAGGCTGTGACATTCCTTGATAGCCTGGACAATTG

CCGGCTTCGCTTCATTTTCCGCAAACCTAATCTTTCTTACACCTCTAACATATTCTCCCTCCCTTTCTCATCGGGGGTTT

GGCTGGCCACTGCTGTGTCAGCCATTGTTGCGGCTCTGGCGCTGTATGCGAGCGTGAGGTTGACTGAGAAGCAGCCAGTG

ACGATGGGGGATGCTTTTTTGTTGAGCGTGGGAGCAGTCGCTCAGCAGGGATGCGAGTTGCAGCCGAAGAATTTGTCAGC

TCGAATAATCCAATGGGTAATCTTCGCGACCCTCATGGCTATTTACGCGGCTTATTCTGCCAACATCGTGGTGCTACTCC

AGGCGCCTTCGAACTCTATCAACAACCTGGCGCAACTGGCAAAGTCGAAAATGACTCTGGCTTGTCACGATGTGGACTAT

AACCATTTCGTTTTTAAAGGTGCTGCGGATCCAGTCCGGAAAGCTATTGCAAAGAGGGTCGATCCAGATCGTGGTCCGAA

GCCGTTCTACAGTTTGGCTGAAGGCGTCGCGAGAATGCGAAAGGGTCTGTTCGCATTCCACATCCTGTCAGAACCGGCGT

ACCGTGAGGTGGAGAAGACATTCTTCGAAGCGGAGAAGTGCGACCTTGTGGAGTTGGACCACGCAGGTTATCAGGGGTTC

TTCGTGCCCGTCTACAAGCAGTCTCCGTACATGGAGCTTTTGAGGGTTGCGTTCAAGCAAATACGTGAAGTGGGCATGAA

ATCCGCCGTGAACCACCGCCTGGAAGTGGGCAAGCCTTCTTGTAAGAGCTCTGCAGCTATGTTCATCAGCGTAGGCGCGA

TGGAGATGCGGTCCGTGCTGCTGTTCATGGTGTACGGGGTGCTGCTGAGTGTCGCGGTCGCTGGCGCTGAGATCTTCGTG

TTCCGAGCGAACCGCTATCATCAGAAGGGTAGAGCGGAGATTGATCAGAAGCCGGAAATAAAAATATCAGCC

>Cher_IR76a

ATGTCTGGGATGGAGCTTATCATTTCGTCATTCTGCAATGCCACTTTTTGTGACGTCGTCAATGATAATCCTCTTACAGG

TCAGAGTTTAGCGACAGAACGTCAAGCTGAGACTCTAGCAAACAAAAATGAACTAAACGGAAAACATTTGAAGGTAGCCA

CTTATGATAACTATCCTCTAAGCTGGGTTGCGACCTCCGACAACGGCACTCTGACGGGACGCGGAGTCGCCTTCATCATA

TTCGATATCCTGAGGGAACAATTCAACTTTACCTTCGATGTCATCACACCATCGCAAAACTTCGAAATAGGCGGCACCGA

GCCAGAACAGTCGCTAATCGGATTGGTTAATAGTACCGTAACTACCAATGTGGACATGGCGGTAGCATTCGTGCCCATAC

TGTACAAATACCAGAGCATGGTCGATTTCTCGTCGATCCTTGACGAGGGTGTGTGGAACATGATGCTGCGGAGACCCAAG

GAGTCAGCCGCCGGTTCCGGACTCTTGGCTCCGTTTGAAATTCACGTGTGGTACTTAATCCTAGCAGCAGTACTATCCTA

TGGCCCCTGCATCACTTTCCTGACCTACATCCGATCTAAGTTGGTGAAGGACGACGAGACTCACATCACCCTCTCGCCTA

GCTTCTGGTTCGTCTATGGAGCCTTCATTAAGCAGGGGACTACATTGGCTCCTGAAGCAAACACAACCCGAGTCCTCTTC

ACAACCTGGTGGCTCTTCATAATACTCCTCTCTGCGTTCTACACGGCGAACCTGACTGCCTTCCTGACTCTCTCCAAGTT

TACTCTGGACATTGAGAACCCTCAGGATATATTGAAGAAGAACTATCGCTGGGTCGCACCAGAGGGCAGCACTGTGCAGT

ATACTGTGAAAGACGTGGACTCAGATCTTTACTACCTCAGTAAAATGGTAGCCAACGGCCGAGCGCAGTTCCCTTCAGTG

AACGCAGACCGTCAATTTTTGCCTTTAGTGGCCGGGGGAGCAGTTCTGGTGAAGGAGCAGACGGCTATAGACCATCTCAT

GTATGGAGACTATGTACGGAAGACGAAGGACGGCGTTGCAGAAGCTGACAGATGTACCTACGTCGTGGCTCCGCATCCGT

TTATGGAGAAACTGAGAGGTTTTGCCTTCCCAAGAGGCAGCAAGTTAAAGCCATTGTTTGATCCTGTCCTCACATATCTT

CTACAATCCGGAATAGTGAAGTTCCTAGAACATCGAGACCTGCCAAGCACCAAGATATGTCCCCTGGACTTGCAGTCCAA

GGACCGTAAACTGCGGAACAGCGATCTCACTATGACGTACATGATTATGGTGACTGGGCTGGCTGCTGCTATTGCTGTGT

TTGTTGGAGAGATGATGATCAGACGTTACGTCCGTGTAAAGATGAAAAAGATGCACGGTGACCAGCCCAAAACAAAGCCA

TCTAAGAACGGGACCATGTCACGAAGGTACAAGCAAGACGACAGCCGACCGCCGCCGTACGATTCTCTGTTCGGGAACAA

CTCGCGATACAAAATGACGAACTCTAAAAGGAAGATTATTAACGGTAGAGAGTACTGGGTGGTTGGTACCGTTAATGGGG

AGTCCAGGTTGATACCGGTTCGGACTCCGTCGGCGTTTTTGTACCAACGCGACAAGTAA

>Cher_IR76b

ATGTCTGGGATGGAGCTTATCATTTCGTCATTCTGCAATGCCACTTTTTGTGACGTCGTCAATGATAATCCTCTTACAGG

TCAGAGTTTAGCGACAGAACGTCAAGCTGAGACTCTAGCAAACAAAAATGAACTAAACGGAAAACATTTGAAGGTAGCCA

CTTATGATAACTATCCTCTAAGCTGGGTTGCGACCTCCGACAACGGCACTCTGACGGGACGCGGAGTCGCCTTCATCATA

TTCGATATCCTGAGGGAACAATTCAACTTTACCTTCGATGTCATCACACCATCGCAAAACTTCGAAATAGGCGGCACCGA

GCCAGAACAGTCGCTAATCGGATTGGTTAATAGTACCGTAACTACCAATGTGGACATGGCGGTAGCATTCGTGCCCATAC

TGTACAAATACCAGAGCATGGTCGATTTCTCGTCGATCCTTGACGAGGGTGTGTGGAACATGATGCTGCGGAGACCCAAG

GAGTCAGCCGCCGGTTCCGGACTCTTGGCTCCGTTTGAAATTCACGTGTGGTACTTAATCCTAGCAGCAGTACTATCCTA

CGGCCCCTGCATCACTTTCCTGACCTACATTCGATCTAAGTTGGTGAAGGACGACGAGACGCACATCACCCTCTCGCCTA

GCTTCTGGTTTGTCTATGGAGCCTTTATCAAGCAGGGGACTACCTTGGCTCCTGAAGCAAACACAACCCGAGTCCTCTTC

ACAACCTGGTGGCTCTTCATAATACTCCTCTCTGCGTTCTACACGGCGAACCTAACTGCCTTTCTGACTCTCTCCAAGTT

CACTCTGGACATCGAGAACCCACAGGATATATTAAAGAAGAACTATCGCTGGGTCGCACCAGAGGGCAGCACTGTGCAGT

ATACTGTGAAAGACGTGGACTCAGATCTCTACTACCTCAGTAAAATGGTAGCCAACGGCCGAGCGCAGTTCCCTTCAGTG

AACGCAGACCGTCAATTCTTGCCTTTAGTGGCCGGAGGAGCAGTTCTGGTGAAGGAGCAGACGGCTATAGACCATCTCAT

GTACGGGGACTATGTGAGGAAGACGAAGGATGGCGTGGCAGAAGCAGATAGATGTACTTACGTCGTGGCTCCACATCCGT

TTATGGAGAAACTGAGAGGTTTTGCCTTCCCAAGAGGCAGCAAGTTGAAGCCGTTGTTCGATCCTGTCCTCACATATCTT

CTACAATCCGGAATAGTGAAGTTCCTAGAACATCGAGACTTGCCGAGCACCAAGATATGTCCCCTGGACTTGCAGTCCAA

GGACCGCAAACTGCGGAACAGCGATCTCACTATGACGTACATGATTATGGTCACTGGGCTGGCTGCTGCTATTGCTGTGT

TTGTTGGAGAGATGATGATCAGACGCTACGTCCGTGTAAAAATGAAAAAGATGCACGGTGACCAGCCCAAAACAAAGCCA

TCTAAGAACGGGACCATGTCACGAAGGTACAAGCAGGACGACAGCCGACCGCCGCCGTACGATTCTCTGTTCGGGAACAA

TTCGCGGTACAAAATGACCAACTCTAAAAGGAAGATTATTAACGGTAGAGAGTACTGGGTGGTTGGTACCGTTAATGGAG

AGTCCAGGTTGATACCGGTTCGGACTCCGTCGGCGTTTTTGTACCAGCGCGATAAGTAA

>Cher_IR87

ATGCGCTCGAGTATATTTCTACTTTATCTATATTTTATTCACTTTGCCGCAGCAAAAACCAACACTCTGCTAATGCCCTC

TGAAGATCCCGGAAAACTACTTACCGCCGCCGAATGCGTACTCAAAATGTCAGAAAAATATTTCGTTGAACATAAAGCAC

TGAGTGGCAGTATCGTCATCAGCAGTATCAACTCTGTAATTTCCATAGCACAGAGGGCAATGTACCAAACTATCCACCGA

GGAATAAAATATTCCATCATGGTAAAAGATTCTCAGTTTCCGCACCGTAATGCGTCGCATTTTCGTGAGATAGCTAAAAA

TTATTTGATCATTTTGGAAGCAAAAAATGAGCTAGAACGTGATCTGATGCAGTTAAGCCATCTCCCGACTTGGAATCCTT

TGGCTAAAACGATAGTTTTTTATCAACTCTTACCTGGCGAGGACGGCGAACAAATCTCCATAACGTTAATCAATTTGATG

AGGGACTTCAAACTTTTCAAAAGCATAATTTTCATGTTTTCACCCGAAAATGTTGAAGTAATTTCTTATTCGTGGGCTCC

TTACAGTGACACGAATTGTGGGGGCGAATGTGTTTCAGTTTATATTCTAGACAGGTGTAGGAACAGCAAGGTGAGAGAAA

TACATAGTCAACTAGAGATGTTCCCTTCAAACATGAAGCAATGCCCTCTGGTTACATACGCCGTCGTCTCCGAACCATAC

GTCATGCCACCTGTAAGGAAATTAACGAACACGGCTTACGACGACGCCTACGAATTCCAGAAAGGCGGCGAAATAAACCT

CGTGAAACTAATAAGCGAATTCACAAACATGAGTCTAATTGTACGGATGTCTGATTTACCGGAAAATTGGGGTCTAATTG

ACGCAAACGGGACGGCGACAGGCGCGTTCGGAGTGCTCAGGAACGACTCGGTTGATTTAGTGATCGGTGATATAGAAGTA

ACGAGAACTATCCGAAAATGGTTCCACCCGACCGTGAGTTACACGCAGGATGAAATGACGTGGTGCGTGCCCAAGTCAGC

GCAAGCGTCGACATGGAATAATTTGGTGATCATATTCCAATGGTCCACATGGGTGGCTACGATTCTCAGCATCGTCATAA

TGGGCCTTGTGTTTCACTACATTTACTATAGAGAAAATGATAGAAAAGTAACTAAATGGCCGACGAATTCTTGGCTGTAC

ACGCTGAGCATGCTACTAGGCTGGGGTGCATCGTTCAATCCCAAGAGTGCAACGTTCCGTATCTTAATCTTCGCTTGGCT

TTTCTTCGGCTTAATCATGGGCATATCTTACGAGTCGTTTCTGAGAACTTTTCTAATGCACCCGCGATACGAGAAACAGA

TAAGCAGCGAGACAGATCTCATACGATCCCGAATTCCTTTGGGAGGCAGGGGGATCTACCGATCCTATTTTGAGACTAAT

AACGAGAGTTCGGTTTATTTGTATCGCAAATACGTCCCGACAAGTTTCTCGGATGGGATTAGGCGTGCCGCTTTGAGCAG

GAATTTCGCCGTTGTGTCGTCGAGACGACAGGCGGAATACCAGGACCAGAAGTTAGGTAAAGGACAGCCGCTTTTGTACT

GTTTCAAAGAAGGGAATAACCTTTATAAGTATGGCGTTGTCCTTGTAGCTAAGCGGTGGTATCCGATGCTGGAGAGATTT

AATAATATGATAAGAAGGGTGTCTGAAAATGGTTTGATAGATAAGTGGAATCAGGAGTTGTTTATTCATAGCGTGAGTGC

CGAAAGTTCTGGCACAATAGAGTCTTTAAAAATTCAGCATTTAATGGGTGCCTTCATGTTTATAGGTTTGATGTACGCGT

TCAGCGCGCTAGTGCTCATCGGGGAGATATGCGTAAGTTTCATAGGCAAACGGAGGAATAATAAAACGGCTGTCTATCGG

GTCAAGTTAATTTAA

>Cher_IR93

ATGCGAGTATGGCTGATGTTCGTTTGTTTCGTGGGGGTGAGCGCAGAGGATTTTCCGTCGTTGATCACGGCGAATGCTTC

TATTGCGGTGGTCCTAGATCGTCAGTACCTAGGCGAGCAGTACCAGTCTCTGCTGGAGGGTTTGAAGGACTACATCAAGG

AGCTAGCCCGCGTCGACCTGAAGCACGGAGGGGTGGTCGTTTACTACTACTCGTGGAGCACCATCAGTCTCGAGAAAGGT

TTCATAGCAGTGTTCAGCATAGCGTCATGCAAAGACACCTGGGCCCTGTTCTCACGAACAGAGGAGAAGGAACTCCTGCT

ATTCGCTCTCACCGAAGTCGACTGCCCTCGTCTGCCGCCAGACTCTGCCATCACCGTCACTTACACAGATCCTGGACAGG

AGTTGCCGCAGCTGTTGCTAGATCTCAGAACAACTAAGGCTTTCAATTGGAAGTCGGCAATAATCTTGCATGATGATACT

TTAAATCGAGACATGGTGTCCAGAGTGGTGCAGTCGTTGACGTCTCAAATTGATGACGAAGATGTGCCAACTATATCCCT

GACGGTATTTAAGATGAAGCATGAGATCAACGAATACTTGAGGAGGAAAGAGATGAATAGAGTTCTGTCGAAGCTGCCAG

TTAAACATATTGGCGAAAACTTCATAGCAATAGTAACCTCAGACGTGATGTCAACAATGGCGGAGACAGCCCGCTCGCTG

GGCATGTCGCACACGCAGGCGCAGTGGCTGTACGTGGTCTCGGACACGGACAGCGGGCGCGCCAACCTGTCCGCGCTCAT

CAACGCGCTGTACGAGGGGGAGAATGTCGCGTACATCTACAATATTACGGAGGACCATGAGGGGTGTAGGAACGGACTAA

TGTGCTACGCCGAAGAGATGATGACAGCCTTCGTCTCAGCGCTGGACGCAGCCGTACAGGAGGAGTTCGACGTGGCAGCC

CAGGTCTCTGATGAGGAGTGGGAGGCTATCAGACCCTCGAAGCTGCAGCGAAGGGATATGCTGCTGAAGCATATGCTGCA

ACACATAACAGCGAACAGCAGGTGCGGCAACTGTTCGACGTGGCGAGCCATCGCGGCCGACACGTGGGGCTCCACGTACC

GCGGCTTCTCGGAGACCAGCGAAAACGAGACCACTGGGATCATTGAGAAGATTGAGCTGCTGCAAGTGGGCTACTGGCGT

CCTATTGACGGGACCAGGTTCACGGATTTCTTATTCCCGCACATCGCTCACGGGTTCCGAGGGAAAGTCTTGCCGGTTAT

CACCTATCATAATCCTCCATGGACCATCCTGAAAGCCAATGAATCTGGTTCCATCTCCAGCTACTCAGGACTTATATTCG

ACATCGTCAACCAGCTCGCCAAAAACAAGAACTTTACTATCAAACTTTTATTCCCCGGAGATATTAAAGACGCCTTGTCT

AATAAGACTTCGGCTGAAAACACGCACAGCCAAAGCGCTACGTTGACCATGATGGCGGTAGCGAAAGGTCAGGCTGCATT

TGCTGCTGCCTCTTTCACTGTGCTACCAGATAAAAACCCAGGTATAAACTACACGGTTCCTGTGAGCACGCAGCCATACG

CTTTCCTGATAGCGCGGCCGCGAGAGCTAAGCAGAGCCATGCTGTTCCTTCTTCCTTTCACCACCGATACCTGGCTCTGC

CTAGGCTTTGCCGTGGTCCTCATGGGTCCAACTCTTTACATCGTTCATCGTCTCAGTCCGTACTACGAAGCCATGGGTGT

CACAAGACAAGGGGGGCTGGCCACCATACACAACTGCTTGTGGTACATTTACGGCGCTCTGCTGCAACAAGGCGGCATGT

ACCTCCCTCGTGCAGACAGTGGTCGCCTGGTTGTCGGAACCTGGTGGCTAGTAGTTCTGGTAGTAGTAACAACCTACTCC

GGAAATCTGGTGGCTTTCCTCACGTTCCCAAAGCAGGAGATGCCCGTGACCACGGTGGCTGAACTGCTGGAGAACAGAGC

CTTGTACACCTGGTCGATTAGCAAGGGGAGCTATTTGGAGATTGAGCTGCAGAACTCCGATGAGCCCAAATACGTTTCGC

TTCTCAAAGGCGCTGAGCTTACTACGGAATCTACTGGCATGGAAGGACACTTACAAATCAGTGGATCTCCACTGCTAATG

CGCGTACGCAACGCGCGCCACGTGATCATCGACTGGAAGCTGCGACTCAGCTACCTGATGAGGGCTGAGCACCTCGCCAC

TGACACCTGCGGCTTTGCTCTCAGTGCAGAGGAGTTTTTGGATGAACAAGTAGCAATGATCGTGCCGGCTGGCAGTCCCT

ACTTGCCAGTATTTAACAAGGAAATAAACCGCATGCAGAAGGCAGGGCTCATCGCAAAGTGGTTGTCTGCTTACCTGCCG

AAGCGCGACCGGTGCTGGAAGACCTCTTCCATTGCCCAGGAGGTCGACAACCACACGGTCAACTTGAGCGACATGCAGGG

TTCTTTCTTCGTGCTGTTTCTGGGATTCGTGTCCGCAACCTCAGTGCTGCTAATCGAGTGGTTCTACAACAGGAGAAAGA

GCAAGCGAGAAGACGTCGCCATTAAGCCTTATGTCGAATGA

>Cher_IR94

AGTTTCTCTATTGAGAACAATGCCTCACAGTTTAAAGAAAAGCAAGGTCACAGAGCTGGTTTAATAATAAAAACATCCTG

TGAAAATTGGACACAGGTTTTTGATCTTTTTGATTCAAAATTATTCTATAGTTCCCTCTACTCTTGGCTGATATTTACTG

ATGATGTTTCATCTACTACAGAAACCTTATCGACATATCCAATAGAAGTTAATTCAGATGTAACCGTTATTCATAAAACA

GGGACTGTCTTTAATTTTTACGAAGTCTACAACACTGGTTTCTTTTCAAGTGGGAGTTTTCACATACAACCAGTTGGGTA

TTGGGGTTCAACATTACAGTTAAAGAAACATAGGAGAATGGACTTGAGCGGCGTCGTGTTAAAAAGTGTCGTCGTGGTCA

CGCATAGCATCGTGAACCAAACATTTGAGGATTACATGACTCGGTCTAAACTGGAAGTCGACTCACTACACAAACTTAAA

TATTTTACCCTCTTGATGTACTGGCGGGAAATGTTTAATTTTAGCTTCGTGCTGATACGCAGCAACTCCTGGGGATACCT

GCGAAACGGCAGCTTCGACGGCATGGTGGGGGCGCTGCAGCGGCGCGAGATCGAGGTGGGCGGCTCGCCGGTCTTCATCA

GGGCGGACCGCGCCAGATTCATCGACTATGTCTCGGCGACGTGGCCTGCTAAACCATGCTTCATATTCCGGCACCCGAAG

CACCCGGGCGGCTTCATCACAATCTACACGCGGCCGCTTTCCGACGAGGTGTGGTGCTGCATCCTGGTGCTGGTGCTCGC

GGCCAGCCTGCTGCTCTGCGGTCTGCTGCGGCTCAGGGTCGTGCGGACGGAGGGCGAGGTAGCAGATCTGTCCGCGAGCC

TTGCACTGCTCTCTGTTTGGAGCGCCGTGTGCCAACAGGGAATGGCGGTGAACCGGCGCGCGTCATCAGTCAAGCTGGTT

CTCTTCACCTCCTTTCTGTTCTCGCTGACGGTCTACCAGTATTACAATGCTGTGGTGGTGTCGACGCTGCTGCGCGCGCC

GCCCGTCACCATCCGAACGCTGGAAGACTTGCTGGCCAGCAAGCTGAAGGCAGGAGTTGAGGACACGCTGTATAATAAGG

ATTATTTCAGGCGCACCACAGACCCAATAGCCTTGGAGATGTACGCCAAAAAGATCGCAGCCGCACCTCCCAAGTTCTAC

CCGCCCGAGCAAGGCATGTCGCTGGTCAAGCGAGGGGGTTTCGCTTTCCACGTCGATACAGCGTTCGCTTACCCCATCTA

TAAACGCACGTTTACGGAGCGAGAGATCTGTGAACTGCAGGAGGTGAACCTATATCCGCCGCAGTATCTGTTTGCTGTTA

TGAAGAAGGATTCGCCTTATAGGAAGCATTTCAGTTACTGTATCCGCAAGATGTACGAAGCAGGGCTGATGCAGCGTCTC

AAGACCGCCTCGGACGAGAGTAAGCCGCCGTGCGTGCGCACTCCCGATTCCAGTATCTTCACCGTTTCCCTGCGAGAGTT

CTCGACGCCGATTCTAATTCTCTTCGTTGGCATGGTGATGGCCGCTTTGGTCTTTTTAGCAGAACTTGTGGTGTTTAAAG

TGCGTACTGAGGGAAGGATACAATTCACGCTTTGA

>Cher_IR95

ATGTTTTTGGTTGACTTAGCATGCCCTGGGGTGACTGAGTTTCTTCAACAGGCAAATTCCAGCGGATTATTTTCCAAACC

CTATAGATGGTTTATTGTAACCAGTTCAATCTCCGAAAGTACTGTACCTTCAGAATTGGATGATTTGCATATTCTACCTG

ACGCAGAAGTTTATATTACACAATGCAATCAAAACGACAGTTGCAGTGTAGATTTAGTGTACAAAATAAGGCCTCATCTC

GATTGGATCATAGAGAACTATGGCATCTGGACAACGAACGTCGGTCTCGTCAGATCAAGACATGTAATGGAAGTCTCAAT

ACCAATGAGACGCAAAAACCTCGCGCGTGAAACTATTACGACCTCAGTGGTAGTTAATGATAACTATACGATTGATAACT

TGGAGGATTTGAAATACACTGAAATAGACTCCATATCCAAAAGTGGATACCGCCATCTGTCGCCTCTCTACGACTTCATC

AACGCTACCAGAAAAATTATATACACTGATATTTGGGGATACTACATCAATGGGTCTTGGACCGGCATTCTTGGACATCT

ATCTCAAAGGAAAGCTGAATTACCAGGGTCAGTTATGTTCATAACCAAGGAACGGATGGCATTAGTAGAATACCTAATAT

TCCCTACCCCCACCACGGTCAGTTTCATCTTTCGGGAGCCACCTTTGTCGTATCAGAACAACCTGTATCTGCTGCCTTTT

AAGGCCACTGTGTGGTCCTGTATCGGGTCTTTTGTCATCATTATGATCTTCTTTACATACATCAATGCGTATTGGGAGGA

GAAGAAATTTGGAGATGGGGAAGACCATAATACAACGGTGTTGGTCCCGAAAGTGAGCGACGCGACTGTGTTTGTGGTGA

GCGCCATCTCTCAACAAGGAAGCACCGTGGAACTAAAAGGTACCTTGGGCCGCTTCGTCATCTTCATCCTCTTCCTCATA

TTCCTGTTCCTCTACACCGCATACTCTGCCAGCATTGTGGTGCTTCTGCAGTCCAGCTCTGACCAGATACGGACCCTCTC

CGACCTGCTGCAGTCTAAGTTGGAGCTTGGGGTTGAAGATACACCGTACAACAGATACTTGTTTGCGATGCAAAAAGAAC

CAGTAAGAAAAGCAATCTACGAAAAAAAAGTAGCGCCAGCGGGCTCCAAACCAAAATTTTATATTTTGGATGAAGGAATT

CGTCATCTACAGAAGAAACCCTTCGCCTTCCACTGCAATAAGGGCTTAGCGTACAAAATGATGGAGAAATACTTCTACGA

GCACGAGAAATGCGGCCTCCACGAAATCGCGTACCTTCAGGACATCTCCCCATGGCATGGCTGCAGAAAGGGATCTCCTT

ACATGGAGATATTTAAAATTGGTATGCTCCGGAACGTAGAAAACGGCTTGAACGACCGCACCAACCACCTCATCTTCTCC

AAGAAGCCAATCTGCTCCGCGCGAGGAGGCAGCTTCGTCTCAGTTTCCCTGGTGGACTGCTATCCAGTCTTGGTACTGCT

TCTGTATGGGATGGTACTGGGAGTACTGATATTGCTGGTGGAGGTGATCCACCATCGCCGGGTCTCTAGGAGGGGAGAAA

GGCTGGGGAAGTAG

>Cher_IR-iGluR1

ATGTTACGGCACCAGGGAATAAATTACGTGACAGTTATAACAATTCTCCTTACTGCCGGCAACAGTGAAGCTGCATTTCG

AAACTTCGAGACTCTTAAGACCCCGATAAATATTGGTGCGATATTTCCTCCGAACACAGTGACGGAGGTGGCTTTTGCTT

CGGCTCTGGCACGGGCTTCTATGGAAAGTGAACATTATGTGTTTGTAATGAAGCCTCAGTACGCACCGTACGAAGACAGC

TTTGCTGCATCTAAAGCTGCCTGTCAGCTCATTTCGCAGGGAGTTATCGCAATTTTCGGGCCCACTGATTCCACATCGGC

TTCCGCTGTCGAAGCGCATTGTAGAGCTGCCAGGATTCCTCACATACAGGCAGTATGGAGGCCACCTCCAGTCCGAGGTC

TGGAGCTACCGACTCCACCTGGTATTAATTTATATCCCGAAGCTGTGGCCCTGTCGAAGGCCGTTGCCCATTTCGTAAGA

GATAGCGATTGGCAATCATATACGTTACTTTACGATGACGATTACGGTCTAATACGTCTTCAAGAAATTTTGAAATACGC

TGACCCTGAACATAAATGGTCGGTAAGGAGATTAACCCCAGGAGAAGACAATCGACCTTTACTAAAACAATTGAAGTTAT

CCGGAGAAAGCAGGGTTATAATTGACTGTCCACCGCAGAGAGTAATGGAGTATTTGCGGCAAGCAAACGAGGTGCAGTTT

TTTGAAGATTACATGAGCTACGTCCTCATGTCACTTGATGCACACACACTAGACTTAGAAGAACTACGTTATGGTTTGTC

GAATGTAACTTGCTTGAGAATATTCGCACACGAAGATTCAAGGACACGGTCTTATTTGTCCGACTGGAAAGCAAGGGCTA

CACCCGACGTCAAGATACCTCGAGAGACACAGGACATAACGGTCGAAGTCGCATTAGCGAGCGACGCCGCAAGACTAATT

ACGGATGCAGTGGAAAGCGCTCCGGAAGAATTCAAAATTAAAGCGCAAGAGCTCGAATGTAACTCCGATAACCAGTGGGA

AATGGGCGAAGAGTTCATAAATCATTTATTAACGAACCCAATAACGGGCATTACTGACGAGATTACATTGAACAATATCA

CCGGCGAACGAATCAATTTCAATGTGGAAATAATGGAATTATCAAATAGTGGTTTCAATAGCATCGCCAAATGGAATCCC

GATACGGGTTTCGTGTACGCGCGCTCGCAGGACGAAGTTTCCGATTTGCTCGCGGAAAAATGGCAAAATAAAACTTTTCG

AGTTGTGTCGCGGATTGGAGCCCCGTACCTCATCGAAAAAAAACCTGAAGACGGCGTGGAGTTGACTGGAAATGACCGAT

ACGAAGGTTATTCAAAAGATTTGATACACGAAATCCTTACAGAAACGCTGCATTTAAATTATGTGATTGAGATCGTGCCA

GGGAATTTATACGGTTCTTACCATAAAGATACAAGGAAATGGGACGGACTCGTCGGGCATCTTCTGGAAAGGAAAGCCGA

TTTAGCCCTATGCGACCTGACGATCACCTATGAGCGGCGATCGGCTGTGGACTTCACGACGCCCTTCATGACGCTAGGAA

TAAGCATCCTGTATGCTAAGCCCGCTCCTCCTGTACCTGAACTCTTTGGCTTTCTCAAGCCCTTCTCGGTGGATGTATGG

ATTTACATGGCCGCGTCATTTCTGATGGTTTCTTTGCTGCTGCATGTTTTAGCTAGGTTCGCTCCAAATGATTGGGAAAA

CCCCCATCCCTGTGATAAGTCTCCAGAAGAGCTCGAAAATATATGGCATATCAAAAACTCGGCGTGGCTCACCATGGGTT

CTATAATGACGCAGGGATCCGATATTTTACCGAAAGGTTACTCAACGCGATGGGTGTGCGGTATGTGGTGGTTCTTCGCG

CTGATCATGTGCTCCTCTTACACGGCAAACCTTGCCGCGTTTCTCACGAACGCTGCCATGGACGACTCCATCAAGAGCGC

TGAAGATTTAGCTGGACAGACTAAAATCAAATACGGAACTGTTGAGGGAGGTTCCACTTACTCCTTTTTCAAGCGCTCAA

ACTTTACCACTTTCCAAAAAATGTGGGCCGCAATGGAATCGGCCAGACCGTCGGTGTTCGTGAAAAACAACGATGTCGGT

GTAGAGAGGGTGCTAAAGGGGAAACGGGAGTACGCGTTTTTAATGGAGTCTACGGCTATCGAATACCAGTTAGAGCGTAA

ATGTGATTTGATGCAAGTAGGCGGCCCGCTCGACTCCAAGGGATATGGTATCGCTATGCCGTTCTTGTCATCTTATCGCA

CCGCTATTGACAACGCAGTCTTGAAGTTAGCTGAAAGCGGCAGACTCCTCGAACTCAAGAATCGATGGTGGCAAGCACCA

GAAGAGGGTGCATGCGTTGCAGAAGAAGCAGGGGAGGAAGGGGCGAGTGCGGCCGAACTGGGAGTGGACAATGTTGGCGG

TGTGTTTGTGGTGCTGGGTGTGGGCTGTGGGATCGCCGCTGGCATGGGACTATTCGAGTTTTTGTGGCATGTCAAGGACG

TTGCTATTGAACAAAAGATGTCGCGAACCGAAGCGTTTTGGGCGGAGCTGATATTTGCGCTGAGTTTCTGGGAAACGGAG

AAGCCGGTCGCACACTCTAGGCCTTCGTCCACGGCCTCCGGAGATGTGTCCGGCAAAGGCTCCCGGGCCTCTTCGGTGCT

TCGCTCAGCCGCCAACCTGTTCCACTTGGAAGTATTTAAATAA

>Cher_IR-iGluR2

ATGTTACCTAAAAGCTTGTGGTACATAGTGTTAGTGATTAACTACTTTGCATTTGGAAGAGCACAAAAAGTCATAGGAGC

GATTTGTGATGACGGCGACTTTTTACTGGAAGCAGCTTTTACAGTTGCTCTGGAAGCTGTTTCTAGTGAAGAAGATCCAT

ACGAGGCTTCTGTCCAGCGGACAACCCCCGGGGACGTATTGGAGGCTCACACAGCTATGTGTACAGTTTTAGAGGAAAAT

GTTTTCGGAGTTTTCGGACCGGCAAATATAAATGCATTAAAACATGTGCAGTCCATAGCTGATTTTGTTGAAGTGCCACA

AATACTAGTCGAAACGGTGGCAGTTCAAAATCGTAATTGGTCAGCAATCAATTTGTATCCCCACCATGTTGCATACTCAC

AAGTATTCGCTAATATAATTGAAGAGAAGGGTTGGGAAGAATTAACACTGATTTATGAAGGCGCCGAACTTTTACCATTT

TTCGATAGCATACTCGCCTTACAAGATTTAGAAACAGGGAAAAAATTCTTAATGACTATCGTTCAGTTGCCAGACGGTGA

TGATTACAGATCATATTTACAAACGGTCAAAAAATCGGGATCGTTAAATTACTTAATCAACTGTAGACGAGAAACTTTGC

CAGAGTTTTTACTGCAAGCTCAACAAGTTGGAATAATGTCTGATGAGCACAGCTATATTATAATGAATCCAGACTTTCAA

ACTATTGACATAGATCCTTTCAAACATGGGGGCGCAAGCATTACAGGAGTTCGATTCTTTGACCCGAGCTTGGAATCAAT

TAAAACATTAATTGAGTCTATAAACGGAAAAGTAGCTGAACTAACTAAGGATCAGATTAAAGATGCATTAGCAGAAAACG

GACTGATACTTAATTTAGCGTTGATATATGACGCAGTAACGGTATACGTAGCCGGTATCAAGGCTATGGCTCTTAAGGAT

GGTTCCAATGTTACTTGTGAGGGGGATATCAGCTGGAATTATGGATCTAGTCTCCTTAATACTATAAAGTCGATAGAAGT

AGAAGGACTAACTGGATTTATCAAATTTGACGATGATGGGTTTCGTTCCGACTTTGAAATAGAGGTTCTTGAAGTCATGC

CTCATGGTTTTGAAAAGGTTGGCTCCTGGAATGTTGAGGATGGTTACGTGGAATCTAGAACAATTATTCCACCTGCAGAA

ATAGAAGGGACAGAGTCGATGAAGGGAAAGCATTTTATTATTCTTACAGCTTTAAGTGCCCCTTACGGAATGCTAAAGGA

GTCCTCGGTGAAACTGGAAGGAAATGATAGATACGAAGGATTTGGAATCGAGCTTATCGAAGAATTGGCGAAAATGAACG

AATTCAATTACACCTTTGACATCCAAGTCGATGGCGTGTATGGGTCATTGGACAAGAAAACCGGTAAATGGAACGGTATG

ATGGAAAAAGTTATGGATGGAAGAGCAGATTTTGGAATAACTGATTTGACAATAACATCGGCTCGCCAAAAAGCAGTCGA

TTTTACAAGTCCATTCATGAATTTAGGCATCACTATTCTATACAAAAAGCCAACTAAACAACCACCGGACCTGTTTTCGT

TCATATCACCGTTTTCTTTAGAGGTGTGGGGTTGGCTAGCAGGAGCGTACGTTGGTGTGTCACTTTTGCTGTTCTTCCTT

GGAAGGATAGCTCCCGAAGAATGGCAGAATCCATACCCATGTATAGAAGAACCAGAAACGTTGGACAATCAGTTTACAAT

GGCTAATTCATTCTGGTTTACTCTGGGGAGTGTGCTTACGCAAGGATCTGAAATAGCTCCAATAGCGGTCTCAACGAGAA

TGGCCGGCAGTATGTGGTGGTTCTTTACTCTCATTATGGTATCCTCCTACACTGCTAACTTGGCAGCGTTCCTGACAGTC

GAGTCAAAGTTCTATGCCATAAAAACAGTGCAGGACTTGGCAAGTAACCCATATGGCATCACGTACGGTTCTAAAAGAGG

AGGAGCTACCTTTGGTTTCTTCAAGGAATCTGACAATCTATTGTATCAAAAAATGTATCAATACATGGAGGACCATGAAG

AGCTTCAAACATCTTCGAATGATGAAGGATTGGCAAGAGCTAAATCTAACGATGAAAATTATGCATTCTTAATGGAATCC

ACATCAATTGAGTACATGGTAGAAAGAAATTGTGAAGTATCACAAGTAGGTGGTTTATTGGACAGCAAAGGCTATGGAAT

CGCTATGAAGAAAAACAGTCCGTATCGGCAACCTATGAGTGAATCTATACTACAGCTGCAAGAAGAAGGCAAATTGACGA

GAATAAAGGATAAATGGTGGAAGGAGAAACGCGGTGGTGGAGCGTGTGCGGACGACGATGCTGGTAGCGCGGAAGCTCAG

CCATTGGTGTTGGCGAATGTCGGTGGTGTGTTCATCGTGCTAGCTGCTGGATCCGGCATGGCTGTCATCTGCGCCTTACT

AGAGATGCTTGTCGATATATGGGTCGTTTCACACCAGGAAAAGGTATCCTTCCTAGATGAGTTGAAAGCCGAGCTAAAGT

TCATCATCAGCGGTGATGCAGAAACGAAACCGGTTCGCCATAGAGAACCCTCAGGTAGCGGCTCAGGAGGCTCCAAGAAA

TCGAAAGCCGAAGACGAAGAATCACTGAAAGACGACGAAGATCGGTTAAACCCTGCGCCCACGCCACGGTCTGAGCGATC

GGCGCAGTCCAACCATACGCGCCACACCTTGCATAGTAGGAGGCAGAGCAACGCTGTGCAAATGGCAAGAATGCGAAAGT

TCAGTAAGGCTACTTACTGA

>Cher_IR-iGluR3

ATGGCTTCAAACATGTGGAAGTGGACGTTGATTGTTTGTGTTGTTATTCAAATGTGTTCGTGTCAGATCAACATTATAAA

AGAAAAAAAGAAATCTTCATTTAAAATAATCGGGTTGTTCGAGCCAACGGCAGACGCGGAGATGCGTGCGTTCAACGAGA

GCATGCACCATGTCACAGTGGGCAACCTTCAGTTCGAGCCCGTCCTCTACACTCCGAAGCGAAGCGAAAGCTACTCTTTT

TGGCGTGAACTATGTTCAAACAATGCAGTACAGACAATTGCCGTATTTGGTCCACAAGACCCGATAGCTGATAGAGGAGT

CTTTAATCAGTGCTCAAAACTGCATATACCCTTTATACAAGCTACTTGGCAGCCTCATGATCCTGATATGGAATTTGAAG

AGCCAACTGAAGAAGAAGAGGAAAGGGAAACAAAATATCGAAACGTTATAATTAATTTCTACCCTGATGCGAATGAAATT

GCTGCCGCTTATGCGGAAGTATTGAGGTATTATAACTGGGAGAATTTTGTAGTGCTGTATGAAGACGATTACGGTTTGCG

AAGAGTTCAGAAAATCATAGCGGAATATTCCAAGAAGTTTCCCATCATAGTTCGACGTTTGGATCCTGATTCTGAGAATT

ACGAGGTATTCAAAGAAATAAGTAAGATGCAAGAGAGTCGCATTCTGTTGGATTGCCACGTTGAGAGAATACTACGATAT

TTAGGAGTTGCTGCAGGCCTCAAAATGACCAATCCCTATCAGCATTATGTTTTTATGTCCATGGACTCGAACATGGTAGT

GGACGATCTGGTAAAAAACTACACTTCCAACTTTACTTGGCTCAGCCTGGTGGACTATGAGAGCCTGAAGGACCCGCAGC

ACTACTTGACAGTGAGAGTCGGGAAGTGGGACAAGCATAACGTTATTCCACCACCACTTGTCACCAGTTTTCCGACTGAT

GCTCTGTTAATGGATGACGTGGCAAATCACGTGTTAAAGTCGATGGCAGCAGTGGACGCCCAGGGCATGCATCCCTGCTC

CACTTCCGGCAGACCTTGGAGATTCGGAGCTGAGCTCCAAGACACAATATTAAAGAAAGAATCTGCGGGAATAACAGGAA

ACATAACATTTGATCAGTATGGACGAAGAACCAATTATTCTTTGAATATAAATGAAATAAATCTGAAGCAGATGCGCCGC

ATAGCCTCGTGGGAGTCCAAAACCAGGAAAATTGTCTTCATACCGAAATTTGATGAAATTCAAAGTAATCTGACCAGTAA

CAAAAAATTCATTGTGATTTCACGCATGGCTGAACCATATTTCCAAATCAAAAAGGTATGCAACCCAGCACCTTGTGAAG

GAGATGAATATCCCAATGAACCCTACGAAGGTTTTTCTGTAGATTTGGTGAGAAATATATTTGGAGTCCTTAATGAAGAA

AAATATAATTACACCTACGAATTTCTTGCGGAAAAAGACATATCTTATGGCCAGTTCAACAACGTATCGAAGAAATGGGA

TGGCTTGATTGGGAAATTGCTGGACAAGGAAGTTGACTTGGTCGTATGTGACTTGACAATCACCGAGCGACGAAAAAAAA

TCGTGGACTTCTCAGTACCATTCATGACTCTGGGAATAAGCATTCTCTTCACAAAAGACGTCAAGGTTCCGCCTGAAATA

TTCTCTTTCCTCAAACCTTACTCCGTGGAGATGTGGATTTACACCGCGACTGCGTACTGCGTTGTGTCCATTCTACTCTT

CGTCTGCTCGAGGATATCACCTTCTGATTGGGAGAACCCGCAGCCATGTGACAAGGATCCAGAAGAACTGGAGAACATTT

GGAATTTCAAGAACTGCACCTGGTTGACCATGGGCTCCATCATGACACAAGGATGTGACATTTTACCTAAAGCATTCGGC

TCCCGCTGGGTTTGTGCGATGTGGTGGTTCTTCGCCATGATAGTGTGTCAAACCTACATCGCTCAGCTAGCCGCCTCCAT

GACGACAGCCATGGAAAACGCCCCCATTAACACCGTGGAGGACTTGGAAAAACAAACCAAGGTCTTGTACGGGGCGTATC

ACGGCGGATCTACCATGGACTTCTTTAGGAATTCAAAGGACAAAATGTTCCAAAAAATGTATGAGAACATGTTATCTAAC

CCAGGAGTATTAGTGGACAGCAACGAACTGGGCGAACAGACTGTGTTGAAGGGGAAAAACAAATACGCCTTCTTCATGGA

ATCGGTGACTATAGAGTACAAGCAGAAAAGGAATTGTAACCTGAGGAAAATAGGAGGCGAACTGGACTCAAAGGATTATG

GGATTGTGATGCCAGCTAATTCCCCGTACCGGTCAACTATCAACAGAGCAATATTAAAACTGAAGGAGAATAATAAACTG

AACGACATCAAAAACAAGTGGTGGAATGAAAAATTTGGCGCTAAACCATGTGAAGATTCAAAAGACGCTAACGACGTTGA

AGGCGATCTCGAGATGGAACATCTACGGGGTGCATTCTACGTGCTGATGGGCGGTTTGTTCATCTGTCTGTTCATCACCG

CGGCCGAGTTCATGAACGAGATACGGAACATCGTCGTTCGTGAAGAGGTTAATCACAAGGAAGTGTTCATAAAGGAGCTG

AAGGCATCGCTGAATTTCTTGCAGCTACAGAAGCCGGTGCTCCGTAACCCGAGCCGAGCGCCCTCTATCGCTGGTTCGGA

GAAATCGACCAAATCGAGCAAATCGAACAAGTCCAACAAGTCTAAAAAGCGGGCACAGTTTCTAGAGAACTTTCTAGAAA

TGGAGAAGGAGCCTCAGTAG

>Cher_IR-iGluR4

ATGATTAAAGTGTTAATATTAGCTGTGTTAATAAAATGTGTCACGTCTGTCATGGAGGAACAGTTTGTTGTTGGAGGCCT

CTTCTACGAAGGCGACGATGACTTGGAAGAGGCTCTGACCGCAAGTGCAACAATGTATAACTTCAACACGTCAGTTAAGA

AAGTGTCCAAGAAGGGCGAAGTGTTGGAAGCAGGAAAGCACGTGTGCGCTTTAGCTGCAGAGGGCGTAATAGGCATCATC

GATGGGACAAGCGGTCGAACAAGCGCACACGTGCAAGCAATATGCGACCTTCTCGACATCCCTCATGTCCTCATTGACCA

TAATGACCTTGTCAACGAGAATTGGTTGCACATCAATCTGAACCCTAGCCCTACTGCATACAACATGGTACTGGAGGAGT

TGGTAACATTGAAAGAATGGACAAACCTCACAATATTATATGAGAAGGGCTACAGTTTGCTTCGAGTGACCAGTCTGCTG

GAAATGGCTTCCAAAGAAATGACAGTCTCTGTGAGGGAACTTAGTGGAAACGATTACAGAGATGTCCTAGATGATGCAAA

AAAGAATGGCTACGTTAACTTCGTAGTAGATTGTCCATCACGCAAGCTGAAACAGTTCCTAACCCACGCCCAGCAGGTGG

GTTTGATGGCCGACGAACAATCTTACATTTTCCTTTCGCTCGATCTGTTTACGATGGACTTAACTCCGTATAGGTATGGA

GGGGTTAACATGACCGGTTTTCGACTAGTCAACATTGATGACTCCAAACTTCGCGATGACTTTCTGAAAGCAATGCCGCA

TGCAACAAACGAAACCGAGTACGACCCACAAGAAATGAATACACGACTATTTCTAATACATGATGCGATGAATATTTTTG

CACAGGCAAGCTCGAAAATAGTCAACATGGAGACTAAACCTGAAGCAGTAGATTGTAAAAATTTCACATCTTGGGAGTAT

GGAACGTCACTGCTAAGTTTTATGAAAACTACCAAAATAGAGGGAATTACGCGATCATTAAACTTTGATGACTCTGGGCA

AAGGTCCGAGGTAATCATGGAGGTTGTGGAGCTGACACCAGCCGGTCCTCAAGCGATTGGCAAATGGGAGTCTAACGCTT

TAAACATAATTCGACCGTTCGTGCCTCCCGCAGATGACGGTGATAATATTATGAGAAACAAGACATTCAAAGTTCTTGTT

TCATATAAAGCAGCACCCTTCACTTATCTTACAAAATCATCCGAACTTCTAGAAGGCAATGATCGTTATGAAGGCTACGC

TATTGATTTGTTTAACAAATTAGGTGAAATGTTGGGATTTAACTGTGAATTTGAAAATAATTTTGTATACGGAGGGTACA

ATAATGGGCAAATGACTGGAATGCTCAAGGAAGTTAATGAAGGCCGGGCGGACTTCTGCATTTGTGATTTTACCATCACA

TCTGACCGTCAAAAAGGTCTTGACTTCTCTATACCTTTCATGTCGCTTGGCATTGGAATAATCTACAAGGAACCCAGCAA

GCAACCGCCAGAAATGTTCTCTTTCATGGCCGTCTTCGCACCGGAGGTTTGGTATTACATGGTCCTCATTCAGCTCGCTT

TAGGAGTGATAATGATTGTGGTGGGCCGGCTGTCCAATAAGGAGTGGCAGAACCCGGTGCCGTGTATTGAGGACCCAGAG

GAGCTCAGCAACCAGTTCAGTTTCGCTAACTCCGTCTGGCTTATCATCGGTTCCGTCATGCAGCAAGGATCAGAAATTGC

ACCAATCGCTCTGGCTCCTCGCATGATAACCAGTATATGGTGGTTTTTCACCATGATCATGGTTGCTTCATACGTCGGGA

CATTGGTAGCCTTTCTTACAGTGGAGAAAAATGTTTTGCCATTTGAGAACGTGCAGGAATTGTACGAACACAAATCCATT

AAGTACGGTTGCAAAGAAAATGGTTCCACAAAAAACTTTTTTATGAATCAACCACCAGGTAGCATGTTCTACAAAATGTT

TGAGAAAATGAGGGACAACAATTGGAATGTCAACGAAAATGACGATGGAATTAAGAAGGCCGAGGCAGGATCTTATGCCT

TCTTCATGGAGTCGTCGACCATAGAATATTATAAGGAAAGGAGTTGCTCGTTGCTGCAAATTGGTGACCTCTTGGATTCG

AAGAGCTACGGAATCGGAATGAAAATGAACACGCCCTATAAAAAACTTATTGACGATAAACTGCTAAAACTCATGGAGAG

CGGGGAGCTGCAAAAATTGAAAGATCTCTGGTGGAAAGAAAAAAGAGCAGGAAATTGTGGGCAACAAGCAGCAGCGGAAG

AAAAACAATTGGGCATGAAGAACATGCAAGGTGTGTTTGTAGTGCTGAGTGCCGGGTGCATCGTCGGAGTGCTCATCTCC

ATACTCGATATGCTATGGGGAGTGTTCAAACGGTCCCTCAAATACAGCACAACCTTCAAATACGAGCTGGTCGAAGAGAT

GAAATTTGCCATCACATTCAGCGGAGACGTCAAGCCTGTGAAGCGTCCGGAAAAATCCGACGACAGCGCCAGCGTCGAGG

CTTTAGCTGAAGCAGATGCCGAGGCTCAAGACGAAGTCAAGTCCTTGCGTTCGATAACGTCTGTCAGGTCCAATGACAGT

CACAGGACATGTCATTCGCATGGCTCACGGCACTCACGGCACTCGCCCTCTGGCTTGAGCATAGCCTTTGCGAGACGACG

AAAGTATTCGTGA

>Cher_IR-iGluR5

GCAGTAGGAGCACGCTTGGTCCAAGGTGCTGATGAGGCTGGAGTTGAGATAGCTATCTTAGCGCGGAGACTACCACCTGC

CTTAGACGAAGAGGCCTTGCGAAACCTACTACTAGTGCTAAAGAAAGCAGAACACACACGGTTCATAGTGTGGTGTAATG

AGGCCTGTATCGAGCGGGTGCTGAGCGCGGCCCAAAGGGTTGGGTTGCTGTCTGAACCGCACTCTTATATTCTGCTGGCG

CTTGATCTGCATACCGTGGATATGGAACCTTTTAGCCATGGAGGCGCCAATATTACAGGTCTACAGCTATTTGATCCCAA

GAATGAAACGGAAATGCTAATGAATTGGCGTAAAACGTATCTAAGGCGCATGCGCGCCGCGACTGAAAGTGATGAAAACT

CAGACTCTCTAATGGACGACGCTGAGACACTAGCTGATGAGGTAGTTGCAAAACCACTAACAGCGTTGATTCTTACGTAC

GATGTGGTGCTAACTGCCAGCGAGATGCTGCTTCGGCTGGCCGTTATCGAAAACGATGAAGACGAGGCATCTTGCGAGCC

CCTCCACTCTCATGCGCATGCTGACACTCTGATCAACTATCTTCGTTCGAATGACAGTGAAGAATGGCCCGGCACGGCGG

GCCAGCGGCGCGCGGGCCGCTTACTCGTCGCGGAGCTGGCTCGCGGCGGCGACCTGACGACCGTGGGCATTTGGGACCCA

CGCTATGGCGTCATGTGGGAACGGGAGCTGATCGAGGTGCCTCCACTGCCGCCAGGCGTCATGGCCAACCAGACTTTTGT

GGTGCTCATTATGCGTAACCCGCCGTATGTGATGATGAAGTCGGCATCGGAAAGACTTTCAGGGAATGATCGATACGAAG

GGTTTTGTATCGAGCTGTTTGAGAAGCTGTCGAACATACTTAATTTCAACTATACGCTGCTGGAGCTGCCTGGCGATGCG

TATGACCAGATGCTGGACCGCATCAACAAGACAAAAGACGTGCACTTCGCGATAACCGACCTGACGATCACGGCGGACCG

CGAGCGGAAGGTGGACTTCACCACGCCCTTCATGAACCTCGGCATCAGCATACTCTACCGCAAGCCGACGGCGCCCGACC

CCAAACTGTTCGCTTTCATGTTGCCTTTCTCCACTGGAGTTTGGTTTTGTTTAGGGGTAGCATATGTCGGGACTTCACTA

GTATTGTATGTCGTTGGACGCCTATGCCCTGAGGAGTGGCAGAACCCATACCCCTGCATAGAAGAGCCTGAAGCTTTGGA

GAACCAATTCACACTAGCCAATGCGATGTGGTTCACCCTCGGAGCCATACTTTGCCAGGGATCTGAAATTGCTCCAGTTG

CGTACGGTTCTCGTGCAGTAGCGAGCGCTTGGTGGATATTCGCTCTGGTCATCACCAGTTCCTATACCGCCAACCTGGCG

ACGCTGCTTGCCAAGAAGAGCAACAATGAAGAC

>Cher_KAT

ATGAGGATAAAGTTAATTCAACAAGTTAGAAATTATGCGAGTTCACCGACAAGACGAAGAGTTGTGGTAACTGGATTAGG

AGCAGTCAGCCCTTTGGGCTCGGGAACGGAATTAGTATGGCATAACCTTTTAAAAGGTCACAGTGGAATCGTTGCACTTC

AAGGAGATGAATATTCCAAATTACCGTGCAAAGTTGCAGGGCTGATCCCAGTCCAGGAAGGAGATGACGTTGCCAAAGCA

TTGTCCAAGTCGAATTTGAAATCCATGGCTCCAGCCACTTGTTTAGCCCTCCTCGCTACCTCTCAGGCGTTAACCGATGC

CAAGTGGATCCCTGACTCAAATGAAGACAAAGAAAATACAGGGGTTGCACTAGGGATGGGAATGATAGACCTTAGAGACG

TGTGTGCCACAAATGATGCTTTAAAATTAGGATATAATAAAGTCAGTCCATTCTTTGTACCTAGAATCTTACCGAATATG

GCTGCTGGTCATATTAGCATAAAATACGGCTTCAGAGGTCCAAATCACGCTGTATCAACGGCTTGTGCGACAGGGGCACA

TTCAATTGGTGACGCTTTTCGATTTATAAGGAATGGAGACGTGGATGTGATGGTGTGTGGGGGAGCAGAGTCCTGTATTA

GTCCACTAGCCATAGCGGGGTTCTGTCGCCTGCGAGCTTTGAGTACCTCATTTAACGACAAGCCAGATCATGCATCCAGG

CCATTTGATAAAAAACGGGACGGGTTTGTAATGGGCGAAGGAGCTGCAGTTTTAGTTTTAGAGGAATATGAACATGCCCT

CAAACGGGGTGCTAAAATGTATGCTGAAATACTAGGTTATGGTTTGTCAGGAGATGCTTCACATATCACAACCCCAAGGG

AAGATGGGAGTGGGGCCGTGCTGTCAATGAGCAGAGCTCTAAAAGATGGTAACATTAGAAAAGAGGAAATAACTCACATT

AATGCTCATGCCACATCTACTCCTGTTGGAGACGGGATAGAATCAACAGCAATAAAAGCACTATTCAAAGAACACAGCTC

CAAAATATCTGTTTCATCCACCAAAGGAGCACATGGGCACTTGTTGGGTGCAGCAGGCAACTTAGAAGCAGTATTTACTA

TACTGGCCTGCCACCATGGAATATTGCCACCAATATTAAATCTAGATGATGCTGTGGATGATTTGAACTATGTTGCAAAG

GTGTCCAGAGAATGGAATGCTGAGAGAAGAATAGCATTGAAGAATTCTTTTGGCTTTGGTGGCACCAATGCTACACTTTG

TATAGGACAATTATGA

>Cher_OBP1

ATGTATTTCAAATTTTGTTTCCTTGCCTTTGTCATGATAAGCCTCAAGCATTGTTCAATGGCAGATTCTGTAGATGACTT

TAAGATGAAATACGTGAACTACGTCATGGAATGCTCAAAAGATTTCGGCATTACGACAGAAGATTTTCTTACGTTGAAAA

AGAAGGAGTTGCCCGCTGGAGAAAACGTTAAATGCTTGCTGGCTTGCGTTTATAAGAAAACTGGAATGATGGATGCCGAC

GGAAAACTTTCCCCTGAAGGAATTCAAAAGATTTCTGACGAAGTCTTCACTGACAAGCCCGAATTAAAGCAGAGGAGTGA

AGACTTTACAAACGCATGCAAATTTGTAAACGACGAAGAGTTAACCGGCGACAATAAGGACTGTGAAAGGGCAGCAAAGA

TCTTCAAATGCAGTGTCGAGAGAGGGGCTCAGTTCGACTTTACTACATGA

>Cher_OBP2

GACGATATAGCTAGTTTGAGGGCAAGGAAAGTGCCCACTGGACCCAACGCCCCATGCTTTTTGGCTTGCGTGCTGAAGAA

ATCTGGCGTCATGGACGAGAACGGTATGCTGCAAAAGGAAACCATTCTGGAGAAAGCCAAGCAAGTGTTCAATGATGCAG

AGGAACTGAAGTCTATCGAAAATTATCTGCATTCGTGCTCTCACATAAACAACGAAGCAGTCAGCGATGGTGAGAAGGGC

TGCGAGCGCGCCGTACTGTCTATCAAGTGTATGACAGAGAATGCCGCACAGTTTGGGTTCGAAATGTAA

>Cher_OBP3

ATGTCCAAAATACTTCAGTTTCTGTTATGCTGTTGTGCTATTGGAGTTGCCTTTGGCAAAACCGAGGCTGAGGTCAAAGC

CTACTTCATGAAGCTGGGCATCCAATGCAGCAGGGACTGGAACATCACTCCTGATGAGATGGTGATGATGCAGAAGCACA

AGTTGCCGGACAGCGAGAACGCACGGTGCCTGATGGCSTGTGTGTACCGGAAGGCTGAATGGATGGACGCGAAAGGCATG

TTCGACGTAGCTGCAGCGGAGGCGATGGTGGAGAAGGCTCACGGCGAGGACACTGCTATGATCGAGAACTCGAAGAAGTT

ATTCGAAGTCTGCAAGGCCGTAAATGATGAAACCGTGAAGGACGGCGAAAAGGGCTGTGACAGAGCCGCGCACATGTTCA

AATGTTTAACTGAAAATGCCACTAAGGTAAGC

>Cher_OBP4

ATGACCGACGAGGAGATAAAGATTGAGTTCACCAAGTACGTGATGAAATGCCAGAAAGACCACCCGGTAGAAGTCAGCGA

ACTGATGGCACTGCAGGCTTTCGTGACGCCAAAGAACAAGGACACCAAGTGTCTCCTGGCTTGCGCTTACAAGGCTGAGG

GATCGATGACCGCCAAGGGTTTGTATGACCTGGATCACGGATATAAAGTTGCGGAGAAGACCAAGAACGGCGACGAAAAA

CGTTTTGAGAATGCCAAGAAACTGGCTGATTTGTGCGCTAAAGTAAACGACGAGCCCGTGACAGACGGCGAAAAGGGATG

CGACAGGGCCGCACTCATGTTCAAATG

>Cher_OBP5

TCATTTCAGGCGCTATCAACGATCCAGAAAACCTTAGTCCAAGCCCAGTTCCTATCAAAAGGACTAATATGTGTCAAGAA

CAATCCACTCACATTAGAAGACATAAATACCTTCAAGATGCTGAAGATGCCCGAAGGCAACCACGCTAAGTGTTTCGCCG

CCTGCTTGTTCAAAAATATTGGCATTTTGGACGACATGGGGAAACTGACGGCATCAGGCGCTCGGCAAAGCGCGAAGCAA

GTCTTCGCAAACGATGAGAGCAGTTTGAGCAAMGTCGAGAAAATCGTCCAGGAGTGTTCCAAAGTTAACGACGAAATGGT

AGAAGATGGTGAGAAAGGTTGTGAAAGAGCTGCTCTAGCCTTTGCTTGCCTGACTCAAGTTGGACCTAAGTACGGACTTG

ATCTCCAATTCTAA

>Cher_OBP6a

ATGGCGAAATTACAATTGGTACTKCTGGTCGCAGTTGTGTGTGCTGGTCTGAAAAAAACTCATGCGGTGACAGAAGAAGA

GCACAAAGAGATGCATGAGCTGATGATGCCAATCATCGCAGAGTGCAGCGAGCAGCACGGCAAAAACCCTGATGACATCA

CTGTTGCCGAGAAATCGAAGGATGTCGACGCGATGGACGCGTGCCTGATTGACTGTGTGTTCAAGAAGCTTGGCGTTATA

AACGACGACGGTATGTTCGTCGTTGAGAAGTTTACAGAGAACATGAAGAAAATCATGAAGAAGGACGGAGACTCAGAGAA

AGTTGAAGATTTGGCGAAAAACTGCGCATCAGTAAACGACAAGGACTCCGACGACAAGTGCGGGAGGTCCAAGGCGCTGC

TCACTTGCTTGATGGAGCAGAAAGAAGTGTTGGGCTTGTGA

>Cher_OBP6b

GATGTTGATGCAATGGACGCGTGCGTGATCGACTGTGTGTTTAAGAAGCTGGGCGTTATTAACGACCACGGCATGTTTGT

CGTGGACAAATTTACTGAGAAYATGAAGAAAATCATGAAGAAAGACGGWGACTCRGAAAAAGTTGACGAGTTGGCCAARA

ATTGCGCATCAGTAAACGACAAGGACTCCGATGACAAGTGCGGAAGATCCAAGGCGCTGCTCACTTGCTTGATGGAGCAG

AAAGAAGTGTTGGGCTTGTGA

>Cher_OBP6c

ATGGCGAAATTGCAATTGGTACTGCTGGTCGCAGTTGTGTGTGCTGGTCTGAAAAAAGCTCATGCGGTGACAGAAGAAGA

GCACAAGGAGATGCATGAGCTGATGATGCCAATAATCGCAGAGTGCAGCGGCAAAAACCCCGATGACATCAGTGCTGCTG

AAAAATCTAAGGATGTTGATGCAATGGACGCGTGCGTGATCGACTGTGTGTTTAAGAAGCTGGGCGTTATTAACGACGAC

GGCATGTTTGTCGTGGACAAATTTACTGAGAACATGAAGAAAATCATGAAGAAAGACGGTGACTCAGAAAAAGTTGATGA

GTTGGCCAAAAATTGCGCATCAGTAAACGACAAGGACTCCGATGACAAGTGCGGAAGATCCAAGGCGCTGCTCACTTGC

>Cher_OBP7

ATGGTGTTATTTGTAATTGTGAAGGTTTTGTTGTTAACGGCTTTTTGTGAGGCGATGACGATGAAGCAGATAAAGAATAC

AGGGAAAATGATCAGAAAAAGTTGCCAGCCAAAAAATAACGTCGAAGATGAAAAAATAGATCCGATCAAAGATGGCGTGT

TCATCGAGGAGAAAGAAGTCATGTGCTATATGGCCTGCGTCATGAAAATGGCTAACACGGTAAAGAATGGAAAGATAAAC

TACGAAGCAGCTCTGAAGCAAGCAGACTTACTGCTGCCTGAAGAAATCAAGGAACCAGCTAAAGCAGCCTTGGCAGCTTG

CAAGAAAGTTCCGGATCAATACAAGGACATCTGCGAATCCTCCTTCCACGTAACGAAATGCATCTACAACAGCAATCCTG

AAATATTTTACTTTCCCTAA

>Cher_OBP10

GAGACGCGATGCCGGAACCCTCCGACAGCACCGCAGAAGATCGAACGAGTCATCACGCTCTGCCAAGATGAAATCAAGCT

CTCCATACTTAGAGAGGCACTGGACGTGATAAAGGAGGAGCACACGATGCCGGCGCAAAAGAGACGCAACAAGCGTGACG

TCCCGTTCACGCACGACGAGAAACGCATCGCCGGGTGCCTTCTGCAGTGCGTGTACCGGAAAGTGAAAGCTGTGGACGGC

TTTGGGTTCCCGACCCTCGAGGGCTTGGTGGGGCTGTACTCGGACGGCGTGAACGAGCGCGGCTACTTCATGGCCGTTCT

AGAGGCTTCGCGGGAGTGCCTCATGAAGAACCATGACTTGTTCTCCCGGACGGTTCCCATGGATAACGGACGCAACTGTG

ACGTGTCGTTCGACATCTTCGAGTGCATATCGGACCGCATCGGCGACTACTGCGGCAACGCCGGACTCTGA

>Cher_OBP11

ATGCGTTTCGTAGCCTTTTTTTGTTTTATTTTTGTTTTGGAGTGTACATGTGAAGTGGTGATACAGTTGGAACCTGAGAA

AACAGCAAAAGTTTTAGAAAGTGCATTCAAATGCACAAGCAAACACGGATTTAACCTTGATGTCTTGGAAAGACTAAGAA

CCAGGCATTCAACGAAAGATGAAAAGTTCCTACAATTCCTTTACTGCACGCTTGATGATCTAAAAGTTGTCAAAAAGAAT

GGTCATTTAATTGAAGAAGAAGCAGTGAAATTTGTACCAAAGCAACAAAGAGGTGTTCTAAAGAAGGCTCTGGAAGAATG

TAATAAGGAATCCGGGACAGATCGCCTTGATGTTTTATATAATATTAGCAAATGTATCCACGAGAAAAGTAATGTGCGCG

TATCAGTATAA

>Cher_OBP12

ATGAAGACCTTCATCGTATTAGCTGTCTGCCTAGTCGCGGCTCAGGCCCTCACAGAYGAGCAGAARGARAAGCTSAAGAA

RCACCGYTCRGAATGCCTCACRGAGTCCAAGCCTGACCAGCAGATGGTCGACAAACTTAAGACTGGAGACTTTAAGACRG

ACAATGAYGCSYTAAAGAAGTACGTTCTGTGCATGATGATCAAATCGGAGCTGATGACCAAGGATGGCAAGTTCAAGAAR

GACATCGCCCTKGCCAAAGTTCCTAACAGTGCTGACAAGCCCCTAGTCGAGAAGGTCATCGACAGCTGCCTCGCCAACAA

AGGCAACACCCCACACCAGACCGCGTGGAACTACGTCAAGTGCTACCACGAGAAGGACCCCAAGCATTCCATCATCATCT

AA

>Cher_OBP13

ATGAAAACAATTTTGGTGTTATTCGCTCTGGTGGCAGTAGCCTACGCTAAAAATGTCGAACTGTCACAGTCTCAAAAAGA

CAAAGCCCACCAGCATACCTTAGAATGCGTAAAACAGACCGGCGTCAAGCCAGAGGTTCTGGCGGAAGCGAAGAAAGGAA

ACTTCAAGGACGACGAAGGGTTAAAGAAGTTCACACTTTGCTTCTTCCAAAAGGCTGGCATCCTCAATCGTGATGGAAGA

TTGAACGTAGATGTGGCCTTAGCCAAGTTACCTCAGGGTGCCGACAAGGCAGGAGTAAGGAAGGCTCTAGAAGAATGCAA

GACTAAGAAAGGACGGGATGCTGCTGACACCGCATTTGAAGTCTTCAAGTGCTACCATCGCGCTACTCCTACACACGTCA

TGCTTTAA

>Cher_OBP14a

ATGTATCGTGTAATAATTTTGGCTGTTGTGCTGGCCGTGGTGGTGGCCGATTCCGAAGATGAAATCAAGAGAAAATGTCA

TAGGCTGGGACACCCTGGGACCATGCGATGCTGTAAGACCCAGATGGCGCCTCCGAAAATAGACATTTCTGAACTGAAGG

AATGCATGGAGATTCCTCACAATCCGCATTCTTGCGAGCACGACATCTGCATTGGAAAGAAACGTGGGTACGCATCAGAT

GACGGCACTGTTGACAAAGCGGCATTCGAAAAGACTTTCTCAGAGGACTTCCCTACGTCTACTGACTTGATCAAGGCCGT

GAAGGACAACTGCCTTACTGGGGATCTGGATGCCTTCGGCCCACCTGATGMATGCGARCTGATAAAGATTAAGCATTGCG

TTCACATGCAGGCTATTAATGACTGCAAGGAATGGGAGGACAATGGCCCCTGCGCCGGTATCAAGGATCTGGTGAAGGAG

TGCGCGAAGGAATTAGCTTAA

>Cher_OBP15

ATGTTCAGAGGTTGTGTTTTGTTTGCTGTGTTCCATGTAATCTTTGCTCAAGAACAAGGACCTCCACTGCCACCTAATCT

ACCACCAAACTTTCCACCAAAATGCCTCGGACCACCACCTGCTGTTGAAAAACCTCACGAATGCTGTCAAATCCCGCCGT

TCTTCCCGGACGAAGACTTCGCAGCATGCGGCTTCCAGAAACTGGACGAGGCCTCCCCGGAGAGGAAGCGAGGACCACCT

GACTGCTCAAAACAAATCTGCATGCTAAAGAAGTACAACCTCCTCGTGGACGAGGAGTCTATAGACGAGGAAGGTATCAA

GAAGTTCCTGGACGAGTGGGGCGCGAAGAACGAGGCTTTCCAGCCGGCCGTGGAGGCTGCCAAGGAGAGGTGCATTGGCA

AGAGCCTCTTTGGGCCTCCTCAGATCTGTGAGGCTAATAAGCTGGTGTTCTGCGTCAGCTCTACTCTGTTTGAGCAATGT

CCAAACTGGCAAGGAACAGAAGGGTGTTCATCATTGAAGCAACACATGGATGACTGCAAAGCCTTCTTCCCTAAATAA

>Cher_OBP16

ATGTTTAAAGTGATTGTTTTACTTGCTTTTAGTGCTGTGGTGTCACACGCGGACGTAGCAGTGACTCCACCGCCTGTCTA

TTGTGGTATTACCCCACAAAATATCTACAAATGTCTTGGCACCCCTAGAATAGTGACGAGCGGTGTTGCTTCTCAATGCA

AGTCATCGACTACCGAGTGCGAGCGCATAAAGTGTTTCTTCGAGAAGTCGGGCTGGATGAACGGAGACGCCATCGACAAG

TCGAAGGTGGTCGCGCATTTCGAGCAGTTCGCGAAGGACAACCCTGCCTGGACGCCGGCGATCAACCACGTTAAGGCGGC

TTGCCTGGCAGGGCCCGTGCCTGTCCAGGGTTGGGAGCTGAACTGCCCGGCCTACGATATCATGCACTGCGCGTTGACTG

GATTCTTCAAGAAAGCACAACCCTCGCAGTGGTCAACAGCTGAGGAGTGTACCTMCCCCCGCCAGTTCGCCGCATCGTGT

CCAATCTGCCCCGAGAACTGTTTCGCATCCGCCATCCCCACCGGCTCGTGTAACGCCTGCCGACTCCTGCCGTCGACGCC

CTAA

>Cher_OBP17a

ATGTTCAAGCTTATCGCTCTAGTCACCCTTTGCGCTGTGATGATCCAGGCGGATCCAGTGCCGTTCAAGTGCGGGCCGTT

CCCGTGGACCATCCTGAAATGTATTGGAGCCCCCAAAGTGATTAAGAAGGAGATTGCGGACCAGTGCAGCGGCGATCCTT

CCCCGTGCGAGAACAACAAATGCGTGTTCAGGAAGCTCGGATGGATGAACGATGCGCAGGTGATCGACCATACCAAGGTC

AAGGCTTACTTCGACGATGTTGCCAAGGAATACCCTGTCTGGGCTGACGCCGTGGAACAGGTCAAGGCCGCGTGCTTGGT

CGACACCCTGCCTAACCTAGGCACGCAGACCAACTGCCTGCCCTATGACATCATGGCCTGCTCCATGGCGAAGTTCATGA

AGATGGCGAAGCCCTCCCAATGGAAGACCGACGCCGAGTGCATCATCCCCCACCAGTACGTGGCCAACTGCCCCATCTGC

CCCCAGGGCTGTGTGGCCCCCTTCGCCGCGGGCACCTGTAACGCCTGCAAGGTTTAA

>Cher_OBP17b

ATGCTCAAGCTTATCGCTCTAGTCACCCTCTGCGCTGTGATGATCCAGGCGGATCCCGTGCCGATCAACTGCGGACCCTA

CCCGTGGGCGATCCTGAAATGTATTGGAGCCCCAAGAGTGATCAAGGCGGAGATTGCTGACCAGTGCAGCAGCGATCCTT

CTCCGTGCGAGAACAACATCTGCGTGTTCAAGAAGCTCGGATGGATGAACGAGGAGAAGGTGATCGACCAGAACAAGGTC

AAGGCTTACTTCGACGAAGTTGCCAAGGAATACCCTATCTGGACTGACGCCGTTGAGGCGATCAAGTCCGAGTGCTTGGT

CGACAACCTACCTAACCTGGGCACGCAGACCAACTGCCTGCCTTACGATACTACCCAGTGCGCCATGTCCAAGTTCATGA

AGCTGGCGAAGCCCTCCCAATGGAAGACCAGCGACGAGTGCGTCTTCCCTCACCGCTACGTGGCCAACTGCCCCATCTGC

CCCCAAGGCTGCATCGCCCCCTTCGCCGAGGGCACCTGTAAGGCCTGCAAGGTTTAA

>Cher_OBP18

ATGTTCCAAGCGACGCTGCCAGTGCTCAGCGCGCTGGTAGCCTTTGCTTGCGCTGGGAAGGAGACTCCAGTATTCAGTGA

TGAAATCAAAGAAATAATACAACACGTTCACAACGAGTGCGTCAGTAAGACCGGGGCTGCCGAGGAGGATATAGCGAATT

GCGAAAACGGCATATTTAAGGAGGACCCGAAATTGAAGTGCTATATGTTTTGCCTCATGGAGGAGGCGAGTCTCACCGAT

GAACACGATAACGTCGACTACGATATGATGGTGAGCCTGATCCCGGAGCAGTATACCGATCGAGTCAAAAAAATGATTTT

CGGATGTAATCATTTAGATACGCCCGATAAAGATAGATGCCAACGGGCATTCGATGTTCACAAATGCTCGTACGAAAAGG

ATCCTCACTTCTACTTCTTATTCTAA

>Cher_OBP19

ATGGCCTTTAACTTGTACACTGTGTTGGTTGTGGCTATCTTCGTCGCTTCGATAAACACTTGTTCGGCACTCACTGATGA

GAAGAAAGCTGAAATCAAGGAGAAGCTTAAAGCTCACGTCACGGAATGCGGCGGAGAGTATGGCATCACACCGGAATCCT

TGAAGGAGTTCAAGGAACAGAAGAAACAGCCTGACGACACCAACAAATGCTTTTTCGCCTGCATGTTCAAGAAGATTGGC

CTGATCGACAGCCAAGGCCTGTTCGCAGAGGACGTCGCCATCCAAAAAGTCTCGCAGTACGCCGAAGAATCAAAACTGGA

AAACGCAAAGGCAGCCGCTAAAGCTTGCACATCTGTGAACCAAGAGGCTGTCAGCGACGGTGAAGCTGGCTGTGACAGGG

CTAAGTTGCTCTTTACCTGCATAATGACACAGAAAGAACTGTACGGATTCGCTGTTTAA

>Cher_OBP20

ATGGTTTACTTTATTTTAGCAATTTTCGTGGTCGCGGCACCTCAGGTGTTCGCCGACGAAGAAGCAGGAGTAATGTCGTG

CCTAGAGATGTTCAAGCCAGAGCATATGGAAGAGGGATGCTGCGAGGACTTGAACGCGTTTGAAAAAGAGTTTGAATCCT

GCAAAACCAAGGAGGATGAGTGGGAGTGTGAAAACGCAAAGTGCGTTCTGGAAAAAGCCGGCGTACTGAGCGGTGACACC

ATCGACGAGAAGAAAGTAGAAGAAACCATGGAAAAGCACGCGAAGGAAACACCTGCCAACAAACCGATGCTCGACCGAAT

CAAGGCCAAGTGCCTGAACGGGAAATTCGAGGAGTACCCGCCTAAGGAGGCTTGCCCGCTGATCAAGTTCCAGATCTGCA

GCTACATTAACTCTGTTGTGGAATGTCAATCTTGGAAACAGAGCGACACGTGCAAAAAGATGGCGGACCACGCAAAGACC

TGCAAATCTGCGCTCGACAACGGCGAGAAGTAA

>Cher_PBAN

TTTCAGGACGAGAACTTCGAGCGAAACATTCGCAGTGGCCGCGCTGGAGTCGTCTTCAAACCCATCTTGGGGAAGCGGAC

ATTCACTGAAAACAACAGAGAAGCGGTGCTCAGAATGCTGGAGGCAGCTGACGCTCTGAAGTACTACTACGACCAACTGC

CCTACTTCGATGCTCAAGCTGACGACCCTGACATGAAAGTGACGAAAAAGGTGATATTCACCCCGAAACTGGGGCGTAGT

CTCGAGGATATGTACGAGGAGAAGCGGGCGTACGATGTGGACTTCACCCCGCGGCTGGGCCGGCGGCAGCCCGAGGCTGT

CACCTCCTCGGACGAACAAGTATATCGCCAAGACACGAACCAGATCGACGGTAGACCCAAATACTTCTCCCCGCGCCTTG

GACGCACTGTGAAGTTGACC

>Cher_PBANr

ATGGAGGAATATTTCGATGACCTCGTGATAAACACGACGAACAGCACCGTGCGGCCGGCGGGCTACGGCGAGCCCGACAC

GCTGGACCTGCTGGTGCCGCTCAGCATCACGTACGCCATCATCTTCGTGGCCGGCATCCTCGGCAACATCAGCACCTGCG

TGGTCATCGCGCGCAACCGTTCCATGCACACCGCGACCAACTTCTACCTCTTCAGTCTTGCAATCTCTGATCTCATGCTC

CTAGTCTGCGGCCTGCCCTTCGAATTCCATCGCCTCTGGAACCCGAACATGTACCCGCTCGGAGAAGCGCCGTGCATCAT

CCTCGGCCTGGCGTCCGAAACAGCTGCCAACGCCACAGTGCTAACGATAACGGCTTTTACTGTGGAAAGGTACATCGCCA

TCTGCCGCCCGTTCATGTCACATACAATGTCCAAACTGTCAAGAGCGGTTCGATTTATCGTTGCAATCTGGTTTCTAGCT

CTGTGTACGGCCGTCCCGCAAGCGATGCAATTTGGAATAGTTTCATATGTGGACAACGGGCAAAACGTGAGTGTGTGTAC

AGTGAAAGGACACGGAGTGCATCAAGTGTTCGTTATATCTAGCTTTGTGTTCTTTGTGGTGCCCATGTCAGTGATAACTG

TGCTGTATGCACTTATAGGTGTTAAGTTACGTACCTCGCGGATTTTGCACCCGGCGAAGAAGCTGTCGGTGGAGAGCAAC

GGGCGGCCGGCCGGCCCGGTGAGGTACAGAAATAGTGCTTCTCAGAGGAGGGTGATTCGAATGTTAGTCGCCGTGGCGCT

GTCGTTTTTCGTGTGCTGGGCGCCCTTCCACGTCCAGAGGCTTCTGGCGATCTATGGCAAGAGCCTGGAACACCCCACGG

ATACTTTCTACCTGGTGTACATTGTGTTGACGTTCCTGTCGGGTGTGCTGTATTTCCTGTCGACGGCCATCAACCCGTTC

CTCTACAACATAATGTCGAATAAATTTAGAAACGCCTTCAAGGTGACCCTATCCTCATGGTGCGGCAGAAAGAGCGCGCC

GCGACTCGGGCGCACATACAGCGCGCTGCTCGCGTCCCAGCGGCAACGCGCAGGAGGTGAGGACCGCGCGCGCAGCGGCC

GTCGCCTGCGCCGCCTGTCGACAGCCACCACCCAGCTGTACGACGCGCCGCCGCGAGCGCAGTGTCTCAACGGAAGGGAC

CTCTCAGTCGTGAACGAGGTGCCCAGCGGCAACGGGCACTGGGGCCGGCCGTGGCGTCTGCGGCAGGCGGAGCCCTCGGA

CTCCATAGGCTCCCCTCGCAGCATCTCCAACTCCAGCCTCCGGGAGGTCGACGAGGAACTCACTGGGGAAGAACTAGCCA

CCTACATGTACCAAGTCAACTGTAACATAGGAGGCCTCACCTGA

>Cher_PBP1a

ATGAAGACGTTCTTGAAGCAGAAAAAGGTGGTTTGGTTCGTGGTGGTATGCCTGAGTTTAAACCAGGTGGTACAAACCTC

GAAGCAAGTACTTGAAGGCATGACATTGAACTTTGGTAAGGGGCTGGACGAATGCAGGACGGAGATGAACCTCCCTGAAT

CTGTCACTGCAGACTTCATCAATTTTTGGAAGGACGACCACGTACTGTCTAATAGGGACACAGGCTGTGCCATAATGTGC

CTGGCCAAAAAACTGGAACTGGTCTCCGACGGCAAACTGCATCACGGGAATACCCTAGAATTCGCTAAACAACATGGAGC

TGATGCGACTACTGCCCAGGAAATAGTGGACCTGATCCACACCTGCGAGAAGTCACTACCAGMTCTTGACGATCCTTGCA

TGATAGTGCTGGAATGGGCCAAGTGCTACAAAGCGGAGATCCACAAGCRGAACTGGGTACCTTCCATGGATGTAATGGCG

GGAGAATTGTTAGCTGAAGTA

>Cher_PBP1b

ATGGTGAAGCAGAAAGAGGTGGTTTTGTTCGTGGCGGTTTGCCTGAGTTTAAGCCAGGTGGTGGAACCCTCGAAGCAAGT

GGTTGAAGGCATGACACTGAACTTCAGTAAGGGCCTGGACGAATGCAGGAAGGAGATGAACCTCCCCGACTCTGTCACCG

CAGACTTCATCGACTTCTGGAAGGACGATCATGTCCTTACCAACAGGGACACAGGCTGTGCCATAATGTGCCTGGCCAAG

AAGCTGGAACTGGTCTCCGAAGGCCAATTACATCACGGGAATGCCCTAGACTTCGCGAAACAACATGGAGCTGATGCGAC

AGTAGCCCAGGAAATAGTGGATTTGATCCACAATTGCGAGAAGACGCTGCCAGCCCTCGGCCTCGACGACCCCTGCATGA

TCGTGCTGGAGTGGGCCAAGTGCTACAAAATGGAGATCCACAAGCGGAGCTGGGCACCTTCAATGGATGTGCTAGCTGGA

GAATTGTTGTCTGAAGTATAG

>Cher_PBP1c

ATGTTGAAGAAGAAAAAGGTGGCTTTGTTCGTGGCAGTATGCCTGAGTTTGAACCAGATAGTGGAACCCTCGAAACAAGT

AGTTCAAGGCATGACATTCAACTTCGCTAAGGGGCTGGACGAATGCAAGAAGGAGATGAACCTCCCCGACTCTGTCACCG

CAGACTTCATCGATTTCTGGGAGGTCGACTTCGTGCTGACCAGCAGGGACACAGGCTGTGCCATAATGTGCCTGTCCAAG

AAGCTGGAACTGGTCTCGGACGGCAAACTGCATCACGGGAACACCCTGGAATTCGCTAAGCAACATGGTGCTGATGAGAC

AGTAGCCCAGCAAATGGTGGACATGATCCACACCTGCGAGAAGGCACTGCCAGACATGGACGACCCCTGCCTGAGAGTGC

TGGAGTGGGCCAAGTGTTTCAAGGTGGAGATCCACAAGCGGAACTGGGCACCTTCCATGGCTCTAGAGGCGGAAGAATTT

TTAGCTGAAGTTTAG

>Cher_PBP2a

ATGGCCGTGATTTCAAAATGGCGGATGCTGGCATTGATGCTCTGCTTGACTCTGGGAGTGCATAGGGTGGAGTCATCAGC

TGATGTTATGAAGAAACTAACCGCTGGATTTGCTACCGTCTTGGAAAAGTGTAAGAATGAGCTGAACATCCCCGACGCAG

TGATGCAGGACTTCTACAACTTCTGGCGCGAGGACTACGAGCTGGTGAACCGGGACATGGGCTGCGCCATCATGTGCATG

GCCAGCAAGTTCGACCTGGTCACCGACGACCAGAAACTGCACCACGGGAATGCGCACGAGTTCGCCAAGAGCCACGGTGC

TGACGACGAAATGGCTAAACAGCTGGTGTCGATGCTCCACGAGTGCGAAAAGCAATATGCCAGCATCAGCGACGACTGCA

GCAAGACCCTGGAAATTGCCAAGTGCTTCCGGACTAAGATCCATGGGCTCAAGTGGGCGCCCAGCATGGAGACTATCCTG

GAAGAGGTCATGACTGAGGTCTAA

>Cher_PBP2b

ATGGCCGCGATTACAAAATGGCGGATGCTTGCTTTGCTCTGCTTGGCTCTCGGAGTGTGTAGAGTAGAGTCATCAGCTGA

CATCATGAAGAAACTCACCGCTGAATTTGCTACTGTCTTGGAAAAGTGTAAGAATGAGCTGAACATCCCAGACGCAGTGA

TGCAGGACTTTTATAACTTCTGGCGCGAGGACTACGAGCTGGTGAACCGGGAGATGGGCTGCGCGCTCATGTGCATGGCC

AGCAAGTTCGACCTGGTCACCGACGAACTGAAGCTGCACCACGGGAATGCGCACGAGTTCGCCAAAAGCCATGGAGCTGA

CGACGACATGGCAAAACAGCTGGTGACGATCCTCCACGACTGCGAGAAGCAGGCTGCCGGCATCAGCGACGACTGCAGCA

AGACGTTGGAGATCGCCAAGTGCTTCCGCACCAAGATCCATGGGCTCAAGTGGGCGCCCAGCATGGAGACTATCCTGGAA

GAGGTTATGAGCGAGGTCTAG

>Cher_PBP3

GCACGTTTGTCCATCTTTGTCGCTCTTGTGGTATTGGCGATGGGCGTGAGTGAGATAGACTCGTCGGCAGAAACCATGGA

GGATCTGACTGCGGGCTTCCTTAAAGTGCTAGAGGAATGCAAGAAAGAGCTCAACCTCCCCGATAACATCATCAATGACT

TCTACCACTACTGGAAGGAGGACTACTCCCTGCTCAATCGAGACACCGGCTGTGCCATCATCTGTATGAGTAAGAAGCTT

GACCTCATCGACGCCAATGGCAAGATCCACCATGGGAACGCTGAGGAGTTTGCTGTGAAGCATGGGGCTGCCACTGACGT

AGCTGCCAAGCTGGTGGGCATCCTCCACGAATGCGAAAAGACTCACGAAGCCATTGAAGACGACTGCATGATGGCCCTTG

AGGTCGCCAAGTGTTTCCGGACTAACATTCATCAGCTCAACTGGGCGCCTAAAATGGACGTCGTTATTACTGAAGTCCTC

ACTGAAGTA

>Cher_SNMP1

ATGCAGCTCCCGAAGCATCTAAAAATAGCGGCGGGCTCCGCCGGGGCAGCCATCTTCGGCGTACTGTTCGGATGGGTCAT

TTTCCCCGCTATCCTCAAGGGACAGCTGAAAAAGGAAATGGCACTCACGGCGAAGACGGACGTACGTGAGATGTGGCAGA

AAATCCCTTTTCCTCTGGACTTCAAGGTGTACCTTTTTAACTATACCAATGCGGCTGAGGTGCAGAAAGGCGCCATTCCT

ATTGTCAAAGAAATTGGACCTTACTACTTTGAAGAATGGAAGGAGAAAGTAGAGGTAGAAGAGAATGACGAAGAAGACAC

GATTAATTACAAGAAATTAGACGTGTTCCTGTTCAAACCGGAACTGTCGGGACCTGGCTTGACAGGCGAGGAGACCATCA

CCATGCCACATCCGTTTATGTTGGGCATGGCAACAGTCATCCACCGCGACAAGCCATCAATGCTCAACATGATCGGCAAA

GCTATAGCTGGTATCTTCGACGCCCCAGAAGACGTCTTCATCAGGGTCAAGGCTCTAGATCTTATGTTTAGAGGTGTAAT

CATAAACTGTGCCAGGGCTGAGTTTGCTCCGAAAGCCGTTTGCACGGCGCTGAAGAAAGAAGCTGTTAATGGCTTAGCAA

TTGAGCCCAACAATCAGTTTAGGTTCTCTTTGTTTGGTATGCGTAACGGCACAGTGGACTCTCACGTGGTAACTGTTAAG

CGTGGCAAGAACAATGTGATGGACGTCGGGCAAGTCATCGCCATTGACGGGAAACCGCAGCAAGAGATCTGGAAGGACCA

GTGCAACGAGTACGGTGGCACCGACGGGACAGTATTTCCGCCTTTCCTGACTGAGAAAGATAGGCTGCAGTCGTTTTCTG

GAGACTTGTGCAGATCTTTCAAGCCCTGGTACCAAAAGAAGACATCCTACCGAGGCATAAAGACCAATCGTTATGTCGCC

AATATTGGGGACTTTGCCAACGATCCTGAGCTCCAATGCTTCTGCGACACCCCGGCCACATGCCCTCCGAAGGGCCTCAT

GGACTTGAATAAGTGTCTGGGAGCACCTATGTATGTGTCCCTGCCACATTACTTGGAGAGCGATCCAACTTTGCTGGATA

ATGTCAAGGGCCTGACTCCAGATGCCAGTGTACATGGAATCGAAATTGACTTTGAGCCGATCAGCGGCACTCCCATGGAA

GCTCGTCAAAGGGTGCAGTTCAGTATGAAGCTGTTGAAAACTGACAAGTTGGAGCTGTTCAAAGACTTGCCTGGCACTAT

GGCTCCTTTATTCTGGATTGAAGAGGGTTTGGCCCTCAACAAAACCTTTGTCAACATGCTGAAGCATCAGCTCTTCTGGC

CGAAGCGGGCCGTCGGCGTGCTCAAGTGGCTGCTGGTCTCCTTTGGGATCCTCGGAGCCCTGGGCGGTGCCGTGTTTCAC

TTCAAAGGGAATATAATGAGCTTCGCCGTCTCCAATAACTCAGCATCTACCACCAAAATCAACCCTGAAGAAGACCAGAA

GGACATCAGCGTCATCGGCCAGGTCCAGGATCCTGCCAAGATCTCCATCTAA

>Cher_SNMP2

ATGCTGAAGAAGAATTTCGTCTTCGACCAAAAAGAGTCGGGCCAGCTCACTGAGGATGACGAAGTTNAAGAGTTGATGCC

AGGTTTAGTTGGAGTGATCAACACTGCACTGGGAGACCTGTTTGGCAACCTTACAGACCCATTCATGAGGGTCAAAGTTA

AGGATCTATTCTTTGACGGAGTATACCTAAACTGTGTCAGCGAGAACTCGGCTTTGGCTCTTGTTTGTGGAAAAATGAAA

GCTGATAAGCCCCCCACTATGAGGCTTGCTGACGATGGAAATGGTTTTTACTTCTCTATGTTTTCTCATTTAAACCGAAC

AGAAACAGGCCCCTACAAGATGGTCCGCGGAACCCAGAACATCCAAGAACTCGGCCACATCGTCTCCTACAAGGGTCAGA

CCACGATGAAGAACTGGAACGACAAGTACTGCGGCNCCATATTCCCTCCTGTGGGGGACAAGCCTCTGGAGAGACTGTAC

ACCTTCGAACCGGATGTATGCAGGTCCTTGTACGCGAGTCTAGTCGGCAAATCCAGCATTTTCAACNACGGCTGCCTCCT

AATGGGCGTGCTCAACCTGGAGCCCTGTCAGGGTGCTCCGGCCATCGCGTCGCTTCCTCACTTCTACCTCGGCTCCGAAG

AATTGCTGGGGTACTTCGGCGCTGGCATCAGCCCCAATAAGGAGAAGCACAACACTTTCGTGTACTTGGATCCGGTCACC

GGAGTCGTGCTGAAAGGAGTCCGACGTCTGCAGTTCAACATTGAGCTGCGGCAGATGGATGCAGCCCCGCAGTTGGCAAA

AGTGCGCACTGGCGTGTTCCCTTTGCTGTGGATTGACGAGGGTGCAGAACTGCCCCAAGACATCCAAGAAGAGCTGCTCC

AATCCCACAAACTCCTCGGTTACGTGGAGCTGTCCCGCTGGATCCTACTCTCCCTGGCTATACTGGCGACCCTGGTGGGC

GCATTCCTCGTCGCCCGCTCTGGAGCCCTGCCTATGTTCGCT

>Cher_SNMP3

AACATACAGGAGCGGCCGGACTGCTCGCTGGCCTTCTCTCTGTTTGGATATAGAAATGGAATTCCTTCACCCAACTATAA

AATACATAGGGGGATCAACAATGCGAGAGACCTGGCCCAAATTATCAGCTTCGACAACAGCTTGCGCCTGCGCAAGTGGC

CCGAGGGCGACGAGGGCTCCATCGCGGAAGACGAGCCCAACATGTGCAACAGCTTGAACGGCACCGACGCGGGAGTGTAC

CCCCCTTTCGTGGACAAGGACAGATCGGTCTACGCGTTCAGGAGCGATATTTGTAGGTCCGTCGAGCTCCGGCACCAGTA

CGACCACGAGTTGTTCGGCGTGCCGGTGTCGCGCTTCGCGGCCAACGAGTGGTACCTGGACAACCACCAGGGCTGCTTCT

GCCTGAACCTGACGCGCGGCCTGCGCGCCCCCGACGGCTGCCTGCTGCGCGGAGCTATGGAACTGTATTCTTGCGTCGGA

GCTTTCTTGGTGCTGTCCAATCCACATTTCTTGTACGCTGACCCAGGCTATTTAAATGGCGTTATCGGCATGGCACCAGA

CGTTGAAAAGCACAGAATAGCTTTGGATTTAGAACCTAACACTGGCATTGTATTAAGGGGAGGAGCGAAGGCGCAATTTA

ATATTTTCTTGCGTCCAATATTGCGGATAAGCGCCACAGCTAACCTGACGAACATCCTAACGCCTATTTTTTGGATTGAA

GAGGGTATAGAGATGCCGGAAATATATCAAGACGAGCTGAAGGAAAAATTGCTAGCACCGTTGAGGCTAGTCGACATTCT

AGTCCCAATCTTGGTTGCTCTCAGCTGTGTGGTGCTAGTATTAGGTATTGGCCTAACTGTCCGAGCAAGATTAAACAGAC

CGGTAGAAAATAAGACTTGA

>Cobl_ABPX1a

ATGTCAGGACACGCAGCACTCTGCTGTGCCGTGGTGGCAGCATTTGTGCTGGGAGCCCACTGCATGGACGAGGAGATGGC

CGAGCTGGCCAAAATGTTGCACGACAATTGTGGAGAAGAAACCGGAGCTGACCTGAGCTTGGTGGATAAAGTAAACGCTG

GAGCAGACTTGATGCCCGATGCCAAACTGAAATGCTACATCAAGTGCATCATGGAAACTGCGGGCATGTTATCAGACGGA

GAAGTTGATGTGGAGGCAGTGATAGCTCTCTTGCCCGAGGACATGGCCAAGAAAAACGGAGACAACCTGCGAGCATGCGG

CACCCAGAAAGGAGCAGACGACTGTGACACCGCATTCCTCACCCAGGTATGCTGGCAGAAGGCAAACAAGGCAGAATACT

TTTTAATTTAG

>Cobl_ABPX1b

ATGTCAGGACACACAGCACTCTGCTGTGCCATGGTAGCAGCATTTGTGCTGGGAGCCCACTGCATGGACGATGAGATGGC

CGAGCTGGCCAAAATGTTGCACGACAACTGTGGAGAGGAAACCGGGGCCGACCTGAGCTTGGTGGATAAAATTAATGCCG

GAGCAGACTTGATGCCCGACCCCAAACTGAAATGCTACATCAAGTGCATCATGGAAACCGCAGGCATGTTGTCGGAGGGA

GAAGTTGATGTGGAGGCAGTCATAGCTCTCTTACCTGAGGACATGGCCAAGAAAAACGGAGGTGCCATACGAGCATGCGG

CACCCAGAAAGGAGCAGACGACTGTGACACCGCATTCCTCACCCAGGTATGCTGGCAGAAGGCAAACAAGGCAGAATACT

TCTTAATTTAG

>Cobl_ABPX2a

ATGTCTAAATTCACCGAGTGCGCGTTTGCCACAGCAGTTCTGCTGTCTCTGGTACTTTCGCTGGCAGAGTCTGCGAGTAC

CAAGGAATCAGAAAACGCTAAGGATGATGTCAAAGCTAACATCGAAGACACGACTCCTGCGCCGGCTGATCGGGACGAAA

TGGACATCATGAGTGCTATGATGGACTGCAACGAAACCTTCAGGATAGAGATGTCATATTTGCTGGCGTTAAATGAAAGT

GGCAGCTTCCCTGACGAAACCGACAGGACACCAAAGTGCTACATCCGCTGCGTCCTAGAGATGGTGGAGATAGCGTCTGC

CGACGGGCAGTTCGACGCCGCGCGCGCCGAGCCGGCGCTGAGCAGCATCCGCGGCGTGCGCCTGCTCAGCGACGTCGCCG

CGACCGCCGCCACGTGCGCTGCTGACCGACAGGAGTCATGCAAATGCGAGAGATCTTACCAATTCATCAAGTGCCTCATG

GAAATGGAAATCAAGATGGCTGAGAAACCTTAA

>Cobl_ABPX2b

ATGTCTAAATTCACCGAGTACGCGTTTGCCACAGCAGTTCTGCTGTCTCTGGTACTTTCGCTGGCAGAGTCTGCGAGTAC

CAAGGAATCAGAAAACGCTAAGGATGATGTTAAAGCTAACATCGAAGACACGACTCCTGCTCCGGCTGATCGGGACGAAA

TGGACATCATGAGTGCTATGATGGACTGCAACGAGACCTTCAGGATTGAAATGTCATATTTGCTGGCTCTGAATGAAAGC

GGCAGCTTCCCTGACGAAACTGACAGAACACCAAAGTGCTACATTCGCTGCGTACTGGAGATGGTAGAGATAGCGTCGGC

CGACGGGCAGTTCGACGCCGCGCGCGCCGAGCCGGCGCTGAGCAGCATCCGCGGCGTGCGACTGCTCAGCGACGTCGCCG

CGACCGCCGCCACGTGCGCTGCTGATCGACAGGAAGCATGCAAATGCGAGAGATCTTACCAATTCATCAAGTGCCTAATG

GAAATGGAAATCAAGATGGCTGAGAAATCTTAA

>Cobl_ABPX2c

ATGTCTAAATACACCGAGTACGCGTTTGCCACCGCAATTGTGGTGTGCCTGATATTTTCGCTGGCAGAATCTGCGAGTAC

CAAGGAGTCAGAAAACGATAAGGATGATGTCAAAGCTAAGAGCGAAGTCACGTCGCCTGCTCCGGCTGATCGGGACGATA

TGGATATCATGAGTGCCATGATGAACTGCAATGAAACCTTCAGGATAGAAATGTCATATTTGCTGGCGCTGAATGAAAGC

GGTAGTTTCCCTGACGAAACTGACAGAACACCAAAGTGCTACATCCGCTGCGTGCTCGAGATGGTG

>Cobl_ABPX3a

ATGGGACGAAAGGACTGGCTCTTTCTGGTGGTGGTTGTGGTATCAGTTTGCGGGATAAATGCTTTGACAAGAGTACAGTT

GAAAAAGTCGTTGCAAATGTTAAAAAAGAAATGTTTGACAAAGATAGATGTCACAGAAGATATGGTCGTAGATATAGACA

AAGGAAAGTTTATCGAAGAAACTAATTTCATGTGCTACATCGCATGTGTTTACCAGATGGGACAAGTTGTAAAAAATAAC

AAATTGAGTTACGAGGCATCTATGAAGCAGGTCGACTTGATGTACCCTGTAGAGATGAGGGAGGCTGTGAAGAAAGCCAT

AGATAGCTGTAAAGACGTTTCCAAGAAGTACAAAGACCTGTGCGAGGCCTCATACTGGACCGCAAAATGTATTTACGAAG

CCGATCCG

>Cobl_ABPX3b

ATGGGGCGAAAGGACTGGGTTTTTCTGGTGGTGGTAGTGGTTTCAGTCTGTGGGATTAATGCTCTGACCAGACAACAATT

GAAAAAGTCTTCGCAAATGTTCAAAAAGAAATGTATGGCAAAGATAGATGTCACAGAAGATATGGTCGGAGATATAGACA

AAGGAAAGTTTATCGAAGAAAAGAATGTAATGTGTTACATCGCATGTGTGTATCAGATGGCACAAGTTATAAAAAATAAC

AAATTGAGCTATGAAGCATCTATGAAACAGGTCGACTTGATGTACCCTGCAGAGATGAAGGAGCCTGTGAAAAAAGCAAT

AGAGAACTGTAAAGACGTATCCAAGAAATACAAAGACCTGTGCGAGGCTTCATACTGGACCGCAAAATGTATTTACGAAG

ACGATCCGAAGAACTTTGTATTC

>Cobl_ABPX4a

ATGTATTCCACTCAGGTTAATTTTTTCGTTGTTTTAATCTTATATTTTGTGTGGAATACCACCGCGATGACTAGACAGCA

GATGAAAAATTCAGGAAAACTGTTAAAGAAAACTTGCATGCCAAAGCATGATGTCACAGAAGAACAAGTAGGGAAGATAG

ACCAAGGTGTATTCTTGGAAGAGAAGAATGTGATGTGCTACATCTCTTGCATTTATTCGGCCGGAGGGGCGGTGAAAAAC

AATAAAATTGTGCACGAGGCTATGTTAAAACAAGTGGACATGATGTTTCCCCCTGATATGAAGGACCCTGTCAAAGCGGC

TATGGAGAACTGCAAAAGCGTCGCCAAAAAATATAAAGATATCTGCGAAGCTTGCTTTTACACTGCTAAGTGTCTCTACG

ACACTGACCCAGCCAATTTTATGTTCCCTTGA

>Cobl_ABPX5

ATGGCAACATTCAGGATGAATGCTTTATTTGGTTTAGTGTTGGTCACAGTATGTTGCCATACAGCTTATGGGATGACCCG

AGCGCAACTTAAGAAGACAATGACAGTCATGAAGAAGCAGTGTATACCGAAAGTAGGAGTGGCTGAAGACAAAGTGGCTA

GAATTGAAGAAGGCGTGTTCCCAGAAGACCGTAACGTCATGTGCTACGTGGCCTGTATCTATAAAACTATCCAAGTGGTT

AAGAATAACAGGCTGAACAAAGAAATGATATCGAAACAGATCGACATTATGTACCCAGCAGAGATGAAGGCTGCCGTCAA

GAAATCTGTTGCGAAGTGCGTCGACGTTCAGGATAACTACGAAGACGAGTGCGAACGCGTGTACTACGCTACCAAATGTT

TATACGAAGACGACCCACCGAATTTCATATTTCCATAA

>Cobl_ABPX6

CGATCATTTGAGTTGTGTTTTTTCATCGTCTTTATCTTAAGTTTTTTGCTCCTCAATGTTGCTCATGCGATGACGAGACA

ACAACTCAAAAATTCCGGAAAGATGATGAAAAAATCGTGCATGCCAAAAAATGACGTGACTGAAGAGCAAGTTGGTGAAA

TAGAGCAAGGGAAATTTATAGAAGAAAAAAATGTTATGTGCTACATAGCTTGTGTATACTCCATGACACAGGTGGTGAAA

AACAATAAGCTGAGCTACGAAGCCGTCGTCAAACAAGTGGACATGATGTTTCCTGCAGAGATGAGAGATGCTGTGAAGGC

TGCGGCTGAGAAATGCAAGGATGTCTCTAAGAAGTACAAAGACATATGTGAGGCGTCATACTGGACTGCAAAGTGCATGT

ATGACTCTGATCCGAAGAATTTCGTTTTCCCTTAA

>Cobl_ACAT1

ATGGCCGTAGCAATAAACAAAGGTATATTCATAGTCGCAGCCAAGCGCACGCCATTCGGCCGCTATGGGGGCAAGCTGCG

TGATGTGGCACCGTCAGACCTGCTGGCTACCGCCGCCAAGGACGCCTTCAAGGCCGGCGGAGTTGCGCCAGCGCTGGTCG

ACACTGTCAATATTGGACAAGTCAACGGGTTGAGCGGCTCCTCAGACGGCGGTCTAGCTCCGCGCCATGCGTCCCTCAAG

TCCGGAGTACCGCAGGAGAAGCCGGCCCTCGGCGTCAACCGGCTCTGCGGCTCTGGCTTCCAGGCCATCATCAACAGTGC

CCAGGACATCATAACAGGAGCCGCGAAAGTATCCCTAGCAGGAGGAACCGAAAACATGTCAGGCATGCCTTTCGTGGTGC

GCAACGTTCGCTTCGGAACCGCCCTTGGACAGAACATCGAGTTCGAAGACGCTCTGAACAAGGGTTCTCTGGACACGTAC

TGCAACTTCACGATGCCTCAGACCGCTGAGAACCTGGCTGAGAGGTACAAGCTGCAGAGGAGTGAAGTAGATGCTTTCGC

GCTGCAGTCGCAGCAGAGGTGGAAGGCTGCCTACGACGGCGGTTTCTTCAACGAGGAGATGTCTCCCGTCACAGTAAAGG

TCAAGAAGCAGGATGTGGTGGTCTCGATGGACGAGCACCCGCGCCCGGAGACCACGGCCGAGGGCTTGGCCAAGCTGCCC

GTTCTGTTCCGGAAGGGCGGAGTTGTTACTGCTGGGAACTCTTCTGGAGTAAACGACGGAGCCGGCGCTGTGATCCTAGC

CAGCGAGGAGGCGACGAGCCAGAACGGCCTCAAGCCCATTGCCCGCCTGCTGGGATGGTCCTTCGTGGGCGTGGACCCCA

GCGTCATGGGCATTGGTCCCGTGCCCGCTATCCAGAACCTGCTCGCTGTCAACAAGCTCAGTCTTAAGGATATTGATCTC

ATTGAGATCAACGAGGCTTTCGCAGCGCAGACCCTAGCGTGCGTGAAGGAGCTCGGCATTGACCAAAGCATCCTGAACGT

GAATGGAGGCGCCGTCGCACTCGGGCACCCTGTTGGGGCTTCAGGGGCTCGCATCACCGCGCACCTGGCCTATGAACTCA

GACGCCGTGGGCTGAAGAGGGGTATTGGATCAGCTTGCATCGGAGGAGGACAGGGCATCGCTCTGCTCATTGAGACTGTT

TAA

>Cobl_ACAT2

CTAGTTTTCTTTCACTGTGCGACGACAGCGCCGTTGGTGAAGGCAAATTGTGATTTAAAATTCAACATGGCGCTGAAGTG

CAAAGGTGTATTTATTGTTGGCGCGAAAAGGACACCATTTTGTGGCTATGGCGGGCGCCTGCGGGAGTTGCCAGCCTGTC

ACGTGTTCGCCGCTGCGGCAAAGGACGCGATCCGTTCAGCCAACGTCGATCCCAGTACCATCGATAATACTGTTGTTGGA

AATGTTAATTTTCTSAGYCAATGCGATGGYGGAAAGACCGCAAGGTACTGCGGMATTTATTCSGAGGTTCCTWTAGACAG

GCCGGCTTTGGGCGTGAGCAAAGCCTGTGGAACTGGCATTCAGGCCATCATCACGAGTGCTGTGGATATTTTAACRGGTG

CAGCAAAAATATCCCTAACAGGAGGTACAGAGAACATGTCAGCAATGCCACTTGTAGTTCGGAACGYYCGCTTCGGRACT

GYYCTAGGSGGSTCATATCACGTYGAAGACTACATCACGAARCAATACCTAGAYTCWTATAGCGGCCTRACRCTGCAACA

GATTGCAGAGGARGTTGCGAARAAGTGTGGAATCACAAGGGAAGAGGTCGATGAATTTGCAYTGCAAAGCCATTTGAART

GGAAGGCAGGGTACGAAAGCAACGTYTTTGACGATGAAATAGCMARYATCACAGTGGAAWTAAAGAAAAAGAAACTSACM

GTKATRAAGGATGAACTTCCTCGMACTTTCATCGCTGCTGAGCATYTGAGYGCYCTWSCWSCYGTRGCTGGACTTTCCAT

AGTAACTGAGGGGAAT

>Cobl_ACAT3

ATGGCTTCCCACGTAGCGAAAACCTTGTTAAAAGTTCCCCATGCCAGCTCGACGGTCAAGTTTGACACGGCTAGACGAGC

CCTGAGCATCGGGGCAGCGCTACAGTCGAAAAAGACCCTGCAGGACCGCACGGGCAAGAACATTGTGCTCGTGGACGGAG

TCCGCACTCCGTTCCTCGTCTCCTTCACCGACTACTCCAAGATGATGCCCCATGAGCTGGCGAGACACGCGCTGCTGGGT

CTCCTCCAAAAGACAGGCATCGACAAGGACCTGATCGACTACATCATCTACGGCACGGTCATCCAGGAGGTGAAGACGTC

CAACATCGGCCGGGAGGCTGCGCTGGCCGCTGGCTTCAGCGACCGCACACCGTCCCATACTGTCACCATGGCTTGCATCT

CTTCTAACCAGGCTATTACTACTGGCATCGGCATGATAGCAGCCGGCGCATACGACATAATAGTGGCCGGCGGCGTCGAG

TTCATGTCGGACGTCCCAATCCGTCACTCGCGCAAGATGCGCTCGCTGCTGCTGCGCGTGAACAGAGCCAAGACGCCCGC

GCAACGTCTGTCGCTGCTCGCGTCTATACGGCCGGATTTCTTCGCGCCAGAGCTGCCAGCAGTAGCAGAGTTCTCCTCGG

GAGAAACTATGGGCCACAGCGCAGACCGACTCGCAGCTGCATTTGGTGCCTCGCGCCAAGAACAAGATGACTATTCGCTG

CGCTCGCACACGTGCGCCGCCCAGGCTCAGGCCAAGGGGTACTTTACCGACCTCATTCCTGTCAAGGTGGACGGCAAGGA

AGCTCTGGTGGAGAAGGACAACGGCATCCGCGTGTCCACGCCCGAGCAGCTCGCCAAGCTGCGGCCCGCGTTCGTCAAGC

CGCACGGCACCGTCACCGCCGCTAACGCTTCCTTCCTGACGGACGGCGCGTCTGCGTGCCTGGTGATGCCGGAAGCGAAG

GCCAAGGAGCTGGGCTTCAAGCCCAAAGCCTACCTGAGGGACTTTACTTACGTCGCCCAGGACCCCGTGGACCAGCTGCT

GCTGGGCCCGGCGTACGGCATCCCCAAGATCCTGGAGAAAGCCGGCCTCACCATGAAGGATGTTGACTCCTGGGAGATCC

ACGAGGCTTTCGCTGGCCAAATCTTGGCTAACTTGAAGGCTATGGACTCCGACTGGTTCGGTCAGAACTACTTGGGCAGG

CAAGGAAAGGTTGGTTCCCCGGATATGTCAAAGTGGAACAACTGGGGCGGCTCTCTCTCCATCGGACACCCGTTCGCGGC

TACTGGCGTCCGGCTGGCCATGCACACGGCGAACCGGCTGGTGCGCGAGGACGGGCAGATCGGCGTCATCAGCGCCTGCG

CCGCCGGCGGCCAGGGCGTCGCCATGATCCTGGAGAGACATCCCGACGCTAACGCTGAGTAA

>Cobl_ACAT4

ATGTCTGTGGCATCTAAAGGTATATTCATCGTGGGCGCCAAGCGCACAGCCTTCGGCACGTTCGGCGGCGTGTTCCGTAA

CACGTCGGCCACGGAGCTGCAGACGCACGCCTTCAAGGCGGCGCTCACCTCGGCCGGCGTGGCGCCCGCGCAGGTCGACA

CCGTCGTCGTGGGGCAGGTCATGTCGGCATCACAAACCGACGGCATCTTCACCCCCCGCCACGCCGCCCTGAAGGCCGGC

ATCCCGCAGGAGAAGCCAGTTCTCGGCATCAACAGACTCTGCGGCTCTGGCTTCCAGTCTATCGTGAACAGTGCACAGGA

CATCCTAACAGGCGCAGCACAGATCTCCGTAGCAGGAGGCGTAGAGAACATGTCCCAAGCCCCCTTCGCAGTCCGAAACG

TTCGCTTCGGCACCGTGTTAGGCCAAACCTTCGCTTTCGAGGACACCCTGTGGGCAGGCCTTACTGACTCATACTGCGGG

ATGCCGATGGGCATGACTGCTGAGAAACTGGGGGCACAGTATAAGGTTACCAGGGATGAGGTGGATAATTTTGCCCTGCA

GTCGCAGCAGCGGTGGAAGACTTCCAATGACGCCGGCGTATTCAAGGCCGAGATCGAACCCGTGACCATCACAGTCAAAC

GCAAGGAGGTATCTGTCACCACCGACGAGCACCCACGCCCCCAGACAACACTTGAGGGATTGAAGAAACTAGCCCCCGTG

TTCAAGAAGGAGGGATTGGTGACGGCTGGTTCCGCTTCTGGCATCAGCGACGGAGCCGGCGCCCTAGTGCTCGCCAGCGA

AGAAGCCGCCAAAAACCTGAAACCCCTGGCGCGCCTCGTGGGTTGGTCCTACGTCGGGGTGGACCCCAGCATCATGGGGG

TCGGCCCCGTGCCCGCCATACAGAACCTGCTCAAGGTTACCGGGTTCACGCTCAACGATATCGACCTGATTGAGATCAAC

GAAGCCTTCGTAGCGCAGACCCTGTCCTGCGCCAAAGCCCTAAACCTCGACATGTCCAAACTGAACGTGAACGGCGGCGC

CACCGCGCTCGGGCACCCGCTCGGCGCTTCCGGGTCGCGCATCACCGCGCACCTCGTGCACGAACTCAGACGCAAGGGCC

TCAAGAGGGCGATCGGATCCGCCTGCATCGGCGGCGGACAAGGCATCGCCGTCATGGTCGAATCTGTTTAA

>Cobl_ACBP1

ATGTCTCTCCAAGAGCAATTCAAGTCCGTGAGCGATTCCGTGAGGAACTGGAAGACCAAGCCCGCTGACAGCGAGAACCT

AGCTCTGTACTCCCTGTACAAGCAGGCCATCGCCGGCGACGTCAACATCCCCGAGCCCTCCGGTCTGGTCGAGAACGCCA

AATGGAAGGCATGGACCAGCCGCAAGGGAATCTCTGCTGACGACGCCAAGAAGCAGTACATCGACCTCGCCGGCCAGCTC

CAGGGCAAATACGCGTAA

>Cobl_ACBP2

ATGTCGCTCAAGGAAAAGTTTGATGCTGCTGTGAATGTTATAAGAAGCCTTCCTAAGAGTGGATCGTACCAGCCCAGCAA

TGAGCTGATGCTGCGTTTCTACAGCTACTTCAAGCAGGCGACCGAGGGGCCTTGCGACAAGCCCAAACCTGGCTTCTGGG

ACGTCGTCAACAGAGCAAAATGGGAGTCGTGGAACAAGCTAGGCAATATGACGGAGGATGAGGCAATGCAAGCGTATGTC

GACGAGCTACACAAGATAGTAGAGACGATGTCCTACAGCGCTGACGTGGCGTCCTTCCTCTCCGTGGATGATGAGGGCGA

GGAGTTCCCGAGTCGTGACCTGGAGCTGGTGGCGGGCGACGTGCTCAAGCGCGCGCGCAGCGAGCACAACAGCCCCTCAG

GGTCGCGC

>Cobl_ACBP3

ATGGCCGAGGCACTTAGTTATCCCGATTCAGACTTCTCTGACGAGGACCAGTCACCTCTAGACATATCTTTCAACAAGGC

TGCTGATCATGTGAGGAAACTAACCTCCAAACTTGGTAACAACCAGCTGTTAGAACTCTACGGATTATACAAGCAGGGCT

TAGAAGGCGTATGTAATGTGCCCAAGCCAGGTTGGCTAGATGGTAAGGGTCGTAGAAAATGGGAAGCCTGGAGGGCTCTC

GGAGATATGCCTTCGATCGAAGCTAAGGAAAAATACATTGCGCTAGTCCAGAAATACGACCCTGAATGTTCAGACTTAGT

TGACATAAATACCAAAGAGGCCTGGGTGACCGTGTCATCCCTCCGATACTCTCCCGAGCCTGAGCTTATCCACAACGAGT

TGTCTCTACTAGACGCATCACGAGAAGACTGTGGGGAACGGGTCACGGAGTTACTGTCACAAAACCCTGAGCTTAAACAC

GAGAGGAGCGAGGATGGTCTGACTGCGCTACACTGGGCAGCTGACCGAGACGCCACAAAAGCTTTAGCAGCTGCGTTAAA

AGGAGGTTGCTTCATTGATGCAGTTGATGACAATGGTCAAACCGCATTACATTATGCTGCGTTCTGTGGCCATCTCAATT

CCACAATCATCTTGGTGGAGGCAGGAGCTACTTTAGTGAAGGATAGCGACGACTGCACTCCTCTGGACTTGGCAACTGAT

GTTGAAGTGAAGAAGGTTCTCCAAGGTGCTAAGTCT

>Cobl_ACC

ATGACTCCACCCGTTTTCGGATCTTTTGCCACCATCGCTAACTTCGTATACAATCTATTCACAATGATGAAACGACGGAC

CTCAAAGCGCTTCGTCCTCGGGGATAATGAGGAACAGCCTTCATGGGACATGGATGACGAGGCTACAGCGGTGTTGCCAG

ACCTTCAGAAGTTCCAGATGACCTTAGAAGCGGAGCCGGAGAGCGAGAAGGAAGAGAAGAAGGAGCAGAGCACTAGTAAA

GGGCTGCTGCGGCCGCCTAATCAGACTTTGCAACCGTCAATGTCCCAGGGCACCGTCATCCACAGTCAGCGGTTTCAAGA

GAAGAACTTCACCGTTGCCACTCCCGAGGAGTTCGTTAAACGGTTCCAGGGGACTCGGCCCATTAATAAGGTCCTAATAG

CCAATAACGGTATCGGCGCTGTAAAATGCATGCGCTCGATCCGAAGATGGTCCTACGAGATGTTCAAGAACGAGCGCGCC

GTGAGATTCGTCGTCATGGTCACCCCAGAGGACCTGAAGGCGAACGCAGAGTACATAAAGATGGCGGACCACTACGTGCC

GGTGCCGGGCGGCAGCAACAACAACAATTACGCCAACGTCGAGCTCATCGTCGACATCGCCATCCGGACGCAAGTCCAGG

CGGTATGGGCCGGCTGGGGCCACGCGTCAGAGAACCCGAAGCTACCGGAGCTGCTCCACCGAGCCGGCGTGGTCTTCATA

GGGCCCCCCGAAAAGGCAATGTGGGCCCTCGGGGACAAGATCGCTTCGTCCATCGTGGCGCAGACCGCTGACATACCCAC

GCTGCCTTGGAGCGGGAGCGAACTGAAAGCCGAATACAACAGCAAGAAGATCAAAATATCCTCTGAACTGTTCGCCAAAG

GCTGCGTCACCACGCCTGAGCAGGGACTCCAGTCTGCCTACAAGATCGGGTTCCCTGTGATGATTAAGGCGTCTGAAGGC

GGCGGTGGAAAAGGAATTAGGAAAGTTGACAACCCTGATGACTTTCCCAACATGTTTAGACAGGTCCAAGCCGAGGTGCC

TGGGTCACCGATTTTCGTGATGAAGCTCGCCAAATCAGCCCGGCATTTGGAGGTGCAACTGATTGCCGATCAATATGGTA

ACGCCATCTCTCTCTTCGGGCGCGACTGCTCCATCCAGCGTCGTCATCAAAAAATCATTGAAGAGGCACCCGCCGCCATC

GCTAAGCCCGACGTCTTTATTGAGATGGAGAAGTCGGCGGTACGCCTAGCAAAAATGGTCGGCTACGTGTCAGCAGGCAC

AGTAGAGTACCTCTACGAGCCGGGAACAGGGCAGTACTACTTCCTGGAGCTGAACCCGCGGCTGCAAGTAGAGCATCCTT

GCACGGAGATGGTCGCTGATGTCAATTTGCCCGCCGCGCAGCTGCAGATAGCAATGGGTCTCCCCCTATACAATATCAAA

GACATCCGCCTTCTCTACGGGGAGTCCCCATGGGGTCTCTCCGAGATAGAGCTCGATGACCCCAAGCAGCGCCCGTCCCC

CTGGGGTCATGTTATCGCCGCTAGAATCACTTCTGAGAACCCCGACGAAGGTTTCAAACCCTCATCGGGCACAGTCCAGG

AGCTAAACTTCCGCTCCTCAAAGAACGTCTGGGGTTACTTCAGCGTGGCGGCTTCCGGAGGCCTGCACGAGTTCGCTGAT

TCCCAGTTCGGGCATTGCTTCTCGTGGGGCGAGACGCGCGAGCAGGCCAGGGAGAACCTAGTAATAGCTCTAAAGGAACT

CAGTATTCGTGGCGATTTCCGTACCACAGTAGAATACCTGATAACCCTACTAGAGACCAGCGCTTTCCAGAACAACGACA

TCGACACCAGCTGGCTCGACGCCCTTATTGCTGAGAGGATGCAATCAGAAAAGCCAGACATAATGCTGGGTGTACTCTGT

GGCTCTATCCTTATCGCTGACAGCCTCATCACTACTCACTTCCAAGACTTCAAGAGTGGGTTGGAGAAAGGCCAGATCCA

AGGTTCAAGCCAGCTCTCAAACTGCGTCGAAGTGGAACTGATACACACCGGAAGCAAATACCGAGTGCAGGCGTGCAAAT

CTGGCACCACTACTTACTTCCTGGCTATGAACGGGAGTTTCAAGGAGTTGGAGGTCCATAAGTTGACTGATGGCGGCACC

CTCCTCTCAGTAGACGGCGCTTCCTACACAACCTACCTAAAAGAAGAGGTGGACAAATACCGCGTAGTCATCGGAAACCA

AACCGTTGTCTTCGAGAAGGAGAAAGACCCTTCGAAACTGAGAGCGCCATCTGCCGGCAAACTTATTAACACTCTGATAG

AGGACGGCGGGCACGTTGATAAGGGACAGCCTTATGCTGAGATTGAGGTGATGAAAATGGTGATGACGTTATCAGCTCCG

GAATCCGGGAATGTGACCTTTAATCTAAGACCGGGCGCGGTTCTAGAGAGCGGCTCCCTTATCGGTATTCTAGAGCTCGA

CGACCCGTCCCTAGTGACAACAGCGCAACTGTACAAGGGACAATTCCCTACTGAAGATAACCCCCATTTGTCGGAGAAAC

TCAGTCAGCAACACACCAAGTATAGGGCTATACTTGAGAACGTTCTAGCAGGATACTGCCTCCCTGAGCCCTACAATACA

CCTCGCTCCAGAGATATCGTGGAGAAATTCATGCAGAGTCTAAGAGACCCGTCGCTGCCTCTACTGGAGTTGCAGGAGGT

ACTATCATCAGCCTCGGGCCGTATACCAGTAGCCGTGGAGAAGAAAGTTCGGAAACTGATGGCTCTGTACGAGAGAAACA

TCACCAGTGTGTTAGCACAGTTCCCGAGTCAGCAGATCGCCAGCGTTATTGACCACCACGCAGCATCTCTGCCAAAGCGA

GCCGACCGAGACGTATTCTTCATGAGCACGCAAGCTCTAGTCGTATTAGTTCAGAGATACAGGAACGGTATTCGTGGTAG

AATGAAGGCGGCCGTTCACGATTTGCTCAGGCAGTACTATCAAGTTGAGAGCAACTTCCAACTGGGATCCTATGATAAGT

GCGTGATGGCTCTTCGGGATAAACATAAGGATGACATGCAAGCAGTCGCCGACATCATATTCTCGCACAACCAAGTCGCC

AAGAAGAATCTATTGGTGACCCTGTTAATTGACCACCTCTGGTCCAACGAGCCCGGATTGACAGATGAGTTGGCGGCCAT

TTTGAATGAGCTGACGTCACTCCATCGCGCAGAGCACAGCCGCGTCGCTCTTAGAGCAAGACAAGTACTAATAGCAGCCC

ACCAACCAGCATACGAGCTGCGTCACAACCAAATGGAGTCCATTTTCCTCTCAGCCGTCGACATGTATGGCCACGATTTC

CACCCGGAAAACCTTCAGAAACTGATACTCTCGGAGACTTCTATCTTCGACATACTGCACGATTTCTTCTACCACACTAA

TGCTGCGGTATGCAACGCGTCTCTTGAAGTGTATGTGCGTCGCGCCTACACTTCATACGAGATCCAATGCCTCCAGCATC

TGGCCTTGTCTGGCGAATTGGGCGTGGTTCATTTCCAGTTTGTGCTGCCGACTGGACACCCTAACAGGATCCCCCTCAGC

CAATCAGAGATAGAGCTAGCATCAGCGGAGGACGCAGAAGGCATACCAGCAGAACTGTGCACAGCGGCGATGAGCAAATG

CCACCACCGCACCGGGGCGTTAGCCGCGTTTGACTCCTTCGATCAGTTCGCTCAGTACGCTGACGAGTTACTGGATCTGG

TGCATGACTTCGCTAGCACCGCTTCTGTGAGGAGAGAGGACCTCCAAGCCTTACAAGACGGCAGCGAAGGCCGCGACAGC

ACCAGTATCAACGTCGGTATGGACTTCAAGCCGGTGGATGCTGAAGAAGCGGACCTGGAGCCAATCCACATCCTGATGAT

CGGCGTGCGTGACAACGGGGAATCGGACGACGTCGCTCTAGCTCGTCGTTTTGGTGGCTTCTGTCGCGCGCATCGCCACG

AGTTGCATCAGAAGAGGGTGCGAAGAGTGACGTTTATGTCGCTTATCAAACGCCAATTCCCCAAATTCTTCACCTACCGC

GCCCGCAACGATTTCACAGAAGACACAATTTACCGCCATCTTGAGCCCGCATCAGCCTTCCAACTAGAACTGTACAGAAT

GAGGAGCTACGAGCTAGAAGCTCTGCCTACCAGCAACCAGAAGATGCATCTGTACCTAGGAAAGGCTAAGGTCAAGAAGG

GTCAAGAGGTGACCGACTTCCGTTTCTTCATCCGCTCCATCATCCGGCATCAGGACCTCATCACGAAGGAGGCCAGTTTC

GAGTACCTGCAGAATGAGGGAGAGAGGGTGTTGCTAGAAGCGATGGACGAGTTGGAGGTGGCTTTCTCGCACCCTCTAGC

TAAGAGGACGGACTGCAACCACATATTCCTCAACTTTGGACCCACCGTCATCATGGATCCTTCGAAGATTGAAGAGTCCG

TCCTTGGTATGGTGATGCGCTACGGACCTAGGCTGTGGAAACTAAGGGTTCTCCAAGCTGAGATCAGATTCACACTGCGA

ATCGGACCTGGAGCGCCCACTAAGAACGTGCGTCTGTGTCTCTCCAACGGCTCAGGTTATACCCTGGATGTCTACACCTA

CGAGGAAGTTTCCGATCCCAAGATTGGCGTGATAATGTTCCAATCGTTCGGAACTCGCCAAGGCCCGATGCACGGCCTGC

CTATCTCGACGCCCTACGTTACCAAGGATTATCTTCAGCAGAAGAGGTTTTTGGCAACGTCGCAGGGCACAACTTACGTG

TATGACATGCCCGACATGTTCAGACAGATGGTTGAAAAGAGATGGCGCGAGTGCATCGAGGACGGCAGCGTTGAAGGTCC

GGCACCGGATAACGTGATGTCTGCAGTCGAGCTGGTGGTGGAAGCGGAAGGCGAACGCAGGGTTGTCGAGGTCACGAGGC

TACCTGGACAGAATACTGTCGGCATGGTAGCATGGCGTCTAACCCTCTTCACGCCGGAATGCCCTAAGGGCCGAGACATA

GTGGTCATAGCGAACGACTTGACATACTATATGGGATCTTTCGGACCCCAAGAGGACTGGGTGTATTATAGAGCTTCGCA

GTACGCCAGGGAACGTAAGATTCCTAGGATCTACATGAGCGTAAACTCAGGCGCCCGAATCGGGGTCGCCGAAGAAGTGA

AATCAGCGTTCAACGTAGCCTGGATAGAAGCGGAAAGGCCGGAGCGCGGCTTCAAATATCTCTACCTCACGCCGGAGGGC

TACAGCAAACTGGGCCCGCTGGGCGCAGTCAAGACGCAGCTGATCGAGGACGAGGGCGAGTCGCGGTACAAGATCACTGA

CATCATCGGCAAAGAAGACGGCTTGGGCGTGGAATGTCTGCGCGACGCAGGCCTCATAGCGGGTGAGACCGCGCAGGCGT

ATGAAGATATAGTCACCATATCCATTGTGACGTGCCGCGCTATTGGCATCGGCGCTTATGTCGTCAGACTCGGCCACCGC

GTGATTCAAGTGGAGAACTCCTACATAATCCTAACCGGCTACGCAGCCCTGAACAAGGTGCTCGGGCGCGCTGTGTACGC

CAGCAACAACCAGCTCGGCGGCATTCAGATCATGCACAACAACGGTGTGACCAGCGCCGTCGCTGCTACTGACTTAGACG

CTGTCGGTTCGGCCGTACGGTGGCTCAGTTACGTACCCAAGGACAAACTCTCAATGGTGCCTATAATGCGAGTCTCAGAC

CCTATCGACAGGCCAGTGGAATGGAAGCCTCCACGCTCCGCGCACGACCCGCGCTTAATGTTGACCGGTGACGGCGCCCG

GGGCGGGTTCTTTGATGCCGGCACCTTTGACGAAGTGATGCGTCCGTGGGCGCAGACTGTTATAGCTGGCCGCGCCCGCC

TGGGCGGTATACCAGTGGGCGTGATCGCAGTGGAGACGAGAACAGTGGAACTGACGCTGCCCGCTGATCCTGCCAACTTA

GACTCTGAATCCAAGACTTTACAGCAAGCTGGGCAGGTTTGGTTCCCGGACTCAGCCTACAAGACGTCTCAGGCTATCAA

CGACTTCTCACGCGAGGGGCTGCCCATCATGATATTCGCCAACTGGCGAGGCTTCAGCGGCGGACAGAAGGACATGTACG

AGGAAATACTCAAGTTTGGTGCCGAAATCGTCCGCGCCCTCCGCCGCGCGACCGCCCCCGTACTCGTCTACATCCCGCCG

GGCGCCGAGCTGCGGGGCGGCGCCTGGGCGGTCGTCGACCCGAGTGTGAACAGCGCGAGGATGGAGATGTATGCCGACCC

TGAAGCCAGAGGCGGCGTGTTAGAGCCAGAAGGCATAGTAGAAGTGAAATTCAAGGAACGCGACACCGTCAAAGCGATGC

AGCGGCTTGACCCGCAGTTACAACGACTTATCGCTAGGATCGCTGAACTAAAAGAACAAATAGCAGAGCTGACGAAAAAC

TTCGACCGCCGCGGTTCCATAGACGACGTACTAATAAAGACCGATGCGGGAAGAGAGGCGGAGAGCAGAGCGAGGGAGAT

GGAGACAGAGCTGCTGCAGTGCGAGAGGAGCGCCAAGGCGAGGGAGAAGGAGCTAGGGCCCATATACCACGAGATCGCAG

TACAATTCGCCGAACTTCACGACACGGCTGAGAGAATGCTTGAAAAGGGTTGCATATTTGACATAATCCCCTGGCGCGAC

TCCCGTCGCTTCCTCTACTGGCGCCTGCGGCGTCTGATGCTGCAAAACGAACAAGAGAGACGCGTACAGAGCGCGGTTCA

AACGGCGGATAGTATGGACCACGGGGCAGCCTCCGCTACTCTCAGACGGTGGTTCACTGAGGACCGTGGAGAGACACAGT

CGCACCAATGGGAGCACGACAACGAAGCCGTCTGCCATTGGCTGGAAGCGCAGGTTTTGGACGACAATTCCGTGCTGGAG

CGAAATCTGCGAGCCATCAAACAAGACGCCGTTATGCAGGCTGTCAATGGACTTGTTATGGATCTGACGCCGTCACAACG

GGCCGAGTTCATAAGAAAACTCACCGCACTAGAAATGGAGCAATAA

>Cobl_ACOT1

ATGGAACCGAATCCGGTAATTATTGCAGCGACTGCAAAACAAACAGCATCCCTAATATTTTTGCATGGGCTCGGTGATAC

TGGCCACGGATGGGCGAGCACGATCGCTGCTATAAGAGGGCCTCACGTAAAAGTGATCTGCCCGACGGCCGCGACGATCC

CAGTGACGCTGAACGCGGGGTTCCGGATGCCGTCCTGGTTCGACCTGCGGACCCTGGACGCGACCGCTCCGGAGGATGAG

GAGGGCATCATGAGAGCCACTGGCCTGATCCATGGCCTGATTTCAGATGAGATTAAAGCTGGCATCCCAGCTAACCGAGT

TCTGCTTGGCGGTTTCTCCCAAGGAGGAGCACTGGCCCTGCATTGCGCCCTCACATACACTCAGCCACTTGCTGGAGTCA

TTTCCCTGTCGTGCTGGCTGCCGAGACATGCTCACTTCCCCGATGGAGTGAAAGCACCTTTGGATATGCCGATATTCCAA

GCCCACGGAGATTGCGATCCCGTGGTGCCCTTCAAGTGGGGTCAAATGACTGCATCCTTCCTCAAGACATTCATGAAAAA

TATTGAGTTCACCACATATCAAGGACTAACACACAGCTCCTCGGAGGCAGAGCTTAAAGACATGCGGGCATTCATCGAAA

GGACGATACCTGCCGCCAAATGA

>Cobl_ACOT2

ATGAGGCTATTAGTTTGTTTAGTGTTTATAATCAAAACAATAATTGACCCTGCCGATACGACACCGACTCCGATAGTATT

ATGGCATGGAATGGGTGACACATGCTGTACTGCATACAGCCTAGGTGTGTTTAAACAGTTCTTGGAGAAACAAATACCTG

GGGTATATGTGAATTCTGTAAGGATCGGCAACAACACTATTGAAGACTTTGAAAATGGTTTCTTCATGAACCCTAATACA

CAAATAGAATCGGTGTGCAAACAACTTGCTGTGGACCCTAAATTGCAGAACGGATTTAATGCCATTGGATTTTCTCAAGG

GGGTCAATTTCTACGAGGTGTGATCGAGCGGTGCGGTCACAAATTGCCACCGGTCAAAAACTTCATCACGCTCGGAGGAC

AACATCAAGGAGTGTATGGGCTCCCGCATTGCATGGCCCTGGCTCACAAGACTTGCAACTATATCAGGGAGTTGCTTAAC

TATGCAGCATATCATAACTGGGTGCAAAAAGCTCTAGTCCAAGCGACATACTGGCACGACCCGCTCCACGAAAACGACTA

CAAACGCGGAAGTATCCTCATAGCAGACATCAACAACGAGCTAAATATCAACCAGACATACAAAGACAACCTGAGCAAGC

TAGACCACTTCATCATGGTCAAGTTTGATAATGACTCTATGGTGCAGCCGAGGGAGTCTGCTTGGTTCGGCTTCTATGCA

CCAGGACAGGGCAAGGTTCTGCTGACGTTGCAACAGTCGCAGATTTATTTGGAGGACCGCCTCGGTTTGCAGAAGATGGA

CAAGGCCGGGAAACTCGTGTTTCTCTCCCTCCCCGGCGACCATCTGCATTTCACCGTCGACTGGTTCGTCGCTAACATCA

TTAAACCTTATCTGCTTAATTAA

>Cobl_ACOX1a

ATGACGGATTCCGGTAAAGTTAATCCCGATCTCCAAAGAGAAAGGGACAATTGCACTTTTGATGTTACGGAATTGACAAA

TTTAATTGATGGGGGTGTTCAGAAAACAGAGGAGAGAAGGAAACGAGAGGAAATGGTGCTAAAGGAAGGCATCCACATAG

AAGAGGTGCCGTCCGTGTACCTCAGCCACAAGGACAAGTACGAGCTGGCCGTCAAGAAGGCCTGCTACCTGTTCAAGATG

ATCCGGAGACTGCAGGAGGAGGAAAATACTGGGATGGAGAACTATCAAGAGGTGCTAGGCGGCTCCCTAGGCTCAGCCAT

ACTTGGGGACGGTTCCCCCCTAACCCTCCACTATGTGATGTTCATCCCCACTATTATGGGACAGGCGTCTGTGGAGCAGC

AAGTACATTGGCTCGGGAGGGCCTTCAACTGTGATATTATTGGGACTTATGCACAGACGGAGCTAGGTCACGGTACGTTC

ATCCGTGGCCTAGAGACGACAGCTACTTACGACCCCTCCACCAAGGAGTTTGTCTTACACAGTCCCACACTCACGTCTTA

CAAGTGGTGGCCCGGCGGACTGGCCCATACAGCAAACTACTGCATAGTAGTCGCCCAGCTATACACCAATGGCAAATGCC

ATGGCATCCACCCATTCATAGTGCAATTACGCGACGAAGAAACCCACATGCCACTCCCTGGCATCAAGATTGGCGAGATT

GGCGCCAAGCTTGGCATGAATGGCACCAACAATGGCTTCCTTGGCTTCGACAAAGTTAGGATACCGAGGGAGTATATGCT

GATGAAGAACGCCAAGGTTTTAGAGGACGGTACATACATGAACGCGCCAAGCTCTAAGCTGGCGTACGGAACCATGATGT

TCGTGCGAGTAGTACTGGTCAATGACATGTGCTGTTACATGGCTAAAGCGGTCACCATCGCGACTAGATACTCGGCCGTG

CGCCACCAATCGCAGCCCAAACCCAACGAACCAGAGCCTCAGATCCTAGAATACGTGACGCAGCAGCACAAACTAATCAT

CGGTATTGCGTCAGTACACGCGCTGCGTCTCAGCGGCACCTGGATGTGGAATATGTACAACAATGTTACCGCTGAGCTGG

AGAGCGGCGACCTGGACCGACTGCCCGAGCTGCACGCGCTCTCATGCTGCCTGAAGGCGGTGAGCACGGCCGACGCGGCC

GAGTGTGTCGAGCGCTGCCGACTCTCGTGCGGCGGCCACGGCTACATGCTAGCGTCGAGCCTGCCGCTCATGTACGGGAT

GGTGACCGCCGCCTGCACGTACGAGGGCGAGAACACTGTGCTGCACCTGCAGACTGCCAGATACCTAGTAAAGGCGTGGC

AGCAGGCGTTGGGTGGTGACGTACTCACCCCGACCGTCGCGTACATCGGTAAAGTCAGCAACGGCCGTCGCTCGCCGCCC

TGGGAGAACACCATCCAGGGAATAGTGCTCGGGTTCCAGAGAGTCGCTGCCGGTAAGATCGCTCAGTGCGTGGCGAACAT

TGAAAAGCGACAGAAGGGTGGTATGTCCTATGAAGATGCCTGGAACATGACATCTGTACAGTTGACCGCCGCTTCTGAGG

CCCACTGCCGCGCCTTCATCTTATCCACATACTTCGAGGAAACGGAGAATCTGGTGAAGAATGTTTCTCCAGCGCTGCGC

ACAGTGCTTCTCCAACTAGTGGACTTATATGTCGTGTTTTGGTCGCTGCAGCGCGTCGGCGACCTGCTGAGGTTCACGTC

GATATCGGAGCGGGATATAGAGCAACTCCAGACCTGGTACGAAGATTTGCTGCTGAAGCTGCGCGTTAACGCCGTGGGAT

TGGTCGACGCGTTCGACTTGAAAGACGAGATCCTAAACTCAGCCCTCGGCGCATACGACGGGCGCGTGTACGAGCGGCTG

ATGGACGAGGCGATGAAGAGCCCGCTCAACGCAGAACCCGTCAATGACAGCTTCCACAAGTACCTCAAACCCTTCATGCA

GGGGAAACTGTGA

>Cobl_ACOX1b

ATGTGCGAAGTCAACACAGATTTGCAGAAGGAGCGGGACAAATGCACGTTCGAAGTCAAGGCTCTGACGACTTTTATAGA

TGGAGGGGCCGACCGGACTTCGGAGCGTAAATATCGAGGTAAANNNNNNNNNNNNNNNNNNNNNNNNNNNNNNNNNNNNN

NNNNNNNNNNNNNNNNNNNNNNNNNNNNNNNNNNNNNNNNNNNNNNNNNNNNNNNNNNNNNNNNNNNNNNNNNNNNNNNN

NNNNNNNNNNNNNNNNNNNNNNNNNNNNNNNNNNNNAGGGAAGTGATGAACGGGCTGCTAGGGACATCAAACACGCGTGA

CGGCTCCCCGCTCGGGCTCCACTACATCATGTTCATGCCGGCCATCATCAACCAGGGCAGCGAGGAGCAGCAGGCGCAGT

GGTTACCCAGAGCCTGGAATTGCAGCATTCTTGGCAGTTACGCACAGACGGAGCTTGGCCACGGGACCTTCATCCGAGGG

CTAGAGACAACAGCGACGTACGACCCTGAGACCAAAGAGTTCGAGCTACACAGCCCCACATTAACTTCCTACAAATGGTG

GCCCGGTGGACTGGGCCAAACATGCAACTACTGCATAGTGGTTGCCCAGCTATACACCAAAGGCCAATGTCACGGCATCC

ACTCCTTCATCATCCAAGTTAGAGACGAGGAGACGCACATGCCCCTCCCCGGGATTAAAGTGGGGGACATCGGGGCCAAA

CTCGGGCTCAACTCGGTCAATAATGGCTTCCTCGGGTTTGAGCATTTGAGGATACCGCGGGAACAGATGCTGATGAAGCA

CGCTCAGGTTTTAGAGGACGGTACCTACGTCAAATCGAAGAGCAGCAAACTCAACTACGGCGCCATGGTATTCGTGCGTG

TCGTCATCGTGTACGACATGGTGAACTATCTGTCGAGGGCTGTGACCATCGCCACGCGCTATTCTGCCGTGAGGAGACAG

AGCCAGCTCAGACCGGGGGAANNNNNNNNNNNNNNNNNNNNNNNNNNNNNNNNNNNNNNNNNNNNNNNNNNNNNNNNNNN

NNNNNNNNNNNNNNNNNNNNNNNNNNNNNNNNNGCTGTGGGAGACCTTCCACGATGTGTCGGAGCAGCTGGTCGGCGGCA

ACCTGCAACGGTTACCGGAGTTGCACGCGCTAGCCTGTTGCCTCAAAGCTGTGAGCACGGCCGACACCGCCATGTATGTA

GAGCGCTGCCGGCTAGCGTGCGGCGGCCACGGATACATGCACTCTTCGAACCTGCCTATGACGTATGGCCTGGTCACAGC

CGCTTGCACCTATGAGGGAGAGAATACTGTGCTGCTGCTGCAGACCGCTAGGTACCTAGTAAAGACCTGGCAGGCGATAG

ACAGCACAGATATGGCCCAGATTCCGACGGTAGCCTACATGAAGGCAGCCAGTGCCGCCGACTTCTGCGCTCGCTGGGAG

AACTCCGTGGAAGGCATCATCAGAGGGCTGCAGAAGGTCGCTATGGGAAAGGTGTCTACGTGCGTGAATAACATAAAGAA

GAGGGTCAATTCTGGAATGACGCCCGAAGACGCGTGGAACGTCACCTCAGTCCAGCTTGTTTCTGCCGCTGAAGTGCACT

GCAGAGTAATAATAGTGTCGACATTCTACGACGCAATATCGAAGCTGACCAACACTGTTCCAGCTGACGTGAGACAGGTG

CTGCATCAGTTAGTAGAGCTGTACGCCCTCTACTGGGCTTTGGAGAAACTAAATGATTTGTTGTTGTACACGTGCATGTC

AGAGCCTGACGTGGACGACCTACAATACCGCTACGAGGAGCTGCTGAGCAAGGTGCGACCGAACGCTGTGGGGTTGGTCG

ACGGCTTCGATATCGTTGATGGGATCCTAAACTCCACCCTCGGCGCATACGACGGGCGCGTGTACGAGCGGCTGATGGAT

GAGGCAATGAAGAGCCCGCTCAACGCAGAACCCGTCAATGACAGCTTCCACAAGTACCTCAAACCCCTCATGCAGGGGAA

ACTGGTGCCTCACAAACTGTAA

>Cobl_ACOX1c

ATGGGTGTAAATAAAAAAGTAAACGAAGACTTGCAGAAGGAGCGGGACAAATGCAATTTTAATGTCGCTGAGCTGACACG

GTTCCTTGATGGAAGCGACAAGCTTACGAAGAAAAGGAAAGAGATCGAGAACCGAGTCCTCAGCGTGGAAGGTCTCATAG

ACGCGGTGCCTGATGAGTACATGAGCCACAAGGAGCGGTACGAGAATGCGGTGCGGAAGTCCGTCATCATGCATCAGACC

TTGGGAAACCTGGAAGACAAGACCCTGTCTGTGGTTGAGAGCGCCAGGTTTGCGCTCCGCTCCACCATAACCTCAGCCAT

ATTCAAGGACAACTCGCCTCTGATGCTGCACTACTCCATGTTCACCAACGTGGTGGAAGGTCAGGGCGACGAGGAGCAGA

AGAAATACTGGTTACCGAAGATAAAGAATATGGAAATTATTGGCACTTACGCTCAGACGGAGTTGGGCCACGGCACGTTC

ATCCGTGGTCTGGAGACCATTGCCGAATACGACCCGAAGACCGAGGAGTTTGTGCTCAACAGCCCCAAACTCACAGCGTA

CAAATGGTGGCCTGGTGGACTGGCGCACACAGCCAACCACTGCGTCGTCATGGCACACACCTACAGCCAGGGCAAGAGCT

GCGGCATCCAGCCCTTCGTGGTCCAGATCCGGGACCTGGAAACCCACATGCCGATGAAAGGCGTGAAGCTTGGAGAAATC

GGCGCTAAACTCGGCTTCAACACTGTGAATAATGGATTCCTGGGCTTCGAGCACCATAGGATCCCTAGGAATAAGATGCT

GATGAAGAATTCTCAGATCATGAGGGACGGAACATTCAGAACAGGTCCCAACAGTAAGCTGACGTACGGCACCATGGTCT

ACATCAGGGTATCCATTCTGGGATCGATGGCCAATGCCTTGGGCAAGGCGGTCACCATCGCTACCCGCTACGCTGCGGTC

AGGCGGCAGTCGCAACCAAAACCAAACGAGCCGGAGCCTCAGATCCTCGACTACGTGACGCAGCAGCACAAGCTGTTCAT

CGGCATCGCCACCAGCCACGCGTACTACGTCAGCTGCTTCTGGCTGTGGACGCTCTACAGCAAAGTCAACAAGGAGCTGG

CTGCTGGCAACCTGGACAACCTGGCTGAGCTGCACGCGCTAGTTTGCTGTCTCAAGGCGACGACATCAGCTGACGCAGCA

GTGTTAACCGAGCGCTGCCGACTGGCTTGCGGCGGCCACGGATACATGCTGTCTTCGAACTTGCCCCAGACTTATGCTAA

CGTCACCGCTGCCAACACCTATGAGGGAGAGAACACTGTGCTGCACCTGCAGACTGCGAGGGCTTTGGTGAAAGCCGGCA

TGCAAGTAGCGCAAGGGCAGAAAGTGAGCCCCTCCATGGAGTACCTAGCCGACCAGAGCAAGATTGCCAAGTGGGACAAT

TCCGTGGACGGCATTGTTAAGGGATTCCAGAAGGTTGCTGCTGGGAAAGTGGCAGCTTGCCTGGCCAGCCTGCAGAAGCA

CACCAACAATGGGCAATCATCCGAAGACGCGTGGAATTTGACCTCAGTGCAACTGGTGGCTGCTTCTGAGGCCCACGCCC

GCCTGATTCTGGTGTCAGTATTCAAGTCCCAGATGGATGCAACGACGTTCACGCTCAGCGGAGAGACCAGAACTGTCCTC

TCTCAGCTAGTGGAGTTGTACGTGGTTTACTGGGCACTTGAGAAAGTCGGCGATTTGCTACTGTACACCCCGATTACCGG

CAAGGACGTGCAGGAGCTGCAGCAGACGTACGAGGGTCTGCTGGCGAAGATCAGGCCCAACGCCGTCGGCCTGGTCGATG

CCTTTGACTTCAGGGATGAGGTCCTAAACTCGGCTCTTGGCGCATACGACGGGCGCGTGTACGAGCGGCTGATGGATGAG

GCAATGAAGAGCCCGCTCAACGCAGAACCCGTCAATGACAGCTTCCACAAGTACCTCAAACCCCTCATGCAGGGGAAACT

GGTGCCTCACAAACTGTAA

>Cobl_ACOX3a

ATGGAAGACTTGAGTTTCGTGCCTGATCTGCCGAGCGGGCCTCTGGATGCCTACAGGAGTGCTGCTTCATTCGACTGGAA

GCGTTTAAAGCTGGCTCTCGAAGGGGACATAGAGAATCTTAGACTTAAGTACAAAATATGGCATACGCTAGAGAAGGACC

CCCTCTTCTCGCACCACTCTGTGACCCCGCCCACCGAGGAGCAGAAGCGCCTCACGCAGCTCCAGGTCCTCAGGATAAAC

CAGTACAAGTTCCTGTCAGATGACGTGTTCAGATCCAGTTATAGTAAAAAGACCAGAGCACTGATGTCCCTCAACGAGGC

GGTGCAGTCCCTGAACCCGAGCGTGTCTGTCAAGATGGCGATCGGGATATACCTCATCTCGAACGCTCTGCTCTCGCTAG

GCACCGAGCGCCACTACAAATTCTATGAGGCCACTTTAATTAAGAGAGAGATCCTAGCATGCTTCGCGCTGACAGAGGTA

GCGCACGGGTCTGACGCTCGACTCATGCGCACCACGGCTACCTACGACGCAAAAACGAAGGAATTCGTCATCCATTCGCC

AGACTTTGAGGCGGCCAAGTGCTGGCTCGGGAACCTAGGCAGAACATGCACGCACGGCCTCCTCTTCGCGCAGCTGATCA

CGCCGGACGGGACCAACCACGGGCTTCACGGGTTCGTGGTCCCCATACGGGACCCGGCCACGCTCGAGACGTACCCCGGC

CTGATAGTGGGGGACATGGGAGAGAAGATCGGGGTCCACGGGATTGATAATGGATTCATGATGTTCAACCAGTACCGGAT

ACCGCGCGAGAATCTCCTGAATCGATCAGCCGACGTAACGGAGGAAGGCGTGTATGAGAGCAAGTTCTCAGAGCCTTCCA

GAATACTCGGATCCGCCTTGGAGAATTTGTCTGCAGGTCGCATAGGCATAATGCAGGAGAGCTGTCACTCCATATCGAGC

GCTGTCTGCATCGCGATCCGCTACGCCGCCACGCGACAGCAATTCGGGACGGCAGCGCAAGAGATACCGCTCATCGAGTA

CCAGTTGCACCAATGGCGACTGTTCGGATTCGTGGCTGCAGCGGTGGTGTTCAAATTATACATTCAGAATATGACACAGG

TGTATTTGAAGGTTGTGGAGAACTCTAACGCAGGCTTGAAGCTCGAGAACTTGAGCGAGACGGTATCAGAAATCCACGCG

ATGGTATCAAGCAGCAAGCCTATGACGACGTGGACGGTGCTCCGAGCTGCACAGCAGTGTCGTGAGGCTTGCGGTGGACA

CGGGTTCCTCAAGAGAGCAAACCTCGGCGACATCCGCAGCAACCACGAAGTGACCGTCACCTACGAGGGAGACAACAGCG

TGCTCTCTCAGCAGGCCGGCAACTGGCTGCTCCGGCAGTTCGAGTGCGCGCGCGCAGGACAGCCAGTGGACTCGCCGCTG

GGGACCGTCGCCTTTCTGCCGCGCTGGCAGGCCATACTAGCCACCAAGTTTAGCTGCCAGGGGACTGGCGAGCTGAAGAA

TACTGAATTCATCCTCTCAACCTACCGATGGCTCCTCTGCTGGCTGCTAAAGGAGACCCACGAACAATACGAAACCCTAA

TCGCTTCCGGCCTCACAAAGTTTCAAGCCAAAGCCAAAACGCAAGTCTACAAATGGCAGACACTAACTGAAGTATACGCA

GAATACCTCGCTTTAATATTTAGCTTAGAATCCATTACACGGAAAGAAAAGAAGCTCCAACCAATTCTAGAAAAACAATT

CTTGCTTTATGGTCTATGGTGTTTAGACAGGCATTTGGTGGAGTTGTACCAAGGGGGGTTCGCGACGGGAGAGGGATTGG

CTAAGCTGGTCAGGCAGGCTGTGTTGGAACTCTGTTCGGATGTGAAGGGCGAGGTTGTCAGTATTGTGGACGCGCTGGGC

CCGACGGATTTCGTGCTCAATTCGGTGATTGGGAAGAGCGATGGCAAGTTGTACCAAAACCTACAAAAGGAGTTATTCCA

CAACCCGGGAGCGATGCAGCGGGCGACGTGGTGGCGAGAGGTAGTGCCTTCCAAACTC

>Cobl_ACOX3b

ATGGAGGTAAAAAAAATACCACCAGCCAGTGATGAAGAACTCAAGAGTTGTTTCTCTGATCTGCCTTCAGGGCCTTTGGA

CATATATAGAAAGAAGGCTTCTTTCGATTGGCGGCGAATGAAACTAGCTTATGATAGCTTAAACACTATTAAAACCAAGA

ATAAAGTATGGAACTTCATGGAGTCCCATGCCCTCTTCAAACACCCGGAAGCCACTCCGACGTTGGACGAGCAGCGCCAA

ATTGCCACAAAGAGGATGTATGTCATCCACAATGTTGACTTTGTGCCTTTGGAAGAGATAGCCGACGACCCGAGACTATT

CCAATCCTTCACGGAAGCAATCTTCATGTTCGACAGCTCGCTAGCCGTCAAACTGTCTCTAACGTTCCGTATGTTCGCAA

ACACCATCCGAGGCAGCGGCAGGGAACACCACTTCCATCTAATCGAGGATAATGACAATGGGAAGATCGGTGGATGCTTC

GCTTTAACGGAGATTGCTCACGGTTCCAACGCTAAAGGAATGAGGACAACGGCTACGTATAATGTTGAGGAGAAGTGCTT

TTTCATGCATACTCCTGATTTTGAGGCAGCTAAGTGCTGGGTCGGATCTATGGGAAAATGCGCGACCCACGCAATAGTCT

ACGCGAAACTAATCTCAAAGGGCAAGAACCACGGCCTGCACTCGTTCGTTGTACCCATTCGGGACACGCGGACCCTGAAA

CCCTTCGCTGGTATCACTGTGGGCGATATTGGGGAGAAGGTTGGACTTAATGGGATTGATAATGGTTTCCTAATGTTCAA

CAAATACCGGCTGCCGAAAGAGGCATTACTGGACAAACTGGGAGGCGTAGACGACAACGGCGACTACAAAACTCCTTTCA

AAGACCCCAGCAAGAGGTTTGGAGCTGCTCTCGGTATTCTGTCAGGCGGCAGAGTGCACATAACCTCAATTTCAACCAAC

TACCTCCAAAAAGCAATAGTCATTGCTGTCAGGTACTCAGCAGTCCGAAGGCAGTTCGGACCAGAAAATGCTACCGAAGA

GACTCCAGTATTGGAATATCAGCAGCAGCAAATCCGTCTCCTGCCATACTTGGCAGCAACTTACGCTATGCGCGTGTTCT

GCAACTGGTTTGGGGCAATGCACGTTGCTATGACCATTAACAGCATGCTGGGTGCGGACGACAACGCAGCGGCGCGTGGA

ATAGAGATGCACGCTCTCTCGTCCGCTGTGAAGCCTGTGTGCGGCTGGACGGCCAGGGACGGCATACAGAATTGCCGGGA

ATGCTGTGGAGGGCATGGATATTTGAAAGCCTCTGCCATCGGTGACCTTCGCAACGATAACGACGCTAACTGCACATACG

AAGGCGAGAACAGCATGCTACTCCAGCAGACCAGCAACTGGCTTCTAAATGTTTGGGCCAAGCGGGCCACGCCAGGGTTC

GCTGACACCCCGTTCGGTTCCATCGAGTTCCTGAACGGAGCTCAGGGTTTACTGGCTGGGAAGTGCCAATGGAACAGTGT

GAAGGAGATTGTGCAGCCTGCCAACGTCGTGCACATGTACAAATGGCTGACAGCCTACATGGTGAAACTGACAGCGGAAA

AGGTGGCCCAGCTAAAAAGCCAGGGCAAGGACAGTTACGAAGCCAAGAACGATTCGCAGTCGTACAACGCTGTCACACTG

TCTGCTGTGTATGGAGAGAACTTCATCGTGAACCATTTCTACAAAGCGGCGCTGGCGTTCAAAGACGCGGCGTGCAAAGC

CGTGCTTCTGAAACTTGTGTCGCTGTACGGATCTTTCCTACTGGAGAAACACATGGCTACTTTATACATTGGTGGGTTCT

TCTCAGCGGAACAAGGCCTTCTACTTCGTGAGGGCATTCTCTCTCTATGCGCTGAGCTGACACCAGAAGCGGTGGCGCTA

GTTGACACCCTGGCGCCCCCTGATTGGTGCTTGAAGTCGGTGCTAGGGCAGGCTGATGGTGAAGCCTATAAGCATATCCA

AGACAGCATAATGACGTACCCTGGATCGATGACAAGGCCTGAATGGTGGCGCGACGTCGTTCACTGGGAGACCTACGTGC

CGGCCAAATTGTAG

>Cobl_AGPAT1

ATGATTAACTTAAACATAATAAAACAATCGACCTTCGTCCATGTCTGTTTTGCTATTTCCTACTTCACATCGGGAATATT

ACTCAGCATGGTGCAAGCATCCTTATTCTATGGACTCAGACCTTTCAACAGGAGTTTGTACAGAAAAATAAACTATTATC

TTGCTTACTCACTCAACAGCCAGTTGGTGTTCATGTCACAATGGTGGGCAAGGACCAAGATGTCAGTTTACATCAAGAAG

GATGAATATGACAAATACTATGGCAAAGAGCATGCTTACCTTTTCATGAACCATAGCTTTGAAATAGACTGGCTTATGGG

CTGGCATTTCTGTGAAGTCATCAAAGTTTTAGGAAACTGTAAAGCCTACGCCAAGAAATCAATCCAGTACATGCCGCCCA

TCGGATGGATGTGGAAGTTTTCCGAATTCGTGTTTTTGGAGAGGTCATTCGAGAAAGATAAGGAAACTATAAAGACGCAG

ATAAAAGAAATATGTGACTACCCCGATCCAGTTTGGCTACTAATGACCCCAGAGGGCACGAGATTTACGAAGAAGAAACA

CGACGCGTCGCTAAAGTTTGCGAAGGAGAAGAATCTACCGCTCCTAAAGCACCATTTGACGCCGCGCACCCGGGGGTTTA

CTACCAGCCTGCCCCACTTCAGGGGGAAGATTCCAGCCATTTACAACATACAGTTGGCGTTTGATAAAGATGCCAAGAAC

CCGCCAACCATAACGAGCTTGCTCTACGGAAAGCAAGTGCACGCACATCTGTATATCGAGAGGATCCCCTTGGAAAAAGT

GCCGGTGGACGAAGCAGCCGCATCGCAATGGGTCCATGACGTTTTTGTTATTAAGGACAAGATGGCGGAATCTTTCCACA

ACACTGGAGATTTCTTCCTCGAATCCGGAGTGGAACGGATCGAGCCTTTCGACTACCCAGTCCGTATCTACTCTTTAATC

AACACGCTGGGGTGGGCGATTATCACGCTTACGCCGATGCTGTATTATCTCATGGGATTGCTGCTGAGCGGCAAGCTTCT

GTACTTCTCTATCGGTGTCGCCATCTTCTTTGGATTTTACATCATCCTGCAGAAATCCATAGGCATGTCAAAAATTAGTG

AAGGTTCATCTTATGGGACTGAAAAGAAGTAA

>Cobl_AGPAT2

ATGTCTGCCTTCAACGTCGTGCTGGGGTGCGTGATGGCACTTCTCTTCATATTATTTACGACAAGTGCTATCGCACGATA

CTATATTAAGTTCACATTTTTCATGGTAGCGTCGCTGGTGTTCTCGTCGGCCCCACTCCCGCTGATGCTGTTCAGACCCT

TCAGTCCGAAGAATGCATTACTTCCCGCGGCGCTGCTACGGTATTCGGCGAGGCTTCTCGGGATCCGCTTCAAGGTGCGA

GGGCTCGAGAACGTGGACAACTCGCGAGGCACTGTCGTGTTGCTGAACCATCAGAGCGGACTGGACCTCTTTATACTGGC

AGTGCTGTGGCCGCTGATGGCTCGCTGCACGGTGATATCGAAGCGCTCGATGCAGTACGTGGTGCCCTTCGGCACCGCCG

CTTGGTTGTGGGGCACGGTGTTCATCGACCGCGGGTCCCGCTCCGCGCACGACGCCCTCAAGAAACAAGCGAGGGCCGTC

AAGGAAGAAAAGCGCAAGCTGTTGCTCTTCCCCGAGGGCACACGGCACGGCGGTGACAAACTGCTGCCCTTCAGGAAAGG

GGCCTTCCACGTCGCTATGGACGCAGGGGCCGCCATCCAGCCAGTCATCGCTTCCAAGTACCACTTCCTTGACTCCGAAC

GACACCGGTTTGGTTCAGGTGAGGTAATCGTCACGATATTACCCATGGTCGAAACAGCCGGCTTGACCAAAGACGATCTC

GAGGCGCTGATAGAGAAGACTTATGCGAATATGCAGGAGACGTTCACCAGGTCGTCCCAGGAGACCCGCCGGCCGGTTCC

AAGATTGAACGAGTAA

>Cobl_AGPAT3

ATGGCTTCCTCCTATTCATATCCTGAATTATTTTTGGCGGGTTTTATATTTACCTTGCCATTTCTTTACGAAAAAAGTGA

TGTTTTTCGATATTACCTAAAGTTTTTTCTTTATTACGTATATGTTCTAACGATTTGCACTGTCTTGTTACCCGTAGTTC

TATTATATCCGCGCGACGTAACTAACTTAGTTGTTGCTTCCAGGTTTTGCCGCTACGCCTCTTATATAGTGGGAATAGAA

TGGGAGTTGAGGGGTCTCGAGCACTGGGAGAGTGACCAGTGTTACATTGTGATTTCCAATCATCAGAGCTCCCTGGACAT

CCTAGGGATGTTTGAGATGTGGCCTCGAATGAAGCGCTGCACCGTGGTTGCTAAGAGACCTCTGATGTTCACCGGAGCAT

TTGGATTTGGTGCTTGGTTGTCTGGGCTGGTATTCATTGATCGCCTGAAGACTGACCGAGCTCGGCTGCTTATGAGAGAA

GCTACTGAGCGTGTTATTCAAGAAAAGACTAAGCTCTGGATATTCCCGGAAGGGGCGAGGTTCAACAAGGGCTGTATCCA

GCCATTCAAAAAGGGAGCGTTCTATCTAGCCATTGACGCTCAGATACCTATAATGCCTGTTGTATTCAGCCAGTACTATT

TCCTAGATAGTGACACTAAAACATTTGAGCCGGGAAAAGTAGTGATCACCACCCTGCCACCTATACCAACAAAAGGAATG

ACCCGCGACGACATTGAAGTACTCTCCGAGATGGCGCGCCAGAAAATGATCGAGGTATTCAACGAGAGTTCTAAGGATCT

GGTAATGCAAAAAAAAAATCGCCATCTAATTCAGCCATTGCAAAACAACTGA

>Cobl_AGPAT4

ATGTGGGACATAGCAATTTATCTAGTCGTAATAACTTGTGTCGCCTACGTGCTGAAACAATTATTTTCAGAGACTCCCAA

TTTTGTGAAGTTCAAGTTGAAATTTCTAATTTATTACGTATGGACCTCCTTCATTTCCCTGATGTTGATGCCATTCTTCA

TACTAGATCCAAAGAACGTGAAAAATATTCTATTCTCCGTTGCTATTATTAAACATGTAACAAAAGTAATCGAGGTGAAA

TGGCATCTAAGGAATGGGAAAATCTTGGCGGAAGACAGAGGAGCGGTGATTGTGTCAAACCATCAGTCTACTATTGATGT

TCTGGGCATGTTCAACATTTGGCATGTAGCTGATAAAGTAGCCGCTGTTGCCCGGAAAGAACTATTCTATGTGTGGCCTT

TCGGTCTTGCTGCATACCTCTGTGGAGTGGTGTTCATAGACAGAAACAATGCCAAGGATGCCTACAAACAACTGCAAGTT

ACATCAGACATCATGGTGAAAAATAAGACTAAGATCTGGCTGTTCCCGGAAGGCACCAGAAATAAGGATTTCACGAAGCT

GTTGCCATTCAAGAAAGGAGCTTTCAACATCGCCGTGGCAGCACAAGTGCCAATTATTCCTGTTGTTTTCTCCCCATACT

ACTTCATCAACAGGCCGAAGTATATTTTTAACAAAGGACACGTGGTCATCCAATGCCTGGAAGCCGTGCCGACAGCGGGT

CTCACGATGGAGGACGTGCCCGAGCTGATCGACCGCGTTCGCAGCACCATGGAGATGGCATACAAAGAGCTCTCCAAGGA

GGTCCTTAGTGCCCTCCCGTCCGACTATCCGCTGTACAGTGAGAGTGCCATCTGA

>Cobl_CSP1

TGCCAATGCCGCGCTGAAGCTCCGACGTACACGACGAAGTATGACGGGATCAACTTGGACGAGATACTAGCCAACAATCG

ACTCCTGACTGGCTACGTCAATTGCTTAATGGATTTGAAACCCTGTACGCCTGATGGAAAGGAACTGAAGAAAAATCTCC

CCGACGCCATATCGAACGACTGCGGCAAATGCACGGAGCGGCAGCAGCAGGGGGCCGACCAAGTGATGCACTACATCATC

GACCACCGCCCCGACGACTGGGAGCAACTCGAGAAGAAGTACAACTCCGACGGCTCTTACAAGCGAAAATACATGGAAAA

G

>Cobl_CSP3a

ATGAAGACCATAATCCTCGTTTGCTTCTTCGCCCTGGCCGCGGTGGCCTACGCCCGTCCCGGGGACGTCTACACCGACAA

GTTCGACAATGTCGACCTGGACGAGATCCTCTCCAACCGTCGGCTCCTCATCCCGTACCTTAAGTGCGTGCTGGAGCAGG

GGAAGTGCACCGCTGATGGCAAGGAGCTGAGATCTCACATCAAGGAGGCTCTGGAGAACAACTGCGCCAAGTGCACGGAG

GCCCAGCGCAAGGGCACCCGCCGCGTCATCGGCCACCTCATCAATAAGGAAGACGATTACTGGAACCAGCTCTGCGTCAA

GTACGACCCTGACCGCAAATACGTCGCCAAGTACGAGAAGGATCTGAAGACTGTCGCTTAA

>Cobl_CSP3b

TTCGACAATGTCGACCTGGACGAGATCCTGTCCAACCGCCGACTCCTCATCCCATACCTCAAGTGCGTGCTCGAGCAGGG

AAAATGCACCGCTGACGGCAAAGAGCTGAGATCCCACATCAGGGAGGCTCTCGAGAACAACTGCGCCAAGTGCACCGAAA

CCCAGCGCAAGGGCACCCGTCGCGTCATCGCTCATCTCATCAACAAGGAGGACGACTACTGGAACCAGCTCTGCACCAAG

TACGACCCTGACCGCAAATACGTAACCAAATACGAGGAGGACCTGAAGACTGTCGCTTAA

>Cobl_CSP4

ATGTCAATCAGGTGTTTAATTCTGGTCACCGTGCTCTCAGTGGCTGTCGCGGACTTCTACAGTCCCAAGTACGATGACTT

CGACATCCAGCCTATCCTTGAAAATGACAGGATCATGGTCAGCTACACCAAGTGCTTCCTCGACAAGGGTCCGTGTACAC

CGGACGCTAAAGACTTTAAAAAGGTAATTCCGGAGGCTCTCCATAGCACCTGCGCGAAATGCACCCCGAAGCAGAAGCAG

TTGATCAAGAAAGTCATCAAGGGCATCATGGAGAAGCATCCTGACTCCTGGAAGGAGCTCGTGGAGAAGTTTGACCAGGA

CAAGAAGTATAGAGAGAACTTTGATAAATTTCTAGCCGAAGATAGAAAGTAA

>Cobl_CSP6a

ATGAAGACTGTACTGATTTTGTGTCTGGCCGCGTTTGTGGTCGCAGAAGAAAGGAAATATGACGACGTTGGTGATGTTGA

TCTTTCGAGCGTGCTAAACAACCAGAGGTTATTGGTGCAGTACACCAACTGCCTAATTGACAAAGCCCCCTGCACCCCTG

AATTGAAACAACTGAAAGAAAAACTGCCAGAGGCTCTGGAAACCCATTGTGCCAAGTGCACAGACAAGCAGAAGACAGTC

NNNNNNNNNNNNNNNNNNNNNNNNNNNNNAAAGCACCCGGATCTATGGCAGAAACTGGTTGCAAAATATGATCCTACTGG

CAAATACCATGAGTCTTTCGAAGATTTCCTTAAGAACTGA

>Cobl_CSP6b

ATGATTTTGTGTCTGGCGGCGTTTGTGGTGGCAGAGGAACAGAAATATGATGATGTTGATAACATTGATCTTTCGAACGT

GCTAAACAACCAGAGATTGTTGGTGCAGTACACCAACTGCCTCATCGATAAGGCCCCCTGCACCCCTGAATTAAAACAAC

TGAAAGAAAAATTGCCAGAGGCTCTGGAAACCCATTGCGCCAAGTGTACAGACAAGCAGAAGACAGTCGGGAAGAAGTTG

GTTCAGGACTTGAAGGCAAAGCACCCGGATCTATGG

>Cobl_CSP7

ATGATCAAATATTTTGTGATCCTGTGCTNCGTGGTCCTGTGCTGTGTGGCGTTTGCGCTGGCCGAGGAAAAATACACGGA

CAAGTATGACAACATTGACTTGGACGAAATCTTGGACAACAAGCGGCTCCTCCAGGCTTATGTTAACTGCATACTCGATA

AGGGCAAGTGCACACCTGAAGGCAAGGAGCTGAAAGATCATCTCGAAGACGCTCTCACGTCAGGTTGCGAGAAATGCACA

GAAGCCCAGAAGAAGGGGTCATCAAAGGTCATTGAGCACCTCATCAAAAACGACCAGCCCATCTGGCAGGAGCTGACTGC

GCGCTTCGACCCTGAGGGGAAATTCAAGAAGGTGTATGAGGAGCGCGCGAAAGCCAATGGCATCACGATCCCGGAGTAA

>Cobl_CSP9

ATGAGGAGCTGCATTGTGTTCGCTTGCCTGCTTGTGTCNNNNNNNNNNNNNNNNNNNNNNNNNNNNNNNNNNNNNNNNNN

NNNNNNNNNNNNNNNNNNNNNNNNNNNNNNNNNNNNNNNNNNNNNNNNNNNNNNNTTCTTGGACAAGGGCCGGTGCACGC

CCGAAGGCTCGGATTTCAAAAAAACTCTCCCCGACGCCGTCGAGACAACGTGCGAAAAGTGCACAGAAAAACAGAAGATC

AACATTAAGAAGGTGATCAAAGCCATCCAGGCCAGGCACCCGAAGCAGTGGGACGAGCTCGTCAAAAAGAACGACCCTAC

CGGCAAATACATCGCGAACTTCAACAAGTTC

>Cobl_CSP10

ATGAAGTTCGTCATTGTCCTGTCAACCCTGATAGTCCTGGCTTTCGGGCAAGGCACCTACACCTCGGAGAGTGATAACCT

GGACATCGATGCCATCGTGAGCAACCCTGAGAAGCTGCAGGCCTGGTTCGGCTGCTTTGTGGATAAGAGCCCTTGCGACA

AGGTGCAGAGCAGCTTCAAAGTGGACATGCCCGAGGCGATCCGCGAAGCTTGCGCAAAATGCACCTCAGCACAGAAGGCA

ATCCTCAAGAAGTTCCTCACAGGCCTTCAGGAGAAGTCACCAGCCGACTACGAAGTCTACAAGAAGAAATACGACCCAGA

AAACAAATACTTTGCACCACTTATTAAGGCTATTGCATAA

>Cobl_CSP11

ATGTGGGCAGTACTTTTCGTGTTCGCGTGTGCGTCCGTGGTTTTAGCACAGAATATAAACGATGTGAGGAACTTGCCGAA

ATACGATAAGAGATATGACTATTTGAGTGTTGATGCCATTCTGGAGAATAAGAGACTTGTCAGGAACTATGTGGATTGCC

TGATCAACGCGAAACCTTGCACGCCTGAGGGGAAGGCGTTGAAAAGGATACTGCCAGAAGCGTTGCGTACAAAGTGCATA

CGTTGCACCGAGAGCCAGAAGCAGACCGCAGTGAAGGTGATACGGCGCGTGAAATCAGAGTACCCTGAGGATTGGCACAA

GCTCGCCTCCCGCTGGGACCCTACTGGCGACTTCACCAGATACTTCGAAGAGATCTTGATAAAGGACCACTTTAATACCA

TACCTAACAGCAACGAGATCCCCGGCTCCTCGCCAACCACGCCGCCCGCGCGCTGGCCCAGCACGCAGCCACCGGCACCC

AGCACGCAGCCACCGTCGCCGCTACCGCTGTTCATACCCCCCAGCCTGCCAACGTTCCCGCCCGCCAGCCCGCCAGCGTT

TGTGCCCGCCAACCAGCCAGCGCTGGTCACGCCGCCGCGACCGGCTGTCTTGAACCGTTTTGACGAGGATGGCGAGTTGA

TGGTGAACCAGAACAACCCGCAGGCGCGCCCGCCGTCACCGCCGGTCGTCCGCCAGACCACCGCGCGACCTGTCGCCACC

ACCCGATCTTCCCAAATGACCTGGGCGACGGCGGCTTCTGACAACATCCCTACACGGTTCAACCTTCGGCCAACGAAGGA

CATCTCACCTCCGTACACCACAGCTATAACCCTGATCGACCAAATCGGGTACAAGATCATACGGACCACGGAACTGGTGA

CTGACATACTGAGGAACACCGTCAGGGCAGTTGTGGGGTGA

>Cobl_CSP13

ATGTTCTATTTATACCTGTGTGTGTTGTTGTTTGGTGCAGCGTACGCGCAAGATGCGTTAGTGAACACGACCCAAGGCCA

AGTGCAGGGGGCATTGGCGAACGATGGAAACTACTTAGTGTTCTACGGGATCCATTATGCTGGGTCAGCGTCGGGGACTA

ACCGATTCAAGCCACCAACGCCGCCTGCGCAATACCCAGGAGTGTTCCATGCGGTTGACAACAGCGTCATTTGCGCGCAC

CCCACCCCGCGCGGCATCGTCGGAGTAGAAGACTGCCTGGTCCTCAGCATCCACACAAGAAACCTGACTTCACCCAAACC

AGTCTTAGTTATTCTCTTAGGAGACGAGTACACCTCCATAGACACCAGGACTTACTCCTTCAGCCGGCTAGTCCTCTCTG

ATGTCGTAGTAGTGAAGATGAACTATCGACTATCGATCTTCGGCTTCTTGTGTTTGGGCGTGCCGGACGCACAGGGCAAT

GCCGGATTAAAGGACGTCGTTCAAGGATTAACTTGGATTAGGAACAATATTGCCGGATTTGGTGGCGATCCGAATAATGT

TGTACTGTTCGGACACGGCTCAGGAGCGGCCATGGTTGATTTAATTACGATGTCGCCTTCAGCTGACAATTTAGTGCACA

AAGCAATCACGCAAAGCGGGTCCGCTCTCGCGCCCTGGGCAGTCGCGTACGAACCGCTTCAATACGCTCGAGTGTTTGCT

GAAAAGTTAAGCTATTCCGGTGACACGCCTCAAGAACTTGCTCGAAAACTAGCGGTAACCGACTTGAGTTTACTGTCAGC

CGCATTACAAGACTTCAGTTTCTACAACAATTCTGCCTTGTTCGCACCCTGCCTTGAAAACAGAGCGTTGAACAATACGT

TTATCACCGAAGCGCCTATCGACCTACTGCGTTCTGGAAACTATAGCCAAATCCCTTACATTGCCGGGTATACTGACAGA

GAAGGCACGATCAGATCAGGACAAGCTGTAAACGATGGATGGCTAGAGCAGATGGAAGCGAACTTTACAAACTTCGTCCA

AGTTGATTTAGCGTTCAGCAGCATAGAGAACAAGACTGCTGTAGCGACTAACATCAAGCAGAACTACTTCGCAACGAGTG

CTGTGAGCATGGCGACTATAAGAGATTACTTGAACTATCACGGAGATACAATGATACGCGTGTCGGTGATACGAGGGGCA

CGGGAAAGAGCGTTGACTTCAAGAGCTGATGTAAGACTGTTGGAATTCACATACAGAGGAACCAGGAACTCTGATTGGAT

TTTCCAACAGATACCTATTGACGGGGTGCCCCATGGCGGCCTGCTGAACTACTTGTTTGATTACGACTTAAATCCTGCAG

ATGAAGCAATCAGAGCTTCGATTTACAGACGTTACATTTCGTTTGCTAATACCGGAAACCCTATCAACAATGCCTCAGAA

CAAACTATGTGGAATCCTGTCACCAGCTCAGCTACCAACTACCTGATCTATACTGGAGAAACCAGTAACCTAACTACCAT

CTGGGGAGAATCTGCTCGGTCCAACCCCCACTCAGAAATCATGACATACTGGGACAACCTCTACCGCATTTACTACCAGC

CTCCAGTTTCTATGTCGTCGGCTGATAAAGTGATTAGCGTTGCATTTGTTTTGTTGCTATCTCAATTTGTGCAGCGGCTG

TTATAA

>Cobl_CSP14

ATGAAGACCGTGCTAGTTCTGTCCGTGTTGGTGGCAGCGGCGCTCGCGCGGCCCGACGAGCCCTTCTACAACACTCGTTA

CGACAGCTTCGATGCGCAGGAGTTGGTCGGCAACATCCGCCTGCTGAAGAGCTACGGCAACTGCTTCCTCGGCAAGGGAC

CTTGCACTTCTGAGGGGACCGATTTTAAGAAAACGATCCCAGACGCTCTCAGGACCAGCTGCGGCAAATGCTCGCCGAAG

CAGCGCGAGCTCATCCGAACAGTGGTGAAAGGCTTCCAGTCCAAGCTGCCAGAGCTCTGGAAGGAGCTATCCCAGAAGGA

AGACCCCACTGGCCAGTACAAAGCCAACTTCAACAAGTTCCTGAAC

>Cobl_CXE1

ATGTGCTATCAACGGACTCTAGTCGGAGACATATTTCGGGGACAAGAACATTGTCTGATACTAAATGTGTTCACTCCGGT

GTACCCCTCGGAGACTCTGCTTCCGGTCATGGTATTCATACACGGAGGAGGATTCATAGAGGGATCAGGAACACCTTTGG

TATATGGCCCAAATTATTTGGTTCCGAAAGGTGTAATTTTAGTGACAATTAATTACAGGCTTAATATTCACGGGTTCCTC

GGTCTGGGAATCAAGGAAGCGCCTGGCAACGCTGGAATGAAAGATCAAGTCGCAGCATTGAAGTGGGTTCAGAAGAATAT

AAGAGTGTTTGGAGGTGATCCTGATAATGTGACTCTATTCGGCGAGAGCGCTGGTGGTGCCTCGGTGTCGTACCACTTAC

TGTCTCCTATGTCCGCTGGGCTCTTCCATAAGGCAATAACACAGAGCGGGTCAGCCCTGACGGCATGGGCACATCAAGCG

AACCCCGAGTACAATGCTCGACTTGTTGCAAAAGCTATGGGATACAATACTGAAGATCCTTACGAACTGTATAACATTTT

CAAAAACGCGCCTTTAGATGAACTTACTAAAGTAACCATCCCTCTAGATGTAAAAAAAGTGATGTTATCTAGACTCATTT

ACTCACCCAGTGTTGAAAAAGTATTTGAAGGAGTAGAACCTTTTTTGACAAAGGATCCTTACAAATTATTTCTCAATGGA

GAGTACAACAAAGTTCCTATGATTACAGGAACAAATGACGAAGAAGGCTATTTATTCGCAGGCGTAGAGGACCCTTTAGT

CATACCTAATGTTGATATCGAGCGCTCTCTACCAAATAATTTAGAATTTCCGTCAGAACGTGAGAGAAAACGAGTTGCAG

AAGAAATTAGGAACATGTATATGGGTGACGAACCCATATCGGAGTCAGCTTCCTCTCAGAAGAAATTTTCCAGATTTCAT

GGAGAACCATTCTTTAATTATCCTCCGATGGCTGAAACAGAACTCTTGCTGCATTCAAGTGGTCTCCCGTTATACCATTA

TTACCTAACCTATGATGGGTGGAGAAACTTTGGAAAATTTGCGTCTGGTGCCATCTTCAGAAATTCTTCTGGAGTGTCGC

ATGCAGACGACTTATTTTATTTATTTTATCAACCCTTTTTACCGGGGTGGATTGAGATGGAAATGATCAATAAAATGACT

ACCTTATGGACCAATTTTGCAAAGTATGGAGACCCAACACCTGAAACGACGGAGNNNCTGCCTGTACGCTGGCTACCCGC

TACCAAGGAGGCGCCACAGGCTTTTGTTTTAGACAAGAAGCTGTCTACCACCCCAATGTGGCATAGAGAGTCGTTGCGAT

ACTGGAAAGACGTGTACTCAAAATATAGGAGGAAATAA

>Cobl_CXE2

ATGGTTCTGGTAAAAGTGAGCGAGGGTACGTTGGAGGGTTCCCACGAGCAAAATCCGTATGGAGGCTCGTTCTACAGCTT

TAAAGGGGTACCTTACGCTGAACCACCGATTGGAGAGCTTAGATTTAAGGCTCCACAGCCTCCCGTACCATGGAAAGGTA

TACGATCTGCAAAAGAGTTGGGCTCCGACTGTTATCAAGCAGCCGGCTTCGCTGGAGTACCCGTGGCAAAGGGCAGCGAG

GACTGCCTCTATCTCAACGTCACCACGCCTGAATTGACACCTGTCAAGCCTCTCCCTGTCATGGTCTGGATCCACGGTGG

CGGATTCTTCAGCGGCAGCGGCAGCGACGAAACCTATGGCCCTGAATTTCTCATCAGACATGGCATTGTCTTTGTCTCCA

TCAACTACAGACTTGACGTACTCGGCTTTCTTTGCTTAGACATAGAAGAAGTGCCGGGCAACGCTGGACTCAAAGACCAG

GTAGCAGCTTTTCGTTGGGTCAAAAAAAATATCAGCAATTTCGGTGGGGATCCGGAAAATATTACCATATTTGGTGAAAG

TGCTGGAGGTGCTAGTGTCTCCTATCATTTGATGTCGCCCATGACAAAGGGATTGTTCAAGCGTGCTATAGCTCAGAGTG

GAGCGTCCATCTCTAACTGGCCCAATATCGATGAACCACTTGAGAAAGCCGTAAAATTCGCAAGAAAATTGGGTTTCAAG

TCAACAGATCCACAGGAAATCTACAAATATTTGAAATCTCTGCCAGTGGAAACTATTGTAAAATTAAACGTCCCGGTATT

ATTTGACGAGCAAGCTGTTTTTCCCGGTCGCTTAAATTTTGCAGTGGTCAGTGAAAAGAAATTTGGTGACAATGAAACCT

TTTTCAGCGGTGATGTGTTTGAAGTCTTGCGCCAAGGCATCCACGAAGGAGTTGAGGTGATGTCCGGATATAATGAACAC

GAAGGTGTTTTTGGATTACTTCTCAGGGGATATTACGACAATATTTTAAAAACAGCAGAGTTTTCGGAGTTCTACTGCCC

AGAAGACATAAAATATAATTGTTCGGCGAAGGAATCATTAGAAGTGGGCAGGAAAGTAAGGAAGTTTTACCTCAAGGATG

AATTGGTTTCAAAAGAAATCATGTTACCCATTGCACGATTTTGCTCCTTATACGATTTCATATTTGGTGCTATTAGCTGG

CAAAAGATTGCAGCAAAGGCGAGTCGAAATAAATTTTACTTGTATAAATTTACCTGCAAGTCTGGAATCAACAAGTTTAC

AGACTTATTGGGTTTGCGGGATCTGGTCGGACCTGGTGACTTCGTGTGCCACGCTGACGATACAGGGTATCTGTTCTGGG

CAAAGATGTTCAAAAAGACAGTAGATATGGACTCGAAAGAGTTCAAGATGATTGAAACCGTCAACACGCTCTGGACGAAC

TTTGCTAAATACGGGAACCCCACTCCTGATGACTGCCTGGGTGCGACATGGAACCCCTACACCCTGGATAAACAGGAGTT

CCTGGACATCGGGGAACAACTTGTCCCCGGGACTAAACCTGAGCAAGAAGATGTAGAATTTTGGGAGGATATTTACAAGC

AGTATCTCCCTAAACATGTTCTTTGA

>Cobl_CXE2.2

ATGAGTCAAGTGGAAGTCAGTGATGGCCTGGTGGAAGGTGAGCTGGTGGAGAACCCCTTGGGTGGACAGTTCCATAGCTT

CAAGGGGATTCCGTATGCTGCACCGCCTGTTGGAGATCTGAGGTTTAAAGCCCCGCAACCCCCAACTCCATGGGAAGGAG

TCCGCAGTGCCACAGAATTTGGATCCATCTGCTATCAAGTAGACCCTTACTTCGACCCCACGCCTAAGGGCAGCGAGGAT

TGCCTGTACATCAACGTGTACACTCCTGATCTGACTCCAACCAAGCCCTTGCCTGTCATGGTTTGGATCCACGGCGGAGG

CTTTATCTCCGGCAGCGGGAACGATGACTTCTACGGCCCTGAGTACTTGGTCAGAAACGACGTCATCCTAGTCACTTTTA

ACTACCGGTTGGAAGTCCTCGGGTTTCTTTGCCTTGACACTGAAGAAGTACCCGGAAACGCGGGTATGAAGGACCAAGTG

GCGGCTCTGCGTTGGGTCCAGAAAAACATCAGCCAGTTTGGTGGAGACCCTGACAATGTCACCATTTTTGGAGAGAGCTG

TGGAGCGGCTAGCGTCAGCTTCCACTTGATCTCGCCCATGTCTAAAGGACTGTTCAAACGCGCTATTGCGCAGAGCGGTC

ATGTCACTAATGGCTGGCCGGTCTCTGAAGTGCCGCTGTTGAGGGCTGCTGCGTTAGCTCGACAGCTGGGCTTCGAATCC

AATGACCCTCAAGAGTTAGCAGCGTTCTTTAAAGAGCAACCTATGTCCTCCCTTGTTAAAGTGAGGCCCCATATCTTTTT

AGCTGAAAAAGTGATGGATGCCATCGATATATTCTTCGCAGTGGCCAGCGAGAAGCAATTTGGTGACAACGAGAGATTTT

TCTATGGTAACCCAATCGAAGCGCTGCGTAACGGTATCCATGAGGGGGTAGAAGTCATCACCGGGTACAATGAACACGAA

GGTGTGTTCAGTTTCCTTCTCAGAGGAGGCTACGATAAAATTAAAGATCAAGCAAAAACCTACTTTGAATACTTCGTTCC

GAGACCTTTCTTGTTGAACTGTGCGCCACCTGTGCAGTTCGAGGCAGCCATGAAAGTAAAACAGTTCTATGGGAATGGAG

GCAAGGTGGAAGACGTTGACACGTTGACGACGTTCTGTACCGTGGAAGATTTCGCTTTCGGAATCATACAGTGGCAAAAG

ATAAGTGCGAAAGCGAGTAAGAATAAATTTTATTTCTACAAGTTTTGTTGCAAGTCTGAGTTGAATGTGCTGACTCATGC

CTTCGGGCTGGGGGAGACCTTTGGACCTCGGCATATGGTGTCCCATGCCGATGATCTGCCATACCTGTTCCCCATGAAAA

TTATGAACATGAAGATTGACCCTGAATCCGCTACATTTAAAATGGTTGACAATGTGACGAAACTTTGGACGAACTTTGCT

AAATACGGGAACCCGACGCCAGACGAAAGCCTGGGTGCCCAGTGGCCCGAATACACCACAGAGACAGAAGCTTACCTGGA

CATCGGAGAACAGCTGGTACCATCATCAGCTCCCCACCAGAAGGAGGTGGAAATGTATGAGAGCATCTACAGGGAACATC

TGCCGCATTTGATCGCTTGA

>Cobl_CXE4

ATGGTCAAAGTAAAGGTACAGCAGGGTTGGTTGGAAGGAGACAAGCGCGAGGTGGTGACTAAGGATGGCCACTACTTCAG

CTTCAAGGGAGTGCCCTACGCAGCCCCACCCGTTGGTAAACTGCGGTTCAAGGCGCCGCAACCACCCCTGCCATGGGACG

GAGTAAGAAAGGCGGTCGAACACGGCCCTGTCAGCCCCCAGCAGGACATATTCACCCAAGAACTGATCCCCGGCAGTGAA

GACTGCTTGTACCTCAACGTGTACTCGCCAGAGCTCGAACCTAAGACTCCTCTTCCAGTCATGTTCTTCATCCACGGGGG

AGGCTACAGGAGTGGTTCTGGCAACGACTCCCACTATGCTCCTGATTTCCTAGTCAAACACGGGGTGGTCCTGGTAACGA

TTAACTACAGATTAGAAGCCTTTGGCTTCCTTTGCCTGGACACCAAAGATGTCCCTGGAAATGCGGGGTTAAAAGACCAA

GTTGCAGCTCTGAAATGGGTGAAGGAGAATATAAGCAAGTTTGGTGGTGATCCCTCCAATGTTACGGTCTTTGGTGAGAG

CGCCGGAGGAGCGTCAGCAGCGCTCCATGTTCTGTCTCCATTGTCGAAAGGGTTGTTTAAGCGATCGATTCCAATGAGTG

GTGTGCCATTGTGTGACTGGTCCGTGGCCTTCCAACCGGCCAAGCGAGCCTTTACTCTTGGTAAAATATTGGGATTCGAA

ACCGATGATCCCGATAAACTTCTAGAGTTTTTACAAGGCGTTCCATCAGAGAAACTTATCCTTACCGATCCTTGCGTATT

GAGCTTTGAAGAAAAAGCCAACAACCTTTTGAAAATGTACCACTTCACGCCGGTTGTTGAAAAAGACTTCGGACAAGATT

ATTTTATGATCGAGGAGCCGTTGGAAATTTTGAAACAAGGAAAAACTAATGCAGTTGATGTACTAATAGGGCATACGAGC

GAAGAATCTCTTCTTGGAGTACCTGTTTTCGAAAGCGTGTTGATAAAACAGTACAACAGGTATCCAGAAATATTAGTACC

ACGGGAACTGCTTTATCAGTGCACTCCTAAGAAGATTCTGGAACTGTCACAAAAAATCCAAGAGCATTACTTCGGCAAAA

AACCGATCGGTGTGGACACCATGAAGGAATTCGTCGGCTATGTTTCCGAAACCTCGTTCATATACGATATCCACAGGTAC

TTGGAGAAATTACCGAAGAATGGCAAAAACAAGACATATTTCTACAGATTCTCCAGTGTTTCTGAGCGCAATATTTACGG

CGCTAGCGGTGCTAAGTTCGGGCTTACTGGTGCAAGCCACTTAGACGACTTGATGTACCTGTTCCAAGCCAATTATGCTA

GTGTGCTGATTGACAAGAGCGGCAAAGGATATAAAATGATTCAGCTCGCTTGCACAGTCTTCACTAACTTTGCGAAATAT

GGAAACCCAACTCCTGACGCCTCTTTGCCGACCTGGCCGACCTACGACAATGCCTCGAAGAGCTACGGAGACATCAGCGA

CACAGTGACCATTGGACAGGCACCGCACTCCAAGGCAGTAGCCTTCTGGAAGTCCGTGTTTGATGCGGCTGGAGTTGACT

TCTAA

>Cobl_CXE6

ATGAATCCTTTAGACGCAGTCGATAAAAAGATAGTATGTCCACAAGGCAAGTTTCCATTCGTGGATATCACAACGTACAC

CATGCAAGAAGACTGCTTAATTGCAAATGTTTACATGCCGGACACAGAGGAGACCAATCTACCCGTACTAGTNNNNNNNN

NNNNNNNNNNNNNNNNNNNNGGCTTTGGTTACTTATTTGGACAGAAGAGTTTGGTGAAGTCTCAAAAAATTGTAGCAGTC

AACTTTAACTATCGGCTTGGTATACACGGCTTTCTATGTCTCGGCACAGAGGGCGCTCCCGGCAACGCGGGCATGAAGGA

CCAAGTGGCGCTGCTGCGCTGGGTGCAGAAGAATATTGGCAACTTTGGAGGAAACCCTGATGACGTCACTATTGCTGGAG

GCAGCGCGGGATCCTCGGCAGTAGATCTGCTTATGCTTTCTAAAGTGACAGATGGTCTTTTCAATAAAGTTATACCCGAG

AGTGGTGCCAGCCTTAGCCCATTTAGTATTCAGCTAGACCCAGTACAGACTGCGAGAGAATACGCCAAAAAGCACGATTT

TAATGAAGTTGACGACGTTCACGCCTTAGAAGATTTCTACAGAACGGTTTCCTATGACGTTTTACTCAGTGACATATCTC

TTTACTCAAACGTATCCCTAATGTTCATACCATGCGTAGAGCGATACGTTGCAGGCATTGAAACATTCCTTGAAGATACT

CCGGTAAATATTTTGAAACAAGGAACATACAAAAAGGTTCCTTTGCTCTACGGATTCACAAATATGGAAGGCTATATGTT

TCTAGGGGCATTTGACGCTTTAAAAGATAAAGTGAATGAGCGCTTCTCCAACTATATACCTAATGATTTACAATTTGACT

CTGAAGCGCAGAAGGTGGAAATAGCACAAAAAATTAAAGAATTCTATTTCGGAGACCAACCTATTAGTGAAGACAATATT

GTTGATTTTATCAACTACAACTATATTGTGTTTGGCTATCCGCATCTAAGATCTACACAGTTGCAAGTAGAAGCCGGTAG

CCATTCTATATATTTGTATGAATATTCATTTCCATCTCCTGTCCCTGAAGGTGATCATGTACCTGAAATCATGAAGAAAG

TTCAGGGAGCGCCTCATTGTGCACAGTCTGCAGCAGTTCACGATGTTGTATTTGATTTAGTGCCACTTGATTCGAGTGAG

GAGTATGTAAAGCTGAGAGAAACTTTGAGAGAGATGTGGCTGAACTTTATAATAACTGGAAAACCGGTTCCGGAAGGATC

TGAATTGCCAGCATGGCCTCCAGTTGGAGCCAATAGATCCCCCTACATGGACTTAGGAGAGGAAATAAAGCTCAAAAGTT

CACTTCTAGAGGAGCGAGCTCGTTTCTGGGATGGCATCTACGAACAATTCTACAAGTCACCCATTGCGCCGAAGCACACA

CGAGTTCGAACTGAACTGTAA

>Cobl_CXE11

TTCCAGGCTTCCATACCCCACCCAATATTCGAGGACACTTTCGAGGCCTACGACGACTCGGCTCTCTGTCCTCAGTTAGA

TGACAACACCAACACCATCAGAGGAACCCTGGACTGTCTCCATCTCAATGTCTTCTCACCGAACTCTGCACACTCCGGCA

ACCTCCTGCCAGTAATGGTCTGGATCCACGGAGGCTTCTTGCAAAGAGGCGCTTTCGGCAGGCAGACTTACGGACCAAAG

TTCCTTGTCAGACACGACGTTATTTTAGTAACTATTAACTACCGCTTAGGACCTTACGGGTTTATGTGTCTTGACACACC

AGAGTATGCTGGGAACCAAGGACTGAAAGACCAGATAGTAGCTTTGAAGTGGATTCAAGATAATATTAGAGCTTTCGGTG

GGGATCCAGCTCGAGTCACCTTGGCAGGACAGAGTGCTGGAGGTGCAGCCGTAGATTTCCATTTGTTTTATCCTGGAGAA

AGGCTCTTTAATAAGGTAATACTACAGAGCGGAGTATCTTTAACTCCTAGAAGACTTGTCGAATCAGATACCACGAAGCC

AATTACGCTAGCTGCACATTTCGGATTTGTTACCAGCAATTTAGACGAAGCTCTATCATTCCTAGCAACCGTAGAGACCG

ATTTAATCATAGCAGCAACGCTTGAACTAGGGTTAGAATTCAGATCGTGCGTTGAGAAGAAGTTTGATGGTGTGGAAACA

TTTATACCGCAGCATCCTGCTAATGCCGATAGACCACAAGTTTCAAACATTCCTGTCCTGATAGGGACCGTTGACAAAGA

ACTGTTAGCAAAATATATCAACCAAAATTCTGAATATTTTAGAAACCTTAATGTGTTTGAAGAGGAATTGGAAAAGACAT

TCGATTTCGGTGAGAATTTGGACGCTATGCATGAGGTTGTAAGAAACTTTTACGTCGGTGATGATGTCATGACTGAAGAT

GACAGATGGAGTGTCATAGACTTTGAATCAGATTTTACTTATGTCCATGCTGCCCAGCGCGCTATGAGGAAGTATATTCA

AAGTGGGGCAGAAAATATATTCTTCTACGTCTTTACTTATGAAGGTGGAAGAAATATGATTAAATATATGAATGGCATAT

TACATGTGCCAGGAGTGGCTCATGCCGATGAACTGGGGTACATTTTTGACCCCTCGTTTATCACAGAAGAACCTACTCCA

GCAGACCAGGCTGTTATTGACAGAATGACTACAATGTGGACTAACTTCATGAAGCAAGGAAACCCAACCCCTGAAACATC

AGAGCTACTGCCCGTCTCGTGGACACCAATCACAACTGAAAAGTGGCACTGCCTCAACATAGGTGCAGAGTTAACCCTCC

AAGGCAGGCCTTTCCACAGGAGAATGGCTTTCTGGGATCTGTTCTTTAGCATGAATGGGAAATTAGAAAGGGGAGTAGAG

ATTGTAAGAGAGTAA

>Cobl_CXE12.1

ATGAGGGACAACGTCGTTGTCATCACATTCAACTACAGGGTCGGGCCGTTTGGTTTCCTCTCGCTGAACTCTTCGAGTAT

CCCGGGCAACGCGGGGCTGCGGGACATGGTGACGCTGCTGCGCTGGGTGAAGGACAACGCGGCGGCCTTCGGCGGCGACC

CCGACGACGTCACGCTGGCGGGCCAGAGCGCCGGCGCCTGCTCCGCGCATCTGCTCACACTGTCGCAAGCCGCGCAGGGG

CTTTTTAAGAGAGCTATACTAATGAGTGGGTCAGGTGTACGGAACTTTTTCTCCCCGTCGCCGGTTTATGCGCAGTACGC

AGCGGACTTGTACCTTACCCAGCTCGGCATCAACGGCACCGACCCCGAGGAGGCGCACCGCTTGCTCGTCGCCATGCCAC

TCGAGGATATCATCACTGCCCATAACGCTCTTCAAGACACTATCGGTATCACAGTTTTTGCTCCAGTAGTGGAATCCCCG

CATGCCGGCGTGGAAATAATCTTGGATGACGACCCAGAAGTTTTGCAAGCTCAGGGACGAGGCAAGGACATCCCCCTGCT

AATAGGTTTCACAAACGCCGAGGGTGAAACTTTCCGCCCTATATTTGAGGCTTCTGGCATAGCGAGTCAAATCGAAAGCA

ACCCTCAGGTGTTGCTACCTCCAGGGGCGTTATATGCTCTGCCAACGGATGAGGTCCTTCCCAAAGCGAGACAAATCGAG

CAGAAATATTTCAACGGAACGGCGACTCTGGACAGGTTCTTAGAGATGGCTACAGACTTTTACTTCGTGTATCCTACGTT

GAAGCTGGCGGAGGCGAGAGCCGCGAACGGGGGTGCTCCCTTGTACATGTATCGGTTCGCGTACAACGCCGACTACAGCG

TGTTCAAGGAAGCACTGAACCTGACATACAGCGGTGCGGGACACTCGGAGGACGTCACCTTCGTGTTCCGCGCGAACTAC

TGGCTCGGGAACATGACGTTGTCATCAAACGACGGAACGATGGTGGACATCATGACGACGTATTTCACTAATTTCATGCG

ATACAGTGATCCATTAAACGGAGAAGCTGGGTGGCCGGCAGCAGGTGCAGAAGGTGAGGGGCTGGCGTACCAGGACATCG

CCGGCTCCGAACCTAGCTCCGTGGAGGTGAGCGGGGCATATCAAGAGGTGCTAGACTTCTTTGATGACATCTACACCGTT

AACAGTACGGCTACAAAGTGA

>Cobl_CXE12.2

ATGAGGGACAACGTCGTTGTCATCACATTCAACTACAGGGTCGGGCCGTTTGGTTTCCTCTCGCTGAACTCTTCGAGTAT

CCCGGGCAACGCGGGGCTGCGGGACATGGTGACGCTGCTGCGCTGGGTGAAGGACAACGCGGCGGCCTTCGGCGGCGACC

CCGACGACGTCACGCTGGCGGGCCAGAGCGCCGGCGCCGCCGCCGTCCACCTACTCACGTTGTCTGAAGCCGCACGGGGA

CTCTTCAAAAGGGCCATACTGATGAGCGGCAGCAGCTCACGAAGTTTCTTCTCGACCTCACCGGCTTACTCCCAGTACGT

GGCACAGCTGTTCCTCACAAAACTTGGCATTAACGGCACCGACCCTGAGGAGGCGCACCGCCAGCTTATAGCCACGCCCA

TCAAGGATATCGTCGATGCACATGACTATCTTCAAGATATTATCGGTATCACAGTTTTCGCACCGGTCGTCGAATCCGCA

CAACCCGGCGTGGAAACGATCTTGGATGACGACCCGGAAGTTTTGCAATCGCAAGGGCGAGGCAAAGACATCCCTCTGCT

GGTGGGCTTCACAAGCGCTGAGTGCCAAACGTTCCGCCCACGATTTGAAGCTCTCGACATCATGAGCCGGATCGAAGAAA

GCCCTGTGCTGGTGATGGCCCCCGGGGCAATTTATGTTACGCCACCGCAGGAGATGCCCAACAAAATAGGACAAATCATA

CAGCGATACTTCAACGGAACACCGAACTTGGACAAGTTCATAAGGTTGTGTTCGGATTCCTACTTCGTATACCCTGCTTT

AAAGATTGCGGAAGCGAGAGCCGCGAGCAACGGTGCCCCCGTGTACTTGTATCGTTTCGCGTACGAAGCCGACTACAGCG

TGTTCCAGAGATCGCTCGGGCTGAAGTTTCGCGGCGCGGGGCACTCGGAGGACCTCACCTTCGTGTTCCGAGCCGACCAC

GTGCTCGGGGACAGGCCTTTGTCCTCGAGAGACCGGATGATGGCCGACGCCATGACGACGCACGTCACCAACTTCATGCT

GCATAGCAACCCAACCAACGGAGCATTAGGGTGGCCGGCAGTGACGCCCGAGAAGCTGCAGTATCAGAACATCATCACCC

CCGATCTCAGGACCACCGAGGTCAGCGGGAGTCAGCGCGATATGATGACGTTCTTCGATGGCATTTACAGCTGA

>Cobl_CXE14

ATGTCTAAGCTGTTGACTTGGTGTATTTTTAAAGTGCTGTTCCTTCAGTCACAATGCGCTTCCTTACACACCCCCGTGCT

TCAAATCCCTCAAGGGAGATTGGTGGGCCTGGAAACCAAACGAGGATATTTGAGGCAGTATTTGGGAATACCTTACGGCA

CGGTGGATGAAAGATTTCAGGAAGCAGGACCACCCCCGCGCTGGCTCGGGGTTTTCAACGCTTCAGACATGAGCATCGCC

TGTGCCCAATACCACGAGCGGCTAGGGCTACCCACAGGGGTCGAAGACTGCCTCACGCTGAACGTGCACACCCCTGGCCA

AGTCAGCTTCAAGCGGCTCTACCCCGTCATGGTGTTCATACACGGAGGCGGCTACAAGACTGGCAGCAACACCAACTATA

TCTACAACCCACAACTTCTAGTCCAAAAAGGTGTCATCGTCGTCACCGTTAACTACCGCTTAGGTGCCTTTGGCTTCCTC

TGCCTCAGGATCAAAGGTGCACCGGGAAACATAGGCTTGAAGGATCAAGTAGCAGCTCTTCGATGGGTTAAGGAGAATAT

TAGAACTTTTGGTGGCAATCCCGACTCGGTGACCATATTCGGCGAAAGTGCAGGCTCCGCGTCGGTCAGCTACCTCATTA

TGTCGCCCACAGCTAAAGGACTGTTCAGACGAGCCATCATGGAAAGCGCCTCAGCTCTCTCACCGTTCGCTTTTTCCAAC

GATCCCATCGAAAGAGCATCATTAGTTGCATCCAAGATGGGTTACAACACCAAAAACCCTTTTGAACTTTTAAGGATTTT

TCGGAATGCTACCAAAAACGAGATTTTAATGGCCAGTGCATCGAACACGTCGGCTAATTTGTGGTCGAAATACGTTTTTA

GGCCGTGTGTCGAAAAACAGACGGTAAGCAGTAAACCGTTCCTTACCATGAGCCCTCAGGAAATGCTGGAGTCGGGGACT

TACAATAAAGTGTCCATGATCATTGGCTACAACGACAAAGAAGGCATACTTTATGTAAAGCATTACAACAAGCAACTATA

CCAAAAGTTAGACAATAATTTCAGTGACATACTGCCTGACAATCTTTACTTTTCCGACAGCAGAGAAAAACGAAAAGTCG

CAAGTAAAGTTAAGTCGTTTTACTTTGGAAATCGGACAATAAATGAAGATTCTGTCGACGGTTTAATAGATTTCATATCT

GATGTGATGTTCCATTATCCTTCAGTGTCAATAACGGAATACTTCTTGAACCACAATCACTTGCCTATCTTCAATTACTA

CTTTAAGTATGATTCCTTCAGGAACCTGGCCAAGATTCTGTTGGGGATGAAAGGGCAGAAAGGGGCAGCCCACGGTGATG

AGCTGTTTTATTTATTCCAGCCTGTTATATTTTGGCCTGTGCCTTTAGTTGGAAATGATAAAAAAGTCGTGGAAAGAATG

ACCAGTATGTGGACGAACTTTGCTAAGTTTGGAAACCCAACATCGTTGAAATCGCCAGTGTTGACTGTAAATTGGACTGC

CAGTGACGACACCAGCCTAAGATATCTCACCATAGATAAAGGTTTATCCATGGATTATCTGCCAAATCCCGAACGCATAG

CCTTCTGGAGAAATCTTTACAAGACGCAACAAAATTACTTCCTAAGATCTAACCGTTTACTTAATATAACTTTTTAA

>Cobl_CXE15.1

ATGACGAGACTGCTGCTGCTGCTGGTGCTCTGCGCTTGCGCAGCCGCGTATTCAGGTTCTACAGCCACTGCAGCACCCAC

CAAGCCTACAGCCCCTACTAAGGGCGAGGCCTCTACCAAACCCGAGGCCCCTACGTCGCCCGCGCCCCCGACCAAGCCGG

CGCCAGTAACGGTGACGCCCTGCGGCGTTTTCCGCGGCTCGTGGATGGAGTCGCGGCGCGGGCGCAGCTTTCAGTCATAC

CGCGGCATCCCCTACGCGCAGCCGCCCACAGGAGAACTGAGGTTCCAGCCGCCTGTAGACATGCTGAGGTATACTTCTGA

GGTGGACGCGAGCGAGGATGGCCCCGCGTGCCCGCTGCCCGTCGAGCCTGGCGAGTACTACGTCGACGAGGACTGTCTGC

GGCTCAACGTCTACACGCCTGCCAATAACAGTACCAAAAAGCTGCCGATAATATTCTTCATCCACCCGGGCGGCTTCTAC

TCCATGACCGGGCGCAGCGACCTCTTCGGGCCGCATTATCTCCTGGACCATAACATCGTGCTCATTACCATCAACTACCG

CCTCGCCTCGCTCGGTTTCCTCAGCACGGGCGATGCGGTGGCTCCCGGCAACAACGGCTACAAGGACCAAGTATCGGCGC

TGCGCTGGGTGCAGCGCAACGCAGCGGCCTTCGGCGGAGACCCGGGACAAGTGACGATCGCGGGCTGCAGCGCCGGCTCC

ATCAGCGTTATGCTGCACATGATCTCGCCTATGTCTAAAGGTCTATTCCACCGCGGTATCGCATCGTCGCCGGCACCGAT

GGGCAAGGAGCCACTGCCTTCGCATCAATTAGACCTGGCGCAGAAGCAGGCGCGCATCCTCAACTGCCCCACCAACTCCT

CGCGCGCTATCATCGACTGCCTCAAGACCAAAACTTGGCAGGAGCTGGGGAACTCGCTGCTTGGATTTTGGGATCAATTC

GGCTTCGATCCCATCTGCTTGTGGACGGCTGTGGTCGAGCCGGACGTGGGGCAGCCGCGCTTCCTGGCCGAGCAGCCGGT

CGCGGCGGTGCGCTCGGGCCGCTTCTACAGCGTCCCGCTGCTCGTCAGCCGGACTGAAGACGAGTTCTTTTGGAAGGCTT

TCAGTGTGACCAAGAACAAGACGCTCCTGGACCGCATGAACGCGGAGTGGGAGACGATCGCGCCGCTCTCGTTCGACCTG

CCCAAATTGCCTGCTACCAACGCGGCCGCTGCCAGCCGCCGCCTCAAGGAGGCGTACCTGGGAGGGCAGGATGTGGCGGA

TACTGCTGAGAGCGCGGATGGCCTGGGAAAACTGTATGGAGACTCCTGGGTTGGACTCGCGGCGCACAGGTTGGCCAACC

TGATGTCCGTACATTCTTCTAAGCCTGTCTGGTACAGCGACTTCAGCTACATCGGCAACAACAGTTTCTACGAGGACGAA

AAAACCGGGAAACCCGCTGGCGCAGCTCATCATGACGACCTGATCTACCTGTTCTCGATAAGCTACTCCCGCCCCCCGAT

CGCGGCGCAGTCGCCGCCGCCGCCCGCGCCGCAAGACTCCGTCATCGTGGACCGCATGACCGCCATCTGGTACACCTTCG

CCAAATACGGAGACCCCAACCCCCGTAAAGGCGAGCTGCCGGAGCTGGCGACGCTCAACTGGCCGGTCATGAAGCCGGAA

GACAGGAAGTACCTTCGTGTAGACAAGGAGTTCTCCGTCCATGAAAAAATGCACGAGAGTCAACTCCAAGTGTGGGAGGA

GCTGTACCCTATCGTGTATTAG

>Cobl_CXE15.2

GTGACGCCCTGCGGCGTTTTCCGCGGCTCGTGGATGGAGTCGCGGCGCGGGCGCAGCTTTCAGTCATACCGCGGCATCCC

CTACGCGCAGCCGCCCACAGGAGAACTGAGGTTCCAGCCGCCTGTAGACATGGTGAGGTATACCTCCGAGGTAGACGCGA

GCGAGGATGGCCCCGCGTGCCCGCAACCCGTCGCGCCCGGGTACTACGTCGACGAAGACTGTCTGCGGCTCAACGTCTAC

ACGCCTGCCAATAAAAGTTCCAAGAAACTGCCGATAATATTCTTCATCCACCCGGGCGGCTTCTACTCCATGACCGGGCG

CAGTGACATCTTCGGGCCGCACTATCTCCTGGACCATAACATCGTGCTCATTACCATCAACTACCGCCTCGGCTCGCTCG

GTTTTCTCAGCACGGGCGACGCTGTGGCTCCCGGCAACAACGGCTTGAAAGACCAAGTATCGGCGCTGCGCTGGGTGCAG

CGCAATGCAGCGGCCTTCGGCGGAGACCCGGGACTAGTGACAATAGCGGGCGACAGCGCCGGCTCTACCAGCATCGGGCT

GCATATGGTCTCGACTATGTCTAAAGGTCTATTCCACCGCGGTATCGCAATGTCTGGATCCCCGTTTAGCAAAGGGCCAG

TGCTGCCACACCAGCTCGATCTGGGGCAAAAGCAGGCGCGCATCCTCAACTGCCCCACCAACTCCTCGCGCGCCATCGTC

GACTGCCTCAAGACCAAGACGTGGCAGGAGCTGGGGAACTCGCTGGATAAATTTTGGGATCAATTCAGCTTCGACCCCAT

CGGCTTGTGGACGGGTGTGGTGGAGCCGGACGTGGGGCAGCCGCGCTTCTTGGCCGAGCAGCCGGTCGCGGCGGTGCGCG

CGGGCCGCTTCACGGGCGTCCCGCTGCTCGTCAGCCAGACTGAAGACGAGCTCATCTGGAAGGCCGTTGGTGTGATCAGG

AACAAGACGCTCCTGGATCGCATGAACGCAGAGTGGGAGACGATTGCGACGCTCGCGTTCGAATTGCCCGCAACCACTGC

AGCAGCTGCCAGCCGCCGCCTCAAGGAGGCGTACCTGGGAGGGAAAGATTTGACGGCTACTGCTGAGAGCACGAATGGCC

TGGGGAAGCTGTATGGAGACGCCTGGCTTGGACTCCAGGCGCACAGGTTGGCCAACCTGATGTGCCGACGCTCGTCTAAG

CCCGTCTGGTACAGCGACTTCAGCTACGTCGGCAACAACAGCTACCACGAAGACCCAGCAACCGGAAAACCCATTGCAGG

TGCAGCGCATCAGGACGACCTGATCTACCTGTTCTCAATGAACTACTTCCACAAGCCGATCGCGGCGCAGTCGCCGCCGC

CGCCCGCGCCGCAAGACTCCGTCATCGTGGACCGCATGACC

>Cobl_CXE15.3

ATGAAGTCGCGGCGCGGACGCGGCTTCCAGTCGTACCGCGGCATCCGCTACGCGAAGCCGCCCACAGGGGAACGGAGGTT

CCAGCCGCCTGAAGACATGCTGAAGTATACCTCTGAGGTGGACGCGAGCGAAGATGGCCCCGCGTGCCCGCTGCCCGTCG

AGCCCGGCGAGTACTACGTCGACGAGGACTGTCTGCGGCTTAATGTCTACACGCCTGCCAATAACAGTTCCAAGNNNNNN

NNNNNNNNNNNNNNNNNNNNNNNNNNNNNNNNNNNNNNNNNNNNNNNNNNNNNNNNNNNNNNNNNNNNCTCGCTCGGCTT

CCTCAGCACGGGCGACGCAGTGGCTCCCGGCAACAACGGCTTCAAGGACCAAGTGTCGGCGCTGCGCTGGGTGCAGCGCA

ACGCAGCGGCCTTTGGCGGAGACCCGGGACTAGTGACGATCGCCGGCGACAGCGCTGGCTCCATCAGCGTCATGCTGCAC

ATGATCTCGACTATGTCTAAAGGTCTTTTTATCCGTGGTATCGCAATGTCTGCGTCTCCGATGGGCAAGGAGCCACTGCC

GTCGCACCAGTTCGAGCTAGCACAGAAGCAGGCGAGCATCCTCAACTGCCCGACCAACACCTCGCGCGCCATCGTCGATT

GCCTCAAGACTAAAACCTGGCAGGAGATGGGGAACTCGCTGGTTGGATTTTGGGATGATGGCTCAGACGCGGTCAGCTTC

TGGAAGCCGGTAGTTGAGCCGGACGTGGGTCAGCCGCGCTTCCTGGCCGTCCAGCCGGACGAGGCGGTGCGCTCACGCAG

CATCTACGCCGTGCCGCTGCTCATCAGCCAGACTGAGGGCGAGTTCTTCTATATGGCTTTCGGTGTGACCAGGAACAAGA

CGGTCCGGGACCGCGTGAACGCGGAGTGGGAGACTATTGCGCCGGTGTCGTTTGACCTGCCCAAAACCAACGCGGCCGCC

GCCAGCCGCCGCCTCAAGGAGGCGTACCTGGGAGGGAAGGATGTGGAGGATACTCCGGAGAGCGCAAAAGCCTTGGGAGA

GATTTATGGAGACAGTTGGATTGGATTCAACGTGCACAGGATGGCCAACCTGATGTGCCGGCACTCGCCTAAGCCTGTTT

GGTACAGCGAGTTCAGCTACGTCGGTAACAACAGCTATTACGAGGACCCACTCACCGGGAAACCCGCTGGCGCGGCNNNN

NNNNNNNNNNNNNNNNNNNNNNNNNNNNNNNNNNNNNNNNNNNNNNNNNNNNNNNNNNNNNNNNNNNNNNNNNNNNNNNN

NNNNNNNNNCGCTCTCCTGGCCGGCCATGAAGCCGGGAGACAGGAAGTACCTTCGCGTGGACAAGGAGTTCTCCGTCCAC

GAGAAGCTGCACGAGACGAGGCTGCAAGTGTGGGAGGAGCTGTACCCTATCGAGTATTAACCCTTTCGGCGCAAGGCCCC

TGCCGCGTTGCAACCATTCGCGTTGCAAACGGTTCAGGCGCAGTACAATTCGTCGCCTAG

>Cobl_CXE15.4

ATGGAGTCGCGGCGCGGGCGCAGCTTCCAGTCATACCGTGGCATCCCCTACGCGAAGCCGCCCACAGGGGAACTGAGGTT

CCAGCCGCCTGTAGACATGCTGAGGTATACTTCTGAGGTGGACGCGAGCGAGGATGGCCCCGCGTGCCCGCTGCCCGTCG

CGCCCGGCGAGTACTACGTCGACGAGGACTGTCTGCGGCTCAACGTCTACACGCCTGCCAATAACAGTACCAAAAAGCTG

CCGATAATATTCTTCATCCACCCGGGCGGCTTCTACTCCATGACCGGGCGCAGCGACCTCTTCGGGCCGCATTATCTCCT

GGACCATAACATCGTGCTCATTACCATCAACTACCGCCTCGCCTCGCTCGGTTTCCTCAGCACGGGCGACGCGGTGGCTC

CCGGCAACAACGGCTACAAGGACCAAGTATCGGCGCTGCGCTGGGTGCAGCGCAACGCAGCGGCCTTCGGCGGAGACCCG

GGACAAGTGACGATCGCGGGCTGCAGCGCCGGCTCCATCAGCGTTATGCTGCACATGATCTCGAATATGTCTAAAGGTCT

ATTCCACCGCGGTATCGCAATGTCTGCGTCCCCGATGTACAAGGAGCCGCTGGCATCCAACCAGTTCGATCTAGCACAGA

AGCAGGCGAGCATCCTTAACTGCCCGACCAACACCTCGCACGCCATCGTCGACTGCCTCAAGACTAAAACGTGGCAGGAG

ATAGGAAACTCACTGCCTGGATTTTGGGATCAAGGCGCAGGTTTACAGTCAGACGCAATCGGTTTCTGGAAGCCGGTGAT

CGAGCCGGACTTGGGGCAGCCGCGCTTTCTGACCGTCCAGCCGGACGAGGCGGTGCGCTCGCGCAGCATCCACGCCGTGC

CGCTGCTCATCAGCCAGACCGAAGACGAGTTCTACTGGAAGGCTTTCGGTGTGACCAGGAACAAGACGGTCCGGGACCGC

GTGAACGCGGAGTGGGAGACTGTTGCACCGGACTCGTTCGACATGCCCAAATTGCCCAAAGACAAAGCGTCCTTCGCCAG

CCGCCGCCTCAAGGAGACGTACCTGGGAGGAAAGGATGTGGAGGATACTCCGGAGAGCGCAAAAGCCTTGGGAAAAATTT

ATGGAGACAGTTGGATCGGATTTAACACGCACAGAATGGCCAACCTGATGTGCCGGCACTCGCCTAAGCCTGTTTGGTAC

AGCATGTTCAGCTACATTGGTAACAACAGCTACTTTGAGGACCCACTCACTGGGAAACCCGCTGGCGCGGCGCATCATGA

CGACCTGATCTACCTNTACTTCCGCAAGCCGATCGTGGCGCAGTCGCCGCCGCCGCCCGCGCCGCAAGACTCCGTCATCG

TGGACCGCATGACCGCCATCTGGTACACCTTCGCCAAATACGGAGACCCCAACCCCCGTAAAGGCGAGCTGCCGGAGCTG

GCGACGCTCAACTGGCCGGTCATGAAGCCGGAAGACAGGAAGTACCTTCGTGTAGACAAGGAGTTCTCCGTCCATGAAAA

AATGCACGAGAGTCAACTCCAAGTGTGGGAGGAGCTGTACCCTATCGTGTATTAG

>Cobl_CXE16.1

ATGGCGAAAGTCAAAGTAAATGACGGTGTTTTAGAAGGAGAAGTCTTAGATAATGTGCTCGGAGGAAAGTACTACAGTTT

CAAGGGTGTTCCTTACGCTGCTCCTCCGGTCGGAGACTATAGATTTAAGGCGCCTCAACCAGTTAAACCTTGGGATGGAG

TCCGCAGCGCCACTGCCCACGGCCCAATCTGCCACCAGTTCGACATGTTTACTCACACCATTGACATTGGCAGTGAGGAC

TGCCTGTACCTAAATGTGTACACCCCAACATTGACACCTAAAAAGCCTCTACCTGTCATGTTCTGGATACATGGTGGTGG

TTTCATGAGTGGCAGTGGCAACAGCAACACCTACGGACCGGACTTCCTCGTCGCCCAGGATGTGGTCCTGGTCACCATTA

ACTACAGGCTAGAGGTTCTCGGATTCCTCTGCCTGGATACAGAGGACGTTCCAGGAAATGCTGGAATGAAGGACCAAGTA

GCAGCCCTTAGATGGGTACAAAAAAATATAGGACATTTTGGTGGTGACGCTAACAATGTCACTATATTTGGTGAAAGTGC

CGGCGGAGCGTCGGTTGCTTACCACTTAGTATCGCCCATGACGAAAGGCCTATTTAAAAGGGGTATACTTCAGAGTGGTG

TGAATTTATGCCATTGGGCTCGGAACTTCGAACCGCACCTGAGAGCTGAAGCGATTGCGAGATCACTTGGCAAGGACACT

ACAGATGATAAAGAACTGTATGAGTTTTTCAAATCACAACCTATTGAAAGAATAGTAGCAATGGCCGTGCCACTAACAGT

GGAAGAAGAAGCTGAAATACGAATAAATATAACATTAAATGTAGCTGATGAGAAACAGTTTGGTGACAATGAGAGGTACT

TTTATGGTGATGTTTATGAACGGCTACGTAGCGGCGTTCACGAGGGCGTTGAAATCATGATAGGATATACTACCCATGAA

GGGACTATTCAATTCAATAATTTGCCAGAAGAGGGTACTTTCGATAAACAGAAAAAGTTTTTACAAAGCTTTGTTCCGAT

AGACTTGCTGCTGCAATGTTCAGTGAAAGACCAGCTGGCAATTGGCAAAAAGGTCAAAGAACACTATTTTGGAAACAAAC

AAGTGGGAACTGATACTGTTGACCCACTTATAAAATACTATACATTGCAGCTATTGGAGTATGATATTTTTCAATATGCC

AAAACCTGTGTACAGTCGAATGCCAATCCATTGTTCTTTTATCAGTTTGATGTTGAAACAGAAAGAAACATATATGCCGA

CATGTTTAAAGCCAGACCCTTAATAAAAGGGAAGAATGTCTGCCATTGCGACGATTTGATGTATTTGTTTGATTATGAAA

AGGTACCTGTAAACCTGAATTCTGAAAGCCACAAAATGATTAAAAATGTCACAAAATTATGGACTGACTTCGCCAAATTC

GGAGACCCGACTCCGGACGGTAAACTGCGCGTGCGCTGGCCGGCGTTCGACCTGCAGCGGCAGCAGTATCTCGTCATCGG

GAACGAGCTGACCGTCGCAGCATTCCCTGACAAGGAGGAGTTACAATTCTGGGAGGAACTCCACCAACAATACTTGAAGA

AATAG

>Cobl_CXE16.5

ATGGCGCAAGTCCGAGTTAGTGACGGTGTTTTGGGAGGAGAAATCCTGGATAATGTACTCGGGGGAAAGTACTACAGTTT

TAAAGGAGTGCCTTACGCTGCCCCTCCTGTCGGTGACCTGAGGTTTAAGGCGCCTCAACCAGTTGTACCTTGGGATGGAA

TCCGGAGCGCCAATGCCCATGGACCAATTTGCTGCCAGTACGATATATTTTTTAGCACTGCCGAAACCGGCAGTGAAGAC

TGCCTGTACCTAAACGTGTACACACCAACATTGAAACCTAAAAAACCCCTATCCGTCATGTTCTGGATACACGGTGGTGG

TTTCTTAAGTGGCAGTGGCAACAGCCAAATCTTTGGACCAGACTTTCTCGTAAAACAGAATGTAGTTCTTGTCACTATTA

ATTACAGACTAGAAGTTCTCGGATTCCTGTGTCTGGATACAGAAGACGTTCCAGGGAATGCTGGCATGAAAGATCAAGTG

GCTGCCCTTAGATGGGTACAAAAAAACATAGGACATTTTGGTGGTGATCCTAACAATGTTACTATTTTTGGAGAAAGTGC

TGGTGGGGCATGCGTTGCTTACCACCTGGTGTCGCCAATGACGAAGGGGCTATTTAAACGGGCTATACTTCAAAGTGGTC

AGAATTTAAGTCATTGGTCTCGGAACTTTGAACCGCGTTTAAGAGCTGAAGCGATTGCGCGATCACTCGGCAAAGACACT

ACTGATGATAAAGAACTGTATGAATTCTTCCAATCTCAACCAGTTAAAAACTTAGTAGCCATTACCGCACCACTGACAGT

TGAGGAAGAAGCTGGAATATTTATGCATTTAAAATTAAACGTCACTGATGAGAAACTGTTTGGTGACAATGAGAGATACT

TTTACGGTGACGTTTACGAAAGATTGCGTCACGGCATTCATGAGGGAATTGAAGTCATGATGGGATACACCACACACGAA

GGCCTGCTTAGATTCAATTATTTGAACAAAGAGGGCACTTACGATCAATTTAACAAGTACTTACAGTGTTTTGTGCCTGT

AGAGTTATTGTTGCAATGTTCTGTGAAAGACCAGCTGGCCATTGGCAAAAAAGTNGTCAAAGAATATTACTTCGGAAACA

AAAAAGTGTCTATTGATACTGTCGACTCGTTTATCAAATGCATTTCAATGCAGTTCTTACAGTATGATAGTTTGCAATAC

GCAAAAACATTCGTACAGACGAATCCTAATAGATTGTTTTTATATCAATTTGACATTGACACTGAGAGAAACAAATATGC

TGATCACTTTAAAGCCAGACCTTTTATAAAAGGGAAAAGTGTCTGTCACTGCGACGACTTGGTGTATTTGTTCAACGATG

AAGAAGTACCTATAAACCTCAATGCAGACAGTTACGAAATGATCAAAAATGTGACGAAATTGTGGACTGACTTCGCCAAA

TTCGGTAACCCGACTCCTGACGGTAGCCTAGGTGTGGATTGGCCGGCGTTTGACCTACAACATCAGAAGTTTCTCATCAT

CGGGAATCAATTGACAATCGCAGACTTCCCCGAAAAAGACGAGTTAGAATTCTGGGAGCAACTACACCAACAGTATCTAC

CTAAGTAG

>Cobl_CXE17.1

ATGAAGTATGCAAAGTACGTGGTACTATTTACGTTGTTCGCCTTGAACCTGGTGGACCAACCGGCGCCAGAGCTCGAGAT

TGAGCAAGGGAAATTGAGCGGTAAAGTCAGCGATGATGGGTCGTATTTTGAATACGTCGGGATACCATATGCTTCGACTA

ACCACAGCACGAGATTTCAGGCACCCGGTCCTCCGCCATCATGGGAAGGCGTGTTCAAAGGAACAGACCACATTTACATG

TGTCCTCAAAACTCCAGGACTGGGACAGTAGGCAGAGAAGACTGCCTCGCCATCAATGTTTACGTCCCGACCATGGCCAA

TGGCCCCTTACCCGTCATGGTCTACATTCATGGAGGAGCATTTGCCCTTGGAAGTGGAGGAAAATTCATGTACGGACCTG

ACTTCCTTGTGAAACATGATGTTATAGTAGTCACCTTCAACTATAGACTAGGAGCTCTCGGATTCATGTGCTTGGGAATC

AAAGAAGCACCAGGTAACGCTGGCCTTAAAGACCAATTGGCAGCTCTGAGATGGGTTAAGAAGAATATAGCTGCGTTTGG

TGGTGACCCTGAGAATATTACTGCTTTTGGCGAAAGCGCTGGAGCGGCGTCGCTGTCTTTGCTAGTGGCTAGTAATGTGG

CGGATGGCTTGTTTAACAGAGCTATTATTCAAAGCGGATCGTCTATAGGTAATTGGGCGATTAACAGGAAGCCAGTTTGG

GTTTATAGCCTTATTGCGAAGCAGCTAGGATATGAAACAGAAGATCCACATGAACTTTACAATATTTTTAAAGATCTACC

AGTCAAAGATTTCCTACGAACAAGACCAAGAAAGCCTCTTAGCAAGTTTTTTGATACCCAGTTATTGCATCTGCCTTGTG

TTGAGAAATCTTTCCCTGGTATAGAGCCCATTATCACAGAGTTACCGTATGACATTTTGCAAAGAAAAACTATAAATATA

CCGTTAATGTACGGTTCAAACAGCAAAGAAGGCTTATTCTTGATAAGTGAAGAGACGCCAGAAACAGTTGATGAAAGGAA

CGGCCGCTACCTGTTTGCCTCTGATTTAGAATTCAAAGATGAAGATACAGCAGACAGAGAAGCTGAAACAGTAAAAGAGT

TTTATTTCGGCAAAGAAACTATTGATATGACTAAAATACTTAACATGTCGGACCTTTATACACATTTGTACTTTGAAATG

CCGGCATTGATGGAAGCTGAAGTTGTAGTCAGTCGAAGTAATAAGCCCTTGTATAATTACTACTTCGACTATGCAGGAAG

CAGAAACGTACTGAAGTACATATCGGGGTATGGCGGAAGTGAAGGAGCATGTCACGGGGATGATTTGTTCTATTTGTTTA

AACAAGATTTTTTGCGATTCTGGATTAATAAAGAAGATAGGAGAATAATTGACAGCATGACTGAGATGTGGACCAATTTC

GCCAAATATGGGGTTCCAACACCGGAGTCCTCAACCAATCTACCTCGTTGGACCCCAAGCACAAAGGAGGAAACAAAGTT

ACTTTATATAGGAAAAGAGTTGAAAATGGGCCCCATACCCAATCCCAAAGCGTATGAAATGTGGAAACGGGTGTTTACCA

CGTATCGTAGGAAGTCTAAGTAA

>Cobl_CXE17.2

ATGAAGTATGCAAAGTACGTGGTACTATTTACGTTGTTCGCCTTGAACCTGGTGGACCAACCGGCGCCTGAGCTCGAAAT

TGAACAAGGCAAATTGAGCGGTAAAGTCAGTGACGATGGGTCGTATTTTGAATACGTCGGGATACCATATGCTTCGACTA

ACCACAGCACGAGATTTCAGGCACCCGGCCCACCACCATCATGGGAAGGCGTGTTCAAAGGAACAGACCACATTTACATG

TGTCCTCAAAACTCCAGGACAGGCACAGTAGGCAGAGAAGACTGCCTCACCATCAATGTTTACGTCCCAACCATGGCCAA

TGGCCCCTTACCCGTCATGGTCTACATTCATGGAGGAGCATTTGCCCTTGGAAGTGGAGGAAAATTCATGTACGGACCTG

ACTTCCTTGTGAAACATGATGTTATAGTAGTCACCTTCAACTATAGACTAGGAGCTCTCGGATTCATGTGCTTGGGAATC

AAAGAAGCACCAGGTAACGCTGGCCTTAAAGACCAATTGGCAGCTCTGAGATGGGTTAAGAAGAATATAGCTGCGTTTGG

TGGTGACCCTGAGAATATTACTGCTTTTGGCGAAAGCGCTGGAGCGGCGTCGCTGTCTTTGCTAGTGGCTAGTAATGTGG

CGGATGGCTTGTTTAACAGAGCTATTATTCAAAGCGGATCGTCTATAGGTAATTGGGCGATTAACAGGAAGCCAGTTTGG

GTTTATAGCCTTATTGCGAAGCAGCTAGGATATGAAACAGAAGATCCACATGAACTTTACAATATTTTTAAAGATCTACC

AGTCAAAGATTTCCTACGAACAAGACCAAGAAAGCCTCTTAGCAAGTTTTTTGATACCCAGTTATTGCATCTGCCTTGTG

TTGAGAAATCTTTCCCTGGTATAGAGCCCATTATCACAGAGTTACCGTATGACATTTTGCAAAGAAAAACTATAAATATA

CCGTTAATGTACGGTTCAAACAGCAAAGAAGGCTTATTCTTGATAAGTGAAGAGACGCCAGAAACAGTTGATGAAAGGAA

CGGCCGCTACCTGTTTGCCTCTGATTTAGAATTCAAAGATGAAGATACAGCAGACAGAGAAGCTGAAACAGTAAAAGAGT

TTTATTTCGGCAAAGAAACTATTGATATGACTAAAATACTTAACATGTCGGACCTTTATACACATTTGTACTTTGAAATG

CCGGCATTGATGGAAGCTGAAGTTGTAGTCAGTCGAAGTAATAAGCCCTTGTATAATTACTACTTCGACTATGCAGGAAG

CAGAAACGTACTGAAGTACATATCGGGGTATGGCGGAAGTGAAGGAGCATGTCACGGGGATGATTTGTTCTATTTGTTTA

AACAAGATTTTTTGCGATTCTGGATTAATAAAGAAGATAGGAGAATAATTGACAGCATGACTGAGATGTGGACCAATTTC

GCCAAATATGGGGTTCCAACACCGGAGTCCTCAACCAATCTACCTCGTTGGACCCCAAGCACAAAGGAGGAAACAAAGTT

ACTTTATATAGGAAAAGAGTTGAAAATGGGCCCCATACCCAATCCCAAAGCGTATGAAATGTGGAAACGGGTGTTTACCA

CGTATCGTAGGAAGTCTAAGTAA

>Cobl_CXE19

ATGGTGAACCGAGATCTAAGAGTGTGGGTGAAACAAGGGTGGCTAGAAGGAGAAAGAAAAGAGTTGATCACAGGAAATGG

GACCTACTACAGCTTCAAGGGTATACCGTACGCGGCGCCACCAATCGGGAGATGGCGATTTAAGGCACCTCAACCGCCTC

TCTCTTGGACAGGAATCAAGAAAGCAACTGAACACGGACCAGTATGTCCTCAATATGACTTGCTGAAGAACCAGATGTGC

CCAGGCATAAGTGAAGACTGCCTATACTTAAACGTTTACACTAGAAATATAAAACTAGAAAAACCTCTTGCAGTCATGGT

TTTCATTCATGGAGGAGGCTTCAAATTGGGCTCCGGAAACGAAGACAATTACGGTCCAGATTTCTTAGTTGAACAAGACG

TAGTCTTAGTAACGATTAACTATAGGCTAGATGTACTAGGATTTCTTTGTTTAGACACGAGAGATGTCCCAGGTAATGCA

GGAATGAAGGACCAAGTGGCAGCCTTGAAGTGGGTCAAAGATAATATCCGGCAATTTGGTGGCGATCCTCGTAACATTAC

AGTTTTTGGTGAAAGTGCTGGTGGAGCTTCTGTTTCGCTTCATGTCATATCACCACTAGCTAAAGGACTGTTCAATAGAG

CACTTACAATGAGCGGAACAGCGTTTAGTGAGTGGGCAATGACTGAAGAACCAGTAAAGCGAGCCTTTGCTCTCGGAAGA

CAATTAGGAATAGACACTGAAGACCCCGTTGAACTGCATAATTTCCTACAAAACGTTCCAGTAGAGAGCCTTGTAAACAC

TAAGATAGCCCTTAATGACTTTGAAGAAAAAACGAATTATATGTGGAGAGTAAATCAGTTTGGTCCAACTGTGGAAAAGG

CACTAGGACAAGAGTCATTCTTAGCTGAGGATCCAATGAGTCTAATGCTGAAGAGAGGTGTCGGCCAAGACGTAGATGTT

ATGATAGGTTATACAAACGAAGAAGGTATATTAGGCATATCAACGTTTGCAGAACTTTGGGTTAAATCATTCAGCGAATC

TCCAGAGCTTTTTCTGCATGCGCCCATTGTCACTAAAAGCAACAAAGTTCCAGAGCTGTCTCAAAGAGCTTTCGATCGCT

ACTTTGGAGACAAAGATTTAAATGAAAAAGGTGCCAGAGGAATGGTGACATACTTGTCAGAAAAGGCAATTTATGCGATT

TTACTGTACCTCAATCTTCTGGCGAAATCTGGAAAATCGAAGAGATATCTCTACCGATTTTGTTGCGTATCCGAAAGGAA

TATTTATGGCAATACTGGTCAGGAATACGGTATTAAAGGAGCTAGCCATATGGATGACATGGTGTATCTGTTTGATCCAA

AACAGATGAATTTACAATTGGACACAAGCAGCAAAGCTTATGAAATTATTAAACTGTCGTGTAAAGTTTTTACCAATTTT

GCAAAGTTTGGTAATCCAACTCCTGATTCTTCGCTCGGCATCAAATGGCCTATCTACAGCGACTACTCGAGGAGTCACGT

GGACATCGGCGAAGAACTGACCCTGGGTGCAAATCTCGACGAAGACGTTCTCGCCTTCTGGCGAAAGATATTCTTAGACG

CAGAATTGCCTAAATGA

>Cobl_CXE21

ATGAAGACTATTGTGCTGATATGTCTGGTGGTCGCGCGCGCGGCGTCCGCGCAGAGCGAGGCGCGCGAGTCGCGCACCGT

GCAGACCGCGCAGGGGCCCGTGCGCGGGTACAAGACTGACGACGTTTTTCAATTTCTTGGAGTACCTTACGCTACGGCGC

CGACTGGTGAAAACCGGTTCAAAGCACCACTTCCAGGACCTATTTGGATGAACCCTTTAGAAGCGGTTAATAATAAAATA

ATCTGTCCACAAGGCACAAGCGTATTTTTTAGCTTTTCTTCATTCTCTATGCAAGAAGACTGCCTTGTTGCAAATATTTA

TATGCCGGACACGGAAGATACTAACCTGCCAGTAATGGTTTATGTGCACGGGGGCGCGTATCAAATGGGATGGGGTGAAA

TGATCACACCAAAATCTTTGGTACAAAGCAAAAAAATTGTGGCGGTCTCTTTCAACTACCGACTCGGTATCCATGGTTTC

CTGTGTCTGGGAACAAATGACGCTCCCGGCAACGCGGGCATGAAGGACCAAGTCGCGCTGCTGCGCTGGGTGCAGAAGAA

CATTGCCAACTTTGGTGGAAACCCTAAAGAGGTCACAATTGTTGGCAGCAGTGCAGGATCATCTTCAGTTGATCTCTTAA

TGCTTTCCGAAACTACAAAAGGTCTCTACAGTAAGGTTATTCCTGAAAGTTGTGCTAGTGTTGGAGTTTGGAGTGTTCAG

CTCGATCCGGTGCAAAACGCAAAAGATTTCGCGGGAAAGAACAATTTTACTGAAGTAAACGATATCCATGCGTTAGAACA

ATTTTATAAAACTGCATCATTTGAACTACTTACCTCGGACATATTTTTAGGTCAAAAAGATTCCACTTTTGTATTCTCCC

CTTGCGTAGAAAACAACATCGGCGAAGAAACTATTCTTGAAGATAATCCAGTAAATATTCTGAAAGAAGGAAAATACAGA

AAAGTTCCTGTTCTCTATGGATTTGCAAATATGGAGGGTCTGTTTAGGATGGATAGATTTGGAGAATGGAAGGACCAAAT

GAACGAGCATTTCGCAGACTTTCTGCCAGCCGACTTAACGTTTAAATCTGAAATAGAACGTTCTGAAGTTGCACAAATAG

TCAAGGAATTTTATTTTGGTGATAAATCGGTGGGAGTAGAAACTGTTCAAGCTTACATCGACTATTTCAGTGATGTCATG

TTTGCTTACCCGCATCTTAGATCCACACAGCTACAAGTGGCAGCTGGTAGTGACTCCATATACTTGTATGAGTATTCCTA

TTATACACCCCCACCGCAATTGGAGGGTATGCCACCTGTACCAGAATTCATGAAACAAATCCGTGGAGCTGATCACTGCG

CTCAATCCAGGGCAGTCTACGACACTATAATGCTTCCGGGTGTTGAAATCGACAAAGAATACCAAAAGAACAGAGATACA

ACACAAGAAATATGGTTAAACTTTATGACAACCGGGAAACCAGTTCCTGAAGGTTCGAAGCTCCCAGCGTGGCCTCCAGT

CGGTGCTAATAGGTCCCCGTACATGGACCTCGGGGAGGAGATGACACTCAAGGGTCCCCTCCTGGAGAAACGAGCTCGCT

TCTGGGACAACATATATGAGCGATACTACAGGTCGCCAGTGGCGCCTGCGGCGCATTACCCAGCAAGAGATGAATTGTAA

>Cobl_CXE22.1

ATGAAGACTATTGTGCTGATATGTCTGGTGGTCGCGCGCGCGGCGTCCGCGCAGAGCGAGGCGCGCGAGTCGCGCACCGT

GCAGACCGCGCAGGGGCCCGTGCGCGGGTACAAGAATGACGAGATCTTCGAATTTTACGGAATTCCTTACGCCACCGCTC

CTACTGGAAAAGACCGGTTTAAAGCGCCGCTGCCAGGTCCTGTTTGGATGAGTCCTTTAGAAGCAGTCAATGATAAAATT

ATGTGTCTACAAGGCCAAATGCCAGGTTTTCCTATGAACGGATTAACCATGCAAGAAAACTGTCTGATCGCAAATATCTT

AATGCCTGATACTGAAGAAACAAATTTGGCAGTTGTTGTTTATGTCCACGGGGGTGGATATCAATTGGGAGCCGGAAATA

TGCTTCAACAGAAAAGTCTAGTAAAGAGTAAAAAAGTAGTAGCAGTATCCTTTAACTACCGCCTTGGTCTCCATGGGTTC

CTGTGCCTTGGCACAGAAGGTGCTCCTGGCAATGCGGGTATGAAAGATCAAGTCGCCCTTCTGCGATGGGTAAAGAAGAA

CATTGCCCACTTTGGCGGAAACCCCGACGACGTTACCATCGGCGGCTATAGCGCAGGGTCCTCGGCAGTGGACCTCCTCA

TGCTTTCTGAATCAACCAAAGGTCTTTTCAATAAGGTCATACCCGAAAGCGGTGCCAACGTTGCTGTTTGGAGTATTCAA

ATTGATCCTATACAAAACGCGAAAGAATTTGCTAGAACTAACAATTTCACAGCAGTAGACGACATCAACGCCTTGGAAGA

ATTTTACAAAACTGCGCCATTCGACTTACTTATGTCGGATGTATTTCTGTATAGACTGGACTCAACGTTTTTAGCTTCTC

CGTGTGTAGAGCGCAACATTGGTCAAGAAACGTTTTTAGAAGACAACCCAGTAAATATTCTGAAGCAAGGAAAATACAGA

AAAGTCCCTATCTTATATGGGTTTGCAAATATGGAGGGTCTATTTAGAATGGATAAATTTGAAGAATGGAAAGTCAAAAT

GAATGAACAATTTGCAGACTTCTTACCAGCTGATTTAACTTTTAAATCTGAAAGCGAACGTTCTAAAGTTGCACAAGAAA

TAAAAGAATTCTATTTTGGAGATAAACCCGTAGGAGAAGAAACTGTTCAAGGTTACATCGACTATTTTACTGACGTTATA

TTTGCTTATCCGCATCTCAGATCCGTACAATTACAAGTAGCAGCTGGCAGTGACTCCATATACTTGTATGAGCATTCTTT

CTATTCACCACCTCCTGAAATCGAAGGCATGCCGCCCATACCGGAATACGTAAAGAAAATTCGAGGAGCCAACCACTGTG

CTCAGTCATTTGCGGTACACGAGGTCACAATACCATTCATTGGCTTAGATAATACTACCGAGTACTTGAGATTCCAGGAA

AAGTTGCGAGAAATTTGGTTAAACTTCGTACTCACCGGAAAACCGGTTCCCGAAGGGTCAAAACTGCCGGCGTGGCCCCC

GGTCGGTGCCAACAGGTCCCCGTACATGGACCTCGGGGAGGAGATCAAACTCAAGGGTTCTCTCCTGGAGGAGCGAGCTC

GCTTCTGGGACGACATCTACGAGCGATACTATAGGTCGCCGGTGCCTCCGTCGCCTACTCAAACAAGAGACGAATTGTAA

>Cobl_CXE24_PDE

ATGTTAGCTGTCAAACTTCTTTTGTTTCTCGCGGTGGTGGCCTGTGCGCGGGCGCAGGCGTCCAAGCCGGTGGTGAGGGT

GACGCAGGGCATACTCCAAGGGTCCTGGAAGGTGTCGACTAAGGGAAGAACTTATGCCAGCTTTGAAGGGGTCCCGTATG

CGAGGCCGCCTGTCGGAAAATATAGGTTTAGGGAGCCACAGCACTTAAAGCCATGGGCAGGAGTGTGGGACGCGACCAAA

ACACTCCCCAACTGTATGCAGTACGAGCCATTCAATAAGAAGGTTGAAGGCTCAGAGAACTGTCTCTACGTGAACGTGCA

CACGCCAAAACTCAGCGCCGGCGCAGCCTTGCCCGTCGTGGCGTTCATCCATGGCGGCGCGTTCATGTACGGCGCTGGCT

CCCTGTACGACGCGGGACATCTCATGGACAGGGATGTGGTGGTGGTGACCATCAACTACAGATTGGGACCGCTGGGATTC

CTCAGCACAGGAGACGAGTTCGCCCCAGGCAACGCTGGCCTGAAGGACCAGTCCTTCGCTCTCCAGTGGATCCAGAGCAA

TATCATGATGTTCGGCGGAGACCCTGACAGTGTCACGCTGACGGGGTGCTCCGCTGGCGGCGCCAGTGTCAACTACCACT

ACCTGTCGCCGATGTCCAAAGGCAAGTTCGCCCGCGGCATCGCGTTCAGCGGCGCTGCTTTCGCCTCGTGGTCGCATGCG

GTCAAGCCGCTGCAGAATGCGAAGAGCTTAGCAGCCATCGTCGGGTGCCCCACCGGCGCCAGCAAAGAGCTGGTGGACTG

CCTCAAGTACCGACCGGCTGAGGTCGTGGTCGGCGCGCAGACTGAGTTGTTTGAGTTCCCCTACCTCGAAATGTTCACGC

CGTTCACGCCGACGGTGGAGCCAGCCGGTACCAGGGACGCATTCCTGACGCAGTACCCGTTCCACGTGGCTCAAGCTGGG

GGCATGCACAAGGTGCCCCTCATCACCTCGGTGACGTCTGAGGAGGGGCTGTACCCTGCTGCTGTCTACCAAACGTCACC

TGACATCCTGCCTTACCTGGACGCCAACTGGGAGCAACTGGCCAGCAACATCTTCGAGTACAACGACACTTTGGCCGTTA

ACCTGAGGGCCAATGTAGCGGCCAAGATCAAGCAGCACTACCTTGGGAACAAACCTGTCAGCCAGGAGACCTTCCCGCAG

TTGGTTCAGGCTCTCGGCGACCGTCTGTTTGGGGTAGACGTGGGCAAGCTGGCTGAGATCCACGCACGCAAGTCTGGCCA

GCCTACCTACTTGTACCGGTACTCGTTCCGCGGCGAGAAGAGTCTGTCCAACACGATGGCTCGCAACAACAACAACTATG

GTGTGAGCCACGCTGATGACGTTTTCCACATCTTCAAGTTCGACGTGCTGTCGTCCACGACGGCTGAGGATATGAAAATG

ACGGAAGCCCTTCTCGACATGATTTACAGCTATTCTACTACTGGCAACCCGAAGTTGACCAACGAGGCGCCAGCGTGGAC

GCCACTCACTCCTGGCTCCACCGAGCTGAATTACCTGGAGATAGCGTCGCCGAGCAGCATCGAGATGAGAACCAGCTCCG

ACTTCGGGCAGCGTAGCTTCTGGGGCAGCCTGGGGTTGGTCGAGAACGAGAATTACCGGGTTAGGGACGAGCTTTGA

>Cobl_CXE25

ATGAAGTGGGTGGCGCTGGTCTCCCTGATCCTGGCTACGGTGCTGCAGCAGCCCACGCCGCTGGTGCGCACGCGCGGCGG

CCTGGTGCGCGGCCGCGTCTCCCACAACGGCCGGTTCCATGAGTACCTGGGGATCCCCTATGGGACCGTCGGCAAGGAGA

ACCGGTTTCAGGCGCCGCTACCCCCACCGAAATGGGAGGGGGTGTTCGAAGCCGTCAACACCAACACGAGATGCCCGCAG

ACCCTGCTCGGCTCCGTGGGCATCATCGGCCAGCCCGACTGCCTCAAGCTGAACGTCTACACCCCCGTCCGCACCAGGAC

GGACAAACTCCTGCCTGTCATGGTCTACATTCACGGGGGCTGCTTCTTCGAAGGCACGGGATCTGCCTTCCTGTACGGAG

CCGACTACTTCGTTGAACATGGAGTTGTATTCGTTGGAATCAACTACAGATTAAACGTGGAAGGCTTCCTGTGCTTAGGC

ATTAAAGAAGCACCAGGTAATGCTGGCTTGAAAGACCAAGTAGCAGCGCTGAGGTGGATCAAGGATAACATAAGCGCTTT

TGGAGGAAACCCAGACAGCGTGACGTTATTTGGTGAAAGCGCTGGAGCTGTGTCGTCCTCGTTCATGGTGCTCTCACCAG

CAGCCAGAGGACTGTTCCATAGAGTGATCCTTCAAAGCGGCTCCTCGCTGGCCCCATGGGGACTGCAACACGACCCAATA

AGAACAGCTCATGCTCTAGTCAAACAATTCGGCTACGATACCAAAGACCCCTACGAAATATACCACATCCTATCAAATAA

AACTGTAAACGAATTAATTAAAGCTAAAGCTTTTGATAATAAGAACTACATCACATCTGACCGGCTGTTCGTTCCGTGCA

TCGAAAAAGACTTACCTGACGTCGAAGCTATCGTCACCGAGTACCCCACGAGCATAATAGAGTCTGGTAACTACACTAAA

GTTCCCATGATAATTGGTTTTAACGACAACGAAGGCATTTACTTCGTCGCTAAAGATTATGGCACTAGTCTGAAATCGAT

CGATCCCAAAGAAAACCTGACGCCTGACCTGGAGTTCCCTACTGAATTCGACATGAACAACACGGTGGAAAGTATAAGGA

GTCATTATTTTCCGCCGGATAAGGAGGAGCTGATTCAGGACATGGTGGACCTGTACTCTGATCTCCACTTTAAGTATCCG

TCTGTGGTGGAGTCGGACCTGTACGCGAAGACGTCGGGGAAGCCGATATACTTCTACCTCTTCAAGTACGGCGGCTACAG

GAACATGGCTAAGATGATCTCGCGGTTCGGGGCGACGGGCGGCGCGTCGCACGCCGACGAACTGTTCTACCTCTTCAAGC

CGCACTCCATCCCGTTCCCGCACTACTTGGAGAGGCGCATGATCAAGCGCATGGTGACGCTGTGGACGAACTTTGCCAAG

TACAGCGACCCAACTCCGCAGACGTCGCCGCTGCTGCCGCTGCGCTGGCGGCCCGGCCGCGCCGGCAACCCCGCTGCGCT

GGTGATCGACAGCCAGATGTCCACAGCGAGACTCTGGGACCACACTGCCGTCGAGCTCTGGAACCACACCTACACTAGAT

ACAGGAGAAAGCGGTATGGGTTCCACTGA

>Cobl_CXE26.1

GGACCCGAACCAAAATGGGACGGAATCTTCGAGGCATAYAAYGAAAACGTCAAATGCTCGCAACGTTYCAACTYGTTCGT

YAKCGGTCAAGAAGACTGCTTAGTAGTCAACGTGTACGCCCCCCTCGCCCCCGCGCCCCCGCTGCCCGTGCTCGTCTTCA

TCCACGGCGGCGGCTTCCGCGACGGGTCGGGGTCCCCCTTTCTCTACGGACCTGAATACTTAGTCAAAAAAGGAGTTATA

CTAGTCACCTTTAAYTATAGATTRGAAATCATGGKATTCTTATGTCTGGGCACTAAAGATGCWCCAGGAAAYGCGGGTAT

GAAGGACCAGGTGGCAGCTTTGCGATGGGTGAAAAGAAACATAAGAGCGTTCGGTGGTGACCCCGACAATGTAACAGTTT

TTGGTGAGAGTGCCGGCGCGGCGTCCGTGTCCTATCATCTTATTTCACCAATGTCTAAGGGACTATTTAATAAAGCCATC

ATGCAAAGTGGCACAGCCATGGCGCCGTGGAGTCTTCAGTTTGAACCCGCAAAGATCGCCAAAGCGTTAGTAAAGAACAT

GGGCTACAAGACCGAAGATATAGATGAGATTTATAAAATCTTGATGACAAATTCAGCTAAAGAATTAATAAGTACACGAG

TACCAAGAGATGAAGGTAATCAATATCAGTCTGAAATCATTTTTGTTCCCTGTGTAGAAAGTAAAGTGTCAGGAGTCGAA

CAATTCTTGCCAGATACTCCTTATAATCTTATATCAAATGGACAGTATCTTAAAATACCGATGATAATTGGTTACAACAA

CGCAGAAGGATACGTCTTTGCTGGCAAAGAAAACGATACAACGATATCTAAAATAGATTTCTCGAAATCCTTGCCAAGGG

ATCTGATCATACATAACGAAGAAGATAAATTGGAAGTAGCTCGTAAGTTACGGGATATATACATGGGTGAGAAAGATATA

TCGAAGGAGACGTTAGAGCAATTCTCTTTTTTCGAAGGTGACTCGACAATCACATACCCTGTTCTAGCTACCATAGACCT

GTTTCTTAAGACCAATAAAAAACCTATATTTACGTATAGATTAGATTATGATGGCTGGTTRAATGTGGYCAARMTGSTRT

CTGGTTTCAWGAGKGCKCCAGGGGCGACGCATGCCGACGATCTKTTCTACATGTTYAAGCCYTYCTTTCCRACGCTTCAT

ATGTTGGAACGAGATATRGTGGATAAARTCACCAGCATGTGGACGAATTTTGCTAAATTTGGGGACCCCACACCGGAAGC

TTCGAAATGGCTTCAAAGATGGCTGCCAACTGACAGCGCAGACCCTCACGTCTTCGTCATAGACAAGAAATTTCACACAC

AACCGCTATGGAAAGACGAAACAATGCGGTTTTGGAACCAAACGTACTCAAAATTTAGAAGAAAAAACAGTAACTTTTCA

GTAAAAGTTAATCCGGACTGA

>Cobl_CXE26.2

ACGCCGGAAAACCGTTTTAAGGGTCCAGGACCCGAACCAAAATGGGACGGAATCTTCGAGGCATATAATGAAAACGTGAA

ATGCTCGCAACGTTTCAACTCGTTCGTTACCGGCCAAGAAGACTGCTTAGTTGTGAACGTGTACACCCCCCTCGCCCCCG

CGCCCCCGCTGCCCGTGCTCGTCTTCATCCACGGAGGCGGCTTCCGCGACGGGTCGGGGTCCCCCTTTCTCTACGGACCT

GAATACTTAGTCAAAAAAGGAGTTATACTAGTCACCTTTAATTATAGATTAGAAATCATGGGATTCTTATGTCTGGGCAC

TAAAGATGCTCCAGGAAATGCGGGTATGAAGGACCAGGTGGCAGCTTTGCGATGGGTTAAAAGAAACATAAGAGCGTTCG

GTGGTGATCCCGACAATGTAACAGTTTTTGGTGAGAGCGCCGGCGCGGCATCCGTGTCCTACCATCTTATCTCACCAATG

TCAAAGGGACTATTCAATAAAGCCATCATGCAAAGTGGCACGGCAATGGCGCCGTGGAGTTTTCAGTTTGAACCCACAAA

GATCGCCAAAGCGTTAGTAAATACCATGGGCTACAAGACTGAAGATATAGATCAGATTTATAAAATCTTGATGGCAAAAT

CAGCTACAGAGTTATTGAAGACACGAGTACCTAGGAATGAAGGTGATGTTGTTCAGTCTGAAAACATTTTTGTTCCCTGT

GTAGAAAGAAACATACCCGGAGTCGAACAATTCCTGCCAGATACTCCTTATAATCTGATATCAAATGGACAGTATCTCAA

AATACCGATGATAATCGGTCACAACAACGCAGAAGGGTACTATTTTGCTGGTAGAGAAAACGATACAACGATATCAAAAC

TAGATTTTTTGACAGCTTTGCCAAGGGATCTGATCATACCTAACGAAGAAGATAAATTGGAAGTAGCTCTGAAGTTACGG

GATCTATACATGGGTCAGAAGGATATATCAAAAGAGACGTTAGAGCAATTCTCTTTTTTTGAAGGTGACTTGGGGATCAC

GTATCCAGTTCTAGCTACAACAGACCTGTTTCTTCAGACCAGTAAAACACCTGTTTTTACGTATAGATTAGATTATGATG

GCTGGTTAAATGTGGTCAAAATGGTGTCTGGTTTCACGAGTGCTCCAGGGGCGACGCATGCCGACGATCTTTTCTACATG

TTCAAGCCTTCCTTTCCAACGCTTCATATGTTGGAACGAGATATAGTGGATAAAATCACCAGCATGTGGACGAATTTTGC

TAAATTTGGGGACCCCACACCGGAAGCTTCGAAATGGCTTCAAAGATGGCTGCCAACTGACAGCGCAGACCCTCACGTCT

TCGTCATAGACAAGAAATTTCACACACAACCGCTGTGGAAAGACGAAACAATGCGGTTTTGGAACCAAACGTACTCAAAA

TTTAGAAGAAAAAACAGTAACTTTTCAGTAAAAGTTAATCCGGACTGA

>Cobl_CXE27

ATGATCTTTATACACGGTGGCGCTTTTTTTCTAGGCGATGGCGGAAAAATATTCTTCGGACCACAATTCTTACTTAACCA

TGACGTCATTCTAGTCACTTTTAATTACAGACTTGGAATCTTAGGCTTCTTGTGTTTAGGTATTGAGGAAGCACCGGGAA

ATGCTGGTCTTAAAGACCAGCTAGCAGCTTTGAGGTGGGTCAAGAAGAATATAGCAGCATTTGGAGGAGATAGCAATAGG

ATTACCGTTTTTGGAGTCAGTGCTGGTGCAGCGTCGGTATCATTACTTATTGCAAGTGGGAAAATAGATGGGGTGGTTAA

TAGAGCTATAATACAAAGTGGTTCTGCTGTAGCTCATTGGTCAATCAGCAGGAAGCCGGTGTGGGTTGCAAGCTATGTTC

TCAAATCGTTAGGCTATGATACCGAAGATCCGTATGAACTATATAACATTTTGTCCAAAATGTCTGCGAGAGAATTAGCA

GCATTGCCGCATGAGAAACCACTCGAAATATATCTCGATACCCAGCTGATACACTTACCATGTATTGAGAAATCGATTCC

CGGGACAGAATCACTAATAACTGATTATCCTTTTGATCTTTTCAAAAAGAATCCTACTAACATATCCATTGTTCATGGTA

CTACTAACAAGGAAGGACTATTTTTTGTAGCATATGACCAAGAAGAGGGAATTAACTACAGAGATGGAAACTATTTATTT

GGGTCCGATTTAGAATTCGGAACCAGTGAAGAGGCTGTAAACGTGTCCAAAGGTATACATGAATTTTATTTTGGAAAGAG

ACGTATTAATACAGAAAGTATTATGGAACTGTCCGAGTTGTACAAACACTTACACTTTGAGATACCAAGCTTAATTGAAT

CGGAGATCATAGCAGAAGTCAGTAGCGCTCCGCTGTACAATTATTATTTCAAGTATTTTGGTAACAGGAACTACTTGAAA

CTCTCTTTAGGGATTGACAAGTCAGAAGGCGCGTGTCATGGAGACGAACTACTGTACCTTTTTGATGCAGTATTTTGGCC

TTTTAAAATCAATGACAAAGATGACCTTATGATTAGAAGAATAACTCAAATGTGGACAAATTTTGCAAAATACGGGGACC

CAACCCCGGAAATAACAAATGATCTCCCAGTAAAATGGCTGCCAAGTTCAAAGGACAAGCTGAAATTCTTCCACATAGGA

AACAACATGAGTATGGAAGACATGCCCAGCCCAGAAGCATACAAAATGTGGAAGGATATCTACACAAAATATCGTAAAAC

TAGGTTATAA

>Cobl_CXE28

ATGCCCCCAAAGAGGCATCGGCGAAGAGGACTGCCTCGTCGTGAACGTCTTCACCCCCACCACAATAGTCAACAGCCGAG

CCCCTGTGCTGGTTTACCTCCACGGAGGATCCTTCATCATGGGAGCAGCGCAAACCCAAGGAGTCGGGCCTTTGATCAAG

CAGAACATCATAGTCGTCACGGTCAACTACAGGCTCGGGGCACTGGGCTTCCTCTGCCTGGGAATAGANGGCTGAAAGAC

CAGAGCGCAGCGCTCCTATGGGTTCAGAAGAACATAGGAAATTTTGGAGGAGATCCTGCCCAGGTGACTTTGTATGGCAT

GAGTGCTGGAGCGGCGGCGGTAGAGCTCCACGTCCTATCGAAAACAAGCAAGGGATTGTTTCAGAGAGCAATCGTGGAGT

CGGCGTCTGCCTTGTCTGTCTGGTCTGTCGACGCTGAGCCGATACAAACCGCCTTGACCTTCGCGGAATCTTTACACCTT

CCCTCACATATAACCAAACGTCTGTACAGTTTGATCAGCTACCTCCAACACGTGCCCGCTGATACACTAAGCACATCCAA

CCATGAGTATTATAGCAACTTTACCGACATGACGTTCGGTTTCGCACCGTGTATAGAGAAGAAGTTCTACGGCACCAAAC

CATTCATATCGAAGTCCCCTTTGGAGATATTAACCAAACAGAACTATTCCAAGGTTCCGATCATGTTCCTCTATTCGGCT

TTAGAAGGCCTGTTCCTCAAAAGTGAAGAGTATTACACGCAAGATTACAAGGACAGAATGGAGACGAATTTTCTTGAGTT

CATGCCATCTGATCTTGTCTTTGACACGGAAGAGATAAAGCAAGAAGTAGGCAAGAATATAAAGAGTTTCTACTTCGGCA

ATAAGACGGTCGGAGAGAACAGTGTAATTAACTATTTGAACTACTTTGGGGACGGGGCTGTAATGGACGCGATGCTCAAT

TCTGCTGAGTTGCACGCTAGAGGCGGGAACCCGGTGTACTTGATGGAATTTGCATATAAAGGGAACATGGGAAGCTATGA

GAAGTTTTATGAGAACCTGACGTTGGCGGGGCATGGTGACGTGGTGAAGCATGCGATCTTGGAGAAGCCTGTGAGAAGCC

CGGCCGATAGACTAGCGGTGGACCGTGTCGCAAAGTTGATTGCAAACTACGTCAAGTTTGGTAATCCCACACCAGCCCCA

ACAGAGCTCCTCCCAACCCACTGGCCCACAGTGAAACCCGGCAACGTGAGCTGTCTCTACATGGACCAGGCCTTCACAGT

GCACGAGGAGCCCCACTGGGAGCAGCGGCGGTTCTGGGACAGCCTGTACAGC

>Cobl_CXE29

CGGCTGGTGGGCACGGCGCAGGGGCCGGTGCGCGGGTACCGCGCGGCGCAGGGAGACCACTTCGAGTTCCAGGGCATCCC

TTACGCTACTGTGCCTGCCGGATCTAGCAGGTTCATGGCACCTCTCCCTGGACCAGTTTGGCTGCAGCCTCTGGAAGCCG

TCAACGAAACTACGATATGCCCTCAATTCAACGCTATACCTCGGATGGCAACAGACAAATTTATCAGAGAAGATTGCTTA

ATTGCAAATGTTTATGTTCCTGATACAGAAGAAAAAAACCTTCCTGTAGTCGTCATAGTGCACGGAGGTGCTTACCAAGT

CGGGTTCGGTAATATGATGAAGCCACAAGGCTTAATGAAAAGTAAAAAAGTGATTGCGGTAACATTTAACTATCGTCTAG

GAGTTCATGGCTTCCTGTGCCTGGGCACAGAGGGGGCTCCAGGCAATGCCGGTATGAAAGATCAGGTTGCCCTGCTGCGA

TGGGTGAAGAAGAATATTAGGAAATTCGGTGGCAACCCCGACGAAGTCACAATCGCTGGCTACAGCGCTGGCAGTTCATC

TGTGGATCTTTTAATGTTATCGAAAATGACAAAAGGTCTCTTTAACAAAGTCATTCCGGAAAGTGGTGCCAATGTGGGAG

TGTGGAGCGTGCAGATTGATCCGCTTGAAAACGCAAAGGCATTCGCTAAGCAATTTGGCTTCGAGGACGTGGACGACGTC

TACGCCTTGGAAGAATTTTACAAGAGCGCTCCCTTGGAGTCTTTGGCCGCGGCCATGTTTACAGATAGGAAAGATTCCAG

TTTTGTTTTTTCTCCGTGTGTTGAACGGAATACCGGGGTTGATAAATTTCTGGATGATACGCCAGTAAATATTATAAAGC

AGGGTAAATACAGCAAAGTCCCCGTCTTGTACGGCTTTTCAAATATGGAAGGTATTTTCCGTTTACCGGAATTCGAAAGG

TGGAAGGATGAAATGAACACAAATTTCTCTTTGTTTTTACCGCTGGATCTCGAGTTTCAAAATACGGAAGAGAGAGACGA

AGTTGCCAATTCAATAAAGGAGTTTTATTTCGGTAGCAAGCCGGTCGGGGAGGACACGGTTTTGGATTACATTGATTACT

TTTCGGACGTCATTTTTGCTTATCCGACGTTGCGCTCGACGCAGTTGCAAGTGGAAGCAGGAAGCAAGTCAATATTCTTG

TATGAATATTCTTTTCCTATTAGTATATTTACAAGTTACTTCTTCAACCCCGACCCGGTGCCCGAGTACCTGCGGCGCGT

GCGCGGCGCGCGGCACGTGGCGCAGACCGACGCCGTGCTCGACTCGATGCTGGCTGCGCCGGCGCTGCCCGACGGCACTA

CAGCCGAAGACTACGAAAAGCATAAAGAAGTAATGAAAGAATTGTGGATCAATTTTATAACTACTGGCAAGCCTAGCAGC

ACCGACGCAAGCGCC

>Cobl_CXE30

TTCATCATGTCGGGCCAAGTAGACGTCGCCCAAGGCACTCTTCAAGGAAAACTGTGCACTTACAAAGAGAAAACATACTA

TAGTTTTGAAGGCATACCATATGCTAAGCCGCCCGTTGGAAAGTTGAGATTCAGAGAACCACAAGAGCCTGAAAGTTGGA

CAGGAGTTCGCAATGCCACAAAACCAGGAAACAAATGTGTGCAAATCAATCCAGCAACTATGACTGACATAATTGGCTCC

GAAGACTGCTTATACCTCAATGTTTACACACCTAACTTGCCAAAAGAGCAGCTCAAGAAACTTCCTGTAGTATTTTTTGT

CCATGGTGGCAAACTAATATTCGGCTATGGAGACTACTATAGACCAGACTATTTCATTGAACAAGATGTTATTCTTGTGA

CAATGAATTATAGATTACATGTGCTTGGTTTTCTATGTCTTAATGAAGTTGAAGTAGCAGGAAATATGGGACTTAAAGAT

TCTGTCATGGCATTAAACTGGGTTAAAAGTAACATAGAAAAATTTAATGGTGACAGTAATAACATAACTGTATTTGGAGA

GAGTGCCGGTGGGGCGGTCGTGACATCATACTTAACATCCAAAATGGCGACTGGTTTCAACAGAGTTGTTGCAATGTCGG

GCTGCTGCGTGTCAGACTTGTATTTGATCGACGATGATCCTATTTTTAAAGCTAGACATTTAGCTTCCGTTTTAGGAAAA

GAATTCCGTGATCCCAAAAGTTTACATGAATTCCTGGTAAATGTTCCAATAGAGGAGTTAATATTTGGATTGGGCGTTGT

GGAATCGTCTAGGCCCCCATCAATAATAAATGCATATTTTCTCCCCGTCATAGAAAAGAAATTCGATGGTATGCAGCGCT

TCTTTGATGAACATCCTATTGTCACATTACGTGAGAATCGGTACAAAAAAGTACCTTTGCTGTTTGGCGTAAATAGCCAC

GAAGGGGCATTTTTTGCACAAAAAGATAAAAAAGGACGCATAGTGTTCGAGAAGGACTTACTGTACTTTGTACCACGCTT

TTTGTCAGTGAATTCATACAGTCCTGAAGCTATTAAAATTGCAAATAATTTGAGAGATTTCTACTTCAATGGTGAAGCAA

TAGACGAAACTAAAATGTTGGAATACATTGAATTTGTTTCAGATGCTTATTTTAATAGAGATATTGTGACTTTTTTGGAT

GCTTTCGGAAAATATCATAATGAAGTATATACGTTTCGGTTATCATATTCAAGTAACATGAATACTAGGATTATGAAAAA

TCTTGGTCTGAAAGGCACGACACACGGGGATTTGCTGCAATATATTTTTTACAGAAAGAGGAAAGCTGACAGTGCTAACG

ATAAAGATAGAGAAATAATGAACATTTTGAACGAGACTGTCTGCAACTTTGCGAAAACTAGTATTCCAACATGGTCGACG

AAAGCTGTAAAATGGGAACCATACTCCTCTAATAAACGCAGCTTCCTCGAAATTAACGAAGAACAAAAACTTGTGCACAA

TTTCAACAACAATAATCATAAAGTTTGGCTGAAAATGGGAGAGAGGTCTAAACTGTGA

>Cobl_CXE31

ATGGTCTTCATCCATGGAGGAGCATTCATGGAAGGATCAGGTTCACCCGGCCTATACGGCCCGAAATATATAACCTCAAA

AGGGGTCATCCTTGTCACTATAAATTACCGACTCAACGTTCATGGGTTTCTCTGTTTACGTATAAAAGAAGCTCCGGGAA

ATGCGGGTGCTAAAGACCAAGTGGCTGCGCTGAGGTGGCTCCAGAAGAATATCAGGGTGTTCGGAGGAGATCCCGATAAT

GTGACGCTGTTCGGTGAGAGCGCCGGCGGGGCATCCGTGTCCCATCACCTATTGTCCAGAATGTCTAAGGGTTTGTTCCA

TAGAGCGATCATGCAGAGTGGATCAGCTCTAGCTTCTTGGGGACATCAGTCAGATCCGGTGAGGGTCACCACCTCTGTTG

CAAAGACCATGGGTTACAAAGTTTACGACCCGCTCGAAATTTACAAACTGCTCATGAGTATGACCGAAGTAGAACTTATT

ACCCCCAGGATACCTCGTGAGCTCGGTGGTACCATCATGTCTGAAATGCTAGGTTCTCCGTGCGTCGAAAAGATAATAGA

TGGCGTGGAACCATTTTTAACAGAAGACCCTTACACGCTGCTGGCGAAAGGAGAATTTACAAAGATGCCTGTAATGATAG

GCACAAACAGCCAAGAAGGGTATTTCTTAGTCAGTTACGAGAACAGCACAACGTTATCACGGATGAATTTTGAGAGTGCT

CTGCCCAGTAATCTAGAGTACCCTACAGAACAGGAACGAAAAGAAGTAGCGTGTAAAGTTAAGGAAATGTACATGGGTAA

CGATACGATTTCTAAAGATAGTCTTGAGAAATTGTCTAAGTTTCATGGCGAGCCGTTCTTCAAGTATCCTGCGGTAGCTG

AGACGGAGTTGATCTTGGAAAATAGTGATCAGCCGGTGTATAACTACGTGTTTAACTATGATGGTCGAAAGAGCATAGCT

AAGTTGTACGCTGGGTTTTGGGGGTTGCCTGGCGCGTCGCACGGCGATGAGTTGTTCTACTTGTTCGAGCAACACAGCGT

GCCCAACATGTTCGAGTGGAGGATGATGGACAAGGTCACGACCTTGTGGACAAACTTCGCCAAGTTCGGAGACCCAACCC

CAGAGGTGTCAGAACTGCTACCAACGAGATGGCAGCGGACCAACAAAACCAGTCCTCAAGCATTTGTCATAGATCGAGAA

CTCTCCACTGCCCCTCTGTGGTCCAGTAGCTCGCTCAACTACTGGCACGAGGTGTATACTAAATATAGGAAAATTTCATC

GCAATGA

>Cobl_CXE32

GAGGAGCACAAAGGAGGTGCTCTGCCGGCGCGACACCGACATGATCAGTCGGTGCTCATTTTCCTAGAGTCGGACCGACG

TTCTAAAAGTACGAGAGCCGTGCGTTTGTTTCCACTTGGTTTTTTGCTTTCGAGGCAGCACGATCATCTCGCAACCATGC

CAAACCAGTTTACATTATTTAGTTATTTTAGTGTGGGTCGTAATAAATCATTATCGGTGTTGATATTTTGCATGATTGCG

ACTGCATTTGTCGGTGCCGGTGCTGATAGTCCCACAGTCGAGACTCCTCTCGGTGCAGTGAGCGGACAATACCTTAAGAC

ACGAGAGGGAAGAACTATTTCGGCGTTCACGTCTGTCCCGTACGCCGTACCGCCTGTCGGAGATTTGAGATTTAAACCTG

TGGTACCAGTTGAGAAATGGGACGGAACATTAGACGCGACGCAAACGAGCCCTGCCTGCGTGCAGAGGAACCCCTACACC

CGTCAGCAAGAGATAGTGGGGCAAGAAGACTGCTTATACGTCAACATATATACCCCGTACACCAGTGAAGATCTCGCCGG

GAGCCACAAGCCACTGCCAGTGATGGTCTTCATCCATGGAGGCGGCTGGATGTGTGGAGACTCTTCCACGGACATGTACG

GGCCAAAGCACCTTTTAGACAGAGACGTGCTGTTGGTGGGGATCAATTACAGACTGGGGCCCCTGGGCTTCCTGTCCACG

CAAGACGCCCTCTGTCCCGGCAACAACGGGCTCAAGGACCAACAAGAAGCATTTAGGTTCATCCAAAAGGTCATTTCCAG

CTTTGGAGGAGATAAAGACTCCGTAACAATTTTCGGGGAGAGCGCTGGTGGCGCTAGCGCCAGTTTCCACATGTTGTCTC

CAACCAGTAGAGGGCTGTTTCACAAAGTGATAGCGGAGTCAGGAGTGTCGCTGGTGCCGTGGGCGGAGGCCCCGCCCGGC

GAGGCGCTCAAACAGGCCTTCCGTCTGGCTAAGTTCTTGGACTGCCCGCAGGCGCCTTCCGAAAAAATGCTCCAGTGCCT

TCGCACCAAGGACAGCTATGATGTTATCAACACTGAATTCCAGTTTTACGGTTGGGACTATGAGCCTATGACTCCGTTTA

AGGCAGTGGTTGAGCCAGACCTCCCCGGAGCCTTTTTGACTGCCGCGGCGAGAACGATTCGTGACATCGCTGACGTGCCA

ATGATGACGGGTCTGAACAATGCGGAAGGATGCCTAAAATCTGTCTGGATAACAGCCAATGAGTCAAGATACAAAGAATT

TAAGTCTGGATTCGACACAATAGCACCCATCACGTTCTATTACGAAAATTCGCCATTTGCTGATGAGATTACCAAGGAAG

TCAAATCATTTTATTTAGACGGAAAAGACGAAGAGGGATACAAACAAGGCATTCTCGATATATACACGGACTCCTACTTT

GCGTATCCCGCAATAGAGTCCCTTGAACACACGATGAGTTACTCCAAAAGCCCGGTATACCTGTACGAGTTGTCGTACAG

GGGGAATAACAGCTTTTCTCAGATATTCGGCGATACGACTGGGGATTACGGTGTATGCCACGCTGATGAACTAATGTATT

TGTTCCCGGTAGGATTTCTTCTAAATGAACCTTCAAAAGAAGATGTGATAATAAGTAAACTCATTGTTACTTTGTGGACA

AATTTTGCCACCACCGGGAACCCAAGTAAACCAAATGAAGTGCCCTTCAAGTGGGCGCCGGCGACTAGCTCGAAGACTCT

AGAATACCTGGACATAAGCGAGCAGCCGACTATGAAGAAGCACCTTTCGGAGCGCGCGCGCTTCTGGGGTACGCTGCCAC

TGTGGCATAGCCTGCGCACGCACAGACTCGCCGACGAGCTG

>Cobl_CXE33

ATGATATCAGCGGTCAATGAGTTCCTTGATGACTTGCGGGGTGGCAAAATGACCGAAGCGCCGATAGTCCAGGTGGAACA

GGGCCAGCTCCAGGGTCGCGTGGTGACCAGCCCTGCCGGGAAGTCATTCTACAGCTTCCAGGGCATCCCTTACGCCAAAC

CACCCCTGGGCTCCTTGAGGTTTCAGGCACCTCACCCCCCAGAAGCATGGGAGGGAGTGCGCGACGCAACGGCAGAGGGC

AACATCAGCGCCCAAATCAACTCCTTCGGCGCCAAAATCTACGAGGGCGACGAAAACTGCTTGTTCCTCAACGTCTACAC

CCCAAATATAGATGGAGAATTTCTCCCAGTCATGGTCTACATCCACGGAGGCGGCTTGCAATTCGGCTCAGGCAATTCTA

GCTCTTACGGCGCTGATTTCCTAGTCGAGAAAGACGTGGTGGTGGTCACTATTAACTACAGGGTAGGAGCGTTAGGTTTC

CTCAGTCTAAACAGTCCTGAGGTGCCCGGAAACGCTGGCTTGAAAGACATGATTGCCGCGATACGATGGGTGAAGGAGAA

TATTAAGAATTTCGGTGGCAATTCAGGTAATATCACGCTTTTCGGTGAAAGCGCAGGCGGTGGGGCTGTATCTGTCTTGA

CAGCTAGCCCATTGACAAAAGACTTGATCAGTAAAGCGGTCATCCAATCAGGGACCGGTCTCAACAGCTGGGCATGGCAG

AAGAGTCCCTTTGAAAACGCTAAGAAGCTCGCTCACTACATCGGTTGCGAATCGGACGATTCTGATGAAATCTTAGAGTT

TCTCCGAGCAACTGCTATCAAGGATATAGTAGAAGCTACGAACAAATTGTTCCCGTTGGACCTATTCTTAGAAGGCAAGG

AGTTTGGCTTCGCTTTCGTGGTTGAAAAGGAGTTCCCAGGCGTTGAAGCAGCTGTCACCGAATCATTCCTAACCATGCTT

ACTTCTGGACGCGTCGCCGAAGTGCCAATCATGATCGGTTCAACCACAGTCGAATTCCTCTTTAAAATCGACTCCGAAGA

CTTACAAGTCTTCATTCCTAACAACCTAAATATCGAGAGGAATTCAGACGAAGCTATCCAAATAGCCAATCGGCTCAAGA

CTCTATACTTCAAAGGGAACCACACAGCTGTTGAAAACATCCACGGCTACTTCGAACTGATTTCAGACAAGCTCATTAAC

ATAGACACCCACAGACACGTGCAGTATCTAGTGCAGGCTACCACGAAACCAGTGTATTATTACAAGTTTGATTACGTGGG

TGAGCTGAACCTTTCTAAGAAGATGATGGATTCTTTGAACGTGACCCACGCAGGCCATACAGATGAGCTCGGGTATCTGT

TCAAGAATGACTCGCTTGATGGCATTGAGCCGACGGCGCAGGATGTCAAGATGAGGGAACGGATGGTACGGCTGTGGACT

AACTTCGCTAAGTCTGGAAACCCAACCCCAGACGAGAATCACTACATCAACATAACCTGGCAGCCAGTGACGAAAGACAA

CCTATACTACCTGAACATCGGCTCAGAACTCTCCGTCCAAACCAGCCCTGACAAGGAGAAAATGGACTTCTGGGACGAAC

TCTACAGCAAGTATTTTAAGATCTGGGACCATCCGCAGACCAACCAAGAAGAGTCGCCAGTTAAAGCCCATAAGGAGTCA

GTAGAAGTTGTTGAACCAGAGGTTATTCAACCAGAGGTGATACAACCAAAGGTTGTACAATCAGAGGTTGTACAACCAGA

GTCAGAGTCGGAACCGGTGGAGCCAGAAACGGGAGAGGCAGTCCAAGAGACGGTTGAAGAAGTATTGGTCGAAAATTTGG

TCGCGGTCGAGCCTCAGGCCAATGGTCACGCGAACGGATTAAATGGAGATACAGAGAGAAAGCCTAGGGCTTCCAATGAG

ATTAAGATGGTCCAAAGCAATGGCGCTCCTAAGGACGTCATCAGGGCAAATGATCCACCAGAAGATGATTTGCCTAAGAA

CATTGGCGTCAATAAGTTTGTGAATTTCTTCGAGTCACTGGGCGGTAAAAAGTGA

>Cobl_CXE34

ATGCAGCCTTTCGAGGCGGTGAAAAACGACATCATATGTCCTCAAGGAAAGTTCAAATACCTAAATCTGGACGACTACGA

CATGCGAGAGGACTGCTTGGTCGCCAACGTTTACATACCAGACACAGAGGAGACCAACCTTCCAGTCGTGGTCTACGTCC

ATGGCGGAGCTTACCAAGTAGGCTTCGGTGAAGCCATGACACCAACAGCTTTAGTGAGAAGTAAAAAGATAGTCGCTGTC

ACTTTCAACTACCGATTAGGTGTTCACGGCTTCCTGTGTCTGGGCACAGAGGGCGCTCCCGGCAACGCGGGCATGAAGGA

CCAAGTCGCGCTGCTGCGCTGGGTGCAGAAGAACATTGCCAACTTTGGTGGAAACCCTGATGACGTCACCATCGCTGGAT

ACAGTGCAGGTTCTTCGGCAGTGGATCTTCTCATGCTCTCTGATACGACTGAGGGACTTTACAATAAAGTAATACCTGAA

AGCGGTGCAAGCGTCGCCGTTTGGAGCGTTCAGATCGACCCGGTGCAAAATGCGAAGAAATTCGCCAAGCAATTGAACTT

CAGTAATGTAGACGATATTTATGCCCTAGAAGAGTTTTATTCTACTGTTTCCTATGATGCCATAACATCGGATGTGTTCT

TTGATCAAAAAGATTCAACCTTTGTATTTTCGCCTTGTGTGGAGCGTGATACAGGCGTAGAAAGGTTTCTTGTCGATGCT

CCTGTAAATATCTTAGCGGAAGGCAAGTACCGGAAAGTGCCTGTTTTATACGGGTTCGCAAATATGGAAGGTCTATTCCG

TATGATGGACTTTTTTCAATGGAAAGACCAAATGAATGAGCGTTTCTCGGACTTCTTACCTGCTGATCTGCAATTTAGCA

GTGACAAGGAAAGAGAACGAACTGCGCGAGCAATTAAGGAATTTTACTTTGGAGACGACCCGGTGTCGGAAAAAACTATA

CAAGGCTTCATTGACTATTTCTCGGACGTCATATTTGCTTATCCCCATCTGAGATCCGTGGAGTTCCAAGTCAACGCTGG

TAGTAATTCCATATACCTCTATGAATACTCATTCTTCAAGCCATTCCCTGAGAACATAGGCCTGCCAGACTTCATAAAGA

ATATACACGGAGCAGCCCACTGCGCTCAGTCAAATATGATTCAAGAAGGAAGCAAGTACTCCAGTAGTGTGGTTGATGAA

GAAGAAGATTTTAAGGAAATGAAGGCAACTATGGTCGAACTCTGGCTGAACTTTATAACCACCGGTAAACCGGTGCCTGC

AGGGTCAAAGCTTCCATCTTGGCCTCCTGTTAAAGGGAACAGGTCACCCTATATGGAGCTCAATTCAGAGTTAGCTCTGA

AGGGCCCTCTCCTCAAGAAGCGTGCGCTTTTCTGGGATGACATTTATGACCGGTTCTACAGAATGCCAGTGGCGCCACCT

GCTCCAAAAGTTAGGAGTGAACTTTAA

>Cobl_CXE35.1

GCAAAAAAAGCCGCGCGTAAAACAAGTAATCGGTTGCTTCCCCAGAATGATCCTGTGGTGACAGTAAAGCAGGGCAAGTT

GCTGGGAGCCGTGGACCAGCTGCATGATGGCTCGTCCTTCTTCAGCTTTAAGGGTATCCCTTACGCGCAGCCGCCGCTCG

GGCAGCTTAGGTTCAGGGCCCCTTCGCCACCTCAACCATGGAGCGGCACCCGTAACGCGACGCAGTACGGCGACATCTGT

ACCCAGTACGACCCCGAAGCAGGTGCCATCCTGGGCAGCGAGGACTGCCTTTTCGTCAATGTCTTCACTAAGTCCCTGAT

TCCTTCCTCCAAAATACCCGTCATGGTTTTCATCTATGGAGGATCCAACTCAGCCGGATCAGGCAACGACGACCTCTATG

GACCGAAATTCTTCCTCCAACAAAACGTTATCCTAGTTACTTTTAACTATAGACTTGAAGTACTAGGTTTCCTAGCCTTA

GATACCCCTGAAGTACCCGGCAATGCGGCCATGAAGGACCAAGTCGCGGCGCTGAAGTGGGTGCAGTGTAATATTGAACA

GTTCGGAGGAGATCCTAACAAAGTTACCCTCTTTGGTGAAAGTTCGGGCGCGGCTTCTGTCGGATTCCACATGCTGTCAC

CAATGTCCCGGGGTTTGTTCCACAAGGCTATAATGCAGAGCGGGACCAGTGCCAACGATTGGGCAGAAGGCCATGACGGT

AGAATCAGGGCGTTCAGAGTTGGCAAAGTCTTGGGAAAAGATACCAACGATGTAAATGAACTCTTGGCGTTCCTAAGAAG

CGTCCCAGCGGTGGATTTAACTAATATGACCATGAGGACTTTGTTGGAAGATGAAAAATATAGAGGCCTGCCTGAGACCT

TCATAACGGTTGTTGAAAAGAAGTTTGACAATGTCGTAGCGTTTTTGCCTGAACATGCATTGGACATTCTTGTCTCGAAA

AGAGCGTGCAGTGTTCCGTTGATGCTCGGATACAATTCCGCTGAAGCTATAATAATGCTTGAAGATAGGATGCAAAAATT

AGACATTTACAATAACAATCCCTCCTATGATGTTCCTAGAGAAATAGCTGAAAAGGTTACGCAAACAAAAATGACTGAAT

TTGGGGAAAGAATAAGGAACTTCTACGTCGGAAATAGAAAATTTACAAAGAATGATGGGGAACAAATTGTGGCCATGTTG

TCTGATATGCATTTCGTATACGGTGCGCATAGATTTGCTTATTTGTATTCTAAGTATAGCAATCCAATCTACATGTACAG

GTTTAGTCTCGTGAGTGATCTCAATATTGTTAAGAATGCAATAAATTCGACTGTAAAAGGAGTATGCCATGCCGATGACC

TATTCTACATGTTTTCAAATGATTTGACTAAAGATCAATATGAAGCACAGGATAAATTGAAAACCTATGTTTCGAAGGTG

ACGAAACTGTGGACCAACTTCGCAAAAACAAGTAACCCGACCCCAACCAGAAGCCTGGGGGCGACGTGGCCGCGGTACAC

AGCGAAGAACAAACGTTTCCTGGACATCGACGTTGATCTGACGCGCGGAAAATACGCAGAAAAAGAACGGGTGCAGTTCT

GGAATTGTCTGTTTGAGGAAGTTGACTTGCCTCATATAGACTAA

>Cobl_CXE35.2

ATGTCCCGGGGTTTGTTCCACAAGGCTATAATGCAGAGCGGCACCAGTATCAACGATTGGGCGGAAGGCTATGACGGTAG

AATCAGGGCATTCAGAGTTGGAAAAGTCTTAGGAAAAGACACCAATGATACAAATGAACTCTTGACGTTTTTAAGAGGCG

TCCCAGCGGTTGATTTAACAAATATGACAGTCAGGACTTTGTCGGAAGTTGAAAAATATAGAGAGCTTGAGACCTTTACA

CCTGTCGTTGAAAAGAAGTTTGCTAATGTCACAGCGTTTTTGCCTGAACATGCCGTAGACATTCTTGTCTCGAAAAAAGC

GTCCAGCGTTCCGATAATACTCGGATATAATTCTGCTGAAGATATAATAATGCTTGATGATAGGCTTCAAAATTTAGACT

TTTACAATAAAAATCCCTTCTACGATATTCCTAGACAAATAGCTGACAAAGTGACGCAAGAAAAATTGACTGAATTCGGT

GATAGAATAATAAAGTTTTACGTCGGAGATAGAAAATTTACACAGAATGATGGAGAGCAAATTGTGGCCATGCTGACTGA

TGTGAATTTCGCGTACGACGCGCATAGATTTGCGTATTTGTATTCTAAGTATAACAATCCAATTTATATGTACAGGTTTA

GTTTTGAAACTGATCTCAACGTTATTAAGAATGCAGTGAATTCGACGGTTAAAGGAGCGTGCCATACTGATGACTTATTC

TACATGTTTTCATCAAATGTGACTAAAGATCTATACGAAGCGGAGGATAAATTGAAAGACTATGTATGGAAGGTGACCAA

ACTGTGGACCGACTTCGCAAAAACGAGTAACCCGACCCCAAATAAATGCTTCGATGCGCTGTGGCCGCGGTACACGGCGA

AGAACAAAGAGTACCTGGACCTCAACGTCAAACTGACGCCCGGAAAATACGCGGAAAAGGAACGGGTGGAGTTCTGGAAT

AGTCTGTACAAGGAAGTTGGCTTGCCTCATATAGACTAG

>Cobl_CXE36

ATGTCCCGGGGCTTGTTCCACAAGGTTATAGCGCAGAGCGGGACCAGCGTCAACGACTGGGCGGAAGGCAAAGACGGCAT

TAACAGGGCATTCAGAATTGCCAAAGCCTTAGGAAAGGATACCAACGATAAAAATGAACTCTTGGAGTTGTTAAGAACCG

TCCCAGCCGTTGATTTAACTAGAATGACCATGAAGACTATGTCGAAAGACGAAAAGTATAGAGGCCTGCCCATTCACTTC

GCACCGGTCGCTGAAAAGAAATTTGACAATGTCGAAGCGTTTCTGCCTGAAAAGCCATTGGACACTCTGCTCTCGAAAAG

AGCGAGCAGTGTTCCGTTAATGCTCGGATACACTTCCGCTGAAGGCATAATCATGCTNTTGAAAAAATTAGATTTTTACT

ATGACAATTCCTCCTATGATGTTCCTAGAGAAATAGCTGGGATAGTAACGCAAGAGAAAATGAGTGAATTCGGTGAAAGG

ATAAGAAAGTTTTACGTCGGAGATAGAAAATTTACAAAAGAAGATGCAGAACAAGTTACGGCATTGCTGTCCGATCTGCA

TTTCGTGTACGGCACGCATAGATTTGCTTACTTATATTCTAAGTACAACAATCCAATTTATATGTACAGATTCAGTCTTG

TGAGTGAACTCAATTTCTTGAAGAATATATCAAATCCGAATATAAAAGGAGCCTGCCACGCCGATGATTTGTTCTATATG

TTTTCAACTAATATATTGAGTGATCAATATGAAGCGCAGGATAAATTGAAAGACTATGTATGGAAAGTAACGAAACTGTG

GACGGACTTCGCAAAAACAAGTAACCCGACCCCTGACAAAAGCCTCGGCGTGACCTGGCCGCGGTACACAGTGACGAACA

AAGAGTTCTTGGACATCGCCGAGCAGCTGACGCCCGGAAAATACGCCGAAAAGGAACGGGTAGAGTTCTGGAACAAATTG

TACGCGGAAGTCGGCTTGCCTCATATAAGTAGGAGCAACTTGTAA

>Cobl_ELOVL1

ATGGAGTCCGCAACTTGGACCTACAATTTGGACACTACGCCAAACTACTCGTACATTTTTGATTTTGAGAGCGAGTTTAT

TCACCAGAATTCTAGGAAATGGATGACCGAGAATTGGACTGTGGCGTTTTACTACATCTCGGTGTATATGGCGTTTATTT

TCTTCGGGCAGTTGTACATGCAGAACCGTCCCAGATTCGAGCTCCGACGGACACTGATCATCTGGAACGGTGGACTGGCC

GCCTTCAGCATCATGGGAGCCTGCCGGACGCTCCCTGAATTCATCCACGTGCTACGAAACTATGGGATCTACCATTCTGT

CTGCGTGCCAAGCTTCATCGAAGCGGACAAAGTATCCGGCTTCTGGACGTGGATGTTCGTCCTCTCCAAAGTGCCCGAGC

TAGGGGACACAGTATTCATTGTACTCCGCAAGAAGCCGCTCATCTTCCTCCACTGGTACCACCACATAACGGTGCTGCTG

TACTCCTGGTTCTCCTTCACCGAGTTCACCTCCTCGGCCCGCTGGTTCGTGGTCATGAACTACTGTGTGCACAGTGTGAT

GTACTCCTACTACGCTCTAGTAAGCATGGGCAAGTACCCCCCAAAATTCCTCGCAATGACAATCACCTTCCTCCAACTCA

CGCAGATGATCGTCGGCTGCGCGATCAACATTTGGGCGCACAACTACTTAACCTCCTCCACAGGTTCTTGCGGCATCAGC

CAGTTCAACATCAAACTGTCCATGGCTATGTATTTCTCCTACTTTATTCTCTTTGCCCGCTTCTTCTATAAAGCCTACCT

AGCCCCCAAAGCGGGCAAGAAGGCTAAGGCCGAGCCTGCTATCGATATCCCGAGCGCCAAGAAGAATTATGACGAATACC

CTCGGCAACGGAACGGCGTCGTCGCTCACTAG

>Cobl_ELOVL2

ATGTCGGGCTTTGTGAAAATCGCCATCGACGATTACTATTCCGATAGTAATTGGACGACCATCATCGAAAAGTACTGGAT

GATGGCCGAAAAAGTTTCAGATCCTCGCGTGCAAGGATGGTTCCTTTTCGACACGCCCCTCCCCACTCTTGCCATGGTGA

TAGCCTACATCGCCTTCGTGATGGTCATCGGACCCCTGTGGATGGAGAACAAGAAACCTTTCCAGATCAAGAACACCCTG

GTGGGATACAACGCTTTCCAGGTGTTGCTGTCGTCGTATATGTTTTATGAGCACTTGATGTCGGGATGGTGGGGAGATTA

CAGTCTGGCGTGCCAACCTGTCGACTACAGCGAGAGTGAACAAGCGAGAAGGATGCTGCATCTATGCTGGGTGTACTACT

TCTCCAAGCTGTCAGAGTTCGCAGACACGGTGTTCTTCGTGCTAAGGAAGAAGAAGAGTCAGATCACGTGGCTGCATCTA

TACCACCACTCCCTTACGCCTTTTGAGGCTTGGATGCTGGTCAAATTCATTGCTGGCGGTCACGGAACCTTCTCGAACAT

CGTGAACAACCTCGTCCACATCATCATGTACGCGTACTACATGGCATCGGCCATGGGACCCCATATGCAGAAGTACCTAT

GGTGGAAGAAGCACCTCACCACTCTTCAACTAGCCCAATTCTTCATGGTGCTGTTCCACTCCATCAGTGCTCTCGTCTGC

GACTGCGGATATCCAAAGATCATCGCCTTCGGCCTCATTCTCCACTCGAGCATCTTCATCGTCCTCTTCACAAACTTCTA

CATCCAAGCGTACAAGAAGGACAAAATCAAAGCTAAACCAGAACCCACCAATAACAACAGCCTTGCCAACGGTTACGCCA

AAACCAACGGCCAAGTGCCCAATGGCCAAGTATCAAACGGCCAAGTATCCAACGGCCAAGTGCCCAATAGCCAAGCCAAT

GGCCATGTCAAAAATGGCTTAACGAACGGTCACGCCACTGACGTCAGAGAAAAAACTAAAGTACAGTAG

>Cobl_ELOVL3

ATGGAGCAGCTGAATAAGCTAGTCCAGGGTTACCATGACCTTATGGACAACAAGAGTGACCAGAGGGTCAAGGATTGGCC

ACTGATGTCCTCTCCCTTCCCCACATTGGCGATATGTCTCACATACGTGTTTGTAGTCAAAGTAGTGGGGCCAAAGTTTA

TGGAAAATAGAAAACCGTACGAATTAAAACAAGTGCTTATCTGGTACAACTTAATTCAAGTCATCTTCAGCATTTGGTTG

TTTAATGAGAGTGTGGTCAGCGGATGGTTCACGACGAACAGTTTCCGCTGCCAGCCCGTCGACTACTCGCGGTCGCCCAT

CGCTATGCGGACGGCGAGCGGTTGTTGGTGGTACTATTTTTCGAAATTTACGGAATTCTTCGACACGCTCTTCTTCGTGA

TGCGCAAGAAGTTTGACCACGTGTCGAAGCTGCACGTCATCCACCACGGCATCATGCCCATGTCCGTCTGGTTCGGAGTC

AAGTTCACACCAGGTGGCCACTCGACCTTCTTCGGATTCCTGAACACGTTCGTGCACATCATCATGTACTCGTACTACCT

CCTGGCCGCGCTCGGGCCCAGCGTTCAGAGGTATCTCTGGTGGAAGAAGTACCTCACCGCTCTGCAAATGATCCAGTTCG

TGTTGGTATTCTTACACGCGTTCCAGTTACTGTTCGTTGATTGCGACTACCCTCGTGCCTTCGTCTGGTGGATCGGCATG

CACGCTGTACTGTTCTACTACCTATTCTCTGACTTCTACAAGCAGGCCTACCGCAAGAAGGCAAAGGCCCAAATCGCTCG

AGCGAAAGCAAAAGTAGCTGAAGCGGAGGAGACGAGCAAGTCGGAGACGAAAGACATGATGTACCCGTTGCTGAATGGGT

TTAGCAACGGCGCGGCACTCGGCGACGTGCGGCAGCGGGTCGCGGGCGCCGTGGCCTCGCAGTGA

>Cobl_ELOVL4

ATGGGGACACTAGTTCATAACATCACGCAGTTCTACCATTATATGAACACAGATTTGGCAGATCCTCGATCGAACAGTTT

CTGGCTGGTATCGAGTCCATGGCCCATTATGATCATAATGTACGCGTACCACCAGTTCGTGCGCAAATGGGGCCCCGCCT

TCATGGAGAATCGGCCTCCGTACCAGCTCAAGAACCTTATCATTCTGTATAACGTTGTGCAGATATATTTTTCGGCATAC

ATAGCGTTGCGTTGTTTCCTGACCCTGTATTGGTCGGGGCATTACAGCCTGTGGTGCCAGAAGATGATCTTCGACGAATC

GCCCATCGAGACGATGGTGGTCAACCACGTGCACTTTTACTATATCATCAAAGTCATAGACTTGCTTGACACGGTTTTCT

TCGTGTTGCGCAAGAAGTTTTCGCAGGCGTCGTTCCTGCACGTATACCACCACCTCGGGATGTGTTTGCTAGGATTCGTC

GGGACCAAATATGTGCCAGGTGGGCACGGCGTGATGCTCGGCTTCATCAACTCGCTAGTCCACGCAGTCATGTACTCGTA

CTACCTGGTGTCCATCGTGCGCCCGCAGTGGGTGCGGCGCTGGTGGAAGAAGTACATCACGCAGATGCAGATCTTACAAT

TCTTCCTTCTCATCCTGCACTTCGGGCACGTACTATTCGAGCCGTCATGCGAGTACCCGAAGTGGGTGTCGCTCGCCTTT

TTGCCGCACAACATATTTATTCTGTTCCTATTCTTAGACTTCTACTTTAAGGAATATGTGTACAAAAAACAAACTAAGGC

TAAG

>Cobl_ELOVL5

ATGGATGAAATGGCTAATCCCCAAGTCAACATGTCGATAATCGACTCTTACAAGAGCTTTATGGAACGAAATAGTGATCC

CAGGACAGAAGCATGGACACTAATGTCTGGACCGGGCCCCCTGCTCACTATCCTTGCCACTTACCTGTACTTCTGCACGT

CAGTGGGGCCCAGCTATATGAGGGACCGGAAACCATTCGACTTAAAAAACACGATTGTGATATACAATGTCATCCAAGTA

TTGAGCAGCATGTTTTTGGTATATGAGGGTATGGCTTCGGGTTGGTGGAATGACTACAGTTTTTCTTGCCAGCCAGTGGA

CTATTCAGATAATCCCAAAGCCAAAAGGATGGCAGCAGCGGTATGGTGGTACTTCATGGCTAAGGTCGTTGAACTGCTGG

ACACTATCTTCTTCGTGCTAAGAAAAAAGAACCGGCAAATTTCATTCCTCCACCTTTACCATCACTTCATGATGCCCATC

TGTGCCTGGATCGGAGTGAAGTTCTTGCCAGGCGGTCATGGCACCCTTCTGGGCGTCATCAATTCCTTCATCCACATCAT

CATGTACACGTATTATCTCATCTCTGGACTCGGGCCCCAGTACCAGAAGTACTTGTGGTGGAAAAAGCATGTAACTACTT

TACAGTTGATCCAGTTTTGCATTATCTTCTACCACAACTTCTCGGTAATGTTCTGCGACTGCAACTACCCTAAGGTCATC

AATTTCTTGCTTGCTCTTAATGCTGGTCTCTTCTTGTACATGTTCGGTAAATTCTATTACAAGAACTATGTCAAATCCAA

CACCAAGTTAGTTCAAAATGGTAGTGTTAAGAAAGAAGTAGTTGTTAATGGTGATCTTAAAATCAATGGTGCGCCCAATG

GAGAAGCGCAAAAGGAATCTGATCATAAAACAGAAGTTAGTAACGGAAAAGCAAAACAATGCTAG

>Cobl_ELOVL6

ATGAGCTACCTGGAGAAAGTCGACCGGTACCTGGACTCGCTAAAAGTTGGGAAAAGCGCCATGGTGGACTCATGGTTCAT

GATGTCCAGTCCCATGCCGATCCTTGCCGTGGTGATAGCTTACCTGATCGTCGTGCGAGTGGGGCCGCGGATGATGAAGA

ACCGACCCCCGCTCAGGATCACCAGACTCATCACTTACTACAACGCCACACAAGTGCTGTTCTCGGTGATGATCGTTTAT

AAGGCCCTCAATTTGAACATATTTCGAGACGGCGTCTTATATGCCGGGTGCAGATATCCGTCGAATACGCAAAATAAGCA

GTTATTGGACCTGGGCTGGTGGTACTTCTTTGCAAAATTCACTGAACTTTTAGACACGGTGTTCTTCGTATTGCGGAAGA

AAAATAAACAGGTGACGTTCCTCCACGTGTACCACCATGCGATAATGGCGCTGTACTCGTGGTCCTACCTCAAGTTCGCA

GCCGGTGGGCAGGGCACCATCCTCGCGCTCCTCAACTCCGCAGTGCACGTAGTCATGTATACCTACTACCTCCTGTCCGG

GCTGGGACCCCAATTCCAAAAGTATCTGTGGTGGAAGAAGTATGTTACTACTTTGCAGTTGGTCCAATTTGTGATAATGC

TTCTCTACTGCCTGTGGACTCACTTCTCTCCTAGATGTCAATTTGCAGTCGGGTTCACCTACTTTATCTCTACGAACGTA

ATCATCTTCCTCTTTCTCTTCCTCAATTTCTATTCTAAAAGTTACAAAAAAAAGAGAATCGCGGAAAAAGAGATCCAAAA

GGAGTTTGAGAGAGCGAGAAATGGGACCAAACAAAATGGAGTCACTGAAGCAAATGGACTTGCGGAGAAATGTGGGAAAA

AGAACGGGGTGTGCACAAATGATTGTTCAGATATTCCCAATTTAGAGGAGGTCCCTTGTGAAGTAGATAAAGCGTGTGGT

GACTACATCAAAAAGGGTAAAGTGTTCCTTACTAGACGTACGGCTAAGCAAGCCTACGGACCAGAATATGACTTCGAAAG

CATAACAAAGAAATGA

>Cobl_ELOVL7

ATGTCGCTCTCCAACAACTCATTCGCACGGACCTACAACCGACTATTTGTAGAATTAGCAGACCCCCGCACCAATGACTG

GTTCCTGATCACCAGCCCGATACCAGGGCTCACAATCATATACCTGTACCTCCAGTTCGTACTGAAATGGGGCCCGCGCT

ATATGGCAGACAAGCAGCCCTTTCAGCTTCAGAAGACCATGGTGGTCTACAATTTCCTGCAAGTGCTGGTTAGCTGCTGG

CTGTTCCACGAGGGTTTGGATGCGGGATGGCTGAGGACTTACAGCTGGAAATGTCAACCAGTAGACTTCTCGGATACGCC

AGAAGCAGTCCGGGTCGCGCGAGGAGTATACGTCTACTTCCTAGCAAAGATGTCAGAGCTCCTCGACACAGTTTTCTTCG

TGATCCGAAAGAAGGAGCGGCAGATCACGTTCCTCCACATGTACCATCACACGGTCATGCCCATGATATCCTGGGGCGCC

ACCAAATACTACCCCGGGGGTCACGGGACCCTCATCGGGGTCATCAACTCCTTCGTCCACATCATCATGTATACCTACTA

CATGTTCGCTGCCATGGGGCCGAAGTACCAGAGACTGTTTTCGTGGAAGCGTCATATAACTACGCTGCAAATGGTCCAGT

TCTGCATCACGTTCCTCCACTCCTCTCAGCTCCTGTTTTACGACTGCGGCTACCCGCGCTGGTCCGTAGTCTTCACTCTA

CCGAACTCCATCTTCTTCTACTATCTCTTCTACGATTTCTACTACAAGTCCTACAAGACCCCGGAAGAAACAAAAACGAA

CGGCCAAGTCAAAGAAAACGGAGTAAAGGACCACCCAAAATTAGGAAATGGGACCAACGGGATTAAAGATCAATGCGAAG

CGAAAAAGGTCAAATAA

>Cobl_ELOVL8

ATGGCGCTAATTTTGAGGACTGCGTGGCAAGGGTACCGATACCTGTTTGAAGATTTGACGGACCCAAGAACGAACACATG

GTTTTTAGTGGCAAAACCTCACCAAGGCGTCGCATTATTGGGCCTTTATCTCATGTTCGTGCTGAAATGGGGACCTGATT

TTATGAAGAATCGACCAGCATACAACTTGGATAAGGTCCTTATCGTGTATAATGCACTTCAGATATTGGTTTGCGCTCGA

CTTTTTGCTGGGTCCTTATTATATGCCTGGGCCTTCCACTACCGATGGATTTGCGAGCCTGTCGACTTCTCGAATAATGA

ACATGCTATCCAGGTAGCAACATACGTCCACGCCTACTTTCTCATCAAGGTCTTTGACCTCCTCGACACAGTATTTTTCG

TTCTGCGTAAAAAATTCAATCAAGTCACTTTTCTGCACGTCTATCATCATTTCGGAATGGTCATGCTGACTTGGGGAGCT

GCCACTTACTTCCCAGGTGGTCACGGCACACTGGTGGGGGTAATAAACTCCTTCGTTCACGTGGTCATGTATGGATATTA

TCTGCTGACAGTGGCCATGCCGAGTTTGAAGAACTCCTTGTGGTGGAAGAAATACATCACACAGTTGCAAATTTTACAGT

TCTTCTGGACCGTAGTGCACATGGGGGCTTTAGTTTTTAAGACGGACTGTGCCTACCCTCGGTGGGTGGCTGCCATATTT

TTACCACAGAACTTATTTATGCTGGTTCTGTTCATAGATTTCTACATAAAAGCCTACATTAAACCTAATAAAGGGAAGAA

AGAAAATGGCGTCCAAAATGGTAACCAGAACGGCGTCCAAAATGGTAACCAGAACGGCGTCCAAAATGGTAACCAGAACG

GTGTTCAAAATGGTGCACAAACCGGAGTCCAAAATGGTCAAAAGAATGGACTTAATGGTGTTAGTGAAGGAAATGGACGA

GTGAAAGCCGATTAG

>Cobl_ELOVL9

ATGGCGCTAATATTAGAGTATATAGACCGTATAAATATTTTTATGGACAAATATGGAGATTCCCGGACAAATCCGTGGTT

CCTGATGAGTTCCCCATTCCCTACATTAATATTATGTTTAAGCTATGTTTATCTAGTCAAGGTTCTAGGACCAAGGTTTA

TGGAAAACCGAAAACCATACAATTTAGGAAATATTTTAGTAGTATACAACTTTTTACAAGTACTGTTCAGTACGTGGCTG

TTTTACGAGATCGGGTTCTCGGGATGGTTCACAGGCAGGTATAACTATCAATGTCAGCCGGTCGACTACTCCAAACATCC

ACAAACAATGAGGATGGTACACGCTTGCTGGTGGTACTATTTCTCGAAGTTCACAGAATTCTTTGATACGATCTTCTTCG

TGATGCGTAAGAAGTACGACCACGTGTCGACGCTGCATGTGATCCACCACGGAGTAATGCCGATGTCTGTCTGGTTCGGG

GTTAAGTTCACACCAGGCGGCCACTCCACTTTCTTCGGTCTGCTCAATACGTTCGTGCATATAATCATGTACACGTACTA

TATGCTCGCGGCAATGGGACCCTCGCTGAGGAAATACCTTTGGTGGAAGAAGTACCTCACTGCGCTGCAAATGGTACAAT

TCATTGGCATCATGCTGCACGCGTTCCAGCTCCTGTTCATCGACTGCGACTACCCGCGCGCGTTCGTCTGGTGGATCGGC

ATGCACGCCGTTATGTTCTTCTTCCTGTTCAAAGACTTCTACAACCAGTCTTACAGCAAGAGGCCTAGGGTCCGCGCAGC

ATCCCCCAAGCCGGAGATAACAGAAATCCCAGCGCCGTACAAGAACGGCGCGCCGCTCAAGAACGGCTTCAGCAACGGGC

ACGCGAACGGCGCGCCGGCGCTGCGCGCGCGCACCGTGCTGCCG

>Cobl_ELOVL10

ATGACGAGCGTCTACAACTGGTACCGGGACCTCCTGGACAACAGGAGCGATCCTCGCGTGAAGGATTGGGCGATGACGTC

ATCACCTTGGCCCACGGTGGCTGCGTGTCTCTGCTACGCTTACTGCGCCAAGTGGCTCGGCCCACGGCTGATGGCAAACC

GCAAACCCTTCGAACTAAGGGGCGTCCTCATCGTATATAATCTAGCCCAGACACTCTTCAGTACCTGGATATTCTATGAG

TACATGATGAGCGGCTGGTGGGGTCACTACGACTTCAGGTGTCAACTCGTCGACTACAGTAAGAGCCCAATGGCCATGAG

GATGGCAGCGACGTGCTGGTGGTACTACTTCAGCAAGTTCACGGAATTCTTCGACACTCTGTTCTTCGTGCTCCGCAAGA

AGAACGAGCACGTGTCGACTTTGCACGTCATCCACCACGGGATTATGCCCTTCTCTGTCTGGATGGGGGTTAAGTTCGCA

CCAGGTGGCCACAGCACTTTCTTCGCACTGCTCAACACGTTTGTGCACATCGTGATGTATTTCTACTACATGGTGTCGGC

CATGGGACCCAAATACCAGAAGTACATCTGGTGGAAGAAATATCTCACCGCTTTCCAGATGATCCAGTTCGTGCTAATCT

TCAGCCACCAGCTCCAAGTGCTGTTCCGGCCGTCGTGCCAGTATCCGCACGCGTTCGTCTACTGGATCGCCATGCACGGC

TTCCTGTTTTTGTTCCTCTTCAGTGACTTCTACAAGGCGCGGTACACGAAGCGCGAGAAGTCGAACAAGCGGACCGGAGG

ACTCTGCATGGTGGTGGACGAAAGCGGCCCGCTGAACGGCAAGAACGGATACAAGCAAGAGGAGGGTTCGGAGGTCCCGA

GTTCGTACGCATCTTCGTCGGCGGACGCTTTCGTGCGCCGGCGCCCCGTGTCCTAG

>Cobl_FA2H

ATGGATACTCCGCACTTCCCGGTCCAAGTCGACGGCAAGCGCTATGACATAAAAAAGTTCCTTCGCGACCACCCCGGGGG

GGTGGGAACCCTAAAAAACTATGAAGGGAAGAGCATCCTTCAAGCCATGGAAAAGTTCGGACACAGTATCAGTGCGTATC

ACATGTTAAATGACTTCAAAGTGGACAGTGAGTTAAAAGATAGTAATTTGACGGGTGGAGTGAGTGCGAACGGTAGGATT

ATCACAAACGACGAGGGCAGCCGGGATAAGGCGGAGATCGAGTATTTGGAGGAATTGGAGGGCAGACTAGACTGGTCAAA

GCCAATTCTCTGCCAGCTCGACGCCATCGCGCCCGACTACGAGCGATGGGTCAACAGCGCGGTCTACCGGCACTGCCGGA

TCTTTGCCAGCCCTATCCTGGAAGCCATGACCTTCACACCCTGGTACCTGGTCCCAGCCTTCTGGATACCCATCATACTG

TATCTGGGGTACACGCAGTTCGGCGAGCACGTGCTGAATGCAGATACACAAAACGCGATGTCCCTCCTCGAGTACGGGTA

CCACATGGTCTTCGGGACCCTCATGTGGACCATCCTGGAGTACTCGCTCCATCGGTGGGTGTTCCATCTGGACCCGGGGA

GGTCCATCACTATGATCAAGCTGCACTTCCTCATCCATGGCTTACATCATAAGGTGCCGTTCGACGGTCTTCGTCAGGTG

TTCCCTCCAATTCCAGCGTTCGTGTTGGCGTCAATAATCTACATGCCGGTGCGGCTCCTGCTATCTTATCCACTGATCAA

ATTGACCGGAGGACTCATCGGTTACCTAATATACGATATGATCCACTACTACGTGCACCACGGTTCTCCAGAGGACGGCA

CGTATCTCTACACCATGAAGCGGTATCACTCTAACCACCACTTCATTAACCATGACAGAGCATTTGGCATCAGCAGTGAC

ATCTGGGACCACGTTTTCAAGACATTTGTAACCGTCAGGAGGCTAGGTTTCAGTTTGAGATGGCAGTAA

>Cobl_FAAH1

ATGGAGGTAGGGGTCCAGATAATAGGCATAGTTCTACGACTCATCAACGCGCTGTTGGCTCCGTTATTCTGGCTCTACAC

GCGAGGGCCGCAGCCCCGTTTGCCACCGACGAAGGCCATACATCTGAGGAGCGCTACCGACCTAGCCAGAGCAGTTAGAG

ATGGAGAGATGACAAGTGAGCACCTAGTATCGACGTTCATCGATAGAGTCAAAGAAGTGAACCCACTTCTCAATGCCGTT

GTCGATGAAAGGTTCAGCGCGGCGCTAGCGGATGCTAAAGAGGTGGACCGGCAAATAGAAGAAGCCAAAAGAAGTGGGAG

CTTCAAGGAGCTAGTGGCGAAGAAGCCGCTGCTGGGGGTCCCTTTTACGGTTAAGGAAAGCTGTTCATTGGCTGGCTATT

CAAACGCAGTGGGTTGCCTCGAGAATGCTGGTCGCCGCGCGACCACGGACGGAGCCGCGGTCAAACTGGTAAAAGAAGCC

GGGGCCATACCTCTCCTCGTGTCCAACACTCCTGAACTATGTCTGGGGTGGGAGACCACCAACCTGGTCAACGGCACAAC

CAACAACCCTTACTGCCTCACTAGGACACCTGGCGGGTCGTCGGGCGGTGAAGCGGCTCTCCTAGCGTGCGGCGCCTCAG

TATTCGGTGTGGCGTCGGACATCGCAGGTTCAATCCGTATCCCAGCTGCCTTCTGCGGTGTGTTCGGACACAAACCCACG

CCCGGCATAATCCCAATCGACGGCCACATCCCGACTCTGAACGATGAAAACTACCCCAAGTTCCTGACCGTCGGGCCGAT

AACGCGCAAAGCCGAGGATTTGCCGCTGCTACTGAACGTTATGGCGGGAGAGAACCGACACAAGCTCAGGCTGAATGAAC

CTGTTGATCTGAGTAAACTAAAGGTTTTCTACATGACTGAAGCAACCGACTCCATAGCTCTACTTAATGTGGATAAAAGT

ATAAAGGATACGATCAGAAACGCTGCTCGGTATCTTGGGAAAGAATGCGGTGCTACTATAAGTGAAGAAAAGTTTGAAGA

GCTAAGAGACTCGGTGGAGTTATCGATATCAGTATTCTTCTCTATGAAGGACATTCCTAACATGTTGCAGGACCCAGCAA

ATCCAAAGCGTGAACGCAGTCTAATACTCGAGCTTATCAAATACTTCCGCGGCGACGGTTCCCGGTCTCTACAAGGATTA

GGGTTCTCTCTCATCGACAAGACCAAACTCTTCATACCGGCCAGCCGGCGGAAACACTACTGCGAGAGAGCTGACGCGCT

GAAGGAAAAGATCACGCGGACGCTCGGCGACAACGGTGTGCTGCTGTACCCGGTGTTCACCGACACGGCACATCATCACC

ATCAGGTGTTCGCGCGGGCGTCCGGTGTGGTTTACACAATGCTGGTCAACGTGCTCGGCCTGCCCGCGGCCGCCGTGCCC

GCCGCCGCCGCCCGAAGCCAACTGCCCGTCGCCATACAGGTTATAGCGGGCCCATATCAAGACCGGCTATGTTTGGCGGT

GGCGAAACAACTCGAAGTCGGTTTCGGCGGCTGGCAACCACCCTCTTAG

>Cobl_FAAH2

ATGCTGAACGGCTTCATGCGATGGCTGCTCCGGTTCCTGGTGTCCCTCATAGCGATCGGAGTCCGGCCCCTCACATACCT

CCTCACCATCAGGAGAACGAGGAAATGCCCTCCACCAACCAACCCCATACTCTTCAAGTCTGCCACTACGCTCACTATGA

TGATTAGGAATAAACAGATCACCTCAGAAGAAGTAGTGGCAGCGTACATAGAGAGATGCAAAGAAGCGAATCCCTACATC

AATGCTGTGGTGGAACCACGGTACGACCAGGCCTTGAGAGAAGCCAGGAGTATAGACAAGATGATAGCTTCTACCGACAG

GACACCTGAAGAGCTGGGGAAGGAGTATCCATTGCTTGGAGTCCCCATGACTGTCAAGGAAAGCATAGCTGTTGAAGGCA

TGAGCAACGACTGCGGAACCGTCCGTCCGACCAGAATCCCAGCAACTAAAGACGCTGCCATCATCAGCCTAGCCAGGGCC

GCGGGGGCCATACCCATAGCTGTCACCAACACCCCTCAGTTCTGCATGAACTGGGAGACTTACAACAACGTCACGGGACT

CACTATGAACCCTTATGATCAAAAGCGGACTACAGGGGGGTCTTCGGGTGGAGAGGCTGCCTTGATATCGTCCGCTGCGT

CAATAATGGGCGTGGGATCCGACATTGCGGGTTCCTTGAGACTACCTCCCATGTTCACGGGTATCTTCGGGCACAAGCCT

ACTCCCAGATTACTGTCCGTTGTAGGTCATGTTCCAGACTGCCCTGCGTCTGATTTTGAAGATTATTTCGCTCTGGGACC

TTTAACTCGATACGCTGAAGATCTGAGTTTACTTCTCAAGGTCTTGCGACAACCAGGAGCACCGGACGTGCCACTAGACA

AGCCCGTAGACGTTTCAAAGTTGAAGTTCTACTACATGGAGGGGGACAACAGCAACGTCACCGACAAAATTGGCTCTGAC

GTCAAAAAGGCGATGGAAAAGGCAAAGGCGTACATGAAAAGTACTTACAACATTGAAGTTAAAGAGCTCAAAATAAAGAA

CATCGAGCACATATGGGAGATCAGCGTAAGAGTTCTAATGAACATCCGCAACGTTCAGAACATCTACACTGACCCGGAAA

AAAGGGACCAATGGGTGTCCGTATGGCCTGAAGTACTCAAGAAAATGGTCGGTCTCTCTGACCACAACTTCACGTGTGTG

GCGTATGGACCTCTGCAGAAATTCTTCGACTCCCTACCTAAGAGCTACTACGCGAAACTGTTGGCTATGTTTGAGGAAAT

GAAGAAAGATTTTGAGGCTGCCCTATCTGACGACGGCGTATTACTATACCCAGCGTTCCCTTACCCAGCTCATTTACACT

ACAGAGTGTATTACAAGTTCCTAAACTGCGGGTACCTGACCATTTTCAACGCTTTAGGATTACCAGCGACGGCGTGTCCT

ATAGGACTTACTGACAAGGGCCTACCAGTGGGCCTGCAGCTAGTGGCTAACAAATGCAATGACCATCTGACGATAGCTGT

TGCTAAAGAGTTCGAGAGAGCATTCGGTGGCTGGACTCCACCCAACAAAGAAATGATTAGCGTCTAA

>Cobl_FAAH3

ATGGCTACGAACAAAGTCGTAAGAGGAACAGAACAAAATAAACGGGGAAAAACAACAAAAATCCTCAAGGGTATGGCGTT

CAACATGTTCAAGCATGTCTTCATCTTCATCCGAGGATACGTGGAGCTGATCATCGACCTGATATTCGCGTTCATATGGG

ACGGGGAACGGCAGGCCGTGCCCGATTTGGAGGCAAGGCACGCAATGCTGATGGAGAGTGCCGTGGAACTGGCTAAACGG

ATTCGGAATAAGACTTTGAAGTCCGAAGAGCTGGTGCGGGCTTGCGTGGAGAGGATTCAGATTGTAAACCCAGTACTGAA

CGCAGTAGTGGACCAGAGGTTTGAAGAGGCGCTGAAGGAGGCACAAGAGATTGATAAGCAGATCCAGAGTGGCCTGCCCG

ATAAGTACTTTGAGGAGAGGCCTTTTCTGGGCGTGCCATTCACGGCTAAGGAGAGCCACGCAGTGCGCGGCCTGCTGCAC

CCCCTCGGCATCGCCGCGCGAGCTAACGTGAGGGCTACTGAAGACGCGGAGTGCGTGCGTTTGCTGCGGGCCGCTGGAGC

CGTACCGGTGGCTGTCACTAACGTGCCTGAGATTAACAAGTGGCAAGAGTCCCGCAACAACGTGATCGGGCAGACTTGCA

ACCCGTTCCACACGGGGCGCACGGTGGGCGGCTCCAGCGGCGGCGAGGGAGCCCTACACGCTGCGCTCGCCACGCCCATC

TCACTGTGTTCTGACATTGGTGGGTCTACGCGTATGCCTGCATTCTACTGTGGATTGTTCGCGCTTAACCCCACTGCTGG

TTACACCAGCCTGAAAGGGTCTGCGCTGCGTACCGGGCTGGACCCAACAATGGCGTCGATCGGGTTCATCAGCAAACACC

CTGCGGACCTGGCGCCGCTCACCAAGGTGGTCGCAGCCGACCAGGCCGGGAAACTCAACCTGGACCGAGTTGTTGATTTG

AAGACTGTGAAGGTGTACTACGTGGAAACAGCTCAGGATCTGCGCGTGAGTCCCGTCAGCTCCGAACTTAGGGAGGCTAT

GAACAAAGTGGTGACAAAACTGTCAGAACTCGGTCGAGTGACAGCCTCATCACCTCAACCATACTACCACCCCGGGTTCG

AGCATATGTTCGCGCTGTGGAAGCACTGGATGACCAAGGAGACCGAAGACTTCCCACGAATGCTGACTGATAATAAAGGA

GTCGCTCACGGCCTTACGGAGCTTGGGAAAAAGCTAATTGGCACATCCCAATACACGATCGCCGCCATCTTGAAACTCTT

GGACGAGCAAGTGCTGCCGCCTGTCAACGCTGCGTGGGCTGAAAAGTTGACCGACAAGATGCTAGAAGAACTCACGACGG

TGCTCGGCGACTCGGGAGTGCTCCTCTTCCCGTCGGCGCCCATGGTGGCGCCCTACCACTACTCACTGCTGCTGCGCCCC

TTCAACTTCGCCTACTGGGGCGTGCTTAACGCGCTCAAGTTTCCCGCCGCGCAGGTCCCCCTCGGTCTGAACTCAGCCGG

CATCCCCCTGGGCATACAAGTGGTGGCGGCGCGCGGGCAAGACGCACTCTGCCTCGCCGTCGCTAAGCATCTCGACTCGC

TGTTCGGCGGCTACCGGCCGCCCTGCACCATACTGCACTGA

>Cobl_FAAH4

ATGGCTACATCAGATATTACATCGAGTAAACCGCCCGGGATGAGGGTCAAAGTGTTACATGTAGTTAGATTGGTCCTCGC

CGGTCTCGCCAGGTTGTTCTGTTCCCTGTACTATGGAAAGACGGGAGAGAAGTTTCCGCCGATAACCGCCGACATCCTGA

AACTGCCGGCTGTCGAGGTCGCAAGCAAGATCAGGAATAAAGAGATCACCAGTGTGGAAGTGCTGGAGGCTTGCAAACAA

CGCATCAAAGACATAAACTCGGCTCTCAATTGCTTTGTCGAAGATCGTTTTGAGCTGGCTTTGCAAGAGGCTAAAGAGGC

CGACGAACTCGTTCGAAGTGGGGCCATGACCGTCGAGCAACTGGCGAGAGAAAAGCCCTTCTTAGGCGTGCCTTTCACTA

CGAAAGACTGCATCGCAGTTAAAGGCTTACATCACACGTCGGGAGTCGTTCTTCGCAAAGATGTCATCGCTAAAGAAGAC

GCTGAATCAATAAAGTTGTTGCGAAATAATGGAGCCATTATTATCGGACTAACCAACGTGCCGGAACTTTGCATGTGGTG

GGAGACCCACAACCACATTCACGGGAGGACCAACAACCCCTACAACACGACCAGGATTGTTGGAGGCTCGTCTGGTGGCG

AAGGGTGCCTTCAGGGCGCCGCGGGCAGCCTGTTTGGCGTTGGTTCGGACATTGGAGGCTCTATCCGAATGCCAGCTTAT

TTCAACGGTATTTTCGGACATAAGCCATCGCGACTTATAGTTTCCAACGAAGGCCAGTATCCCACGCCCCAAACCGAGCT

TCTGAACTCATATCTAGGTCTCGGACCAATGACACGACATGCGGTTGATCTCAAACCCCTTTTGAAGATTATGTCAGGCG

ATAATGCAAGAAAACTCGACCTAGATAAAAGTGTCGACGTCGGGAAACTAAAAGTCTTCTATCAGTTGAGTAACAACGCT

CCCATGACGGACTCTGTTGACCCAGAAATCACGGCAGCGTTGAAGAAAGTTGTCGAATTCTTTAGTATCAAGCACAAAAT

ACAGGCTGAGGAGAAAAAGTTCAAATTTCTGCAAAAATCCCTCCCAATCTGGATGACTACTATGAAGAACAAAGAGCCTT

TCGAAAGCTTGATAATGGGAAAAGAAGGCACCGTAGCTATTGTTCTGGAAATCTTCAAGAATCTCATTGGATGTTCAGGC

AACACGATGATTGGTCTTTTCACGGCGTTGATGGACCGTTCTGGTGCAGAGATCGGCAGTGGCAAATACAACTACCACCT

CAAATTACGTGATAATTTAGAGAAGCTGTTCGTCGAGATGCTCGGAGACGACGGTGTGTTTCTGTACCCCACGCATCCGA

CGCCGGCCCCCTACCACAACGAGCCATTGATCAGACCTTTTAACTTTAGTTACACTGCCGTTATAAATTGCCTTGGCCTC

CCCGCAACGACAGTTCCCCTGGGGTTGAGCAGCGAAGGGCTACCTATTGGAATTCAAGTGGTAGCTAATCATAATAATGA

CCGGCTATGTCTAGCGGTAGCTGAGGAACTAGACAAAGCTTTTGGAGGTTGGGTGGAACCGAGGCACTGA

>Cobl_FAAH5

ATGGCGTGCACAACATTAAAATCATTTTTATGTTGCCTTCGGGTCTACATGGATAAAATGATAGATTTCTTCTTCGGGCT

GTATTGGGACGGCAAGAAAGTGTCCATACCGCCCTTGAGCAAGGAGCATGCATTTCTGGCTGAAAGCACTGTGACCCTGG

CGAGAATGATAAAAGAAAAACAGCTGAAGTCTGAAGACTTGGTCAGAGCTGTCATTAAGAGGATAAGAGAAGTAAACAAC

TTAGTGAACGCTGTCATCGAAGAGCGGTATCACGAGGCGATAGAAGACGCGCGGGCGGTGGACGACCTCATAGCAGCTGG

GCTGCCTGAGGAGGAAGCTGCCAAGAAACCGTTCTTAGGTGTTCCCTTCACGACCAAGGAGAGCCAGGCCATCAAGGGTT

TCCGCTACACCATGGGCCTCTGGTCGCGTCGGGAGATGCGCGCCGACGAGGATTCCGAAGCCATTGTCAGGCTCAAAGCA

GCCGGCGCCATCCCCTTGGCTGCTACTAACCTGCCTGAGCTTCTGATCTGGCAGGAGACCCGTAACCCCGTATACGGCAT

GACGAACAACCCCCACCACGCGGGCCGATCGCCAGGAGGCTCCAGCGGCGCCGAGGCAGCCCTCACTGCCACCTACGCTA

CACCTATCAGCTTGTGCTCCGACTTGGGAGGATCCACCCGTATGCCCGCTTTTTACTGCGGAATGTTCGGTCACCACGCG

ACTGCCGGAACCACCAATGGGAGAGGAGTCATTTTCCGCAAGGGTGACGAGGAATCTATGCTCAGCTTAGGGTTTATAAC

TAAACACGTGGAAGACTTGGCACCTTTGACCAACATTATTGCTGGGGAAAAGGCCCCACTTCTCAAACTCGACCGCAATG

TCGATATCAAGAACATAAAGTTCTACTACTTGGAGTCTGCTAACGATTGTCGGCTCAGTTCCATCAGGCCAGAGCTCAAG

GATGTTATGACGAGCGTTGTATCAAAGCTCGGCAAGGAAATGCCTCCTCAGAACAGCCCTGAGGCTTACCAGCACGAAGG

GTTCGATCACATGTACCAGCTGTGGTCGTACTGGATGAGCAAGGAGCCCGAAAACTTCGCCAGCCTGTATAACAACTACA

AGGGCGAGGCCAACGGATTTGTGGAGCTGCTCAGAAAGATCTTTTGCCTCAGCAAGCACGGACTGTCCGCTGTCATCCGG

TTATTTGAGATCCAGATCATGCCGCTGTTCCCCGCCTGGGCAGACAAACTCACTACTGAACTGAAACAAGATCTTTTTAG

TAAACTGGGTGACAACGGTGTCCTGTTATTCCCGAGCGCCCCTCACCCCTCCCCGTACCACTACTCTTGCTTCTTGCGGC

CGTACAATTTCTCTTACTTTGCCGCCGTCAATATGCTCAAGTGTCCTTCTACTCAGGTGCCTCTGGGTGTGAACAGTGAT

GGTCTTCCACTTGGCATTCAGGTGGTAGCAGCACCCTACAACGACGCGCTCTGTCTCTCCGTCGCCAAGTATCTAGAAAA

AGAATTCGGCGGCGCCGTCATGGCTTGTAAAAAGCAATAA

>Cobl_FAS

GCCTTCCTGCGCGAGCGGTTCCCCGCGCTGCCCGAGGCGCACGTGGGCAACTCGCGCGACTGCAGCTTCGAGCAGCTGGT

GCTGCGGCGCACGCGCGGGCGCGGCGTGGACCTGGTGCTGAACTCGCTGGCCGGCGACAAGCTGCAGGCGTCGCTGCGCT

GCCTGGCCGTGGGCGGCCGCTTCCTGGAGATCGGCAAGCTGGACCTCAGCGCCAACTCGCCGCTCGGCATGGCCGTGCTG

CTCAAGAACACCACCGTGCACGGCATCCTGCTGGACGCGCTGTTCGACGCGCGCGGCGACCACCCCGAGAAGGCCGAGGT

GGTGCGCTGCGTCACCGAGGGCATCGCCCAAGGCGCCGTGCGCCCGCTGCCCGCCACCGTCTACGCCGACTCGCAGCTGG

AGCAGGCGTTCAGGTACATGGCGACGGGCAAGCACATCGGCAAGGTGCTGATCCGCGTGCGCGAGGAGGAGGCGGGCGCG

CGC

>Cobl_FAT1

AACGACGGAGGGATCATTGAGCGCCGGCTACGTCCTATTTACGATTGGCTGGACAATGGAAATAATAAGAAAGCGCTGCA

AGAGGCGGAGAAGGTGCTTAAAAAGAGTCCTTCGCTGCAGGCGGCCCGGGCGTTGAAGGCTCTGTCGCTTTTTAGGTTAG

GAAAAGCACCGGAGGCCCACGGCGTGCTAGAAGCCCTAGCGGACGAGAAGCCGTGTGATGATACTACGCTGCAGGCCATG

ACGATATGCTACAGGGAGTGCCAGCAATTGCACAAAGTGTGCTCCCTGTACGAGGCGGCGGTGAAGGCCGACCCGACAAG

CGAGGAGCTCCACTCCCACCTGTTCATGTCCTACGTCCGTGTCGGGGACTACCGGGCACAGCAGCGGGCCGCGATGGTGC

TCTATAAGTTTGCACCTAAGAACCCGTATTACTTCTGGGCCGTGATGAGCATTGTGTTGCAGGCGAAAGAATCCGAGGAC

GCCGCGAAAAAGGGCATCCTTCTCGCTCTAGCGCAGCGCATGGTCGACAACTTCATATCCGAGAACAAAATGGAAGCCGA

ACAGGAAGCCCGACTCTACATCATGATCCTAGAACTGCAGGAAAAATGGGAGGATATCCTTAATTTCATAGAGAGCCCGT

TGTACGCGAAACTCCTCCCTGGAGCCACGGCCCAGGCCTGCATACCTTACTTGAAGAAACTCCAGCAGTGGCGACGTCTC

AACCTCATCTGCAAGGACCTTCTCTACGACAATCAAGACCGATGGGACTACTACGTACCCTACTTCGAATCAGTCTTCCA

ACTGATGAAGTGTTCGGACAAGAACGACTTGACCGTAGACGACACGGCTGAGAAATGTCACGAGTTTATCTGTCAATTAG

TGGAGAGTATGTCGTCTGGGAGGGTGCTGCGGGGGCCTTATTTAGCTAGACTGGAACTGTGGAAACGTTTGTCTGTGGAT

GGTGATCCAACGGAGCTCCTTGGTAGCGGGGTGGCGTTGTGTGTGCAGTATTTGCGGGTGTTCGCGAACAAACCGTGTGC

CGTGCCGGATCTGAGGCCGTATCTTGAGATGATACCGCAGAGCGAGAGAGAGGAGCATTGCAGGGATTTCCTCACGTGTC

TCGGGTTCGATGAGAATAGTGAGCCTAGTAATCCGGACGACATCCAGCGCCACATCTCCTGCCTCTCGGCGTGGCGGCTG

ACGGCGGCGCCGCTGCCCGCG

>Cobl_FAT2

TGCTTGCCAGAAAATTATCAGATGAAATATTACTTCTACCACGGACTATCCTGGCCACAGCTCAGCTACGTAGCTGAAGA

TGAAAAAGGACACATTGTTGGCTATGTGCTGGCTAAGATGGAAGAGGATGGCGAAGACAACAGACACGGGCACATTACAT

CTCTTGCTGTGAAGCGGTCGCATCGCCGGCTGGGGCTCGCCCAAAAACTTATGAACCAGGCTTCACTGGCTATGGTGGAA

TGCTTCCAGGCAAAATATGTGTCCCTCCACGTCAGGAAAAGCAATAGGGCTGCGTTGAACCTCTACACTAACTCGCTAGG

CTTCAAGATCCTGGAGATCGAACCCAAGTACTATGCTGATGGCGAAGACGCTTACTCAATGATGAGGGACTTGAGTTCAT

TCTCCGCAGACAGCAAACCAGAGACTCAGGCGTCAGATAATTTGGAAATCAAATCTGATTCTGCTGTTATATCGCAGTGT

TGA

>Cobl_FAT4

ATGCCACCAAGTAATCCTTTACCGCCTAAAGAAAATGCGCTATTTAAAAGAATTTTGCGTTGCTACGAACATAAACAATA

CAAAAATGGCTTGAAATTTGCCAAGCAAATCCTGTCCAATCCAAAATTTGCGGAGCATGGAGAAACATTGGCAATGAAGG

GCCTAACACTAAACTGCCTTGGCCGCAAAGATGAAGCATATGAGTATGTCCGCCGAGGCCTCCGTAATGATCTCAAGTCC

CCAGTTTGCTGGCACGTTTATGGTCTCCTGCAACGGTCTGACAAGAAATATGACGAGGCCATCAAATGTTACCGGAATGC

TCTCAAGTGGGAAAAGGAGAACATACAGATTCTTCGAGATCTCTCTCTCCTGCAGATCCAAATGAGAGATTTAGAAGGAT

ACAAGGACACCCGCTACCAACTCTTCATGCTGCGCCCAACACAGAGAGCATCATGGATAGGCTTCGCGATGAGCTACCAT

CTCCTCGGAGACTACGAGATGGCCAACAGTATCCTGGACGCCTTCCGTACCAACCAGATGAAAGGCCCGTACGATTACGA

GCACTCGGAACTGCTGCTGTATCAAAACATGGTGTTGGCTGAGTCTGGGCAGTTTGATCGGGCGCTCTCGCATCTACACA

AGTTCCAGACGCAGATACTTGATAAGCTATCGGTGAAGGAGACTAGTGGCGAATATTATTTGAAGTTAAAGAGGTTCAAA

GAGGCTGAGGCGGTGTACGAAGACTTACTGAAGAGGAACCCTGAAAATGTTATGTATTACCATAAACTTATAGAAGCTAA

ACAACTAAGTGATCCTGATGAAAAAGTGGCCTTCTTTGATGTATATAAGAAAGAGTATCCCAAAGCAATAGCGCCCCGTC

GGCTGCAGCTCTCGGCGACGGGCGCGCCGGCCGCGTTCGCGCGCCTCGTGGACGAGTACCTGCGGCACGGGCTGCACAAA

GGCATCCCTCCGCTGTTTGTTGACATTAGATCCCTATACACGGACAAAGTCAAAGCGGAAACGATAGAGAACCTAGTCCT

CCAGTACATAGAGAATTTATCCAAGTCCGGAGCATTTAGCTCAGACCCGAACGAGGTGAAACAACCTGCCAGCGCTCTAT

TGTGGGCTTACTACTATGCCGCCCAGCACTTTGACTATAAGAAGGATACGGACCGGGCTCTCAAGTACATTGACGCGGCG

ATAGAACACACGCCGACTTTGATAGAGCTATTTATTGTTAAAGGAAGGATATTTAAGCACGCCGGCGACCCCATATCCGC

GTACCAGTGGCTCGAAGAGGCGGCGGCGATGGACACGGCCGACCGCTACGTCAACAGCAAGTGTGCTCGCTACATGCTGC

GCGCGGGACACGTGGCGCGCGCGGAGGAGATGTGCGCCAAGTTCACTAGAGAAGGTGTGCCAGCGACAGAGAACCTGAAC

GAGATGCAGTGCATGTGGTTCCAGACGGAAGCGGCGACGGCGTACCAGCGCGCGCAGCTGTGGGGAGAGGCTTTGAAAAA

GGCGCACGAGGTTGATAGACACTTCTCCGAAATAATGGAAGACCAGTTCGACTTCCACTCCTATTGCATGCGCAAGATGA

CACTCCGTTCCTACGTGGGCCTCCTCCGGCTCGAGGACGTTTTGCGCGCTCATCCGTTCTACTTCCGCTGCGCGCGCGCC

GCCATACAAGTGTACTTGAGACTAGACGCTTTCCCGCTGCAGGATGTCCCACAGACACAGGAGCCCGATACAGAGAACCT

GGCGCCGTCCGAACTGAAGAAGTTGAGGAATAAGCAGAGGAAAGCGAAACGCAAAGCTGAGCAGGAGAGCATGCTGGCCG

CACAAGTTCAGGTGAAGCGCGAGCAGCACCACAAGGCGCGGCAGCAGCAAGAACAGGGCGACCCGGAGGCGCCGCAACTC

GACGAACTCATACCCGACAAGCTGGCGAGGGCGGACGATCCTCTAGAACAAGCGATAAGATTCCTGCAGCCGCTCCGGAC

GCTCGCCAACGATAGGATAGAAACACATTTAATGGCCTTCGAAATATACTATAGAAAAGAGAAACCCCTCCTCATGCTGC

AGAGCATAAAGCGCGCGTGGCGGCTCGACAGCGGCCACCACCACCTGCACGACTGCATGGTGCGGTTCCGCGGCTTTCTG

GACGAGCACCCCGACCAGCCCGCCGCCGTCGCGGAGGTGGTCGAGAAGGAGACGCGGGAGTCGGTCCGCGGTCGCACAGC

GCGGCAGATGGCGGAAGAGTTCATGACTCACTCGGCGCAGCAGTCCCAGAGCGCAGCCCTGTGGGGCGCGCGCGCGCTG

>Cobl_FAT5

ATGGCTGTTACGGGTGCCAGGAGTATTGTGAATCTGAAGAAGTCCCACTTCGAACATGAGAATGCTAGGATCGATAAATA

TACGCTGGCGGACGAGCTGCCGGCGCAGCTGTCGGAGCTCGGCCTGCGCCCGCGCAGCATGCAGAAGCCCGGCTACACGC

TGCGCAAGCTCTCCGCCAGCGACAAGGAGATCACGCTCGAGTTCTTGAGACGGTTTTTCTTCCGCGATGAGCCCATGAAC

TTGGCAGTCCAGCTGTTGGAGACAGCAGACTCCCGCTGTCCTGAGCTGGAGGACTATGCGTCCTCGTCTCTGACTGATGG

CGTGTCCGTCGCTGCCTTCGACGACCTGGGAGAGATGGTTGGAGTGGTCGTGAATGGGGTCGTCAGGAGAGAGGAAGCCG

ACTACACGGACAAGTCCGAAGAGTGTCCCGACCACAAGTTCCGGCGGATCCTCAAGGTGCTGGGACACCTGGACCGCGAA

GCCAAGATCTGGGAGAAGGTGCCTGAGAACTGCAACAAGGTGCTGGAGATCAGAATAGCGTCGACACACTCTGACTGGCG

CGGGCGAGGGCTTATGCGGGTGCTTTGCGAAGAGACAGAACGCTTGGCCCGTGCGATCGGCGCAGGCGCCCTCCGCATGG

ACACGACATCGGCGTTCTCCGCCGCCGCCGCTGAGCGGCTCCACTTTCGCAGCGTATACTCAGTGTTGTACGCAGACCTG

CCAGACGCGCCGCAGCCGGAGTCTCCGCATCTAGAGCCTAGAGTCTACATTAAAGCTCTGTGA

>Cobl_FAT6

ATGACGACAATAAGACCATTTACATGCGAAGATATGTTGAAATTTAACAATGTGAACTTGGATCCATTAACAGAAACCTA

TGGTTTATCTTTCTACACACAATATTTGGCTCATTGGCCGGAGTATTTTCAAGTTGCGGAATCGCCAAGCGGTGAAATTA

TGGGCTACATTATGGGGAAAGCAGAAGGTCTCGGTGAAAACTGGCATGGACATGTAACTGCCCTGACTGTGAGCCCTGAT

TACAGAAGACTGGGCTTAGCTGCCACTTTGATGAGCATTTTAGAAGATGTATCGGAAAAAAAAAAGGCTTACTTTGTGGA

TTTGTTCGTAAGAGTGAGCAACAAAGTAGCCATTAACATGTACAAGAACCTGGGATACATTGTTTACCGGACTGTTCTGG

AGTACTACTCCGGTGACCCTGATGAAGATGCTTATGACATGCGGAAAGCCTGTTCCAGAGATGTGAATAAAACTTCAGTT

ATACCATTAGCACATCCTGTTAGACCTGAGGAAGTGGACTGA

>Cobl_FAT7

ATGCCTGTGCAGGTGTACCCGAGCCCGGGGGCGGCGCGCGGCGTGTGGGCGCTGCCGCAGCCGCCCCCCTGCAGCATGGC

GGCAGAGGACTGCTTACCGCCCTGTTCGCCCCTCTCCCCCGATGCGCCCGCCGCACCGCCCCGTACCATCACCAGCTTTT

ATCACCACTCGGATCTGCATCAGCTGATCTTTGAGGCTGTGAGGTCCAGCGAGGTGTCAGAGATCGAGCGTCTGGTGGAG

AAGCTGGGTGCAGAAGTACTAAGCGCACGCGATCAGCATGGATATACGCCTGCGCACTGGGCCGCACTCGATGGCAGCGT

TGCGGTCATGCGTTACTTGGTGGAACGTGGCGCGCCTGTTGACTTGTCCTGTCTGGGCACTCAGGGTCCTCGACCAATCC

ATTGGGCCTGTCGCAAAGGCCATGCCTCAGTGGTGCAAGTGCTGTTACAGTGCGGGGTCGCAGTAAACGCAGCTGACTTT

AAAGGCTTAACCCCCCTCATGACAGCCTGCATGTACGGCAAAACCGCTACAGCAGCGTACCTCCTAGGCATGGGAGCAGC

CACCAGACTCTCAGATATCAATGGAGATACCGCCCTCCATTGGGCTGCGTACAAGGGCCATGCAGATTTGGTGCGACTGT

TGATTTATTCTGGAGTCCCGCTGCACTGTACGGATAACTTCGGCTCTACACCGCTGCACCTGGCTTGTCTGTCGGGGAAC

CTGACGTGTGTGAGATTGCTGTGCGAGAAGGTAAAAGCAGAGCTAGAGCCTCGCGACAAGAACGGAAAAACTCCCCTAAT

GTTGGCCCAAAGCCACCGCCACGCAGAAGTAGTGAAGCTACTGACAAAAGAGATGAAACGCAAGTCCCACTGGATGCCGC

CGCTGTCGGAGTTGTGGGCTCTGCTGTTTGGCGGCGCTGGGGATTCTAAGGGGCCATTGCTGTTTTTCTTGGTTTCTGTG

CTGCTGTGGGGGTACCCTATGTATGTTTTTAGATGTATCCCCCTAACCTGGACAACGCTCCGCCTCTCCCACTACTGCTT

CCTCTACTGGAACGCGATCATGTGGCTGAGCTGGGTTATCGCCAACCGCCGCGATCCCGGATACATCCCGCAGAACTCAG

ACACGTACTACCGCGCCATCCGGCAGATACCGTACTACGACAAGTGGAAGAAGCGAAACGTGGTGTTGTCTCGCTTGTGC

CACACTTGCCGCTGCTTGCGGCCGCTGAGAGCCAAGCATTGTCGCATCTGCAAGCGCTGCGTCGCGTACTTCGACCACCA

TTGCCCCTTCATCTACAACTGCGTCGGCGTCCGCAACCGAATGTGGTTCTTCCTGTTCGTGATGAGCGTCGCCATTAACT

GCACGCTGTCCATCTACTTTGCGTGCTACTGTCTGCTGCTGGAGGGATTCGGGCTGCTGTATTTGCTGGGGCTGCTGGAA

GCACTCACGTTTTGCGCGCTCGGATGGATTTTGACGTGTACTTCTATCCTCCATGCCTGTATGAACTTAACGACCAACGA

GATGTTCAACTACAAAAGATACCCCTATTTAAGAGACAAGCGAGGCCGATACCAGAATCCCTTTTCTAGGGGTCCTATAA

TGAATTTATTCGAGTTTTTCGTCTGCCTACCAGATAAGTGCGACGAGCAAGATTTCTTCCACGAGGAGAGTATTTAA

>Cobl_FAT9

ATGGGAAGACAAAGCTTAGAAAATGGCTGGGACATGACAGGTCGCTGTCAAAACACAGAGTCAAACAATTTGCTCAAAAA

TTGGAAAATTGTTGGAAATATTTCCTTTAGTGTATACCTATACCTATTCAATAAAAACATTTACATCTATCATCAAGCAA

TGAGTAACCCACATAATACCAGCCAGCTACCGGAATGTTTGAGGGAGGATGAAAATGCAGATGGGAAGACAAAGCTTAGA

AAATGGCTGAACATGAATTTCAGAATTGAAATGACAGACGGAAGGGTCCTTATTGGTGTCTTTCTGTGTACAGATCGGGA

TGCTAATGTGATATTAGGTGCTTGCTCAGAGTATCTCAAGGGCAATGACGGTGAAACCGAAGAGCCCAGAGTGTTGGGTC

TCGTCATGGTCCCCGGCCGCCACATAGTCTCCATACAAATAGACGACATGACCGCACATCGCAGAAGTGCTCCCATAATG

AATGACTCTATGTGGCTTTAA

>Cobl_FAT11

ATGGAACCAGAAATACTCGAAGTGCTGCCGCTACATAATTATCCTGAGTTTATGAAGGATACCTGCGACTTGATTAATGA

TGAGTGGCCCCGCGGCGAAAACGCCCGGCTGATGTCCCTACAAGCCTCGTGTGACTACCTACCGACAAGTTTTATTTTAG

TTAGAGATAGAAAGCAAGTATTAGGCCATTGTAAACTAACCGCTATACCCAGTATTCCAGAAAGTTGTTTTATAGAAACC

GTAGTCATAAGTAAAGCTTTGCGTGGGAAGAAACTAGGAACTTATTTGATGAAACACGTCGAAGATTACTGCAAGAATGC

ACTGAAACTAAAGATGGCGCATTTGTCGACGAAGGGCCAGGAAGAGTTTTATGCGAAACTTGATTACGAGATTTGCGCAC

CAGTGTCCATATACGGCGGTTATTTGCCAGCCAATTCTAAACTTAGACCTAGTAGCCATACGATTGAAAATTCAGTGCCA

GAAATGAGCGTGCCGGTTAGCGGCGCGCCGCCGCCACCGCCGCTCCCACCGACGTACGAAACTGCTTGGAACAATAATAA

TAACAACACCATAAAAACTACCAAGACCTACATGTTTAAGTATTTGTAG

>Cobl_FAT12

GTGAAAGCGCCAACGCAAGACGACTTGACGGACCTCGACGAGATCGAGCGCGAATTGAAAATGCTCGACGAACAGATACT

ACTGATGACGCAGGTCGACGAGCCCGCGCCTGCTAACCTCCCGCAAGCCAGCACAGCCCCTACAACGGAGGCTGTCAAGG

ACGACACAGCTGACAAGCTGTTCCCTATCTTCAGCAAACCTAACCCTGGGACCAGCACACCTAGCTCTAAAGGCACAGCT

ACCAAAAGCAAGAAGTCGATATTGAAGGACGGGAGCGATCAATACGTGATCGACGCAGGACAGAAGCGATTTGGAGCGAC

TCAGTGCACAGAGTGCGGTGTTATTTACCAGATTGGCGATCCTCAGGACGAGCACTATCATTTGGTGCACCACAACGCGA

CGGACGTACTTAAATTCAATGGCTGGAAGGAAGAGTGCATAGTGGGCCGGTGGGGCGCGGCGCGCTGCGTGCGCGTGCGC

GGCGGCGAGCCCAACTGGCGCCGCGTCGCCGCGCTCCTGACGAGGGTC

>Cobl_FAT13

GAAGGGCATGCGGGGCTCGGGCGCGGGCATGAGTCGCTCCGGTTCCCAGCGGGGAGGCGTACGCGTGCGGCCAGAGACAC

ACAGCGAGCCATGTCTGCGACCGACGCCGTCACCATCCGCCTGGGGCGCGCAAACGACATGCCAATCGTCTTCGAGATGA

TAAAAGAGCTAGCAGCCTACGAGGGTGTTCCTGATGGACCGAAATTATCTCTGGAGGATCTAGTGGAAGATGGTTTCAAC

AGTAGCCATCCATGGTTCTTTGTCCTGCTGGCGGAGAAGAACGGCGAAGTGCTGGGCTCGGCGCTGTGCAACCGCGCCTA

CTCCAGCTGGTCGCGGCGCGCCTTCTACATCGAAGACCTGTTCGTGCGGCCCGCCTCGCGGCGCATGGGCATCGGCCTGC

TGCTGATCAAGGAACTGTGCAAGGCGGCGGTGGAGGAGGGCGTACACCGCGTGGATTGGCACGTGCTGAAGAGCAATGCG

CCCGCGCTGGCCTTCTACAACAACATCGGCGCGCGCGACCTGCGCGTCACGGAGGGCCGGACGGCGCTGCGCCTCGACCG

AGACCGAATCGAAGCCCTAGCTCAGGACGGCTAG

>Cobl_FAT14

ATTCTAACGTTGTTTTGTTTATTATCTGAAAATAATTTGTTAAAAATGGTGAAAAAGAAGATTGATAACCGTATTAGGGT

TATGATAGAAAATGGAGTCAAGCTGGGACACCGTACAATGTTCCTGCTTGTCGGAGATAAGAGCAGAGATCAAGTGCCAA

TTCTATATGATATTCTCGTCAAATCAACTGTCAAATCCCGACCTACGGTTTTATGGTGCTACAAAAATAAAGATGAAGCT

ATAAGCAATCATGGCCGCAAACGGGCTAAGAAAATAGCAGCAGGAAAACTGGAGGTGTCAGAAGAATCTCTGTTTGATGC

GTTCAGAGTGGCGACTACGATACATGGGCGGTATTACTCTGAGAGTCATGCTATGCTCGGTCAGACCTATGGAGTGTGTG

TGTTACAGGACTTTGAAGCCCTCACTCCTAATCTGATGGCTCGAACAATAGAGACAGTGGAAGGTGGTGGACTCATCATC

TTTCTGCTAAAGACCATGGATTCTCTACGACAGCTGCATTCCATCACCATGGATGTGCATTCAAGGTTCAAAACAGAAGC

CCATGATACAGTAGTAAACAGATTCAACGAGCGGTTCCTATTGTCATTAGCTGACAACCCCCGTTGTTTGATTCTTGATG

ATGCACTGACAGTGCTTCCAATATCTTCGAAAACTGCACAAGTGGAACCAGTCAATGAGACACCAGAGCGTATCAACCCG

AAGCTGACAGAGCTAGTGTCGTCCCTCTCCGACTCGCCGCCCGCCGGCCCGCTGGTCGCTCTCTGTCGCACCTACGACCA

GGCGTCAGCGCTTATCGCTATCATAGACACACTAGCTTCCAAAGCATCCAGGCCTCCCCACTGCCTCACAGCGGCCCGGG

GCCGCGGCAAATCAGCCACTCTAGGCCTCGCGGTCTCAGCAGCAGTGGCTTTAGGCTACGTCAACATATACGTAACCTCT

CCCCACCCAGAGAACTTAATAACCTTCTTCGAGTTTGTCCTGAAAGGCCTTGACGTCTGCTTGTACCAGGAGCATATCGA

TTACAGTATACTGAGGTCGACGAACCCGGATTTCAAGAAGGCTATTGTTGGGATTAATGTGGCTAGGAATTCGAGGCAGA

CTGTGCAGTACATAACCCCTGACGATCACTCGCTCCTAAGCGCCGCAGACCTAGTACTAGTAGACGAAGCAGCTGCCATC

CCAATAGCTCACGTCTCCGCGGCCGCTACCAAGGCACCGCTGGCTTTGCTGTCGTCAACTGTCTCTGGGTACGAGGGCAC

GGGACGAGCACTATCTTTGAAACTGTTTGCTATGCTGCAGACGAAACACGATGCACCGGCTCCGATAAAACTAGAAGAGC

CAATCCGCTACCGCGCCAACGACCCGATAGAGGCGTGGCTTAACGCCCTCCTCTGCCTCGAGTCCCCCGCCCCCTCGGTG

GGCGTGGGTTCCCCCTCCCCCCACTCCTGCGAACTGTATAGAGTCAACAGGGATGCCCTGTTCTGCTATCATAAGGCGGC

AGAGGCTTTCTTGCATAGGCTGGTGGCTATTTATGTTGCGAGTCATTATAAGAACAGCCCTAACGACTTACAACTCCTGG

CGGACGCACCAGCGCACTGTCTGTTTGTGCTACTAGCGCCGACACCGCCCAACGCCACGTCTATGCCCGAAGTGCTCTGC

GTGATACAAATGTGTCTGGAGGGGAATATCTCTGACAAGTCGGTTCGCCACAATCTCGGGCGCGGTCGCAAGGCGGCGGG

CGACCTGATCCCCTGGAACATCTGCGAGCAGTTCGGGGACAAAGACTTTCCCAAGCTGTCGGGAGCCAGGATCGTGAGGA

TAGCCACGCATCCCTCGTATCAGCGGATGGGCTACGGCAAACGCGCTCTGCAACAACTAGCGGCGTACTACTCGGGAGAG

ATCCCGTATCTGGACGACGCGCAGCCCGACAGCGACGACGGTAACGACCGACGCAGCGACACGCTGCACACAGAGACCAT

AGTGCCTAGAGCGAAACCACCAACTCTTCTGAAGCGGCTGTCAGAGGTGCAAGCAGAACATCTCGACTATCTTGGAACCA

GTTTCGGTCTCACTGAGGACTTGCTCAAATTCTGGAAGTCACAGAAATACGTGCCCGTGTATGTAAGTCAAAAAGCCAAC

GAGCTAACCGGCGAGCACTCCTGCATAATGCTCCGCTCTCTGTCTGACGGCGCCTGGCTAGTCGCCTACAGCGCAGATTT

CCGCCGCCGCCTGGCGCGTTTGCTCGCGCGCGCGTTAAGGACGCTGCCGGCAGCGTTAGCGCTGCCCACGCTGGTCAATG

ATAGCGTCGCTGTTAAGAAGGCTGCGTTAACAAAAGAGCTAATATCCCAACACCTGACAAACCACGACTTAGCCCGACTA

GAGTCCTACTGCCGGCAGCAAGCCGACTACAGACTCATCACGGACCTCCTCGCGCCCATCGCCGGCCTCGTGTTCCAGGC

CAAAGGGGCCAAGCTGGACGCTATTCAGCAGGTCATATTCGTAGCAATGGGCTTCCAAATGAAGGAACCTGACGACATAG

CGTCCGAACTCGGCCTCCCCGGTTCACAGATCCTAGCCAAATTCTACGAGGCCTGCAAAAAGATCAACGCTAGCTTCAAC

TCAGTCGTCGAACAATCGGCAGCCAAGGAGATCGGTATAGAGGATATGTCGGCCGAGTTGGAGGGAAGCGGGCCCGTCAA

ACAGAGCCTGAATGAAGAACTTAACAAGGCTGCTAAGGAGTTGGAGCGGAAGCAACGCAAGGAACTGTCCAAGCTGATGG

GCGAGGATCTGGCTCAGTACCGCATCAAGGGTTCCGATCAGGATTGGGGGCAGGCGCTTGCAGCTACTAAGGCTAAGGCG

CTGGTGTCCGTTAAGAGCGGTGAGAAGCGGCTTGGCGATGACAGCAAAGAATTAGACGACCTGATGGAGCGAGACAACAG

CAAGAAGAAGAAGAAA

>Cobl_FAT15

ATGGCTGGCTTCAGCTGGTACCTTTCTGAAAGCTTCCAAGTAATCATTGAGAAGTCGAAAGATGAAAAATGCTCCTTACA

AGATGTGCAATTACGTTTCTTGTGCCCTGACGACTTAGAAGAGGTACGGTGTCTATGCAGAGAATGGTTTCCCATAGAAT

ACCCGCAGTCTTGGTACGAAGACATCACATCATCAGAGAGATTCTTCGCTCTAGCCGCTGTCTACAAGTCGCAGATCATT

GGCCTTATTGTGGCAGAAATCAAGCCTTATCTAAAACTGAATGCCGAGGACAGAGGCATATTGTCAAGATGGTTCGCCTC

AAAGGACACCCTTGTCGCGTACATACTGTCTCTAGGCGTGGTCCGCTCGCACCGGCGCTCGGGCGTAGCAACGATGCTGC

TGGACGTGCTGATGCGGCACCTGGCCGGCGCGGCGCCGCGGCCGCCGCACGACCACCGCGTAAAGGCCATTTTCCTCCAC

GTCCTCACCACCAACACCGAGGCAATACTGTTCTATGAGCAGAAAAGATTTCGCCTCCACTCGTTTCTCCCCTACTACTA

CTCCATCAAGGGCCGCTGCAAAGACGGCTTCACATACGTATACTACGTGAACGGGGGCCACGCGCCGTGGGGCATCTACG

ACTACGTGAAGTACGTGGCGCGGGCGGCGTGGCGCGGCGGCGGCCTCTACCCCTGGCTGTGGGGCAAGCTCCGCACTGCA

CTCACCATCGCGTGGCACAGGAATGCCTACAGAATTTAA

>Cobl_FAT16

ATGAAGTTAAATTATTGTACCAAAATAGTTGGTGAACAAATCGTATTAGTCCCGTACAGAGATTTACATGTCAGAAAATA

TCATGAGTGGATGAAATCTGAAGAACTCCAAATGCTAACAGCATCAGAACCTTTAACGCTGGAACAAGAATTTGAGATGC

AGAAATCCTGGAGAGAAGATAAAGACAAATGTACATTCATTGTATTGGATAAAGCCAAGTTTGAAGATTGTAATGAAGAA

GTTGATGCAATGATTGGTGACACCAATATATTCATCACTGATAAAGAGACCTCTACAGGAGAAATAGAAATAATGATTGC

AGAGAAGAATGCCAGGGGCAGAAAAATAGGATGGGAAGCTGTCATCTTAATGTTACTGTATGGGATCAAGCAAATAGCTC

TAAAGAACTTTGAGGCTAAAATTTCGATGAAGAATGTAATAAGCATCACAATGTTTCAAAAATTACTCTTTAAGGAAACT

TCAAGAAGTGAAGCGTTTCAAGAAATAACACTTGCAAAGGCAGTTGATGAGGATTGGATAAATTGGTTAGAAGAACATTA

TAAATATCAAATACAGCCACATTAA

>Cobl_FAT17

ATGTCGCAACCACCATACACATTGGAGCCGGTGGAGAAGGGAGACGAAGAAAATATCATGAAACTGCTCAAAAAGACATT

CTTCATCGACGAGCCCCTAAACCAAGCAGTGGGTCTCATAACCTCAGAGAACGAGACCTGCACAGAACTGGAGGAGTACT

GCACGCATTCCCTGATGGAGGGGCTGTCCTTCAAAGCGGTGGACAGCCAGCACCATATAGTGGGGGTCATGATCAGCGGG

GTTATTCCCCTTAAAGAAGACACCATAAACGGCAACGATCTTCTAAGCCAAGCGCAGCGGTGCAAGAACCCGAAATTCCA

GAAAATCCTATACATCCTGGCGCGAAGGGAGGCGGGAGCTCGTCTCTGGGAGAAGTACCCGGAAGAACAGAACCTGGTCG

AGATCATGGTGGCAGCTACTGACCCTGGCTGGAGGAGGCGGGGGATTATGAATGAGTTGCTGAATAGGACTGAACAAGCC

ACAGCGCAGCGAGGCATCCGTCTCTTACGCATGGACACATCGAGTGCGTACTCCGCGATGGCCGCAAAGAGGTTCGGATA

CACGAATGTGTACAAGGCTCTATACACGGAGATTAAGATGGACGGACGGCCTATTGTAGTGCCTGAACCACCGCATGTGG

ATGACAGGGTTAATATTAAAAAGTTATTTGATTAG

>Cobl_FAT18

ATGAATCAACTCGCTGACGAAAATGTGCAGAACGTCAAGAGTAGTCTCAAAACGACGAGCACAAAAAACAAAAATAAAAC

CGCCAAAGATAACTCACCTTCTAAAACGACTGACGAGGTAAGAATAGTAAATGAAGAGAAATTAAGTGCGGCTCTAGCTA

GTACTTTACACGTTAACAACATCAATAGCAACCTAACAAATGGCACGAGCGACCACTCGAGAGAGGAAATAGCAACGTCC

GACAATGACAAAGCGGGCGACGAAGGCCCTAGAGATGCAAGCAGTGTACAGCCAAGTGATGCCAGCAACGGGGACAGCAG

AGGGACATGTGCTTCTCAAATGAATATGGTCAGATACATCAGCGAAGAGACTCTAGAAAACCTTGAGAACACTTTCGACC

TGCAAGGTCAGTCCTCGCAAGAAGAAATTGAAATCATATCATATGAATCTGAACTGCAAATGCCTGAAATCATGAGAGTC

ATACAGAAGGATCTATCAGAACCATACTCAATCTACACTTATAGATATTTTATACACAACTGGCCTAAGCTTTGCTTCTT

AGCGACTCATGGTGAGAAGTGTATTGGTGCCATTGTATGTAAGCTAGACATGCATCGAAATGTGGTGAAAAGAGGATATA

TTGCTATGCTAGCTGTGGATGAGAAATACAGGAATAAGAAAATTGGTTCAAGGCTGGTACGGAAAGCAATACAGGCAATG

ATTAACGATAATGCAGATGAAGTAGTCTTAGAAACTGAAATAACGAACAAACCAGCACTCATACTTTACGAGAACTTGGG

GTTTGTGCGTGACAAGCGTTTATTTCGATATTACCTCAATGGTGTGGATGCGCTGCGATTGAAGCTATGGTTAAGGTGA

>Cobl_FAT19

ATGGCGAAGATGACAGAAGCCGAGATCCTGGCCAAAATGAAGGTGCTGGAAGACCGGATCAAGGCGCCTTCGATCTGGGG

CCGCGTGCCCTGCGGAGTCCGGTTTGAGGATCTGCAGGAGAAGCGGTACGAAGACACTGTAAGGCTGCTGAAGAAGCACT

ATCTACCTGAGGAGCTAACATACAGATCAGTGAAGATAGCCGACGACAAAGAAGGTACCGACGAGTTCACCCACAACCTG

AGGATATGGATGAAAGACAAGATGTCCATCGCTGCCGTGAAGGAGGGGACCGACAAGCTGGTGGGCTGCCTCATCATGAG

GATACAGGAGAAACACGCCTTCTCCCGAACCTTCAGCCGGGTTAAGATAACCTACAATCCGCAGTACAGCACAGTGATGA

CGTTCTACAACGCCGTCGAGAAACCAGTGGACGTGTACGATAGACTCGGCGTGAGAAGATACTTCAAAGTTTACCTCGTG

GCCCTGAAGCCCAGATATCGGCATCGAGGGTTAGCCAAGGAGATGCTAAAAGCCGCCTTCTTATTGGCTGCAAGTGCCAA

TATCCCAGCAGTCGCTGGCATATTTACCACCGGCAATATGCATCAGAAAGCTGAAGAGCTGGGCTTCCAGAAGTTCAACG

AGATATACTACGTTAGATATCTTATTAACAATGAGATTGTCTTCTGGGACACGGGCCTCGGTAACTATGGCGCTGCCCTA

ATGGGGTACAGGGTACCAGATGTAGAGGAACCTGCGGAATTGAAAAAGCAGCAGTCCTCGAGGTTCAACGTACAATTGGA

AGATATCGAGGGTGATGAAGACGAATAG

>Cobl_FAT20

ATGGGTGATAACGACGGTTATTACGATGGTGTCGGGACCATGGAGTCTATGCCAGGTTCACCTGAAGTCACCTACAACTG

GGTTGATGTAACTACAGATTTCTTCAAAAATATAGAAGACCTCCGACTTGGGGAGTTATTGCACGATGGCCACCTATTCG

GCTTGTTCGAAGCGATGTCGGCCATCGAAATGATGGACCCTAAGATGGATGCAGGGATGCTTTGTAACCGAGGCGTTGCT

AAGCCTCTTAACTTTACGCAGGCTGTTCAGGCCGGAAAACTGAAAATTGATGATCTTGAACCAAATGAACTGATTGGTAT

AATCGACGCTACCCTGGCTTGCATAGTGTCTTGGCTAGAAGGCCATTCCCTAGCCCAGACTGTCTTCACTAACCTGTACT

TGCATCAACCACATTCAATAGTGAACAAGACTTTGAAGGCATATTGTATAGCTGTTTATAAACTACTGGATTGTATAAGG

GACTGTATTAACAAGGCCCAAGTGTTTGAAGAAGAAGATTTCCAACCAATGGGTTATGGTTATCGTCTGGGCTCGAACCC

GCAGACTGGCAGCACTTTTGACCCCAACCTGGATGTTTCAGAGCAGAAATGCATTGCTCTGCTGCGAGAACAGGAGGAAG

AGCTGAATAAGAGGTCTCGGGGTACAGATGATGAGGACAACCTTTGGACTGCACTAGCAGCTAGAATAAGGTTCACAAGG

ATGTTCTACCAAGCTCTTCTCCTGATCACCAAGAAGGACTCACAGTCCGGTGCTGACTGTGTAGCTCTGCTCAATGGCTG

CTCTGAAATGATGAAAGTCATCATCAGGACATCTCCGAAGGGGACACAAGCTGTGGAGAATTCGGACTCTCCAAACCCCA

TGGGGTTCGAGCCGATGATCAACCAGCGGCTGCTACCGCCCACGTTCCCCCGCTACACGCGCATCAAGCCGCGGGCCGAG

GCGCTCGCCTACTGCGACGAGCTCGTGGCGCGGCTGCGGCGCGCCTGGAAGATCACGTCTTGCACCAACTTCCATACGGC

TTTGGACTTCTTCATGGAGTTCAGCCGCGCGCGCGCCTGCATCCTGTCCCGCTCGGCGCTCCAGCTGCTGTACCTCAGCC

CCTCCCCCGCGACCACCGCCTCCATGGCCCAGAGCGCCATGAGCGCACCGCCCACCGCCCCCCGCCCGCCCCACGCTTTC

AGGGAGATACTGCGCGAGTCGGTGCGGAGCTTTGTTAACCCGCCCGCGCTGACGCCGAAATCTCCAGTGTTAGCGACTCC

ACAG

>Cobl_FAT21

CTCGCTCGCTGCGTGCGTCCGTTCGCGGTGCTGCTGCAGGTGTGCGGGCACAACCGCGCGCGGCAGCGGGACAAGCTGGC

CCTGCTGCTGGACGAGTTTGCTGCCCTACAGGAAGAGGCAGAAAGTGTGGACGCGGTGGTGAGCGGAGCCGCGGGGACCG

CCCCGCGCGCCTGCTTCGGCACTTGGCTCCTGTACCACGTGCTACGCGTCATGATCGCGTATTTACTCTCCGGGCTCGAG

CTAGAACTGTACAGTGTGCACGAGTACCATTATATCTTTTGGTATCTATACGAGTTCCTCTACGGCTGGCTAGTATCAGC

CCTAGGGCGGGCGGAAGGCCTAGCGGGCGAGGGCGCGCGCAGGCCCGAGGCGAAAAAGAGCGGAGCGCGCAAACAGAAGA

AACGGACGCGGCCTTATGCGCGGGAGGCGCTCATGTGCCAAGTCATGCAGAATATGTGCGGGGGGTATTATAAGGCCCTA

GTAGCATTCAAACTCCAAGGCAAGATCCGTCAGCCAGCATCGGAGTTCGACAACGCTTGCGTCCGCTACAAGCACCGCTT

CGCGCCGCTGTCCGTGCTCACGCCGCCGCAGGTGCACTACCACGAGTTCGTGGAGATGACGCAGCCGCTGCAATATGAAA

ACCCCGTGATACTATACCTAGGCGGCTGCAAGCACTTCCAGCAAGCTCGATCACTTCTAGAAACTGTCACAACACCAGAC

CAAGAGGTAACGGATCTCCTAAAGGTAGCAAAAACAAATTTCGTAGTACTCAAATTATTAGCCGGTGGACACAAGCGCGA

CTCGACGACGCCGCCAGAATTCGATTTCACCGTACATAGACATTTTCCTATCATCAAACTTGTATAA

>Cobl_FAT22

GGCGCGGCGCTGGGCGCGGCGGCGTACCAGCGCGGCTATTGGTCGTTCAGCGGACGCACGCTCGTGGTCTTCGGCCTGTA

TGTGAGCCCCCCGGCGCGTCGCCAAGGGGTCGGAAGACGCCTGGTGCTTGAGATGTGTCGGTTGGGGCTAGCAGAGAACG

CGCAGGTGGACTGGCTGCAACCCCGAGGTCCTGACTCTGAGTCATTCATAGCACAGTTCGGGGCTGTCAGAAACGAGGTT

TTTGATGACCATCACATGATGAGGATGCCATTTAACTCCATTAACTTAGCTGCCAGCGGAGGCCATGGAAGCAGCGGTGT

GCACAATCAAGGGCCGGATGCAAATGAAATCGTCCTAATTCGCCGGGCGAGCCGAGATGACATGGCAGCCATGATCGACA

TGATAAATGAGCTGGCTACCTTTCAAGGGAAACCGGAGGGGCCTCAACTAACTGTTAAAGATTTAGTGGAGGACGGTTTG

ACGTGTTCCGTGCCGTGGTTCTTCGCGTATGTGGCGGAGCTCGGCGGTGCAGTGGTGGGCATGGTCATTGGGACTCGCCC

CTGGAACGGCTACAGGGTCCATTATCTCCAGAATCTGTACGTGCGGCCCTGCGCACGGCGGCGCGGCGTCGCCGACCAGC

TGATTAGGGAATTTTGCAAGTTTTCCCTGGAGGAAGGGGTGGAGCACGTGGACTTGCACGTGTCACACAACAACCCGCCG

GCTCAGCAGCTGTACCGACGCCTGCAGGCCTCCGAGGTGCCCGCGCGAGACATCCCGCCTGTCTACCGGCTCACCAGGAA

CCAGATCAAAGACATTGTCGCGCAATCTTTACTATAA

>Cobl_FAT24

ATGACTGCTGAGAAACTGGGGGCACAGTATAAGGTTACCAGGGATGAGGTGGATAATTTTGCGCTGCAGTCGCAGCAAAG

GTGGAAGACTTCCAATGACGCCGGCGTGTTCAAGGCCGAGATCGAACCCGTGACCATCACTGTCAAGCGCAAGGAGGTGT

CTGTCACTACCGACGAGCACCCACGCCCCCAGACGACACTTGAGGGATTGAAGAAACTAGCCCCCGTGTTCAAGAAAGAG

GGGTTGGTGACGGCTGGTTCCGCTTCTGGCATCAGCGACGGCGCCGGAGCCCTAGTGCTCGCCAGCGAAGAAGCCGCCAA

AAACCTGAAACCCCTGGCGCGCCTCGTGGGTTGGTNTGACCCCAGCATCATGGGGGTCGGCCCCGTGCCCGCCATACAGA

ACCTGCTCAAGGTTACCGGCTTCACGCTGAACGATATCGACCTGATTGAGATCAACGAAGCCTTCGTAGCACAAACCCTC

TCCTGCGCGAAAGCCCTAAACCTGGACATGTCCAAGCTGAACGTGAACGGCGGCGCCACCGCGCTC

>Cobl_FATP1

ATGGCAGAAAAGACTGAGGTTCGCTTCGAGGAGGCGGAGCGCAACAAGGCAGCCATCTTAGCTTCCATCGCCGCAGTCAG

CACAGTCCTGGGATGGCTCTCCCTCGGCTCAGGGGTCCTCGTGGCCGCTGAGGCCCTGGTAGCCATTTACCTGATGACCG

GGGACCGGTATCAATGGATCTACATATGGCACAAGACTGTTTACAGGGATTTGTTAGGTCTCCGGGTGCTCCTCATCACG

ATGTTCAAGATCTGGATGTGGGAGCGGCGCGGGACGACGGTGGTGAAGCGCTGGGAGGAGGTCGCACGCTGGGCGCCCGA

CAAGAAGGCTTTCATCATGGGCGAGCGCTCGCTCACCTTCCGGCAGGGCGACGAATTCAGCAACCGCATAGCCTGGTACT

TCAAACGGCAAGGGTTCAAGAGTGGCGAGGTCATCGCGCTCTTCATGGAGACGCAGCCGGAGTACGTCTTCATCTGGCTT

GGGTTGGCCAAGCTCAGGGTCACCACTGCCCTCGTCAACACCAACCTTAGAGGAGCTCCACTCACGCACTGCCTGAAGAT

CGCGGGATGCAAGGCGCTGATCTTTGGGGATGAGATGACGGATGCTGTCAAAGAAATCCAGAACGAGATTCGTGACATCC

CACTCTTCCAATTCAACTCGATTGAGCGTAGTGAAGCCGCCACTCTCCAGGACACGTCTCCCCTGGCTTCCGAGCTGACG

GAGATGAGCTCAGAACCGTTCATCGAAGCGGAGCCGGCGAACCCGCGAGACACGCTCATGTATATCTACACCAGTGGTAC

CACTGGGTTCCCTAAGGCTGCTGTTATCACGAACATCAGGTATTTCTTAATCCCACTCGGCGTGCACACATCAGCGTGTC

TTTCTTCGGAGGACATAATTTACGACCCCCTTCCCCTGCACCACACGGCGGGCGGTGTGCTGGGAGCAGGACAAGCCCTC

ATATTGGGCAGTACGGTTGTGTTGAGGAAGAAATTCTCTGCTTCTAACTACTGGAGCGACGTGGCGAAGCACGGTTGCAC

TGTAGCCCAATACATCGGTGAGATCTGCCGCTACCTCCTGGCTGTCCCGCCAGCGGCCACCGACCAGGCGCACCGAGTCC

GCGTGATCGTGGGCAATGGTCTAAGGCCGCAGATCTGGCAGGAGTTCGTCCAGAGATTTGGAATTGAACGGGTGCTAGAG

TTCTACGGGGCTACTGAGGGGAACAGCAATTTAATCAATTTGGATTCAAAAGTGGGTGCGATAGGATTCTTGAGCCGTTT

AGTATCTTCGGTGTACCCGCTTACTCTAGTCAAATGTGATGAAATATCAGGAGAAATATTGAGGAATAGTGAAGGAAGAT

GTGTCAACTGCGGGCCACACGAACCAGGACTCCTACTAGGCAAAATAGACGCCAAGAAGGCAATCCTAACGTTCTCCGGT

TACGCCGACAAGACAGCATCGGCGAAGAAGATGGTCAGAGACGTGAAGAAGGAGGGCGACTGCTACTTCAACACGGGGGA

CATCCTCGTCATGGACCACTTCGGGTACTTCTACTTTAAGGACCGGACTGGGGACACTTTCAGGTGGCGTGGTGAGAACG

TGTCAACCGCAGAGGTGGAAGGAGTCATCAGCACCCTCGTGGGATTAAAAGACGCCATTGTGTATGGAGTTGCGATTCCC

AACGTAGAAGGTAAAGCAGGCATGGCGGCCATAGCCGATCCAGAAAGGAAGCTGGACTTCTCCCTCCTCACGAAAAGCCT

CCGTTCGTCGCTGCCAGTATACGCTAGACCCCTCTTCATCAGGATACTGCCAGAGCCCCCCCTCACAGCCACCTTCAAAC

TCTGCAAGAAGGATTTAGTGGAACAAGCGTTCGACTTGGGAGCCCATGAGGATCCTATGTACTTCTTGGACCAGAAGACT

GGCGAATATGTTCCTTTGACGAAGAAGTTGTATGATGATATTGTTAAGGGTGTTGTGAGATTGTGA

>Cobl_FATP2

ATGTTGTTCGTACTGGGAGGTGTAGCCGCGGCTTGCGTCGCCGTTTGGCTGTATTACGGCTTTCTCTCCACTTTAGCACT

TTCTGCAGCCTTAGTGTTATTGTACGGTGTTTTGTTCCATTGGAGATGGATCTACGTCGCCCTGAGGACGGCCCCTCGGG

ATCTTCTAGCACTAACATGCTATGTAAAGCTGCTGTGGACCACATGGAGGTTTTCAAAGAAGAACTGGACTCTCCCGGAC

ATGTTCCATGATACCGTGAGTCGCTACCCGGACAAACCTTGTTTCCTGCACCAGGACGAAGTGTGGACATTCAAGGAGGT

CGAAGACTTCAGTTTACGGGTGACAGCCGTCCTCAAGGCCCAAGGCGTCAAGAAAGGCAGTATTGTGGGCATGTTAGTAA

ACAATTGCCCCCAAATGCCCGCCATTTGGATGGGCGCGGGGCGGATCGGGGCGGTCTGCCCCCTCATCAACACTAATCAA

CGCGGGAACGCGCTGGTACACTCAGTCAATGTCGCTCAGTGTGACGTCGTCATATTCTCCGAGGAATATCAATCAGTGAT

TCAAGAAGTGGCAAGTCAACTAAGCCCGTCAATTAAATTTATGAAGTTTATTCACCGTCGCCTGAACACGGCGAATGCAG

AGGTCAAAGCCAGCGGTGACCACTTTGCTGATTTGACCTCTTTGCTGGAGAACACCCCGCCGGCGCCTTGGACCTTAGCT

GATGGGGAAGGCTTCCATGGAAAACTACTTTATATCTACACATCGGGGACCACTGGGTTGCCTAAGGCCGCTGTTATCTC

TAGTTCAAGAATCGTGTTCATGGCAGCAGGCCTTCACTACCTGGCGGGTCTAAGTCCAGATGACATCGTGTACTGCCCAC

TGCCACTCTACCACACTGCAGGAGGCGTGATAAGCGTCGGCCAAGCACTCATTTTCGGGTGTACAGTCGCTGTAAAAACC

AAGTTCTCCGCGTCTCAATATTTCCCTGACTGTATCAAGTATAAAGCCACAGTAGCCCACTACATCGGCGAAATGTGTCG

CTACGTTCTCGCCACGCCGCCGGCTCCCGCCGACAGACAACACTCGGTGCGAACAATATTCGGCAACGGCATGAGACCAC

AGATCTGGGATGAGTTTGTGACCAGATTCAACATTAAGCGGGTCGCCGAATTTTATGGAGCCACTGAAGGAAACGCAAAT

ATTGTGAACATCGACAGTAAAGCCGGAGCAATAGGTTTCGTGTCCCGGATATTCCCGCGAGTGTACCCGATCGCCATCAT

TAAAGTGAACCAAGAGACGGGCGAGCCCATCCGAGATGCGAACGGACTTTGTCAGTTGGCTCAAGCAGAAGAGCCGGGCG

TGTTCATCGGCAAGATCTCGGCGGGCAACCCGGCGCGCGCGTTCCTCGGCTACGTCGATAAATCCGCTTCCGACAAGAAG

ATCGTACGAGACGTGTTTGCTAAAGGGGACTCTGCTTTTATTTCTGGCGACATCTTAATATCAGACGAGCTAGGCTACCT

ATACTTCCGCGATCGCACGGGCGATACGTTCCGCTGGCGCGGAGAGAATGTCAGCACCACAGAGGTCGAGGCCGCCGTCT

CCAGAGTCGCCGACAACAGAGACGCTGTCGTTTATGGAGTCGAGGTACCGAACACGGAAGGCCGCGCGGGCATGTGCGGC

ATTGTGGATGCAGATGGCACGCTCGACCTGACGAAGCTGGCTCGCGACCTGGCAAAGGACCTACCAGCCTACGCCAGACC

TACCTTCTTACGGGTCATGGAATCCGTCGATATGACAGGTACTTTTAAAATGAAGAAGACAGACTTACAAAAGAATGGTT

TCGATCCGAATGCAGTAAAGAAAGACAAGCTCTACTATCTCGATTCCAAACTGGGCCAGTATCTTCCACTCGGCCCCGAG

GAGTACGAAAAAATTATCAACGGCCGAATAAGACTATGA

>Cobl_FATP3

ATGTCGAGTACAGAGGCGATTGTCGATGACAACATGAACAAGACTGATGCAAGGCCGCAGAATGAGGTGAAACAAAATGG

TAAAGATTTAGAAAGTGGCAAGACATCAGATGAACCTAGGCAATGGGGTCGGATGATAGCTATGATTCTCGCTATTGGAG

TCTTGGTGGCAGCCTGCACTGTCACGTGGGTGTTCCAGGATTGGCAGACCAGTCTCCAAGTTTTCGCTATTCTCGTAGTG

GTTTACGTCATTGCGTTCCATTGGAGATGGATTTATATCGCCATCAGAACAGCACCAAGAGATTTCTCAGCGTTATACTG

CTACATCAAGATTTTGCTGCTCACAAGAAAACTGACCAAGAAAGATTTTGTGATGCCAGACATGTTCCACGACTTCGTGA

AAAAGCACCCCAACAAAGCCTGCTTCTTATTTGAGGAAGAAACGTGGACATTCCAACAGGTCGAGGAGTACAGCTTGCGC

GTGTCAGCTGTTCTCAGAGCCCAGGGTGTCAAGCCTCAGAGTAAGGTGGCTGTGATGATGATCAACTGCCCCGAGATGCC

GGCGACGTGGATCGGCGCCTGCCGACTGGGTGCGATTGCACCGCTCATCAACACCAACCAGACCGGTGCTGCGCTGATAC

ACTCCATCAATATTGCTGACTGCGATGTTGTTATTTACGGGAGCCAGTTTGAATCTGCGATACAAGACATTGCCAAAGAT

CTCAACCCATCCATCAAACTCTTCAAGCACATTCGCCGTCCTCTGAACACGTCTGGTGATGGCGTGAAGGTGGCAGAATC

TACCAACGACTTTACGGCAATGCTGGAGTCCACTCCGCCGTCACCCTGGTTCCTGACTGAAGGGAATGGGTTTAACGGCA

AGATGCTGTACATCTACACCTCTGGAACCACTGGGTTGCCCAAAGCTGCTGTTATTTCCTCGTCCAGAATGGTGTTCATG

GCTTCAGGCGTCCACTATCTCGGCGGTTTGAGAAAAAAAGACATAATTTACTGCCCTATGCCGCTGTACCATTCGGCTGG

CGGCTGCATCACGATGGGACAAGCCTTCATCTTCGGCTGCACGATCGTATTGAAGACCAAGTTCTCGGCATCTGCGTACT

TCAAGGATTGTGCCAAATATAATGCTACTGCTGCTCATTATATTGGTGAGATGTGCCGTTATATTCTTGCGACTCCTCCT

TCAGCGGCTGACAAGCAACACAAAGTGCGCACGGTATATGGAAACGGAATGAGACCAACTATATGGACCGAGTTTGTAAC

CCGGTTTAACATAAAAAAGGTTGTGGAATTTTACGGCGCCACGGAAGGAAATGCTAACATAGTGAACATAAACAACAAAA

CTGGCGCAATTGGATTTGTATCGAGAATCATACCGCAAGTGTATCCTATTGCTATTCTTAAATGTGACGAAGAGACGGGT

GAACCTATTCGAACTTCACGAGGATTATGCCAGGTGGCCAAGCCTAATGAGCCAGGAGTGTTTATCGGCAAAATCAAACC

GAACAATCCTTCAAGAGCCTTCCTCGGCTATGCAGACAAAGCAGCGTCCGAGAAGAAAGTCGTGAAAGACGTTTTCACTC

GTGGAGACTCTGCTTTCATATCAGGTGACATCCTGATAGCAGACGAGCTGGGCTACCTCTATTTCCGCGACCGCACGGGT

GACACGTTCCGGTGGCGCGGAGAGAACGTCAGCACCACAGAGGTCGAGGCCTCCGTCTCCAGAGTCGCTGACAACCGGGA

CGCAGTCGTTTATGGAGTTGAGGTCCCCAACATCGAAGGTCGCGCGGGCATGTGCGGCATCGTCGACACAGACGGCTCGT

TAGACCTGGAGCAGCTGGCGCAGCGCCTGGCGAGAGACCTGCCGGCGTATGCGCGGCCCGTTTTCATGAGGGTCATGACT

AGCTTGGATATGACGGGTACATTCAAAATGAGGAAGGTTGACTTACAAAAGGAAGGATTCAATCCAACAATTATAAAAGA

TAAATTATTCTACTTAGATTTAAAACAGGGGAAGTACCTTTCTCTTGGTCCAGAAGAGTACCAGAAGATTGCGTCTGGGC

AAATAAGACTGTGA

>Cobl_FATP4

ATGGACGCAGTGCTGGCAGCTCTCGCCGCGCTCATGGCACTGGCCGCCGCGATGGCCGCCGTGCTAAGCACGCTCTCCAA

AGCCGCGATTTTCGCTCTCCTCGCGATCGCGCCCTGCGTATACCGCTACAGGAAGCGCATTTACGTAATCGGCAAGACGT

TACCCCGGGATCTCAAGTTCCTATGGCGCTACGGCAACAGCATGGTCCGTCAGATGGCGTGGGGCCGGCAGAACGCAACG

GTGGCCGCTCTGTTCACGGCTCGCGCCGAGCGCACGCCCGACGCTGCCTGCTTCATCGTCGTCGGCGACCGCACCTGGAC

CTACAAAGAGATAGCGTCCAAATCCAACCAAGTGTCGCGTGTGATGCAAGAGCACGTCGGCCTGAAGCGCGGCGACGTGG

TCTGCGTCTTCATGCCCAACTGCGCAGAATACGTCTGTACCTGGATGGGCATGGCCAAAGTCGGCGCTGTCTCGGCCCTG

ATCAACAGCAACCTTCGCCACAAGCCGCTGCTGCACTGCATCCAGGTTGCGAAGGCCAAGGCCGTCGTGTTTTCGGATCA

GCTGGCTGAAGCCATCGCCGAGATCCGCGACCAGCTGCCTTCAGACATGAAGCTGTTCCAGCTCTACGGTCAGTGCGCGC

CAGGAGTGCTGGACTTTAGTGCGGAGATGGCGCGCCACCCGCCCGACTATCCGCTGGTGACGGAACAGTTGAATTACAAG

GATACGCTCCTGTACATCTACACTTCGGGAACCACTGGGATGCCTAAGGCTGCTGTGCTGCCTAATTCCAAGTACCTCCT

AATCGTGCTAGCAACGGTCCACATGCTGGGTCTCCGCGCCTCGGACCGCATGTACAACTCTCTGCCGATGTACCACACGG

CGGGCGGCGTGGTCGGCACGGGCGCGGCGCTTGTGGATGGCATCCCTTCTGTCATCCGGCCGCGGTTCTCCGCCTCTAAC

TTTTGGACGGACTGCATTAAGTATCAGTGCACGGTGGCGCAGTACATCGGCGAGATGTGCCGCTACTTGCTGGCGCAGCC

GGCGCGCGCGACGGACGCSCAGCACCGCGTGCGCATCATGGTCGGCAACGGCATGCGCCCCGCCATCTGGCAGAGCATCG

TCGACAGGTTCAAAGTACCGCAGATAAATGAAATATACGGCGCGACCGAGGGCAACGCCAATATAATAAACGTGGACAAC

ACGGTGGGCGCGGTGGGTTTCCTGCCGAAGCTGCTGCCGGCGCGCGCGCTGCCCATCGCCGTCGTGCGCGCCNNNNNNNN

NNNNNNNNNNNNNNNNNNNNNNGACGGATACTGCGTGCGCTGCGCACCCGGCGAGCCGGGCATGTTCATCGGCCTGATTT

CTCAGGGGAACGCTTCGAGGGAGTACTACGGTTATGTAGACAAGAGCGACAGCAACAAGAAGCTCGAGCGCGACATATTC

TGCAAGGGCGACGCAGCCTTCGTGAGCGGCGACATTCTCGTCGGCGACGAACTCGGGTACCTCTACTTCCGCGACCGCAC

CGGCGACACCTACCGCTGGAAGGGGGAGAACGTGTCCACTGGGGAAGTTGAGAACGCGATTAGCCCCGCTGTGGGACAGA

AGGAGGCTGTCGTGTATGGAGTGTCGATCCCAATGAACGAGGGCCGCGCAGGCATGGCAGCGGTAGCGGACCCTGCGCGT

GCGCTCGACCTGGCGCAGGTGGCGCGCGATCTCGACGACTCGNNNNNNNNNNNNNNNNNNNNNNNNNNNNNNNNNNNNNN

NNNNNNNNNNNNNNNNNNNNNNACATTCAAGCTCAAGAAACTGCAGTACCAGAAGGAGGGTTTCGACCCCGACGTGATCA

CCGATCCGCTGTACTTCCGCGCCGGCGCGCAGTTCGTGCCCATCACCTCCGCGCTGTTCAAAGACATCTGCAACGGACAA

GTCAAGCTC

>Cobl_GOBP1a

ATGAAGGACGTCACGCTGGGTTTCGGAGAGGCGGGCTGGCTGGGTCGGGCGCTGCTGCTGGTGGTGATGCTGGGCGCCTC

GCAGAGCACCGTCGAAGTCATGAAGGACGTCACGCTGGGTTTCGGAGAGGCGCTGAATAAGTGCAGGGAGAGCAGCCAGY

TGTCGGAGGAGAAGATGGAGGAGTTCTTCCACTTCTGGCGCGACGACTTCAAGTTCGAGCACCGCGAGCTGGGCTGCGCC

ATCCAGTGCATGTCGCGCCACTTCAACCTGCTCACCGACAGCGAGCGCATGCACCACGAGAACACCGACAAGTTCATCAA

GTCCTTCCCYAACGGTGAGGTGCTCTCGCAGCAGATGGTGTCGCTGATCCACGGCTGCGAGCAGCAGCACGACGCGGAGC

CGGACCACTGCTGGCGCATCCTGCGCGTGGCCGAGTGCTTCAAGACCGGCTGCACGGAGCGCGGCATCGCGCCCACCATG

GAGATCCTCATGGCCGAGTTCATCATGGAGTCTGAGTCT

>Cobl_GOBP1b

CTGGGTCGGGCGCTGGTGCTGGCGACGCTGCTGGGAGTGGCGCGGAGCACTGTCGAGATCATGAAGGACGTCACGCTGGG

TTTCGGAGAGGCGCTCAAGCAGTGCAGGGAGAGCAGCCAGTTGACGGAGGAGAAGATGGACGAGTTCTTCCACTTCTGGC

GCGAGGACTTCAAGTTCGAGCACCGCGAGCTGGGCTGCGCCATCCAGTGCATGTCGCGCCACTTCAACCTGCTGACCGAC

AGCGAGCGCATGCACCACGACAACACGGACAAGTTCATCAAGTCCTTCCCCAACGGCGAGGTGCTGTCGCAGCAGATGGT

GGCGCTGATCCACGGCTGCGAGCAGCAGCACGACGCGGAGGAGGACCACTGCTGGCGCATCCTGCGCGTGGCCGAGTGCT

TCAAGGTCGGCTGCCAGGCGCGCGGCATCGCGCCCACCATGGAGATCCTCATGGCCGAGTTCATCATGGA

>Cobl_GOBP2a

GCTGGGGGGCAGATGGTGGCGGGAACTGCTGAGGTGATGAGCCATGTTACTGCTCACTTTGGGAAGGCTTTGGAGCAGTG

TCGGGAGGAGTCAGGCCTGTCTCCAGCGATCCTCGAGGAGTTCCAGCACTTCTGGCGCGACGACTTCGAGGTGGTGCACC

GCGAGCTCGGCTGCGCCATCCTCTGCATGAGCAACAAGTTCTCGCTCATGCAGGACGACGCGCGCATGCATCATGAGAAC

ATGCATGACTATGTCAAGAGCTTCCCTCAAGGTGAGGTCCTCTCAGCAAAGATGGTGGACCTGATCCACAACTGCGAGAA

GCACTACGACGACATCAAAGACGAYTGYGAYCGCGTGGTCAAGGTGGCGGCTTGCTTCAAAGTGGACGCCAAGAAGGCTG

GCATCGCTCCTGAAGTAGCCATGATTGAGGCTGTTATGGAGAAATAT

>Cobl_GOBP2b

TGTGCTGCGGTGCTGGTCGCTGGGGGGCAGACGGTGGCAGGGTCTGCTGAGGTCATGAGCCATGTCACTGCCCATTTCGG

AAAAGCTTTAGATGAGTGTCGAGAGGAGTCAGGCCTGTCTGCAGAGGTCCTAGAGGAGTTCCAACACTTCTGGCGCGACG

ACTTCGAGGTGGTGCATCGTGAGCTCGGCTGCGCCATTCTCTGCATGAGCAACAAGTTCTCCTTGATGCAGGACGATGCT

CGCATGCATCATGAGAACATGCACGACTACGTCAAGAGCTTCCCTCAAGGTGAGATCCTCTCAGCAAAGATGGTGGAGCT

GATCCATAACTGCGAGAAACCTTATGATGACATCAAGGACGACTGCGCCCGCGTTGTCAAGGTGGCGGCTTGCTTCAAAG

TGGACGCCAAGAAAGCTGGCATCGCACCTGAAGTGGCCATGATTGAGGCTGTGATGGAGAAATAT

>Cobl_IR1

GTGCGCCAGCAGGCCACAGCCTGGCGGCGGCTGCTGCGCCAGCGGGACGCGGCCGTGGGCGTGGTGCTGGACGGGGCGTG

TCGCAACACACAGGATGTTTTGATTGACGCCTCGGAATCCAGGTTGTTCGACGCTGCGCATGCGTGGCTGGTGCTTAACG

ATGATAAAGATGGCGCCGGCGTCGGCGCACGATTCGCCGATCTGAAACTTAGTGTTGATGCCGATGTTGTGGTTGCTACT

TACTGCGAGGATGACGGTGTGTACCACTTCAGTGATGTGTTCAACTTCGGACGCATCCAGGGCAATCCGTTGGAGGTTAA

ACAACTAGGAACCTGGACTATGGAGACCGGTTTGAACGTTTCGTCCCCAAGATTCAAGTACTATGATCGCTGGGATTTCC

ACAATCTTACTCTAAGGGCAGTATCCGTGGTACGAGACATTTCTCAAAAGTTCGACGAGAAGATGCTCTCTGCGCCAGCG

TACACGGACGGCGTGGCCGCCATGACCAAGATCAGCTCGCAACTCCTCAACTTGCTCAAGGAAATGCACAACTTCCGTTT

CAACTACACCATAGTGGGGCGCTGGATAGGCTTGCCGGAACGCAATACTACTCCGACAGTCTCCAACACGCTGTTCTGGC

GCGAGCAGGACATCTCCTCGACGTCAGCGCGGCTGTTCCCGGCCTGGCTGGAGTGGGTGGACACCTTCTTCCCCCCCGTC

ACGCACCTGGAAACGAAATTCTACTACATAATACCAGACAAAGGCGTCGGCGACTATGATAACCAGTTCCTCACCCCCAT

GTCGCCAGGAGTGTGGTGGTGCTCGGCTGCAGCCGGCGTGGTGTGCGCGTTGGTGCTTATTGCTGCCGCCGCCACGGAGG

GAAGACCAGAGCCGACGTCCTATGGGATATTCAGCGTTCTAGCTGCTGGGTTTCAGCAGGATTATGAGGATGGAGCGCAA

TCGCTAGAAGATAGTTCAACTAACAGCCGTAAACTAGCGCTGCTAGTAGTGGGTCTGACAAGCATGCTTCTATACAACTA

CTACACGAGCAGCGTGGTATCCTGGCTCCTGAACGCGGCAGCACCATCCTTGGACTCGCTGGACGCTCTTATCAAGAGTG

ACTTCGAGCTCATCTTCGAGGATATAGGGTACACGCAGGGCTGGATGGCCAATCCTGGATTTTACTACTACAGCGGCTTC

ACAAACGAAAAGGAGGACGAATTGCGACTGAAGAAAGTGACCAACGCCAAGCGAACAGTGCCGCTGATGCAGACTGTCGA

GGCTGGGACCGAGTTGGTGCGGACTGGCAGTTATGCGTACCACACAGAACCGTACACAGCGAGCCAGATCATATCCCGCA

CGTACGTGGACAAGGAGCTGTGCACGCTGGGAGGCCTGCAGATGATGATCCCGGCGCACGTGTACGTCATGGGGCAGAAG

AGGAGCCCTTACAAGCAGTTCTTCGTCTGGAGTATGATGCGCCTGCTAGAGCGCGGCCACATGAAGGCGACGCGTGCGCG

CGTGGGGGGCGTAGTCCCCCCTTGCTCGGGCACAAACCCTCGAGCACTGTCGCTCGGGCAGGCGGCGCCGGCTTTCCTGC

TGCTGGCTCAAGCCATGATGCTCGCTCTGTTCATGTTGACCGTTGAGCTGTGGTGGCACAGGTACAAACAGCGCACAGCG

ATCGGCTACAGTAGGGGACAACCGCCACCAGTACAGGTGGTGGAGAGATCACGTTGA

>Cobl_IR3

ATGCTTTCAAACACTTTGTTGCTTTTCTTAGTTGCTACAGTTTCGGGTTTGCAGCAAAATGTTAAGGAATTTCCTTTAGA

TTTCTTTAAAACAAGAGATGTGAAGTTTATCTGTTTGTTGACTTGCGGGGATAAAAGCTGGAATCAGCAGTTTGCTGTTA

ATGCCTCAAAATCGTCAATTGCTGTTTCAAGTGTTACTATTGACAATTCATCATCAGATTATGCTGATGCATTAAGAGTT

TGTTTGACGCATAAATACACAGCCATTGGCGTTTTCATAGATGAACAATGTCCACTTTTTGAAGACGTGCTGCTATTTGC

TTCTGAAAGCGGTTTCTTTGACGCAAATCACAAATGGTTGATAGTTGACAATGATGCCTGGATGTCCAATGTCACAAGTG

GTGCTGAAGATTTTGAGCGAAACATTAATGTCACCAGAGACTCTCAATACTCTGAGAGTAGCGTGAATTTGACCAGGCTA

CATAATATATATGATAAACTAAATCTAAGTGTAGATGCTGATGTGACGCTTTCTTTGCAAGAAGGTCAAGAAAATAATAT

TTACGAAGTCTACAGCTATGGTACAATTCGCGGTGGTAATACAATCGTCAATAAACTTGGATCTTGGGAAAACAAATCAG

TTTTAGTGCCGCAACTGAACGGATACAAATACTATCGACGGTGGGACTTCAATCAGTCATCTTTAAACGTAGTTGCTGTT

ATGTCGACTCCCCCCGAAATATTTGACGTAGACATGATAATTGGAAACCAGCCCAAGGTGGGTGTTGCCATAATAACTAA

AACGAGCCTGGAGGTTCTGGAGGAACTGAGAGAGCTGCACAATATCAGATATAACTATACAATTGTGGACCGGTGGATAG

GTGACTTTAATAAGAACAGTTCAAGGGTTGCTGCTAATTCTCTGTACTTCAAGGAACAGGACATCACACCAGTACTACGT

GTCACGCGTGAAATTTTCCAGAAAGTGGACATGCTGCTTCCACCACTGACAGCTATTGAAACAAGGTATTACTACCGCAT

CCCAACGACTGGCCCTGGAAAGTTCGAGAACCAATTCCTACGTCCATTGACCCCTGGTGCATGGGGATGCGTGACAGCAG

TCACTCTTCTATGCAGCTTCATGCTATTCCTGGCAGCTAAGGTGGAACGACGTCCATCGGCGGTTCAGTATGCAGTGTTC

TCTGTCATGGCCACATTTTGTCAGCAATTTTATGACGATAATATTGGCAACGAAGATCCAACACAGCAATCCTCAGCTCG

GCAGCTTGCCGTGTTGGTGACAGGAGCATCCTGCGTACTGATCTTCAACTACTACACCAGTAGCGTGGTCAGCTGGCTCC

TGAACGGGCCACCACCTTCTATCAACTCCTTGGAAGAACTCTTGGAGAGTCCCCTTTCGCTTATATTCCAGGATATAGGA

TATAGTCGTTCTTGGCTGCAGAACCCAGCATATTACTTCAACAAAAAAAACGCCGAAGTTGAAGACAAATTACGCAGATA

TAAAGTATTCAATAAAAAAGTGGGGGCTCCCTTACTGGTGCCTCTGGAGGAAGGCATCGAGATGGTGAAAGCTGGAGGAT

ATGCCTACCACACTGAAGTGTACAACGCCAATACTCTGATCGCTAAGACGTTCACGCAGAGTGATCTGTGCGAGCTCGGC

TCTCTCCAGTCGATGGAGAAGTCGCAACTCTACGCTTCAGTTCCAAAAGATAGTCCTTACAAGGAGTTCTTCAATTGGAA

CCTCTTCCGCCTCCACGAGACTGGCGTCATCTCTCGCATCCAGCGCCGGACCAGCAGCCCTGAGATCGCCTGCGAGGGCA

GTTCCCCGCGAGCCCTCGCTCTGGGAGGAGCAGCGCCTGCTTTCATGTTGCTCGCCTTTGGATTCTTTCTGTCCACTGTT

ATACTTCTGATGGAGAGATTTATCAGCAGAGACCTTTCGAGACTGTCAAGAGGAATACATTTTAAAGCACGAGAATGTTA

A

>Cobl_IR4

CGCCGCCTCGATCTACAGCGCTACCCTATGAGGATCGCTACTCCGGTCGGACACTATGATGAAGCTGAGTACGGTGATAG

CTTCGCACAGTACGTTCTTGACAACTCCATGCTAGAACGCGACTCCGCCATCAGATGTGGCTACGGCGCCTCCGCGCTCA

TCTTGGAGGCACTCAACGCTAGAGAGGTCTTAGTGCAAGTTCAGTTCTGGACCCCAGAAGTGGGCAACAGTTCGAGTATG

ATGACGGAGGTTGCAGGCGGTACGTCACAGCTGAGCGGCGCGACTCTGCGCGTGAGAACGGACCGAATCGAGCGTCTTGA

CTATGTCTTGCCTATATGGCCATTTGTGGTGGGCTTCACATACTTGTCGGAGCGAGAGAGCAGCAGCAACATGTTCGTGA

TGCCGTTCTCGGGGCGCGTGTGGTGCGCGTGCGCGGCGCTCGCGCTCGTGCTTGCGCTGGCGCAGCGCGCGACGGCCAAG

GCGCCCGCGGAGAAGGACGGCGCGTGCCTCGCCGTCATCGCCACCTGGCTGCAACAAGACGCTAGTGCCGTGCCGGAGGG

TGCGTCGGGGCGGCTGACGTTCATGATGCTCTCGCTGAGCTCGATGCTGGTGTACGCGTACTACACGTCTGCGGTGGTGT

CGGCGCTGATGGCGGCCGGCGGCGGCGGGCCCGCCTCGCTGCGCGCGCTCGCTGACTCCCGGTACGGCCTCGCTTCGGAG

GACTACGACTGGATCCGATACGCCATGTTCGACGTAAAAACAACCTGGGAGGACCTCGAATACCTCAAGCGCAAGAAGAT

GACGTCAAACTTCTACCAGAGCACACAGCGCGGCATGCAGCTCGTTCTAGAAGGAAACACGGCCTTCCACGCCGAATACA

ACCACATATACCCGTACATGAACATATTCAACGACGAGCAGATTTGCAAACTACAATATGTGGACACAGTGCCCGAAATT

ATGTCATGGGTGGTGACAACGAAACACAGTCAATGGAAGGACGTACTCCGCTCCGCTGGCGGCTGGGTTTACGAGACAGG

GCTGGCAAAACGCCTCCTGTCCCGCTGGCAGGTGAAGCCCCCGCCCTGTCGAGCTGCCCTGCTCGCCGAGCGCGTCAAAT

ACGGCGACGTGGCACCCCTGCTCACTTTGTCCACTGTCGGCTTTATAACGTCCGTGGTACTACTGTTCGTAGAGAGGACT

GTGGCGAGATGGACGGCTATGAGGAGAGTAAAGTCAACTGGCGATGATGAGGCTGGAGTATCGGGAGAGGAGAATAATTG

A

>Cobl_IR7

ATGATTTTAATAGTTATTTTTCTTGTGCTCGGAGTAAAAGCGCGAATCAACCCACATGGACCCACAGTAGTGAATGATTT

CGCGAATTGTGTCAACAAACTCATAGACATAAATTTTGCTCAACCGGGACTTCTTTACTTCATCAACACGAATGAAGTTA

GCACATCAGCCGCCGCGATAAGAACAGCAATACTAAAATCAGTGCACGATAAACTTAAATATTCTGTAAAAATTGCTAGA

CCAACTAAACAAGATAAAGCTATCTGCAAGAATGACCAAGAAGTATTAGCGACGTCAGGTTTGCACCTAGACCACTTCGA

AGCAGTACCACTTGCTGACTACTTCGTTGTCATTATAGACAACTACGATGATTTCACCCATGTTGCGAGTAGACTGGCAA

GAGCAAGAAGTTGGAACCCAAAAGCGCTGTTCATTATTGTATTTTTTGGAATAACTAACACAGACGGTCAGAACATCGAC

CACGCAGAAAGCATGTTACTCTGTCTTTTTAAACTGAATGCCATCAACACTGTGGTCATTATTCCACAAGCAAGTAATAT

TAGACGAGCAACCGTTTATGGTTGGAAACCTTACGACCCCCCTCAATATTGCGGTTATTCCAATGAATCAGCTAGAAAAA

GGTTGTTTATAGAAAACGAGTGTGACCGCGGCATTGCCAAGTTTACGAAGAGTATTTTTGAAGACAAAATTCCATCGGAT

ATGAAAGGATGCACTTTGAAAATGCTTGCACTGGAACGACAACCGTTTATTAGTCGAGAACCTAGTGATGCCAACATTGA

GCAGTTGTTGATCAACGATGTTGCAAAACGATATAATATAGTATTGAGTTACGAATTCCTTAATGTATTTCGTGGTGAAA

AGCAGACAGCAGGACTCTGGGATGGAGCTCTTAATTACCTTGTRTCTAGAAAAGGACAGATGCTTTTAGGAGGTATTTTT

CCTGACAATGAAGTTCATGAAGACTTTGAATGCAGCTCTACTTATTTAGCCGACTCTTACACCTGGGTTGTGCCACGAGC

TTTTCATCAACCATTATGGTTGGCGTTGTTTATCATATTTCAGAACACAGTGTGGCTCTCAGTTGTAGCGGGGTTTATTG

TACTTGCATTAAGTTGGAAGATTTTCGCTAAATTGAGTGGAGACCCTACCTATAGGACTAATCTAGAACATTACTTTATC

AACACTTGGATCAGTAATTTGGGATTTGTCGCGTTTTGTCGGCCTGTAACTCATAGTTTGCGTTTGTTTTTTATTTTCCT

TAATTTATACTGTGTTTTGCTATTAACGGCATATCAAACTAAACTAATCGATGTCCTGACAAATCCGAGCTTTGAACATC

AGATATCGACTATTGAAGAATTAGCAGCGAGCGATTTAGGATGCGGAGGTTCAGAAGAATTACGTGATCTTTTTGAAAAC

TCGACTGATCCTATGGATATATATTTTTCTAGTAAGTGGGAGAACATTATTGATATAAAGGAAGCAATTATTCACGTGGC

ACTTTACAGGAATTTCTCTCTGCTATGTAGCCGTCTGGAGTTGGCACACATTTCAGCAGTAGTACCGGAGCTCAGTGACG

AGTTTGGGAACTCCAAAGTCTATGCATTTGAAGGTAACGTTTTCACAGTACCTTTGGAGATGGTATCTTTAAAAGGATTA

CCATTTATGAAGAAGTTTTCGAAGACTTTAGAGTCATTTAGACAGTACGGTGTAAACGACAAGGTGCGTCGATACTTTGC

AGGCTACACGTTAATGAAGAAAGCAACGCTACTAGATGATCTAGAAATGGAAAACACTGGTAGAGAAGCCTTATCCACTG

AATCTTTACAGGGTGGATTTTTGGCGCTTGTCTTTGGTTATATGTTCGGTAGCATAGTTCTTGTAATAGAAATAATGATG

AATACCAAATTTGTTAAGAGTATAAAAATATTAAAGAAGTCCGTTTATACGTTGTTTTAA

>Cobl_IR8

ATGGATTTCTATTGTATTTTTTTGTTTATTTTCGTTATAAACTTGGGCTGCGTGGTTTCAGAACTGAGTTTACGATTTGT

GTTTATCGTAGAAGCTCACGAGCAGGACTTGCCGCAGCTGGTCGGCCGAGCGCTGAAGATAGCCGAGGACTCGCAGCCGG

AGACCAGGCTTAGTGACTCCATCGTTCTGCTGGACCGGGAGAACGAGGAGGACAGCTATAGGCAGCTATGCTCATCGGTA

TCCAGCGGCGTGTCCACAATCATCGACTTATCCTGGTCCCCCTGGGACTCGGCAGACCAGCTGGCCGCTGATTCGGGAGT

GCCTCTAGTGAGGACGCTGCTAGGGTCACAGCAGCTGCTTAGGGCTTTGGACGAGTATCTGGAGTCGAGGAATGCGACTG

ATGCAGCGCTGTTGCTGGAGAGTGAGGGAGACGTGGACCGCACCTTGTACGAGTTGCTGGGCGAGTCCAACATCCGCGTG

TGGGTGCACGCCGGCCTGACGCGGGACTCGGCGCGCGCGCTGAAGGCCATGCGACCTGAACCCAGCTTCACAGCTATCAT

TGGAGGGAGCGCGTTCGTTGCTGATACGTATAAGAGGGCCGTAAAGGAGAAGCTGGTCCGTCGCGATTACCGCTGGAACC

TGGTACTGACAGACTACAGCACCCTGGACGTGTCTCCGGCAGTACCCGCTATGACGCTGCACGTGGACCCTGCTGAGTGC

TGCAGGATCCTCGGACGAAAGGACGGGTGCAGCTGCGGGCAGGATTTCGAGAGGAAGCTGCCGATCCTCTCGGCCCTCCT

CCAGCTCCTGGCAGAAACCTACTCCAAGCTGGAAGACGAAAGCTCCTTGACTGCCAGCGTGGACTGCGATAACCTGGTGC

CAGAGCTGAACGATACCAGGGCGCGGCTGTACCGCCAACTGGCTGAGGATTCGGGAGCTAGCAATGAGAGCGTGTTCTAC

TGGGATGGGAACAGGTACGGTCTCTTCCTCCGCTCCCGCTTCATACTCTCGACCTCCAAGCCTGACGCCGGCCTGCAGAC

TGTCGCCTCGTGGACCGCTGATGAAGAGTACAAGCTATTACCAGGAATGACACTGGAGCCGCTGCGACAGTTCTTCAGAA

TTGGGACTGCTGCTTCAGTACCATGGACGATGGCAAAGCTGGATCCGACCACGGGGGAACCGATGTTCAATGAAGAAGGG

CAACCTTTGTACGACGGTTACTGCATCGACCTGATACAGAAGCTTTCTGAGGCAATGGATTTTGACTACGAGATTGTGAC

CCCAAAAGCGGGTACTTTTGGACGTAAGCTCCCAAACGGGACTTGGGATGGCGTGATCGGGGACCTTATGAGAGCGGAAA

CTGATATGGCTGTCTCATCTTTGACTATGACGGCGGAAAGAGAGGAGGTTATTGATTTCGTGGCGCCGTACTTTGAGCAG

AGCGGAATACTTATCGTGATCCGCAAGCCGACCCGAAAGACGTCCCTCTTCAAGTTCATGACAGTCCTTCGTACGGAGGT

GTGGCTGAGCATCGTGGCGGCGCTGGTGCTGACCGGCTTCATGATCTGGCTGCTGGACAAATACTCGCCGTACTCCGCCG

CCAACAACCCCGGCGCGTACCCTTACCCCTGCCGGGAGTTCACCCTGAAAGAGAGTTTCTGGTTCGCGCTGACGTCCTTC

ACTCCACAAGGAGGCGGAGAGGCCCCGAAGGCGCTCTCAGGCCGGACCCTGGTGGCAGCCTACTGGCTGTTCGTGGTGCT

AATGCTTGCGACCTTTACTGCCAATCTGGCTGCGTTTCTGACTGTGGAGAGGATGCAGACGCCGGTGTCTTCGCTGGAGC

AGCTGGCCCGCCAGTCCAGGATCAACTATACAGTCGTGGAAGGTTCGACCATCCACCAGTACTTCATCAATATGAAGTTT

GCTGAGGACACCTTGTACAGGGTATGGAAGGAGATAACCCTGAACGCCACTTCCGACCAATCACAGTATAGGGTTTGGGA

TTATCCTATCAGAGAACAGTACGGGCATATACTGCTGGCTATCAACGCTTCTATTCCTGTATCGGACGCTAAGACAGGGT

TTAGACAAGTGGACGAACACACCGACGCCGATTTCGCATTCATACACGACTCTGCCGAGATCAAATACGAAGTGACCCTG

AACTGCAACCTGACGGAGGTGGGCGACGTGTTCGCTGAGCAGCCGTATGCCATCGCCGTGCAGCAGGGCTCCAGGTTACA

GGAGGAGCTGTCGCGAGCACTGCTGGACCTGCAGAAGGAGAGGATGCTGGAACAGCTCGCTGCTAAATACTGGAACGAGA

CGGCCAGACAGCAGTGCCCTGATGCGGACGAGTCAGAAGGCATCACTTTGGAGAGTTTGGGAGGTGTATTCATAGCAACT

CTCTTCGGCTTGGGTCTCGCCATGATAACCCTCGCGTGGGAGGTGTTCTACTACAAACGGAAGGAAAAGAACAAGGTGCA

AGTTCTGGAGGACACGGAGAAGAAACCTAAGAAGGCTTTTGAGAAGGATTTAGATAAGAAGATTAGTGATGGTGTTGCCA

GGCTCAGAAAGGGANAAAAAAAGAAAGCAGGAGTTACAATAGGGGACACCTTCAAACCTGTCTCGGACAAAGAAGGCGTT

TCTTACATTAGTGTGTACCCCAAAAATGAATTTAAGCCGTGA

>Cobl_IR21

ATGATACTAACAATAGTTCTATTAAAAGTTGTCATACTTCATCAAGCAGCAAGTCAAGATGTTGAATTCTATCCTTCACA

GTCTTTACTGAATAAAGTCGACAGAAAAATACTTCCTAAAAGACATAAACTTAAATTTGATGTAAAATCTAAAATATATC

TTGAAAGCCACTTTGAATATAAAAGGGAAGTACAGTGGAGGAAATTTCATAAGGACGATGCAGATGACGTGCCTAAAAAC

AATACCCAAAAGAGAGCTGTAGACCCGGTCTTTCATGGCCATCCAAAAACAAGGGAAGAGCTATGGAACGAGCGTTTTAT

AAACACAAGCTCGACTTTCGATCAAACGCCGTCATTAATAGGCTTGATACGTAATATAACCCTAACCTATCTCACCGATT

GCATCCCAGTCATACTATACGACAGTCAGGTGAAGTCCAAAGAAAGTTACTTATTTCAGAATCTTCTGAAAGATTTCCCG

ATAGCCTTTGTACATGGGTACATCAACGAAGATGACGAAGTTGAGGAGCCAAAGCTTCTTCGTGCTACAAAAGACTGTGT

TAATTTCATTGCATTTTTCTCTGATGTCACTAAAAGTGCAAAGATTTTAGGGAAACAGGCGGAAAGCAAAGTGGTCATAG

TGGCTAGGTCCTCGCAATGGGCCGTGCAAGAATTCCTTGCTGGGTCACAGTCTAGAATGTTCATCAATTTGCTTGTTATT

GGACAAAGCTTTACAGACGGCGAAGATGATTCCTTGGAAGCGCCTTACATCATGTATACTCACAAGCTGTATACAGATGG

CTTAGGAGCTAGTCGGCCAGTAGTTCTAAATTCCTGGTCTCACGGCAAGTTTTCGAGACCAGTGAACTTATTTCCACCTA

AAATGGCTCAAGGATATGCAGGCCACAGATTTGTAGTAGCTGCAGCGCATCAACCACCTTTTATATTTCGAACAATAAAG

ACTGATCTCGACGGAGGCAACCCAAGAGTTGCGTGGGAAGGAGTCGAAATGAGGCTGCTGGCATTATTAGGTGAAAGGAA

CAATTTCTCCATCGAAGTTAAAGAACCTCAAGATTTGCATTTGGGGTCAGGAGATGCAGTAGCAAGGGAAATTAAATCTG

GTCGAGCGGACATAGGAGTGGCTGGTATATATTTAACCAGCGAGAGAACCAGGGACATGGACATGAGCCTCCCTCATTCA

CAGGACTGCGCTGTCTTTGTCACACTCATGTCGACAGCCTTGCCTAGGTATCGAGCAATACTCGGTCCATTTCATTGGCA

CGTCTGGCTGGCTCTCACTCTGACATATCTTTTCGGCATATTTCCCCTGGCTTTCTCAGACAAACATACTTTGAGGCACT

TGTTGCATAACAGTGGAGAAGTCGAAAATATGTTCTGGTACGTTTTCGGAACATTCACCAACTGCTTCACATTTGTTGGG

AAAAACTCTTGGAGCAAAACTACGAAGATAACGACTAGGCTTCTAATCGGTTGGTACTGGGTCTTTACTATAATCATAAC

AAGTTGCTACACCGGTTCCATTATTGCGTTCGTGACGTTACCGGTGTTTCCTGAAACGATTGATAGCATACAACAATTGC

TAGATGGATTTTATCGAGTTGGTACTTTAGATCGAGGCGGTTGGGAGAAATGGTTCCTCAACTCTTCGGATCCCCAAACG

AATAAACTCTTGAAGAAACTGCAGCTGGTGTCAGACGTGGCATCAGGCATTAGGAACACAACGAAGGCATTCTTCTTGCT

ACCCTTTGCCTACCTAGGTTCACAGGCAGAGCTTGAATACATCATTCAATCGAATTTCACTCTAAGTAAAAAAAGCAAAA

GAGCAACGTTACACATCTCCAACGAATGCTTTGTACCGTTCGGTGTATCTTTAAGTTTCCCGAACAACTCCATCTATTCG

GCAAAGTTAAGTGGCGACATTGCTAGAATGGTGCAGAGCGGGCTAGTGAAGAAAATCGTGGATGAAGTGAGATGGGAAAT

GCAACGAAGTGCCACCGGAAAACTACTAGCTGTATCTTCAGGATCAATTAACATTGTATCAGTTGAAGAGAAAGGATTAA

CTTTAGCTGATACACAAGGCATGTTCCTACTTCTAGCTGCAGGTTTCATTCTAGGAGCTTCAGCACTAATATCTGAATGG

ATGGGTGGTTGTTCTAGGAAATGTCGTTTGAGAAAGAAAGAAGATACTCCATCTAGTGCAAATTCTAGAGAACACTTGGT

ATTAACACCTAAAACAGATATTGAATCTGAAATTAAAGTTATATCTGATAGTGCTGACAGTAGATTTCATCTGAACCCTA

GGACTGCTAGCGCAGATTCTAAAAGCAGTCTGGATGGTGCAATTATTAATTTAACTAAGGACAATATTAGTATACATGAT

AACTTTTCCGTTGATGGTTGGGGTTCTAGAAGGTCCAGTTCAGTCGACATTGATAGAGAAGTCAAAGAGATATTTGAGAA

AGATGAAAACAGAAGGAGGGTTAAGTCTACAGCGGGTGTGGAGTTAACAGATAGCCAAAGGCAAGCAACAGCTTCTAAAG

GGGCCTTTGGTGCACATCTATCTGATCCTTAA

>Cobl_IR25

ATGTCTTCACTCACCATCTTGCTTCTATTCCTGTTCGTTCCAGTTACGCTTTCTCAGACGACGCAAAATATAAACGTCCT

GCTTATCAATGAAGAGAATAACGCGTTAGCTGAGAAATCTTTCGAAGTAGCGAAGGAGTATGTTCGGAGAAATCCAACTC

TCGGGTTGGCCGTTGATCCTGTGATTGTGGTAGGCAACAGAACTGACGCTAAGGCTTTTTTAGAGAATGTTTGCAGAAAA

TATAACGACATGTTGTCAGCGAAGAAAACTCCCCACGTCGTCCTCGATTTTACAATGACCGGCGTCGGCTCGGAGACCAT

AAAGTCGTTTACTGCAGCATTGGCCCTGCCCACTATTTCGGGCTCGTTCGGGCAGGCCGGTGACTTGCGCCAGTGGAGGA

ACCTGAATGCAAACCAAAGCAAGTTTCTTCTGCAAGTGATGCCGCCAGCTGACATCCTTCCAGAATCTATCAGAGCCATA

GTTACTAAGCAAGATATTACAAACGCTGCCATTATTTTTGATGAGTTCTTTGTCATGGACCATAAATACAAATCGCTCCT

GCAAAACATCCCGTGCAGGCACGTCATAACGCCGATCAAGAGTTTTAACAAAGACGAAATTAAGACGCAACTGAGAAGTT

TGAGGGAACTGGACATTGTCAACTTCTTCGTGGTTGGGAGTCTACGGACTATAAAGAATGTTCTAGACGCAGCGGACGAG

AATCAGTACTTTGGGAGGAAAACTGCGTGGTTCGCTTTATCTCTGGACAAAGGGGATATAAGTTGCGGGTGTAAAGATGC

CACCGTAGTTTACATGAAACCGACCCCTGACGCAAAGAGCAGAGATCGCCTCGGCAAAATCAAAACAACGTACAGCATGA

ACGGCGAACCTGAAATCACATCTGCGTTCTACTTCGACCTCTCCCTAAGAACGTTCCTGACAGTCAAATCACTGCTGGAC

TCTGGCAAGTGGCCAAATGATATGCGATATATTACTTGCGACGACTACGACGGCAAGAATACCCCCAACCGGACCTTGGA

TCTGAAATCGGCATTTCAAGAGATAAAAGAAACACCGACGTACGCGCCGTTTTTTATTCCCGAAGACGATCCAATGAACG

GGAGGAGTTACATGGAATTTAGTACCGATCTGACGGCAGTTACCGTGAAGGACGGCGCGTCTATAGGTAGCCGCTCGCTC

GGTTCCTGGAAGGCAGGCCTCGCGAACCCGCTGTCGTTGACCGATCCGGAAAATATGAGCGATTACTCTGCCCAGCTCGT

GTTTAGAGTGGTCACAATAGAGCAAAACCCCTTTATTATAAGGGACGACGACGCGCCGAAGGGGTATAAAGGTTACTGTA

TCGATCTTATAGAGGAAATACGTCAAATTGTGAAGTTCGACTACGAAATAACGTTAGCGCCTGACGGTAATTTCGGGACT

ATGGACGACAACGGTAACTGGAACGGGATTATCAAGGAGCTCATCGAGAAACGGGCGGACATCGGTCTGACGTCATTGTC

AGTGATGGCAGAGCGAGAAAACGTGGTAGACTTTACTGTGCCCTACTACGATTTAGTAGGGATCACTATCCTCATGAAAC

TGCCCCGAACACCGACCTCCCTTTTCAAATTTTTAACGGTCTTAGAGAACGATGTCTGGCTGTCCATATTGGCTGCATAT

TTCTTTACTAGTTTCCTCATGTGGGTATTCGACAAGTGGAGTCCGTATAGTTACCAGAATAACCGCGAAAAGTACAAAGA

TGACGAAGAAAAACGCGAATTCAACCTAAAGGAGTGTCTATGGTTTTGCATGACGTCACTCACCCCCCAGGGAGGGGGGG

AGGCGCCCAAAAACCTCTCTGGCCGGTTACTGGCCGCGACTTGGTGGCTGTTTGGGTTTATCATCATAGCGTCATACACT

GCTAATTTGGCGGCGTTCCTTACGGTGTCAAGACTGGACACGCCTATAGAGTCCTTGGATGACCTGTCAAAGCAATACAA

GATTCAGTACGCCCCGCTCAACGGGTCTGCTGCGATGACGTACTTTGAACGCATGGCGCATATCGAAGTCAGATTTTACG

AGATATGGAAAGAGATGAGTCTAAACGACAGCTTGAGCGACGTGGAGCGAGCAAAGCTAGCGGTCTGGGACTACCCCGTC

AGCGACAAGTATAGCAAGATGTGGCAGGCTATGAAGGAGGCTGGGCTGCCTAATTCTGTGGAGGAGGCTGTTCAGAGGGT

GCGAGACTCCAAGAGCTCAAGCGAGGGCTTCGCCTGGCTCGGCGACGCGACGGACGTGCGCTACCACGTGCTCACCAGCT

GCGATCTGCAGATGGTAGGAGACGAGTTCTCGCGGAAGCCTTACGCCATCGCCGTGCAACAGGGGTCTCCATTGAAAGAC

CAGTTTAACAATGCGATCCTCCAACTCCTGAACAAACGGAAGCTAGAGAAGCTCAAGGAAAACTGGTGGAACAACAATCC

TGAAGCAATGAAGTGCGAGAAGCAGGACGACCAGTCAGACGGGATCTCCATCCAGAATATTGGCGGCGTGTTCATTGTCA

TCTTTATGGGAATTGGATTGGCTTGCATTACGCTGGGGGTGGAGTATTGGTGGTACAAGTGGCGGAAAAGGCCGTTGGTT

GGTGATGTTACGCAGGTGGAACCCTCAAAAACAACAAGAAACAACGCTGACCATGGAACCACAAAAATTGGTGACGGATT

CACATTTAGATCAAGGAATTTAGGTCTTTCTAACCTAAGATCCAAGTTCTAA

>Cobl_IR41

ATGTTAGTACCAAGCACGTTACATCCGGTAGAAACATTACTCCAAATTTTAATAAATAAGTACTTGCTGGTATCGTATTG

CGTAACGTTAGTTACGGAAACAGAATTAACATTCCGTCCGCCATCAAACCTAAGCTTCATGTATATTCATCCCGAGTCCA

ACCTCACGGATCAACTCTTGGATGCATCAGAAAAAGGCTGCTCCGATTTCATTATACAAATGAACGAACCTGAGATCTTC

ATGGCTGCTTTTGAAATGACTAACCATTTGGGAGACATAAGAAGAAGCGACAGAAAGTTGATATTCTTACCTCTACAAGA

TAAGGTTTTCAACGGTAGTGCTCTTGATATTTTGGGGCTCAAGGTGACAGGATTCGTAGCAAACATTTTGCTAGTTCTAC

CATCGGCGAATTGCCCAGAAGACTGCGATTACTATGACTTAGTGACGCATAATTTCGTTGGCCCGGATGCAGAGGTCGAT

CAGCCTCTTTACTTAGCCCGATGGGACTTTGGCCTAGAACAGTTCGACGAGGGCGTCAATTTGTTTCCTCATGACATGTC

TAACATGAACAGCAAGACGGTTAAGGTAGCTGCTTTCACGTATAAACCGTACGTATTGCTGGATCTTGACCCGTCAGTCA

ATGCCTTTGGTCGCGATGGTATAGAAATGAGGATCATTGAAGAATTTTGCAGATGGGTAAACTGTACCGTGGAAATTGTA

AGAGACGACGAAAACGAGTGGGGCGAAATATATGACAATCTCACAGGGGTGGGGATTTTAGGTAACGTCGTCGAAGATCG

AGCCGATATGGGAATCACGGCTCTGTATTCGTGGTATGAGGAATACAGAGTGATGGATTTTTCGGCTGCAGTTGTTAGAA

CGGCTATAACTTGCATCGCACCTGCAGCCAGGATCCTATCGAGCTGGGATCTACCATTCCTGCCTTTCGCTGGGTTGATG

TGGCTTTGCCTCATTTTCACATTCTTCTATGCTGCCTTTGCTCTGTTCATAGCCCAGCGCTCAACCGACAAGATATTTTT

ATCGACATTCGGGATGATGATTACACAAACACGAGATGACTCCGGCGACTCGTGGCGCATACGCAGCATCACTGGCTGGT

TGCTAGTGACCGGTCTAATCATAGACAACGCATACGGGGGTGGCTTGGCTTCCAGCTTCACCGTGCCCAAGTACGAAGCC

TCCATAGACACGGTCCAGGATATGGTGGACCGGAAGGTGGAGTGGGGCGCCACTCATGATGCTTGGATATTTTCTATTAT

TTTATCAGAAGAGCCTCTTGTAAAGAAATTAATCAATCAGTTTAAGACGTATCCCGCTGAAGAATTGAAACGGAAGAGTT

TTACACGGAGCATGGCTTTCAGCGTTGAGCATTTGCCGGCAGGTAGCTTCGCAATCGGCGAGTACATAACACAAGAAGCG

ACAGAGGACCTAGAGCTGATGCTAGAGAACTTCTACTACGAGCAGTGCGTGGTGATGCTGCGCAAGAGTTCCCCGTACAC

CGCCAAGCTGAGCGAGCTGGTGGGCCGGCTCCACCAATCAGGGCTCTTGCTGGCGTGGGAGACACAGGTCGCTTTAAAAT

ATCTCGATTTCAAAGTACAACTAGAAGTTAAACTCTCACGCACCAGACGAGATGTGGACGAAGTTAAACCATTGAGTTTA

AAACAAATCCTGGTGGGTATCTTCATAATATACTTCGGTGGTTTGTTCATATCTTTAGTTTGTTTCCTGGTTGAATTAGT

TACAAGATGCGGGAAAACTTCCATTGTAATATAA

>Cobl_IR68

ATGTCTCTGTGGTCAGCCGGGACATTAGGTTTCTTTGATCAGTTAGTTGTGGTTGGGAATATGTTGAAATGTTTCGTTTT

CGTCGTTTTGGTTGTATTCTGCGTCTACGCAGAGATATCCCCCATAATAAAAGACATGGAAGCTCGCAAGGACCTCGAGT

CAGTGCTCATAGACCTGCTGAGCGGCCTGTCTCGCCGCCGCGACGTGGCGTGCGCGGCGATCGTATGCGACGCGGTCTAC

TTGAACGTGTTCGAGGGCGCGCTGTTCAAGCGCACGCTAGATGTCTCTGTGGTTATGATAGTAGTAGAAGAATACGAAGA

CCTGCTATCTCCAAACTTCTACACGCTGGAGTCGCTGCGGCAGGCGAGGAAAGACGGGTGCAACGTTTATATCATTTTAT

TAGCTAATGGCCTGCAAGCGACACGATTGCTTAGATTCGGTGACAGGCATCGAGTCCTAGACACCAGAGCAAAATACATA

ATGCTTCACGACTTCAGACTCTTCCACAGCGACCTTCACTACATATGGAAGCGTATCGTCAACGTAGTCTTTCTCCGATA

CCATAAGAAGATACACGGGTCAGCGAAGGGCAAAGCCTGGTTCGACCTGTCCACTGTACCGTTTCCGAACCCTATAAAGA

CCATATTTGTGTCGAGAAGAGTGGATATATGGAGGAATGGGAGATTTCATTATAACAGACCGCTGTTTGCAGATAAAACG

AAGAATCTAAACGGCGAAGTGCTGAATGTAGTGTACTTAGACCACGTGCCTTCTGTTGTTGTTGTTATGAAAAACAACGA

CACAAACAAAATCGGTGGAGTTGAAATTGAAATTCTTAACACCGTAGCCGAGAAGATGAATTTCAAACCGCATCTGTACC

AAGCAATGAATGCTGAATACCATAAGTGGGGGCAGAAGCAAGCTAACGGGTCCTTTTCAGGTCTTCTCGGAGAGATGGTG

CACGGCCAAGCAGACGTGGCGCTGGGCAACCTGCAGTATACCCCGTACCACCTGGAACTGCTGGACCTCAGCATCCCCTA

CACGTCGCAGTGCTGGACCTTCCTCACGCCCGAGGCGCTCACCGACAACTCCTGGAAGACGCTGATATTGCCGTTTAAAC

TGTACATGTGGATAACCGTCCTGCTGGTGTTGCTGATAACGGGCGCCATTTTCTACGGCCTGGCGAGGTTCTACATGCAC

CTCCTGCAGTATCAGAGCGACCATAGTCCTGTAGTCGTAACTGACAAGGAAGAAACAGATGAATATTTCGACGAGAAACC

CGTCGGCATGTACCTGTTTGGCGATATAATTAACAGCATACTGTACACATACGGAATGTTGCTAGTAGTTTCCCTTCCAA

AGTTACCCACTGGCTGGGCTATCAGACTTCTCACGGGTTGGTACTGGCTTTACTGCATACTACTGGTAGTTTCTTACCGA

GCTAGTATGACCGCGATCCTAGCCAATCCAGCTCCTAGAGTCACCATAGACACGCTACAAGAACTAGTGGATAGCAAAGT

GACCTGTGGCGGCTGGGGTGCTGAAACGAAGCATTTCTTCGAAGAGTCTATAGACGAGATCGGACAGAAGATCGGCGAAC

GCTTCGAAATGATTGATGATCCTGATGAAGCAGCTAACAAGGTAGCCCAGGGAGTTTATGCCTATTACGAGAACGAGTAT

TTTTTGAAGTATTTGAGCGTGAAGCGGAAGAATTCGGATGAGAAAATGAATATAGAAAGCCAAAATTCGACCAATGCAAC

CGTTCAGATCAAACAAGAGTCTGATAGGAACTTGCATATCATGACGGATTGTGTAGTCAACATACCTATATCACTAGGTT

TTCATAAGAACTCTCCTTTGAAACCATTAGCTGATGTCTATATGAGAAGAACAGTAGAAGTAGGCCTGGTGGGAAAGTGG

ATGAATGATGTCATGTACCCTTTAAGAGCTTTGGACGCTACGGACAATGAAATCAAAGCTCTCATGAACCTGAAAAAACT

GTATGGAGCTTTTATAGCTTTAGCTATCGGTTATTTTCTAAGCTTAGTTTGTTTAATTGGCGAACTTATTTACTGGAATT

GTATTGTGAAACGAGATCCTAGATTCGACAAGTATGCGATGGACCTCTATTATGAAAAGAAAAAATAG

>Cobl_IR75b

ATGAAGACTTTCTTTTTATTAAACATTGCTTCATGCTTGCGTTTAGTATTTAGTTTGGGCAGCGACGATATTAACATGAT

AATCTCATTTTCCAAACAAGATGGTAGACCTACGTCTGTTCTCGCACCTCACGTTTGCTGGAGCTCATACGAACTGGCCT

CCTTGGCGAAGAGTCTCCACGAGGTCGGCGTGAGCGTGGCCGGGTCCCTGCAGCCGCGCCGGACGGAGTACTGCCTGCAG

AACCTGCTCATCCTGGCCGACCTTGGCTGCAGAGGGACTGAGGAGTTCCTAGTAAAGGCAAGCGACGAAGGTTTCTTCAA

GTCTCCGTACCGCTGGCTGCTGGTGACCAAGGATGCAGACGACCTGGACATTCTGGAGCGTCTCGTCATGCTCACGGACA

GCGATGTGGTCCTGGCGCAGAAGACCAATGACAGCTATGAGCTGGTAGAAGTGTACAAAATCATCGCCAACTCCGAACTG

ATTTTCAATACTCGAGCTGTGTGGCGAGCCAGCAACGGAACTATCGCAAATACGACAATAACAGACGGCAACGATACGGC

TCTTGCCACCAACGACAAAATCACCAGTATGACTGCAGTGGAAGACAAATATGGAGTTCTAGAAGACCATAGAGCGAGCA

AAGTTCTGTCGACCAGGAGAATGGACATAAGGAAGCACACGCTGACCATGTTGAATGTGATAACGGACAGCAACGACACG

ATGAAGCACATGGCTGATAGATTGCAACTCCACCAGGACTCCATAACCAAGATGACGTGGATGGTGATAAGGATCTGCTT

CGAGATGATGAACTCGACGGAGAAGATGGAATTCTACAACACCTGGGGATACGTTGACAAGAATGGGTCGTGGAACGGGA

TCGTAGAGCAGTTCATCAAGAAACAAGGGGACTTGGGAACGCAAATGCTTTTCACTCAAGCACGCATGGAACTCATCGAC

TATGTCGCGATGGTCGGTACAACGGGTATCCGATTTGTTTTTAGGGAGCCGCCACTTGCCTACGTGTCCAACATATTCGC

GTTGCCCTTCGCCGGCTCAGTGTGGTTGGCGAGCTTTGCGTGTGTGCTTGCGTGCGCTCTGTTCCTATACGTCACGTCCA

AATGGGAAGCCACCATGGGAGTGCACCCTACGCAACTTGACGGGTCGTGGGAAGACGTACTGATCCTGATAATTGGCGGT

GTGCTGCAGCAGGGGTGCATTCTGGAGCCGAGGTTCGCAGCAGGTCGTATGGTAACTCTTCTGCTGTTCATCGCCCTCAC

GATTCTGTACGCGGCTTACACGGCCAACATCGTGGTGCTCCTCAGGGCTCCAAGTTCCTCGGTCAAGAATCTCCAGGACA

TGCTGGACTCTCCTCTGGAACTGGGCGCCAGCGACTTCAATTATAATAGATATTTTTTTAAGCAATTGAACGAGCCGCTG

CGCAAAGAGATATATAACAAGAAGATTGCTCCGAAAGGAAAGAAGGCCAACTTTTACACAATGGAGGAGGGGGTGGAGAA

GATACGACGGGGTCTATTCGCCTTCCACATGGAGCTGAACCCCGGATACCGCCTCATCCAGGAGACTTACCAGGAAGAGG

AGAAGTGTGACCTGGTGGAGATGGACTACATCAGCGAAATAGATCCCTGGGTCCCGGGACAGAAGAGATCGCCATACAAG

GATCTGTTCAAGATCAATTTCATAAAAATCCGCGAAGTCGGCCTCCAATCCGCCATCCACCAGCAGATACAGGTTGGCAA

GCCGCGCTGCCAGGGCGAGGTCAGCACGTTCAGCAGCGTCGGCATCACTGACATGTACCCAGCCATGCTGGCCACACTGT

ACGGCGTGCTGCTCGCGCCTGCCGTGCTGCTGCTCGAGCTGGCATATCACCGGCTGATTTTGATTAGAAAAAAAAGACTA

GCGAAGTTTCAATGTGAACAAGACACCACATAA

>Cobl_IR75c

ATGAAAAAGTACTATTTAATGGTGTCACTAATAATATTTTTTTCAGGTTGTTACGCGGAAAATGATTCAAAGCTATTTAT

GGTAGCCGACGTCATCCGTTCTATGCAAAGGCCGTCGTCCGTGATCGCCATGCTTTGCTGGTCTTCACGTATGAAGATAC

AACTATATTCGGCCCTGGGAGAAAATGACACGCATATTAATATGATGCAGGTTTTGAAAAGTGGGACAGTGCCTCAGAGG

CATGCTCAGGATCAACATATTGTGTTCTTAGCTAACTTGGACTGTCCTGGAATATCTTCGTACTTTGCTATGAGCAGTTC

AGAAAAGCATTTTCGATCTCCCTTTCGTTGGCTTCTTGTCGGCACTGACTACAATGACACCGGAGTGGAGGGAAGCTATA
[truncated: 685,539 more chars]
